# ESICM LIVES 2016: part three: Milan, Italy. 1–5 October 2016

**DOI:** 10.1186/s40635-016-0100-7

**Published:** 2016-09-29

**Authors:** T. Velasquez, G. Mackey, J. Lusk, U. G. Kyle, T. Fontenot, P. Marshall, L. S. Shekerdemian, J. A. Coss-Bu, A. Nishigaki, T. Yatabe, T. Tamura, K. Yamashita, M. Yokoyama, J. C. Ruiz-Rodriguez, B. Encina, R. Belmonte, I. Troncoso, P. Tormos, M. Riveiro, J. Baena, A. Sanchez, J. Bañeras, J. Cordón, N. Duran, A. Ruiz, J. Caballero, X. Nuvials, J. Riera, J. Serra, A. M. F. Rutten, S. N. M. van Ieperen, E. P. H. M. Der Kinderen, T. Van Logten, L. Kovacikova, P. Skrak, M. Zahorec, U. G. Kyle, A. Akcan-Arikan, J. C. Silva, G. Mackey, J. Lusk, M. Goldsworthy, L. S. Shekerdemian, J. A. Coss-Bu, D. Wood, D. Harrison, R. Parslow, P. Davis, J. Pappachan, S. Goodwin, P. Ramnarayan, S. Chernyshuk, H. Yemets, V. Zhovnir, S. M. Pulitano’, S. De Rosa, A. Mancino, G. Villa, F. Tosi, P. Franchi, G. Conti, B. Patel, H. Khine, A. Shah, D. Sung, L. Singer, S. Haghbin, S. Inaloo, Z. Serati, M. Idei, T. Nomura, N. Yamamoto, Y. Sakai, T. Yoshida, Y. Matsuda, Y. Yamaguchi, S. Takaki, O. Yamaguchi, T. Goto, N. Longani, S. Medar, I. R. Abdel-Aal, A. S. El Adawy, H. M. E. H. Mohammed, A. N. Mohamed, S. M. Parry, L. D. Knight, L. Denehy, N. De Morton, C. E. Baldwin, D. Sani, G. Kayambu, V. Z. M. da Silva, P. Phongpagdi, Z. A. Puthucheary, C. L. Granger, J. E. Rydingsward, C. M. Horkan, K. B. Christopher, D. McWilliams, C. Jones, E. Reeves, G. Atkins, C. Snelson, L. M. Aitken, J. Rattray, J. Kenardy, A. M. Hull, A. Ullman, R. Le Brocque, M. Mitchell, C. Davis, B. Macfarlane, J. C. Azevedo, L. L. Rocha, F. F. M. De Freitas, A. M. Cavalheiro, N. M. Lucinio, M. S. Lobato, G. Ebeling, A. Kraegpoeth, E. Laerkner, I. De Brito-Ashurst, C. White, S. Gregory, L. G. Forni, E. Flowers, A. Curtis, C. A. Wood, K. Siu, K. Venkatesan, J. B. H. Muhammad, L. Ng, E. Seet, N. Baptista, A. Escoval, E. Tomas, R. Agrawal, R. Mathew, A. Varma, E. Dima, E. Charitidou, E. Perivolioti, M. Pratikaki, C. Vrettou, A. Giannopoulos, S. Zakynthinos, C. Routsi, E. Atchade, S. Houzé, S. Jean-Baptiste, G. Thabut, C. Genève, S. Tanaka, B. Lortat-Jacob, P. Augustin, M. Desmard, P. Montravers, F. J. González de Molina, S. Barbadillo, R. Alejandro, F. Álvarez-Lerma, J. Vallés, R. M. Catalán, E. Palencia, A. Jareño, R. M. Granada, M. L. Ignacio, GETGAG Working Group, N. Cui, D. Liu, H. Wang, L. Su, H. Qiu, R. Li, K. Jaffal, A. Rouzé, J. Poissy, B. Sendid, S. Nseir, E. Paramythiotou, M. Rizos, F. Frantzeskaki, A. Antoniadou, S. Vourli, L. Zerva, A. Armaganidis, J. Riera, J. Gottlieb, M. Greer, O. Wiesner, M. Martínez, M. Acuña, J. Rello, T. Welte, E. Atchade, T. Mignot, S. Houzé, S. Jean-Baptiste, G. Thabut, B. Lortat-Jacob, S. Tanaka, P. Augustin, M. Desmard, P. Montravers, S. Soussi, E. Dudoignon, A. Ferry, M. Chaussard, M. Benyamina, A. Alanio, S. Touratier, M. Chaouat, M. Lafaurie, M. Mimoun, A. Mebazaa, M. Legrand, M. A. Sheils, C. Patel, L. Mohankumar, N. Akhtar, S. K. Pacheco Noriega, N. Navarrete Aldana, J. L. Ávila León, J. Durand Baquero, F. Fernández Bernal, E. Ahmadnia, J. S. Hadley, M. Millar, D. Hall, H. Hewitt, H. Yasuda, M. Sanui, T. Komuro, S. Kawano, K. Andoh, H. Yamamoto, E. Noda, J. Hatakeyama, N. Saitou, H. Okamoto, A. Kobayashi, T. Takei, S. Matsukubo, JSEPTIC (Japanese Society of Education for Physicians and Trainees in Intensive Care) Clinical Trial Group, H. B. Rotzel, A. Serrano Lázaro, D. Aguillón Prada, M. Rodriguez Gimillo, O. Diaz Barinas, M. L. Blasco Cortes, J. Ferreres Franco, J. M. Segura Roca, A. Carratalá, B. Gonçalves, R. Turon, A. Mendes, F. Miranda, P. J. Mata, D. Cavalcanti, N. Melo, P. Lacerda, P. Kurtz, C. Righy, L. E. de la Cruz Rosario, S. P. Gómez Lesmes, J. C. García Romero, A. N. García Herrera, E. D. Díaz Pertuz, M. J. Gómez Sánchez, E. Regidor Sanz, J. Barado Hualde, A. Ansotegui Hernández, J. M. Guergué Irazabal, V. Spatenkova, O. Bradac, P. Suchomel, T. Urli, E. Heusch Lazzeri, R. Aspide, M. Zanello, L. Perez-Borrero, J. M. Garcia-Alvarez, M. D. Arias-Verdu, E. Aguilar-Alonso, R. Rivera-Fernandez, J. Mora-Ordoñez, C. De La Fuente-Martos, E. Castillo-Lorente, F. Guerrero-Lopez, S. P. Gómez Lesmes, L. E. De la Cruz Rosario, E. D. Díaz Pertuz, A. Ansotegui Hernández, J. C. García Romero, M. J. Gómez Sánchez, A. N. García Herrera, J. Roldán Ramírez, E. Regidor Sanz, J. Barado Hualde, J. P. Tirapu León, L. Navarro-Guillamón, S. Cordovilla-Guardia, A. Iglesias-Santiago, F. Guerrero-López, E. Fernández-Mondéjar, A. Vidal, M. Perez, A. Juez, N. Arias, L. Colino, J. L. Perez, H. Pérez, P. Calpe, M. A. Alcala, D. Robaglia, C. Perez, S. K. Lan, M. M. Cunha, T. Moreira, F. Santos, E. Lafuente, M. J. Fernandes, J. G. Silva, L. E. de la Cruz Rosario, S. P. Gómez Lesmes, A. N. García Herrera, J. C. García Romero, E. D. Díaz Pertuz, M. J. Gómez Sánchez, E. Regidor Sanz, J. G. Armando Echeverría, A. Ansotegui Hernández, J. Barado Hualde, V. Podlepich, E. Sokolova, E. Alexandrova, K. Lapteva, P. Kurtz, C. Shuinotsuka, L. Rabello, G. Vianna, A. Reis, C. Cairus, J. Salluh, F. Bozza, J. C. Barrios Torres, N. J. Fernández Araujo, P. García-Olivares, E. Keough, M. Dalorzo, L. K. Tang, I. De Sousa, M. Díaz, L. J. Marcos-Zambrano, J. E. Guerrero, S. E. Zamora Gomez, G. D. Hernandez Lopez, A. I. Vazquez Cuellar, O. R. Perez Nieto, J. A. Castanon Gonzalez, D. Bhasin, S. Rai, H. Singh, O. Gupta, M. K. Bhattal, S. Sampley, K. Sekhri, R. Nandha, F. A. Aliaga, F. Olivares, F. Appiani, P. Farias, F. Alberto, A. Hernández, S. Pons, R. Sonneville, L. Bouadma, M. Neuville, E. Mariotte, A. Radjou, J. Lebut, S. Chemam, G. Voiriot, M. P. Dilly, B. Mourvillier, R. Dorent, P. Nataf, M. Wolff, J. F. Timsit, O. Ediboglu, S. Ataman, H. Ozkarakas, C. Kirakli, A. Vakalos, V. Avramidis, O. Obukhova, I. A. Kurmukov, S. Kashiya, E. Golovnya, V. N. Baikova, T. Ageeva, T. Haritydi, E. V. Kulaga, J. J. Rios-Toro, L. Perez-Borrero, E. Aguilar-Alonso, M. D. Arias-Verdu, J. M. Garcia-Alvarez, C. Lopez-Caler, C. De La Fuente-Martos, S. Rodriguez-Fernandez, M. Gomez Sanchez-Orézzoli, F. Martin-Gallardo, J. Nikhilesh, V. Joshi, E. Villarreal, J. Ruiz, M. Gordon, A. Quinza, J. Gimenez, M. Piñol, A. Castellanos, P. Ramirez, Y. D. Jeon, W. Y. Jeong, M. H. Kim, I. Y. Jeong, M. Y. Ahn, J. Y. Ahn, S. H. Han, J. Y. Choi, Y. G. Song, J. M. Kim, N. S. Ku, H. Shah, F. Kellner, F. Rezai, N. Mistry, P. Yodice, V. Ovnanian, K. Fless, E. Handler, R. Martínez Alejos, J. D. Martí Romeu, D. González Antón, A. Quinart, A. Torres Martí, M. Llaurado-Serra, A. Lobo-Civico, A. Ventura-Rosado, A. Piñol-Tena, M. Pi-Guerrero, C. Paños-Espinosa, M. Peralvo-Bernat, J. Marine-Vidal, R. Gonzalez-Engroba, N. Montesinos-Cerro, M. Treso-Geira, A. Valeiras-Valero, L. Martinez-Reyes, A. Sandiumenge, M. F. Jimenez-Herrera, CAPCRI Study, S. Helyar, P. Riozzi, A. Noon, G. Hallows, H. Cotton, J. Keep, P. A. Hopkins, A. Taggu, S. Renuka, S. Sampath, P. J. T. Rood, T. Frenzel, R. Verhage, M. Bonn, P. Pickkers, J. G. van der Hoeven, M. van den Boogaard, F. Corradi, L. Melnyk, F. Moggia, R. Pienovi, G. Adriano, C. Brusasco, L. Mariotti, M. Lattuada, M. J. Bloomer, M. Coombs, K. Ranse, R. Endacott, B. Maertens, K. Blot, S. Blot, M. P. van Nieuw Amerongen, E. S. van der Heiden, J. W. R. Twisk, A. R. J. Girbes, J. J. Spijkstra, P. Riozzi, S. Helyar, H. Cotton, G. Hallows, A. Noon, C. Bell, K. Peters, A. Feehan, J. Keep, P. A. Hopkins, K. Churchill, K. Hawkins, R. Brook, N. Paver, R. Endacott, N. Maistry, A. van Wijk, N. Rouw, T. van Galen, S. Evelein-Brugman, A. Taggu, B. Krishna, S. Sampath, A. Putzu, M. Fang, M. Boscolo Berto, A. Belletti, T. Cassina, L. Cabrini, M. Mistry, Y. Alhamdi, I. Welters, S. T. Abrams, C. H. Toh, H. S. Han, E. M. Gil, D. S. Lee, C. M. Park, S. Winder-Rhodes, R. Lotay, J. Doyle, M. W. Ke, W. C. Huang, C. H. Chiang, W. T. Hung, C. C. Cheng, K. C. Lin, S. C. Lin, K. R. Chiou, S. R. Wann, C. W. Shu, P. L. Kang, G. Y. Mar, C. P. Liu, S. Dubó, A. Aquevedo, M. Jibaja, D. Berrutti, C. Labra, R. Lagos, M. F. García, V. Ramirez, M. Tobar, F. Picoita, C. Peláez, D. Carpio, L. Alegría, C. Hidalgo, K. Godoy, J. Bakker, G. Hernández, Y. Sadamoto, K. Katabami, T. Wada, Y. Ono, K. Maekawa, M. Hayakawa, A. Sawamura, S. Gando, H. Marin-Mateos, J. L. Perez-Vela, R. Garcia-Gigorro, M. A. Corres Peiretti, M. J. Lopez-Gude, S. Chacon-Alves, E. Renes-Carreño, J. C. Montejo-González, K. L. Parlevliet, H. R. W. Touw, M. Beerepoot, C. Boer, P. W. G. Elbers, P. R. Tuinman, S. A. Abdelmonem, T. A. Helmy, I. El Sayed, S. Ghazal, S. H. Akhlagh, M. Masjedi, K. Hozhabri, E. Kamali, I. Zýková, B. Paldusová, P. Sedlák, D. Morman, A. M. Youn, Y. Ohta, M. Sakuma, D. Bates, T. Morimoto, P. L. Su, W. Y. Chang, W. C. Lin, C. W. Chen, F. Facchin, F. Zarantonello, G. Panciera, A. De Cassai, A. Venrdramin, A. Ballin, T. Tonetti, P. Persona, C. Ori, L. Del Sorbo, S. Rossi, G. Vergani, M. Cressoni, D. Chiumello, C. Chiurazzi, M. Brioni, I. Algieri, T. Tonetti, M. Guanziroli, A. Colombo, I. Tomic, A. Colombo, F. Crimella, E. Carlesso, V. Gasparovic, L. Gattinoni, A. Serpa Neto, M. Schmidt, T. Pham, A. Combes, M. Gama de Abreu, P. Pelosi, M. J. Schultz, for the ReVA Research Network and the PROVE Network Investigators, B. H. Katira, D. Engelberts, R. E. Giesinger, C. Ackerley, T. Yoshida, D. Zabini, G. Otulakowski, M. Post, W. M. Kuebler, P. J. McNamara, B. P. Kavanagh, R. Pirracchio, M. Resche Rigon, M. Carone, S. Chevret, D. Annane, S. Eladawy, M. El-Hamamsy, N. Bazan, M. Elgendy, G. De Pascale, M. S. Vallecoccia, S. L. Cutuli, V. Di Gravio, M. A. Pennisi, G. Conti, M. Antonelli, D. T. Andreis, W. Khaliq, M. Singer, J. Hartmann, S. Harm, S. Alcantara Carmona, P. Matia Almudevar, A. Naharro Abellán, J. Veganzones Ramos, L. Pérez Pérez, B. Lobo Valbuena, N. Martínez Sanz, I. Fernández Simón, M. Arrigo, E. Feliot, N. Deye, A. Cariou, B. Guidet, S. Jaber, M. Leone, M. Resche-Rigon, A. Vieillard Baron, M. Legrand, E. Gayat, A. Mebazaa, from the FROG ICU Investigators, M. Balik, I. Kolnikova, M. Maly, P. Waldauf, G. Tavazzi, J. Kristof, A. Herpain, F. Su, E. Post, F. Taccone, J. L. Vincent, J. Creteur, C. Lee, F. Hatib, Z. Jian, S. Buddi, M. Cannesson, S. Fileković, M. Turel, R. Knafelj, V. Gorjup, R. Stanić, P. Gradišek, O. Cerović, T. Mirković, M. Noč, J. Tirkkonen, H. Hellevuo, K. T. Olkkola, S. Hoppu, K. C. Lin, W. T. Hung, C. C. Chiang, W. C. Huang, W. C. Juan, S. C. Lin, C. C. Cheng, P. H. Lin, K. Y. Fong, D. S. Hou, P. L. Kang, S. R. Wann, Y. S. Chen, G. Y. Mar, C. P. Liu, M. Paul, W. Bougouin, G. Geri, F. Dumas, B. Champigneulle, S. Legriel, J. Charpentier, J. P. Mira, C. Sandroni, A. Cariou, J. Zimmerman, E. Sullivan, M. Noursadeghi, B. Fox, D. Sampson, L. McHugh, T. Yager, S. Cermelli, T. Seldon, S. Bhide, R. A. Brandon, R. B. Brandon, J. Zwaag, R. Beunders, P. Pickkers, M. Kox, F. Gul, M. K. Arslantas, D. Genc, N. Zibandah, L. Topcu, T. Akkoc, I. Cinel, E. Greco, M. P. Lauretta, D. T. Andreis, M. Singer, I. Palacios Garcia, M. Cordero, A. Diaz Martin, T. Aldabó Pallás, J. Garnacho Montero, J. Revuelto Rey, L. Roman Malo, A. A. Tanaka Montoya, A. D. C. Amador Martinez, L. Y. Delgado Ayala, E. Monares Zepeda, J. Franco Granillo, J. Aguirre Sanchez, G. Camarena Alejo, A. Rugerio Cabrera, A. Pedraza Montenegro, T. Pham, G. Beduneau, F. Schortgen, L. Piquilloud, E. Zogheib, M. Jonas, F. Grelon, I. Runge, N. Terzi, S. Grangé, G. Barberet, P. G. Guitard, J. P. Frat, A. Constan, J. M. Chrétien, J. Mancebo, A. Mercat, J. C. M. Richard, L. Brochard, The WIND study group, E. Soilemezi, E. Koco, S. Savvidou, C. Nouris, D. Matamis, Plug Working Group, R. Di Mussi, S. Spadaro, C. A. Volta, M. Mariani, A. Colaprico, C. Antonio, F. Bruno, S. Grasso, A. Rodriguez, I. Martín-Loeches, E. Díaz, J. R. Masclans, F. Gordo, J. Solé-Violán, M. Bodí, F. X. Avilés-Jurado, S. Trefler, M. Magret, L. F. Reyes, J. Marín-Corral, J. C. Yebenes, A. Esteban, A. Anzueto, S. Aliberti, M. I. Restrepo, GETGAG/SEMICYUC, J. Skytte Larsson, B. Redfors, S. E. Ricksten, R. Haines, J. Powell-Tuck, H. Leonard, M. Ostermann, R. E. Berthelsen, T. S. Itenov, A. Perner, J. U. Jensen, M. Ibsen, A. E. K. Jensen, M. H. Bestle, T. Bucknall, J. Dixon, F. Boa, I. MacPhee, B. J. Philips, AKI Research Group, St George’s University of London, J. Doyle, F. Saadat, T. Samuels, S. Huddart, B. McCormick, R. DeBrunnar, J. Preece, M. Swart, C. Peden, S. Richardson, L. Forni, P. Kalfon, K. Baumstarck, P. Estagnasie, M. A. Geantot, A. Berric, G. Simon, B. Floccard, T. Signouret, M. Boucekine, M. Fromentin, M. Nyunga, A. Sossou, M. Venot, R. Robert, A. Follin, A. Renault, M. Garrouste, O. Collange, Q. Levrat, I. Villard, D. Thévenin, J. Pottecher, R. G. Patrigeon, N. Revel, C. Vigne, O. Mimoz, P. Auquier, IPREA Study Group, S. Pawar, T. Jacques, K. Deshpande, R. Pusapati, B. Wood, R. A. Pulham, J. Wray, K. Brown, C. Pierce, S. Nadel, P. Ramnarayan, J. R. Azevedo, W. S. Montenegro, D. P. Rodrigues, S. C. Sousa, V. F. Araujo, A. L. Leitao, P. H. Prazeres, A. V. Mendonca, M. P. Paula, A. Das Neves, C. I. Loudet, M. Busico, D. Vazquez, D. Villalba, A. Lischinsky, M. Veronesi, M. Emmerich, E. Descotte, A. Juliarena, M. Carboni Bisso, M. Grando, A. Tapia, M. Camargo, D. Villani Ulla, L. Corzo, H. Placido dos Santos, A. Ramos, J. A. Doglia, E. Estenssoro, M. Carbonara, S. Magnoni, C. L. Mac Donald, J. S. Shimony, V. Conte, F. Triulzi, F. Stretti, M. Macrì, A. Z. Snyder, N. Stocchetti, D. L. Brody, V. Podlepich, V. Shimanskiy, I. Savin, K. Lapteva, A. Chumaev, M. C. Tjepkema-Cloostermans, J. Hofmeijer, A. Beishuizen, H. Hom, M. J. Blans, M. J. A. M. van Putten, L. Longhi, B. Frigeni, M. Curinga, D. Mingone, S. Beretta, A. Patruno, L. Gandini, A. Vargiolu, F. Ferri, R. Ceriani, M. R. Rottoli, L. Lorini, G. Citerio, S. Pifferi, M. Battistini, V. Cordolcini, A. Agarossi, R. Di Rosso, F. Ortolano, N. Stocchetti, C. Mora Lourido, J. L. Santana Cabrera, J. D. Martín Santana, L. Melián Alzola, C. García del Rosario, H. Rodríguez Pérez, R. Lorenzo Torrent, S. Eslami, A. Dalhuisen, T. Fiks, M. J. Schultz, A. Abu Hanna, P. E. Spronk, M. Wood, D. Maslove, J. Muscedere, S. H. Scott, T. Saha, A. Hamilton, D. Petsikas, D. Payne, J. G. Boyd, Z. A. Puthucheary, A. S. McNelly, J. Rawal, B. Connolly, M. J. McPhail, P. Sidhu, A. Rowlerson, J. Moxham, S. D. Harridge, N. Hart, H. E. Montgomery, T. Jovaisa, B. Thomas, D. Gupta, D. S. Wijayatilake, H. P. Shum, H. S. King, K. C. Chan, K. B. Tang, W. W. Yan, C. Castro Arias, J. Latorre, A. Suárez De La Rica, E. Maseda Garrido, A. Montero Feijoo, C. Hernández Gancedo, A. López Tofiño, F. Gilsanz Rodríguez, L. K. Gemmell, R. Campbell, P. Doherty, A. MacKay, N. Singh, S. Vitaller, H. Nagib, J. Prieto, A. Del Arco, B. Zayas, C. Gomez, S. Tirumala, S. A. Pasha, B. K. Kumari, P. Martinez-Lopez, A. Puerto-Morlán, P. Nuevo-Ortega, L. Martinez Pujol, R. Algarte Dolset, B. Sánchez González, S. Quintana Riera, J. Trenado Álvarez, S. Quintana, L. Martínez, R. Algarte, B. Sánchez, J. Trenado, E. Tomas, N. Brock, E. Viegas, E. Filipe, D. Cottle, T. Traynor, M. V. Trasmonte Martínez, M. Pérez Márquez, L. Colino Gómez, N. Arias Martínez, J. M. Milicua Muñoz, B. Quesada Bellver, M. Muñoz Varea, M. Á. Alcalá Llorente, C. Pérez Calvo, S. D. Hillier, M. C. Faulds, H. Hendra, N. Lawrence, K. Maekawa, M. Hayakawa, Y. Ono, A. Kodate, Y. Sadamoto, N. Tominaga, A. Mizugaki, H. Murakami, T. Yoshida, K. Katabami, T. Wada, A. Sawamura, S. Gando, S. Silva, L. Kerhuel, B. Malagurski, G. Citerio, R. Chabanne, S. Laureys, L. Puybasset, L. Nobile, E. R. Pognuz, A. O. Rossetti, F. Verginella, N. Gaspard, J. Creteur, N. Ben-Hamouda, M. Oddo, F. S. Taccone, Y. Ono, M. Hayakawa, H. Iijima, K. Maekawa, A. Kodate, Y. Sadamoto, A. Mizugaki, H. Murakami, K. Katabami, T. Wada, A. Sawamura, S. Gando, A. Kodate, K. Katabami, T. Wada, Y. Ono, K. Maekawa, M. Hayakawa, A. Sawamura, S. Gando, L. W. Andersen, T. Raymond, R. Berg, V. Nadkarni, A. Grossestreuer, T. Kurth, M. Donnino, A. Krüger, P. Ostadal, M. Janotka, D. Vondrakova, N. Kongpolprom, J. Cholkraisuwat, P. T. Pekkarinen, G. Ristagno, S. Masson, R. Latini, S. Bendel, T. Ala-Kokko, T. Varpula, J. Vaahersalo, S. Hoppu, M. Tiainen, M. M. Mion, M. Plebani, V. Pettilä, M.B. Skrifvars, FINNRESUSCI Study Group, Y. Son, K. S. Kim, G. J. Suh, W. Y. Kwon, J. I. Ko, M. J. Park, F. Zama Cavicchi, E. Iesu, L. Nobile, J. L. Vincent, J. Creteur, F. S. Taccone, H. Tanaka, N. Otani, S. Ode, S. Ishimatsu, L. Martínez, R. Algarte, B. Sánchez, I. Romero, F. Martínez, S. Quintana, J. Trenado, D. Vondrakova, P. Ostadal, A. Kruger, M. Janotka, F. Malek, P. Neuzil, Y. C. Yeh, Y. S. Chen, C. H. Wang, C. H. Huang, A. Chao, C. T. Lee, C. H. Lai, W. S. Chan, Y. J. Cheng, W. Z. Sun, S. Kaese, C. Horstmann, P. Lebiedz, M. Mourad, P. Gaudard, J. Eliet, N. Zeroual, P. Colson, P. Ostadal, M. Mlcek, M. Hrachovina, A. Kruger, D. Vondrakova, M. Janotka, M. Mates, P. Hala, O. Kittnar, P. Neuzil, A. Jacky, A. Rudiger, D. R. Spahn, D. A. Bettex, A. Kara, S. Akin, D. Dos reis Miranda, A. Struijs, K. Caliskan, R. J. van Thiel, E. A. Dubois, W. de Wilde, F. Zijlstra, D. Gommers, C. Ince, L. Marca, A. Xini, W. Mongkolpun, C. P. R. Cordeiro, R. T. Leite, O. Lheureux, A. Bader, L. Rincon, C. Santacruz, J. C. Preiser, A. Chao, A. S. Chao, Y. S. Chen, W. Kim, C. Ahn, Y. Cho, T. H. Lim, J. Oh, K. S. Choi, B. H. Jang, J. K. Ha, A. Mecklenburg, J. Stamm, G. Soeffker, M. Kubik, K. Sydow, H. Reichenspurner, S. Kluge, S. Braune, B. Bergantino, F. Ruberto, E. Magnanimi, E. Privato, V. Zullino, K. Bruno, F. Pugliese, G. Sales, V. Girotto, F. Vittone, L. Brazzi, C. Fritz, A. Kimmoun, F. Vanhuyse, B. Trifan, S. Orlowski, E. Albuisson, N. Tran, B. Levy, V. Chhor, J. Joachim, A. Follin, B. Champigneulle, J. Chatelon, G. Fave, J. Mantz, R. Pirracchio, D. Díaz Diaz, M. Villanova, M. Aguirregabyria, G. Andrade, L. López, E. Palencia, G. John, R. Cowan, R. Hart, K. Lake, K. Litchfield, J. W. Song, Y. J. Lee, Y. J. Cho, S. Choi, P. Vermeir, D. Vandijck, S. Blot, A. Mariman, R. Verhaeghe, M. Deveugele, D. Vogelaers, L. Chok, E. B. Bachli, D. Bettex, S. R. Cottini, E. Keller, M. Maggiorini, R. Schuepbach, T. Fiks, C. Stiphout, M. Grevelink, I. Vaneker, A. Ruijter, M. Buise, P. E. Spronk, S. Altaba Tena, L. Galarza Barrachina, J. H. Rodriguez Portillo, G. Pagés Aznar, L. Mateu Campos, M. D. Ferrándiz Sellés, M. Arlandis Tomás, A. Belenguer Muncharaz, L. Skinner, S. Monsalvo, E. Olavarria, R. Stümpfle, S. J. Na, J. Park, C. R. Chung, C. M. Park, G. Y. Suh, J. H. Yang, T. Witter, C. Brousseau, M. B. Butler, M. Erdogan, P. C. Mac Dougall, R. S. Green, T. E. F. Abbott, H. D. T. Torrance, N. Cron, N. Vaid, J. Emmanuel, S. S. Siddiqui, N. Prabu, H. K. Chaudhari, V. P. Patil, J. V. Divatia, S. Solanki, A. P. Kulkarni, L. A. Rincon Gutierrez, A. Bader, A. Brasseur, O. Lheureux, J. L. Vincent, J. Creteur, F. S. Taccone, D. Hempel, N. Stauffert, F. Recker, T. Schröder, S. Reusch, J. Schleifer, R. Breitkreutz, F. Sjövall, A. Perner, M. Hylander Møller, R. B. Moraes, F. K. Borges, J. A. V. Guillen, W. J. C. Zabaletta, PICS- HCPA: Programa Intrahospitalar de Combate à Sepse do Hospital de Clínicas de Porto Alegre, J. Ruiz-Ramos, P. Ramirez, M. R. Marqués-Miñana, E. Villarreal, M. Gordon, M. Sosa, P. Concha, A. Castellanos, R. Menendez, C. Sánchez Ramírez, M. Cabrera Santana, L. Caipe Balcázar, S. Hípola Escalada, M. A. Hernández Viera, C. F. Lübbe Vázquez, J. J. Díaz Díaz, F. Artiles Campelo, N. Sangil Monroy, P. Saavedra Santana, S. Ruiz Santana, A. Gutiérrez-Pizarraya, J. Garnacho-Montero, C. Martin, K. Baumstarck, M. Leone, I. Martín-Loeches, R. Pirracchio, M. Legrand, J. L. Mainardi, J. Mantz, B. Cholley, A. Hubbard, P. Ruiz Frontera, L. M. Claraco Vega, P. Ruiz de Gopegui Miguelena, M. C. Villuendas Usón, A. Rezusta López, E. Aurensanz Clemente, P. Gutiérrez Ibañes, A. L. Ruiz Aguilar, M. Palomar, P. Olaechea, S. Uriona, M. Vallverdu, M. Catalan, X. Nuvials, C. Aragon, F. Alvarez Lerma, ENVIN-HELICS Study Group, Y. D. Jeon, W. Y. Jeong, M. H. Kim, I. Y. Jeong, M. Y. Ahn, J. Y. Ahn, S. H. Han, J. Y. Choi, Y. G. Song, J. M. Kim, N. S. Ku, G. Li Bassi, E. Aguilera Xiol, T. Senussi, F. A. Idone, A. Motos, C. Chiurazzi, C. Travierso, L. Fernández-Barat, R. Amaro, Y. Hua, O. T. Ranzani, Q. Bobi, M. Rigol, A. Torres, I. Fuentes Fernández, E. Andreu Soler, A. Pareja Rodríguez de Vera, E. Escudero Pastor, V. Hernandis, J. Ros Martínez, R. Jara Rubio, M. Miralbés Torner, S. Carvalho Brugger, A. Aragones Eroles, S. Iglesias Moles, J. Trujillano Cabello, J. A. Schoenenberger, X. Nuvials Casals, M. Vallverdu Vidal, B. Balsera Garrido, M. Palomar Martinez, L. Mirabella, A. Cotoia, L. Tullo, A. Stella, F. Di Bello, A. Di Gregorio, M. Dambrosio, G. Cinnella, L. E. de la Cruz Rosario, S. P. Gómez Lesmes, J. C. García Romero, A. N. García Herrera, E. D. Díaz Pertuz, M. J. Gómez Sánchez, E. Regidor Sanz, J. Barado Hualde, A. Ansotegui Hernández, J. Roldán Ramirez, H. Takahashi, F. Kazutoshi, Y. Okada, W. Oobayashi, T. Naito, D. K. Baidya, S. Maitra, R. K. Anand, B. R. Ray, M. K. Arora, C. Ruffini, L. Rota, A. Corona, G. Sesana, S. Ravasi, E. Catena, D. N. Naumann, C. Mellis, S. L. Husheer, J. Bishop, M. J. Midwinter, S. Hutchings, F. Corradi, C. Brusasco, T. Manca, A. Ramelli, M. Lattuada, F. Nicolini, T. Gherli, A. Vezzani, A. Young, A. Fernández Carmona, A. Iglesias Santiago, L. Navarro Guillamon, M. J. García Delgado, M. Delgado-Amaya, E. Curiel-Balsera, L. Rivera-Romero, E. Castillo-Lorente, F. Carrero-Gómez, E. Aguayo-DeHoyos, ARIAM registry of adult cardiac surgery, A. J. Healey, C. Cameron, L.R. Jiao, R. Stümpfle, A. Pérez, S. Martin, O. Lopez del Moral, S. Toval, J. Rico, C. Aldecoa, K. Oguzhan, O. Demirkiran, M. Kirman, S. Bozbay, M. E. Kosuk, G. Asyralyyeva, M. Dilek, M. Duzgun, S. Telli, M. Aydin, F. Yilmazer, L. E. Hodgson, B. D. Dimitrov, C. Stubbs, L. G. Forni, R. Venn, D. Vedage, S. Shawaf, P. Naran, N. Sirisena, J. Kinnear, B. D. Dimitrov, L. E. Hodgson, C. Stubbs, L. G. Forni, R. Venn, J. Gonzalez Londoño, C. Lorencio Cardenas, A. Sánchez Ginés, C. Murcia Gubianas, E. Clapes Sánchez, J. M. Sirvent, V. Panafidina, I. Shlyk, V. Ilyina, S. Judickas, G. Kezyte, I. Urbanaviciute, M. Serpytis, E. Gaizauskas, J. Sipylaite, C. L. Sprung, G. Munteanu, R. C. Morales, H. Kasdan, T. Volker, A. Reiter, Y. Cohen, Y. Himmel, J. Meissonnier, M. E. Banderas-Bravo, C. Gómez-Jiménez, M. V. García-Martínez, J. F. Martínez-Carmona, J. F. Fernández-Ortega, M. J. O‘Dwyer, M. Starczewska, M. Wilks, J. L. Vincent, The Rapid Diagnosis of Infections in the Critically Ill Team, M. Torsvik, L. T. Gustad, I. L. Bangstad, L. J. Vinje, J. K. Damås, E. Solligård, A. Mehl, M. Tsunoda, M. Kang, M. Saito, N. Saito, N. Akizuki, M. Namiki, M. Takeda, J. Yuzawa, A. Yaguchi, Tokyo Womens Medical University, F. Frantzeskaki, P. Tsirigotis, S. Chondropoulos, E. Paramythiotou, M. Theodorakopoulou, M. Stamouli, K. Gkirkas, I. K. Dimopoulou, S. Makiko, M. Tsunoda, M. Kang, J. Yuzawa, N. Akiduki, M. Namiki, M. Takeda, A. Yaguchi, S. Preau, M. Ambler, A. Sigurta, S. Saeed, M. Singer, S. Jochmans, J. Chelly, L. V. P. Vong, O. Sy, J. Serbource-Goguel, N. Rolin, C. M. Weyer, R. I. Abdallah, C. Adrie, C. Vinsonneau, M. Monchi, U. Mayr, W. Huber, E. Karsten, T. Lahmer, P. Thies, B. Henschel, G. Fischer, R. M. Schmid, O. Ediboglu, S. Ataman, I. Naz, G. Yaman, C. Kirakli, P. L. Su, P. S. Kou, W. C. Lin, C. W. Chen, J. A. Benítez Lozano, P. Carmona Sánchez, J. E. Barrueco Francioni, F. Ruiz Ferrón, J. M. Serrano Simón, Z. Riad, M. Mezidi, M. Aublanc, S. Perinel, F. Lissonde, A. Louf-Durier, H. Yonis, R. Tapponnier, J. C. Richard, B. Louis, C. Guérin, PLUG working group, M. Mezidi, H. Yonis, M. Aublanc, F. Lissonde, A. Louf-Durier, S. Perinel, R. Tapponnier, J. C. Richard, C. Guérin, K. Marmanidou, M. Oikonomou, C. Nouris, C. Loizou, E. Soilemezi, D. Matamis, P. Somhorst, D. Gommers, K. Hayashi, T. Hirayama, T. Yumoto, K. Tsukahara, A. Iida, N. Nosaka, K. Sato, T. Ugawa, A. Nakao, Y. Ujike, S. Hirohata, F. Mojoli, F. Torriglia, M. Giannantonio, A. Orlando, S. Bianzina, G. Tavazzi, S. Mongodi, M. Pozzi, G. A. Iotti, A. Braschi, PLUG Working Group, D. Jansen, S. Gadgil, J. Doorduin, L. Roesthuis, J. G. van der Hoeven, L. M. A. Heunks, G. Q. Chen, X. M. Sun, X. He, Y. L. Yang, Z. H. Shi, M. Xu, J. X. Zhou, S. M. Pereira, M. R. Tucci, B. F. F. Tonelotto, C. M. Simoes, C. C. A. Morais, M. S. Pompeo, F. U. Kay, M. B. P. Amato, J. E. Vieira, S. Suzuki, Y. Mihara, Y. Hikasa, S. Okahara, H. Morimatsu, On behalf of Okayama Research Investigation Organizing Network (ORION)investigators, H. M. Kwon, Y. J. Moon, S. H. Lee, K. W. Jung, W. J. Shin, I. G. Jun, J. G. Song, G. S. Hwang, S. Lee, Y. J. Moon, H. M. Kwon, K. Jung, W. J. Shin, I. G. Jun, J. G. Song, G. S. Hwang, A. Ramelli, T. Manca, F. Corradi, C. Brusasco, F. Nicolini, T. Gherli, R. Brianti, P. Fanzaghi, A. Vezzani, B. A. Tudor, D. A. Klaus, D. Lebherz-Eichinger, C. Lechner, C. Schwarz, M. Bodingbauer, R. Seemann, K. Kaczirek, E. Fleischmann, G. A. Roth, C. G. Krenn, A. Malyshev, S. Sergey, Y. Yamaguchi, T. Nomura, E. Yoshitake, M. Idei, T. Yoshida, S. Takaki, O. Yamaguchi, M. Kaneko, T. Goto, N. Tencé, I. Zaien, M. Wolf, P. Trouiller, F. M. Jacobs, J. M. Kelly, P. Veigas, S. Hollands, A. Min, S. Rizoli, C. M. Coronado Robles, M. A. Montes de Oca Sandoval, O. Tarabrin, D. Gavrychenko, G. Mazurenko, P. Tarabrin, I. Palacios Garcia, A. Diaz Martin, M. Casado Mendez, V. Arellano orden, R. Leal Noval, C. McCue, L. Gemmell, A. MacKay, J. Luján, P. Villa, B. Llorente, R. Molina, L. Alcázar, C. Arenillas Juanas, S. Rogero, T. Pascual, J. A. Cambronero, P. Matía Almudévar, J. Palamidessi Domínguez, S. Alcántara Carmona, D. Palacios Castañeda, A. Naharro Abellán, A. Pérez Lucendo, L. Pérez Pérez, R. Fernández Rivas, N. Martínez Sanz, J. Veganzones Ramos, P. Rodríguez Villamizar, S. Javadpour, N. Kalani, T. Amininejad, S. Jamali, S. Sobhanian, A. Laurent, M. Bonnet, R. Rigal, P. Aslanian, P. Hebert, G. Capellier, PS-ICU Group, M. R. Diaz Contreras, C. Rodriguez Mejías, F. C. Santiago Ruiz, M. Duro Lombardo, J. Castaño Perez, E. Aguayo de Hoyos, A. Estella, R. Viciana, L. Perez Fontaiña, T. Rico, V. Perez Madueño, M. Recuerda, L. Fernández, A. Sandiumenge, S. Bonet, C. Mazo, M. Rubiera, J. C. Ruiz-Rodríguez, R. M. Gracia, E. Espinel, T. Pont, A. Kotsopoulos, N. Jansen, W. F. Abdo, A. Gopcevic, Z. Gavranovic, M. Vucic, M. Zlatic Glogoski, L. Videc Penavic, A. Horvat, L. Martin-Villen, J. J. Egea-Guerero, J. Revuelto-Rey, T. Aldabo-Pallas, E. Correa-Chamorro, A. I. Gallego-Corpa, P. Ruiz del Portal-Ruiz Granados, V. Faivre, L. Wildenberg, B. Huot, A. C. Lukaszewicz, M. Simsir, C. Mengelle, D. Payen, N. Martinez Sanz, B. Lobo Valbuena, M. Valdivia de la Fuente, P. Matía Almudena, L. Pérez Pérez, S. Alcántara Carmona, A. Navarro Abellán, I. Fernández Simón, J. J. Rubio Muñoz, J. Veganzones Ramos, S. Alcantara Carmona, P. Matia Almudevar, A. Naharro Abellan, M. A. Perez Lucendo, L. Perez Perez, J. Palamidessi Dominguez, R. Fernandez Rivas, P. Rodriguez Villamizar, S. Wee, C. Ong, Y. H. Lau, Y. Wong, M. E. Banderas-Bravo, V. Olea-Jiménez, J. M. Mora-Ordóñez, C. Gómez-Jiménez, J. L. Muñoz-Muñoz, J. Vallejo-Báez, D. Daga-Ruiz, M. Lebrón-Gallardo, G. Rialp, J. M. Raurich, I. Morán, M. C. Martín, G. Heras, A. Mas, I. Vallverdú, S. Hraiech, J. Bourenne, C. Guervilly, J. M. Forel, M. Adda, P. Sylla, A. Mouaci, M. Gainnier, L. Papazian, P. R. Bauer, A. Kumbamu, M. E. Wilson, J. K. Pannu, J. S. Egginton, R. Kashyap, O. Gajic, S. Yoshihiro, M. Sakuraya, M. Hayakawa, A. Hirata, N. Kawamura, T. Tsutui, K. Yoshida, Y. Hashimoto, Japan Septic Disseminated Intravascular Coagulation (JSEPTIC DIC) study group, C. H. Chang, H. C. Hu, L. C. Chiu, C. Y. Hung, S. H. Li, K. C. Kao, S. Sibley, J. Drover, C. D’Arsigny, C. Parker, D. Howes, S. Moffatt, J. Erb, R. Ilan, D. Messenger, I. Ball, J. G. Boyd, M. Harrison, S. Ridi, J. Muscedere, A. H. Andrade, R. C. Costa, V. A. Souza, V. Gonzalez, V. Amorim, F. Rolla, C. A. C. Abreu Filho, R. Miranda, S. Atchasiri, P. Buranavanich, T. Wathanawatthu, S. Suwanpasu, C. Bureau, C. Rolland-Debord, T. Poitou, M. Clavel, S. Perbet, N. Terzi, A. Kouatchet, T. Similowski, A. Demoule, P. Diaz, J. Nunes, S. Escórcio, G. Silva, S. Chaves, M. Jardim, M. Câmara, N. Fernandes, R. Duarte, J. J. Jardim, C. A. Pereira, J. J. Nóbrega, C. M. Chen, C. C. Lai, K. C. Cheng, W. Chou, S. J. Lee, Y. S. Cha, W. Y. Lee, M. Onodera, E. Nakataki, J. Oto, H. Imanaka, M. Nishimura, A. Khadjibaev, D. Sabirov, A. Rosstalnaya, R. Akalaev, F. Parpibaev, E. Antonucci, P. Rossini, S. Gandolfi, E. Montini, S. Orlando, M. van Nes, F. Karachi, S. Hanekom, A. H. Andrade, U. V. Pereira, C. A. C. Abreu Filho, R. C. Costa, M. S. W. Parkin, M. Moore, A. H. Andrade, R. C. Costa, K. V. Silva Carvalho, C. A. C. Abreu Filho, H. J. Min, H. J. Kim, D. S. Lee, Y. Y. Choi, E. Y. Lee, I. Song, D. J. Kim, Y. Y. E, J. W. Kim, J. S. Park, Y. J. Cho, J. H. Lee, J. W. Suh, Y. H. Jo, K. S. Kim, Y. J. Lee, J. Ferrero-Calleja, D. Merino-Vega, A. I. González-Jiménez, M. Sigcha Sigcha, A. Hernández-Tejedor, A. Martin-Vivas, Á. Gabán-Díez, R. Ruiz-de Luna, N. De la Calle-Pedrosa, I. Temprano-Gómez, D. Afonso-Rivero, J. I. Pellin-Ariño, A. Algora-Weber, R. R. L. Fumis, A. B. Ferraz, J. M. Vieira Junior, H. Kirca, O. Cakin, M. Unal, H. Mutlu, A. Ramazanoglu, M. Cengiz, E. A. Nicolini, F. G. F. Pelisson, R. S. Nunes, S. L. da Silva, M. M. Carreira, F. Bellissimo-Rodrigues, M. A. Ferez, A. Basile-Filho, H. C. Chao, C. M. Chen, L. Chen, M. Hravnak, G. Clermont, M. Pinsky, A. Dubrawski, J. Luján Varas, R. Molina Montero, L. Alcázar Sánchez-Elvira, P. Villa Díaz, C. Pintado Delgado, B. Llorente Ruiz, A. Pardo Guerrero, J. A. Cambronero Galache, H. Sherif, H. Hassanin, R. El Hossainy, W. Samy, H. Ly, H. David, P. Burtin, C. Charpentier, M. Barral, P. Courant, E. Fournel, L. Gaide-Chevronnay, M. Durand, P. Albaladejo, J. F. Payen, O. Chavanon, A. Blandino Ortiz, S. Pozzebon, O. Lheureux, A. Brasseur, J. L. Vincent, J. Creteur, F. S. Taccone, F. Fumagalli, S. Scala, R. Affatato, M. De Maglie, D. Zani, D. Novelli, C. Marra, A. Luciani, D. De Zani, M. Luini, T. Letizia, D. Pravettoni, L. Staszewsky, S. Masson, A. Belloli, M. Di Giancamillo, E. Scanziani, R. Latini, G. Ristagno, Y. C. Kye, G. J. Suh, W. Y. Kwon, K. S. Kim, K. M. Yu, G. Babini, G. Ristagno, L. Grassi, F. Fumagalli, S. Bendel, M. De Maglie, R. Affatato, S. Masson, R. Latini, E. Scanziani, M. Reinikainen, M. Skrifvars, F. Kappler, M. Blobner, S. J. Schaller, A. Roasio, E. Costanzo, S. Cardellino, E. Iesu, F. Zama Cavicchi, V. Fontana, L. Nobile, J. L. Vincent, J. Creteur, F. S. Taccone, M. Park, K. M. You, G. J. Suh, W. Y. Kwon, S. B. Ko, K. S. Kim, A. Xini, L. Marca, O. Lheureux, A. Brasseur, J. L. Vincent, J. Creteur, F. S. Taccone, A. Beane, M. C. K. T. Thilakasiri, A. P. De Silva, T. Stephens, C. S. Sigera, P. Athapattu, S. Jayasinghe, A. Padeniya, R. Haniffa, A. Iglesias Santiago, V. Chica Sáez, R. de la Chica Ruiz-Ruano, A. Sánchez González, N. Kunze-Szikszay, S. Wand, P. Klapsing, A. Wetz, T. Heyne, K. Schwerdtfeger, M. Troeltzsch, M. Bauer, M. Quintel, O. Moerer, D. J. Cook, W. B. Rutherford, D. C. Scales, N. K. Adhikari, B. H. Cuthbertson, T. Suzuki, T. Takei, K. Fushimi, M. Iwamoto, S. Nakagawa, N. Mendsaikhan, T. Begzjav, G. Lundeg, M. W. Dünser, D. González Romero, J. L. Santana Cabrera, J. D. Martín Santana, Y. Santana Padilla, H. Rodríguez Pérez, R. Lorenzo Torrent, R. Kleinpell, I. Chouris, V. Radu, M. Stougianni, A. Lavrentieva, D. Lagonidis, R. D. T. Price, A. Day, N. Arora, M. A. Henderson, S. Hickey, M. I. Almeida Costa, J. P. Carvalho, A. A. Gomes, P. J. Mergulhão, K. K. C. Chan, H. P. Shum, W. W. Yan, B. Maghsoudi, S. H. Tabei, M. Masjedi, G. Sabetian, H. R. Tabatabaei, A. Akbarzadeh, Student Research Committee - Shiraz University of Medical Sciences, S. Saigal, A. Pakhare, R. Joshi, S. K. Pattnaik, B. Ray, A. F. Rousseau, L. Michel, M. Bawin, E. Cavalier, J. Y. Reginster, P. Damas, O. Bruyere, J. C. Zhou, H. Cauwenberghs, A. De Backer, H. Neels, I. Deblier, J. Berghmans, D. Himpe, J. A. Barea-Mendoza, I. Prieto Portillo, M. Valiente Fernández, R. Garcia Gigorro, J. L. Perez Vela, H. Marín Mateos, S. Chacón Alves, G. Morales Varas, A. Rodriguez-Biendicho, E. Renes Carreño, J. C. Montejo González, J. S. Yang, C. H. Chiang, W. T. Hung, W. C. Huang, C. C. Cheng, K. C. Lin, S. C. Lin, K. R. Chiou, S. R. Wann, K. L. Lin, P. L. Kang, G. Y. Mar, C. P. Liu, J. C. Zhou, Y. J. Choi, S. Z. Yoon, A. Gordillo-Brenes, M. D. Fernandez-Zamora, L. Perez-Borrero, M. D. Arias-Verdu, E. Aguilar-Alonso, A. Herruzo-Aviles, M. Garcia-Delgado, R. Hinojosa-Perez, E. Curiel-Balsera, R. Rivera-Fernandez, ARIAM-ANDALUCIA, S. P. Gómez Lesmes, L. E. De la Cruz Rosario, A. Ansotegui Hernández, A. N. García Herrera, E. Regidor Sanz, M. J. Gómez Sánchez, J. Barado Hualde, O. Agudo Pascual, J. P. Tirapu León, J. M. Guergue Irazabal, A. González Pérez, P. Alvarez Fernández, L. Lopéz Amor, G. Muñiz Albaiceta, S. P. Gómez Lesmes, L. E. De la Cruz Rosario, A. Ansotegui Hernández, E. Regidor Sanz, M. J. Gómez Sánchez, S. Aldunate Calvo, A. N. García Herrera, J. Barado Hualde, O. Agudo Pascual, J. P. Tirapu León, A. Corona, C. Ruffini, A. Spazzadeschi, F. Marrazzo, A. Gandola, R. Sciurti, C. Savi, E. Catena, M. W. Ke, C. C. Cheng, W. C. Huang, C. H. Chiang, W. T. Hung, K. C. Lin, S. C. Lin, S. R. Wann, K. R. Chiou, C. J. Tseng, P. L. Kang, G. Y. Mar, C. P. Liu, P. Bertini, F. De Sanctis, F. Guarracino, P. Bertini, R. Baldassarri, F. Guarracino, S. H. Buitinck, P. H. J. van der Voort, J. Oto, E. Nakataki, Y. Tsunano, M. Izawa, N. Tane, M. Onodera, M. Nishimura, S. Ghosh, A. Gupta, A. De Gasperi, E. Mazza, R. Limuti, M. Prosperi, N. Bissenova, A. Yergaliyeva, L. Talan, G. Yılmaz, G. Güven, F. Yoruk, N. D. Altıntas, D. N. Mukherjee, L. K. Agarwal, K. Mandal, M. Palomar, B. Balsera, M. Vallverdu, M. Martinez, M. Garcia, D. Castellana, R. Lopez, F. Barcenilla, G. E. Kaminsky, R. Carreño, A. Escribá, M. Fuentes, V. Gálvez, R. Del Olmo, B. Nieto, C. Vaquerizo, J. Alvarez, M. A. De la Torre, E. Torres, E. Bogossian, S. Aranha Nouer, D. Ribeiro Salgado, S. Carvalho Brugger, G. Jiménez Jiménez, M. Miralbés Torner, M. Vallverdú Vidal, B. Balsera Garrido, X. Nuvials Casals, F. Barcenilla Gaite, J. Trujillano Cabello, M. Palomar Martínez, M. Doganci, S. Izdes, S. Guzeldag Besevli, A. Alkan, B. Kayaaslan, C. Sánchez Ramírez, L. Caipe Balcázar, M. Cabrera Santana, M. A. Hernández Viera, S. Hípola Escalada, C. F. Lübbe Vázquez, S. M. Marrero Penichet, F. Artiles Campelo, M. A. De La Cal López, P. Saavedra Santana, S. Ruíz Santana, X. Repessé, M. Artiguenave, S. Paktoris-Papine, F. Espinasse, A. Dinh, F. El Sayed, C. Charron, G. Géri, A. Vieillard-Baron, K. Marmanidou, M. Oikonomou, C. Nouris, K. Dimitroulakis, E. Soilemezi, D. Matamis, A. Ferré, M. Guillot, J. L. Teboul, D. Lichtenstein, G. Mézière, C. Richard, X. Monnet, T. Pham, G. Beduneau, F. Schortgen, L. Piquilloud, E. Zogheib, M. Jonas, F. Grelon, I. Runge, N. Terzi, S. Grangé, G. Barberet, P. G. Guitard, J. P. Frat, A. Constan, J. M. Chrétien, J. Mancebo, A. Mercat, J. C. M. Richard, L. Brochard, The WIND study group, S. Prīdāne, O. Sabeļņikovs, F. Mojoli, A. Orlando, I. Bianchi, F. Torriglia, S. Bianzina, M. Pozzi, G. A. Iotti, A. Braschi, PLUG Working Group, G. Beduneau, T. Pham, F. Schortgen, L. Piquilloud, E. Zogheib, M. Jonas, F. Grelon, I. Runge, N. Terzi, S. Grangé, G. Barberet, P. G. Guitard, J. P. Frat, A. Constan, J. M. Chrétien, J. Mancebo, A. Mercat, J. C. M. Richard, L. Brochard, The WIND study group, E. Kondili, C. Psarologakis, S. Kokkini, V. Amargianitakis, D. Babalis, A. Chytas, I. Chouvarda, K. Vaporidi, D. Georgopoulos, O. Trapp, A. Kalenka, F. Mojoli, A. Orlando, I. Bianchi, F. Torriglia, S. Bianzina, M. Pozzi, G. A. Iotti, A. Braschi, PLUG Working Group, J. A. Benítez Lozano, P. Carmona Sánchez, J. E. Barrueco Francioni, F. Ruiz Ferrón, J. M. Serrano Simón, S. Spadaro, D. S. Karbing, A. Gioia, F. Moro, F. Dalla Corte, T. Mauri, C. A. Volta, S. E. Rees, Plug working group, M. V. Petrova, R. Mohan, A. V. Butrov, S. D. Beeharry, M. V. Vatsik, F. I. Sakieva, F. Gobert, H. Yonis, R. Tapponnier, R. Fernandez, M. A. Labaune, J. F. Burle, J. Barbier, B. Vincent, M. Cleyet, J. C. Richard, C. Guérin, C. Righy Shinotsuka, J. Creteur, F. S. Taccone, S. Törnblom, S. Nisula, S. Vaara, M. Poukkanen, S. Andersson, V. Pettilä, E. Pesonen, Z. Xie, X. Liao, Y. Kang, J. Zhang, K. Kubota, M. Egi, S. Mizobuchi, S. Hegazy, A. El-Keraie, E. El Sayed, M. Abd El Hamid, N. J. Rodrigues, M. Pereira, I. Godinho, J. Gameiro, M. Neves, J. Gouveia, Z. Costa e Silva, J. A. Lopes, J. Mckinlay, M. Kostalas, G. Kooner, G. Dudas, A. Horton, C. Kerr, N. Karanjia, B. Creagh-Brown, L. Forni, A. Yamazaki, M. Sanz Ganuza, J. A. Martinez Molina, F. Hidalgo Martinez, M. T. Chiquito Freile, N. Garcia Fernandez, P. Medrano Travieso, A. Bandert, R. Frithiof, M. Lipcsey, D. Smekal, P. Schlaepfer, J. D. Durovray, V. Plouhinec, C. Chiappa, R. Bellomo, A. G. Schneider, S. Mitchell, J. Durrant, H. Street, E. Dunthorne, J. Shears, C. Hernandez Caballero, R. Hutchison, S. Schwarze, S. Ghabina, E. Thompson, J. R. Prowle, C. J. Kirwan, C. A. Gonzalez, J. L. Pinto, V. Orozco, J. A. Patiño, P. K. Garcia, K. M. Contreras, P. Rodriguez, J. E. Echeverri

**Affiliations:** 1 Texas Children’s Hospital, Clinical Nutriiton, Houston, USA; 2 Texas Children’s Hospital, Intensive Care, Houston, USA; 3 Baylor College of Medicine, Pediatrics, Houston, USA; 4 Kochi Medical School, Department of Anesthesiology and Intensive Care Medicine, Nankoku, Japan; 5 Vall d’ Hebron University Hospital, Critical Care Department, Barcelona, Spain; 6 Vall d’ Hebron University Hospital, Anesthesia & Reanimation Department, Barcelona, Spain; 7 Vall d’ Hebron University Hospital, Neurocritical Care Department, Barcelona, Spain; 8 Vall d’ Hebron University Hospital, Coronary Care Unit, Barcelona, Spain; 9 Sagrat Cor University Hospital, Critical Care Department, Barcelona, Spain; 10 St Elisabeth Twee Steden Hospital, ICU, Tilburg, Netherlands; 11 National Institute of Cardiovascular Diseases, PCICU, Bratislava, Slovakia; 12 National Institute of Cardiovascular Diseases, Bratislava, Slovakia; 13 National Institute of Cardiovascular Diseases, Pediatric Cardiac Intensive Care Unit, Bratislava, Slovakia; 14 Texas Children’s Hospital, Intensive Care, Houston, USA; 15 Baylor College of Medicine, Pediatrics, Houston, USA; 16 Texas Children’s Hospital, Renal Service, Houston, USA; 17 Bristol Royal Hospital for Children, Paediatric Intensive Care Unit, Bristol, UK; 18 Intensive Care National Audit & Research Centre (ICNARC), London, UK; 19 University of Leeds, Leeds, UK; 20 Southampton University Hospitals NHS Trust, Southampton, UK; 21 Great Ormond Street Hospital, London, UK; 22 UCCC, ICU, Kyiv, Ukraine; 23 Catholic University, Department of Anesthesia and Intensive Care, Rome, Italy; 24 International Renal Research Institute of Vicenza (IRRIV), Department of Nephrology, Dialysis and Transplantation, Vicenza, Italy; 25 University of Florence, Department of Health Science, Section of Anaesthesiology and Intensive Care, Florence, Italy; 26 The Children’s Hospital at Montefiore, Pediatric Critical Care, Bronx, USA; 27 The Children’s Hospital at Montefiore, Bronx, USA; 28 Shiraz University of Medical Sciences, Pediatric intensive Care, Shiraz, Islamic Republic of Iran; 29 Shiraz University of Medical Sciences, Shiraz, Islamic Republic of Iran; 30 Yokohama City University Hospital, Intensive Care Unit, Yokohama, Japan; 31 Yokohama City University Hospital, Department of Anesthesiology, Yokohama, Japan; 32 The Children’s Hospital at Montefiore, Pediatric Critical Care, Bronx, USA; 33 Cairo University/Kasr Alainy Medical School, Anesthesia, Pain and Surgical ICU, Cairo, Egypt; 34 Cairo University/Kasr Alainy Medical School, Cairo, Egypt; 35 The University of Melbourne, Department of Physiotherapy, Melbourne, Australia; 36 Royal Melbourne Hospital, Department of Physiotherapy, Melbourne, Australia; 37 Peter MacCallum Cancer Centre, Department of Physiotherapy, Melbourne, Australia; 38 Flinders Medical Centre, Department of Physiotherapy, Adelaide, Australia; 39 University of South Australia, Member of the International Centre for Allied Health Evidence (iCAHE) and the Sansom Institute, Adelaide, Australia; 40 National University Hospital, Department of Rehabilitation, Singapore, Singapore; 41 Escola Superior da Saude, Health Sciences Program, Brasilia, Brazil; 42 Hospital de Base do Distrito Federal, Brasilia, Brazil; 43 University College London Hospitals, Division of Critical Care, London, UK; 44 University College London Hospitals, Institute of Sports and Exercise Health, London, UK; 45 National University Hospital, Division of Respiratory and Critical Care, Singapore, Singapore; 46 Brigham and Women’s Hospital, Department of Rehabilitation, Boston, USA; 47 Brigham and Women’s Hospital, Department of Medicine, Boston, USA; 48 Brigham and Women’s Hospital, Renal Division, Boston, USA; 49 Brigham and Women’s Hospital, Channing Division of Network Medicine, Boston, USA; 50 Queen Elizabeth Hospital NHS FT, Birmingham, UK; 51 City University London, School of Health Sciences, London, UK; 52 Griffith University, NHMRC Centre of Research Excellence in Nursing, Menzies Health Institute Queensland, Brisbane, Australia; 53 Princess Alexandra Hospital, Intensive Care Unit, Brisbane, Australia; 54 University of Dundee, Dundee, UK; 55 University of Queensland, Brisbane, Australia; 56 NHS Tayside, Dundee, UK; 57 Hospital Israelita Albert Einstein, São Paulo, Brazil; 58 Odense University Hospital, Anesthesiology and Intensive Care Medicine, Odense, Denmark; 59 Odense University Hospital, University of Southern Denmark, Odense, Denmark; 60 Royal Brompton and Harefield NHS Foundation Trust, Rehabilitation and Therapies, London, UK; 61 Royal Surrey County Hospital, ICU and SPACeR research group, Guildford, UK; 62 Royal Surrey County Hospital, Guildford, UK; 63 Guys and St Thomas NHS Foundation Trust, London, UK; 64 Khoo Teck Puat Hospital, Rehabilitation Services, Singapore, Singapore; 65 Khoo Teck Puat Hospital, Anaesthesia & Surgical Intensive Care, Singapore, Singapore; 66 Clinica Sagrada Esperança, Luanda, Angola; 67 Escola Nacional de Saude Publica/UNL, Lisboa, Portugal; 68 Fortis Escorts Heart Institute, Critical Care Medicine, New Delhi, India; 69 University of Athens, Medical School, Evangelismos Hospital, Athens, Greece; 70 National Technical University of Athens, Athens, Greece; 71 Evangelismos Hospital, Athens, Greece; 72 Hopital Bichat Claude Bernard, Paris, France; 73 Hospital Universitari Mútua de Terrassa, Intensive Care Department, Terrassa, Spain; 74 AGAUR, Grup Recerca Emergent, Terrassa, Spain; 75 Hospital General de Catalunya, Intensive Care Department, Sant Cugat del Vallés, Spain; 76 Universidad Autónoma de Barcelona, Departament de Medicina, Barcelona, Spain; 77 Hospital Universitari de Tarragona Joan XXIII, Intensive Care Department, Tarragona, Spain; 78 Hospital del Mar, Intensive Care Department, Barcelona, Spain; 79 Hospital Parc Taulí, Intensive Care Department, Sabadell, Spain; 80 Hospital General de Vic, Intensive Care Department, Vic, Spain; 81 Hospital Infanta Leonor, Intensive Care Department, Madrid, Spain; 82 Hospital del SAS de Jerez, Intensive Care Department, Jerez de la Frontera, Spain; 83 Hospital de Bellvitge, Intensive Care Department, Barcelona, Spain; 84 St James’s University Hospital, Dublin, Ireland; 85 Peking Union Medical College Hospital, Critical Care Medicine, Beijing, China; 86 Nanjing Zhongda Hospital, Southeast University School of Medicine, Critical Care Medicine, Nanjing, China; 87 Peking University First Hospital, Peking University, Research Center for Medical Mycology, Beijing, China; 88 Lille University Hospital, ICU, Lille, France; 89 Lille University Hospital, Mycology and Parasitology Lab, Lille, France; 90 Attikon University Hospital, Athens, Greece; 91 Vall d’Hebron University Hospital, Critical Care, Barcelona, Spain; 92 Universitat Autònoma de Barcelona, Barcelona, Spain; 93 CIBERES, Madrid, Spain; 94 Hannover Medical School, Department of Respiratory Medicine, Hannover, Germany; 95 German Centre of Lung Research (DZL/BREATH), Hannover, Germany; 96 Hopital Bichat Claude Bernard, Paris, France; 97 AP-HP, GH St-Louis-Lariboisière, Department of Anesthesiology and Critical Care and SMUR and Burn Unit, Paris, France; 98 AP-HP, GH St-Louis-Lariboisière, Mycology Unit, Paris, France; 99 AP-HP, GH St-Louis-Lariboisière, Pharmacy Unit, Paris, France; 100 AP-HP, GH St-Louis-Lariboisière, Plastic Surgery and Burn Unit, Paris, France; 101 AP-HP, GH St-Louis-Lariboisière, Department of Infectious Diseases, Paris, France; 102 Dudley Group of Hospitals NHS Foundation Trust, Anaesthetics, Dudley, UK; 103 Dudley Group of Hospitals NHS Foundation Trust, Microbiology, Dudley, UK; 104 Hospital Simón Bolívar, Burn ICU, Bogotá, Colombia; 105 Adult Critical Care Unit, Royal London Hospital, Barts Health NHS Trust, London, UK; 106 Queen Mary University of London, London, UK; 107 Royal London Hospital, Barts Health NHS Trust, Microbiology Department, London, UK; 108 Japanese Red Cross Musashino Hospital, Emergency And Critical Care Medicine, Tokyo, Japan; 109 Kameda Medical Center, Intensive Care Medicine, Chiba, Japan; 110 Saitama Medical Center, Jichi Medical University, Department of Anesthesiology and Critical Care Medicine, Saitama, Japan; 111 Japanese Society of Education for Physicians and Trainees in Intensive Care, Tokyo, Japan; 112 Hospital Clinic Universitari Valencia, Valencia, Spain; 113 Paulo Niemeyer State Brain Institute, Rio de Janeiro, Brazil; 114 Complejo Hospitalario de Navarra, Intensive Care Medicine, Pamplona, Navarra Spain; 115 Complejo Hospitalario de Navarra, Neurosurgery, Pamplona, Navarra Spain; 116 Complejo Hospitalario de Navarra, Neurology, Pamplona, Navarra Spain; 117 Regional Hospital, Neurocenter, Neurointensive Care Unit, Liberec, Czech Republic; 118 Military University Hospital and First Medical School, Charles University, Department of Neurosurgery, Prague, Czech Republic; 119 Regional Hospital, Neurocenter, Department of Neurosurgery, Liberec, Czech Republic; 120 IRCCS Istituto delle Scienze Neurologiche, Anesthesia and Intensive Care, Bellaria Hospital, Bologna, Italy; 121 University of Bologna, Bologna, Italy; 122 Hospital Serrania, Ronda, Spain; 123 Hospital Regional, Intensive Care, Malaga, Spain; 124 Hospital Infanta Margarita, Intensive Care, Cabra, Spain; 125 Hospital Neurotraumatologico, Jaen, Spain; 126 Hospital Virgen de las Nieves, Intensive Care, Granada, Spain; 127 Complejo Hospitalario de Navarra, Intensive Care Medicine, Pamplona, Spain; 128 Complejo Hospitalario de Navarra, Neurology, Pamplona, Spain; 129 Complejo Hospitalario de Navarra, Neurosurgery, Pamplona, Spain; 130 Complejo Hospitalario de Granada, Granada, Spain; 131 Fundación Jiménez Díaz, Madrid, Spain; 132 Hospital Rey Juan Carlos, Móstoles, Spain; 133 Dalin Tzu Chi Hospital, Buddhist Tzu Chi Medical Foundation, Chiayi, Taiwan, Province of China; 134 Centro Hospitalar Tâmega e Sousa, Intensive Care, Penafiel, Portugal; 135 Complejo Hospitalario de Navarra, Intensive Care Medicine, Pamplona, Navarra Spain; 136 Complejo Hospitalario de Navarra, Neurosurgery, Pamplona, Navarra Spain; 137 Complejo Hospitalario de Navarra, Neurology, Pamplona, Navarra Spain; 138 Burdenko Neurosurgery Institute, Moscow, Russian Federation; 139 Fiocruz, Rio de Janeiro, Brazil; 140 Paulo Niemeyer State Brain Institute, Rio de Janeiro, Brazil; 141 D’Or Institute for Research and Education, Rio de Janeiro, Brazil; 142 Hospital Gregorio Marañon, Intensive Care Unit, Madrid, Spain; 143 Hospital Gregorio Marañon, Microbiology, Madrid, Spain; 144 Hospital Juarez de México, ICU, Mexico City, Mexico; 145 Max Superspeciality Hospital, Critical Care, Pulmonary and Sleep Medicine, Mohali, India; 146 Dr. H.S.J Institute of Dental Sciences, Pharmacology, Chandigarh, India; 147 Instituto Nacional del Tórax, Fellow Respiratory Medicine, Santiago, Chile; 148 Hospital Militar de Santiago, Intensive Care, Santiago, Chile; 149 Bichat University Hospital, Medical ICU, Paris, France; 150 Bichat University Hospital, Anaesthesiology, Paris, France; 151 Bichat University Hospital, Cardiovascular surgery, Paris, France; 152 Dr. Suat Seren Chest Diseases and Surgery Training Hospital, Intensive Care Unit, Izmir, Turkey; 153 Xanthi General Hospital, ICU, Xanthi, Greece; 154 N.N. Blokhin Russian Cancer Research Center, Medical ICU, Moscow, Russian Federation; 155 N.N. Blokhin Russian Cancer Research Center, Express Laboratory, Moscow, Russian Federation; 156 N.N. Blokhin Russian Cancer Research Center, Biochemical Laboratory, Moscow, Russian Federation; 157 N.N. Blokhin Russian Cancer Research Center, Microbiology Department, Moscow, Russian Federation; 158 Hospital Serrania, Ronda, Spain; 159 Hospital Infanta Margarita, Intensive Care, Cabra, Spain; 160 Hospital Regional, Intensive Care, Malaga, Spain; 161 CHL Hospitals, Dept of Critical Care Services, Indore, India; 162 Shalby Hospital, Dept of Critical Care Services, Indore, India; 163 Hospital la Fe, Intensive Care Unit, Valencia, Spain; 164 Hospital la Fe, Valencia, Spain; 165 Yonsei Wonju University College of Medicine, Department of Internal Medicine, Wonju, Republic of Korea; 166 Yonsei University College of Medicine, Department of Internal Medicine, Seoul, Republic of Korea; 167 Yonsei University College of Medicine, AIDS Research Institute, Seoul, Republic of Korea; 168 Saint Barnabas Medical Center, Department of Medicine, Livingston, USA; 169 CHU Bordeaux, Bordeaux, France; 170 Hospital Clínic, Barcelona, Spain; 171 Rovira i Virgili University, Nursing Department, Tarragona, Spain; 172 Dr Josep Trueta University Hospital, Intensive Care Unit, Girona, Spain; 173 Quiron Salud-Hospital General de Catalunya, Intensive Care Unit, Barcelona, Spain; 174 Verge de la Cinta University Hospital, Intensive Care Unit, Tortosa, Spain; 175 Hospital de Sant Joan Despí Moissès Broggi, Intensive Care Unit, Barcelona, Spain; 176 Sant Pau I Santa Tecla University Hospital, Intensive Care Unit, Tarragona, Spain; 177 Joan XXIII University Hospital, Intensive Care Unit, Tarragona, Spain; 178 University Hospital Vall d’Hebron, Medical Transplant Coordination Department, Barcelona, Spain; 179 King’s College Hospital, ACET Research Team, London, UK; 180 St. Johns Medical College Hospital, Critical Care Medicine, Bangalore, India; 181 St. Johns Medical College Hospital, Bangalore, India; 182 Radboud University Nijmegen Medical Centre, Intensive Care, Nijmegen, Netherlands; 183 Ente Ospedaliero Ospedali Galliera, Anestesia e Rianimazione, Genova, Italy; 184 Deakin University, School of Nursing and Midwifery, Melbourne, Australia; 185 Deakin University, Centre for Quality and Patient Safety, Melbourne, Australia; 186 Victoria University, Wellington, Graduate School of Nursing Midwifery and Health, Wellington, New Zealand; 187 University of Canberra, Faculty of Health, Canberra, Australia; 188 Royal Devon and Exeter Hospital, Royal Devon and Exeter Clinical School, Exeter, UK; 189 Plymouth University, Faculty of Health and Human Sciences, Plymouth, UK; 190 Monash University, School of Nursing and Midwifery, Melbourne, Australia; 191 Ghent University, Faculty of Medicine and Health Sciences, Ghent, Belgium; 192 Ghent University, Dept. of Internal Medicine, Ghent, Belgium; 193 VU University Medical Center, Intensive Care, Amsterdam, Netherlands; 194 VU University Medical Center, Epidemiology and Biostatistics, Amsterdam, Netherlands; 195 King’s College Hospital, ACET Research Team, London, UK; 196 King’s College Hospital, Audit Team, King’s Critical Care, London, UK; 197 Royal Devon and Exeter Hospital, ICU, Exeter, UK; 198 Plymouth University/Royal Devon and exeter Hospital Clinical School, Exeter, UK; 199 Monash University, School of Nursing and Midwifery, Melbourne, Australia; 200 Royal Brompton and Harefield NHS Foundation Trust, Rehabilitation and Therapies, London, UK; 201 Senior ICU Nurse Quality and Safety VU University Medical Center, Amsterdam, Netherlands; 202 ICU Nursing, VU University Medical Center, Amsterdam, Netherlands; 203 VU University Medical Center, Amsterdam, Netherlands; 204 St. Johns Medical College Hospital, Critical Care Medicine, Bangalore, India; 205 St. Johns Medical College Hospital, Bangalore, India; 206 St. Johns Medical College, Bangalore, India; 207 Fondazione Cardiocentro Ticino, Cardiac Anesthesia and Intensive Care, Lugano, Switzerland; 208 IRCCS San Raffaele Scientific Institute, Department of Anesthesia and Intensive Care, Milan, Italy; 209 3rd Hospital of HeBei Medical University, Department of Intensive Care Medicine, Shi Jiazhuang, China; 210 University of Liverpool, Institute of Ageing and Chronic Disease, Liverpool, UK; 211 University of Liverpool, Institute of Infection and Global Health, Liverpool, UK; 212 Royal Liverpool University Hospital, Liverpool, UK; 213 Samsung Medical Center, Surgery, Seoul, Republic of Korea; 214 Samsung Medical Center, Critical Care Medicine, Seoul, Republic of Korea; 215 Royal Surrey County Hospital NHS Foundation Trust, Department of Intensive Care Medicine and Surrey Peri-Operative Anaesthesia and Critical Care Collaborative Research Group (SPACER), Guildford, UK; 216 Kaohsiung Veterans General Hospital, Critical Care Division, Kaohsiung City, Taiwan, Province of China; 217 Kaohsiung Veterans General Hospital, Cardiovascular Division, Kaohsiung City, Taiwan, Province of China; 218 Kaohsiung Veterans General Hospital, Department of Medical Education and Research, Kaohsiung City, Taiwan, Province of China; 219 Universidad de Concepción, Facultad de Medicina, Departamento de Kinesiología, Concepcion, Chile; 220 Hospital Dr. Sótero del Río, Unidad de Pacientes Críticos, Santiago, Chile; 221 Hospital Eugenio Espejo, Unidad de Cuidados Intensivos, Quito, Ecuador; 222 Universidad de la República, Hospital de Clínicas, Centro de Terapia Intensiva, Montevideo, Uruguay; 223 Pontificia Universidad Catolica de Chile, Facultad de Medicina, Departamento de Medicina Intensiva, Santiago, Chile; 224 Hospital Guillermo Grant Benavente, Concepcion, Chile; 225 Hokkaido University Graduate School of Medicine, Emergency and Critical Care Medicine, Sapporo, Japan; 226 Hospital 12 de Octubre, Medicina Intensiva, Madrid, Spain; 227 VU University Medical Center, Intensive Care Medicine, Amsterdam, Netherlands; 228 VU University Medical Center Amsterdam, Anaesthesiology, Amsterdam, Netherlands; 229 University of Alexandria, Critical Care, Alexandria, Egypt; 230 Medical Research Institute, Biomedical Informatics & Medical Statistics, Alexandria, Egypt; 231 Shiraz University of Medical Sciences, Anesthesia and Intensive Care Department, Shiraz, Islamic Republic of Iran; 232 Anesthesiology and Critical Care Research Center, Shiraz University of Medical Sciences, Shiraz, Islamic Republic of Iran; 233 Shiraz University of Medical Sciences, Shiraz, Islamic Republic of Iran; 234 Regional Hospital Liberec, Department of Anesthesia and intensive Care, Liberec, Czech Republic; 235 Chungnam National University Hospital, Anesthesiology and Pain Medicine, Daejon, Republic of Korea; 236 Hyogo College of Medicine, General Internal Medicine, Nishinomiya, Japan; 237 Hyogo College of Medicine, Clinical Epidemiology, Nishinomiya, Japan; 238 Brigham and Women’s Hospital, General Internal Medicine, Boston, USA; 239 National Cheng Kung University Hospital, Department of Internal Medicine, Tainan, Taiwan, Province of China; 240 National Cheng Kung University, Medical Device Innovation Center, Tainan, Taiwan, Province of China; 241 University of Padova, Department of Medicine (DIMED), Padova, Italy; 242 Azienda Ospedaliera di Padova, Emergency Department, Padova, Italy; 243 University of Toronto, Interdepartmental Division of Critical Care Medicine, Toronto, Canada; 244 Università degli Studi di Milano, Dipartimento di Fisiopatologia Medico Chirurgica e dei Trapianti, Milano, Italy; 245 Policlinico Di Milano, Dipartimento di Anestesia, Rianimazione, Urgenza ed Emergenza, Milano, Italy; 246 Plug Working Group, Milan, Italy; 247 Università degli Studi di Milano, Dipartimento di Fisiopatologia Medico-Chirurgica e dei Trapianti, Milano, Italy; 248 Georg-August-University Goettingen, Anesthesiology and Intensive Care Medicine, Goettingen, Germany; 249 Univesity of Zagreb, Department of Intensive Care Medicine, Rebro, Croatia; 250 Hospital Israelita Albert Einstein, Critical Care Medicine, São Paulo, Brazil; 251 Academic Medical Center, University of Amsterdam, Intensive Care, Amsterdam, Netherlands; 252 Australian and New Zealand Intensive Care Research Centre, School of Public Health, Monash University, Department of Epidemiology and Preventive Medicine, Melbourne, Australia; 253 Institute of Cardiometabolism and Nutrition (iCAN); Hôpital de la Pitié-Salpêtrière, Assistance Publique-Hôpitaux de Paris, Medical-Surgical Intensive Care Unit, Paris, France; 254 Groupe Hospitalier Henri Mondor; Assistance Publique-Hôpitaux de Paris, Service de Réanimation Médicale, Créteil, France; 255 Inserm, Sorbonne Paris Cité, ECSTRA Team; Université Paris Diderot, UMR 1153, Paris, France; 256 Inserm; Université Paris Est Créteil, UMR 915, Créteil, France; 257 Université Pierre et Marie Curie-Paris VI, Service de Réanimation Médicale, Paris, France; 258 University Hospital Carl Gustav Carus, Technische Universität Dresden, 21Pulmonary Engineering Group, Department of Anesthesiology and Intensive Care Medicine, Dresden, Germany; 259 IRCCS San Martino IST, University of Genoa, Surgical Sciences and Integrated Diagnostics, Genoa, Italy; 260 Hospital for Sick Children, Physiology and Experimental Medicine, Toronto, Canada; 261 Hospital for Sick Children, University of Toronto, Critical Care Medicine and Anesthesiology, Toronto, Canada; 262 University of Toronto, Interdepartmental Division of Critical Care Medicine, Toronto, Canada; 263 Hospital for Sick Children, University of Toronto, Neonatal Medicine, Toronto, Canada; 264 Hospital for Sick Children, Division of Electron Microscopy, Toronto, Canada; 265 Keenan Research Centre, Li Ka Shing Knowledge Institute, St. Michael’s Hospital, Toronto, Canada; 266 University of Toronto, Surgery and Physiology, Toronto, Canada; 267 Hopital Europeen Georges Pompidou, Anesthesiology and Intensive Care Medicine, Paris, France; 268 University of California Berkeley, Berkeley, USA; 269 Université Paris Diderot, Paris, France; 270 University of Washington, Seattle, USA; 271 Université de Versailles Saint-Quentin-en-Yvelines, Garches, France; 272 Faculty of Pharmacy-Ain Shams University, Cairo, Egypt; 273 Critical Care Medicine Department Cairo University Hospitals, Cairo, Egypt; 274 Faculty of Medicine - Ain Shams University, Cairo, Egypt; 275 Sacro Cuore Catholic University, A. Gemelli Hospital, Department of Anesthesiology and Intensive Care, Rome, Italy; 276 Bloomsbury Institute of Intensive Care Medicine, University College London, London, UK; 277 Dipartimento di Fisiopatologia Medico-Chirurgica e dei Trapianti, Milan, Italy; 278 Danube University, Department for Health Sciences and Biomedicine, Krems, Austria; 279 Hospital Universtario Puerta de Hierro Majadahonda, Intensive Care Unit, Madrid, Spain; 280 AP-HP, Saint Louis and Lariboisière University Hospitals, Department of Anesthesiology and Critical Care Medicine, Paris, France; 281 AP-HP, Hôpital Cochin, Medical Intensive Care Unit, Paris, France; 282 AP-HP, Hôpital Saint-Antoine, Medical Intensive Care Unit, Paris, France; 283 Saint-Eloi University Hospital, Department of Anesthesiology and Critical Care Medicine, Montpellier, France; 284 AP-HM, Hôpital Nord, Department of Anesthesiology and Critical Care Medicine, Marseille, France; 285 AP-HP, Saint Louis University Hospital, Biostatistics and Clinical Epidemiology Research Unit Department, Paris, France; 286 AP-HP, Hôpital Ambroise Paré, Department of Anesthesiology and Critical Care Medicine, Paris, France; 287 1st Medical Faculty, Charles University, Prague, Czech Republic; 288 3rd Medical Faculty, Charles University, Prague, Czech Republic; 289 University of Pavia, Pavia, Italy; 290 Erasme University Hospital, Université Libre de Bruxelles, Intensive Care Unit, Bruxelles, Belgium; 291 Experimental Laboratory of Intensive Care, Université Libre de Bruxelles, Bruxelles, Belgium; 292 Edwards Lifesciences, Critical Care, Irvine, USA; 293 University of California Irvine, Biomedical Engineering, Irvine, USA; 294 University of California Los Angeles, Anesthesiology, Los Angeles, USA; 295 University Medical Centre Ljubljana, Department of Anaesthesiology and Surgical Intensive Therapy, Ljubljana, Slovenia; 296 University Medical Center Ljubljana, Department of Pulmonary Diseases and Allergy, Ljubljana, Slovenia; 297 University Medical Center Ljubljana, Center for Intensive Internal Medicin, Ljubljana, Slovenia; 298 Seinäjoki Central Hospital, Department of Anaesthesiology and Intensive Care Medicine, Seinäjoki, Finland; 299 Tampere University Hospital, Department of Intensive Care Medicine, Tampere, Finland; 300 Tampere University Hospital, Department of Emergency Medicine, Tampere, Finland; 301 Helsinki University Hospital, Department of Anaesthesiology, Intensive Care, Emergency Care and Pain Medicine, Helsinki, Finland; 302 Kaohsiung Veterans General Hospital, Department of Critical Care Medicine, Kaohsiung City, Taiwan, Province of China; 303 Kaohsiung Veterans General Hospital, Cardiovascular division, Kaohsiung City, Taiwan, Province of China; 304 Kaohsiung Veterans General Hospital, Department of Emergency, Kaohsiung City, Taiwan, Province of China; 305 Kaohsiung Veterans General Hospital, Cardiovascular Division, Kaohsiung City, Taiwan, Province of China; 306 Kaohsiung Veterans General Hospital, Department of Internal Medicine, Kaohsiung City, Taiwan, Province of China; 307 ICU Cochin Hospital, Paris, France; 308 Emergency Department, Cochin Hospital, Paris, France; 309 ICU Mignot Hospital, Versailles, France; 310 Anesthesiology and Intensive Care, Catholic University School of Medicine, Rome, Italy; 311 University of Washington School of Medicine, Seattle Children’s Hospital, Pediatric Critical Care Medicine, Seattle, USA; 312 University College London, Faculty of Medical Sciences, Division of Infection & Immunity, London, UK; 313 Immunexpress, Seattle, USA; 314 Radboud University Medical Center, Intensive Care Research, Nijmegen, Netherlands; 315 Marmara University, Anaesthesiology and Reanimation, Istanbul, Turkey; 316 Marmara University, Pediatric Immunology, Istanbul, Turkey; 317 Bloomsbury Institute of Intensive Care Medicine, University College London, London, UK; 318 Hospital Virgen del Rocio, Sevilla, Spain; 319 ABC Medical Center, Critical Care Unit, Mexico, Mexico; 320 ABC Medical Center, Mexico, Mexico; 321 Hôpital Tenon, APHP, Medical and Surgical ICU, Paris, France; 322 Université Paris Diderot, Sorbonne Paris Cité, UMR 1153, Paris, France; 323 University of Toronto Saint Michael’s Hospital and Keenan Research Centre, Interdepartmental Division of Critical Care, Toronto, Canada; 324 Rouen University Hospital, Medical Intensive Care, Rouen, France; 325 Rouen University Hospital, UPRES EA3830 IRIB, Rouen, France; 326 CHU Henri Mondor, Medical ICU, Créteil, France; 327 University Hospital of Lausanne, Intensive Care and Burn Unit, Lausanne, Switzerland; 328 CHU d’Amiens, Cardiothoracic and Vascular ICU, Amiens, France; 329 Université Jules Verne, Picardie, INSERM U1088 - CURS, Amiens, France; 330 CHU Hotel Dieu, Medical ICU, Nantes, France; 331 Hospital Le Mans, Intensive Care Unit, Le Mans, France; 332 CHR d’Orleans, Medical ICU, Orleans, France; 333 CHU Grenoble Alpes, Medical ICU, Grenoble, France; 334 Université Grenoble-Alpes, Inserm U1042, Grenoble, France; 335 CHR de Mulhouse, Medical ICU, Mulhouse, France; 336 Rouen University Hospital, Surgical Intensive Care, Rouen, France; 337 CHU de Poitiers, Medical ICU, Poitiers, France; 338 Université de Poitiers, INSERM, CIC- 1402, Équipe 5 ALIVE, Poitiers, France; 339 CHU Henri Mondor and ReVA Network, Medical ICU, Créteil, France; 340 CHU d’Angers, Clinical Research Institute, Angers, France; 341 Hospital de Sant Pau, Servei de Medicina Intensiva, Barcelona, Spain; 342 CHU d’Angers, Medical ICU, Angers, France; 343 Annecy Genevois General Hospital, Annecy, France; 344 ‘Papageorgiou’ General Hospital, ICU, Thessaloniki, Greece; 345 Dipartimento dell‘Emergenza e dei Trapianti d‘Organo, Università degli Studi “Aldo Moro”, Bari, Italy; 346 Ferrara University, Ferrara, Italy; 347 Hospital Universitari Joan XXIII, Critical Care Medicine, Tarragona, Spain; 348 Universitat Rovira i Virgili/IISPV, Tarragona, Spain; 349 St James’s University Hospital. Trinity Center for Health Sciences, Anaesthesia and Critical Care, Dublin, Ireland; 350 Hospital Parc Tauli, Critical Care Medicine, Sabadell, Spain; 351 Hospital del Mar/CIBERES/UPF, Critical Care Medicine, Barcelona, Spain; 352 Hospital del Henares, Critical Care Medicine, Madrid, Spain; 353 Hospital Dr. Negrín, Critical Care Medicine, Las Palmas de Gran Canaria, Spain; 354 Hospital Universitari Joan XXIII, ORL, Tarragona, Spain; 355 UT Heatlh Science Center at San Antonio and South Texas Veterans Health Care System, Pulmonary Disease and Critical Care, San Antonio, USA; 356 Hospital de Mataró, Critical Care Medicine, Mataró, Spain; 357 Hospital de Getafe, Critical Care Medicine, Madrid, Spain; 358 University of Milan - Biocca San Genaro Hospital, School of Medicine and Surgery, Monza, Italy; 359 University of Gothenburg, Department of Anesthesia and Intensive Care, Sahlgrenska University Hospital, Göteborg, Sweden; 360 University of Gothenburg, Department of Cardiothoracic Anesthesia and Intensive Care, Sahlgrenska University Hospital, Göteborg, Sweden; 361 Guy’s & St Thomas Hospital, London, UK; 362 Nordsjællands Hospital, Dept. of Anaesthesiology and Intensive Care, Hillerød, Denmark; 363 Rigshospitalet, Copenhagen University Hospital, Dept. of intensive Care, Copenhagen, Denmark; 364 Rigshospitalet, Copenhagen University Hospital, CHIP & PERSIMUNE, Dept. of Infectious Diseases, Copenhagen, Denmark; 365 University of Copenhagen, Dept. of Biostatistics, Copenhagen, Denmark; 366 St George’s, University of London, London, UK; 367 St Georges NHS Foundation Trust, General Intensive Care, London, UK; 368 St Georges NHS Foundation Trust, Clinical Blood Sciences, London, UK; 369 St George’s, University of London, Renal Medicine, London, UK; 370 St George’s, University of London, General Intensive Care, London, UK; 371 Department of Intensive Care Medicine and Surrey Peri-Operative Anaesthesia and Critical Care Collaborative Research Group (SPACER), Guildford, UK; 372 Department of Anaesthesia, Royal Surrey County Hospital NHS Foundation Trust, Guildford, UK; 373 Royal Devon and Exeter Hospital, Exeter, UK; 374 South Devon Healthcare NHS Foundation Trust, Torquay, UK; 375 Department of Anaesthesia and Intensive Care, Royal United Hospital Bath NHS Trust, Bath, UK; 376 Department of Surgery, Royal United Hospital Bath NHS Trust, Bath, UK; 377 CH Chartres, Réanimation, Chartres, France; 378 Aix-Marseille Université, Unité de Recherche EA 3279, Marseille, France; 379 Clinique Ambroise Paré, Réanimation, Neuilly/Seine, France; 380 CHU Dijon Bourgogne, Anesthésie Réanimation, Dijon, France; 381 CHI Toulon/La Seyne sur Mer, Réanimation Polyvalente, Toulon, France; 382 CH Troyes, Réanimation, Troyes, France; 383 CHU Edouard Herriot, Hospices Civils de Lyon, Réanimation Polyvalente, Lyon, France; 384 Hôpital Européen de Marseille, Réanimation, Marseille, France; 385 CHU Cochin, AP-HP, Réanimation Chirurgicale, Paris, France; 386 CH Victor Provo, Réanimation Polyvalente, Roubaix, France; 387 CH Emile Roux, Réanimation, Le Puy-en-Velay, France; 388 CHU Saint-Louis, Réanimation Médicale, Paris, France; 389 CHU La Milétrie, Réanimation Médicale, Poitiers, France; 390 Hôpital Européen Georges Pompidou, AP-HP, Réanimation Chirugicale, Paris, France; 391 CHU Brest, Réanimation Médicale, Brest, France; 392 Groupe Hospitalier Paris Saint-Joseph, Médecine Intensive et Réanimation, Paris, France; 393 CHU Strasbourg, Hôpital Civil, Réanimation Chirugicale Polyvalente, Strasbourg, France; 394 Groupe Hospitalier de La Rochelle - Ré - Aunis, Réanimation, La Rochelle, France; 395 CHU Beaujon, AP-HP, Anesthésie Réanimation, Clichy, France; 396 CH Lens, Réanimation, Lens, France; 397 CHU Strasbourg, Hôpital Hautepierre, Réanimation Chirugicale, Strasbourg, France; 398 CH Auxerre, Réanimation, Auxerre, France; 399 CHU Nice, Hôpital Pasteur, Réanimation Médico-Chirurgicale, Nice, France; 400 CHU Hôpital Nord, AP-HM, Réanimation Chirugicale, Marseille, France; 401 CHU La Milétrie, Réanimation Chirugicale, Poitiers, France; 402 St George Hospital, Intensive Care, Sydney, Australia; 403 St George Hospital, Sydney, Australia; 404 Great Ormond Street Hospital, Critical Care and Cardiorespiratory Division, London, UK; 405 Great Ormond Street Hospital, Paediatric Intensive Care Unit, London, UK; 406 St Mary’s Hospital and Charing Cross Hospital Imperial College NHS trust, Paediatric Intensive Care Unit, London, UK; 407 Great Ormond Street Hospital, Children’s Acute Transport Service, London, UK; 408 Hospital Sao Domingos, ICU, Sao Luis, Brazil; 409 Hospital Sao Domingos, Sao Luis, Brazil; 410 HIGA Gral San Martin, La Plata, Argentina; 411 Hospital de Trauma F Abete, Municipio Islas Malvinas, Argentina; 412 Sanatorio Anchorena, Buenos Aires, Argentina; 413 Hospital Municipal Chivilcoy, Chivilcoy, Argentina; 414 Instituto De Neurología Cognitiva-INECO, Buenos Aires, Argentina; 415 Instituto Fleni, Escobar, Argentina; 416 Sanatorio Güemes, Buenos Aires, Argentina; 417 Hospital Británico de Buenos Aires, Buenos Aires, Argentina; 418 Hospital Austral, Pilar, Argentina; 419 Hospital San Antonio, Gualeguay, Argentina; 420 Clinica Bazterrica, Buenos Aires, Argentina; 421 Hospital Domingo Funes, Santa Maria de Punilla, Argentina; 422 Hospital San Luis, San Luis, Argentina; 423 HIGA San José, Pergamino, Argentina; 424 Hospital Artemides Zatti, Viedma, Argentina; 425 Hospital de Gastroenterologia Udaondo, Buenos Aires, Argentina; 426 Sanatorio Parque, Rosario, Argentina; 427 Hospital Dr. Carlos Macías, Mar de Ajó, Argentina; 428 Fondazione IRCCS Cà Granda-Ospedale Maggiore Policlinico, Department of Anaesthesiology and Intensive Care, Milan, Italy; 429 Fondazione IRCCS Cà Granda-Ospedale Maggiore Policlinico, Department of Anaesthesiology and Intensive Care, Milano, Italy; 430 University of Washington School of Medicine, Department of Neurological Surgery, Seattle, USA; 431 Washington University, Mallinckrodt Institute of Radiology, St. Louis, USA; 432 Fondazione IRCCS Cà Granda-Ospedale Maggiore Policlinico, Department of Neuroradiology, Milano, Italy; 433 Milan University, Milano, Italy; 434 Washington University, Department of Neurology, St. Louis, USA; 435 Burdenko Neurosurgery Institute, Moscow, Russian Federation; 436 Medisch Spectrum Twente, Clinical Neurophysiology and Neurology, Enschede, Netherlands; 437 Rijnstate Hospital, Neurology, Arnhem, Netherlands; 438 University of Twente, Clinical Neurophysiology, Enschede, Netherlands; 439 Medisch Spectrum Twente, Intensive Care Medicine, Enschede, Netherlands; 440 Rijnstate Hospital, Intensive Care Medicine, Arnhem, Netherlands; 441 Azienda Socio Sanitaria Territoriale Papa Giovanni XXIII, Anesthesia and Critical Care Medicine, Bergamo, Italy; 442 Azienda Socio Sanitaria Territoriale Papa Giovanni XXIII, Neurology, Bergamo, Italy; 443 University of Milan, Milano, Italy; 444 University of Milan - Bicocca, Monza, Italy; 445 Ospedale San Gerardo, Neurology, Monza, Italy; 446 Ospedale San Gerardo, Anesthesia and Critical Care Medicine, Monza, Italy; 447 Fondazione IRCCS Ca’ Granda Ospedale Maggiore Policlinico, Neuroscience Intensive Care Unit, Milan, Italy; 448 Università degli Studi di Milano, Milan, Italy; 449 Hospital Insular Las Palmas GC, Las Palmas de Gran Canaria, Spain; 450 Hospital Insular Las Palmas GC, Department of Economy, University of Las Palmas de Gran Canaria, Las Palmas de Gran Canaria, Spain; 451 Academic Medical Center, University of Amsterdam, Medical Informatics, Amsterdam, Netherlands; 452 Mashhad University of Medical Sciences, Pharmaceutical Research Center, Tehran, Islamic Republic of Iran; 453 Gelre Hospitals Apeldoorn, Intensive Care Medicine, Apeldoorn, Netherlands; 454 Academic Medical Center, University of Amsterdam, Intensive Care Medicine, Amsterdam, Netherlands; 455 Queen’s University, Centre for Neuroscience Studies, Kingston, Canada; 456 Queen’s University, Medicine and Critical Care, Kingston, Canada; 457 Queen’s University, Anaesthesia and Perioperative Medicine, Kingston, Canada; 458 Queen’s University, Surgery, Kingston, Canada; 459 Queen’s University, Medicine (Neurology) and Critical Care, Kingston, Canada; 460 University College London Hospitals NHS Foundation Trust, Division of Critical Care, London, UK; 461 University College London Hospitals, NIHR Biomedical Research Centre, London, UK; 462 National University Health System, Division of Respiratory and Critical Care, Singapore, Singapore; 463 UCL/University College London Hospitals NHS Foundation Trust, Institute of Sport, Exercise and Health, London, UK; 464 Guy’s & St Thomas’ NHS Foundation Trust and King’s College London, NIHR Biomedical Research Centre, London, UK; 465 Guy’s & St Thomas’ NHS Foundation Trust, Lane Fox Clinical Respiratory Physiology Research Centre, London, UK; 466 St Mary’s Hospital and Imperial College London, Hepatology & Gastroenterology, London, UK; 467 Kings College Hospital NHS Foundation Trust, Institute of Liver Studies, London, UK; 468 Kings College Hospital NHS Foundation Trust, Department of Radiology, London, UK; 469 Kings College London, Centre of Human and Aerospace Physiological Sciences, London, UK; 470 King’s College London, Department of Asthma, Allergy and Lung Biology, London, UK; 471 Queen’s Hospital, Critical Care Department, Romford, UK; 472 Queen’s Hospital, Pharmacy Service, Romford, UK; 473 Pamela Youde Nethersole Eastern Hospital, Hong Kong, Hong Kong China; 474 Tuen Mun Hospital, Hong Kong, Hong Kong China; 475 Hospital Universitario La Paz, Madrid, Spain; 476 Queen Elizabeth University Hospital, Anaesthetics and Intensive Care, Glasgow, UK; 477 Royal Berkshire Hospital, Critical Care Unit, Reading, UK; 478 Hospital Costa del Sol Marbella, Malaga, Spain; 479 NRI Medical College and Hospital, Department of Critical Care, Guntur, India; 480 Hospital Universitario Virgen de la Victoria, Malaga, Spain; 481 Hospital Universitari Mutua Terrassa, Intensive Care Medicine, Terrassa, Spain; 482 Hospital Universitari Mutua Terrassa, Critical Care Department, Terrassa, Spain; 483 Clinica Sagrada Esperança, Luanda, Angola; 484 Lancashire Teaching Hospitals Trust, Critical Care, Preston, UK; 485 Fundación Jiménez Díaz, Madrid, Spain; 486 Hospital Rey Juan Carlos, Madrid, Spain; 487 Broomfield Hospital, Intensive Care Unit, Chelmsford, UK; 488 Hokkaido University Hospital, Emergency and Critical Care Center, Sapporo, Japan; 489 University Teaching Hospital of Purpan, Critical Care Medicine, Toulouse, France; 490 INSERM 1214, Toulouse NeuroImaging Center (TONIC), Toulouse, France; 491 University Teaching Hospital of Purpan, Toulouse, France; 492 School of Medicine and Surgery, University Milano Bicocca and Hospital San Gerardo, Department of Anaesthesiology and Critical Care, Monza, Italy; 493 University Hospital of Clermont-Ferrand, Critical Care Unit, Clermont-Ferrand, France; 494 University Hospital and University of Liège, Cyclotron Research Center and Department of Neurology, Liège, Belgium; 495 Groupe Hospitalier Pitié-Salpétrière, APHP, Critical Care and Anaesthesiology Department, Paris, France; 496 Hôpital Erasme, Université Libre de Bruxelles, Department of Intensive Care, Brussels, Belgium; 497 Ospedali Riuniti, Department of Perioperative Medicine, Trieste, Italy; 498 CHUV, Department of Neurology, Lausanne, Switzerland; 499 Hopital Erasme, Université Libre de Bruxelles, Department of Neurology, Brussels, Belgium; 500 CHUV, Department of Intensive Care, Lausanne, Switzerland; 501 Hokkaido University Hospital, Emergency and Critical Care Medicine, Sapporo, Japan; 502 Hokkaido University Hospital, Clinical Research and Medical Innovation Center, Sapporo, Japan; 503 Hokkaido University Graduate School of Medicine, Division of Acute and Critical Care Medicine, Sapporo, Japan; 504 Aarhus University Hospital, Anesthesiology, Aarhus, Denmark; 505 Beth Israel Deaconess Medical Center, Harvard Medical School, Emergency Medicine, Boston, USA; 506 Medical City Children’s Hospital, Dallas, USA; 507 The Children’s Hospital of Philadelphia, Philadelphia, USA; 508 University of Pennsylvania, Philadelphia, USA; 509 Institute of Public Health, Charité - Universitätsmedizin Berlin, Berlin, Germany; 510 Beth Israel Deaconess Medical Center, Harvard Medical School, Critical Care, Boston, USA; 511 Homolka Hospital, Cardiology, Prague, Czech Republic; 512 Chulalongkorn University, Pulmonary and Critical Care Medicine, Bangkok, Thailand; 513 King Chulalongkorn Memmorial Hospital, Bangkok, Thailand; 514 Chulalongkorn University, Medicine, Bangkok, Thailand; 515 University of Helsinki and Helsinki University Hospital, Division of Intensive Care Medicine, Department of Anaesthesiology, Intensive Care and Pain Medicine, Helsinki, Finland; 516 IRCCS - Istituto di Ricerche Farmacologiche ‘Mario Negri’, Department of Cardiovascular Research, Milan, Italy; 517 Kuopio University Hospital, Division of Intensive Care Medicine, Kuopio, Finland; 518 Oulu University Hospital, Medical Research Center Oulu, Department of Anaesthesiology, University of Oulu and Division of Intensive Care Medicine, Oulu, Finland; 519 Tampere University Hospital, Department of Intensive Care, Tampere, Finland; 520 Helsinki University Hospital, Department of Neurology, Helsinki, Finland; 521 University-Hospital of Padova, Department of Laboratory Medicine, Padova, Italy; 522 Seoul National University Hospital, Emergency Medicine, Seoul, Republic of Korea; 523 Université Libre de Bruxelles - Erasme University Hospital, Department of Intensive Care, Bruxelles, Belgium; 524 St Luke’s International Hospital, Emergency, Tokyo, Japan; 525 St Luke’s International Hospital, Tokyo, Japan; 526 Hospital Universitari Mutua Terrassa, Critical Care Department, Terrassa, Spain; 527 Hospital Universitari Mutua Terrassa, Terrassa, Spain; 528 Na Homolce Hospital, Prague, Czech Republic; 529 National Taiwan University Hospital, Department of Anesthesiology, Taipei, Taiwan, Province of China; 530 National Taiwan University Hospital, Department of Surgery, Taipei, Taiwan, Province of China; 531 Far Eastern Memorial Hospital, Department of Anesthesiology, New Taipei, Taiwan, Province of China; 532 Division of Electrophysiology, Department of Cardiovascular Medicine, University of Münster, Münster, Germany; 533 Division of Cardiology, Department of Cardiovascular Medicine, University of Münster, Münster, Germany; 534 CHU Montpellier, Montpellier, France; 535 Na Homolce Hospital, Prague, Czech Republic; 536 Charles University in Prague, Prague, Czech Republic; 537 Cantonal Hospital Winterthur, Winterthur, Switzerland; 538 University Hospital Zurich, Institute for Anaesthesiology, Cardio-surgical Intensive Care Unit, Zurich, Switzerland; 539 Erasmus Medical Center, Rotterdam, Netherlands; 540 University Hospital Erasme, Université Libre de Bruxelles, Critical Care, Brussels, Belgium; 541 National Taiwan University Hospital, Taipei, Taiwan, Province of China; 542 Chang Gung Memorial Hospital, Linkou, Taoyaun Taiwan, Province of China; 543 National Taiwan University Hospital, Department of Surgery, Taipei, Taiwan, Province of China; 544 Hallym University, Emergency Medicine, Seoul, Republic of Korea; 545 Hanyang University, Emergency Medicine, Seoul, Republic of Korea; 546 Hanyang University, Neurosurgery, Seoul, Republic of Korea; 547 Kyung Hee University, Preventive Medicine, Seoul, Republic of Korea; 548 University Medical Center Hamburg-Eppendorf, Intensive Care Medicine, Hamburg, Germany; 549 University Heart Center Hamburg-Eppendorf, General and Interventional Cardiology, Hamburg, Germany; 550 University Heart Center Hamburg-Eppendorf, Cardiovascular Surgery, Hamburg, Germany; 551 St. Franziskus Hospital Münster, Pneumology and Intensive Care Medicine, Münster, Germany; 552 Università La Sapienza di Roma, UOD Terapia Intensiva nei Trapianti d’Organo, Policlinico Umberto I, Roma, Italy; 553 University of Turin, Dept. of Surgical Sciences, Turin, Italy; 554 Città della Salute e della Scienza Hospital, Dept. of Anesthesiology, Turin, Italy; 555 INSERM, U1116, Vandoeuvre-les-Nancy, France; 556 Ecole de Chirurgie de Nancy, Vandoeuvre-les-Nancy, France; 557 Ecole de Chirurgie de Nancy, Vandoeuvre les Nancy, France; 558 CHU de Nancy, Plateforme d’Aide à la Recherche Clinique, Vandoeuvre-les-Nancy, France; 559 Hopital Europeen Georges Pompidou, Paris, France; 560 Hospital Universitario Infanta Leonor, Intensive Care Unit, Madrid, Spain; 561 Glasgow Royal Infirmary, Anaesthesia, Glasgow, UK; 562 Seoul National University Bundang Hospital, Internal Medicine, Seongnam, Republic of Korea; 563 Ghent University Hospital, General Internal Medicin, Gent, Belgium; 564 Ghent University, Public Health, Gent, Belgium; 565 Ghent University, Internal Medicin, Gent, Belgium; 566 Ghent University, General Internal Medicine, Gent, Belgium; 567 Ghent University, Family Medicine and Primary Health Care, Gent, Belgium; 568 Ghent University Hospital, Internal Medicine, Gent, Belgium; 569 University Hospital Zurich, University Zurich, Zürich, Switzerland; 570 Hospital Uster, Department of Internal Medicine, Uster, Switzerland; 571 University Hospital Zurich, University Zurich, Heart and Vascular Surgical Intensive Care Unit, Zurich, Switzerland; 572 University Hospital Zurich, University Zurich, Surgical Intensive Care Unit, Zurich, Switzerland; 573 University Hospital Zurich, University Zurich, Neurosurgical Intensive Care Unit, Zurich, Switzerland; 574 University Hospital Zurich, University Zurich, Medical Intensive Care Unit, Zurich, Switzerland; 575 University Hospital Zurich, University Zurich, Surgical Intensive Care Unit, Zürich, Switzerland; 576 Gelre Hospitals Apeldoorn, Intensive Care Medicine, Apeldoorn, Netherlands; 577 Gelre Hospitals Apeldoorn, Physiotherapy, Apeldoorn, Netherlands; 578 Catharina Hospital, Intensive Care Medicine, Eindhoven, Netherlands; 579 Academic Medical Center, University of Amsterdam, Intensive Care Medicine, Amsterdam, Netherlands; 580 General University Hospital of Castellon, Castellón, Spain; 581 Imperial College Healthcare NHS Trust, General Intensive Care Unit, London, UK; 582 Imperial College Healthcare NHS Trust, Haematology, London, UK; 583 Samsung Medical Center, Seoul, Republic of Korea; 584 Dalhousie University, Critical Care Medicine, Halifax, Canada; 585 Dalhousie University, Anesthesiology, Pain Management and Perioperative Medicine, Halifax, Canada; 586 Dalhousie University, Respiratory Therapy, Halifax, Canada; 587 Dalhousie University, Emergency Medicine, Halifax, Canada; 588 Queen Mary University of London, William Harvey Research Institute, London, UK; 589 London School of Economics, London, UK; 590 Northwick Park Hospital, London, UK; 591 Barts Health NHS Trust, London, UK; 592 Tata Memorial Hospital, Anaesthesia, Critical Care and Pain, Mumbai, India; 593 Erasme University Hospital, Université Libre de Bruxelles, Department of Intensive Care, Brussels, Belgium; 594 Insituto Mexicano del Seguro Social, Internal Medicine, Celaya, Mexico; 595 University Hospital Jena, Department of Internal Medicine IV, Jena, Germany; 596 University of Frankfurt, Frankfurt, Germany; 597 University Bonn, Bonn, Germany; 598 Hospital Frankfurt Hoechst, Frankfurt, Germany; 599 KfH Bad Soden, Bad Soden, Germany; 600 Ultrasound Network in Acute and Critical Care, Frankfurt, Germany; 601 Rigshospitalet, Copenhagen University Hospital, Intensive Care Unit 4131, Copenhagen, Denmark; 602 Lund University, Dep. of Clinical Sciences, Lund, Sweden; 603 Hospital de Clínicas de Porto Alegre, Intensive Care Unit, Porto Alegre, Brazil; 604 Hospital Femina Grupo Hopsitalar Conceição, Intensive Care, Porto Alegre, Brazil; 605 Hospital de Clínicas de Porto Alegre, Internal Medicine, Porto Alegre, Brazil; 606 Hospital Universitari i Politècnic la Fe, Intensive Care Unit, Valencia, Spain; 607 Hospital Universitari i Politècnic la Fe, Pharmacy, Valencia, Spain; 608 Hospital Universitari i Politècnic la Fe, Pneumology, Valencia, Spain; 609 University Hospital of Gran Canaria Dr. Negrín, Intensive Care Unit, Las Palmas de Gran Canaria, Spain; 610 University Hospital of Gran Canaria Dr. Negrín, Microbiology Department, Las Palmas de Gran Canaria, Spain; 611 University Hospital of Gran Canaria Dr. Negrín, Pharmacy Department, Las Palmas de Gran Canaria, Spain; 612 University of Las Palmas de Gran Canaria, Mathematics and Informatics Deparment, Las Palmas de Gran Canaria, Spain; 613 Hospital Virgen del Rocio, Seville, Spain; 614 Hospital Virgen Macarena, Seville, Spain; 615 Aix Marseille Université, Assistance Publique-Hôpitaux de Marseille, Hôpital Nord, Service d’Anesthésie et de Réanimation, Marseille, France; 616 EA 3279 Research Unit, Aix Marseille University & Assistance Publique-Hôpitaux de Marseille, Marseille, France; 617 Department of Clinical Medicine, Trinity College, Wellcome Trust-HRB Clinical Research Facility, St James Hospital, Dublin, Ireland; 618 Hopital Europeen Georges Pompidou, Anesthesiology and Intensive Care Medicine, Paris, France; 619 University of California Berkeley, Biostatistics, Berkeley, USA; 620 Hopital Saint Louis, Paris, France; 621 Hopital Europeen Georges Pompidou, Paris, France; 622 University of California Berkeley, Berkeley, USA; 623 Hospital Universitario Miguel Servet, ICU, Zaragoza, Spain; 624 Hospital Universitario Miguel Servet, Microbiology, Zaragoza, Spain; 625 Hospital de Barbastro, Barbastro, Spain; 626 HU Arnau de Vilanova, Lleida, Spain; 627 H Galdakao, Bilbao, Spain; 628 HU Vall d’Hebron, Barcelona, Spain; 629 HU Doce de Octubre, Madrid, Spain; 630 HU Malaga, Malaga, Spain; 631 Parc de Salut Mar. IMIM (Mar Medical Research Institut), Barcelona, Spain; 632 Yonsei Wonju University College of Medicine, Department of Internal Medicine, Wonju, Republic of Korea; 633 Yonsei University College of Medicine, Department of Internal Medicine, Seoul, Republic of Korea; 634 Yonsei University College of Medicine, AIDS Research Institute, Seoul, Republic of Korea; 635 Hospital Clinic, Pulmonary and Critical Care Medicine, Barcelona, Spain; 636 University of Milan, Fisiopatologia Medico-Chirurgica e dei Trapianti, Milano, Italy; 637 University of Milan, Fisiopatologia medico-chirurgica e dei trapianti, Milano, Italy; 638 Hospital Virgen de la Arrixaca, ICU, El Palmar, Spain; 639 Hospital Virgen de la Arrixaca, Pharmacy, El Palmar, Spain; 640 University of Murcia, Faculty of Veterinary, Murcia, Spain; 641 Hospital Universitari Arnau de Vilanova, Lleida, Spain; 642 Institut de Recerca Biomédica de Lleida, Lleida, Spain; 643 University of Foggia, Anaesthesia and Intensive Care, Foggia, Italy; 644 Complejo Hospitalario de Navarra, Intensive Care Medicine, Pamplona, Navarra Spain; 645 Complejo Hospitalario de Navarra, Neurosurgery, Pamplona, Navarra Spain; 646 Complejo Hospitalario de Navarra, Neurology, Pamplona, Navarra Spain; 647 Juntendo University School of Medicine, General Medicine, Tokyo, Japan; 648 Niijima, Tokyo National Health Insurance Clinic, Tokyo, Japan; 649 Jichii Medical University, Pediatrics, Tochigi, Japan; 650 Tokyo Metropolitan Government, Emergency Medical Services and Disaster Response Section, Tokyo, Japan; 651 All India Institute of Medical Sciences, Anesthesiology Critical Care and Pain Medicine, New Delhi, India; 652 ASST Sacco Fatebenefratelli, Milano, Italy; 653 ASST Grande Ospedale Metropolitano Niguarda, Milano, Italy; 654 NIHR Surgical Reconstruction and Microbiology Research Centre, Birmingham, UK; 655 Kings College Hospital, London, UK; 656 Heartfelt Technologies Ltd, Cambridge, UK; 657 Ente Ospedaliero Ospedali Galliera, Anestesia e Rianimazione, Genova, Italy; 658 Azienda Ospedaliero-Universitaria di Parma, Dipartimento Cardio-Nefro-Polmonare, UO di Cardiochirurgia, Parma, Italy; 659 Royal Brompton and Harefield Trust, Speech and Language Therapy, London, UK; 660 Complejo Hospitalario de Granada, Intensive Care Unit, Granada, Spain; 661 Hospital Regional Málaga, Málaga, Spain; 662 Complejo Hospitalario de Jaén, Jaen, Spain; 663 Hospital Puerta del Mar, Cadiz, Spain; 664 Hospital Virgen de las Nieves, Granada, Spain; 665 Hammersmith Hospital, Imperial College Healthcare NHS Trust, Department of Surgery and Cancer, London, UK; 666 Hospital Rio Hortega, Valladolid, Spain; 667 Istanbul University Cerrahpasa Medical Faculty, Anesthesiology and Intensive Care, Istanbul, Turkey; 668 University of Southampton, Faculty of Medicine, Southampton, UK; 669 Western Sussex Hospitals NHS FT, Worthing, UK; 670 University of Brighton, Brighton, UK; 671 Royal Surrey County Hospital, Intensive Care, Guildford, UK; 672 University of Surrey, Guildford, UK; 673 Southend University Hospital, Essex, UK; 674 University of Southampton, Faculty of Medicine, Southampton, UK; 675 Western Sussex Hospitals NHS FT, Worthing, UK; 676 University of Brighton, Brighton, UK; 677 Royal Surrey County Hospital, Intensive Care, Guildford, UK; 678 University of Surrey, Guildford, UK; 679 Dr Josep Trueta University Hospital, Intensive Care Unit, Girona, Spain; 680 Dr Josep Trueta University Hospital, Clinical laboratory, Girona, Spain; 681 Pavlov First Saint-Petersburg State Medical University, Anesthesiology and Intensive Care, Saint-Petersburg, Russian Federation; 682 Saint-Petersburg I. I. Dzhanelidze Research institute of Emergency Medicine, Saint-Petersburg, Russian Federation; 683 Vilnius University, Clinic of Anaesthesiology and Intensive Care, Vilnius, Lithuania; 684 Vilnius University, Faculty of Medicine, Vilnius, Lithuania; 685 Hadassah Hebrew University Medical Center, Jerusalem, Israel; 686 LeukoDx, Jerusalem, Israel; 687 Regional University Hospital in Málaga, Intensive Care Medicine, Málaga, Spain; 688 Queen Mary University of London, William Harvey Research Institute, London, UK; 689 The Royal London Hospital, Adult Critical Care Unit, London, UK; 690 Queen Mary University of London, London, UK; 691 Erasme University Hospital, Université Libre de Bruxelles, Brussels, Belgium; 692 Nord University, Faculty of Health Science, Levanger, Norway; 693 Nord-Trøndelag Hospital Trust, Levanger Hospital, Internal Medicine, Levanger, Norway; 694 Norwegian University of Technology and Science (NTNU), Department of Neuroscience (INM), Trondheim, Norway; 695 St Olavs University Hospital, Department of Infectious Diseases, Trondheim, Norway; 696 Norwegian University of technology and Science (NTNU), Centre of Molecular Inflammation Research, Department of Cancer Research and Molecular Medicine, Trondheim, Norway; 697 Norwegian University of Technology and Science (NTNU), Mid-Norway Sepsis Research Center, Trondheim, Norway; 698 St Olavs University Hospital, Clinic of Anesthesia and Intensive Care, Trondheim, Norway; 699 Norwegian University of Technology and Science (NTNU), Department of Circulation and Medical Imaging, Trondheim, Norway; 700 Norwegian University of Technology and Science (NTNU), Department of Cancer Research and Molecular Medicine, Unit for Applied Clinical Research, Trondheim, Norway; 701 Tokyo Women’s Medical University, Critical Care and Emergency Medicine, Tokyo, Japan; 702 Attikon University Hospital, 2nd Department of Critical Care, Athens, Greece; 703 Attikon University Hospital, 2nd Department of Internal Medicine, Athens, Greece; 704 National and Kapodistrian University of Athens, Department of Experimental Physiology, Athens, Greece; 705 Tokyo Women’s Medical University, Tokyo, Japan; 706 University College London, Faculty of Medical Sciences, London, UK; 707 Lille Univ Hospital, Lille, France; 708 Melun Hospital, Intensive Care Medicine Department, Melun, France; 709 Melun Hospital, Clinical Research Unit, Melun, France; 710 Plug Working Group, ESICM, Melun, France; 711 Technische Universität München, II. Medizinische Klinik, Munich, Germany; 712 Dr. Suat Seren Chest Diseases and Surgery Training Hospital, Intensive Care Unit, Izmir, Turkey; 713 National Cheng Kung University Hospital, Department of Internal Medicine, Tainan, Taiwan, Province of China; 714 National Cheng Kung University, Medical Device Innovation Center, Tainan, Taiwan, Province of China; 715 Hospital Quirón de Málaga, Intensive Care Unit, Málaga, Spain; 716 Hospital Universitario Reina Sofia, Intensive Care Unit, Córdoba, Spain; 717 Hospital of Manises, Intensive Care Unit, Valencia, Spain; 718 Complejo Hospitalario de Jaén, Intensive Care Unit, Jaén, Spain; 719 Réanimation Médicale, Hôpital de la Croix-Rousse, Lyon, France; 720 IMRB, INSERM 955, Créteil, France; 721 Hôpital de la Croix-Rousse, Réanimation Médicale, Lyon, France; 722 INSERM 955, Equipe 13, Créteil, France; 723 Papageorgiou General Hospital, Intensive Care Unit, Thessaloniki, Greece; 724 Cyprus University of Technology, Department of Electrical Engineering, Computer Engineering and Informatics, Limassol, Cyprus; 725 Erasmus Medical Center, Intensive Care Adults, Rotterdam, Netherlands; 726 Okayama University Hospital, Department of Clinical Engineering, Okayama City, Japan; 727 Okayama University Hospital, Advanced Emergency and Critical Care Center, Okayama City, Japan; 728 Kawasaki Medical School, Department of Acute Care & Primary Care Medicine, Kurashiki City, Japan; 729 Okayama University, Department of Medical Technology Graduate School of Health Sciences, Okayama City, Japan; 730 Fondazione IRCCS Policlinico S. Matteo, Anesthesia and Intensive Care, University of Pavia, Pavie, Italy; 731 University of Chieti, School of residency in Anesthesia, Intensive Care and Pain Therapy, Chieti, Italy; 732 Radboudumc, Intensive Care, Nijmegen, Netherlands; 733 Beijing Tiantan Hospital, Capital Medical University, Department of Critical Care Medicine, Beijing, China; 734 University of Sao Paulo, Faculty of Medicine, Hospital das Clínicas, Anesthesia, Critical Care and Pain, Sao Paulo, Brazil; 735 University of Sao Paulo, Faculty of Medicine, Hospital das Clínicas, Pulmonary and Critical Care, Sao Paulo, Brazil; 736 Hospital Sirio Libanes, Anesthesia, Critical Care and Pain, Sao Paulo, Brazil; 737 University of Sao Paulo, Faculty of Medicine, Hospital das Clínicas, Radiology, Sao Paulo, Brazil; 738 Okayama University Hospital, Department of Intensive Care, Okayama, Japan; 739 Okayama University Medical School, Department of Anesthesiology and Resuscitology, Okayama, Japan; 740 Asan Medical Center, University of Ulsan College of Medicine, Department of Anesthesiology and Pain Medicine, Laboratory for Cardiovascular Dynamics, Seoul, Republic of Korea; 741 Asan Medical Center, University of Ulsan College of Medicine, Department of Anesthesiology and Pain Medicine, Laboratory for Cardiovascular Dynamics, Seoul, Republic of Korea; 742 University Hospital of Parma, Parma, Italy; 743 E.O. Ospedali Galliera, Genoa, Italy; 744 Medical University of Vienna, Department of Anesthesiology, General Intensive Care and Pain Medicine, Vienna, Austria; 745 Medical University of Vienna, Department of Surgery/Div. of General Surgery, Vienna, Austria; 746 Medical University of Vienna, Department of Cranio-, Maxillofacial and Oral Surgery, Vienna, Austria; 747 Pirogov Russian National Research Medical University, Anesthesiology and Intensive Care Medicine, Moscow, Russian Federation; 748 Yokohama City University Graduate school of Medicine, Department of Anesthesiology and Critical Care, Yokohama, Japan; 749 Yamato Municipal Hospital, Department of Anesthesia, Yamato, Japan; 750 CHU Antoine Béclère APHP, Intensive Care Unit, Clamart, France; 751 CHU Antoine Béclère APHP, Department of Hematology, Clamart, France; 752 University Hospitals Birmingham NHS Trust, Critical Care, Birmingham, UK; 753 University of Toronto, Toronto, Canada; 754 Royal College of Surgeons in Ireland, Dublin, Ireland; 755 St Michael’s Hospital, Toronto, Canada; 756 Universidad Autonoma de México, POSGRADO, Mexico, Mexico; 757 Odessa National Medical University, Anesthesiology and Intensive Care, Odessa, Ukraine; 758 Odessa National Medical University, Odessa, Ukraine; 759 Hospital Virgen del Rocio, Sevilla, Spain; 760 Royal Alexandra Hospital, Anaesthetics and Intensive Care, Glasgow, UK; 761 Queen Elizabeth University Hospital, Anaesthetics and Intensive Care, Glasgow, UK; 762 Hospital Universitario Príncipe de Asturias, Intensive Care Unit, Madrid, Spain; 763 Hospital Universitario Príncipe de Asturias, Madrid, Spain; 764 Hospital Universitario Príncipe de Asturias, Hematology, Madrid, Spain; 765 Hospital Universitario Puerta de Hierro Majadahonda, Madrid, Spain; 766 Jahrom University of Medical Sciences, Jahrom, Islamic Republic of Iran; 767 University of Franche-Comté, Laboratory of Psychology EA 3188, Besançon, France; 768 CR CHUM, Montreal, Canada; 769 CHRU Minjoz, Besançon, France; 770 Complejo Hospitalario de Granada, Granada, Spain; 771 Hospital del SAS de Jerez, Intensive Care Unit, Jerez de la Frontera, Spain; 772 Hospital del SAS de Jerez, Jerez de la Frontera, Spain; 773 University Hospital Vall d’Hebron, Transplant Coordination, Barcelona, Spain; 774 University Hospital Vall d’Hebron, Stroke Unit, Neurology, Barcelona, Spain; 775 University Hospital Vall d’Hebron, Barcelona, Spain; 776 St Elisabeth Twee Steden Hospital, Intensive Care Medicine, Tilburg, Netherlands; 777 The Dutch Transplant Foundation, Leiden, Netherlands; 778 Radboud University Nijmegen Medical Centre, Nijmegen, Netherlands; 779 Clinical Hospital Center Sestre Milosrdnice, Anaesthesiology and Intensive Care, Zagreb, Croatia; 780 Hospital Universitario Virgen del Rocio, Coordinación de Trasplantes, Seville, Spain; 781 Hospital Universitario Virgen del Rocio, Critical Care, Seville, Spain; 782 Hospital Universitario Juan XXII, Critical Care, Tarragona, Spain; 783 Université Paris Diderot, Sorbonne Paris Cité, INSERM UMR1160, Paris, France; 784 APHP, Lariboisiere University Hospital, Surgical ICU, Paris, France; 785 Hospital Universitario Puerta de Hierro Majadahonda, Intensive Care Unit, Madrid, Spain; 786 Puerta de Hierro Hospital, Intensive Care Unit, Majadahonda, Spain; 787 Tan Tock Seng Hospital, National Healthcare Group, Anaesthesiology, Pain and Intensive Care, Singapore, Singapore; 788 Tan Tock Seng Hospital, National Healthcare Group, Singapore, Singapore; 789 Regional University Hospital in Málaga, Intensive Care Medicine, Málaga, Spain; 790 Hosiptal Son Llàtzer, Intensive Care Department, Palma de Mallorca, Spain; 791 Hospital Son Espases, Intensive Care Department, Palma de Mallorca, Spain; 792 Hospital de la Santa Creu i Sant Pau, Intensive Care Department, Barcelona, Spain; 793 Hospital Torrejón de Ardoz, Intensive Care Department, Madrid, Spain; 794 Hospital Infanta Leonor, Intensive Care Department, Madrid, Spain; 795 Hospital de Sant Joan Despí Moissès Broggi, Intensive Care Department, Sant Joan Despí, Spain; 796 Hospital Sant Joan de Déu, Intensive Care Department, Reus, Spain; 797 APHM, CHU Nord, Medical ICU, Marseilles, France; 798 APHM, CHU Timone, Medical ICU, Marseilles, France; 799 Mayo Clinic, Pulmonary and Critical Care, Rochester, USA; 800 Mayo Clinic, Center for the Science of Health Care Delivery, Rochester, USA; 801 Mayo Clinic, Mayo Foundation for Medical Education and Research, Rochester, USA; 802 Mayo Clinic, Anesthesia Clinical Research, Rochester, USA; 803 JA Hiroshima General Hospital, Pharmaceutical Department, Hatsukaiti-shi, Hiroshima Japan; 804 JA Hiroshima General Hospital, Division of Emergency and Critical Care Medicine, Hatsukaiti-shi, Hiroshima Japan; 805 Hokkaido University Hospital, Emergency and Critical Care Center, Sapporo, Hokkaido Japan; 806 Chang Gung Memorial Hospital, Taoyuan, Taiwan, Province of China; 807 Chang Gung Memorial Hospital, Linkou, Taoyuan City, Taiwan, Province of China; 808 Queen’s University, Critical Care Medicine, Kingston, Canada; 809 University of Western Ontario, Critical Care Medicine, London, Canada; 810 Hospital Municipal Moyses Deutsch, ICU, São Paulo, Brazil; 811 Hospital Municipal Moyses Deutsch, Quality Sector, São Paulo, Brazil; 812 KCMH, Nursing Department, Bangkok, Thailand; 813 Université Pierre et Marie Curie, UMR_S 1158 and Hôpital Pitié-Salpêtrière, Respiratory Division and Medical ICU, Paris, France; 814 Hôpital Dupuytren, Limoges, France; 815 CHU de Clermont-Ferrand and Université d’Auvergne, Clermont-Ferrand, France; 816 Université Grenoble-Alpes and CHU Grenoble Alpes, Grenoble, France; 817 CHU d’Angers, Angers, France; 818 Hospital Dr. Nélio Mendonça, Serviço de Medicina Intensiva, Funchal, Portugal; 819 Chia Nan University of Pharmacy and Science, Recreation and Health-Care Management, Tainan, Taiwan, Province of China; 820 Chi-Mei Medical Center, Intensive Care Medicine, Tainan, Taiwan, Province of China; 821 Chi-Mei Medical Center, Liouying District, Intensive Care Medicine, Tainan, Taiwan, Province of China; 822 Chi Mei Medical Center, Internal Medicine, Tainan, Taiwan, Province of China; 823 Chi-Mei Medical Center, Tainan, Taiwan, Province of China; 824 Yonsei University Wonju College of Medicine, Internal Medicine, Wonju, Republic of Korea; 825 Yonsei University Wonju College of Medicine, Emergency Medicine, Wonju, Republic of Korea; 826 Tokushima University Graduate School, Critical Care and Emergency Medicine, Tokushima, Japan; 827 Takarazuka City Hospital, Intensive Care Unit, Takarazuka, Japan; 828 Uzbekistan Research Center of Emergency Medicine, Tashkent, Uzbekistan; 829 Tashkent Institute of Postgraduate Medical Education, Tashkent, Uzbekistan; 830 Guglielmo da Saliceto Hospital, Intermediate Care Unit, Piacenza, Italy; 831 Guglielmo da Saliceto Hospital, Local Health Care Unit, Piacenza, Italy; 832 Stellenbosch University, Stellenbsoch, South Africa; 833 University of the Western Cape, Cape Town, South Africa; 834 Stellenbosch University, Physiotherapy, Stellenbsoch, South Africa; 835 Hospital Municipal Moyses Deutsch, ICU, Sao Paulo, Brazil; 836 St Georges NHS Foundation Trust, Cardiothoracic Intensive Care, London, UK; 837 Hospital Municipal Moyses Deutsch, ICU, São Paulo, Brazil; 838 Seoul National University Bundang Hospital, Critical Care Medicine, Seongnam, Republic of Korea; 839 Seoul National University Bundang Hospital, Internal Medicine, Seongnam, Republic of Korea; 840 Seoul National University Bundang Hospital, Anesthesiology, Seongnam, Republic of Korea; 841 Seoul National University Bundang Hospital, Emergency Medicine, Seongnam, Republic of Korea; 842 Intensive Care Unit, Hospital Universitario Fundación Alcorcón, Madrid, Spain; 843 Hospital Sírio-Libanês, Intensive Care Unit, São Paulo, Brazil; 844 Hospital Geral do Grajau, Intensive Care Unit, Sao Paulo, Brazil; 845 Hospital Sírio Libanes, Intensive Care Unit, São Paulo, Brazil; 846 Akdeniz University, Antalya, Turkey; 847 Ribeirão Preto Medical School, University of São Paulo, Division of Intensive Care, Department of Surgery and Anatomy, Ribeirão Preto, Brazil; 848 Intensive Care Unit of São Francisco Hospital, Ribeirão Preto, Brazil; 849 Ribeirão Preto Medical School, University of São Paulo, Social Medicine Department, Ribeirão Preto, Brazil; 850 Chi Mei Medical Center, Intensive Care Medicine, Tainan, Taiwan, Province of China; 851 Carnegie Mellon University, Robotics Institute, Pittsburgh, USA; 852 University of Pittsburgh, School of Nursing, Pittsburgh, USA; 853 University of Pittsburgh, Department of Critical Care Medicine, School of Medicine, Pittsburgh, USA; 854 Hospital Universitario Príncipe de Asturias, Alcalá de Henares, Madrid Spain; 855 Cairo University, Critical Care Medicine, Cairo, Egypt; 856 Clinique du Millénaire, Hérault, Montpellier, France; 857 Clinique du Millénaire, Montpellier, France; 858 CHU Grenoble, Anesthesiology and Intensive Care, Grenoble, France; 859 CHU Grenoble, Cardiac Surgery, Grenoble, France; 860 Erasme University Hospital, Université Libre de Bruxelles, Department of Intensive Care, Brussels, Belgium; 861 IRCCS - Istituto di Ricerche Farmacologiche “Mario Negri”, Milan, Italy; 862 University of Milan, Milan, Italy; 863 Istituto Zooprofilattico Sperimentale della Lombardia e dell’Emilia, Lodi, Italy; 864 Sacco Hospital, Milan, Italy; 865 VHS Medical Center, Emergency Medicine, Seoul, Republic of Korea; 866 Seoul National University Hospital, Emergency Medicine, Seoul, Republic of Korea; 867 IRCCS - Istituto di Ricerche Farmacologiche “Mario Negri”, Milan, Italy; 868 Kuopio University Hospital, Kuopio, Finland; 869 University of Milan, Milan, Italy; 870 North Karelia Central Hospital, Karelia, Finland; 871 Helsinki University Hospital, Helsinki, Finland; 872 Technische Universität München, Klinik für Anaesthesiologie, Munich, Germany; 873 Cardinal Massaia Hospital, Anesthesia and Intensive Care Unit, Asti, Italy; 874 ULB Université Libre de Bruxelles, Department of Intensive Care, Erasme University Hospital, Bruxelles, Belgium; 875 Seoul National University Hospital, Emergency Medicine, Seoul, Republic of Korea; 876 Seoul National University, Neurology, Seoul, Republic of Korea; 877 Erasme Hospital, Brussels, Belgium; 878 Network for Improving Critical Care Systems and Training (NICST), Colombo, Sri Lanka; 879 National Intensive Care Surveillance, Quality Secretariat Building, Castle Street Hospital for Women, Colombo, Sri Lanka; 880 Royal London Hospital, Barts Health, Adult Critical Care Unit, London, UK; 881 Colombo North Teaching Hospital Sri Lanka, Ragama, Sri Lanka; 882 Intensive Care National Audit & Research Centre, London, UK; 883 Office of Medical Services, Ministry of Health, Colombo, Sri Lanka; 884 Department of Clinical Medicine, Faculty of Medicine, University of Colombo Sri Lanka, Colombo, Sri Lanka; 885 Government Medical Officers’ Association, Colombo, Sri Lanka; 886 Lady Ridgeway Hospital for Children, Colombo, Sri Lanka; 887 Mahidol Oxford Tropical Medicine Research Unit, Faculty of Tropical Medicine, Mahidol University, Bangkok, Thailand; 888 Hospital Virgen de las Nieves, Medicina Intensiva, Granada, Spain; 889 University Medical Centre Göttingen, Department for Anaesthesiology, Göttingen, Germany; 890 University Medical Centre Göttingen, Department for Oral and Maxillofacial Surgery, Göttingen, Germany; 891 Sunnybrook Health Sciences Centre, Critical Care, Toronto, Canada; 892 Yokohama City Minato Red Cross Hospital, Department of Emergency and Critical Care Medicine, Yokohama, Japan; 893 Tokyo Medical and Dental University, Health Policy and Informatics Section, Department of Health Policy, Tokyo, Japan; 894 Center for Cancer Control and Information Services, Division of Health Services Research, Tokyo, Japan; 895 National Center for Child Health and Development, Department of Critical Care, Tokyo, Japan; 896 Intermed Hospital, Intensive Care Department, Ulaanbaatar, Mongolia; 897 Health Sciences University of Mongolia, Division of Emergency Medicine and Anaesthesia, Ulaanbaatar, Mongolia; 898 University Hospital Salzburg and Paracelsus Private Medical University, Department of Anesthesiology, Perioperative and General Intensive Care Medicine, Salzburg, Austria; 899 Hospital Insular Las Palmas GC, Las Palmas de Gran Canaria, Spain; 900 Hospital Insular Las Palmas GC, Department of Economy. University of Las Palmas de Gran Canaria, Las Palmas de Gran Canaria, Spain; 901 Rush University Medical Center, Chicago, USA; 902 Giannitsa General Hospital, Intensive Care Unit, Giannitsa, Greece; 903 G. Papanikolaou Hospital, A’ Intensive Care Unit, Thessaloniki, Greece; 904 Heart of England NHS Foundation Trust, Birmingham, UK; 905 Queen Elizabeth University Hospital, NHS GG&C, Intensive Care Unit, Glasgow, UK; 906 Golden Jubilee National Hospital, Department of Anaesthesia, Clydebank, UK; 907 Centro Hospitalar de S.João, Porto, Portugal; 908 University of Porto, Porto, Portugal; 909 Centro Hospitalar de São João/University of Porto, Porto, Portugal; 910 Tuen Mun Hospital, Anaesthesia & Intensive Care, Hong Kong, Hong Kong, China; 911 Pamela Youde Nethersole Eastern Hospital, Intensive Care, Hong Kong, Hong Kong China; 912 Shiraz University of Medical Sciences, Shiraz, Iran, Shiraz Anesthesiology and Critical Care Research Center, Shiraz, Islamic Republic of Iran; 913 Shiraz University of Medical Sciences, Shiraz Anesthesiology and Critical Care Research Center, Shiraz, Islamic Republic of Iran; 914 Department of Epidemiology, School of Public Health - Shiraz University of Medical Sciences, Shiraz, Islamic Republic of Iran; 915 Student Research Committee - Shiraz University of Medical Sciences, Shiraz, Islamic Republic of Iran; 916 AIIMS Bhopal, Department of Trauma & Emergency, Bhopal, India; 917 AIIMS Bhopal, Community and Family Medicine, Bhopal, India; 918 AIIMS Bhopal, Medicine, Bhopal, India; 919 Apollo Hospitals, Critical Care Unit, Bhubaneswar, India; 920 Apollo Hospitals, Bhubaneswar, India; 921 University Hospital of Liège, General Intensive Care Unit and Burn Centre, Liège, Belgium; 922 University of Liège, Liège, Belgium; 923 University Hospital of Liège, Clinical Chemistry Department, Liège, Belgium; 924 University of Liège, Department of Public Health, Epidemiology and Health Economics, Liège, Belgium; 925 Sir Run Run Shaw Hospital, Intensive Care Medicine, Hanghzou, China; 926 ZNA Middelheim General Hospital, Department of Anaesthesiology, Antwerp, Belgium; 927 University of Antwerp, Department of Pharmaceutic Sciences, Antwerp, Belgium; 928 ZNA Middelheim General Hospital, Department of Cardiac Surgery, Antwerp, Belgium; 929 Hospital 12 de Octubre, Cardiac Intensive Care Unit, Critical Care Department, Madrid, Spain; 930 Fooyin University, Department of Physical Therapy, Kaohsiung City, Taiwan, Province of China; 931 Kaohsiung Veterans General Hospital, Cardiovascular Division, Kaohsiung City, Taiwan, Province of China; 932 Kaohsiung Veterans General Hospital, Critical Care Division, Kaohsiung City, Taiwan, Province of China; 933 Sir Run Run Shaw Hospital, Intensive Care Medicine, Hanghzou, China; 934 Pusan National University Yangsan Hospital, Anesthesia and Pain Medicine, Yangsan-si, Republic of Korea; 935 Korea University Anam Hospital, Anaesthesiology and Pain Medicine, Seoul, Republic of Korea; 936 Hospital Puerta del Mar, Cadiz, Spain; 937 Hospital Regional, Intensive Care, Malaga, Spain; 938 Hospital Serrania, Ronda, Spain; 939 Hospital Regional, Intensive Care, Málaga, Spain; 940 Hospital Infanta Margarita, Intensive Care, Cabra, Spain; 941 Hospital Virgen del Rocio, Intensive Care, Sevilla, Spain; 942 Hospital Virgen de las Nieves, Intensive Care, Granada, Spain; 943 Complejo Hospitalario de Navarra, Intensive Care Medicine, Pamplona, Spain; 944 Hospital Universitario Central de Asturias, ICU 1, Oviedo, Spain; 945 Hospital Universitario Central de Asturias, ICU 3, Oviedo, Spain; 946 Hospital Universitario Central de Asturias, Oviedo, Spain; 947 Complejo Hospitalario de Navarra, Intensive Care Medicine, Pamplona, Spain; 948 ASST Sacco Fatebenefratelli, Milano, Italy; 949 Kaohsiung Veterans General Hospital, Critical Care Division, Kaohsiung City, Taiwan, Province of China; 950 Kaohsiung Veterans General Hospital, Cardiovascular Division, Kaohsiung City, Taiwan, Province of China; 951 Kaohsiung Veterans General Hospital, Department of Medical Education and Research, Kaohsiung City, Taiwan, Province of China; 952 University Hospital of Pisa, Department of Anaesthesia and Critical Care Medicine, Cardiothoracic and Vascular Anaesthesia, Pisa, Italy; 953 University of Pisa, Scuola di Specializzazione in Malattie del l’Apparato Cardiovascolare, Pisa, Italy; 954 University Hospital of Pisa, Department of Anaesthesia and Critical Care Medicine, Cardiothoracic and Vascular Anaesthesia, Pisa, Italy; 955 OLVG Hospital, Department of Intensive Care, Amsterdam, Netherlands; 956 Tilburg University; TIAS School for Business and Society, Tilburg, Netherlands; 957 Tokushima University Hospital, Emergency and Disaster Medicine, Tokushima, Japan; 958 Tokushima University Graduate School, Emergency and Critical Care Medicine, Tokushima, Japan; 959 Fortis Escorts Hospital, Critical Care Medicine, Faridabad, India; 960 Fortis Escorts Hospital, Microbiology, Faridabad, India; 961 Niguarda Ca Granda Hospital, Anesthesia CCM 2, Milan, Italy; 962 Niguarda Ca Granda Hospital, Milan, Italy; 963 National Scientific Medical Research Center, Clinical Microbiology, Astana, Kazakhstan; 964 Ankara University Faculty of Medicine, Department of Internal Medicine Division of Intensive Care, Ankara, Turkey; 965 Ankara University Faculty of Medicine, Clinical Microbiology and Infection Disease, Ankara, Turkey; 966 Woodlands Multispeciality Hospital, Clinical Microbiology Dept, Kolkata, India; 967 Woodlands Multispeciality Hospital, Nephrology Dept, Kolkata, India; 968 Cure Clinic Nursing Home, Kolkata, India; 969 HU Arnau de Vilanova, Lleida, Spain; 970 IRBLL, Lleida, Spain; 971 Hospital Universitario de Fuenlabrada, Intensive Care Unit, Madrid, Spain; 972 Universidade Federal do Rio de Janeiro, ICU, Rio de Janeiro, Brazil; 973 Universidade Federal do Rio de Janeiro, Infectious Diseases, Rio de Janeiro, Brazil; 974 Hospital Universitario Arnau de Vilanova, Lleida, Spain; 975 Yildirim Beyazit University, Anesthesiology and Reanimation, Ankara, Turkey; 976 Yildirim Beyazit University, Biostatistics and Medical Informatics, Ankara, Turkey; 977 Yildirim Beyazit University, Infectious Diseases and Microbiology, Ankara, Turkey; 978 University Hospital of Gran Canaria Dr. Negrín, Intensive Care Unit, Las Palmas de Gran Canaria, Spain; 979 University Hospital of Gran Canaria Dr. Negrín, Pharmacy Department, Las Palmas de Gran Canaria, Spain; 980 University Hospital of Gran Canaria Dr. Negrín, Microbiology Department, Las Palmas de Gran Canaria, Spain; 981 Hospital of Getafe, Intensive Care Unit, Madrid, Spain; 982 University of Las Palmas de Gran Canaria, Mathematics and Informatcs Deparment, Las Palmas de Gran Canaria, Spain; 983 Intensive Care Unit, Ambroise Paré University Hospital, Assistance Publique, Hôpitaux de Paris, Boulogne-Billancourt, France; 984 Infection control Unit, Ambroise Paré University Hospital, Assistance Publique, Hôpitaux de Paris, Boulogne-Billancourt, France; 985 Infectious Diseases Unit, Ambroise Paré University Hospital, Assistance Publique, Hôpitaux de Paris, Boulogne-Billancourt, France; 986 Microbiology Unit, Section Biology Pathology and Health Products, Ambroise Paré University Hospital, Assistance Publique, Hôpitaux de Paris, Boulogne-Billancourt, France; 987 Intensive Care Unit, Ambroise Paré University Hospital, Assistance publique, Hôpitaux de Paris, Boulogne-Billancourt, France; 988 Intensive Care Unit, Ambroise Paré University Hospital, Assistance Publique - Hôpitaux de Paris, Boulogne-Billancourt, France; 989 Rescu, Li Ka Shing Institute at St Michael‘s Hospital, Toronto, Canada; 990 Intensive Care unit, Ambroise Paré University Hospital, Assistance Publique, Hôpitaux de Paris, Boulogne-Billancourt, France; 991 University of Versailles Saint-Quentin en Yvelines, Faculty of Medicine Paris Ile-de-France Ouest, Saint-Quentin en Yvelines, France; 992 INSERM U-1018, CESP, Team 5 (EpReC, Renal and Cardiovascular Epidemiology), Villejuif, France; 993 Papageorgiou Hospital, Intensive Care Unit, Thessaloniki, Greece; 994 Hôpital de Bicêtre, Hôpitaux universitaires Paris-Sud, Université Paris-Sud, Service de réanimation médicale, Inserm UMR_S999, Le Kremlin Bicêtre, France; 995 Hôpital Ambroise Paré, Hôpitaux universitaires Paris-Ile-de-France-Ouest, Université de Versailles Saint Quentin en Yvelines, Service de réanimation polyvalente, Boulogne-Billancourt, France; 996 Centre hospitalier de Sens, Service de réanimation polyvalente, Sens, France; 997 CHU Tenon, APHP, Medical and Surgical ICU, Paris, France; 998 University of Toronto, Interdepartmental Division of Critical Care, Toronto, Canada; 999 Université Paris Diderot, Sorbonne Paris Cité, UMR 1153, Paris, France; 1000 Rouen University Hospital, Medical Intensive Care, Rouen, France; 1001 Rouen University Hospital, UPRES EA3830 IRIB, Rouen, France; 1002 CHU Henri Mondor, Medical ICU, Créteil, France; 1003 University Hospital of Lausanne, Intensive Care and Burn Unit, Lausanne, Switzerland; 1004 CHU d’Amiens, Cardiothoracic and Vascular ICU, Amiens, France; 1005 Université Jules Verne, Picardie, INSERM U1088 - CURS, Amiens, France; 1006 CHU Hotel Dieu, Medical ICU, Nantes, France; 1007 Hospital Le Mans, Intensive Care Unit, Le Mans, France; 1008 CHR d’Orleans, Medical ICU, Orleans, France; 1009 CHU Grenoble Alpes, Medical ICU, Grenoble, France; 1010 Université Grenoble-Alpes, Inserm U1042, Grenoble, France; 1011 CHR de Mulhouse, Medical ICU, Mulhouse, France; 1012 Rouen University Hospital, Surgical Intensive Care, Rouen, France; 1013 CHU de Poitiers, Medical ICU, Poitiers, France; 1014 Université de Poitiers, INSERM, CIC- 1402, Équipe 5 ALIVE, Poitiers, France; 1015 CHU Henri Mondor and ReVA Network, Medical ICU, Créteil, France; 1016 CHU d’Angers, Clinical Research Institute, Angers, France; 1017 Hospital de Sant Pau, Servei de Medicina Intensiva, Barcelona, Spain; 1018 CHU d’Angers, Medical ICU, Angers, France; 1019 Annecy Genevois General Hospital, Annecy, France; 1020 Saint Michael’s Hospital and Keenan Research Centre, Interdepartmental Division of Critical Care, Toronto, Canada; 1021 Rīga Stradiņš University, Faculty of Continuing Education, Residency Section, Riga, Latvia; 1022 Rīga Stradiņš University, Department of Anaesthesiology & Intensive Care, Riga, Latvia; 1023 Pauls Stradins Clinical University Hospital, Department of Intensive Care, Riga, Latvia; 1024 Fondazione IRCCS Policlinico S. Matteo, Anesthesia and Intensive Care, University of Pavia, Pavie, Italy; 1025 Rouen University Hospital, Medical Intensive Care, Rouen, France; 1026 Rouen University Hospital, UPRES EA3830 IRIB, Rouen, France; 1027 CHU Tenon, APHP, Medical and Surgical ICU, Paris, France; 1028 University of Toronto, Interdepartmental Division of Critical Care, Toronto, Canada; 1029 Université Paris Diderot, Sorbonne Paris Cité, UMR 1153, Paris, France; 1030 CHU Henri Mondor, Medical ICU, Créteil, France; 1031 University Hospital of Lausanne, Intensive Care and Burn Unit, Lausanne, Switzerland; 1032 CHU d’Amiens, Cardiothoracic and Vascular ICU, Amiens, France; 1033 Université Jules Verne, Picardie, INSERM U1088 - CURS, Amiens, France; 1034 CHU Hotel Dieu, Medical ICU, Nantes, France; 1035 Hospital Le Mans, Intensive Care Unit, Le Mans, France; 1036 CHR d’Orleans, Medical ICU, Orleans, France; 1037 CHU Grenoble Alpes, Medical ICU, Grenoble, France; 1038 Université Grenoble-Alpes, Inserm U1042, Grenoble, France; 1039 CHR de Mulhouse, Medical ICU, Mulhouse, France; 1040 Rouen University Hospital, Surgical Intensive Care, Rouen, France; 1041 CHU de Poitiers, Medical ICU, Poitiers, France; 1042 Université de Poitiers, INSERM, CIC- 1402, Équipe 5 ALIVE, Poitiers, France; 1043 CHU Henri Mondor and ReVA Network, Medical ICU, Créteil, France; 1044 CHU d’Angers, Clinical Research Institute, Angers, France; 1045 Hospital de Sant Pau, Servei de Medicina Intensiva, Barcelona, Spain; 1046 CHU d’Angers, Medical ICU, Angers, France; 1047 Annecy Genevois General Hospital, Annecy, France; 1048 Saint Michael’s Hospital and Keenan Research Centre, Interdepartmental Division of Critical Care, Toronto, Canada; 1049 University of Crete, School of Medicine, Intensive Care, Heraklio, Crete Greece; 1050 University Hospital of Heraklio, Intensive Care, Heraklio, Crete Greece; 1051 General Hospital of Larissa, Intensive Care, Larissa, Greece; 1052 Aristotle University of Thessaloniki, Lab of Medical Informatics, Thessaloniki, Greece; 1053 Asklepios Schlossberg Klinik, Early Neurological Rehabilitation, Bad König, Germany; 1054 University Heidelberg, Anaesthesiology and Intensiv Care Medicine, Heppenheim, Germany; 1055 Fondazione IRCCS Policlinico S. Matteo, Anesthesia and Intensive Care, University of Pavia, Pavie, Italy; 1056 Hospital Quirón de Málaga, Intensive Care Unit, Málaga, Spain; 1057 Hospital Universitario Reina Sofía, Intensive Care Unit, Córdoba, Spain; 1058 Hospital de Manises, Valencia, Spain; 1059 Complejo Hospitalario de Jaén, Jaén, Spain; 1060 University of Ferrara/Intensive Care Unit, Morphology, Surgery and Experimental Medicine, Ferrara, Italy; 1061 Aalborg University, Respiratory and Critical Care group (rcare) Center for Model-based Medical Decision Support (MMDS) Department of Health Science and Technology, Aalborg, Denmark; 1062 University of Milan, Department of Anesthesia, Critical Care and Emergency Fondazione IRCCS Ca’ Granda Ospedale Maggiore Policlinico, Milano, Italy; 1063 Peoples’ Friendship University of Russia, Anesthesiology & Intensive Care, Moscow, Russian Federation; 1064 Hospices Civils de Lyon, Hôpital de la Croix Rousse, Service de Reanimation Médicale, Lyon, France; 1065 Université de Lyon, Université Claude Bernard Lyon 1, Villeurbanne, France; 1066 INSERM, 955 Equipe 13, Créteil, France; 1067 Erasme University Hospital/Université Libre de Bruxelles, Department of Intensive Care, Brussels, Belgium; 1068 University of Helsinki and Helsinki University Hospital, Helsinki, Finland; 1069 Lapland Central Hospital, Rovaniemi, Finland; 1070 West China Hospital, Sichuan University, Department of Critical Care Medicine, Chengdu, China; 1071 Kobe University Hospital, Anesthesiology, Kobe, Japan; 1072 Alexandria University, Critical Care Medicine, Alexandria, Egypt; 1073 Alexandria University, Internal Medicine-Nephrology Unit, Alexandria, Egypt; 1074 Alexandria University, Clinical and Chemical Pathology, Alexandria, Egypt; 1075 Centro Hospitalar Lisboa Norte, Nephrology and Renal Transplantation, Lisbon, Portugal; 1076 Centro Hospitalar Lisboa Norte, Intensive Care Medicine, Lisbon, Portugal; 1077 Royal Surrey County Hospital, ICU and SPACeR research group, Guildford, UK; 1078 University of Surrey, Guildford, UK; 1079 University of Semmelweis, Budapest, Hungary; 1080 Royal Surrey County Hospital, Guildford, UK; 1081 Aomori Jekeikai Hospiyal, Clinical Engineer, Aomori, Japan; 1082 Clinica Universidad de Navarra, Anesthesia and Critical Care Unit, Pamplona, Spain; 1083 Clinica Universidad de Navarra, Nephrology, Pamplona, Spain; 1084 Uppsala University, Surgical Sciencies, Uppsala, Sweden; 1085 Centre Hospitalier Universitaire Vaudois, Adult Intensive Care Unit, Lausanne, Switzerland; 1086 Centre Hospitalier Universitaire Vaudois, Anaesthesiology Department, Lausanne, Switzerland; 1087 University of Melbourne, Intensive Care Medicine, Melbourne, Australia; 1088 Royal Brompton and Harefield NHS Foundation Trust, London, UK; 1089 Barts Health NHS Trust, Adult Critical Care, London, UK; 1090 Nikisso Medical, London, UK; 1091 Hospital Universitario San Ignacio, Internal Medicine, Nephrology, Bogota, Colombia; 1092 Pontificia Universidad Javeriana, Nefrology, Bogota, Colombia; 1093 Pontificia Universidad Javeriana, Internal Medicine, Bogota, Colombia; 1094 Hospital Militar Central, Nephrology, Bogota, Colombia

## CHILDREN, TEENAGER AND FAMILIES IN THE ICU

### A793 Malnutrition and clinical outcomes in critically ill children

#### T. Velasquez^1^, G. Mackey^2,3^, J. Lusk^2,3^, U.G. Kyle^2,3^, T. Fontenot^2^, P. Marshall^2^, L.S. Shekerdemian^2,3^, J.A. Coss-Bu^2,3^

##### ^1^Texas Children's Hospital, Clinical Nutriiton, Houston, United States; ^2^Texas Children's Hospital, Intensive Care, Houston, United States; ^3^Baylor College of Medicine, Pediatrics, Houston, United States

###### **Correspondence:** J.A. Coss-Bu – Texas Children's Hospital, Intensive Care, Houston, United States

**Introduction:** Critically ill children in the pediatric intensive care unit (PICU) are at high risk for developing nutritional deficiencies and undernutrition is known to be a risk factor for morbidity and mortality. Malnutrition represents a continuous spectrum ranging from marginal nutrient status to severe metabolic and functional alterations and this in turn, affects clinical outcome.

**Objectives:** The aim of the study was to assess nutritional status of critically ill children admitted to the PICU and its association to clinical outcomes.

**Methods:** Critically ill children age 6 months to 18 years were prospectively enrolled on PICU admission. Nutritional status was assessed by weight for age (WFA: underweight), weight for height (WFH: wasting), height for age (HFA: stunting) z-scores and mid upper arm circumference (MUAC: wasting) according to the WHO. (1,2) Malnutrition was defined as mild, moderate, and severe if z-scores were > −1, > − 2, and > −3, respectively. Hospital and PICU length of stay (LOS), duration of mechanical ventilation (MV), and risk of mortality (ROM) by the Pediatric Index of Mortality 2 (PIM2) were obtained. Sensitivity and specificity of the MUAC to identify children with wasting (WFH) were calculated.

**Results:** Two hundred and fifty children (136 males), aged 81 months (23–167; median (25-75^th^ IQR)), were prospectively included in the study. The hospital LOS was 8 (4–16) days; PICU LOS: 2 (1–4) days; duration of MV, 0 (0–1.5) days; PIM2 ROM 2.61 ± 0.25 %. WFA, WFH, and HFA z-scores of −0.48 ± 0.14; 0.19 ± 0.13; and −0.95 ± 0.13 respectively; MUAC, 16.3 ± 0.18 cm (6 to 59 months, n = 108); 24.2 ± 0.46 cm (5 to 18 years, n = 142). The prevalence of underweight, wasting and stunting was 26.4 %, 19.6 %, and 44.4 % respectively. The sensitivity and specificity for MUAC vs. WFH to identify wasting was: 34.5 % (20.3-50.6; 95 % CI) and 95.5 % (91.8-97.9), respectively. Values are mean ± SE.

**Conclusions:** Malnutrition in critically ill children is prevalent with half of the patients being stunted, reflecting the chronic nature of the disease process and its effects on the nutritional status. The performance of MUAC as a screening tool in this population was poor, but identified correctly almost all children with wasting. There was an association between nutritional status and length of stay and risk of mortality.

**References**

1 WHO: Technical Report Series, No. 854, 1995

2 Bulletin of the WHO, 1997, 75:11–18

**Grant acknowledgement**

Internal

**FUNDING**

Texas Children´s Hospital

**Table 1 (abstract A793).** Malnutriiton and Outcomes

	Underweight			Wasting			Stunting		
	Odds Ratio	95 % C.I.	p value	Odds Ratio	95 % C.I.	p value	Odds Ratio	95 % C.I.	p value
Hospital LOS	2.40	1.16–4.99	0.019	2.26	1.17–4.36	0.015	1.05	0.64–1.73	0.854
ICU LOS	2.17	1.05–4.49	0.037	2.28	1.05–4.95	0.037	2.33	1.14–4.79	0.021
PIM2 ROM	1.16	1.05–1.28	0.004	1.08	1.01–1.17	0.034	1.12	1.01–1.23	0.024

*LOS* length of stay, *PIM2* pediatric index of mortality, *ROM* risk of mortality

### A794 Retrospective analysis for predicting optimal tracheal tube size in pediatric patients

#### A. Nishigaki, T. Yatabe, T. Tamura, K. Yamashita, M. Yokoyama

##### Kochi Medical School, Department of Anesthesiology and Intensive Care Medicine, Nankoku, Japan

###### **Correspondence:** A. Nishigaki – Medical School, Department of Anesthesiology and Intensive Care Medicine, Nankoku, Japan

**Introduction:** There are several methods to estimate the optimal tracheal tube size in pediatric patients such as the Cole's formula (inner diameter (ID) = 4 + Age/4) [1]. However, these evaluation methods are made based on age in years (not months) and ID. Moreover, outer diameter (OD) may vary according to the type of the tracheal tube.

**Objectives:** We hypothesized that prediction of OD for determining the optimal tracheal tube size in pediatric patients based on age in months is better than Cole's formula. Therefore, we conducted a retrospective analysis to investigate our hypothesis.

**Methods:** The ethics committee of our hospital approved this retrospective study. We included consecutive patients aged < 6 years who underwent tracheal intubation under general anesthesia in our hospital from August 2013 to October 2015. We collected the following data from the anesthesia records: age in months, height, weight, type of a tracheal tube, and ID and OD of tracheal tube. Patients who were intubated using a cuffed tracheal tube or had incomplete data were excluded. We developed a regression formula for calculating ID and OD based on age in months and calculated the coefficient of determination R^2^ by using a regression analyses. A difference of 0.4 mm in the actual and predicted tube size was considered clinically permissible. Then, we compared the rate of a clinical permissible estimation of the Cole's formula and our new formulas used by multiple comparison analysis and a p value less than 0.05 was considered statistically significant.

**Results:** A total of 207 pediatric patients received general anesthesia during the study period. Of these, 67 patients were excluded because they did not meet the inclusion criteria. Finally, we included 140 patients for this analysis. The regression formula for predicting ID by based on age in months was ID = 0.019 × age in months + 3.48, and the coefficient of determination R^2^ was 0.54. The regression formula for predicting OD based on age in months was OD = 0.024 × age in months + 5.21, and coefficient of determination R^2^ was 0.558. The rate of a clinical permissible estimation of our ID and OD formulas were significantly higher than that of the Cole's formula (61 %, 69 % and 43 %, respectively; p < 0.01).

**Conclusions:** Our results showed that the prediction of ID based on age in months is more useful than that using Cole's formula. In addition, estimation of OD based on age in months might be more rational because OD varies according to the type of the tracheal tube used. These results should be confirmed in a future prospective study.

**References**

[1] Cole F. Pediatric formulas for the anesthesiologist. AMA J Dis Child. 1957;94:672–3.

### A795 Teenagers perception towards cardiopulmonary resuscitation

#### J.-C. Ruiz-Rodriguez^1^, B. Encina^1^, R. Belmonte^1^, I. Troncoso^1^, P. Tormos^2^, M. Riveiro^3^, J. Baena^3^, A. Sanchez^1^, J. Bañeras^4^, J. Cordón^1^, N. Duran^5^, A. Ruiz^1^, J. Caballero^1^, X. Nuvials^1^, J. Riera^1^, J. Serra^1^

##### ^1^Vall d' Hebron University Hospital, Critical Care Department, Barcelona, Spain; ^2^Vall d' Hebron University Hospital, Anesthesia & Reanimation Department, Barcelona, Spain; ^3^Vall d' Hebron University Hospital, Neurocritical Care Department, Barcelona, Spain; ^4^Vall d' Hebron University Hospital, Coronary Care Unit, Barcelona, Spain; ^5^Sagrat Cor University Hospital, Critical Care Department, Barcelona, Spain

###### **Correspondence:** J.-C. Ruiz-Rodriguez – Vall d' Hebron University Hospital, Critical Care Department, Barcelona, Spain

**Introduction:** Survival among out-of-hospital cardiac arrest (CA) relies primarily on bystanders and their knowledge of basic life support (BLS) manouvers [1]. Many medical societies and organizations recommend teaching BLS at schools as part of the educative program [2]; being this a reality in North European countries, but not yet an education standard issue in others including Spain. Moreover, less is written about the perception of CA and cardiopulmonary resuscitation (CPR) among the general population, and even less in school age.

**Objectives:** Describe the perception and knowledge about CA and CPR among a teenager school population in Barcelona, Spain.

**Methods:** Prospective, descriptive study carried out between 2007–2009 and 2012–2015 among teenagers school population, based on surveys before and after BLS - CPR classes. During this period , 17th classes were held, in 3 different schools in Barcelona. Before attending the class , each pupil was asked to answer a survey with questions related to previous knowledge of sudden death, CA, and CPR, and their attitude towards them. The class consisted on a three - hour theorical and practical instruction based on the European Resuscitation Council guidelines, adapted for laypersons. Practices were held with an instructor (ratio instructor:pupil 1:6–8), with the Little Anne mannequins (Laerdal®).

After the class, a new survey (post intervention) was distributed, with questions related to the new concepts and skills learnt, the attitude toward CA and CPR.

**Results:** We have instructed 561 pupils (14.02 (±0.79) years, 48.2 % female). The 87.8 % had heard about sudden death and CA before the class. Regarding starting CPR: 40 % said they were not capable of doing it, and 51.2 % suggested they would be able to do CPR but in a wrong manner. In a CA scene 58.9 % would contact the emergency service and start CPR, 27.4 % would call and wait, and 11.4 % would only do CPR. After attending the classes 98.6 % declared had understood the theorical concepts and practical skilles taught; 95.4 % would changed positively their attitude towards CPR; and 97.4 % would be prone to start maneuvers.

**Conclusions:** CPR and CA remain a well known issue among teenager population in Barcelona, as long as being an interesting topic. Nevertheless they do not feel capable of starting maneuvers. The concepts taught during the class were easy to learn , and after the intervention the majority were prone to start CPR. This population is adequate to teach CPR.

**References**

1. Hansen CM et al. The role of bystanders, first responders, and emergency medical service providers in timely defibrillation and related outcomes after out-of-hospital cardiac arrest: Results from a statewide registry. *Resuscitation* 2015;96:303–9.

2. Böttiger BW et al. Kids save lives--Training school children in cardiopulmonary resuscitation worldwide is now endorsed by the World Health Organization (WHO). *.Resuscitation.* 2015;94:A5-7.

### A796 Family = children included , guidance of visiting children at an adult intensive care

#### A.M.F. Rutten, S.N.M. van Ieperen, E.P.H.M. Der Kinderen, T. Van Logten

##### St Elisabeth Twee Steden Hospital, ICU, Tilburg, Netherlands

###### **Correspondence:** S.N.M. van Ieperen – St Elisabeth Twee Steden Hospital, ICU, Tilburg, Netherlands

**Introduction:** To meet the need of patients family members and staff we started to guide visiting children at our adult ICU in the St Elisabeth hospital (EZ) in Tilburg 3 years ago. To do so we developed a guidance leaflet for parents with practical instructions and information. Additionally, practical advice is given, such as what to say to the child and what to expect when visiting. The leaflet is subdivided in developmental stages. Furthermore we developed a book “mees op bezoek”, in which a child visits his father at the ICU. Pictures show what children can expect, which helps prepare the child for visiting at home. An instruction box is present at the ICU with ICU materials such as an iv catheter, a pulse oximetry or a tracheal tube. These materials give children a tactile experience of the ICU. The box is divided in two parts; the second part contains guidance materials for when a patient may die. Pedagogical staff are available to support parents, children and staff. If there are more profound problems a referral to our children's psychologist is possible. We made some improvements to our waiting area to make it more appealing to children. We instructed and educated our nurses and doctors on how to use these materials and how to guide children.

We recently merged with the Twee steden Hospital in Tilburg (TSZ), in this hospital there was no program to guide children. With the merger we also wanted to introduce our “Child as a visitor program” at the ICU on location TSZ. We wanted to know if there were differences of opinion between the nursing staff on guidance of children.

**Methods:** We held a survey among our nursing staff. In TSZ we handed out surveys on paper during an obligatory education. In EZ the same survey was sent by email.

**Results:** Response rate in EZ was 61 % (n = 127). Respons rate nursing staff in TSZ was 100 % (n = 33). Nearly all nurses share the view that children should be allowed to visit an ICU: EZ 97 % and TSZ 94 %. The appropriate age for children was deemed higher in TSZ with an average of 2,5 years, in EZ this was 0,74 years. 65 % of the nurse in EZ responded that children of all ages were welcome versus 33 % in TSZ. In EZ 44 % of the nurses didn't need any more support to guide children. In TSZ this was 3 %. 88 % of the nurses in TSZ wanted more education on the subject. In EZ there was still a great need: 56 % wanted this. More help from pedagogical staff was needed in 53 % of the nurses but in EZ this was 14 %. EZ 9 % needed more informational materials in TSZ this was 40 %.(see graph 1).

**Conclusion:** When you allow children to visit your ICU, nurses want to be educated on the subject, they need practical aids and help from pedagogical staff. The need for more pedagogical help and practical aids are less with the nurses who have more experience. A need for education on the subject will remain. We are introducing the “Child as a visitor program” at the ICU on location TSZ and will expand education in EZ.

**Fig. 1 (abstract A796).**
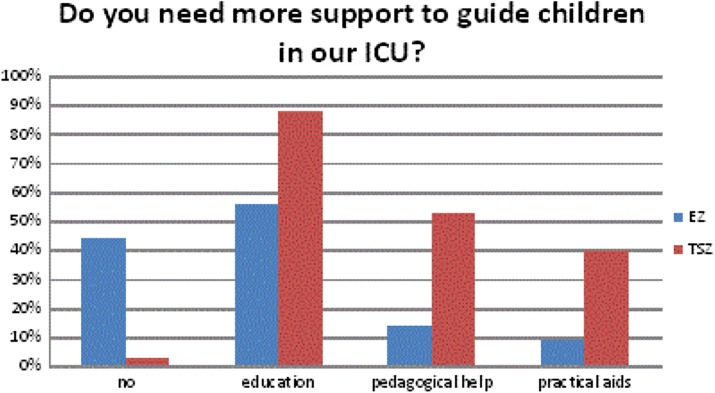
Do you need more support to guide children in our ICU?

### A797 High-frequency chest wall oscillation therapy in pediatric cardiac intensive care unit

#### L. Kovacikova^1^, P. Skrak^2^, M. Zahorec^3^

##### ^1^National Institute of Cardiovascular Diseases, PCICU, Bratislava, Slovakia; ^2^National Institute of Cardiovascular Diseases, Bratislava, Slovakia; ^3^National Institute of Cardiovascular Diseases, Pediatric Cardiac Intensive Care Unit, Bratislava, Slovakia

###### **Correspondence:** L. Kovacikova – National Institute of Cardiovascular Diseases, PCICU, Bratislava, Slovakia

**Introduction:** In critically ill children with cardiac diseases lung complications are frequently highlighting atelectasis and pneumonia. Physiotherapy has an important role in the treatment of these complications. High Frequency Chest Wall Oscillation (HFCWO) has been shown to be effective in helping to clear secretions from the lungs of patients with cystic fibrosis, primary ciliary dyskinesia, bronchiectasis and others. However, the role of HFCWO in children with cardiac diseases has not been established.

**Objectives:** This prospective observational study was conducted to determine if HFCWO treatment, as provided by The Vest™ Airway Clearance System (Hill-Rom, Saint Paul, MN), was safe and tolerated by these patients.

**Methods:** Eighty-five treatment courses were evaluated in 25 pediatric cardiac patients during the stay at intensive care unit. Median age of the patients was 2 months (range; 12 days - 7 years) and weight 4.2 kg (range; 2.4 - 54 kg). Twenty-three (92 %) patients were following cardiac surgery. Patients were receiving invasive or non-invasive mechanical ventilation (31 and 27 courses, respectively), or high-flow nasal cannula oxygen delivered by Vapotherm (27 courses). The main indication for HFCWO was atelectasis detected on a chest x-ray (84 % patients). Other indications included lack of cough reflex, arterial oxygen desaturations, and dyspnea. HFCWO was applied at 7 Hz (range; 5–15 Hz) and a pressure of 2 (range; 1–6 arbitrary units) for 10 minutes. Routine hemodynamic and pulse oximetry data, and qualitative data on patient tolerance were collected before, during, and after HFCWO.

**Results:** Heart rate, systolic and diastolic blood pressure, and respiratory rate increased significantly during HFCWO courses and decreased significantly following therapy. Oxygen saturations significantly decreased during HFCWO and significantly increased after discontinuation of treatment. The differences between pre- and post- HFCWO data were not significant. Patients remained calm during 80 HFCWO courses, and became agitated during 5 courses. No chest tubes, intracardiac lines, or catheters were dislodged in association with HFCWO. No premature discontinuation of therapy was required because of intolerance.

**Conclusions:** The study suggests that HFCWO therapy is safe and well tolerated in children with cardiac diseases in intensive care unit.

### A798 Protein feeding in pediatric acute kidney injury does not delay renal recovery

#### U.G. Kyle^1,2^, A. Akcan-Arikan^1,2,3^, J.C. Silva^1,2^, G. Mackey^1,2^, J. Lusk^1,2^, M. Goldsworthy^1,2^, L.S. Shekerdemian^1,2^, J.A. Coss-Bu^1,2^

##### ^1^Texas Children's Hospital, Intensive Care, Houston, United States; ^2^Baylor College of Medicine, Pediatrics, Houston, United States; ^3^Texas Children's Hospital, Renal Service, Houston, United States

###### **Correspondence:** J.A. Coss-Bu – Texas Children's Hospital, Intensive Care, Houston, United States

**Introduction:** Critically ill children are underfed early in their Pediatric Intensive Care Unit (PICU) stay and this may contribute to worse outcomes. Acute Kidney Injury (AKI) occurs in 10 % of all PICU admissions and the risk of acute and chronic malnutrition is high in these patients with AKI, and the presence of malnutrition in the context of AKI has been associated with more severe clinical deterioration and organ dysfunction. Critically ill children with AKI are at high risk of underfeeding.

**Objectives:** To evaluate the effects of protein feeding on the resolution of AKI.

**Methods:** This is a retrospective study of critically ill children admitted from 10/2012-12/2013 to the PICU. Patients with a diagnosis of end stage renal disease requiring renal replacement therapy or had received a kidney transplant were excluded. Nutritional status assessed by weight and height WHO z-scores after admission and caloric and protein intakes calculated from I.V. fluids and parenteral and enteral nutrition for the first 8 days of admission. Energy and protein needs estimated by Schofield and A.S.P.E.N., respectively. AKI was defined by pRIFLE (creatinine only) and persistent AKI was defined as patients who did not resolve their AKI during the first eight days of PICU stay.

**Results:** A total of 511 patients were included and 156 patients (30.5 %) had AKI. Patients with AKI vs. non-AKI had: age; 1.2 yrs (0.2-6) median (IQR) vs. 1.5 yrs. (0.4-6) (p = 0.10); height: 76 cm (58–110) vs. 81 cm (64–117) (p = 0.02), weight: 9.3 kg (5–21) vs. 11 kg (7–21) (p = 0.04) and mortality: 8.3 % vs. 4.2 % respectively. Forty-four (8.61 %) and 112 (21.9 %) patients had persistent and resolved AKI, respectively, and persistent AKI patients were more likely to have moderate/severe chronic malnutrition vs. non-AKI patients, odds ratio (95 % CI) 2.4 (1.2-4.6) (p = 0.014). Patients with no AKI, resolved AKI, and persistent AKI received in the first 8 days of PICU stay 73 %, 80 % and 80 % of recommended energy needs, and 39 %, 42 %, and 51 % of protein needs, respectively. Compared to 12 % of no-AKI patients, 17 % and 27 % of patients with resolved and persistent AKI, met ≥80 % of protein needs respectively, (p = 0.01) Although patients with persistent AKI received higher protein intake of ≥80 % of goal, was not independently associated with persistent AKI after adjustment for PRISM score (p = 0.13).

**Conclusions:** Protein prescription is improved in children with AKI in our PICU, largely due to ongoing educational efforts. Higher protein intake was not associated with a delay in renal recovery in patients with AKI after adjustment for severity of illness.

**References:**

1. Akcan-Arikan A, Kidney Int. 2007.

2. Schofield WN. Clin Nutr 1985.

3. Mehta NM. JPEN, 2009.

**Grant acknowledgement**

Internal Funding, Texas Children's Hospital

### A799 Current intensive care management for adolescents in the United Kingdom: a retrospective cohort study

#### D. Wood^1^, D. Harrison^2^, R. Parslow^3^, P. Davis^1^, J. Pappachan^4^, S. Goodwin^1^, P. Ramnarayan^5^

##### ^1^Bristol Royal Hospital for Children, Paediatric Intensive Care Unit, Bristol, United Kingdom; ^2^Intensive Care National Audit & Research Centre (ICNARC), London, United Kingdom; ^3^University of Leeds, Leeds, United Kingdom; ^4^Southampton University Hospitals NHS Trust, Southampton, United Kingdom; ^5^Great Ormond Street Hospital, London, United Kingdom

###### **Correspondence:** D. Wood – Bristol Royal Hospital for Children, Paediatric Intensive Care Unit, Bristol, United Kingdom

**Introduction:** The transition between childhood and adulthood is a time of rapid physical, psychological and behavioural change. Adolescents (aged 12–19 years) requiring intensive care differ from both the typical paediatric intensive care (PICU) population, mainly infants and pre-school children, and from the typical adult intensive care unit (AICU) population of much older adults. Critically ill adolescents are distinct from the majority of patients treated in either adult or paediatric intensive care units (ICU). Little data exist to describe how best to meet their needs or those of their families.

**Objectives:** We describe the case mix, resource use, and outcomes for adolescents admitted to AICU and PICU in the UK.

**Methods:** Analysis of national, prospectively collected data for adolescents (aged 12–19 years) admitted to ICUs in the UK between 2007 and 2014.

**Results:** 37320 adolescents were admitted during the study period. Excluding admissions following elective surgery, there were a total of 27442 admissions; in this group ICU mortality was 6.0 % and 5.4 % for those admitted to PICU and AICU respectively, a non-significant difference. The most common diagnostic categories for adolescents in AICU were trauma and drug/alcohol-related; those admitted to PICU most commonly had respiratory diagnoses.

**Conclusions:** ICU mortality was higher for those admitted to PICU than AICU, but this may represent a greater severity of acute illness or underlying burden of chronic illness for adolescents cared for in a PICU. There is increasing recognition that adolescents need special consideration when planning preventative health-care and access to health services. Identifying the appropriate setting for the provision of intensive care for this population may be equally important.

### A800 Comparison of postoperative sedation and analgesia of neonates in cardiac surgery: dexmedetomidine vs standard regimen

#### S. Chernyshuk, H. Yemets, V. Zhovnir

##### UCCC, ICU, Kyiv, Ukraine

###### **Correspondence:** H. Yemets – UCCC, ICU, Kyiv, Ukraine

**Introduction:** Sedation and analgesia are important components of postoperative management of neonates who underwent cardiac surgery. Excessive or inadequate sedation may have a significant adverse effect on patient outcome.

Objectives. We aimed to determine which drug regimen would be most effective with less side-effect and better outcome.

**Methods:** From March 2012 till March 2014 we conducted a randomized controlled prospective study in 60 neonates with congenital heart disease who underwent Arterial Switch Operation in our clinic. Inclusion criteria: 1) gestational age more than 36 weeks, 2) birth weight over 2500 g, 3) age - up to 28 days, 4) absence of concomitant diseases and surgical complications.

Patients were randomized into 2 cohorts: 30 patients (50 %) were given infusion of dexmedetomidine with morphine boluses (study group) and 30 patients (50 %) were randomized to the standard regimen - infusion of morphine with diazepam boluses (control group).

**Results:** In both groups there were no differences in pre- and intraoperative indexes, duration of mechanical ventilation, sympathomimetic support, and time of infusion of dexmedetomidine/morphine. In study group time of ICU stay - 93.5 h - was significantly shorter than in control group -120 h (p-0.02). Onset of peristalsis and start of feeding in study group was earlier than in control group - 1-st vs 2-d day (p- 0.007) and 2-d vs 2.5-day (p-0.035), respectively. In the control group there were more patients who had complicated feeding (start after 3-d day, bloating or vomiting) - 11(37 %) vs 3(10 %) in the study group. We did not observe any decrease of mean blood pressure and heart rate in the study group as it could be expected.

**Conclusion:** Use of dexmedetomidine with morphine hydrochloride boluses for postoperative sedation and analgesia is effective and facilitates feeding process in neonates, leads to earlier onset of peristalsis and start of feeding, decreasing ICU stay.

### A801 Impact of positive end expiratory pressure on cerebral hemodynamic in paediatric patients with post-traumatic brain swelling treated by surgical decompression

#### S.M. Pulitano’^1^, S. De Rosa^1,2^, A. Mancino^1^, G. Villa^2,3^, F. Tosi^1^, P. Franchi^1^, G. Conti^1^

##### ^1^Catholic University, Department of Anesthesia and Intensive Care, Rome, Italy; ^2^International Renal Research Institute of Vicenza (IRRIV), Department of Nephrology, Dialysis and Transplantation, Vicenza, Italy; ^3^University of Florence, Department of Health Science, Section of Anaesthesiology and Intensive Care, Florence, Italy

###### **Correspondence:** P. Franchi – Catholic University, Department of Anesthesia and Intensive Care, Rome, Italy

**Introduction:** Current Brain Trauma recommendations are based to early correction of hypoxemia and avoidance of hypocarbia after severe paediatric TBI. The use of positive end-expiratory pressure (PEEP) in this situation remains controversial. Positive end expiratory pressure (PEEP) may reduce ventilator-induced lung injury by avoiding cyclic recruitment/derecruitment and prevent lung collapse. The aim of this investigation is to evaluate the impact of different PEEP levels on cerebral hemodynamic, gas exchange and respiratory system mechanics in paediatric patients with a severe post-traumatic brain swelling treated with decompressive craniectomy (DC).

**Objectives:** The aim of this investigation is to evaluate the impact of different PEEP levels on cerebral hemodynamic, gas exchange and respiratory system mechanics in paediatric patients with a severe post-traumatic brain swelling treated with decompressive craniectomy (DC).

**Methods:** A prospective physiologic study was carried out on 14 paediatric patients presenting with severe traumatic brain swelling and treated with DC. Intracranial pressure (ICP), and cerebral perfusion pressure (CPP), central venous pressure (CVP), arterial oxygen saturation and the middle cerebral artery mean velocity (Vmed) was determined. After assessment at 0 PEEP (ZEEP), PEEP 4 and PEEP 8 were applied: all parameters were recorded at each level.

**Results:** The application of PEEP (from ZEEP to PEEP 8) significantly increased compliance of the respiratory system indexed to the weight of the patients (CrsI) (P = 0.02) without ICP modifications. No significant variations were observed in values of arterial pressure (MAP), CPP, Vmed, total resistance of the respiratory system indexed to the weight of the patients (RRSmaxI), and ohmic resistance of the respiratory system indexed to the weight of the patients (RRSminI). CVP significantly increased between ZEEP and PEEP 8 (P = 0.005), and between PEEP 4 and PEEP 8 (P = 0.05).

**Conclusions:** We describe cerebral hemodynamic responses to PEEP application in pediatrics. PEEP values up to 8 cm H2O seem to be safe in paediatric patients with a severe post-traumatic brain swelling treated with DC.

**References:**

1. Bein T, Kuhr LP, Bele S, Ploner F, Keyl C, Taeger K. Lung recruitment maneuver in patients with cerebral injury: effects on intracranial pressure and cerebral metabolism. Intensive Care Med 2002;28:554–8

2. Bor-Seng-Shu E, Hirsch R, Teixeira MJ, De Andrade AF, Marino R Jr (2006) Cerebral hemodynamic changes gauged by transcranial Dopp- ler ultrasonography in patients with posttraumatic brain swelling treated by surgical decompression. J Neurosurg 104:93–100

### A802 Randomized clinical trial of high concentration oxygen versus titrated oxygen therapy in pediatric asthma exacerbation

#### B. Patel^1^, H. Khine^2^, A. Shah^2^, D. Sung^2^, L. Singer^2^

##### ^1^The Children's Hospital at Montefiore, Pediatric Critical Care, Bronx, United States; ^2^The Children's Hospital at Montefiore, Bronx, United States

###### **Correspondence:** B. Patel – The Children's Hospital at Montefiore, Pediatric Critical Care, Bronx, United States

**Introduction:** Asthma exacerbation is one of the most common diagnoses seen in the pediatric ED. Several adult randomized controlled trials have shown that administration of high concentration oxygen leads to rise in carbon dioxide and increases admission rates. However, there are no studies in the pediatric population comparing the effects of high concentration oxygen versus titrated oxygen therapy in asthma exacerbation.

**Objectives:** We evaluated the effects of transcutaneous carbon dioxide (tPaCO_2_) in high concentration oxygen therapy versus titrated oxygen therapy to maintain saturation between 92 to 95 % in pediatric patients with acute asthma exacerbation in the ED.

**Methods:** Children 2 to 18 years with previously diagnosed asthma with moderate to severe asthma exacerbation (asthma score > 5) were randomized to high concentration oxygen therapy (100 % oxygen via face mask at >4 L/min.) or titrated oxygen therapy (titrated up from 21 % via a blender continuously) to maintain saturations between 92 to 95 % while receiving their nebulized treatments. Exclusion criteria included disorders with hypercapnic respiratory failure, unconscious patient, history of congenital heart disease, pregnancy, history of smoking or using sedatives and depressants. Asthma therapy was provided per the ED physician. Asthma score, tPaCO_2_, PEFR (age >7 years) were measured at the start of the study and every 20 minutes for the first hour then every 30 minutes until disposition decision. The primary outcome was increase in tPaCO_2_ with high concentration oxygen therapy. Secondary outcome included rate of admission to the hospital.

**Results:** 73 patients were enrolled with mean age of 8.6 years. 60 % were males and 72 % had poorly controlled asthma with mean asthma score of 7.6. There were 36 patients enrolled in the high concentration oxygen group (HCOT) and 37 patients in the titrated oxygen group (TOT). The 0 minute tPaCO_2_ were not statistically different(35.6 ± 3.8 HCOT v. 37.4 ± 4.4 TOT,p = 0.07); whereas, the 20 minutes tPaCO_2_ was statistically different(40 ± 3.8 HCOT v. 37.5 ± 5.1 TOT, p = 0.02). The 60 minutes tPaCO_2_ was 39.2 ± 4.6 HCOT v. 35.5 ± 4.3 TOT, p = 0.0009. At 20 minutes, 89 % of the patients had a rise in tPaCO_2_ in HCOT v. 30 % in the TOT(p = < 0.0001), and at 60 minutes 78 % had a rise in tPaCO2 in HCOT v. 16 % in the TOT(p = < 0.0001). The asthma score was similar in the two groups at 0 minute (7.8 ± 1.4 HCOT v. 7.4 ± 1.3 TOT, p = 0.23); whereas, the 60 minutes asthma score was lower in the TOT(4.7 ± 1.5 HCOT v. 3.7 ± 1.3 TOT, p = 0.002). The rate of admission to the hospital was 36.1 % in HCOT v. 24.3 % in the TOT.

Conclusions: High concentration oxygen therapy in pediatric asthma exacerbation leads to significantly higher carbon dioxide levels. It also causes rise in carbon dioxide from the baseline which increases the asthma scores and rate of admission.

**References:**

1. Chien J.Uncontrolled oxygen administration and respiratory failure in acute asthma.Chest 2000;117(3):728–733.

### A803 Treatment of refractory status epilepticus with thiopental versus propofol in children: a randomized trial

#### S. Haghbin^1^, S. Inaloo^2^, Z. Serati^2^

##### ^1^Shiraz University of Medical Sciences, Pediatric intensive Care, Shiraz, Islamic Republic of Iran; ^2^Shiraz University of Medical Sciences, Shiraz, Islamic Republic of Iran

###### **Correspondence:** S. Haghbin – Shiraz University of Medical Sciences, Pediatric intensive Care, Shiraz, Islamic Republic of Iran

**Introduction:** Refractory status epilepticus (RSE) is a life-threatening condition in which seizures do not respond to first- and second-line anticonvulsant drug therapies and is associated with increased hospital length of stay, mortality and functional disability (1). Coma induction is advocated for its management by different agents (2).

**Objectives:** We aimed to assess the effectiveness (RSE control, adverse events) of propofol versus thiopental infusion in the treatment of RSE.

**Methods:** In this randomized, single blind studying children aged 2 months- 18 years with RSE not due to cerebral ischemia were included. Medications were increased toward the EEG burst-suppression or to maximum limit of medication, and then progressively weaned. The primary endpoint was the proportion of patients with RSE controlled after a first course of study medication; secondary endpoints included clinical outcomes measures.

**Results:** In this study, 40 patients were included, 18 received propofol and 22 thiopental. RSE was generalized in 32 patients and focal in 8.The primary endpoint was reached in 72 % with propofol versus 54 % with thiopental (P = 0.33). However, mean duration of treatment with propofol was 50 hrs (range 12–94), and with thiopental was 10 days. While mortality (44 % vs23% P = 0.18), infection and systemic hypotension were similar in both groups, thiopental use was associated with longer mechanical ventilator (P = 0.02). More patients returned to basic condition at discharge with propofol (P = 0.04). Treatment failure was seen in 7/8 patients with focal convulsion. Two patients died due to propofol infusion syndrome with dose of 8 and 7 mg/kg/hr, so the maximum dose of propofol decreased to 6 afterward. Five patients died due to complications of thiopental infusion.

**Conclusions:** Although this study showed no significant difference between two groups regarding effectiveness, adverse effects and mortality, patients on propofol obtained quicker convulsion control and better return to baseline condition. A previous study did not disclose any difference between these two agents (3). However, care must be taken when it is used in longer than 24 hours with higher dose than 6 mg/kg/hr .

Trial Registration: IRCT.IR IRCT138707231349N1

**References**

1. Abend NS, Dlugos DJ. Treatment of refractory status epilepticus: literature review and a proposed protocol. Pediatr Neurol. 2008,38(6): 37790.

2. Brophy GM, Bell R, Claassen J, et al. Guidelines for the evaluation and management of status epilepticus. Neurocrit Care 2012; 17:3.

3. Rossetti AO, Milligan TA, Vulliémoz S, Michaelides C, Bertschi M, Lee JW. A randomized trial for the treatment of refractory status epilepticus. Neurocrit Care. 2011 Feb;14(1):4–10.

### A804 An evaluation and accuracy of new zero-heat-flux thermometer (3 M SpotOn) in pediatric intensive care patients

#### M. Idei^1^, T. Nomura^2^, N. Yamamoto^1^, Y. Sakai^1^, T. Yoshida^1^, Y. Matsuda^1^, Y. Yamaguchi^1^, S. Takaki^1^, O. Yamaguchi^1^, T. Goto^2^

##### ^1^Yokohama City University Hospital, Intensive Care Unit, Yokohama, Japan; ^2^Yokohama City University Hospital, Department of Anesthesiology, Yokohama, Japan

###### **Correspondence:** M. Idei – Yokohama City University Hospital, Intensive Care Unit, Yokohama, Japan

**Introduction:** In critically ill patients, temperature measurement is a routine important care task and can lead to important decisions. Rectal temperature and bladder temperature are now used as a continuous body temperature measuring method in the pediatric intensive care, but these practices have several disadvantages including the patient´s discomfort, the risk of organ injury and the inaccurate measuring caused by the sensor position. A new temperature monitoring system 3M^TM^ SpotOn^TM^ (SpotOn) is a non-invasive zero-heat-flux thermometer designed to estimate core body temperature from the skin surface. Although the usefulness and accuracy of SpotOn system in adult patients have been demonstrated, there are no reports on pediatric intensive care patients.

**Objectives:** The aim of this study was to evaluate the effectiveness of a new temperature measurement system attached to the forehead, and compare it to rectal temperature sensors in terms of correlation and accuracy.

**Methods:** Pediatric patients weighing less than 10Kg, who were managed in our ICU during the period from February 2015 to March 2016, were enrolled in this study. Core temperature was measured and recorded at every minute from the both thermistor of a rectal thermal probe and with SpotOn in these patients. The data when the forehead sensor or rectal probe was taken out for nursing care was excluded from statistical analysis.

**Results:** 53495 sets of data of 26 children (Mean BW 5630 g) were examined retrospectively. In all patients, SpotOn showed higher than the rectal temperatures. The SpotOn temperature was analyzed to be 0.82 degrees (95 % limits of agreement of ± 0.51) higher temperature than the rectal one with a moderate correlation(r = 0.73).

**Discussion and conclusion:** Rectal temperature measurement is the gold standard method for pediatric patients in ICU despite several complications of rectal injury. Our children´s study demonstrated the slightly higher temperature in the SpotOn than rectal temperature with a substantial correlation. One possible explanation could be that the abundance of brain blood flow of children affected the results. Our study concluded that SpotOn system could be used as a highly reliable noninvasive core body temperature measurement for small pediatric patients.

**References**

1. Eshraghi Y, Nasr V, Parra-Sanchez I et al. An evaluation of a zero-heat-flux cutaneous thermometer in cardiac surgical patients. Anesth Analg. 2014 Sep;119(3):543–9.

2. Hebbar K, Fortenberry JD, Rogers K, et al. Comparison of temporal artery thermometer to standard temperature measurements in pediatric intensive care unit patients. Pediatr Crit Care Med. 2005; 6: 557–561.

**Grant acknowledgement**

None

### A805 Viral bronchiolitis in pediatric acute respiratory distress syndrome

#### N. Longani, S. Medar

##### The Children's Hospital at Montefiore, Pediatric Critical Care, Bronx, United States

###### **Correspondence:** N. Longani – The Children's Hospital at Montefiore, Pediatric Critical Care, Bronx, United States

**Introduction:** Viral bronchiolitis (VB) remains one of the leading causes of hospitalization in early childhood. Despite the heavy burden of VB on the healthcare system, little is known about the incidence of Acute Respiratory Distress Syndrome (ARDS) in this cohort of patients. In 2015, the Pediatric Acute Lung Injury Consensus Conference (PALICC) published guidelines for the definition, management and research in pediatric ARDS (PARDS) (1).

**Objectives:** To study the incidence and prevalence of PARDS in VB and to study the association between PARDS and specific PICU outcomes such as incidence of mechanical ventilation, noninvasive ventilator settings length of PICU stay in this group of patients.

**Methods:** This is a retrospective single center observational cohort study that examined children 0–2 years of age admitted to the PICU with VB and respiratory failure (RF) from 2011–2014. PALICC criteria were applied to define PARDS. Clinical and demographic data was collected. Patients with a diagnosis of congenital heart disease or pre-existing chronic lung disease were excluded. Data was expressed as median with IQR ranges. Test of bivariate association were performed using Mann Whitney U test and chi square test. A two tailed p value of ≤ 0.05 was used to denote statistical significance.

**Results:** Out of 1700 patients with RF, 330 with VB met study criteria. Eighty of these 330 (24 %) patients admitted for VB met the criteria for PARDS or at risk for PARDS. Out of these 80 patients, 25 (31 %) met criteria for PARDS and 55 (69 %) met criteria for “at risk of PARDS”. Median age was 5 (2,11) months and the median weight was 6.9 (5.3, 9.5) kgs. Most common etiology for VB was respiratory syncytial virus (RSV) 68 % followed by Rhinovirus (20 %). There was no statistically significant difference in age, weight, and etiology of VB in patients with PARDS and those “at risk of PARDS.” Patients with PARDS had longer hospital and PICU length of stay (LOS) and more likely to receive diuretics compared to those “at risk for PARDS” (16 (10, 21) Vs 8 (6, 10.5), p = 0.0001; 10 (7, 13) Vs 3 (2, 4.5), p < 0.0001; and 66 % vs 33 %, p = 0.02 respectively). Nineteen (19/25, 76 %) patients with PARDS received invasive mechanical ventilation with a median duration of ventilation of 6 (1, 10) days.

**Conclusions:** Almost a quarter of children with VB developed PARDS or were at risk of PARDS. The presence of PARDS in children with VB was significantly associated with longer PICU and Hospital LOS compared to those “at risk of PARDS”. Children with VB are a high risk group for the development of PARDS.

**References**

1) Pediatric Acute Lung Injury Consensus Conference Group, et al.Pediatric acute respiratory distress syndrome:consensus recommendations from the Pediatric Acute Lung Injury Consensus Conference. Pediatric Crit Care Med 2015 Jun; 5: 428–439

2) Zorc J, Hall C. Bronchiolitis: recent evidence on diagnosis and management. Pediatrics 2010; 25:342–349

### A806 Mean platelet volume dynamics and platelet count as prognostic indicators in pediatric surgical intensive care: a descriptive observational study

#### I.R. Abdel-Aal^1^, A.S. El Adawy^2^, H.M.E.-H. Mohammed^2^, A.N. Mohamed^2^

##### ^1^Cairo University/Kasr Alainy Medical School, Anesthesia, Pain and Surgical ICU, Cairo, Egypt; ^2^Cairo University/Kasr Alainy Medical School, Cairo, Egypt

###### **Correspondence:** I.R. Abdel-Aal – Cairo University/Kasr Alainy Medical School, Anesthesia, Pain and Surgical ICU, Cairo, Egypt

**Introduction:** Mean platelet volume(MPV) seems to be a marker of platelet activation and may be related to severity of illness.^1^Changes in MPV and platelet count(PLC)could be used for disease prognosis and mortality in ICU patients.^2^We hypothesized that MPV changes and PLC could be used as prognostic tools in pediatric surgical intensive care units(PSICU).

**Objectives:** To study the association between MPV changes and mortality and morbidity in PSICU. Also to study the relation between PLC and PSICU mortality and morbidity.

**Methods:** This descriptive observational study was conducted on consecutive 100 pediatric surgical patients who admitted to PSICUs at Cairo University Hospitals starting from 1/6-1/12/2015.After approval by research ethics committee,informed consents were obtained from parents and pediatric cases aged from 1 month-18 years and stayed for > 48 h were enrolled.MPV and PLC were obtained and recorded at baseline(pre-operative values),on the day of ICU admission(day 0),1^st^,2^nd^,3^rd^,5^th^ and 7^th^ days.To measure daily MPV changes; (ΔMPV) was constructed and computed where ΔMPV = ([MPVday(X) − MPVday (0)]/MPVday(0) × 100 %. Pediatric Index of Mortality(PIM)score was calculated on day 0 and the Pediatric Logistic Organ Dysfunction(PELOD)Score was recorded daily.

**Fig. 2 (abstract A806).**
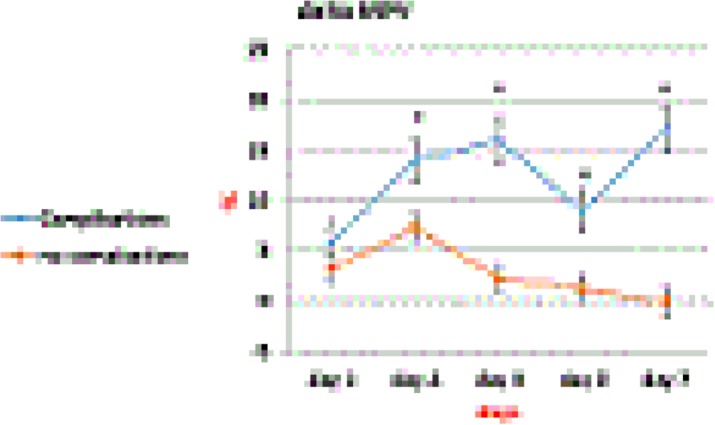
Percentage changes in MPV (Delta MPV) among ICU co.

**Fig. 3 (abstract A806).**
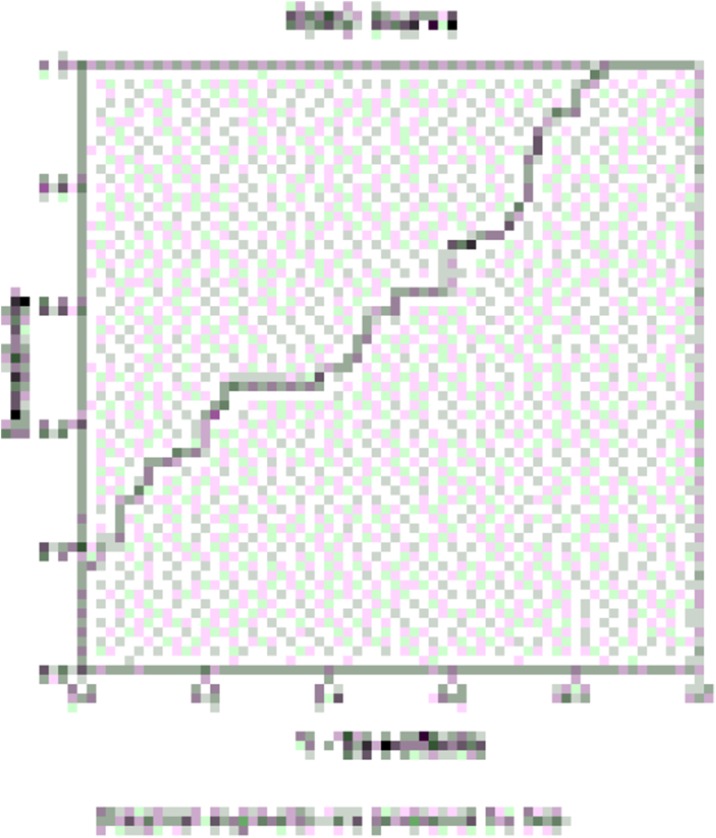
Receiver operating characteristics (ROC) curve for

**Results:** Patients who developed ICU complications (fever, sepsis, pneumonia, required mechanical ventilation, needed vasopressors or blood transfusion); showed higher ΔMPV compared to non complicated cases (Fig. 2). This association was statistically significant on days 2 (**p value = 0.035)**,3(**p value < 0.001)**, 5 (**p value < 0.001)** and 7(**p value = 0.017)** of ICU stay but it´s insignificant on day1(**p value =*0.691)***.According to receiver operating characteristics(ROC) curve analysis, the sensitivity of ΔMPV to detect complications on day 2 was 57.2 % but its specificity on day 2 was 76.6 %.Patients who developed ICU complications showed lower PLC compared to non complicated cases(Fig. 3).This association was statistically significant on days1(**p value** < 0.001**),2(p value <** ***0.001*) and** 3(**p value <** ***0.001*)** but it was insignificant on day 0(**p value =*0.237*** ),5(**p value =*0.861) and 7***(**p value =*0.247)***. On other hand, the sensitivity of PLC to detect complications day1 was **81.4 %** but the specificity was **71.9** %, while the sensitivity of PLC to detect complications day 2 was **81.1 %** but the specificity day 2 was **100** %.

**Conclusions:** MPV dynamics and PLC have prognostic roles and could be used in determining several complications in critically ill pediatric surgical patients. PLC is a more specific and sensitive tool to detect complications than mean MPV dynamics.

**References**

1- Cekmez F et al. Mean platelet volume in very preterm infants: a predictor of morbidities.Eur Rev Med Pharmacol Sci. 2013; 17: 134–137.

2- Cengizhan S, et al. Alterations in platelet count and mean platelet volume as predictors of patient outcome in the respiratory intensive care unit. Clin Respir J. 2014;5:35–40.

**Fig. 4 (abstract A806).**
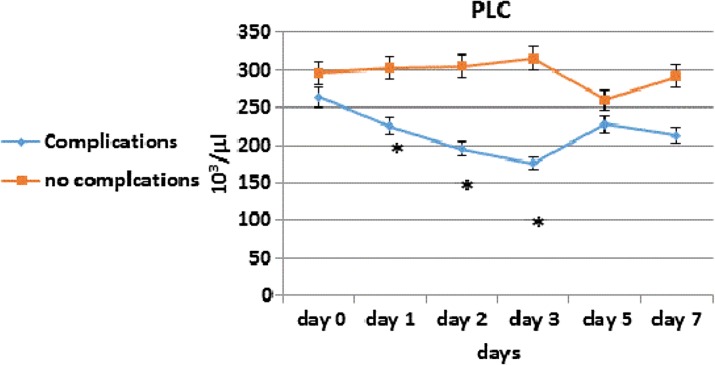
Platelets count (PLC) among intensive care unit.

## REHABILITATION & RECOVERY FROM ICU

### A807 Physical function in critical care (Pacific): a multi-centre observational study

#### S.M. Parry^1^, L.D. Knight^2^, L. Denehy^1^, N. De Morton^3^, C.E. Baldwin^4,5^, D. Sani^6^, G. Kayambu^6^, V.Z.M. da Silva^7,8^, P. Phongpagdi^1^, Z.A. Puthucheary^9,10,11^, C.L. Granger^1,2^

##### ^1^The University of Melbourne, Department of Physiotherapy, Melbourne, Australia; ^2^Royal Melbourne Hospital, Department of Physiotherapy, Melbourne, Australia; ^3^Peter MacCallum Cancer Centre, Department of Physiotherapy, Melbourne, Australia; ^4^Flinders Medical Centre, Department of Physiotherapy, Adelaide, Australia; ^5^University of South Australia, Member of the International Centre for Allied Health Evidence (iCAHE) and the Sansom Institute, Adelaide, Australia; ^6^National University Hospital, Department of Rehabilitation, Singapore, Singapore; ^7^Escola Superior da Saude, Health Sciences Program, Brasilia, Brazil; ^8^Hospital de Base do Distrito Federal, Brasilia, Brazil; ^9^University College London Hospitals, Division of Critical Care, London, United Kingdom; ^10^University College London Hospitals, Institute of Sports and Exercise Health, London, United Kingdom; ^11^National University Hospital, Division of Respiratory and Critical Care, Singapore, Singapore

###### **Correspondence:** S.M. Parry – The University of Melbourne, Department of Physiotherapy, Melbourne, Australia

**Introduction:** Impairment in physical function is a significant problem for survivors of critical illness [1,2]. There is growing urgency to develop a core set of outcome measures which can be adopted in clinical and research practice to evaluate efficacy in response to interventions such as rehabilitation. There is currently not a single outcome measure which can be used across the continuum from ICU admission to hospital discharge for individuals with critical illness [3].

**Objectives:** (1) To determine the clinical utility of two physical function measures: De Morton Mobility Index (DEMMI) and Physical Function in Intensive Care test-scored (PFIT-s) when used in isolation across the hospital admission; and (2) To transform the (15-item) DEMMI and (4-item) PFIT-s into a single measure to evaluate function in ICU survivors using rasch analytical principles.

**Methods:** Multi-centre prospective observational study conducted across four sites internationally. Consecutive eligible participants were recruited who met inclusion criteria; ; Adults > 18 years of age whom were mechanically ventilated > 48 hours and were ambulant at least 10 metres independently prior to their ICU admission. Physical function was evaluated at ICU awakening, and both ICU and hospital discharge using the PFIT-s and DEMMI, administered in a randomised sequence using concealed allocation on each measurement occasion to minimise bias in testing order.

**Results:** 128 participants have been recruited into the study to date across the four sites. 61 % were male (n = 78) with median age of 65 [53–73]; and moderate severity of illness (median [IQR] APACHE II: 22 [17–27]). Median [IQR] ICU and hospital LOS were 9 [5–14] and 21 [13–37] days respectively. The incidence of ICU-acquired weakness was 50 % (n = 67). Aim 1: On awakening mean ± SD PFIT-s was 4.9 ± 2.5 (out of 10) and DEMMI was 19 ± 21 (out of 100). In isolation the PFIT-s had a floor effect of 9 % (n = 11) at ICU awakening, and 1 % (n = 1) at both ICU and hospital discharge; and a large ceiling effect at hospital discharge of 42 % (n = 40). The DEMMI in isolation had a large floor effect in the ICU of 23 % at awakening, and a small ceiling effect at hospital discharge of 14 % (n = 14). Both the PFIT-s and DEMMI were demonstrated to be highly responsive to change in functional recovery over the acute hospitalisation period (p < 0.005). Aim 2: Preliminary exploration of a subgroup with complete data at hospital discharge (n = 73) was evaluated.The data fit the Rasch model Chi squared =10.4, df = 24, p = 0.99 with no item misfit or differential item functioning based on age, gender, BMI, severity of illness (APACHE II) or comorbidity. A new single measure (12-items) has been proposed combining the DEMMI and PFIT-s.

**Conclusions:** The PFIT-s and DEMMI have limitations when used in isolation. A new transformed scale based on rasch analytical principles is promising combining features of both tools for evaluation of functional recovery of critically ill.

### A808 Functional status at ICU admission, physical therapy treatment and critical care outcomes

#### J.E. Rydingsward^1^, C.M. Horkan^2^, K.B. Christopher^3,4^

##### ^1^Brigham and Women's Hospital, Department of Rehabilitation, Boston, United States; ^2^Brigham and Women's Hospital, Department of Medicine, Boston, United States; ^3^Brigham and Women's Hospital, Renal Division, Boston, United States; ^4^Brigham and Women's Hospital, Channing Division of Network Medicine, Boston, United States

###### **Correspondence:** J.E. Rydingsward – Brigham and Women's Hospital, Department of Rehabilitation, Boston, United States

**Introduction:** Limited information exists regarding the association between functional status at ICU admission at and outcomes.

**Objectives:** We hypothesized that initial functional status assessment as well the amount of physical therapy delivered would be associated with outcomes in ICU survivors.

**Methods:** We performed a retrospective cohort study in one Boston teaching hospital on 2,828 adults who received critical care from 1997 to 2011 and survived hospitalization. All patients had a formal evaluation by a physical therapist in the week prior to ICU admission and at hospital discharge. The exposure of interest was functional status determined by a licensed physical therapist based on the functional mobility sub scales of the Functional Independence Measure. All patients received physical therapy to improve functional performance. The primary outcome was 90-day all-cause mortality. We used logistic regression to describe how 90-day mortality differed with functional status at ICU admission. Negative binomial regression was utilized to describe how functional status at hospital discharge differed with functional status at ICU admission, the extent of physical therapy received and hospital length of stay.

**Results:** The cohort was 52 % male, 22 % non-white and had a mean age of 64.1 years. 10 % of the cohort had sepsis, 7 % had acute kidney injury, 32 % had respiratory failure and 53 % were surgical cases. The median [IQR] hospital length of stay was 8 [4, 14] days. The 90-day mortality rate was 14.6 %. Functional status at ICU admission was robustly associated with 90-day mortality. In a logistic regression model adjusted for age, gender, race, surgical patient type, Deyo-Charlson index, acute organ failure, sepsis, length of stay and the extent of physical therapy received, the second lowest and lowest quartiles of functional status at ICU admission was associated with a 1.8 and 2.3 fold increased odds of 90-day mortality respectively, compared to patients with the highest quartile of functional status [OR = 1.80(95%CI 1.26-2.57) and OR = 2.34(95%CI 1.63-3.36)]. Every 15 minute increment in physical therapy completed was associated with a decrease in the adjusted odds of 90-day mortality [OR = 0.60 (95%CI 0.53-0.68)]. Further, in survivors of hospitalization (n = 2,364), patients with the second lowest and lowest quartiles of functional status at ICU admission had a 2.7 and 3.4-fold lower functional status assessed at hospital discharge following adjustment, compared to patients with the highest quartile of functional status [IRR 2.74 (95%CI 2.50-3.01) and IRR 3.42 (95%CI 3.10-3.77)] respectively.

**Conclusions:** In critically ill patients, decreased functional status at ICU admission is associated with increased 90-day mortality. Increased intensity of physical therapy is associated with improved mortality outcomes. Both functional status at ICU admission and the intensity of physical therapy contribute to functional status determined at hospital discharge.

### A809 Does enhanced physiotherapy and early mobilisation reduce the degree of muscle loss for patients admitted to critical care?

#### D. McWilliams, C. Jones, E. Reeves, G. Atkins, C. Snelson

##### Queen Elizabeth Hospital NHS FT, Birmingham, United Kingdom

###### **Correspondence:** D. McWilliams – Queen Elizabeth Hospital NHS FT, Birmingham, United Kingdom

**Introduction:** Patients admitted to critical care are shown to lose significant muscle mass, with the degree of muscle loss as high as 20 % in the first week for those in multi organ failure (Puthucheary, 2013). Early rehabilitation has been demonstrated as a safe and effective method of improving functional status at the point of critical care discharge and reducing both ICU and hospital length of stay (McWilliams et al., 2015), although the specific impact of this on muscle mass has not been reported.

**Objectives:** This study aimed to analyse the impact of enhanced physiotherapy incorporating early mobilisation on the rate of muscle decline for patients admitted to critical care.

**Methods:** Patients admitted to a large UK teaching hospital during the trial period and ventilated for ≥ 5 days were included in the study. Patients were randomised to either enhanced physiotherapy or standard care groups as part of a larger RCT. Baseline measurements were taken on the day of recruitment and then repeated at critical care discharge. Muscle mass was measured using ultrasound to calculate cross sectional area of quadriceps and biceps. To ensure validity , 2 measures were taken and the average of these taken as the final value. All scans were reviewed for agreement by 2 therapists trained in muscle ultrasound.

**Results:** 40 patients were included in the analysis. Patients in the enhanced physiotherapy group mobilised earlier and achieved a higher level of mobility at the point of critical care discharge (see Table 2.) All subjects demonstrated a reduction in muscle mass of both quadriceps and biceps over the course of their critical care stay. The extent of muscle loss was however lower in those receiving the enhanced physiotherapy, although this did not reach statistical significance (Quads 3 % vs 13 %. p = 0.14317; Biceps 6 % vs 13 %, p = 0.12033).

**Table 2 (abstract A809).** Demongraphics

	Enhanced (n = 20)	Control (n = 20)
Median Age (years)	62.5	60
Sex (male)	14 (70 %)	10 (50 %)
Median Time to Mobilise	8 days	9.5 days
Median MMS at critical care discharge	7	5.5
Median Critical care length of stay	16.5 days	18 days

**Table 3 (abstract A809).** USS measurements

	Baseline	Critical care discharge	Diff
Control Quads	2.65	2.30	−0.35 (13 %)
Enhanced Quads	2.55	2.48	−0.07 (3 %)
Control Biceps	2.66	2.32	−0.34 (13 %)
Enhanced Biceps	2.71	2.55	−0.16 (6 %)

**Conclusions:** A programme of enhanced physiotherapy appeared to be associated with a lower rate of muscle loss in both biceps and quadriceps in comparison to standard care. An appropriately powered RCT is required to assess these findings.

**References**

1. Puthucheary ZA, Rawal J, McPhail M et al. (2013) Acute Skeletal Muscle Wasting in Critical Illness. JAMA. 2013;310(15):1591–1600

2. McWilliams D, Weblin J, Atkins G et al. (2014) Enhancing rehabilitation of mechanically ventilated patients in the intensive care unit: A quality improvement project. Journal of critical care. http://dx.doi.org/10.1016/j.jcrc.2014.09.18

### A810 Similarities and differences in patients' and relatives' views of information provision after ICU

#### L.M. Aitken^1,2,3^, J. Rattray^4^, J. Kenardy^5^, A.M. Hull^6^, A. Ullman^2^, R. Le Brocque^5^, M. Mitchell^2,3^, C. Davis^3^, B. Macfarlane^2,3^

##### ^1^City University London, School of Health Sciences, London, United Kingdom; ^2^Griffith University, NHMRC Centre of Research Excellence in Nursing, Menzies Health Institute Queensland, Brisbane, Australia; ^3^Princess Alexandra Hospital, Intensive Care Unit, Brisbane, Australia; ^4^University of Dundee, Dundee, United Kingdom; ^5^University of Queensland, Brisbane, Australia, ^6^NHS Tayside, Dundee, United Kingdom

###### Correspondence: L.M. Aitken – City University London, School of Health Sciences, London, United Kingdom

**Introduction:** Survivors of critical illness experience a range of impairments after intensive care, including physical, cognitive and psychological compromise. The provision of information using a diary to describe the intensive care unit (ICU) experience is one strategy that has been proposed to improve psychological health.

**Objectives:** The purpose of this study was to explore similarities and differences in patients' and relatives' perceptions of information containing strategies, including ICU diaries, to assist recovery after critical illness.

**Methods:** An exploratory mixed-methods study was undertaken in an Australian tertiary hospital with general ICU patients admitted for ≥3 days and their relatives. Semi-structured interviews were conducted 3–5 months after ICU discharge. Transcripts were analysed using content analysis.

**Results:** Twenty-two patients and 19 relatives consented to participation and completed interviews prior to reaching data saturation. Patients were usually male (63 %) and aged 52 ± 14 years. Patients raised similar themes to relatives, although with diverse opinions. Themes of wanting to have a diary kept and considering they would find a diary helpful were consistent across a majority of participants, although with a minority expressing a desire to 'move on' and would not have liked a diary kept. Differences between patients and relatives arose in the areas of the purpose, content, ownership and timing of delivery of a diary. Patients viewed the diary as a therapeutic tool while relatives considered it as an information sharing mechanism, including as a mechanism to demonstrate to the patient 'how sick he really was' and 'what he put us through'. Possibly as a result of these differences, patients considered that ownership of the diary rested with them while some relatives envisaged shared ownership. Patients were more likely to note that the diary should not be provided to them until some weeks after ICU while relatives considered an early time point soon after ICU discharge to be appropriate. Patients were more likely to raise concerns about the potential negative impact of information sharing strategies including diaries and return visits to the ICU.

**Conclusions:** Patients and relatives expressed common themes related to information sharing strategies after ICU, but with some important differences. Differences in purpose, content, ownership and timing of delivery of a diary suggest there is a need to consider whether the same intervention meets the needs of both groups of stakeholders.

**Grant acknowledgement:** This project was funded by the NHMRC Centre of Research Excellence in Nursing, Menzies Health Institute Queensland, Griffith University, Australia.

### A811 PDCA for increasing effective use of CAM-ICU for delirium screening by critical care nurses

#### J.C. Azevedo, L.L. Rocha, F.F.M. De Freitas, A.M. Cavalheiro, N.M. Lucinio, M.S. Lobato

##### Hospital Israelita Albert Einstein, São Paulo, Brazil

###### **Correspondence:** M.S. Lobato – Hospital Israelita Albert Einstein, São Paulo, Brazil

**Introduction:** Confusion assessment method (CAM-ICU) is routinely used for delirium screening in ICU. In research environment, this tool has a very high sensitivity and specificity (1). However, in clinical settings, it may not be reproducible mainly because of inadequate training in CAM-ICU by bedside nurses (2).

**Objectives:** Develop a PDCA (plan-do-check-act) to train bedside critical care nurses in CAM-ICU application.

**Methods:** The study was conducted in a 600 bed tertiary private hospital in Sao Paulo, Brazil. A pre-training questionnaire to test bedside nurses about their knowledge of the correct application of CAM-ICU was applied. Later on, training sessions consisting of video lessons and practical mentored application of CAM-ICU were developed. Also, internal campaigns were developed to increase awareness about CAM-ICU between nurses. A post-training test was applied. Those who had a final test score higher than 90 % was approved. Those who had a final test score was ≤90 % were submitted to another round of training sections and another post-training test was applied until necessary score obtained. The instructors audited all in-training nurses CAM-ICU applications three months after training.

**Results:** A total of 50 nurses participated in the training, with a mean graduation time of seven years and mean hospital admission time of five years. In the pre-training questionnaire, about 83,3 % of bedside critical care nurses correctly answered questions about CAM-ICU. However when bedside application was checked, around 80 % of the nurses applied the tool correctly. The main identified causes for this were high patient turnover, demanding families, and lack of practice in CAM-ICU. After theoretical and practical training sections, all of the bedside nurses correctly answered the post-training test. In the audit period (three months after training), around 97 % of the nurses correctly applied the CAM-ICU.

**Conclusions:** An educational program enhances the correct application of CAM-ICU by bedside critical care nurses.

**References**

1. Ely EW et al. JAMA 2001;286:2703–10.

2. Van Eijik MM et al. Am J Resp Crit Care Med 2011;184:340–44.

**Grant acknowledgement**

None.

### A812 Rocking chair mobilization therapy for critically ill patients in the intensive care unit

#### G. Ebeling^1^, A. Kraegpoeth^1^, E. Laerkner^2^

##### ^1^Odense University Hospital, Anesthesiology and Intensive Care Medicine, Odense, Denmark; ^2^Odense University Hospital, University of Southern Denmark, Odense, Denmark

###### **Correspondence:** G. Ebeling – Odense University Hospital, Anesthesiology and Intensive Care Medicine, Odense, Denmark

**Introduction:** In the Intensive Care Unit (ICU) several patients are disturbed in their cerebral function due to their critical illness and medication, leading to discomfort, agitation, restlessness, pain and delirium.

Rocking Chair Mobilization Therapy (RCMT) is a chair with good seating comfort which gives rhythmic movements. Rocking chair studies have shown concrete results to improve patient satisfaction, balance and well-being in patients who suffered from dementia (1). However, no studies have evaluated the value and the effect of RCMT for critically ill patients in the ICU.

**Objectives:** The purpose of the study was to evaluate whether RCMT could be used in the rehabilitation of critically ill patients in the intensive care. The focus was to explore the impact of RCMT on critically ill patients comfort, pain, agitation and delirium.

**Methods:** The evaluation took place in a medical/surgical ICU in Denmark in the period from May to July 2015. Patients ≥ 18 years, who were physically stable and had the ability to be mobilized to chair could participate in the evaluation. The RCMT session lasted 20 minutes.Each session with RCMT was evaluated by registration of patient consciousness (Richmond Agitation and Sedation Scale (RASS)), pain (numeric rating scale (NRS) 0–10 or by Critical-Care Pain Observation Tool (CPOT)), delirium (CAM-ICU) before and after the session. Patient comfort was assessed by the patients as well as by the nurses during the session.

**Results:** 47 sessions with RCMT were evaluated. 24 males and 23 females, age between 49 and 88 years, participated in the evaluation. The results showed a decrease in patient agitation level and an increase in patient consiousness. Patients´ with RASS > 0 decreased from 18 before the session to 6 after the session. Patients with RASS ≤ −1 decreased from 5 before the session to 3 after the session. A decrease in delirium where 11 patients were assessed CAM-ICU positive before the session and 4 patients after the session. A decrease in pain where six patients scored NRS > 3 before the session compared to one patient after the session and 9 patients had CPOT scores > 2 before the session compared to 5 patients after the session. Assessment and evaluation of comfort by patients themselves and by the nurses, who cared for the particular patient, showed that RCMT was associated with a high degree of patient relaxation and comfort.

**Conclusions:** Promising results gives reason to recommend RCMT for critically ill patients in the ICU, as an alternative holistic non-pharmacological intervention to stimulate patients´ bodily awareness and enhance patient comfort and rehabilitation.

**References:**

1. Watson, Nancy M.; Wells, Thelma J.; Cox Christopher (1998) Rocking chair therapy for dementia patients: Its effect on psychosocial well -being and balance. American Journal of Alzheimer´s Disease. pp. 296–308

**Grant acknowledgement**

None

### A814 Nutritional intake and physical functioning after ICU discharge

#### I. De Brito-Ashurst

##### Royal Brompton and Harefield NHS Foundation Trust, Rehabilitation and Therapies, London, United Kingdom

**Introduction:** Critical illness and immobility in the Intensive care unit (ICU) lead to a loss of muscle mass and reduced exercise capacity for many years following hospital discharge.[1] Nutritional management of the critically ill is challenging and most nutritional studies are focused in this period. Nutritional recommendations are for a high protein diet to minimise muscle breakdown and support protein synthesis during rehabilitation. Nevertheless, during the rehabilitation period little is known of patients' protein intake and physical functioning.

**Objective:** To investigate physical functioning, frailty and dietary protein intake after 6 months of ICU discharge.

**Method:** Our ICU is recognised as a therapy rehabilitation centre and the only ICU member of the UK Rehabilitation Outcomes (UK-ROC). Patients cognitive and physical functioning is assessed as part of their rehabilitation therapy with the Functional Independence Measure (FIM) score[2]. The FIM contains 18 items on motor (13) and cognitive (5) functions that are scored on a 7-point ordinal scale based on the amount of assistance a person requires to perform specific activities. The FIM scores on ICU discharge and also on return to the rehabilitation clinic after 6 months were assessed. In addition, frailty was assessed based on a scale ranging from *very fit to very severely frail, terminally ill[3]* and patients were asked to complete a protein food frequency questionnaire.

**Results:** Twenty patients were assessed. Data are reported as mean and (standard deviation). Patients were male 66 % and 53.4 years (33.5). Paired *t tests* of the changes in FIM scores from discharge showed significant increments; 9.7 ± 11.4 (*P =* 0.05) and 24.7 ± 23.4 (*P <* 0.04) for motor and cognitive scales respectively. Nevertheless, patients reported that they were “vulnerable to moderately frail” in the frailty scale. Dietary intake was also inadequate with a protein intake of 0.83 g/kg (1.15).

**Conclusion:** There was improvement in FIM score after discharge but that was mainly from cognitive function. A lower improvement was observed in motor functioning supporting the vulnerable to moderately frail scale and a reduced protein intake.

**References**

[1] Herridge, M.S., et al. 2011. Functional disability 5 years after acute respiratory distress syndrome. *N.Engl.J.Med.*, 364(14) 1293–1304.

[2] Granger CV, Hamilton BB, Linacre JM, et al. Performance profiles of the functional independence measure. Am J Phys Med Rehabil. 1993; 72:84–89.

[3] McDermid RC, Stelfox HT, Bagshaw SM. Frailty in the critically ill: a novel concept. Critical Care. 2011; 15:301.

### A815 ICU rehabilitation treatment time: outcomes and barriers

#### C. White^1^, S. Gregory^2^, L.G. Forni^1^

##### ^1^Royal Surrey County Hospital, ICU and SPACeR research group, Guildford, United Kingdom; ^2^Royal Surrey County Hospital, Guildford, United Kingdom

###### **Correspondence:** S. Gregory – Royal Surrey County Hospital, Guildford, United Kingdom

**Introduction:** The Intensive Care Society in the UK has published core standards for the provision of physiotherapy and rehabilitation in ICU patients^1^. These state:'*Patients receiving rehabilitation should be offered a minimum of 45 minutes of each active therapy that is required, for a minimum of 5 days a week of therapy per day, at a level which enables them to meet their goals for as long as they are continuing to benefit from the therapy and are able to tolerate it.*'However these standards were adapted from the NICE Quality guidelines for stroke 2010^2^ which targets 45 minutes per day over 5 days or 32 minutes per day over 7 days, rather than the direct needs of patients with Intensive Care Unit Acquired Weakness (ICUAW).

**Objectives:** We have examined our current rehabilitation service against the recommended 32 minute target for active therapy in the critical care unit to see if we achieve these core standards.

**Methods:** We recorded the therapy time provided per day to our emergency critical care patients on a documented Critical Illness Rehabilitation Pathway. Respiratory physiotherapy was excluded in the treatment times. We excluded patients on post-surgical Enhanced Recovery Pathways (ERP's). The study time run over 4 weeks with prospective data collection.

**Results:** A total of 53 patients were included with 417 physiotherapy contacts over the study period. Patients are offered two treatment sessions per day Monday to Friday and once at a weekend, standard practice in our critical care unit. Patients were described as one of 3 categories: self-ventilating, ventilated via tracheostomy and ventilated via Endotracheal Tube (ETT).

**Conclusions:** These results demonstrate that the major limiting factors in achieving the core standards are principally driven by factors unrelated to physiotherapy provision. In particular the most significant limiting factor of the self-ventilating and tracheostomy groups was fatigue/exercise tolerance, followed by medical limitation although self-ventilating patients are more likely to refuse treatment. The ventilated via ETT group, although receiving significant respiratory physiotherapy input receive less rehabilitation due to sedation. These data could be used to help inform any potential ESICM guidelines for ICUAW therapy.

**References**

1. The Intensive Care Society. Core standards for Intensive care units 2013. Available at: https://www.ficm.ac.uk/sites/default/files/Core%20Standards%20for%20ICUs%20Ed.1%20(2013).pdf

2. NICE quality standard (QS2) Stroke in Adults 2010. Available at: https://www.nice.org.uk/guidance/qs2

3. The Sentinel Stroke National Audit Programme (SSNAP). Available at: https://www.rcplondon.ac.uk/projects/outputs/sentinel-stroke-national-audit-programme-ssnap

**Table 4 (abstract A815).** Treatment received (%)

Treatment Offered	Self-Ventilating	Ventilated via Tracheostomy	Ventilated via ETT
Bed Exercises	7.2	19.1	0
Chair Exercises	4	1.1	0
Stair practice	0.8	0	0
Balance and standing exercises	10.4	12.4	0
ROM/stretches	4.8	23.5	79
Mobility and gait re-education	45	41.5	0
Sit on edge of balance/sitting balance	7.2	10.1	0
Transfer practice	26.4	17.9	0
Positioning/UL exercises	8	11.1	31

**Table 5 (abstract A815).** Limitations to Rehab (%)

Limitations to treatment	Self-ventilating	Ventilated via Tracheostomy	Ventilated via ETT
Fatigue and Exercise tolerance	41.6	39.3	3.57
Refusal/Confusion/Anxiety	24.8	5.6	0
Pain	14.4	1.1	7.14
Procedure/Unavailable	4	8.9	10.71
Acutely unwell/CVS/medical limitations/CVVHD	29.6	20.2	71.4
Sedated/Decreased GCS	1.6	12.4	7.14
Respiratory wean	0	5.6	0
Lack of equipment/Time	1.6	3.3	3.57
Other	16.8	6.7	0

**Fig. 5 (abstract A815).**
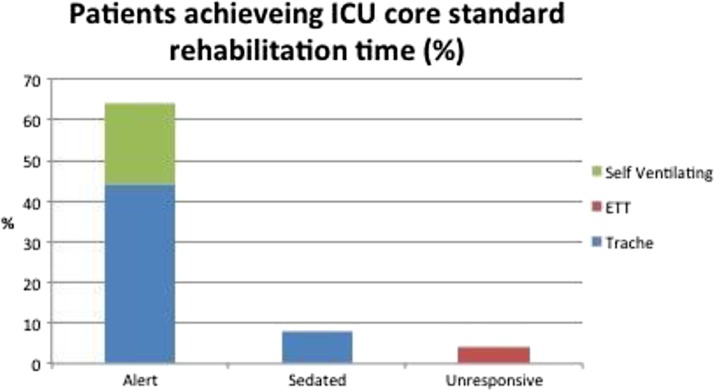
Results

### A816 Exploring current rehabilitation on ITU: can we measure 'tolerance' and 'level' of rehabilitative physiotherapy?

#### E. Flowers, A. Curtis, C.-A. Wood

##### Guys and St Thomas NHS Foundation Trust, London, United Kingdom

###### **Correspondence:** E. Flowers – Guys and St Thomas NHS Foundation Trust, London, United Kingdom

**Introduction:** Rehabilitation in the Intensive Care Unit (ICU) aims to enhance health, wellbeing and recovery beyond survival of critical illness. Current rehabilitation practice requires description and measurement of effect to enhance exercise prescription^1^.

**Objectives:** Physiotherapy rehabilitation is a recognised component of ICU care. The Intensive Care Society - Core Standards^2^ recommends that rehabilitation is '*at a level that enables the patient to meet their rehabilitation goals for as long as they…are able to tolerate it*'. In order to investigate and measure the terms '*tolerate*' and '*level*', physiological measurements and their relationship with self-perceived exertion and tolerance were analysed.

**Methods:** The project was registered with Guy's & St.Thomas' NHS Foundation Trust, Clinical Audit Group, (Project No. 4701). A convenience sample, of ICU patients undergoing active physiotherapy led rehabilitation, were observed between July and September 2014. A Modified Exertion Scale was used to measure patients' perceived effort. Patients also rated tolerance of the session using a Tolerability Scale, created based on the exertion scale. Sessions were timed, heart rate, blood pressure and oxygen saturation were monitored and the cardiovascular impact of the session measured using Heart Rate Reserve (HRR).

**Results:** Nine rehabilitation sessions were observed; mean length of 17 minutes (range 9–28). Minimum target HRR (>30 %) was achieved, but not sustained, by 3 patients, while 1 peaked within a normal target HRR (40-85 %). Of the 9 patients, 6 were able to use the tolerability scale and 7 the exertion scale. There did not appear to be a relationship between HRR and either perceived scale measurements. There did appear to be a link between perceived exertion and perceived tolerability with 5 of the 6 patients scoring within 3 points.

**Conclusions:** Reported perception of exertion and physiological markers could both indicate the 'level' patients are working at. We were able to measure effects of rehabilitation on heart rate. The majority of patients were able to use exertion and tolerance scales. However, the change in heart rate was not great enough to suggest a training effect, despite their exertion scores implying high effort levels. To fulfil the ICU society recommendations, a good understanding is needed of how hard patients are working during rehabilitation. Further research is needed to determine why there may be disparity between heart rate and patient-reported measures of exertion; and if either is a useful guide for exercise prescription with ICU patients.

**References**

1. Calvo-Ayala, E., Khan, B., O. Farber, M,. Ely, E., Boustani, M., (2013), 'Interventions to Improve the Physical Function of ICU Survivors', Chest, issue 144, no. 5, pp 1469–1480.

2. Core Standards for Intensive Care Units. Available online at [http://www.ficm.ac.uk/sites/default/files/CoreStandardsforICUsEd.1(2013).pdf] accessed on 23/12/2015

### A817 Optimising mobilisation in the critically ill - translating knowledge into clinical practice

#### K. Siu^1^, K. Venkatesan^2^, J.B.H. Muhammad^1^, L. Ng^1^, E. Seet^2^

##### ^1^Khoo Teck Puat Hospital, Rehabilitation Services, Singapore, Singapore; ^2^Khoo Teck Puat Hospital, Anaesthesia & Surgical Intensive Care, Singapore, Singapore

###### **Correspondence:** K. Siu – Khoo Teck Puat Hospital, Rehabilitation Services, Singapore, Singapore

**Introduction:** Critically ill patients are at risk of developing deconditioning, muscle atrophy and functional impairments long after hospital discharge. There is evidence demonstrating benefits of mobilization in critically ill patients - improved functional outcome and reduced ICU and hospital length of stay. However, there is limited information about how these advances are translated to clinical practice.

**Objectives:** To obtain a baseline data on patients who are eligible for mobilisation in ICU and how many of these patients are optimally mobilised in ICU. This would enable us to undertake a clinical practice improvement project (CPIP) using the Plan-Do-Study-Act (PDSA) implementation strategy to optimise mobilisation in at least 85 % of all eligible ICU patients.

**Methods: Setting.** 14-bedded intensivist led closed surgical ICU. The mobilisation team composed of physiotherapists, bedside nurses and respiratory therapists who worked along with an intensivist. Prospective audit conducted to collect data on the patients who met the eligibility criteria of mobilisation over a 3-month period. CPIP team

**Results:** Our audit revealed that at baseline, only 44 % of all eligible patients were optimally mobilised. RCA revealed a total of 21 barriers and through multi-voting and pareto-charting, we identified the top 3 barriers to change. Key barriers identified were: 1. Mobility not being a part of the daily review routine 2. Staff were unsure of the eligibility criteria 3. Lack of knowledge the benefits of optimal mobilisation in the critically ill. The team proposed following strategies to overcome the barriers: 1. Combined ICU multi-disciplinary handover rounds with the lead consultant asking the question “Can this patient be mobilised?” for every patient reviewed. 2. Providing a bedside decision-making algorithm on eligibility criteria, displayed within visibility of staff's work area. 3. Undertake sharing session with ground staff on the importance and benefits of optimising mobility of the critically ill.

**Conclusions:** Our audit revealed that less than half of eligible patients received early mobilisation. Our CPIP - a quality improvement initiative identified barriers in translating knowledge into clinical practice. Through various tools of CPIP, we identified the key barriers and strategies to overcome these barriers and thereby achieving the goal of optimising mobilisation in ICU patients.

**References**

1. Engel, Heidi J et al. (2013) ICU Early Mobilization: From Recommendation to Implementation at Three Medical Centres. Crit Care Med 41(9) S69-S80

**Fig. 6 (abstract A817).**
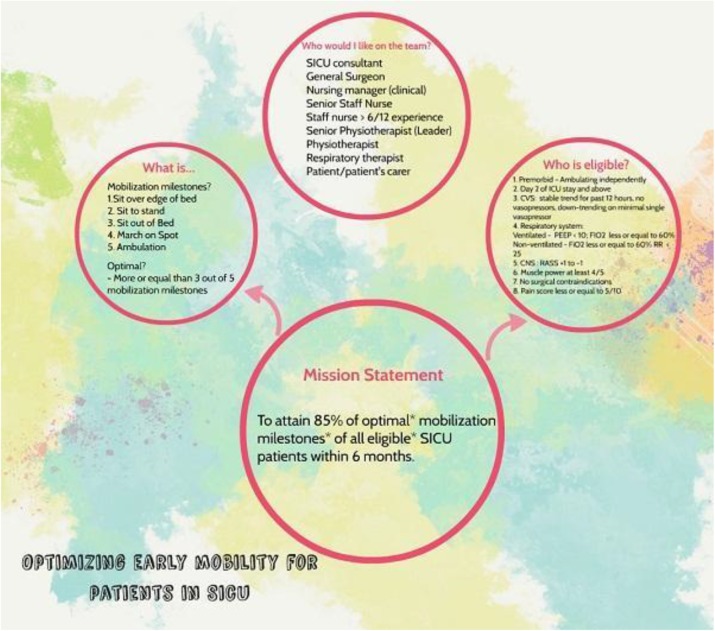
Comprised of multi-disciplinary stakeholders - frontline staff and patient representative. A brainstorming storming process identified various barriers to optimal mobilisation. Quality improvement tools such as Affinity diagram

**Fig. 7 (abstract A817).**
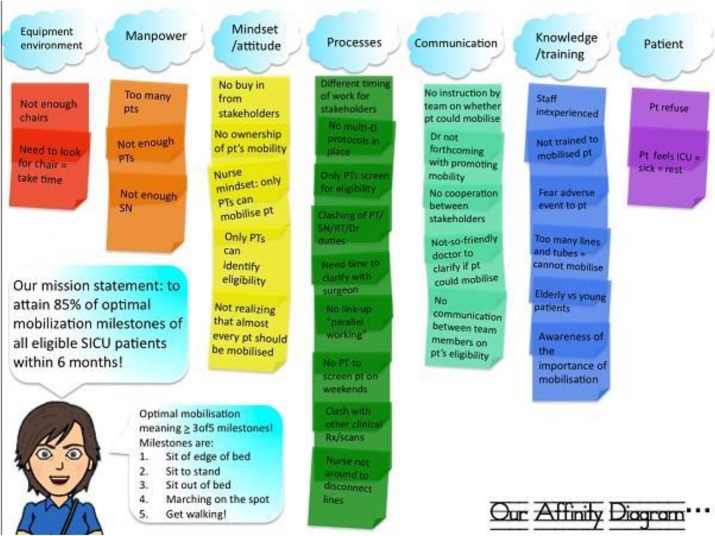
Root cause analysis (RCA)

**Fig. 8 (abstract A817).**
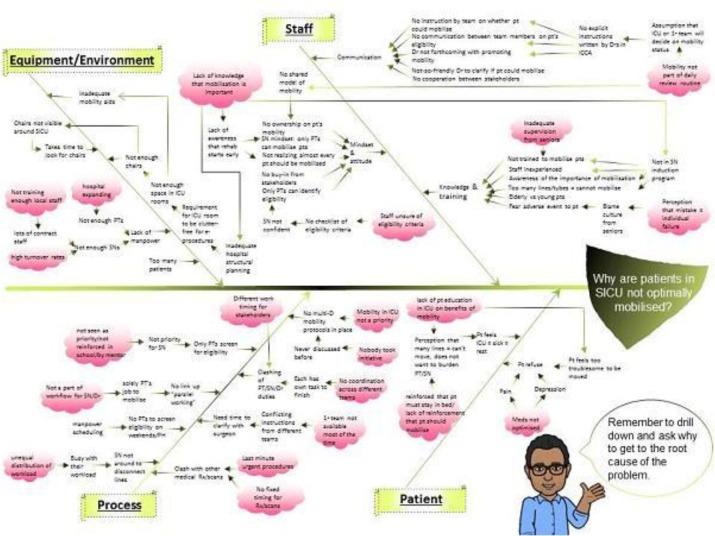
Run charts and Pareto charts were then utilized to form the prioritization matrices

### A818 Impact of physiotherapy on the quality of assistance offered by an Angolan ICU

#### N. Baptista^1^, A. Escoval^2^, E. Tomas^1^

##### ^1^Clinica Sagrada Esperança, Luanda, Angola; ^2^Escola Nacional de Saude Publica/UNL, Lisboa, Portugal

###### **Correspondence:** E. Tomas – Clinica Sagrada Esperança, Luanda, Angola

**Introduction:** Bed rest and immobility during critical illness may result in profound physical deconditioning. The multidisciplinary team in intensive care includes physiotherapists, who are responsible for performing diagnoses and procedures for critically ill patients, such as ventilation, respiratory monitoring and assessments of musculoskeletal, neurological, metabolic and cardiovascular diseases, and for the prevention and treatment of the effects of prolonged immobility.

**Objectives:** To evaluate the influence of physiotherapy on quality indicators in the intensive care unit of the Sagrada Esperança Clinic in Luanda, Angola.

**Methods:** A retrospective before-after study was designed to assess some quality indicators within the intensive care unit between July and September 2013, where there were no physiotherapists specially trained for respiratory care, and from January to March 2014, where the physiotherapists integrated a multidisciplinary team. The quality indicators analyzed were: the average duration of mechanical ventilation, prevalence of ventilator associated pneumonia and the rate of ventilated patients with non-invasive ventilation. The study population comprised 62 patients for 2013 and 71 for 2014. In this study the patients´ categorization was made by age, sex, pathology and also according with the patient classification systems SAPS 3 and SOFA. The statistical analysis used the systems SPSS version 22 for a 5 % significance level.

**Results:** The results obtained after analyzing the two homogeneous groups according to age, gender, type of admission and severity influencing the physiotherapy care in ICU quality indicators, in the Sagrada Esperança clinic, highlights the decrease of the average number of days with mechanical ventilation but it is not observed a significant relation between physical therapy and this indicator (p = 0:06).

Furthermore, it is also observed a decrease ventilator associated pneumonia, and a significant relation between this indicator and the respiratory physiotherapy. Last, there is a strong relation between the increase on the number of patients without invasive ventilation and physiotherapy (p = 0.017).

**Conclusions:** In this study it is demonstrated the respiratory therapy influences in some quality indicators, namely regarding the reduction of ventilation associated pneumonia and the promotion of non-invasive ventilation in the ICU of the CSE.

**References**

1) Pinto, W. A. M., Rossetti, H. B., Araújo, A., Spósito Júnior, J. J., Salomão, H., Mattos, S. S., Machado, F. R. (2014). *Revista Brasileira de Terapia Intensiva*, *26*(1), 7–13.

**Fig. 9 (abstract A818).**
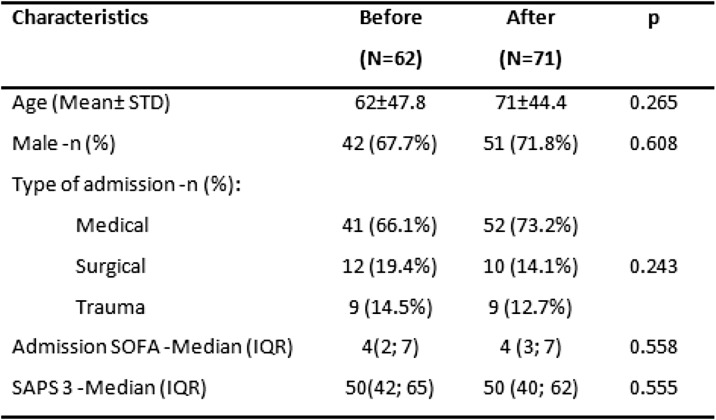
Baseline characteristics

**Fig. 10 (abstract A818).**
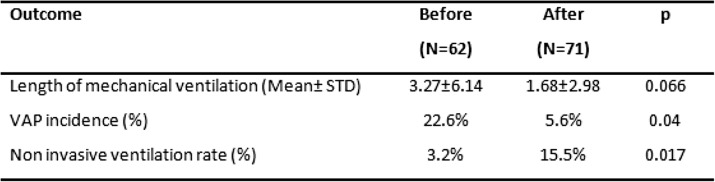
Outcome

## FUNGAL INFECTION AND INFECTION PREVENTION

### A820 Epidemiology, treatment pattern and outcomes of candida infection in non neutropenic patients in a medical icu of a developing country

#### R. Agrawal, R. Mathew, A. Varma

##### Fortis Escorts Heart Institute, Critical Care Medicine, New Delhi, India

###### **Correspondence:** R. Agrawal – Fortis Escorts Heart Institute, Critical Care Medicine, New Delhi, India

**Introduction:** The incidence of fungal infections has increased in Indian ICU`s over last few years^1^. The mortality due to unrecognized and untreated fungal infections is significantly higher.

**Objectives:** To study the incidence and epidemiology of fungal infections in a medical ICU in non neutropenic patients. We also studied the treatment pattern and patient outcomes in these critically ill patients.

**Methods:** The study was conducted from January 2015 to March 2016 over a period of fifteen months.Total patients admitted to the medical ICU were screened and patients with suspected candida infection were noted. The APACHE II score was calculated for assessing the severity of illness.Patients receiving emperical antifungal therapy and type of antifungal drugs used were also noted. The mortality rate in patients with cultures positive for candida was noted and compared to ICU mortality rate.

**Results:** There were total of 3700 patients who were admitted in the 50 bed medical ICU.Out of these 125 (3.3 %) patients were culture positive for candida. *Candida tropicalis* was the most common species followed by *C. albicans* (Table 6). The mean APACHE II score in patients with culture positive with candida was 19.2 whereas it was 17.5 in other patients (9.7 % higher in Candida group). Azoles were the most common antifungal used (58 %) followed by echinocandins (21.4 %). The crude mortality rate in patients with positive cultures for candida was 26.4 % (n = 33) which was significantly higher than ICU mortality rate of 5.5 %. (n = 197), (pvalue < 0.001).

**Conclusions:** Candida infections are a significant cause of mortality in ICU`s .*C. tropicalis* is more commonly isolated than *C albicans*. Azoles are the most common antifungals used though the usage of ecinocandins is rising. Early recognition and treatment of fungal infections is of critical importance. Avoiding overuse and promoting deescalation protocol of antibiotics can help in controlling fungal infections in the ICU.

**References**

1. Chakrabarti A. J Postgrad Med. 2005; 51(1): 16–20. 5. Dellinger et al. CCM. 2013;41:580–637

2. Pappas P, Et al. A Prospective Observational Study of Candidemia: Epidemiology, Therapy, and Influences on Mortality in Hospitalized Patients. Clin Infect Dis (2003) 37(5): 634–643.

**Grant acknowledgement**

None.

**Table 6 (abstract A820).** Candida species isolated

FAMATA	2	1.9 %
GLABRATA	14	13.4 %
GUILLIERMONDII	1	1.0 %
HAEMULONII	2	1.9 %
KRUSEI	1	1.0 %
PARAPSILOSIS	3	2.8 %
RUGOSA	2	1.9 %
TROPICALIS	45	42.3 %
ALBICANS	37	35.2 %

### A821 Candidemia in intensive care unit (ICU) patients: risk factors for non-albicans Candida species and for fluconazole resistance

#### E. Dima^1^, E. Charitidou^2^, E. Perivolioti^3^, M. Pratikaki^3^, C. Vrettou^3^, A. Giannopoulos^3^, S. Zakynthinos^3^, C. Routsi^3^

##### ^1^University of Athens, Medical School, Evangelismos Hospital, Athens, Greece; ^2^National Technical University of Athens, Athens, Greece; ^3^Evangelismos Hospital, Athens, Greece

###### **Correspondence:** E. Dima – University of Athens, Medical School, Evangelismos Hospital, Athens, Greece

**Introduction:** The incidence of candidemia has increased in ICU patients (1). In addition, there are differences in epidemiology among different countries. We have previously shown an increased proportion of non-albicans Candida species in our ICU (2).

**Objectives:** To identify the variables associated with candidemia due to non- albicans Candida species, as well as with fluconazole-resistant strains in a multidisciplinary ICU.

**Methods:** All ICU patients with candidemia were prospectively studied over two time periods (2005–2008 and 2012–2015). Demographics, illness severity, clinical and laboratory variables were recorded. SOFA score value on ICU admission subtracted from the value on the day of candidemia occurrence was defined as Delta SOFA. Patients with C. albicans candidemia were compared to those with non-albicans candidemia. Also, patients with fluconazole-resistant candidemia were compared to those without fluconazole resistance.

**Results:** Among 143 patients with ICU-acquired candidemia, in 55 patients candidemia was due to C. albicans and in 88 patients to non-albicans species. C. parapsilosis was the most common (46 %) followed by C. albicans (38 %). The median time from ICU admission to candidemia onset was 12 and 23 days for C. albicans and non-albicans respectively, p = 0.02. Similarly, the median time for candidemia due to fluconazole sensitive isolate was 14 days and 32 days for fluconazole resistant, p < 0.001. Resistance to fluconazole was 9 % and 51 % in C. albicans and in non-albicans species respectively, p < 0.001).Presence of shock on candidemia day (OR 6.85; CI: 2.2-25, p = 0.001) and the Delta SOFA score (OR 0.74; CI: 0.60-0.89, p = 0.002) were independently associated with candidemia due to C. albicans. Independent risk factors for fluconazole resistant isolates were the length of ICU stay before the development of candidemia (OR 1.03; CI: 1.01-1.05, p = 0.003) and the presence of shock on candidemia day (OR 0.23; CI: 0.07-0.64, p = 0.006). Previous fluconazole exposure (10 patients) was not associated with fluconazole resistance.

**Conclusions:** This study confirms the predominance of non-albicans Candida species, in our ICU patients with candidemia, with high prevalence of fluconazole resistance. Early onset of candidemia and the presence of shock were most likely due to C. albicans whereas late onset was associated with fluconazole-resistant non- albicans species. These findings may be of value for empiric antifungal treatment selection.

**References**

(1) Intensive Care Med 2014;40:1303

(2) Mycoses 2011;54:154

### A822 Early fungal infections after lung transplantation

#### E. Atchade, S. Houzé, S. Jean-Baptiste, G. Thabut, C. Genève, S. Tanaka, B. Lortat-Jacob, P. Augustin, M. Desmard, P. Montravers

##### Hopital Bichat Claude Bernard, Paris, France

###### **Correspondence:** E. Atchade – Hopital Bichat Claude Bernard, Paris, France

**Introduction:** Fungal infections (FI) after lung transplantation (LT) are common and associated with high mortality and morbidity rates (1). Published studies report late invasive infections caused by *Aspergillus sp*, but the early post-transplant period in intensive care unit (ICU) patients is rarely assessed.

**Objectives:** The primary goal of the study was to describe the epidemiology of early FI in ICU after LT. Secondary aims were to evaluate its impact on the outcome on ICU stay and to determine the risk factors for fungal colonisation.

**Methods:** This observational, retrospective, monocentric study analysed microbiologic results, clinical evolution and outcome of 176 LT, in ICU, during a 6-year period. Fungal positive respiratory sample was considered as colonisation when no clinical, radiological or histological criteria for invasive infection were present. Results are expressed as median and interquartile range. Statistical analyses were performed using Chi square, Mann-Whitney and Kruskal Wallis tests. The level of statistical significance was set at 5 %. The local Ethic Committee approval was obtained for the study.

**Results:** During the pre-transplantation period, *Candida sp* colonisation was reported in 17 % of the patients (87 % *C. albicans*), while *Aspergillus sp* colonisation was observed in 4 % of them. In the post-transplantation period, 69 % of patients were colonised with fungi, mainly *C.albicans* (33 % of cases), rarely *Aspergillus spp* (7 %). Median time to onset of *Candida* colonisation was 4 days [1–8] and 5 days [3–14] for *Aspergillus* colonisation. Fungi were significantly associated with the presence of Enterobacteriaceae (OR = 2.61, 95%CI [1.36-5]; p = 0.003) and enterococci (OR = 5.09, 95%CI [1.14, 22.75]; p = 0.02). Risk factors for fungal post-operative colonisation were bi-pulmonary transplantation (OR = 2.49, 95%CI [1.15-5.45], p = 0.02) and COPD (OR = 2.71, 95%CI [1.39-5.32], p = 0.003). Among the patients developing candida colonisation in the post-transplantation period, 57 % received an antifungal treatment during their ICU stay (10 % echinocandins, 97 % azoles). A significant association between pre-transplant *Candida sp* colonisation and mortality rate in ICU was observed (OR = 2.35, 95%CI [1.14-4.9], p = 0.03). Post-operative fungal colonisation was not associated with increased death rate in ICU (OR = 1.32, 95%CI [0.62, 2.82], p = 0.57) or duration of mechanical ventilation ≥3 days (OR = 1.65, 95%CI [0.87-3.12]) while post-operative *Candida sp* colonisation was associated with prolonged ICU stay (p < 0.001) and increased duration of mechanical ventilation (p = 0.002).

**Conclusions:** Prevalence of fungal colonisation is high after LT, most commonly caused by *Candida sp*. The lack of association between post-transplant fungal colonisation and mortality and morbidity suggests to avoid antifungal therapy when no clinical signs of fungal infection are observed.

**References**

(1) Solé A, Transplant Rev Orlando Fla, 2008; 22(2): 89–104

**Grant acknowledgement:** None.

### A823 Clinical significance of *Aspergillus* isolation in critically ill H1 N1 patients

#### F.J. González de Molina^1,2^, S. Barbadillo^3,4^, R. Alejandro^5^, F. Álvarez-Lerma^6^, J. Vallés^7^, R.M. Catalán^8^, E. Palencia^9^, A. Jareño^10^, R.M. Granada^11^, M.-L. Ignacio^12^, GETGAG Working Group

##### ^1^Hospital Universitari Mútua de Terrassa, Intensive Care Department, Terrassa, Spain; ^2^AGAUR, Grup Recerca Emergent, Terrassa, Spain; ^3^Hospital General de Catalunya, Intensive Care Department, Sant Cugat del Vallés, Spain; ^4^Universidad Autónoma de Barcelona, Departament de Medicina, Barcelona, Spain; ^5^Hospital Universitari de Tarragona Joan XXIII, Intensive Care Department, Tarragona, Spain; ^6^Hospital del Mar, Intensive Care Department, Barcelona, Spain; ^7^Hospital Parc Taulí, Intensive Care Department, Sabadell, Spain; ^8^Hospital General de Vic, Intensive Care Department, Vic, Spain; ^9^Hospital Infanta Leonor, Intensive Care Department, Madrid, Spain; ^10^Hospital del SAS de Jerez, Intensive Care Department, Jerez de la Frontera, Spain; ^11^Hospital de Bellvitge, Intensive Care Department, Barcelona, Spain; ^12^St James's University Hospital, Dublin, Ireland

###### **Correspondence:** F.J. González de Molina – Hospital Universitari Mútua de Terrassa, Intensive Care Department, Terrassa, Spain

**Introduction:** Invasive *Aspergillus* infections are well-known complications of immunocompromised states, chronic obstructive pulmonary disease and haematopoietic stem cell transplant. Bacterial coinfection is well described in influenza literature but there is scarce data on invasive aspergillosis complicated severe influenza infection.

**Objectives:** The aim of this study is to describe the clinical and demographic characteristics of patients with *Aspergillus* isolation in severe influenza A(H1 N1) pneumonia.

**Methods:** Prospective, observational, multicenter study conducted in 148 Spanish ICUs from 2009 to 2015. All individuals with severe primary influenza A(H1 N1) pneumonia requiring invasive mechanical ventilation were included in the study. Influenza A(H1 N1) patients without coinfection were compared with those with *Aspergillus* isolation in respiratory samples. All serotypes were confirmed using RT-PCR at ICU admission. Patients´ demographic, clinical, radiologic features, laboratory values, ICU and hospital length of stay (LOS) and outcomes were recorded. Discrete variables are expressed as counts (percentage) and continuous variables as medians with 25th to 75th interquartile range (IQR). Differences between groups were assessed using the x2 test and the Fisher exact test for categoric variables and Mann-Whitney U test for continuous variables.

**Results:** Of 1594 intubated patients with confirmed influenza A (H1 N1) pneumonia at ICU admission, 385 were excluded due to other microorganism coinfection. At all, 1185 patients with H1 N1 pneumonia were compared to 24 patients with H1 N1 pneumonia and *Aspegillus* isolation (AI) in respiratory samples. Patients with AI were older (64 [54–71] vs 49 [38–60], P < 0.001), presented a higher proportion of COPD (39.1 % vs 17.8 %, P =0.024), chronic renal failure (21.7 % vs 7.1 %, P = < 0.023), and immunodeficiency (34.8 % vs 10.8 %, P =0.002). Patients with AI developed more acute kidney injury (47.6 % vs 28.0 %, P =0.048) and were treated more frecuently with corticosteroids (71.4 % vs 47.1 %, P = < 0.044). Overall mortality was much higher in those patients with AI (65.2 % vs 29.6 %, P < 0.001).

**Conclusions:** The mortality rate was significantly higher in H1 N1 patients with *Aspergillus* isolation in respiratory samples. Diagnosis of invasive aspergillosis in critically ill patients in the post-influenza era must be re-evaluated. Clinical studies should be conducted in order to know the clinical significance of *Aspergillus* isolation in respiratory samples in intubated patients with primary influenza A(H1 N1) pneumonia.

**References**

1.- Wauters J, et al. Invasive pulmonary aspergillosis is a frequent complication of critically ill H1 N1 patients: a retrospective study. Intensive Care Med. 2012;38(11):1761–8.

2.- Adalja AA, et al. Isolation of Aspergillus in three 2009 H1 N1 influenza patients. Influenza Other Respir Viruses. 2011;5(4):225–9.

### A824 Initial Therapeutic Strategy Of Invasive Candidiasis For Intensive Care Unit Patients: An Analysis From The China-Scan Study

#### N. Cui^1^, D. Liu^1^, H. Wang^1^, L. Su^1^, H. Qiu^2^, R. Li^3^

##### ^1^Peking Union Medical College Hospital, Critical Care Medicine, Beijing, China; ^2^Nanjing Zhongda Hospital, Southeast University School of Medicine, Critical Care Medicine, Nanjing, China; ^3^Peking University First Hospital, Peking University, Research Center for Medical Mycology, Beijing, China

###### **Correspondence:** N. Cui – Peking Union Medical College Hospital, Critical Care Medicine, Beijing, China

**Introduction:** The empiric or pre-emptive approach can be used as a better target therapy in antifungal treatment and affect mortality.

**Objectives:** To investigate the impact of initial antifungal therapeutic strategies on the prognosis of invasive Candida infections (ICIs) in intensive care units (ICUs) in China.

**Methods:** A total of 306 patients with proven ICIs in the China Survey of Candidiasis study were analyzed. Empiric, pre-emptive, and targeted therapy were adopted based on starting criteria including clinical, microbiological, and other conventional prediction rules. The primary outcome was ICU/hospital mortality.

**Results:** Compared with the empirical initial antifungal therapy and targeted initial antifungal therapy, patients with pre-emptive initial antifungal therapy had significantly less clinical remission [11/53 (21.2 %) vs. 61/142 (43.3 %) vs. 22/73 (30.1 %), p = 0.009], higher ICU [26/53 (57.8 %) vs. 42/142 (32.2 %) vs. 27/73 (43.5 %), p = 0.008] and hospital mortality [27/53 (60.0 %) vs. 43/142 (32.8 %) vs. 29/73 (46.8 %), p = 0.004] and more microbiological persistence [9/53 (17.0 %) vs. 6/142 (4.2 %) vs. 9/73 (12.3 %), p = 0.011]. Kaplan-Meier survival analysis revealed that ICI patients with pre-emptive initial antifungal therapy and targeted initial antifungal therapy were associated with reduced hospital duration compared with patients with empirical initial antifungal therapy after confirmation of fungal infection (log-rank test: p = 0.021).Multivariate regression analysis provided evidence that initial empirical antifungal therapy was an independent predictor for hospital mortality in ICI patients[odds ratio 0.349(95 % confidence interval 0.168-0.724); p = 0.005).

**Conclusions:** The initial therapeutic strategy for invasive candidiasis was independently associated with hospital mortality. Prompt empirical antifungal therapy could be critical to decrease early hospital mortality.

**Acknowledgement**

This study was supported by Merck Sharp & Dohme China. We would like to thank the patients and investigators who participated in this study. We also acknowledge the investigators at each study site, without whom this study would not have been possible.

### A825 De-escalation of antifungal treatment in critically ill patients: incidence, associated factors and safety

#### K. Jaffal^1^, A. Rouzé^1^, J. Poissy^1^, B. Sendid^2^, S. Nseir^1^

##### ^1^Lille University Hospital, ICU, Lille, France; ^2^Lille University Hospital, Mycology and Parasitology Lab, Lille, France

###### **Correspondence:** S. Nseir – Lille University Hospital, ICU, Lille, France

**Introduction:** Although antifungal treatment is common in critically ill patients, only a small proportion of patients receiving antifungal treatment have confirmed fungal infection. Side effects of this treatment include toxicity, resistance and unnecessary high costs. In addition, recent studies suggested no benefit of empirical antifungal treatment in these patients.

**Objectives:** The aim of this study was to identify the incidence, associated factors, and safety of de-escalation of antifungal treatment.

**Methods:** Retrospective study, conducted in a 30-bed mixed ICU, during a 1-year period. All patients hospitalized for >5d and treated with antifungals for first suspected or proven fungal infection were included. Patients receiving prophylactic antifungals were excluded. De-escalation was defined as switch from initial systemic antifungal therapy drugs (except fluconazole) to triazoles, or stopping initial drugs within 6 days following their initiation. Patients with de-escalation were compared with those with no de-escalation using univariate and multivariate analysis.

**Results:** Among the 234 patients who received systemic antifungals, 44 % received empirical, 23 % preemptive, and 33 % targeted treatment. Caspofungin (51 %), fluconazole (34 %), voriconazole (11 %), and liposomal amphotericin B (2 %) were the most frequently used antifungals. Antifungal treatment was de-escalated in 48 (20.5 %) patients.

Factors associated with higher rate of de-escalation in univariate analysis were: sterile repeated mycological samples, empirical treatment, preemptive treatment, positive bacterial samples, apyrexia, and catecholamine withdrawal at 72 hours after initiation of antifungal treatment. Multifocal *Candida* colonization, and mechanical ventilation were associated with significantly lower rate of de-escalation by univariate analysis.

In multivariate analysis, apyrexia at 72 h (OR 15 [2–120], p = 0.009), empirical treatment (3.5 [1.7-7.5], p = 0.001), and mechanical ventilation (0.22 [0.08-0.62], p = 0.004) were independently associated with de-escalation.

No significant difference was found in duration of mechanical ventilation (med (IQR) 17 (5, 30) vs 20d (10, 35), p = 0.06), or length of ICU stay (24 (14, 40) vs 25d (14, 39) p = 0.79) between patients with de-escalation, and those with no de-escalation, respectively. Patients with de-escalation had lower ICU mortality rate, compared with those with no de-escalation (42 % vs 60 %, p = 0.017). However, 1-year mortality rate was similar in the two groups (60 % vs 66 %, p = 0.46).

**Conclusions:** De-escalation was performed in 20.5 % of patients receiving systemic antifungals. Factors independently associated with de-escalation were apyrexia at 72 hours after initiation of antifungals, empirical treatment strategy, and mechanical ventilation. De-escalation of antifungal treatment seems to be safe in critically ill patients.

### A826 Epidemiology and predictors of candidemia mortality in a critical care setting

#### E. Paramythiotou, M. Rizos, F. Frantzeskaki, A. Antoniadou, S. Vourli, L. Zerva, A. Armaganidis

##### Attikon University Hospital, Athens, Greece

###### **Correspondence:** E. Paramythiotou – Attikon University Hospital, Athens, Greece

**Introduction:** Invasive candidiasis is an important cause of morbidity and mortality in the nosocomial setting and particularly in the Intensive Care Unit (ICU). Candidemia is ranked fourth as the cause of bloodstream infection in USA and it is the cause of 7 % of positive blood cultures in Europe. The increasing incidence of non-albicans Candida species is a matter of concern.

**Objectives:** The aim of the present study was to record the epidemiology, risk factors, mortality, strain susceptibility to antifungal drugs, and to evaluate Ostrosky rule's capability to predict invasive candidiasis.

**Methods:** This is a clinical and microbiological retrospective study of all candidemia episodes which were registered during a ten - year period (between 1/1/2004 and 31/12/2014). Patient identification was performed through the records of the laboratory of Attikon university hospital. Medical records were then retrieved. Only the first candidemia episode was evaluated. Special forms were completed for each patient including demographic information, concomitant conditions, Apache II and Sofa severity scores the day of ICU admission, the risk factors within the preceding 10 days, data of colonization and candidemia related information.

**Results:** Attikon hospital is a 640 - bed teaching tertiary care hospital with a 25 - bed medical and surgical ICU. Among them 70 patients developed candidemia. For 7 patients the medical records were incomplete so they were excluded. Mean patients' age was 67 years ± 15.22. Median ICU length of stay was 40.74 + 36 days. Former duration of hospitalization was 16 + 16 days. Medical cause of admission was present in 29 cases and surgical in 34 cases. Species isolated included *C. albicans* 16***,****C.parapsilosis 15 , C. tropicalis* 5 , *Candida* spp, 18, *C.glabrata* 4, *C krusei* 2, and C. non - albicans 3. Median time elapsed between ICU admission and candidemia was 19 days (3–85). Mean Apache II score was 19.5 ± 6.4 (range 12–37) on the day of admission, SOFA score 8 ± 3.6 and overall mortality was 74.6 %. Candidemia was considered the cause of death in 13 cases (20.6 %). Ostrosky's prediction rule was positive in 30 patients. Thirteen pts were submitted to an intra-abdominal operation. Blood cultures were sterilized in thirty patients. Twenty pts received TPN prior to candidemia episode. Fourteen pts were receiving steroid therapy and three were receiving immunocompromising therapy. Caspofungin was the most commonly introduced treatment.

**Conclusions:** The burden of fungemia episodes is not very high when compared to other blood infections but they are associated with a high mortality perhaps due to the severity of the underlying disease. A high Apache II score at admission, multiple site colonization in combination with abdominal surgery should raise a high suspicion index and a prophylactic therapy should start. *Non - albicans* species are increasing.

### A827 Host or worst? Role of *enterococcus* and *candida* in respiratory cultures in lung transplant recipients

#### J. Riera^1,2,3^, J. Gottlieb^4,5^, M. Greer^4,5^, O. Wiesner^4^, M. Martínez^1^, M. Acuña^1^, J. Rello^1,3^, T. Welte^4,5^

##### ^1^Vall d'Hebron University Hospital, Critical Care, Barcelona, Spain; ^2^Universitat Autònoma de Barcelona, Barcelona, Spain; ^3^CIBERES, Madrid, Spain; ^4^Hannover Medical School, Department of Respiratory Medicine, Hannover, Germany; ^5^German Centre of Lung Research (DZL/BREATH), Hannover, Germany

###### **Correspondence:** J. Riera – Vall d'Hebron University Hospital, Critical Care, Barcelona, Spain

**Introduction:** Lung transplant (LT) recipients often receive antibiotic treatments and prophylaxis that may modify the normal respiratory microbiome [1]. Recent studies suggest that a priori non-pathogenic organisms, such as enterococci and candida, may cause infection, especially in immunosuppressed patients [2,3]. In the LT recipient it can be difficult to distinguish between organisms causing pneumonia, tracheobronchitis or if they are merely colonizers [4].

**Objectives:** Assess the association between candida spp and enterococcus spp isolates in bronchoalveolar lavage (BAL) with signs and symptoms of respiratory infection, and compare this association with 2 control groups: the first formed by BAL with positive cultures for pseudomonas aeruginosa and the second one by sterile BAL.

**Fig. 11 (abstract A827).**
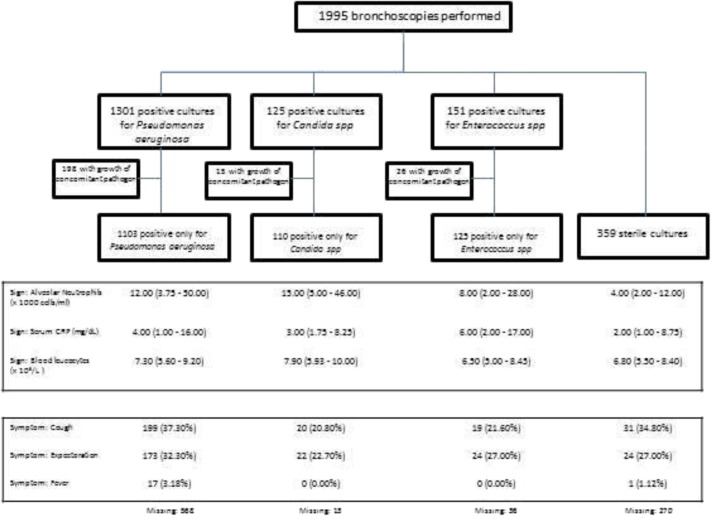
ᅟ

**Methods:** Retrospective analysis of bronchoscopies performed in LT recipients at Hannover Medical School between January 2008 and August 2015. BAL cultures positive for candida spp, enterococcus spp and pseudomonas aeruginosa were identified, as well as sterile cultures (Fig. 11) Reported symptoms and signs suggestive of respiratory infection at the time of the bronchoscopy were then evaluated. Symptoms were recorded since August 2012 using a questionnaire fulfilled by the patients.

**Results:** BAL cultures from 1995 bronchoscopies were analyzed. In total 110 BAL cultures positive for candida spp were identified along with 125 for enterococcus spp, 1103 for pseudomonas aeruginosa and 359 sterile samples (Figures are displayed in pic_01). Levels of alveolar neutrophils in the BAL of patients with positive cultures for candida spp were not different than those of patients with positive cultures for pseudomonas aeruginosa. The same was true for serum CRP (P = 0.45) and blood leucocytes counts (P = 0.09). Patients with positive cultures for enterococcus spp had lower number of alveolar neutrophils compared with patients with pseudomonas aeruginosa (P = 0.02) and lower blood leucocyte count (P = 0.01). No differences were found between the serum level of CRP (P = 0.15). Patients with candida spp positive cultures had significantly more alveolar neutrophils (P < 0.01), higher blood leucocytes (P < 0.01) and higher serum CRP (P = 0.04) than patients with sterile BAL. Patients with enterococcus spp positive cultures had more alveolar neutrophils (P < 0.001) and higher serum CRP (P = 0.04) than patients with sterile BAL. No differences regarding blood leucocytes (P = 0.25) were evident.

**Conclusions:** Candida spp and enterococcus spp may cause some degree of inflammation in the lung allograft. It would be reasonable to treat a LT recipient with signs of infection and positive BAL cultures for candida spp. Enterococcus spp may cause a milder degree of inflammation.

**References**

1. Charlson et al. AJRCCM 2012;186:536–45.

2. Albert at el. ICM 2014;40:1313–22.

3. Bucheli et al. Trans Inf Dis 2014;16:26–36.

4. Riera et al. ERJ 2015;45:726–37.

**Grant acknowledgement**

Supported by FUCAP and CIBERES.

### A828 Use of biomarkers in Candida sp infections after lung transplantation

#### E. Atchade, T. Mignot, S. Houzé, S. Jean-Baptiste, G. Thabut, B. Lortat-Jacob, S. Tanaka, P. Augustin, M. Desmard, P. Montravers

##### Hopital Bichat Claude Bernard, Paris, France

###### **Correspondence:** E. Atchade – Hopital Bichat Claude Bernard, Paris, France

**Introduction:***Candida sp*. colonisation in respiratory tract is frequent after lung transplantation (LT), but only a small proportion of lung recipients develop an invasive candida infection (1). The distinction between colonisation and invasive infection remains difficult leading clinicians to use biomarkers in their decision making process.

**Objectives:** To assess the value of fungal biomarkers in the diagnosis of *Candida sp* infections after LT.

**Methods:** This prospective, monocentric, study performed over 12-months assessed the value of a twice weekly dosage of fungal biomakers (*Candida* serology IgA, IgM, IgG, ß-D-Glucan (BDG) and mannan antigens) in ICU patients after LT. Proven/probable/possible infection was defined according to the EORTC/MSG criteria. Colonisation was defined by presence of *Candida sp* in respiratory samples without any sign of invasive infection. The study was approved by the Ethical Committee. Results are presented as means.

**Results:** We analysed 39 ICU patients after LT. 77 % had a *Candida sp* colonisation while an invasive infection was proven in 4 (10 %) patients. *Candida albicans* was cultured from 72 % of the pulmonary samples. 50 % of the invasive infections were related to *C. glabrata*. Results of biomarkers dosages are presented in the table. Positive *Candida* IgG serology was observed in 45 % of the cases. Mortality rate at 6-months after LT was 71 % in the immunised patients versus 26 % in non-immunised patients. An invasive candidiasis (IC) was present in 14 % of the immunised patients versus 10 % in non-immunised patients. At least one BDG dosage was positive in 60 % of the cases. BDG dosage value decreased after surgery, reaching a non-significant value after the 4th day. In proven IC, BDG measurements reached concentrations >500 pg/ml, 16 days before initiation of antifungal treatment. No patient had positive mannan antigen measurement.

**Conclusions:** A twice weekly dosage of BDG seems to be useful in the decision making process for early initiation of antifungal therapy in LT patients. The cutoff for a significant value of BDG needs to be defined. Pre-transplantation assessment of *Candida* IgG serology could help to identify patients at risk of post-operative fungal infection.

**References**

(1) Flume, Am J Respir Crit Care Med,1994; 149(6):1601–7.

**Grant acknowledgement**

None.

**Table 7 (abstract A828).** Dosage of biomarkers after LT

	Colonised pts		Non colonised pts		Infected pts
Ischemic bronchitis	-	+	-	+	
BDG+,%;mean (pg/mL)	14;32	50;125	11;38	50;63	100;239
IgM + , %	10	0	0	0	0
Ig G +, %	25	56	0	0	33
Ig G + < 7 days, %	20	38		0	33
IgG seroconversions, %	0	21	-	0	100
Ig A +, %	0	14	0	0	33
Mannan antigen +, %	0	0	0	0	0

### A829 Curative or pre-emptive antifungal therapy in invasive candidiasis in severely burned patients?

#### S. Soussi^1^, E. Dudoignon^1^, A. Ferry^1^, M. Chaussard^1^, M. Benyamina^1^, A. Alanio^2^, S. Touratier^3^, M. Chaouat^4^, M. Lafaurie^5^, M. Mimoun^4^, A. Mebazaa^1^, M. Legrand^1^

##### ^1^AP-HP, GH St-Louis-Lariboisière, Department of Anesthesiology and Critical Care and SMUR and Burn Unit, Paris, France; ^2^AP-HP, GH St-Louis-Lariboisière, Mycology Unit, Paris, France; ^3^AP-HP, GH St-Louis-Lariboisière, Pharmacy Unit, Paris, France; ^4^AP-HP, GH St-Louis-Lariboisière, Plastic Surgery and Burn Unit, Paris, France; ^5^AP-HP, GH St-Louis-Lariboisière, Department of Infectious Diseases, Paris, France

###### **Correspondence:** S. Soussi – AP-HP, GH St-Louis-Lariboisière, Department of Anesthesiology and Critical Care and SMUR and Burn Unit, Paris, France

**Introduction:** The antifungal (AF) therapy strategy (pre-emptive vs culture based treatment) in intensive care unit is a matter of debate [1]. The necessity to not delay the initiation of the AF in invasive candidiasis (IC) must be balanced with the cost and risk of selecting resistant pathogens when AF are prescribed too widely. Burn patients are at risk of IC because of the frequent use of antibiotics and immunodeficiency.

**Objectives:** To evaluate our antifungal (AF) therapy strategy in suspected or proven IC in terms of prognosis and risk factors of IC.

**Methods:** Observational, descriptive, retrospective study conducted from June 2012 to September 2015 in the Saint Louis Hospital Burn Unit. Inclusion criteria: patients treated with pre-emptive (severe sepsis or septic shock with Candida Sp colonization) or curative (proven, PIC) AF. The outcome was the PIC (candidemia and/or positive peritoneal sample). Clinical characteristics, organ supports, AF treatments and outcome were collected and compared between PIC and suspected IC (SIC). The results are presented in median (IQR) or n (%).

**Results:** 715 patients were admitted during the study period including 184 with a total body surface area (TBSA) >20 %. 44 treated with AF including 14 PIC (32 %). In those 44 patients: age 55 (43–67), TBSA (43–67), SAPSII 41 (29–53), ABSI 10 (8–12) and SOFA 10 (5–15). Renal replacement therapy 22 (50 %), mechanical ventilation 36 (82 %), parenteral nutrition 7 (16 %). Inhospital mortality = 60 % (68 % SIC vs 32 % PIC, p = 0.33). 3 patients with PIC (21 %) were treated before the IC diagnosis (2 because of filamentous infection before the PIC). The delay between admission and AF treatment initiation was 16 days. Patients characteristics, organs supports were not significantly different between PIC and SIC at the treatment initiation except for the SAPSII (PIC 43 (29–57) VS SIC 37 (26–49), p = 0.03). 30 patients (68 %) received an echinocandin as a first-line treatment. 6 (5.5-6.5) sites were monitored for Candida colonization the week before treatment initiation. Patients with PIC had higher colonization index than those with SIC (55 % vs 33 %, p = 0.01) and a candida score significantly higher (4 vs 3 (0.5-3.5) respectively, p = 0.03). A semi-quantitative estimation of the fungal inoculum had no predictive value.

**Conclusions:** In this study, the majority of PIC were treated after diagnosis confirmation. Only 1/30 (3 %) patient treated preemptively did declare a PIC. The outcome was not different when the treatment was initiated after confirmation. The results of this study highlight the difficulty to identify patients at highest risk of IC, and question the strategy of preemptive treatment in this population.

**References**

[1] Intensive care Med (2014) 40: 1429–48

### A830 An observational study demonstrating a possible link between a procalcitonin driven reduction in antibiotic use and systemic fungal infections

#### M.A. Sheils^1^, C. Patel^1^, L. Mohankumar^2^, N. Akhtar^1^

##### ^1^Dudley Group of Hospitals NHS Foundation Trust, Anaesthetics, Dudley, United Kingdom; ^2^Dudley Group of Hospitals NHS Foundation Trust, Microbiology, Dudley, United Kingdom

###### **Correspondence:** M.A. Sheils – Dudley Group of Hospitals NHS Foundation Trust, Anaesthetics, Dudley, United Kingdom

**Introduction:** Invasive candida infections are implicated in 10 % of intensive care acquired bloodstream infections with a reported mortality of 42.5 %. Amongst the risk factors for invasive fungal infections is exposure to broad spectrum antibiotics and fungal colonisation of mucosal surfaces. Procalcitonin (PCT) guided prescribing algorithms have been proven to safely reduce patient exposure to antibiotics.

**Objectives:** We wanted to determine whether PCT guided antibiotic rationalisation could reduce fungal colonisation and antifungal usage.

**Methods:** We undertook a retrospective observational study at a nine bedded ICU department in the United Kingdom. We collected data on all patients admitted to the unit in the year prior and post the introduction of PCT guided rationalisation of antibiotics. We used the pharmacy database to assess the use of antibiotics, correcting for changes in costs over this time. We used the microbiology database to assess the rate of patients colonising fungal species and those requiring treatment.

**Results:** Since the introduction of PCT, the average expenditure on antibiotics per ICU admission fell 35.11 % (p 0.01). The rate of ICU patients colonised with a fungal species fell from 32.3 % to 14.89 % (p < 0.0001). The incidence of patient's prescribed systemic antifungal therapy fell from 14.58 % to 4.26 % (p < 0.0001).

**Conclusions:** We demonstrated a significant reduction in patients colonised with fungal species and those requiring anti-fungal therapy since introducing PCT guided rationalisation of antibiotics. A prospective randomised controlled trial is required to assess whether this equates to improved patient outcome.

**References**

1) Kett DH, Azoulay E, Echeverria PM, Vincent JL. Candida bloodstream infections in intensive care units: Analysis of the extended prevalence of infection in intensive care unit study. Crit Care Med. 2011;39:665–670.

2) Eggimann P, Bille J, Marchetti O. Diagnosis of invasive candidiasis in ICU. Ann Intensive Care. 2011; 1: 37. (http://www.ncbi.nlm.nih.gov/pmc/articles/PMC3224461/).

3) Eggimann P, Garbino J, Pittet D. Epidemiology of *Candida* species infections in critically ill non-immunosuppressed patient. Lancet Infect Dis. 2003;3:685–702. doi: 10.1016/S1473-3099(03)00801-6.

4) Http://www.who.int/dg/speeches/2012/amr_20120314/en/index.html

5) Karlsson S, Heikkinen M, Pettilä V et al.: Predictive value of procalcitonin decrease in patients with severe sepsis: a prospective observational study. Crit care 2010,

**Grant acknowledgement**

None

### A831 Invasive fungal infections in critically ill burn patients admitted to a colombian national referral intensive care unit (ICU)

#### S.K. Pacheco Noriega, N. Navarrete Aldana, J.L. Ávila León, J. Durand Baquero, F. Fernández Bernal

##### Hospital Simón Bolívar, Burn ICU, Bogotá, Colombia

###### **Correspondence:** S.K. Pacheco Noriega – Hospital Simón Bolívar, Burn ICU, Bogotá, Colombia

**Introduction:** Invasive fungal infections remain a challenge in burn intensive care. In our country there is not available data about the epidemiology of the fungal species involved, the correct management, and the patients' outcome.

**Objectives:** To identify 1) the fungal organism/sp most commonly isolated 2) the fungal infection most commonly diagnosed based on microbiological data 3) the most prevalent risk factors involved 5) the most used antifungal agent.

**Methods:** An observational, descriptive, and retrospective study was done including consecutive patients from June 2012 to December 2015. Setting: National referral Burn ICU in a developing country, 10 beds. Inclusion criteria: Every patient admitted to ICU with positive fungal samples. Patients were followed until discharge from hospital. Microbiology samples were collected before the beginning of antifungal treatment and during the follow- up.

**Results:** 52 patients were included, 65.38 % were male. Mean Age 45.2 (SD +/−19.1), median Apache II score was 15, median SAPS II was 41, median ABSI was 7. 42.3 % were from rural areas and 28.85%were working during the injury, followed by home incidents 21.15 %. Fire was the most common cause found in 50 % of patients, 17.3 % had electrical burns and 17.3 % were in an explosion context. 1 patient suffered a chemical burn. Inhalation injury was present 57.7 %. Total burn surface area (TBSA) was 44.2 %: 40-60 %, 30 %:20 %-40 %, and 15.4 %:60-80 %. Mean stay in ICU 43.8 days (SD 52.5), median 28 days. *C. albicans* was the most commonly isolated species (sp) (34,6 %), **Candidemia was the most common diagnosed infection**. The sp isolated in blood cultures (BC) were: 23,1 % *C. albicans,* 21,1 % *C. haemulonii*, 14.4 % *C. parasilopsis.* We had 2 + BC for *Trichosporon asahii*. Catheter -related infection by *C. albicans, C. parasilopsis, and candida haemulonii* was diagnosed in 6 patients. Positive Urine samples were found mostly for C. albicans 11.5 %. The most frequently factors associated with fungal infection were: > than 7 days in the ICU 90.4 %, urinary catheter 90.4 %, broad- spectrum antibiotic exposure 88.5 %, indwelling central venous catheter (CVC) 82.7 %, feeding tube 82.7 %, total parenteral nutrition 63.5 %, Invasive mechanical ventilation 63.5 %. To highlight an association with *Acinetobacter baumannii* in 44.2 % of our patients. Doctors chose Fluconazole in 88.9 % as a first line of therapy. An antibiogram was performed and the susceptibility was confirmed. ICU mortality rate, 26.9 %.

**Conclusions:** In our environment, *C. albicans* continues to be the species that causes the largest number of invasive candidiasis. Prolonged stay in the ICU is an important risk factor to develop fungal infections. Even with the particular features of a burn patient, their complexity, and the negative impact of each infection; Fluconazole keeps having an important role in the treatment as a first line.

### A832 The effect of introduction of daily chlorhexidine bathing on healthcare-associated infections and acquisition of multi-drug resistant organisms

#### E. Ahmadnia^1^, J.S. Hadley^1,2^, M. Millar^3^, D. Hall^1^, H. Hewitt^1^

##### ^1^Adult Critical Care Unit, Royal London Hospital, Barts Health NHS Trust, London, United Kingdom; ^2^Queen Mary University of London, London, United Kingdom; ^3^Royal London Hospital, Barts Health NHS Trust, Microbiology Department, London, United Kingdom

###### **Correspondence:** E. Ahmadnia – Adult Critical Care Unit, Royal London Hospital, Barts Health NHS Trust, London, United Kingdom

**Introduction:** It has been suggested that daily bathing with chlorhexidine impregnated cloths may significantly reduce the acquisition of multi-drug resistant organisms (MDROs), incidence of central line associated bloodstream infections (CLABSIs), and the development of intensive care unit (ICU) acquired bloodstream infections [1]. However, more recent data have failed to support daily bathing of critically ill patients with chlorhexidine for these purposes [2].

**Objectives:** To determine if the implementation of a daily chlorhexidine bathing regimen affects acquisition rates of MDROs, the incidence of CLABSIs, and ICU bacteraemias.

**Methods:** A quality improvement project was conducted at a 44 bedded adult critical care unit within a UK University Hospital (incorporating major trauma, medical, and surgical patients). During the 1 year control period (December 2013 to November 2014), all patients were bathed using soap and water. During the subsequent intervention period (December 2014 to November 2015), all patients were bathed using 2 % chlorhexidine impregnated cloths (Clinell, GAMA Healthcare). The acquisition of MDROs, incidence of CLABSIs and ICU bacteraemias were recorded during these periods (6 months pre- and 6 months post-chlorhexidine for CLABSIs, one year for the other outcomes).

**Results:** The study covered 34317 patient bed days (16887 pre- and 17430 post-introduction of chlorhexidine bathing). There were an identical number of MDRO acquisitions in each group (290), giving rise to an MDRO acquisition rate per 1000 bed days of 17.17 in the control group compared to 16.63 in the chlorhexidine group (P = 0.70). CLABSI incidence per 1000 bed days was higher in the control group compared to the chlorhexidine group (9.51 vs 7.90; P = 0.26). The incidence of significant bacteraemias per 1000 bed days was similar in the the two groups (5.57 before and 5.45 during chlorhexidine bathing; P = 0.88), but the incidence of bacteraemias due to skin commensals per 1000 bed days was lower in the chlorhexidine group (7.22 vs 5.45; P = 0.04).

**Conclusions:** At our large University Hospital ICU with a heterogeneous patient population, the introduction of routine daily chlorhexidine-impregnated cloth bathing appears to significantly reduce the incidence of bacteraemias due to skin commensals and demonstrates a non-significant reduction in CLABSIs. Given the uncertainties surrounding diagnosis in the ICU, the effect seen may be of benefit in reducing the use of antibiotics to cover for these skin commensals - both in terms of antibiotic stewardship and health economics.

**References**

[1] Climo MW *et al*. Effect of daily chlorhexidine bathing on hospital-acquired infection. *N Engl J Med.* 2013;368(6):533–542.

[2] Noto MJ *et al*. Chlorhexidine bathing and health care-associated infections: a randomized clinical trial. *JAMA.* 2015;313(4):369–378.

**Grant acknowledgement**

Clinell (GAMA Healthcare) provided the first 6 months of chlorhexidine cloths without charge.

### A833 Comparison of the efficacy of three cutaneous antiseptic solutions for preventing catheter colonization: a multicenter, prospective, open-label, parallel, randomized controlled study

#### H. Yasuda^1,2^, M. Sanui^3^, T. Komuro^4^, S. Kawano^4^, K. Andoh^4^, H. Yamamoto^4^, E. Noda^4^, J. Hatakeyama^4^, N. Saitou^4^, H. Okamoto^4^, A. Kobayashi^4^, T. Takei^4^, S. Matsukubo^4^, JSEPTIC (Japanese Society of Education for Physicians and Trainees in Intensive Care) Clinical Trial Group

##### ^1^Japanese Red Cross Musashino Hospital, Emergency And Critical Care Medicine, Tokyo, Japan; ^2^Kameda Medical Center, Intensive Care Medicine, Chiba, Japan; ^3^Saitama Medical Center, Jichi Medical University, Department of Anesthesiology and Critical Care Medicine, Saitama, Japan; ^4^Japanese Society of Education for Physicians and Trainees in Intensive Care, Tokyo, Japan

###### **Correspondence:** H. Yasuda – Japanese Red Cross Musashino Hospital, Emergency And Critical Care Medicine, Tokyo, Japan

**Introduction:** The current CDC guideline published in 2011 for the prevention of intravascular catheter-related infections recommends skin preparation with a greater than 0.5 % chlorhexidine with alcohol solution before CVCs or ACs placement and with dressing changes, which was changed from 2 % chlorhexidine recommended in the 2002 guideline. However, few studies investigated the superiority of 1 % CHG over either 0.5 % CHG or 10 % PVI for the prevention of catheter colonization as CDC guideline recommends.

**Objectives:** Efficacy comparison of three antiseptic solutions [10 % aqueous povidone-iodine (PVI), and 0.5 % and 1.0 % alcoholic chlorhexidine gluconate (CHG)] for preventing intravascular catheter colonization.

**Methods:** This was a open-label, multicenter, prospective, randomized controlled trial conducted at 15 ICUs in Japan. The intravascular catheters included central venous catheters (CVCs) and arterial catheters (ACs). Patients aged >18 years of age undergoing CVC and AC insertion in ICU were randomized to receive one of three antiseptic preparations pre-insertion. Catheters were removed when no longer necessary or if catheter-related infection was suspected. After catheter removal, distal tips were cultured using semi-quantitative/quantitative techniques. Catheter colonization and catheter-related bloodstream infection (CRBSI) incidences were compared.

**Results:** While a total of 1132 catheters were randomized, several catheters were excluded due to withdraw of their informed consent and lack of cultured catheters after randomization, and 796 (70 %) catheters were included in the full analysis (0.5 % CHG n = 261, 1.0 % CHG n = 278, and 10 % PVI n = 257). The median catheterization duration was 3.8 days (95 % CI: 2.0-6.7 days); no significant intergroup differences were observed (p = 0.43). Catheter-tip colonization incidence (per 1000 catheter days) was 10.5, 3.9, and 3.7 events in 10 % PVI, 1 % CHG, and 0.5 % CHG groups, respectively (p = 0.04). Catheter colonization risk was significantly higher in the 10 % PVI group. No significant intergroup differences CRBSI probability were observed (3.2 vs. 2.0 vs. 4.9 per 1000 catheter days, p = 0.42).

**Conclusions:** In this multicenter prospective randomized controlled trial comparing the effectiveness of three cutaneous antiseptic solutions for the prevention of catheter colonization, either 0.5 % or 1.0 % CHG was superior to 10 % PVI.

**Fig. 12 (abstract A833).**
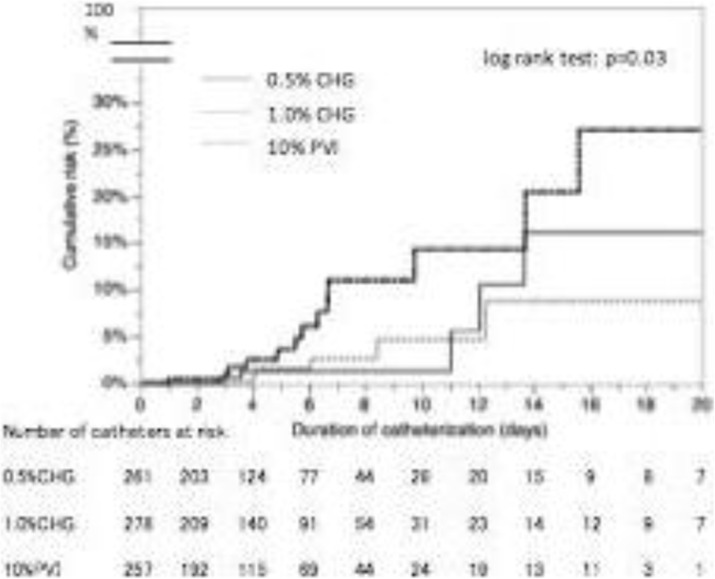
Catheter colonization

## OUTCOMES IN NEUROINTENSIVE CARE

### A834 Hematoma volume and location as prognostic factors for clinical outcome in patients with spontaneous intracerebral hemorrhage

#### H.B. Rotzel, A. Serrano Lázaro, D. Aguillón Prada, M. Rodriguez Gimillo, O. Diaz Barinas, M.L. Blasco Cortes, J. Ferreres Franco, J.M. Segura Roca, A. Carratalá

##### Hospital Clinic Universitari Valencia, Valencia, Spain

###### **Correspondence:** H.B. Rotzel – Hospital Clinic Universitari Valencia, Valencia, Spain

**Introduction:** Spontaneous intracerebral hemorrhage (ICH) is the most fatal stroke subtype worldwide caused by spontaneous vascular rupture due to hypertension or amyloid angiopathy. An accurate prediction of ICH outcome would assist both families and physicians to decide therapies and monitorization at an early stage.

**Objectives:** To evaluate the relationship between the hematoma volume and location with mortality and functional outcome in patients with spontaneous ICH.

**Methods:** We performed a prospective observational study, included patients admitted in ICU with spontaneous ICH. We determined hematoma volume at admission with Kothari modified formula (AxBxC/2) and divided them in two groups according the location as infratentorial or supratentorial. We collected GCS, SOFA, APACHE II and GRAEB at admission, medical history and complications during the first week in the ICU. We established modified Rankin Scale (mRS: poor outcome >2) and Glasgow Outcome Scale (GOS, poor outcome < 4) at ICU discharge. We used %, mean (SD) and median (minimal/maximum). T-Student and χ2 (p < 0.05) were used for the univariable analysis. We conducted a multivariable analysis for mortality with binary logistic regression (95 % CI, OR) p < 0,05. ROC curve was determined for the volume of hematoma associated with mortality (IC 95 % p < 0.05).

**Results:** We enrolled 120 patients. 68 % were men, mean age 62 (±12.6) years. Global mortality was 34.2 %. 83.3 % were supratentorial and 16.7 % infratentorial. Mean APACHE II 14 (±6, 5) and GCS 10.4 (±4,1) and median SOFA 4 (0–14) and hematoma volume 21,35 cc^3^ (1–252). There were no significant differences between the two groups (infra and supratentorial) except ICH volume (p 0.000) and length of stay (LOS)-ICU (p 0.021). In the univariable analysis worse outcome with mRS was related with the volume of the hematoma (p 0,03) but not with GOS (p 0,29). Variables associated with mortality: GCS (p 0.000), APACHE II (p 0.000), GRAEB (p 0.001), SOFA (p 0.000), LOS-ICU (p 0.004) and ICH volume (p 0.000). After the multivariable analysis we determined hematoma volume was an independent risk factor for mortality (OR 1,032; 95 % CI 1,019-1,046; p 0.000). According the location we obtained a significantly association with mortality in the supratentorial group (p 0,000). We performed a ROC curve of this group and obtained an AUC 0,718 (95 % CI 0,603-0,834; p 0.000) with cutoff point of 20,2 cc^3^.

**Conclusions:** Hematoma volume and LOS-ICU are greater in supratentorial ICH. The hematoma volume is associated with a worse outcome at ICU discharge and a supratentorial ICH volume above 20.2 cc^3^ is related to higher risk of mortality.

**References:** Guidelines for the Management of Spontaneous Intracerebral Hemorrhage. AHA/ASA Guidelines. June 2015.

**Fig. 13 (abstract A834).**
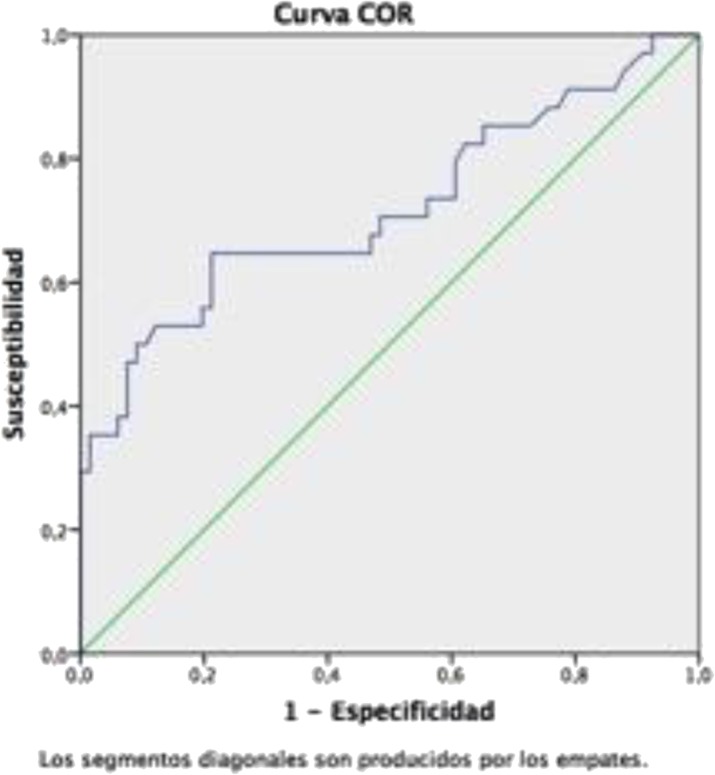
ROC curve for supratentorial ICH

### A835 Subarachnoid hemorrhage in a high volume center in Rio de Janeiro - neurological and clinical complications associated with unfavorable outcomes

#### B. Gonçalves, R. Turon, A. Mendes, F. Miranda, P.J. Mata, D. Cavalcanti, N. Melo, P. Lacerda, P. Kurtz, C. Righy

##### Paulo Niemeyer State Brain Institute, Rio de Janeiro, Brazil

###### **Correspondence:** B. Gonçalves – Paulo Niemeyer State Brain Institute, Rio de Janeiro, Brazil

**Introduction:** Aneurysmal subarachnoid hemorrhage (SAH) is an acute cerebrovascular event, which leads to devastating consequences, high mortality and is an important cause of neurologic disability among survivors. Incidence is reported between 2 to 16/100000 and mortality rates vary widely, ranging from 8 to 67 % among different authors. Many complications associated with SAH, such as delayed cerebral ischemia or hydrocephalus, also play a role in the poor functional outcome in survivors. Paulo Niemeyer State Brain Institute is a reference and high-volume center for SAH, located in Rio de Janeiro, Brazil, receiving patients from all over the state.

**Objectives:** The aim of the study was to describe the characteristics of patients with SAH admitted to the ICU, as part of a large prospective ongoing study, and to evaluate the factors associated with outcome.

**Methods:** From July 2015 to March 2016, every patient admitted to the ICU with aneurysmal SAH, 18 years and older was enrolled in the study. Data were collected prospectively during hospital stay. The primary endpoint was mortality and dichotomized functional outcome, (poor outcome defined as Modified Rankin Scale 4–6) at hospital discharge.

**Results:** A total of 53 patients were included. The median age was 56 (47–66), 43 patients (81 %) were female. Demographic characteristics are presented in Tables 8 and 9. Twenty-nine patients (55 %) were treated by clipping, and 13 patients (25 %) were hydrocephalic and needed an EVD. An intracranial pressure monitor was inserted in 11 patients (21 %). Nine patients (17 %) developed sepsis or septic shock during ICU stay and pneumonia was present in 14 (26 %) patients. Rebleeding was diagnosed in 8 patients (15 %), vasospasm was present in 17 (32 %) patients, post-surgical deterioration was diagnosed in 16 (30 %) patients and 13 (25 %) patients developed DCI. Twenty-two (42 %) patients were mechanically ventilated. Hospital mortality was 11 % (6 patients); and 26 patients had unfavorable (49 %). In univariate analysis, factors most frequently seen in patients with unfavorable outcome were rebleeding (69 % vs 0 %, p = 0.002), vasospasm (46 % vs 19 %, p = 0.031), post-surgical neurological deterioration (46 % vs 15 %, p = 0.013), DCI (42 % vs 7 %, p = 0.003) and pneumonia (42 % vs 11 %, p = 0.01). Although not statistically significant, there was a trend towards the association between sepsis/septic shock (27 % vs 7 %, p = 0.076) and unfavorable outcome.

**Conclusions:** SAH is associated with high morbidity. Neurological complications such as rebleeding, vasospasm, post-surgical neurological deterioration and DCI, as well as clinical complications (eg. pneumonia) were associated with unfavorable outcomes. Therapeutic interventions to prevent neurological and systemic complications may have an impact on clinical outcomes.

**Table 8 (abstract A835).** Demographic characteristics

Variable	All patients (n = 53)
Female Gender	43 (81 %)
Age	56 (47–66)
Treatment	
Clipping	29 (55 %)
Coil	22 (42 %)
Mechanical Ventilation	22 (42 %)
Sepsis/Septic Shock	9 (17 %)
Hospital Mortality	6 (11 %)
Unfavorable Outcome	26 (49 %)

**Table 9 (abstract A835).** SAH Scores on Admisson

Admission WFNS - 1	24 (45 %)
2	12 (23 %)
3	6 (11 %)
4	7 (13 %)
5	3 (6 %)
Admission Modified Fisher Scale - 0	6 (11 %)
1	11 (21 %)
2	7 (13 %)
3	16 (30 %)
4	12 (23 %)

### A836 Patient’s course into the icu after been submitted to a CNS tumor resection

#### L.E. de la Cruz Rosario^1^, S.P. Gómez Lesmes^1^, J.C. García Romero^2^, A.N. García Herrera^1^, E.D. Díaz Pertuz^3^, M.J. Gómez Sánchez^1^, E. Regidor Sanz^1^, J. Barado Hualde^1^, A. Ansotegui Hernández^1^, J.M. Guergué Irazabal^1^

##### ^1^Complejo Hospitalario de Navarra, Intensive Care Medicine, Pamplona, Navarra, Spain; ^2^Complejo Hospitalario de Navarra, Neurosurgery, Pamplona, Navarra, Spain; ^3^Complejo Hospitalario de Navarra, Neurology, Pamplona, Navarra, Spain

###### **Correspondence:** L.E. de la Cruz Rosario – Complejo Hospitalario de Navarra, Intensive Care Medicine, Pamplona, Navarra, Spain

**Introduction:** The management of patient into the ICU after been submitted to a CNS resection is an important challenge. Surgery is indicated for diagnosis, to reduce tumor bulk and to manage raised intracranial pressure. Primary brain tumors are classified based on their cellular origin and histologic appearance. The most common malignant brain tumor is glioblastoma multiforme, this group have a poor prognosis.

**Objectives:** The goal was to make a descriptive analysis about the evolution of patient submitted in the ICU for postoperative control following a surgical resection of intracranial tumors.

**Methods:** A retrospective and observational study was conducted on all elective consecutive surgical procedures for tumor resection admitted into the ICU. We analyzed variables related with the tumor, predisposing pathology, surgical data and evolution in the ICU. We considered as an unfavorable evolution the death into the first month after the intervention or the decrease in two points or more of the Canadian´s scale score (CSS). Is a comparative study analyzed by Student´s t-test, ANOVA of one factor and Pearson´s chi-square test. Comparative study expressed by: mean difference, relative risk and confidence intervals at 95 %.

**Results:** We analyzed 271 patients over of 5 years (2011–2015). Of the total, 31.3 % are high-grade gliomas, 9.6 % low grade gliomas, 32,7 % meningiomas , 8.5 % metastasis and 14.2 % other type of tumors. Average age is 54.6 years (SD 14.8), it is significantly lower in the low-grade gliomas, and in the group of other tumor types compared to other groups. 50.6 % are men , the most common in men (63.5 %) and meningiomas and other tumors in women (60.9 % and 62.5 % respectively) gliomas. 88.7 % are supratentorial location. Average size is 29.9 mL (SD 28.2) . The average score in the preoperative Karnofsky scale is 73.2 (SD 12.9) . The average income APACHE is 6.9 points (SD 4.5). An unfavorable evolution is observed in 14.0 % of patients (6.6 % per patient died and 7.4 % decline in the CSS) after one month , with no differences between different types of tumors. The percentage of deaths in the first month is higher in those undergoing surgery for metastasis (20.8 %, RR 4.0, CI 1.5 to 10.2). Mortality at two years of intervention is 31.5 %, being higher in sifnificativamente undergoing metastasis (75 %; relative risk 5.7, CI 3.6 to 9.0) and high-grade gliomas (63.6 %; relative risk 4.6, CI 3.1 to 7.4).

**Conclusions:** Patients undergoing brain tumors have a significant risk of poor outcome , which is significantly higher in metastatic patients from the first month of intervention and in patients undergoing high-grade gliomas at two years.

**References**

1. Outcome of elderly patients undergoing intracranial meningioma resection - a systematic review and meta-analysis.M. Tin-Chung Poon1 ,2, L. Hing-Kai Fung1 , J. K.-Suen Pu1 & G. Ka-Kit Leung1*BJN*, 2013; Early Online: 1–7

### A837 Preventive multimodal nosocomial infection protocol in neurocritical care

#### V. Spatenkova^1^, O. Bradac^2^, P. Suchomel^3^

##### ^1^Regional Hospital, Neurocenter, Neurointensive Care Unit, Liberec, Czech Republic; ^2^Military University Hospital and First Medical School, Charles University, Department of Neurosurgery, Prague, Czech Republic; ^3^Regional Hospital, Neurocenter, Department of Neurosurgery, Liberec, Czech Republic

###### **Correspondence:** V. Spatenkova – Regional Hospital, Neurocenter, Neurointensive Care Unit, Liberec, Czech Republic

**Introduction:** Nosocomial infection (NI) is still an issue in neuroritical care.

**Objectives:** We analysed NI in a preventive multimodal protocol in patients with acute brain disease.

**Method:** We performed a 10-year prospective observational cohort study in 3464 patients (pts) with acute brain disease admitted to an eight-bed adult neuro-intensive care unit (NICU). We defined our preventive multimodal protocol as: 1) keeping a hygienic and epidemiological regime including isolation of pts with multi-drug resistant bacteria 2) correct antibiotic policy, and 3) regular microbiological screening. There were 198 (5.7 %; wound 2.1 %, respiratory 1.8 %, urinary 1.0 %, bloodstream 0.6 % and other 0.2 %) pts with NI. We compared NI group pts with the control group of 3266 pts and searching predictors of NI in univariete analysis.

**Results:** We did not find differences in age (p = 0.416), male (p = 0.716), weight (p = 0.423) or body mass index (p = 0.966), but there were more stroke pts and fewer tumour pts (p < 0.001). NI pts stayed in NICU longer (mean 15.3 vs 4.8, p < 0.001), on admission had lower Glasgow Coma Scale (mean 11.5 vs 13.1, p < 0.001), higher Therapeutic Intervention *Scoring System* (TISS, p < 0.001), Acute Physiology and Chronic Health Evaluation II (p < 0.001), and CRP (p < 0.001); in the NICU they had higher CRP (p < 0.001) and NICU mortality (p < 0.001); on discharge they had worse Glasgow Outcome Scale (p < 0.001) and higher TISS sums (p < 0.001). NI pts had more accesses, which were strong predictors of NI: artery (odds ratio [OR] 3.68, 95 % CI 2.65-5.11, p < 0.001), central venous (OR 4.97, 95 % CI 3.49-7.07, p < 0.001), airways (OR 7.40, 95 % CI 5.27-10.39, p < 0.001), artificial ventilation (OR 6.74, 95 % CI 4.84-9.40, p < 0.001), urine (OR 4.23, 95 % CI 1.56-11.50, p = 0.005), operations (OR 1.65, 95 % CI 1.14-2.39, p = 0.008), drainage (OR 2.42, 95 % CI 1.71-3.42, p < 0.001). Other predictors were transfusions (OR 6.97, 95 % CI 4.62-10.50, p < 0.001), ulcer profylaxis (OR 2.12, 95 % CI 1.49-3.02, p < 0.001), wound complications (OR 7.21, 95 % CI 4.60-11.30, p < 0.001) and *Methicillin-resistant Staphylococcus aureus* (OR 2.90, 95 % CI 1.22-6.89, p = 0.016) to contrast *Extended spectrum beta-lactamase (*p = 0.227).

**Conclusions:** Our study confirmed that nosocomial infection is associated with worse outcome and higher cost, and that accesses are still risk factors in a preventive multimodal protocol.

### A838 The predictive value of emergency triage codes on the outcome of aneurysmal subarachnoid hemorrhage

#### T. Urli^1^, E. Heusch Lazzeri^2^, R. Aspide^1^, M. Zanello^1,2^

##### ^1^IRCCS Istituto delle Scienze Neurologiche, Anesthesia and Intensive Care, Bellaria Hospital, Bologna, Italy; ^2^University of Bologna, Bologna, Italy

###### **Correspondence:** R. Aspide – IRCCS Istituto delle Scienze Neurologiche, Anesthesia and Intensive Care, Bellaria Hospital, Bologna, Italy

**Introduction:** Outcome of patients with aneurysmal subarachnoid hemorrhage (SAH) was associated in different studies with different variables (baseline illness severity, physical status, treatments, complications), but the relationship between outcome and triage assessment in the emergency setting has never been evaluated. Emergency triage in Italy is carried out with color codes: red (immediate life-saving intervention needed), yellow (urgent intervention needed), green (delayed intervention is sufficient), white (not urgent).

**Objectives:** To study the relationship between triage severity codes assigned to patients with SAH in an Italian emergency setting and the outcome expressed as modified Rankin Score (mRS) at hospital discharge (good outcome for mRS ≤ 3, poor outcome for mRS > 3).

**Methods:** A retrospective clinical study included 52 patients with aneurysmatic SAH admitted to emergency departments of Bologna catchment area, and then to intensive care unit (ICU), from January 2014 to January 2015. Aneurysm coiling or clipping was performed after neuroradiological diagnosis and clinical stabilization, excluding patients too ill to benefit. Intensive care treatment was carried out according to current practical guidelines. Demographic, clinical and interventional data, complications, severity scores and outcome scores were recorded. The following parameters were considered in univariate analysis: age, sex, clinical condition on arrival in the emergency department (triage code, GCS, WFSN scale, vomiting and seizures) aneurysm clipping or coiling and other neurosurgical interventions, hydrocephalus, vasospasm, cerebral infarction (CT scan), fever, sepsis, acute respiratory failure with P/F ≤ 200, cardiovascular complications (hypotension requiring vasopressor therapy, acute cardiomyopathy, arrhythmias requiring treatment); the outcome variable was modified Rankin Score >3 at hospital discharge.

**Results:** Poor outcome (mRS > 3) was observed in 33 % of triage green codes, 53 % of yellow codes, 75 % of red codes. The univariate analysis showed the statistically significant (p < 0.05) association with mRS > 3 for the following variables: triage red code, WFSN scale >2, acute respiratory failure, cardiovascular complications, sepsis. On logistic regression analysis, the red code assigned in the emergency department, cardiovascular complications and sepsis were associated with poor outcome.

**Conclusions:** The severity of general clinical conditions after subarachnoid hemorrhage needing immediate life-saving intervention, feature labelled “red code” in the emergency triage, was associated with poor outcome (mRS > 3), while the other triage codes did not show any significant correlation with outcome. Cardiovascular complications and sepsis during hospital stay were other variables associated with mRS > 3.

### A839 Evaluation of intracerebral hemorrhage (ICH) score in patients admitted in intensive care by supratentorial brain hemorrhage

#### L. Perez-Borrero^1^, J.M. Garcia-Alvarez^1^, M.D. Arias-Verdu^2^, E. Aguilar-Alonso^3^, R. Rivera-Fernandez^1^, J. Mora-Ordoñez^2^, C. De La Fuente-Martos^3^, E. Castillo-Lorente^4^, F. Guerrero-Lopez^5^

##### ^1^Hospital Serrania, Ronda, Spain; ^2^Hospital Regional, Intensive Care, Malaga, Spain; ^3^Hospital Infanta Margarita, Intensive Care, Cabra, Spain; ^4^Hospital Neurotraumatologico, Jaen, Spain; ^5^Hospital Virgen de las Nieves, Intensive Care, Granada, Spain

###### **Correspondence:** L. Perez-Borrero – Hospital Serrania, Ronda, Spain

**Introduction:** Intracerebral hemorrhage is a stroke subtype with high mortality and significant disability among survivors.

**Objective:** To evaluate in our area the Intracerebral Hemorrage (ICH) score in patients with spontaneous supratentorial brain hemorrhage.

**Methods:** Multicenter prospective observational study in three hospitals in Andalusia (Spain). We studied all patients with supratentorial brain hemorrhage admitted to the Regional Hospital of Malaga (between 2006 to 2011), Neurotraumatology Hospital of Jaen (between 2010 to 2012) and Virgen de las Nieves Hospital of Granada (between 2006 to 2011). ICH score was used: Glasgow Scale (GCS) (13–15: 0, 5–12: 1: 3–4: 2 points), age (<80: 0,> 80: 1 point), volume (<30 cc : 0,> 30 cc: 1 point), infratentorial location (No: 0, Yes: 1 point), intraventricular: (No: 0 Yes: 1 point). Data are expressed as mean ± standard deviation and percentage. Student t test to compare means, the Hosmer-Lemeshow to analyze the correlation between predicted and observed mortality and the area under the ROC curve for analyzing discrimination.

**Results:** N = 263 patients. Mean age 59.74 ± 14.14 years, Glasgow score (GCS) at admission 8 ± 4 points , APACHE-II 20.7 ± 7.68 points, ICH score 2.32 ± 1.04 points, intraventricular hemorrhage (IVH) was 62 % of patients. 78 patients were treated by surgery. The hospital mortality was 53.2 %. Patients who died in hospital were older 63.76 ± 12.27 vs 55.16 ± 14.76 (p < 0.001), lower GCS 6 ± 3. vs 10 ± 4 (p < 0.001) and higher APACHE II 23.91 ± 6.61 vs 17.06 ± 7.18 (p < 0.001). Mortality at 30 days was 51.3 % and predicted by the ICH score was 45.4 %. The standardized mortality ratio (SMR) was 1.13 (0.94-1.32) (the differences are not statistically significant).

The Hosmer-Lemeshow test was 39.8 (p < 0.001), so there were statistically significant differences between the observed and predicted by the ICH. Discrimination by the area under the ROC curve was 0.74 (0.68-0.80).

**Conclusions:** Patients admitted to ICU with spontaneous supratentorial brain hemorrhage have a high mortality. The ICH score has an acceptable discrimination. The calibration is inadequate, but the differences between predicted and observed mortality are low.

**Table 10 (abstract A839).** Mortalities according ICH score

ICH score	N	Predicted mortality at 30 days (%)	Observed mortality (%)
0	10	10 %	0 %
1	43	18.6 %	13 %
2	101	45.5 %	26 %
3	74	67.6 %	72 %
4	33	84.8 %	97 %
5	2	100 %	100 %

### A840 Predictors of mortality of spontaneous subarachnoid hemorrhage in patients admitted to an intensive care unit of a referral hospital

#### S.P. Gómez Lesmes^1^, L.E. De la Cruz Rosario^1^, E.D. Díaz Pertuz^2^, A. Ansotegui Hernández^1^, J.C. García Romero^3^, M.J. Gómez Sánchez^1^, A.N. García Herrera^1^, J. Roldán Ramírez^1^, E. Regidor Sanz^1^, J. Barado Hualde^1^, J.P. Tirapu León^1^

##### ^1^Complejo Hospitalario de Navarra, Intensive Care Medicine, Pamplona, Spain; ^2^Complejo Hospitalario de Navarra, Neurology, Pamplona, Spain; ^3^Complejo Hospitalario de Navarra, Neurosurgery, Pamplona, Spain

###### **Correspondence:** S.P. Gómez Lesmes – Complejo Hospitalario de Navarra, Intensive Care Medicine, Pamplona, Spain

**Introduction:** Spontaneous Subarachnoid haemorrhage (SSAH) accounts for only 5 % of strokes.Aneurysms are the cause of subarachnoid haemorrhage in 85 % of cases.Rebleeding is the most imminent danger. Other complications are vasospasm, delayed cerebral ischaemia and hydrocephalus.

**Objectives:** To know mortality predictors of spontaneous subarachnoid hemorrhage (SSAH) in the intensive care unit at the Hospital of Navarra in a period of 10 years.

**Methods:** Analytical retrospective study was carried out of patients admitted to the ICU at the hospital of Navarra between June 2004 and June 2014, with spontaneous subarachnoid hemorrhage diagnosis. Demographic , clinical , treatments and complications variables were studied with mortality at ICU discharge. Univariate analysis (Chi square test or Fisher where appropriate and T of Student) and multivariate logistic regression were used to identify independent predictors of mortality.

**Results:** A total of 169 patients enrolled. Mortality was 36.1 %, of which 62.3 % were women. Average stay of patients who died was 7.34 days (4.23 to 10.46 days) and alives were 13.31 days (10.45 to 16.28 days), p = 0.006. In the univariate analysis, the variables were statistically related mortality: Glasgow coma score at admission (<9 vs > = 9) OR = 5.04 (95 % CI = 2.55 to 9.98), *P* = < 0.001; Fisher scale (I-II vs III-IV ), OR *P* = 0.042; Hunt & Hess scale (I, II, III vs IV, V), OR = 5.53 (95 % CI = 2.73 to 11.19), *P* = < 0.001; aneurysm endovascular embolization, OR = 0.18 (95 % CI = 0.089 to 0.36), *P* < 0.001; treatment with intravenous nimodipine, OR = 0.15 (95 % CI = 0.07 to 0.36), *P* = < 0.001; vasoactive treatment, OR = 6.15 (95 % CI = 2.83 to 13.35), P < 0.001; invasive mechanical ventilation, OR = 4.76 (95 % CI = 1.05 to 21.56), *P* = 0.028; presence of rebleeding, OR = 2.48 (95 % CI = 1.23 to 5.01), P = 0.010; vasospasm, OR = 0.409 (95 % CI = 0.17 to 1.01), *P* = 0.047; hydrocephalus, OR = 0.49 (95 % CI = 0.25 to 0.94), *P* = 0.030: respiratory infection, OR 0.5 (95 % CI = 0.26 to 0.99), *P* = 0.044; intracranial hypertension, OR = 3.57 (95 % CI = 1.62 to 7.84), *P* = 0.001.

Predictors of mortality: age, OR 1.06 (95 % CI 1.03 to 1.10), *P* = 0.001; rebleeding, OR 5.51 (95 % CI 1.97 to 15.38), *P* = 0.001; intracranial hypertension, OR 6.20 (95 % CI = 2.06 to 18.65), *P* = 0.001 and Glasgow on admission (<9 vs > = 9), OR = 6.83 (95 % CI 2.70 to 17, 30), *P* = 0.001). Protective variables included embolization, OR 0.10 (95 % CI 0.04 to 0.26), *P* < 0.001 and placement of an external ventricular shunt, OR 0.36 (95 % CI = 0.14 to 0.91), *P* = 0.032.

**Conclusions:** Poor prognostic factors for mortality independently were older age, coma on admission, rebleeding and development of intracranial hypertension in evolution. As protective factors for mortality include embolization and placement of an external ventricular shunt.

**References:**

1. Subarachnoid haemorrhage. Van Gijn J, Kerr RS, Rinkel GJ. Lancet. 2007 Jan 27; 369(9558):306–18.

### A841 Preventable and potentially preventable traumatic mortality in a neurotraumatologic ICU

#### L. Navarro-Guillamón, S. Cordovilla-Guardia, A. Iglesias-Santiago, F. Guerrero-López, E. Fernández-Mondéjar

##### Complejo Hospitalario de Granada, Granada, Spain

###### **Correspondence:** L. Navarro-Guillamón – Complejo Hospitalario de Granada, Granada, Spain

**Objective:** The reduction in mortality is a basic principle of health systems. Our goal is to quantify preventable and or potentially preventable traumatic mortality and analyze the mistakes that have contributed to the result to know our room for improvement.

**Methods:** A retrospective, descriptive study of patients admitted to the ICU from January 2013 to December 2015 and who died in the hospital (both ICU and ward). It was considered as preventable mortality what occurs as a result of a potentially avoidable diagnostic or therapeutic mistake. Potencially preventable mortality was considered when a mistake was identified but death could have occurred even without him. And unavoidable mortality when it is as a result of extremely serious injuries incompatile with survival.

Quantitative variables were described by median and interquartile range and qualitative variables as percentages.

**Results:** 365 patients were admitted to the ICU, dying 73 of them (20 %). Focusing on the death described: Age: 69 years [47–76], ISS: 27 [25–38] males 71.2 % (52 patients). From emergency room came 67.1 % (49) and transfer from another hospital 33 % (24). Cause of Death: neurologic in 41 % (30), multiorgan failure 30 % (22). In 20 patients (27.4 %) Limitation of treatment was decided.

Of the 73 dead patients, 7 of them were classified as potentially preventable deaths and one preventable death (11 %) which represents 2.19 % of total patients.

The detected errors associated with preventable or potentially preventable mortality were delayed correct interpretation of signs and symptoms (5 cases), delayed implementation of treatment (4 cases), incorrect treatment (2 case) and transfer for incorrect unit (1 case)

**Conclusions:** The management of severe trauma is not free of mistakes that can be fatal, so training in this field must be maintained continuously. However, the room for improvement in this area is limited, therefore, the main efforts should be foccused towards prevention (primary and secondary).

### A842 Have SAH prognostic factors changed in the era of endovascular intervention?

#### A. Vidal^1^, M. Perez^2^, A. Juez^2^, N. Arias^1^, L. Colino^1^, J.L. Perez^1^, H. Pérez^1^, P. Calpe^1^, M.A. Alcala^1^, D. Robaglia^1^, C. Perez^1^

##### ^1^Fundación Jiménez Díaz, Madrid, Spain; ^2^Hospital Rey Juan Carlos, Móstoles, Spain

###### **Correspondence:** A. Vidal – Fundación Jiménez Díaz, Madrid, Spain, ^2^Hospital Rey Juan Carlos, Móstoles, Spain

**Introduction:** Within the clinical importance of the SAH, there are factors described in the scientific literature that speak of an unfavorable evolution of the disease. Our hypothesis is based on trying to demonstrate if only one therapeutic intervention could alter the significance of these factors.

**Objectives:** Analyze the sociodemographic, laboratory findings, clinical and radiological factors that influence prognosis at 6 months in discharged aneurysmal SAH patients treated with endovascular intervention.

**Methods:** We performed a retrospective longitudinal observational study of all patients who were diagnosed with an aneurysmal SAH in ICU services of 2 hospitals between March 1st 2012 and November 10th 2015. They were treated by endovascular intervention.

After being discharged from ICU and after 6 months of neurologic follow-up. Patients were divided into two groups, one formed by those who presented a favorable outcome (EF) and the other by those who didn´t (ED).

The variables studied were age, sex, HBP, DM, smoking and dyslipidemia. At the time of admission PO2, PCO2, leukocytosis, hyperglycemia and hypertension was determined as well as sodium, magnesium and chlorine plasma levels.

The clinical status of patients on admission was assessed using the Hunt-Hess and WFNS scales. The severity of SAH was determined by CT using the Fischer scale. The aneurysm was located by four vessel angiography. The time between the SAH clinic presentation and the procedure was recorded, as well as if aneurysmal occlusion was complete or not. As for the complications, we took into account the presence of fever, hydrocephalus, vasospasm and infarction.

**Results:** For the study, 39 patients who underwent acute endovascular SAH treatment using coils, were selected.

Female sex was the predominant sex 80 % Vs 75.8 % between ED and EF, respectively. The age group most frequently found was between 45 and 65 years (60 % for ED and 62 % for EF).

Logistic regression analysis determined as associated with a worse outcome factor: hyperglycemia on admission(OR 8.94, 95 % CI 1.76-45.3, p = 0.007), clinical status on admission determinated by Hunt-Hess (OR 14.58 CI 2.62-81 95 %, p = 0.0018) and WFNS scales (OR 5.75, 95 % CI 1.21-27.13, p = 0.02). The presence of fever on admission also has proven to be a poor prognostic factor (OR 7.33 95 % CI 1.48-36, p = 0.01).

**Conclusions:** Clinical factors for aneurysmal SAH patients treated with endovascular procedure that have shown relation with the clinical outcome at six months are: poor clinical grade on admission, hyperglycemia and fever. These data are similar to those found in the literature and support the idea that the therapeutic decision (surgical or endovascular) is not the determining factor for the evolution of these patients, however, the ones mentioned above could be.

### A843 Increased risk of ischemic stroke in patients with venous thromboembolism: a nationwide cohort study

#### S.-K. Lan

##### Dalin Tzu Chi Hospital, Buddhist Tzu Chi Medical Foundation, Chiayi, Taiwan, Province of China

**Background:** Conflicting results have been obtained by studies attempting to assess the risks of ischemic stroke in patients with venous thromboembolism, while the long-term risk of stroke in survivors of venous thromboembolism remains unexplored.

**Objective:** We evaluated whether the risk of ischemic stroke in patients hospitalized with venous thromboembolism is higher when compared to the general population.

**Methods:** One million patients from National Health Insurance beneficiaries in Taiwan were sampled. There were 2,145 patients who had been hospitalized with diagnosis of venous thromboembolism and 727,607 unexposed subjects. All adult patients were followed from 1 January 2005 to 31 December 2013 to evaluate if ischemic stroke was diagnosed. Cox regression models were applied to compare the hazards adjusted for potential confounders.

**Results:** After controlling for age, gender, urbanization level, socioeconomic status, diabetes, hypertension, coronary artery disease, hyperlipidemia, history of alcohol intoxication, malignancies, congestive heart failure, atrial fibrillation, smoking, peripheral artery disease and Charlson Comorbidity Index, the adjusted hazard ratio of ischemic stroke was significantly increased in patients with venous thromboembolism (2.47; 95 % CI, 2.16-2.83). A subgroup analysis based on patients who survived longer than 12 months in the cohort also revealed higher hazard ratio in the patients with venous thromboembolism. (1.32; 95 % CI, 1.05-1.66).

**Conclusion:** The possible risk of ischemic stroke is significantly higher in patients hospitalized with venous thromboembolism than in the general population.

### A844 Stess as a predictor of status epilepticus' outcome

#### M.M. Cunha, T. Moreira, F. Santos, E. Lafuente, M.J. Fernandes, J.G. Silva

##### Centro Hospitalar Tâmega e Sousa, Intensive Care, Penafiel, Portugal

###### **Correspondence:** M.M. Cunha – Centro Hospitalar Tâmega e Sousa, Intensive Care, Penafiel, Portugal

**Introduction:** Status epilepticus (SE) is a common neurological emergency with considerable associated health-care costs, morbidity and mortality.^1,2^ SE is defined as a prolonged seizure or multiple seizures with incomplete return to baseline.^1,2^ The overall mortality of SE is around 20 % with convulsive status epilepticus representing about 45-74 % of all cases.^1^ Status Epilepticus Severity Score (STESS) is a prognostic score that relies on four outcome measures (age, history of seizures, seizure type and extent of consciousness impairment) determined before treatment institution that ranges between 0 and 6.^3^

**Objective:** Evaluation of STESS as a prognostic measure of functional impairment, neurologic motor deficits and 28-day mortality.

**Methods:** Retrospective observational study of patients with SE admitted at a general intensive care unit (ICU) from 2009 to 2015. Age, gender, SAPS II/III, type of SE, length of stay, number of anti-epileptic drugs, duration of SE, functional impairment, neurologic motor deficits and 28-day mortality were collected through the ICU informatics database - PICIS®. Data is presented as mean ± SD and we used logistic regression to correlate STESS with study variables. Statistical analysis was performed using XStat 2016®.

**Results:** Sample included 29 patients, 72,6 % male, age 46,6 ± 12,5 years, SAPS II 42,3 ± 13,1, SAPS III 55,7 ± 16,6, ICU length of stay 4,4 ± 2,6 days and hospital length of stay 16,2 ± 13,3 days. Convulsive SE represented 79,3 % of cases. STESS score`s mean was 2,6 ± 0,8. SE lasted more than 1 day in 48,3 %. Electroencephalogram was performed in 51,6 % of the patients. 48,1 % of the patients needed two or more anti-epileptic drug for SE. At hospital discharge 20,7 % had functional impairment and 13,8 % had neurologic motor deficits. Mortality was 6,8 % at 28 days. There was a correlation between STESS and mortality (OR = 10,4; ROC = 0,963), functional impairment (OR = 1,93; ROC = 0,667) and neurologic motor deficits (OR = 4,32; ROC = 0,820). The number of anti-epileptic drugs and SE duration had no correlation significance.

**Conclusions:** We found an excellent correlation between STESS and mortality in our study. Besides this, we also found this score to be a good prognostic tool for functional impairment and neurologic motor deficits. We consider our main limitations the sample size and lower mortality. Despite we recommend using STESS as an outcome predictor.

**References**

1- Lancet Neurol 2015; 14: 615–24; 2 - Epilesia 2015; 56(10):1515–1523; 3 - J Neurol 2008; 255:1561–1566.

### A845 Prognostic factors implicated in a good functional outcome one year after suffering a subarachnoid hemorrhaged

#### L.E. de la Cruz Rosario^1^, S.P. Gómez Lesmes^1^, A.N. García Herrera^1^, J.C. García Romero^2^, E.D. Díaz Pertuz^3^, M.J. Gómez Sánchez^1^, E. Regidor Sanz^1^, J.G. Armando Echeverría^1^, A. Ansotegui Hernández^1^, J. Barado Hualde^1^

##### ^1^Complejo Hospitalario de Navarra, Intensive Care Medicine, Pamplona, Navarra, Spain; ^2^Complejo Hospitalario de Navarra, Neurosurgery, Pamplona, Navarra, Spain; ^3^Complejo Hospitalario de Navarra, Neurology, Pamplona, Navarra, Spain

###### **Correspondence:** L.E. de la Cruz Rosario – Complejo Hospitalario de Navarra, Intensive Care Medicine, Pamplona, Navarra, Spain

**Introduction:** Aneurysmal subarachnoid hemorrhage (SAH) is a serious condition associated with high mortality rates and long-term disability. Prediction of outcome after spontaneous subarachnoid hemorrhage (SAH) lacks accuracy. We present some factors that can be associated with a good functional prognostic after one year of the event.

**Objectives:** Identify predictors of good functional outcome a year to present spontaneous subarachnoid hemorrhage (SSAH).

**Methods:** observational and retrospective study. SSAH admitted to the ICU in a period of ten year were analyzed. Association of demographic, clinical, therapeutic factors and complications, with good functional outcome a one year defined this as a Glasgow Outcome Scale (GOS) between 4 and 5. Poor prognosis was defined as GOS within 2 and 3.Statistics analysis by Chi square (Fisher test when appropriate) and Student T. The independent variables were analyzed by multivariate logistic regression.

**Results:** 97 patients survived after one year, 39.1 % with good functional prognosis. The mean ICU stay was 8.3 days (SD 11.8), mean age 52.4 years (SD 12.6) and poor prognosis 21 days (SD 18,2). Without diabetes mellitus (DM) 98.5 % (OR 0.080 (CI95% 0.009-0.72), P 0.012) and blood pressure (HBP) 74.2 % (OR 0.42 (CI95% 0.172-1 03), P 0.05). Hunt and Hess (I, II and III) and 72.7 % (IV and V) 27.3 % (OR 0.35 (CI95% 0.15-0.86), P 0.02); Fisher (I and II) and 18.2 % (III and IV) 81.8 %, GCS (3–8) and 18.2 % (9–15) 81.8 %. Embolized within 72 hours 83.3 %; external ventricular drainage device (EVD) 24.6 % (OR 0.27 (95 % CI 0.109-0.665), P 0.004); 9.1 % angioplasty; 1.5 % craniectomy (OR 0.104 (95 % CI from 0.01-0.97, P 0.018) Nimodipine perfusion in 89.4 % and 12.1 % during intra-arterial embolization; 40.9 % vasoactive treatment (OR 0,44 (95 % CI 0.18-0.05), P 0.061) and mechanical ventilation 81.8 %; 13.2 % complicated with re-bleeding, vasospasm 25.8 %, 39.4 % hydrocephalus (OR 0.36 (CI95% 0.15- 0.86, P 0.021), 4.5 % intracranial hypertension (OR 0.16 (CI95% 0.04-0.68), P 0.011); 7.6 % stroke (OR 0.2 (CI95% 0.061- 0.66), P 0.01), 6.1 % ventriculitis (OR 0.19 (CI95% 0.05-0.68 ), P 0.016) and 31.8 % respiratory infection (OR 0.34 (95 % CI 0.14-0.81), P 0.014). Using logistic regression, the following variables results but no one of these was statistical significant: DM (OR 0.54 (CI95% 0.005-0.634), P 0.02), hypertension (OR 0.32 (CI95% 0.1-1.0), P 0.05), EVD (OR 0.26 (CI95% 0.07-0.92), P 0.04), craniectomy (OR 0.03 (CI95% 0.003-0.34), P 0.005) and length of stay (OR 0.95 (IC95% 0.90 to 0.99), P 0.02).

**Conclusions:** The presence of comorbidities and the intervention by craniectomy and EVD significantly increase the likelihood of functional impairment annually.

**References**

1. Poor outcome is associated with less negative fluid balance in patients with aneurysmal subarachnoid hemorrhage treated with prophylactic vasopressor-induced hypertension.Sakr Y1,2, Dünisch P3, Santos C4, Ann Intensive Care. 2016 Dec;6(1):25

**Grant acknowledgement**

None

### A846 Prognostic possibility of neurological evaluation scale in patients after fossa posterior surgery

#### V. Podlepich, E. Sokolova, E. Alexandrova, K. Lapteva

##### Burdenko Neurosurgery Institute, Moscow, Russian Federation

###### **Correspondence:** V. Podlepich – Burdenko Neurosurgery Institute, Moscow, Russian Federation

**Introduction:** In order to determine optimal airway protection measures in early postoperative period after fossa posterior surgery (PFS), it is important to carry out a prognosis of neurological dynamics based on the preoperative neurological exam. We have designed Neurological Evaluation Scale (NES).

**Objectives:** Our study was aimed at determining the potential of NES to predict brain stem deterioration in early postoperative period after PFS based on the assessment of the preoperative neurological status.

**Methods:** The prospective study was carried out during the period from December 2013 to June 2014 and included 182 patients operated for fossa posterior tumors (FPT). To be included in the study, patients had to be over 18 years old and operated for fossa posterior non infiltrative paraxial tumors. We examined all patients before and after the operation, immediately after the extubation in ICU. NES provided complex neurological assessment with an emphasis on the brain stem function. Postoperative NES points were subtracted from the preoperative points - AB-criterion (ABc). Positive ABc corresponds to intensification of neurological deterioration. Negative or zero ABc corresponds to neurological improvement. All neurological symptoms were grouped in 7 NES blocks according to their relation to CNS.

**Results:** We divided all patients in two groups depending on their ABc, which revealed that the patients with positive ABc had reliability less NES points before operation compared to the patients with negative or zero ABc. We found out the frequency of occurrence of each NES block for inclusion in the full neurologic status. We discovered that caudal stem affection occurred more frequently in the patients with more NES points. We evaluated the probability of neurological impairment or regression of neurological symptoms depending on ABc with sensitivity 90,7 % and specificity 95 %. We created a prognostic model, which could predict the discharge from clinic outcome on the basis of the NES blocks points assigned during the early postoperative period.

**Conclusions:** We revealed neurological features of postoperative period in patient after FPS. Our data could predict neurological outcomes, and be useful in optimization tactic of airway protection.

**References**

1. Matsui T. Therapeutic strategy and long-term outcome of meningiomas located in the posterior cranial fossa. Neurol Med Chir (Tokyo). 2012;52(10):704–13.

2. Adachi K, Kazuhide Y. ABC Surgical Risk Scale for skull base meningioma: a new scoring system for predicting the extent of tumor removal and neurological outcome. J Neurosurg. 2009 Nov;111(5):1053–61

3. Wijdicks EF , Bamlet WR, Maramattom BV, Manno EM, McClelland RL. Validation of a new coma scale: The FOUR score. Ann Neurol. 2005 Oct; 58(4):585–93.

4. Harner SG, Laws ER Jr.Clinical findings in patients with acoustic neurinoma. Mayo Clin Proc. 1983 Nov;58(11):721–8.

### A847 Brain dysfunction in severe sepsis: an observational study

#### P. Kurtz^1,2^, C. Shuinotsuka^3^, L. Rabello^3^, G. Vianna^3^, A. Reis^3^, C. Cairus^3^, J. Salluh^3^, F. Bozza^3^

##### ^1^Fiocruz, Rio de Janeiro, Brazil; ^2^Paulo Niemeyer State Brain Institute, Rio de Janeiro, Brazil; ^3^D’Or Institute for Research and Education, Rio de Janeiro, Brazil

###### **Correspondence:** P. Kurtz – Fiocruz, Rio de Janeiro, Brazil

**Introduction:** Sepsis associated encephalopathy is a predictor of increased mortality and cognitive impairment in intensive care unit (ICU) patients. Electroencephalogram (EEG) abnormalities are common in the acute stages of sepsis and may correlate with the severity of systemic and brain dysfunction.

**Objectives:** The objective of this study was to establish the relationship between continuous EEG abnormalities, organ failure and mortality in patients with severe sepsis and septic shock.

**Methods:** This is a prospective, multi center, observational study. Continuous EEG for 12 hours was performed in all consecutive patients acutely admitted in ICU for severe sepsis and sepstic shock from community-acquired infection, within 72 hours from admission. EEG was analyzed for its baseline rhythm, presence of epileptiform activity and according to Synek's classification. Quantitative analysis through compressed spectral array (CSA) was also applied. Neurological examination, including assessment of delirium, coma, brain stem reflexes and level of sedation were assessed daily for 14 days. Multiple logistic regression was used to analyze the relations between physiologic variables, EEG findings, delirium and mortality.

**Results:** Nineteen patients were included, mean age was 79 (47–86) years and 11 (58 %) were female. Continuos EEG was performed for a duration of 12 (10–12) hours. Six patients (32 %) developed septic shock and 4 (21 %) were mechanically ventilated. Overall, 4 patients (21 %) developed delirium and 3 (16 %) patients died in the ICU. EEG recordings showed background slowing (theta or delta frerquency range) in 8 (42 %) patients. Absence of EEG reactivity was observed in 2 patients (11 %), periodic epileptiform discharges (PEDs) in 2 (11 %) and no electrographic seizures were recorded. Moreover Synek's score ≥3 was found in 6 (32 %) patients. Organ failure and derranged systemic physiologic parameters were more frequent in patients with altered EEG.

In patients that developed delirium, EEG with a Synek's score ≥3 was more frequent than in those that did not (75 % vs 14 %, P = 0.04). Also among nonsurvivors Synek's score ≥3 was more common than among survivors (100 % vs 19 %, P = 0.02).

**Conclusions:** In this pilot prospective study of 19 patients with severe sepsis or septic shock, continuous EEG with a Synek score ≥3 was more common in patients that developed delirium and among non survivors.

## CONTEMPORARY ISSUES IN INFECTION AND SEPSIS II

### A848 Infections related to external ventricular drainage in critically ill patients

#### J.C. Barrios Torres^1^, N.J. Fernández Araujo^1^, P. García-Olivares^1^, E. Keough^1^, M. Dalorzo^1^, L.K. Tang^1^, I. De Sousa^1^, M. Díaz^1^, L.J. Marcos-Zambrano^2^, J.E. Guerrero^1^

##### ^1^Hospital Gregorio Marañon, Intensive Care Unit, Madrid, Spain; ^2^Hospital Gregorio Marañon, Microbiology, Madrid, Spain

###### **Correspondence:** J.C. Barrios Torres – Hospital Gregorio Marañon, Intensive Care Unit, Madrid, Spain

**Introduction:** External ventricular drains (EVDs) are used for the management of elevations in intracranial pressure secondary to acute hydrocephalus primarily caused by subarachnoid (SAH), intracerebral (ICH) or intraventricular hemorrhage (IVH) or traumatic brain injury (1). A serious complication of external cerebrospinal fluid (CSF) drainage is colonization of the catheter and posterior retrograde infection resulting in ventriculomeningitis. Rates of EVD associated ventriculitis range from 0 to 22 %(1,2,3).

**Objective:** To assess the incidence and risk factors for EVD-related infections in critically ill patients with acute cerebral pathology.

**Methods:** Retrospective study of patients admitted to the Intensive Care Unit (ICU) (2014–2015) requiring placement of an EVD due to acute cerebral pathology. Epidemiological and clinical data were collected, as well as data related to the EVD: insertion site, samples of CSF and microbiological isolates. EVD-related infection was defined as the presence of systemic signs of infection associated with an inflammatory CSF. Descriptive analysis was expressed as a mean (standard deviation) or median (interquartile range) for quantitative variables and percentages for qualitative. The association of factors related to EVD infections was performed using simple logistic regression, with the corresponding Odds Ratio.

**Results:** 30 patients. 56 % males. Age 55 ± 14 years. Charlson Index: 1 (IQR 0–4).Illness severity scores on admission: APACHE II 17 ± 5; SOFA 5 ± 3; GCS 7 (IQR 3–11). Reason for admission: SAH 60 %, ICH 28 % and ischaemic stroke 4 %. IVH was seen in 88 % of cases. EVD was inserted in the ICU in 88 % of patients, 10 % required replacement, lasting 17 ± 12 days. Two samples of CSF per patient (IQR 1–4) were obtained. Inflammatory CSF seen in 56 % of patients, with 35.7 % having a microbiological isolate (20 % of total), the most frequent being GRAM positive bacteria (62 % S. epidermidis). Elapsed time from placement of the EVD to infection was 11 ± 6 days. ICU stay 23 days (IQR 15–30), 16.2 % mortality. Length of hospital stay 52 days (IQR 35–77), 22.5 % mortality. A poor functional status (GOS 1–3) at discharge was found in 46 %.

In the univariate analysis, factors associated with EVD-related infection were IVH (RR 2.75, 95 % CI 1.58-4.78), EVD placement in ICU (RR 2.75, 95 % CI 1.58-4.78) and EVD days (OR 1.10, 95 % CI 1.01-1.22). There was non significant trend towards an unfavorable GOS (OR 2.50, 95 % CI 0.8-16.42), increased ICU stay (18 ± 12 vs 29 ± 14 days, OR 1.03, 95 % CI 0.94-1.12) and longer hospital stay (44 ± 23 vs 82 ± 72, OR 1.02, 95 % CI 0.98-1.06).

**Conclusions:** Our study showed a high incidence of EVD-related infection. Factors associated with its development include IVH, insertion outside the operating theatre and EVD placement days.

**References**

1. Hagel et al. *Interdiscip Perspect Infect Dis* 2014; **2014**: 708531.

2. Beer et al. *Neurocrit Care* 2009; **10**: 363–7.

3. Lozier et al. *Neurosurgery* 2002; **51**: 170–8

### A849 Clinical and ventilatory differences between adult critical patients survivors and non survivors with moderate and severe ARDS due to 2009 AH1 N1 influenza virus

#### S.E. Zamora Gomez, G.D. Hernandez Lopez, A.I. Vazquez Cuellar, O.R. Perez Nieto, J.A. Castanon Gonzalez

##### Hospital Juarez de México, ICU, Mexico City, Mexico

###### **Correspondence:** S.E. Zamora Gomez – Hospital Juarez de México, ICU, Mexico City, Mexico

**Introduction:** The global rate mortality due to 2009 AH1 N1 influenza virus in critical care patients has not been well estimated, México is considered as the origin of this pandemic disease, and some reports of the CDC (Centers for Disease Control and Prevention) suggest the presentation in this country is usually more severe than in others, with a higher risk of death.

**Objectives:** The objective of this study was to determine the clinical and ventilatory differences betwen patients survivors and non survivors with diagnostic of moderate and severe ARDS due to confirmed 2009 AH1 N1 influenza virus, hospitalized in ICU at a mexican tertiary center.

**Methods:** Observational, retrospective study including adult patients with moderate and severe ARDS, due to RT-PCR confirmed 2009 AH1 N1 influenza virus, hospitalized in the ICU during march 2009 to march 2016. Patients were analyzed studied in two groups:survivors and non survivors.

**Results:** 27 patients were analyzed, 14 patients with moderate and 13 with severe ARDS,12 females and 15 males; mortality was of 52 %,13 survivors (sv) and 14 non survivors (nsv). The mean APACHE II score was 26 points (23 pts in sv vs 29 pts in nsv),mean SOFA score of 12 points (11 pts in sv vs13 pts in nsv). The median age was 46.07 years, with a median of 49.07 yrs in sv vs 43.28 yrs in nsv. The median body-mass index was 33.3,29.4 in sv vs 37 in nsv.The median of stay in ICU was 21.25 d, with a median of duration of mechanical ventilation of 18.44 d,23.81 d in sv vs 13.07 in nsv. Tracheostomy was performed in 19 patients (70 %),12 in the sv, 7 in the nsv.Mean PaO2/FiO2 ratio at entry in ICU was 91.5,100.1 in sv vs 82.3 in nsv. Prone position was performed in 20 patients (74 %), with a mean duration of 6.6 d, 7.99 d in sv vs 5.33 d in nsv. The mean highest PEEP was 14.35 cmH2O with a mean of 12 cm of H2O in sv vs 16.7 cm of H2O in nsv. 12 patients (44.4 %) received CRRT,3 (25 %) in the sv vs. 9 (75 %) in the nsv group.

**Conclusions:** In this study the main differences in the group of non survivors patients in comparisson with survivors were a higher body-mass index, higher scores in mortality prediction scales, a minor age, a lower PaO2/FiO2 ratio at entry in ICU, the need of higher levels of PEEP, and a higher need of CRRT as reflect of progression to MODS.

**References**

1.- Writing committee of the WHO consultation on clinical aspects of pandemic 2009 influenza.clinical aspects of pandemic 2009 influenza A(H1 N1) virus infection.N Engl J Med 2010;362:1708–19

2.- Chowell G, et al.Severe respiratory disease concurrent with the circulation of H1 N1 influenza. N Engl J Med 2009;361:674–9.

### A850 H1 N1 pneumonia related ARDS: encouraging results with early corticosteroids

#### D. Bhasin^1^, S. Rai^1^, H. Singh^1^, O. Gupta^1^, M.K. Bhattal^1^, S. Sampley^1^, K. Sekhri^2^, R. Nandha^2^

##### ^1^Max Superspeciality Hospital, Critical Care, Pulmonary and Sleep Medicine, Mohali, India; ^2^Dr. H.S.J Institute of Dental Sciences, Pharmacology, Chandigarh, India

###### **Correspondence:** D. Bhasin – Max Superspeciality Hospital, Critical Care, Pulmonary and Sleep Medicine, Mohali, India

**Introduction:** The emergence of H1 N1 pneumonia leading on to ARDS with its catastrophic course changed the very outlook of how to approach viral pneumonia in adults. The window of time period is very limited in which to act and rapidly deteriorating oxygen saturation despite high PEEP and Fio2 requirement necessitates expedient measures to halt the fast deteriorating process. In this scenario we attempted to use corticosteroids and got excellent results.

**Objectives:** A minority of the patients who contract H1 N1 develops a rapidly progressive pneumonia leading to acute respiratory distress syndrome (ARDS). Role of corticosteroids is controversial in treatment of H1 N1 pneumonia related ARDS. The present study was undertaken to establish the use of corticosteroids in shaping the course of H1 N1 related ARDS.

**Methods:** A retrospective observational study (May 2012 - March 2016) was conducted in a Medical Intensive Care Unit of a tertiary care hospital in Northern India. Total of 24 confirmed cases of H1 N1 pneumonia with ARDS were enrolled. The H1 N1 pneumonia was confirmed with real time reverse transcriptase polymerase chain reaction assay. The response to the administration of prolonged corticosteroid therapy (Methylprednisolone 1 mg/kg/day in a continuous infusion) along with oseltamivir (150 mg twice a day) was studied. Severity of illness and Multi Organ Dysfunction were quantified with Acute physiology and chronic health evaluation II (APACHE II) score at 24 hours of admission, Sequential organ failure assessment (SOFA) and Lung injury score (LIS) on day 1 & day 7 of hospitalisation.

**Results:** On an average after one week of treatment, patients with confirmed H1 N1 pneumonia related moderate to severe ARDS, who required non invasive or invasive ventilation experienced a significant improvement in lung injury score. In 16 of 24 patients, Lung Injury Score was measured to be 3.35 ± 0.42 at the time of admission and was decreased to 1.8 ± 0.9 on day 7 of hospitalisation. In hospital mortality was found to be only 13 % ( 20 out of 23 patients survived)

**Conclusions:** In Patients of H1 N1 with confirmed ARDS, administration of early & prolonged low dose corticosteroids showed significant improvement in lung function with prevention of fibrosis and permanent pulmonary damage. It was also associated with significant low in hospital mortality.

**References**

1. An expanded definition of the adult respiratory distress syndrome. Murray JF, Matthay MA, Luce JM, Flick MR. Am Rev Respir Dis. 1988 Sep;138(3):720–3.

2. Acute Respiratory Distress Syndrome: The Berlin Definition *JAMA.* 2012;307(23):2526–2533.

3. H1 N1 influenza A virus-associated acute lung injury: response to combination oseltamivir and prolonged corticosteroid treatment. Quispe-Laime, A. M.; Bracco, J. D.; Barberio, C. G.; Rolfo, V. E.; Umberger, R.; Meduri, G. U. J ICM 2010 Vol. 36 No. 1 pp. 33–41

### A851 Viral induce acute respiratory distress syndrome (ARDS): characterization of patients in a tertiary hospital in South America

#### F.A. Aliaga^1,2^, F. Olivares^2^, F. Appiani^2^, P. Farias^2^, F. Alberto^2^, A. Hernández^2^

##### ^1^Instituto Nacional del Tórax, Fellow Respiratory Medicine, Santiago, Chile; ^2^Hospital Militar de Santiago, Intensive Care, Santiago, Chile

###### **Correspondence:** F.A. Aliaga – Instituto Nacional del Tórax, Fellow Respiratory Medicine, Santiago, Chile

**Introduction:** ARDS is an important cause of admission to intensive care unit and account a mortality of 40 % in it moderate form. The objective of this study is characterization of viral ARDS patients admitted to our intensive care unit

**Methods:** Descriptive, retrospective study of patients admitted to a critical care unit in a Military Hospital of Santiago Chile with viral ARDS during year 2015. All patient have positive polymerase chain reaction (PCR) to a viral etiology. We analyze demographic, clinics, oxygenation, ventilator settings, prone positioning, neuromuscular blocking, 28 days mortality, intensive care stay and etiology study. Statistical analysis, measures of central tendency, percentages and chi square test.

**Results:** We identify 15 patients. The median age 67 years, 25 % female and 80 % with comorbidities. 66 % need invasive mechanical ventilation (IMV) and 34 % non invasive. Mean PaO2/FIo2 138, with APACHE score of 17. Mean tidal volume 6.2 ml/kg with plateau pressure of 26.7 and driving pressure 13.8 %. 90 % use neuromuscular blockers and 60 % prone positioning ventilation. The mean time of IMV 8 days y ICU stay 18 days. In viral PCR 53 % positive for influenza, 3 adenovirus and 2 rhinovirus. The mortality at 28 days 26.6 %. We observe a statistic association between white cell count over 20.000 and 28 days mortality (p 0.012).

**Conclusions:** We found less mortality than reported in LUNG SAFE study for moderate ARDS and observe and association between mortality and high white cell.

### A852 Early bacterial infectious complications following heart transplantation

#### S. Pons^1^, R. Sonneville^1^, L. Bouadma^1^, M. Neuville^1^, E. Mariotte^1^, A. Radjou^1^, J. Lebut^1^, S. Chemam^1^, G. Voiriot^1^, M.-P. Dilly^2^, B. Mourvillier^1^, R. Dorent^3^, P. Nataf^3^, M. Wolff^1^, J.-F. Timsit^1^

##### ^1^Bichat University Hospital, Medical ICU, Paris, France; ^2^Bichat University Hospital, Anaesthesiology, Paris, France; ^3^Bichat University Hospital, Cardiovascular surgery, Paris, France

###### Correspondence: S. Pons – Bichat University Hospital, Medical ICU, Paris, France

**Introduction:** National priority heart transplantation for severe acute heart failure has increased. The impact of early infectious complications has never been assessed in a large cohort of ICU patients.

**Objectives:** We aimed to determine the characteristics, the determinants and the impact of infectious complications after heart transplantation.

**Methods:** We retrospectively studied all consecutive heart-transplant recipients in Bichat Hospital, between January 1st, 2011 and June 6th, 2015. All infectious complications that occurred within six months after transplantation were considered for analysis. The primary endpoint was the rate of bacterial complications at 6 months. We used a multivariate logistic regression to identify risk factors for bacterial infections. Data are presented as median (IQR) or number (percentage).

**Results:** 113 patients (53 years (40–62), male n = 86 (75 %)) were included. At time of heart transplantation, 65 patients (57 %) were hospitalized in ICU with acute heart failure, 28 (25 %) were under extracorporeal membrane oxygenation (ECMO) support. Length of stay in ICU after transplantation was 16 [11–24] days. Seventeen (15 %) died in the ICU and 19 (17 %) died within 6 months. CMV mismatch was present in 15 (13 %) recipients. Twenty-two (19 %) patients had a multidrug-resistant bacteria (MRB) carriage before transplantation, and 6 (5,3 %) acquired one during the first week in ICU. Nine of those (32 %) developed at least one infection due to the same MRB during their stay in ICU.

Seventy-one (63 %) patients developed one or more bacterial infection within six months post-transplantation. The first one occurred in ICU for 66 (93 %) patients. Sixty-five pneumonia were diagnosed in ICU, among them 46 (70 %) before day 8 post-transplantation. Bloodstream (n = 26) and Scarpa infections (n = 17) were as frequent in the early post-operative period than after day 8. During the first post-operative week, most of bacteria were Gram negative bacteria (n = 51), including 6 ESBL and 7 *Pseudomonas aeruginosa*. After this period, infections in ICU were also predominantly due to Gram negative bacteria (n = 39) with higher rates of *P. aeruginosa* (n = 12) and ESBL (n = 10). Among Gram positive bacteria (n = 25), no Methicillin-resistant *Staphylococcus aureus* was found.

Within 6 months after transplantation, 50 patients (44 %) developed a viral complication (36 CMV reactivations). Fungal infection was found in 16 patients (14 %), including 9 invasive aspergillosis.

Bacterial infection in ICU was associated with a longer stay in ICU (20 days (13–29) vs. 12 days (8–17) p < 0,001), but not with increased mortality. After adjustment, ECMO following heart transplantation was identified as the only risk factor for bacterial infections in ICU (Odds ratio = 3.1 (1.4-6.9), p = 0.006).

**Conclusions:** This study confirms the high rate of early infectious complications after heart transplantation. ECMO after transplantation is an independent factor associated with bacterial infections in ICU.

### A853 Characteristics of viral pneumonia patients admitted to the ICU due to hypoxemic respiratory failure

#### O. Ediboglu, S. Ataman, H. Ozkarakas, C. Kirakli

##### Dr. Suat Seren Chest Diseases and Surgery Training Hospital, Intensive Care Unit, Izmir, Turkey

###### **Correspondence:** O. Ediboglu – Dr. Suat Seren Chest Diseases and Surgery Training Hospital, Intensive Care Unit, Izmir, Turkey

**Introduction:** Hypoxemic respiratory failure due to viral pneumonia during epidemic seasons sometimes may require mechanical ventilation support and ICU stay. Mortality of these patients can be high despite young age (1,2).

**Objectives:** To evaluate the characteristics and risk factors of viral pneumonia patients admitted to our ICU due to hypoxemic respiratory failure.

**Methods:** Patients admitted to our ICU between December 2015 and March 2016 who had hypoxemic respiratory failure due to clinically and radiologically suspected viral pneumonia were enrolled. Demographic characteristics, risk factors, need and type of mechanical ventilatory support, laboratory values, need of extracorporeal pulmonary (ECMO) and renal support (CRRT) and mortality rate were evaluated. Data are expressed as median (25^th^ -75^th^ percentile) or n (%) and compared using Mann Whitney U test or Chi-square where appropriate.

**Results:** Twenty two patients (11 male) met the enrollment criteria. Median age and APACHE II score was 45 (36–63) years and 19 (13–25) respectively. Time from the onset of symptoms to admission to the ICU was 7 (6–9) days. Most common symptoms were shortness of breath, cough and fever respectively. Risk factors were present at 8 (36 %) patients. Maximum set PEEP levels during mechanical ventilation was significantly lower in patients who survived [10 (8–10) vs 13 (10–16), p = 0.025)]. Deceased patients spent more time under a PaO_2_/FiO_2_ ratio below 100 [72 (24–90) vs. 0 (0–48) hours, p = 0.024]. Serum albumin levels were also lower in deceased patients [2.6 (2–3.1) vs. 3.5 (2.6-3.7) g/dl, p = 0.033]. Two patients needed ECMO support and 5 patients underwent CRRT due to acute renal failure. Survival rate was 88 % (7/8) in patients who had noninvasive ventilation (NIV) success while it was only 7 % (1/14) in patients who had undergone invasive mechanical ventilation (p < 0.001). Overall mortality was 64 %.

**Conclusions:** Viral pneumonia may result in severe hypoxemic respiratory failure and ARDS especially during epidemic seasons. Mortality of these patients can be relatively high despite full support including ECMO and CRRT. NIV success, time spent under a PaO_2_/FiO_2_ ratio below 100 and low serum albumin levels at admission may be predictors of severity of the disease and mortality.

**References**

1. Crotty MP, Meyers S, Hampton N, Bledsoe S, Ritchie DJ, Buller RS, Storch GA, Micek ST, Kollef MH. Epidemiology, Co-Infections, and Outcomes of Viral Pneumonia in Adults: An Observational Cohort Study. Medicine (Baltimore). 2015Dec; 94(50):e2332.

2. Kirakli C, Tatar D, Cimen P, Edipoglu O, Coskun M, Celikten E, Ozsoz A. Survival from severe pandemic H1 N1 in urban and rural Turkey: a case series. Respir Care. 2011 Jun;56(6):790–5.

### A854 Impact of FFP transfusion on percentage of ventilated patients developed VAP in ICU patients

#### A. Vakalos, V. Avramidis

##### Xanthi General Hospital, ICU, Xanthi, Greece

###### **Correspondence:** A. Vakalos – Xanthi General Hospital, ICU, Xanthi, Greece

**Introduction:** While plasma donation is still necessary as a unique source of human proteins and to treat coagulation disorders, FFP administration seems to have high rate of inappropriate indication. After all, FFP transfusion is not risk free, and is associated with lung injury, infectious disease, circulatory overload and immunosuppression in recipients. On the other hand, Ventilator Associated Pneumonia (VAP) is one of the most frequently seen infections in ICU setting and may have an impact not only to the length of ICU stay, but o ICU outcome as well.

**Objectives:** The aim of our observation retrospective study was to test the hypothesis that a correlation exists between FFP transfusion and the percentage of ventilated patients developed VAP (% VP) in our both medical and surgical ICU served in community hospital.

**Methods:** From January 2006 to June 2014 admitted to our ICU 620 patients, mean age 64.8 years, mean length of ICU stay (LOS) 14.2 days, mean mechanical ventilation duration per ventilated patient (V. Days) 12.23 days, mean APACHE II score on admission 21.2, predicted mortality 38.9 %, actual mortality 31.45 %, Standardized Mortality Ratio (SMR) 0.80. From our database we looked for the percentage of ventilated patients developed VAP and the following values and indexes according FFP transfusion per year from 2006 to 2014 (mean values). Total, per patient, per hospitalization days (HD), per patient under mechanical ventilation (pts V) and per ventilation days (VD) Using linear correlation method, we looked for linear slope, correlation coefficient (r), and coefficient of determination (r^2^), and by linear regression method using ANOVA test we looked for p value, according % VP and FFP transfusion.

**Results:**

**Conclusions:** According to our data, there was no statistically significant correlation detected between the percentages of ventilated patients developed VAP and FFP transfusion indexes. Our data suggest that even though FFP transfusion may have an impact on immunosuppression and infection disease developing, the impact on the percentages of ventilated patients developed VAP is not statistically significant.

**Table 11 (abstract A854).** Results

FFP	Slope	r	r2	St. Error	L. CI	U. CI	p Value
Total Tran	−1.916	−0.210	0.044	3.369	−9.885	6.052	0.5873
Trans per pt	−0.084	−0.544	0.296	0.048	−0.199	0.031	0.1294
Trans per H.D.	−0.005	−0.450	0.184	0.004	−0.014	0.004	0.5480
Trans per Pt V.	−0.084	−0.540	0.291	0.051	−0.210	0.034	0.1334
Trans per V. D.	−0.008	−0.518	0.268	0.005	−0.020	0.003	0.1529

### A855 Sensitivity of biomarkers of sepsis in patients receiving anticancer therapy

#### O. Obukhova^1^, I.A. Kurmukov^1^, S. Kashiya^1^, E. Golovnya^2^, V.N. Baikova^2^, T. Ageeva^3^, T. Haritydi^3^, E.V. Kulaga^4^

##### ^1^N.N. Blokhin Russian Cancer Research Center, Medical ICU, Moscow, Russian Federation; ^2^N.N. Blokhin Russian Cancer Research Center, Express Laboratory, Moscow, Russian Federation; ^3^N.N. Blokhin Russian Cancer Research Center, Biochemical Laboratory, Moscow, Russian Federation; ^4^N.N. Blokhin Russian Cancer Research Center, Microbiology Department, Moscow, Russian Federation

###### **Correspondence:** O. Obukhova – N.N. Blokhin Russian Cancer Research Center, Medical ICU, Moscow, Russian Federation

**Introduction:** In patients receiving anti-cancer therapy, clinical and laboratory signs of treatment-related toxicity may coincide with the sepsis criteria, which makes early diagnosis difficult. Defining laboratory "markers of sepsis" - C-reactive protein (CRP), procalcitonin (PCT) and presepsin (PRS) is considered an additional method of diagnostics.

**Objectives:** To estimate the sensitivity of laboratory markers of sepsis in patients receiving anticancer therapy.

**Methods:** A total of 56 patients with a reliably diagnosed sepsis (SIRS + defined source of infection ± identification of the infectious agent) were examined. Blood samples for the determination of sepsis markers were carried out within the first hour of ICU hospitalization. CRP and PCT concentration in the serum was determined in all patients and PRC concentration - in 18 patients. The results for each marker are represented as Me (min-max). The calculation of 95 % confidence intervals (95%CI) was carried out by means of the Wilson method. For the diagnosis of sepsis, marker cut-off levels values exceeding 50 mg/l for CRP, 2 ng/ml for PCT and 600 pg/ml for PRS were taken into account.

**Results:** The medians of CRP, PCT and PRS were 102 mg/l (0.7 to 455.9); 4.15 ng/ml (0.5 to 200) and 789.5 pg/ml (343 to 6440), respectively. The sensitivity of CRP, PCT and PRS as positive markers of sepsis were 0.83 (95%CI 0.71-0.90); 0.61 (95 % CI 0.48-0.72); 0.39 (95%CI 0.20-0.61), respectively.

**Conclusions:** CPR was the most sensitive sepsis marker in patients receiving anticancer therapy. In the examined population, values PCT and PRS within first few hours from sepsis development showed relatively low sensitivity.

### A856 Serum biomarkers and scores prognostic in severe sepsis/septic shock

#### J.J. Rios-Toro^1^, L. Perez-Borrero^1^, E. Aguilar-Alonso^2^, M.D. Arias-Verdu^3^, J.M. Garcia-Alvarez^1^, C. Lopez-Caler^3^, C. De La Fuente-Martos^2^, S. Rodriguez-Fernandez^1^, M. Gomez Sanchez-Orézzoli^1^, F. Martin-Gallardo^1^

##### ^1^Hospital Serrania, Ronda, Spain, ^2^Hospital Infanta Margarita, Intensive Care, Cabra, Spain, ^3^Hospital Regional, Intensive Care, Malaga, Spain

###### **Correspondence:** L. Perez-Borrero – Hospital Serrania, Ronda, Spain

**Introduction:** Mortality is elevated in patients with severe sepsis and, even more, in those with septic shock. Early diagnosis and appropriate treatment improve the survival in them.

**Objective:** To explore the prognostic value of procalcitonin (PCT) and C-reactive protein (CRP) serum levels for patients with severe sepsis and/or septic shock in a intensive care unit (ICU) and compare it with scores prognostic.

**Methods:** 50 patients admitted at the intensive care unit (ICU) with the diagnosis of severe sepsis or septic shock were studied. SOFA and APACHE II scores as well as serum were measured at days 1,2 and 5. The influence of these variables on 28-day mortality was analyzed. 20 healthy individuals served as controls.

**Results:** The sample is composed of 50 patients with severe sepsis and/or septic shock with an mean age 63.7 ± 14.25 years and APACHE II on admission of 19.14 ± 7.7 points. SOFA score of 7.62 ± 3.8 points. Mortality was 42 %.

Patients who died on admission showed no statistically significant differences in APACHE-II 19.27 ± 6.08 vs 19.10 ± 8.16 points and SOFA score 8.55 ± 5.59 vs 7.36 ± 3.87 points. The inflammatory markers commonly analyzed had lower values of CRP 166 ± 100 vs 223 ± 117 mg/dL (p = 0.081) and PCT 10 ± 21 vs 24 ± 36.68 ng/mL (p = 0.144) but without being statistically significant differences.

The changes between admission and the first day and its relation with mortality was analyzed, the inflammatory markers compared to surviving patients showed a decrease in CRP , −4.81 ± 55.97 vs −0.19 ± 73.48 (p = 0.891 ) and PCT −15.20 ± 22.65 vs 0.6 ± 14.76 (p = 0.745), and these decreases were not statistically significant.

Clinical severity indicators show a statistically significant decline in the APACHE II between the second day and income −1.56 ± 4.72 vs −0.98 ± 5.13 (p < 0.001) and SOFA score 0.29 ± 2.55 vs 0.45 ± 3.70 (P < 0.001).

The discrimination of changes regarding mortality was analyzed with the area for APACHE II changes of 0.771 (0.62-0.93) and SOFA evolution 0.68 (0.5-0.85). And the area for CRP changes and PCT was only 0.52 and 0.51.

Multivariate analysis with logistic regression showed that mortality was statistically significant associated with the SOFA change wih OR: 1.76 (1.05-2.95) and without statistically significant relation with PCT, CRP and APACHE II changes.

**Conclusions:** In septic patients admitted to the ICU, the improving of APACHE and SOFA score are more sensitive markers of survival than the evolution of inflammatory parameters more commonly used as CRP and PCT.

### A857 Correlates of perfusion markers with base line mortality prediction in icu? An elusive search for the best performer……

#### J. Nikhilesh^1^, V. Joshi^2^

##### ^1^CHL Hospitals, Dept of Critical Care Services, Indore, India; ^2^Shalby Hospital, Dept of Critical Care Services, Indore, India

###### **Correspondence:** J. Nikhilesh –CHL Hospitals, Dept of Critical Care Services, Indore, India

**Background:** There have been various perfusion markers that have been evaluated in literature to implement strategies to optimize management in subsets of sepsis. However, a definitive answer to the same remains debatable.

**Objective:** To determine performance of makers of tissue perfusion such as lactate, base excess and pCO2 gap (central venous to arterial carbon dioxide gap) at baseline in patients of sepsis and correlate them with predicting mortality in these subsets.

**Setting:** Multidiscliplinary 60 bedded medical surgical ICUs of two tertiary care hospitals.

Study Module: Consecutive patients presenting with sepsis getting admitted to ICU were included for a duration dated Jan 2015-Mar 2016 and data was collected at baseline with a focus on demographics, SOFA scores, Lactate, Base excess pCO2 gap, Length of stay (LOS) in days and mortality. Death/discharge from ICU were considered as end points. Patients with COPD/prior ILD, Hepatic failure and out of hospital cardiac arrests were excluded. ROC curves for association with mortality were constructed using SPSS version 22 and logistic regression analysis was done for the same data.

**Results:** Fifty five patients were included (n = 55). Male: Female ratio was (M: F-37:18) with age 42.5 ± 12.3 years (range-22-69).The baseline lactate, Base excess and pCO2 gaps were 9.41 ± 3.43 (range 5–19), 7.23 ± 2.02 (range-4-13) and 6.21 ± 1.66 (range- 3.9-9.6) respectively. SOFA scores at baseline were 13.7 ± 2.08 (range-10-18).LOS was 4.32 ± 1.98 (range-1-9 days).In hospital mortality was 38.2 % (n = 21).ROC curves were drawn and AUC for lactate, Base excess and pCO2 gap was 0.74, 0.028 and 0.169 respectively.

**Conclusion:** In subsets of sepsis lactate levels score over base excess and pCO2 gap in terms of sensitivity in predicting mortality however base excess and pCO2 gap are more specific vis-a-vis lactate levels in prediction of mortality with base excess being the more specific marker. We need to review it further in terms of differentiating these subsets and validating this data in subsets of specific settings of multidiscliplinary critical care.

**Grant acknowledgement**

There are no grants for this project for all the authors.

### A858 Behavior of IgM antiendotoxin-core antibodies in septic shock. A preliminary study

#### E. Villarreal^1^, J. Ruiz^2^, M. Gordon^2^, A. Quinza^2^, J. Gimenez^2^, M. Piñol^2^, A. Castellanos^2^, P. Ramirez^2^

##### ^1^Hospital la Fe, Intensive Care Unit, Valencia, Spain; ^2^Hospital la Fe, Valencia, Spain

###### **Correspondence:** E. Villarreal – Hospital la Fe, Intensive Care Unit, Valencia, Spain

**Introduction:** The bacterial lipopolysaccharide (LPS) or endotoxin is a major component of the outer membrane of all Gram-negative bacteria. LPS plays an important role in the normal physiology, permeability and fluidity of the bacterial outer cell wall. Endotoxaemia is associated with infections caused by Gram-negative bacteria, but can occurs in absence of it. In sepsis, some of the protective mechanisms of gut barrier are disrupted and subsequently, bacterial products, like endotoxin, may cross the gut mucosa and spread to the mesenteric lymph nodes or more distant organs. This phenomenon is known as bacterial translocation (BT).

**Objectives:** Evaluate IgM antiendotoxin-core antibodies (IgM EndoCAb) and inflammatory biomarkers (C-reactive protein, procalcitonin and interleuquin-6), in septic shock.

**Methods:** Prospective observational study of consecutive septic shock patients admitted to our intensive care unit (ICU). Patients were followed during the first three days after SS. Inflammatory biomarkers (CRP, PCT, IL-6), and IgM EndoCAb were measured daily. Clinical, hemodynamic and microbiological data were collected.

**Results:** We included 25 patients.

Urinary and abdominal were the infectious disease more diagnosed (32 %).

Gram negative bacteria (GNB) was isolated in 40 % (n 12) and Gram-positive bacteria (GPB) in 32 % (n 9).

Patients with infection caused by Gram-negative bacteria, showed lower IgM EndoCAb levels all days without statistical significance (p = 0.13).

IgM EndoCAb were lower in patients with shock no resolved (4.9 ± 2; 5.7 ± 3 and ± 2 vs 4.8 ± 24.3 41; 28.3 ± 51 and 22.3 ± 41 respectively) and those who died (5.9 ± 3; 6.5 ± 2 and ± 5.6 25 ± 2 vs 42; 29.4 ± 52 and 23.1 ± 43 respectively) but difference was not significant.

An inverse correlation between inflammatory biomarkers (PCT, CRP and IL-6) and IgM EndoCAb was detected. IL6 showed a higher correlation, but without statistically significant differences.

ICU mortality rate was 8 %.

**Conclusion:** IgM EndoCAb were detected in septic shock caused by GPB, it could be explain by a bacterial translocation.

Patients with major endotoxaemia have higher consumption of antibodies and therefore lower levels of IgM EndoCAb that is associated with a worse prognosis.

### A859 The relationship between the neutrophil/lymphocyte ratio and mortality in the severe sepsis patients

#### Y.D. Jeon^1^, W.Y. Jeong^2^, M.H. Kim^2^, I.Y. Jeong^2,3^, M.Y. Ahn^2^, J.Y. Ahn^2^, S.H. Han^2,3^, J.Y. Choi^2,3^, Y.G. Song^2,3^, J.M. Kim^2,3^, N.S. Ku^2,3^

##### ^1^Yonsei Wonju University College of Medicine, Department of Internal Medicine, Wonju, Republic of Korea; ^2^Yonsei University College of Medicine, Department of Internal Medicine, Seoul, Republic of Korea; ^3^Yonsei University College of Medicine, AIDS Research Institute, Seoul, Republic of Korea

###### **Correspondence:** Y.D. Jeon – Yonsei Wonju University College of Medicine, Department of Internal Medicine, Wonju, Republic of Korea

**Introduction:** The neutrophil/lymphocyte ratio (NLR) is known as the prognostic marker in the various malignancies and cardiovascular disease. Several studies reported about the relationship between NLR and mortality in the severe sepsis patients.^1–3^ But these studies have shown conflicting results.

**Objectives:** So, we investigated the relationship between NLR and 28-day mortality in the severe sepsis patients.

**Methods:** The electronic medical records of patients enrolled in Early Goal Directed Therapy (EGDT) between January 2010 and December 2011 at Severance Hospital, a 2000-bed tertiary care hospital in Seoul, Korea, were retrospectively reviewed. Analysis was performed to access the relationship between NLR and 28-day mortality.

**Results:** Total 198 patients of EGDT were enrolled in the study. 102 (51.5 %) patients were male and the mean age was 63.30 years. The primary focus of sepsis was the pneumonia in 56 (28.3 %), urinary tract infection in 56 (28.3 %), intra-abdominal infection in 23 (11.6 %) and gastroenteritis in 15 (7.6 %). The median NLR was 16.23 (interquartile range, 7.37 to 27.76) and the overall 28-day mortality rate was 11.1 %. NLR (p = 0.975) was not significantly associated with 28-day mortality. On mutlivariate analysis, SOFA score (p < 0.001) was only independent factor associated with mortality.

**Conclusions:** In our study, NLR was not associated with 28-day mortality in the severe sepsis patients.

**References**

1. Akilli NB, Yortanli M, Mutlu H, et al. Prognostic importance of neutrophil-lymphocyte ratio in critically ill patients: short- and long-term outcomes. *The American journal of emergency medicine.* 2014;32(12):1476–1480.

2. Riche F, Gayat E, Barthelemy R, Le Dorze M, Mateo J, Payen D. Reversal of neutrophil-to-lymphocyte count ratio in early versus late death from septic shock. *Critical care (London, England).* 2015;19:439.

3. Salciccioli JD, Marshall DC, Pimentel MA, et al. The association between the neutrophil-to-lymphocyte ratio and mortality in critical illness: an observational cohort study. *Critical care (London, England).* 2015;19:13.

### A860 Evaluating initiatives advocating appropriate fluid therapy in severe sepsis/septic shock according to the surviving sepsis campaign

#### H. Shah, F. Kellner, F. Rezai, N. Mistry, P. Yodice, V. Ovnanian, K. Fless, E. Handler

##### Saint Barnabas Medical Center, Department of Medicine, Livingston, United States

###### **Correspondence:** H. Shah – Saint Barnabas Medical Center, Department of Medicine, Livingston, United States

**Introduction:** Even under ideal scenarios and maximized medical treatment, severe sepsis/septic shock are amongst the leading causes of morbidity and mortality in hospitalized patients worldwide. The Surviving Sepsis Campaign guidelines emphasize early aggressive intravenous fluids (IVF) resuscitation and antibiotics administration in these patients to improve outcomes.

**Objectives:** Our objective is to assess compliance with dosage and timing of intravenous fluid therapy in severe sepsis/septic shock patients and the effect on hospital length of stay (LOS) after education of emergency department (ED) staff of the recommended treatment protocol in the surviving sepsis guidelines (30 mL/Kg within one hour of diagnosis).

**Methods:** Our 633-bed single center retrospective study evaluated patients 21 years of age older with severe sepsis/septic shock admitted to the intensive care unit (ICU) from the ED. We looked at two time frames, before (April 1 to December 31, 2012) and after (July 1 to December 21, 2014) an educational intervention from the ED pharmacist. Specifically, the ED pharmacist held educational group sessions along with one-on-one education with ED medical personnel. Collected data included patient demographics, blood pressures, lactic acid (LA), dosage and timing of IV fluids and hospital LOS. Patients who qualified for this intervention had least one of the following: LA greater than 4 mm/L, systolic blood pressure (SBP) less than 90 mm/Hg, or SBP lower than 40 mmHg from baseline. Data was collected to determine whether the educational intervention would affect patients receiving adequate treatment using Chi-square testing and if there was an effect on mean length of stay using student-t testing.

**Results:** Pre-intervention, forty five patients with severe sepsis/septic shock were admitted to the ICU from the ED. Forty-two qualified for the study. Only one received adequate fluid resuscitation. Post-education, 41 patients were admitted and thirty-six qualified for the study. Of these eighteen received adequate IVF resuscitation, yielding a P value of < 0.000001 and a significant difference following the educational intervention. However, there was not a significant decrease in mean LOS from 14.3 to 11.8 days between the two groups, with a p-value of 0.276, when excluding patients who expired or were discharged to hospice.

**Conclusions:** In our study, there was a significant increase in patients with severe sepsis/septic shock receiving adequate IVF therapy after an extensive educational intervention was performed. Though there was a decrease in mean hospital LOS, it was not calculated to be significant. As patients in both populations frequently had additional comorbidities along with social and financial considerations affecting their hospital discharge, confounding factors affecting their LOS were likely to be present.

**Fig. 14 (abstract A860).**
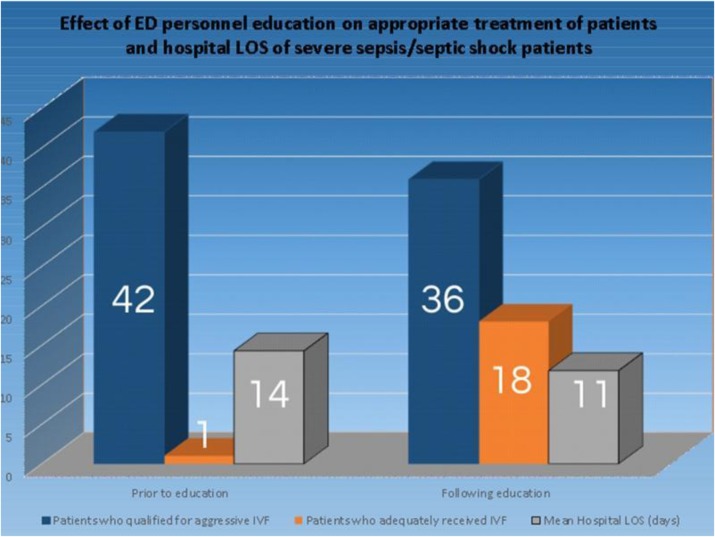
Effect of education on appropriate treatment & LOS.

## SPOTLIGHT ON ICU N&AHP PRACTICE

### A861 Efficacity of mechanical insufflation-exsufflation devices in intubates and ventilated ICU patients

#### R. Martínez Alejos^1^, J.D. Martí Romeu^2^, D. González Antón^1^, A. Quinart^1^, A. Torres Martí^2^

##### ^1^CHU Bordeaux, Bordeaux, France; ^2^Hospital Clínic, Barcelona, Spain

###### **Correspondence:** R. Martínez Alejos – CHU Bordeaux, Bordeaux, France

**Introduction:** Critically ill patients on invasive mechanical ventilation (IMV) often present retention of respiratory secretions, increasing the risk for morbidity. Endotracheal suctioning (ETS) is the main strategy to prevent mucus retention, but its effects are limited to the first bronchial bifurcation.

Mechanical in-exsufflation (MI-E) is a non-invasive chest physiotherapy (CPT) technique aimed to generate high expiratory flows to simulate cough, and improve mucus clearance in the proximal airways. To date, no studies have assessed the effects of MI-E in intubated and sedated critically ill patients.

**Objectives:**

- To evaluate the effect of CPT with or without MI-E device application on mucus clearance and to evaluate MI-E effect on pulmonary mechanics.
- To assess expiratory flows (PEF) generated during MI-E intervention.

**Methods:** Controlled, randomized semi cross-over, single blind trial conducted at Bordeaux Universitary Hospital. We included intubated and sedated patients (>18 ys) connected to IMV > 48 h. We excluded patients with parenchyma damage, respiratory and/or hemodynamic instability, and high infectious risk. All patients received CPT followed by an ETS twice daily. Additionally, patients randomly received MI-E in one of the sessions (one day intervention).MI-E treatment consisted in 4 series of 5 in-expiratory cycles at +/− 40 cmH_2_O pressure, 3 sec inspiratory time, 2 sec expiratory time, and 1 sec pause between cycle.

Mucus clearance was assessed through wet volume of sputum retrieved during ETS.

Pulmonary mechanics was measured before, immediately after and 1 hr after intervention through a pneumotacograph (PNT). PEF during MI-E was continuously measured through a PNT.

**Results:** 10 patients were enrolled in the study

The volume of secretions recovered (Me, IQR) was 1.1 ml (0.76-2.29) and 0.41 ml (0–1.27) with or without MI-E respectively (p = 0.06).

No significant differences in pulmonary mechanics were found before, immediately after or 1 hour after the use of MI-E or between groups.

PEF generated during MI-E was 1.66 ± 0.42 l/s.

**Conclusion:** MI-E may not be a useful technique to improve mucus clearance in intubated and sedated critically ill patients when conventional setting is used. Indeed, the MI-E setting applied in our study (ie: +/− 40 cmH_2_O) did not generate an efficient cough, which is considered to be PEF > 2.7 l/s in this population of patients.

MI-E does not have significant short or long-effects on pulmonary mechanics.

**References**

Branson RD. Respir Care 2007; 52(10): 1328–42.

Homnick D. Respir Care. 2007; 56: 1296–307.

Guérin C et al. Respir Care. 2011 Aug; 56(8):1108–14.

**Table 12 (abstract A861).** Anthropometric values of enrolled patients (X; SD)

	Male	Female
PATIENTS (n = 10)	4	6
AGE (years)	52.7 ± 23.5	59 ± 24.1
BODY MASS INDEX (kg/m2)	23.5 ± 2.9	28.6 ± 14.7
REASON FOR ICU ADMISSION:		
Abdominal or neurologic surgery	3	6
Cardiorrespiratory arrest	1	
INTUBATION (hours)	61.6 ± 2.1	72.3 ± 17.8
APACHE II (%)	33.7 ± 11.8	33.3 ± 8.9
PaO2/FiO2	270.6 ± 62.6	207.5 ± 43.9

**Fig. 15 (abstract A861).**
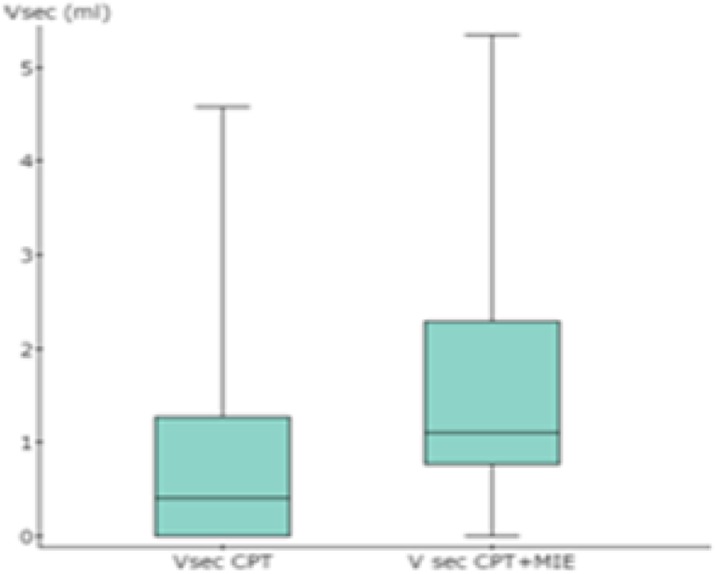
Vol of secretions with or without MI-E

**Table 13 (abstract A861).** Pulmonary mechanics values before, after and 1 hr

	Static compliance (Cst) before (ml/cmH2O)	Cst after	Cst 1 hr after	Respiratory system resistance (Rrs) before (cmH2O/l/s)	Rrs after	Rrs 1 hr after	Airway resistance (Raw) before (cmH2O/l/s)	Raw after	Raw 1 hr after
CPT (X;SD)	53.7 ± 27.15	62.81 ± 45.1	53.71 ± 19.61	17.89 ± 6.54	18.33 ± 6.01	15.81 ± 3.85	2.48 ± 0.68	2.49 ± 0.69	2.14 ± 0.45
CPT + MI-E (X;SD)	58.33 ± 22.2	72.38 ± 32.55	59.87 ± 28.68	17.48 ± 2.75	18.62 ± 3.62	16.31 ± 3.29	2.27 ± 0.62	2.38 ± 0.58	2.19 ± 0.58

### A862 Evaluation of semi-recumbent position compliance in patients with mechanical ventilation: comparison of three data collection methods

#### M. Llaurado-Serra^1^, A. Lobo-Civico^2^, A. Ventura-Rosado^3^, A. Piñol-Tena^4^, M. Pi-Guerrero^5^, C. Paños-Espinosa^6^, M. Peralvo-Bernat^7^, J. Marine-Vidal^7^, R. Gonzalez-Engroba^3^, N. Montesinos-Cerro^2^, M. Treso-Geira^4^, A. Valeiras-Valero^5^, L. Martinez-Reyes^6^, A. Sandiumenge^8^, M.F. Jimenez-Herrera^1^, CAPCRI Study

##### ^1^Rovira i Virgili University, Nursing Department, Tarragona, Spain; ^2^Dr Josep Trueta University Hospital, Intensive Care Unit, Girona, Spain; ^3^Quiron Salud-Hospital General de Catalunya, Intensive Care Unit, Barcelona, Spain; ^4^Verge de la Cinta University Hospital, Intensive Care Unit, Tortosa, Spain; ^5^Hospital de Sant Joan Despí Moissès Broggi, Intensive Care Unit, Barcelona, Spain; ^6^Sant Pau I Santa Tecla University Hospital, Intensive Care Unit, Tarragona, Spain; ^7^Joan XXIII University Hospital, Intensive Care Unit, Tarragona, Spain; ^8^University Hospital Vall d´Hebron, Medical Transplant Coordination Department, Barcelona, Spain

###### **Correspondence:** M. Llaurado-Serra – Rovira i Virgili University, Nursing Department, Tarragona, Spain

**Background:** Semi-recumbent position (SP) is defined as the head-of-bed elevation (HOBE) between 30-45°. Despite being included in guidelines for the prevention of ventilator-associated pneumonia, it is accomplished less than recommended.

**Objectives:** To investigate the factors related to SP from three different perspectives: direct observation, questionnaire and asking the nurse when not complying with the recommendation.

**Methods:** Descriptive, longitudinal and multicenter study in 6 Spanish intensive care units (ICU). Patients were included if they were under mechanical ventilation >48 h, ≥18 years old and had no contraindication for SP, were not mechanically ventilated during the previous 7 days or were not intubated in pre-hospital setting. HOBE was measured 3 times/day with BOSCH GLM80® device. When HOBE was < 30°, the investigator asked the staff nurse the underlying reason. At the end of patients' inclusion, CAPCRI-Q questionnaire was distributed to all ICU staff. Descriptive, bivariate and multivariate analyses were performed according to the source of data. Significance was set at p < 0.05.

**Results:** 276 patients were included (6894 measurements). Mean HOBE was 30.1(6.7)° and 45.9 % of the measurements were < 30° with significant differences among ICUs. In multivariate analysis the most relevant factors related to a decreased SP compliance according to measurements were the ICU(p < 0.001), agitation [OR 0.39 (CI95% 0.28-0.54)], renal replacement therapy [OR 0.56 (CI95% 0.47-0.66)], abdominal vacuum therapy [OR 0.59 (CI95% 0.37-0.95)] and open abdomen [OR 0.65 (CI95% 0.50-0.85)]. From staff nurses, 2146 reasons for non-compliance were obtained. The most frequent causes were incorrect visual perception (n = 568) and that the HOBE measuring device from the bed indicated 30° (n = 460). Patient's clinical conditions represented 33.2 % (n = 742) of the causes for non-compliance whilst factors related to patient care represented 66.3 %(n = 1484). Finally, 223 questionnaires were obtained (77.8 % response rate). According to the results, the most relevant factors with a negative effect to SP compliance were clinical contraindications for SP (98.7 %), patient discomfort (89.2 %), abdominal surgery with open abdomen (85.7 %), unavailability of beds with HOBE measuring device (80.7 %), lack of awareness of the importance of the measure (79.8 %) and lack of experience in critical care (77.6 %).

**Conclusions:** SP compliance is below recommended but the mean HOBE reaches the lower limit of the recommendation. The factors affecting SP compliance differ according to the method used for data collection and include other factors than patient's clinical condition. Politics targeting to increase its compliance should address various areas of care such as team and professionals, resources and equipment and re-consider clinical indications for SP.

**Grant acknowledgement**

The project was funded by the 14° National Award of Nursing Research from Marques de Valdecilla Hospital (Spain).

### A863 A comparison of staff perceptions/attitudes, supportive care standards and patient pathophysiology between the resuscitation room, operating rooms and critical care units of a central London university hospital

#### S. Helyar, P. Riozzi, A. Noon, G. Hallows, H. Cotton, J. Keep, P.A. Hopkins

##### King's College Hospital, ACET Research Team, London, United Kingdom

###### **Correspondence:** P. Riozzi – King’s College Hospital, ACET Research Team, London, United Kingdom

**Introduction:** Critically ill patients presenting to the emergency department (ED) frequently go on to being managed within the operating rooms (OR) and intensive care units (ICU) of the same institution. Here we describe an observational analysis of airway (A), breathing (B), and circulation (C) pathophysiology and supportive care in these three locations to test whether there are differences in practice.

**Objectives:**

1. To compare the perceptions and attitudes of professionals (ED vs OR vs ICU) for supportive care in A, B and C.
2. To compare supportive care standards (ED vs OR vs ICU) in A,B and C.
3. To compare patient pathophysiology, in the first 24 hours of hospital admission, in A,B and C.

**Methods:** Institutional/patient & public approval was gained to collect staff opinions and anonymised patient data. 90 ED, 30 OR and 110 ICU professionals were surveyed following a pilot to determine options, ranking and scoring criteria *a priori* where needed. Anonymous patient data from 36 intubated patients who were cared for in the ED, OR and ICU within their first 24 hours were collected. This included physiological observations and supportive care standards around A, B and C.

**Results:** The most striking differences in staff opinion involved the preferential use of artificial colloid-based fluid resuscitation in sepsis (17 % ED staff; 50 % OR staff; 8 % critical care staff); the value and significance of recording End Tidal CO2 (4 % ED staff; 97 % OR staff; 69 % critical care staff); and the potential preferential use of flow-directed fluid boluses rather than pressure-directed fluid boluses in critically ill patients (22 % ED staff; 90 % anaesthetic staff; 75 % critical care staff). When observing supportive care standards the largest differences were in the use of stress ulcer prophylaxis (only prescribed in critical care); patient positioning (head-up: 94 % patients in CC; 6 % in ED); the recording of sedation level (0 % ED; 6 % anaesthetic; 78 % critical care) and the recording of ventilator parameters-tidal volume, peak pressure and ET-CO2-(3 % ED; 69 % anaesthetic; 100 % critical care) . Finally, in respect to patient pathophysiology, all groups were under ventilated and over oxygenated. Mean arterial pressure was most divergent from baseline in the ED. However, changes in pathophysiology were related to interventions (fluid boluses, analgesia, surgical interventions, inotropes, pressors) rather than location. Despite the divergent views regarding the relative value of flow monitoring, observed fluid boluses were predominantly triggered by pressure changes in all three locations.

**Conclusions:** Differences in staff attitudes; application of standards and patient pathophysiology were identified between care locations. The influence of variation in resources and professional composition of teams (nurses:doctors) on these results requires further work. It remains uncertain whether more uniform approaches would improve patient outcomes.

### A864 Lung comet score (LCS) for evaluation of extravascular lung water (EVLW) in intensive care unit (ICU) patients undergoing renal replacement therapies (RRT)

#### A. Taggu^1^, S. Renuka^2^, S. Sampath^3^

##### ^1^St. Johns Medical College Hospital, Critical Care Medicine, Bangalore, India; ^2^St. Johns Medical College Hospital, Bangalore, India; ^3^St. Johns Medical College Hospital, Bangalore, India

###### **Correspondence:** St. Johns Medical College Hospital, Critical Care Medicine, Bangalore, India

**Introduction:** Lung ultrasound (LUS) is now considered as a valid and fast method that allow quantification and monitoring of EVLW. There is paucity of such studies in ICU patients. Bio-impedance analysis (BIA) is a validated tool for EVLW but is not easily available.

**Objectives:** To test the hypothesis that lung comet score (LCS) is a reliable surrogate of EVLW in ICU patients undergoing RRT.

**Methods:** A prospective observational study was conducted on 450 patients in ICU needing RRT.

Exclusion criteria were Age < 18 years, pregnant, amputees, cardiac pacemakers, pre-existing lung diseases and ascites. Lung comet score as per validated technique , BIA measurements and baseline data were collected pre and post dialysis. Lung comet score and other covariates were fitted into a regression model using BIA as the standard test. Based on BIA delta hydration relative (HS rel), patients were divided into normohydration and hyperhydration using a cut-off of 15 %.

**Results:** A linear regression model in predialysis state showed that only LCS could significantly predict lung water (const 8.79, Coef.0.203, p value 0.00 ). In the postdialysis state LCS perfectly predicted lung water (const 9.98, Coef.,0.261; p value 0.00 ).Bland Altman plots showed good agreement between LCS and hydration status (BIA) pre and post dialysis. The LCS >15 nearly perfectly predicted hydration status in both pre and post dialysis states.

**Conclusions:** Lung comet score is a good surrogate of EVLW and reliably predicts reflects hydration status pre and post dialysis in ICU patients.

**References**

1. Wizemann V, Wabel P, Chamney P. The mortality risk of over hydration in haemodialysis patients. Nephrol Dial Transplant 2009;24:1574–1579.

2. Noble VE, Murray AF, Capp R, Sylvia-Reardon MH, Steele DJ, Liteplo A. Ultrasound assessment for extravascular lung water in patients undergoing hemodialysis. Time course for resolution. Chest 2009;135:1433–9.

**Grant acknowledgement**

None

**Table 14 (abstract A864).** Baseline characteristics

PARAMETERS	normohydration n = 213	hyperhydration n = 237	p -value
Male/Female	190/23	207/30	0.074
Age (years)	50.4 (sd 11.48)	50.95 (sd12.30)	0.811
APACHE II	14.25 (sd 4.8)	14.6 (sd 6.8)	0.082
Body mass index(kg/m2)	20.4 (sd 2.52)	19.95 (sd 6.2)	0.643
Mean Arterial Pressure (mmHg)	90.80 (sd 5.7)	102.52 (sd.6.8)	0.001
Heart Rate (beats/min)	99.8 (sd 7.02)	103.5 (sd 4.8)	0.001
Diabetes mellitus	9.7 %	14.6 %	0.042
Hypertension	14.5 %	16.2 %	0.081
acute febrile illness	21.3 %	14.6 %	0.08

**Table 15 (abstract A864).** Pre and post dialysis comparison of LCS and BIA

Dialysis state	Hydration status (as per BioImpedance Analysis)	LCS (lung comet score) < 15	LCS >15	Fischer exact test
PREDIALYSIS				
	Normohydration	210	0	P = 0.00
	Hyperhydration	3	237	
POSTDIALYSIS	Normohydration	446	0	P = 0.00
	Hyperhydration	0	4

**Fig. 16 (abstract A864).**
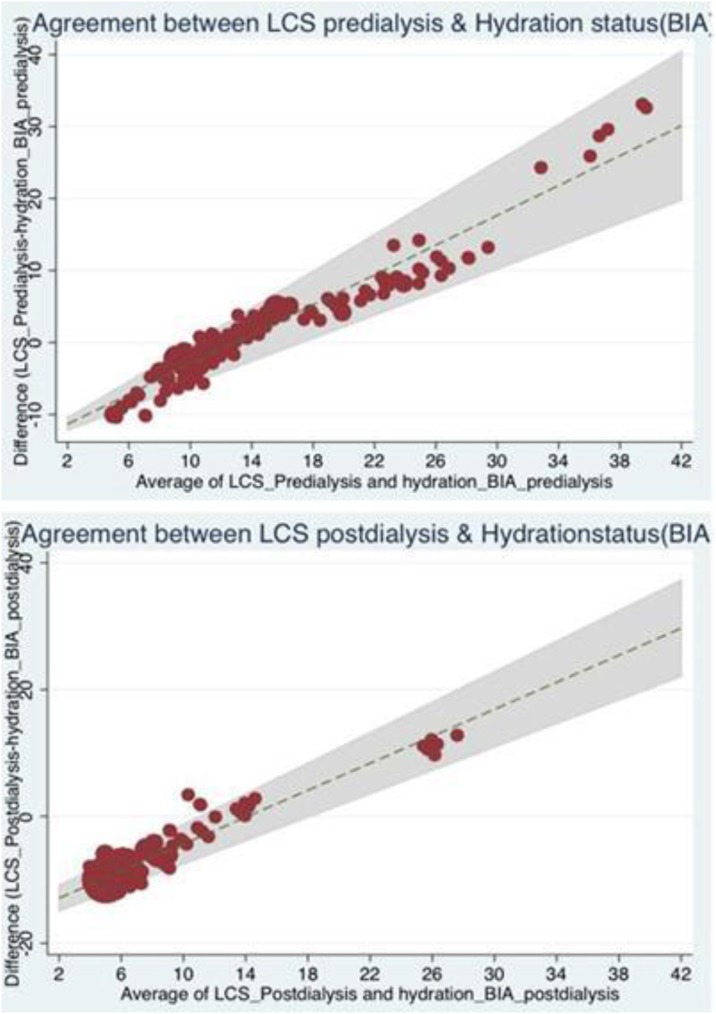
Bland Altman graphs for LCS BIA

### A865 Daily sleep evaluation in the ICU: a one year prospective cohort study

#### P.J.T. Rood, T. Frenzel, R. Verhage, M. Bonn, P. Pickkers, J.G. van der Hoeven, M. van den Boogaard

##### Radboud University Nijmegen Medical Centre, Intensive Care, Nijmegen, Netherlands

###### **Correspondence:** P.J.T. Rood – Radboud University Nijmegen Medical Centre, Intensive Care, Nijmegen, Netherlands

**Introduction:** Sleep is a fundamental need for recovery, and lack of good sleep is associated with adverse effects. ICU patients have increased risk for disturbed sleep architecture. The importance of sleep is internationally recognized. Recently, we determined that the optimal cut-off score for sufficient sleep was a numeric rating score (NRS sleep) of ≥6(1). Subsequently, this sleep NRS was implemented in our ICU practice, supplemented with the Richards Campbell Sleep Questionnaire (RSCQ) when sleep was deemed insufficient.

**Objective:** To determine quality of sleep in daily ICU practice in a large cohort of ICU patients.

**Methods:** A prospective cohort study was performed at the ICU of a university medical center from January to December 2015. In non-comatose patients (RASS −2 to +1), nurses daily asked patients to rate their perceived sleep of the last night on a numeric rating scale of 0–10. When sleep quality was insufficient (NRS sleep < 6), subsequently the RCSQ was performed. Subsequently tailored, non-pharmacological protocolized sleep enhancement interventions were applied, like improving the circadian rhythm by providing stimuli (i.e. light, mobilisation) during daytime, and reducing these (i.e. noise, light, alarms) and clustering of care activities at night. If necessary, medication strategies were added/adjusted. Compliance to the assessments of NRS sleep was measured and defined as compliant when sleep quality was assessed at least once per day after a full night ICU presence.

**Results:** A total of 1603 ICU patients ranked their sleep during their ICU stay. The age was mean 63 ± 14 (mean ± SD), the mean APACHE II score was 16 ± 6, and 62.3 % patients were male. In total 1062 (66 %) were surgical patients, 331 (21 %) medical , and 201 (13 %) were neurological patients. Most patients (77 %) were mechanically ventilated for median 1 day [IQR 1–2]. ICU length of stay was median 1 day [IQR 1–2], and in-hospital stay was median 9 days [IQR 5–17].

The compliance of the sleep measurements was 59 %. Of 4,532 unique daily measurements, 3,199 (71 %) NRS sleep was rated as sufficient by the patients. Overall, the NRS sleep was 6 ± 2. No differences in sleep scores were found between surgical, medical and neurological patients. When sleep was rated as insufficient, multiple RCSQ themes were marked as important factors (Table 16).

**Conclusions:** With this two step-approach of assessing sleep in ICU practice, we determined that 7 out of 10 ICU patients experienced adequate sleep. However, the mean NRS sleep was just at the cut-off value of sufficient sleep which was determined earlier. No differences between specific patients groups were found. Sleep quality and depth of sleep were the most important themes for insufficient sleep measured with the RCSQ.

**References**

1. Rood P, Pickkers P, vander Hoeven J, vanden Boogaard M. Sufficient sleep quality easily measured: a multicenter centre study in dutch ICUS.Intensive Care Medicine Experimental. 2015;3(1):1–2.

**Table 16 (abstract A865).** Measurements of insufficient sleep (NRS sleep <6)

	N = 1949
NRS score, median [IQR]	4 [3–5]
RCSQ theme as important reason for not slepping, n (%)	
Sleep depth,	1539 (79)
Sleep Latency	987 (51)
Awakenings	1186 (61)
Returning to sleep	1156 (59)
Sleep quality	1616 (83)

### A866 Nurse-performed chest ultrasonography for the localization of central venous catheter and detection of post-procedural malpositions and complications as an alternative to chest radiography

#### F. Corradi, L. Melnyk, F. Moggia, R. Pienovi, G. Adriano, C. Brusasco, L. Mariotti, M. Lattuada

##### Ente Ospedaliero Ospedali Galliera, Anestesia e Rianimazione, Genova, Italy

###### **Correspondence:** L. Melnyk – Ente Ospedaliero Ospedali Galliera, Anestesia e Rianimazione, Genova, Italy

**Introduction:** Central venous catheterization (CVC) is considered commonplace in the care of critically ill patients. It has been demonstrated that ultrasounds performed by an expert physician can detect accurately CVC´s mechanical complications and misplacements^1^. By contrast, there is still no consensus regarding their diagnostic power in the identification of silent CVC complications, when performed by a trained nurse.

**Objectives:** To determine the usefulness of nurse-performed ultrasound to evaluate CVC misplacements and detection of pneumothorax (PNX), thus obviating post-procedural radiograph (CXR). After the insertion of a central venous catheter, CXR is usually obtained to ensure correct positioning of the catheter tip and to detect post-procedural complications. Recently published current guidelines for appropriate use of bedside general and cardiac ultrasonography in the evaluation of critically ill patients, suggest a detailed post-cannulation ultrasound examination to confirm CVC location and exclude PNX in adult patients^2^.

**Methods:** A prospective study of 47 CVC procedures, was conducted in an adult mixed intensive care unit. At the end of the procedure, a B-mode ultrasonography was first performed to assess catheter position and detect PNX. Then, contrast enhanced ultrasonography (CEUS) was used to facilitate visualization of catheter tip^1^, avoiding unknown right atrium positioning or artifacts. A post-procedural CXR was obtained for all patients and was considered as a reference technique

**Results:** Internal Jugular Vein malposition was detected in two patients by B-mode ultrasonography and right atrium positioning was detected in 4 patients by CEUS. CEUS yielded a 100 % sensitivity and 98 % specificity in detecting catheter misplacement. k value between the two methods was 0.91 (p < .001). PNX was detected in one patient by ultrasonography and in none by CXR. The mean time required to perform ultrasonography plus CEUS was 10 ± 3 mins vs. 25 ± 8 mins for CXR (p < .001).

**Conclusions:** The close concordance between CEUS and CXR justifies the use of nurse-performed sonography to ensure the correct positioning of the catheter tip and to detect PNX after CVC cannulation to optimize use of hospital resources and minimize time consumption and radiation. CXR will be necessary when sonographic examination is impossible to perform by technical limitations.

**References**

1) Ultrasound localization of central vein catheter and detection of postprocedural pneumothorax: an alternative to chest radiography. Vezzani A et al. Crit Care Med. 2010 Feb;38(2):533–8 2) Guidelines for the Appropriate Use of Bedside General and Cardiac Ultrasonography in the Evaluation of Critically Ill Patients-Part I: General Ultrasonography. Frankel HL et al. Crit Care Med. 2015 Nov;43(11):2479–502

### A867 The art of nurse-family communication: a qualitative descriptive study of how nurses initiate and manage communication with families during treatment withdrawal processes in intensive care

#### M.J. Bloomer^1,2^, M. Coombs^3^, K. Ranse^4^, R. Endacott^5,6,7^

##### ^1^Deakin University, School of Nursing and Midwifery, Melbourne, Australia; ^2^Deakin University, Centre for Quality and Patient Safety, Melbourne, Australia; ^3^Victoria University, Wellington, Graduate School of Nursing Midwifery and Health, Wellington, New Zealand; ^4^University of Canberra, Faculty of Health, Canberra, Australia; ^5^Royal Devon and Exeter Hospital, Royal Devon and Exeter Clinical School, Exeter, United Kingdom; ^6^Plymouth University, Faculty of Health and Human Sciences, Plymouth, United Kingdom; ^7^Monash University, School of Nursing and Midwifery, Melbourne, Australia

###### **Correspondence:** M.J. Bloomer – Deakin University, School of Nursing and Midwifery, Melbourne, Australia

**Introduction:** Treatment withdrawal in intensive care is common^(1)^. Whilst significant research attention has focused on how treatment is withdrawn and what information is communicated to families^(2)^, little is known about how critical care nurses initiate and manage family communication needs at this time.

**Objectives:** The purpose of this study was to explore how nurses initiate and manage communication with family during treatment withdrawal in the ICU.

**Methods:** A qualitative descriptive approach was used. Semi-structured focus group interviews were conducted with adult critical care nurses from four Intensive Care Units, two in Australia and two in New Zealand.

**Results:** Twenty-one critical care nurses participated in the study. The focus groups ranged from 39–58 minutes. Participants had a mean of 13 years experience in critical care and all had experience of treatment withdrawal and providing end-of-life care. Inductive content analysis was used to identify five key themes. READING THE ROOM describes the need to look for non-verbal and other cues to help guide initiating communication. ESTABLISHING THE WHO was seen as important in building rapport and aided in establishing trust. NAVIGATING HOW to communicate was considered just as important as the content. Similarly, JUDGING WHEN to communicate was equally essential to aid family understanding and coping. ASSESSING WHAT to say was individually tailored to each family. In terms of WHERE, participants reported that the art of nurse-family communication was learnt on the job.

**Conclusions:** Initiating and managing communication with family during treatment withdrawal is a complex and multi-faceted nursing activity that can have significant impact on families. There is need for support and education that develops both the art, as well as the science, in this frequently encountered aspect of end-of-life care.

**References**

1. Bloomer, M.J., et al., *End of life management of adult patients in an Australian metropolitan intensive care unit: a retrospective observational study.* Australian Critical Care, 2010. **23**(1): p. 13–19.

2. Truog, R.D., et al., *Recommendations for end-of-life care in the intensive care unit: a consensus statement by the American College of Critical Care Medicine.* Critical Care Medicine, 2008. **36**(3): p. 953–963.

**Grant acknowledgement**

This study was partially funded by an Experience Researcher Grant from the Australian College of Critical Care Nurses, Australia

### A868 Taper-shaped endotracheal cuffs in the prevention of endotracheal tube-associated pneumonia (ETAP): a systematic review and meta-analysis of randomized controlled clinical trials

#### B. Maertens^1^, K. Blot^1^, S. Blot^2^

##### ^1^Ghent University, Faculty of Medicine and Health Sciences, Ghent, Belgium; ^2^Ghent University, Dept. of Internal Medicine, Ghent, Belgium

###### Correspondence: B. Maertens – Ghent University, Faculty of Medicine and Health Sciences, Ghent, Belgium

**Introduction:** Micro-aspiration of subglottic secretions is considered a major pathogenic mechanism of endotracheal tube-associated pneumonia (ETAP), either postoperative pneumonia or ventilator-associated pneumonia. Endotracheal tubes (ETs) with taper-shaped cuffs have been proposed to provide a better seal of the extraluminal airway, thereby preventing micro-aspiration and possibly ETAP.

**Objectives:** To perform a systematic review and meta-analysis to assess the efficacy of ETs with taper-shaped cuffs in the prevention of ETAP.

**Methods:** A systematic search of MEDLINE, EMBASE and CENTRAL/CCTR was conducted in March 2016. Eligible trials were randomized controlled clinical trials (RCTs) comparing taper-shaped cuffs with standard, cylindrical-shaped cuffs in intubated patients. All studies reporting the incidence of ETAP were included. Inclusion of trials was irrespective of publication status, date of publication or language. Random-effects meta-analysis calculated the risk ratio (RR) and 95 % confidence interval (CI) for the incidence of ETAP between both groups using the Mantel-Haenszel method.

**Results:** Three RCTs, given a total of 855 patients, met the inclusion criteria. One trial was published as a conference abstract only *(1),* while the others were published in full *(2,3)*. None of the trials was blinded for the intervention. 440 patients were allocated to the intervention arm and 415 to the control arm. 87 ETAP episodes occurred in the intervention group and 83 in the control group. The pooled RR for the incidence of ETAP was 1.01 (95 % CI, 0.78-1.31; z = 0.11 p = 0.91).

**Conclusions:** The use of endotracheal tubes with taper-shaped cuffs did not show to reduce the incidence of ETAP. However, the number of available studies is small, and there is an inherent risk of bias due to the unblinded designs.

**References**

1. Saito N, et al. 33rd International Symposium on Intensive Care and Emergency Medicine 2013

2. Philippart F, et al. Am J Respir Crit Care 2015

3. Monsel A, et al. Anesthesiology 2016

**Grant acknowledgement**

We received no grants for this study.

**Fig. 17 (abstract A868).**
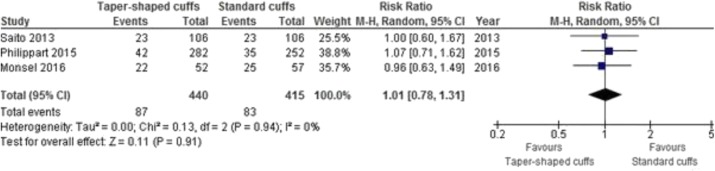
Meta-analysis Forest plot; incidence of ETAP

### A869 Predictability of nursing workload of patients admitted to the ICU after IHCA/OHCA, assessed with the nursing activities score (NAS) in a university hospital

#### M.P. van Nieuw Amerongen^1^, E.S. van der Heiden^1^, J.W.R. Twisk^2^, A.R.J. Girbes^1^, J.J. Spijkstra^1^

##### ^1^VU University Medical Center, Intensive Care, Amsterdam, Netherlands; ^2^VU University Medical Center, Epidemiology and Biostatistics, Amsterdam, Netherlands

###### **Correspondence:** M.P. van Nieuw Amerongen – VU University Medical Center, Intensive Care, Amsterdam, Netherlands

**Background:** Understaffing of ICU's can have serious adverse consequences both for patients and for nurses, and therefore it is important to have an adequate number of nurses on the ward. Nurses however are in short demand and resources are scarce. Being able to predict the nursing workload for a certain group of patients may help to allocate nursing capacity as efficiently as possible and thus to reduce costs, without endangering the patients safety and nurses' health. NAS is a validated tool for the measurement of nursing workload in an intensive care unit.

Goal: This study was conducted to investigate whether it is possible to predict the nursing workload for a homogeneous group of patients, admitted after an in or out of hospital cardiac arrest and to assess the effects of baseline characteristics, vital parameters and admittance time on this workload.

**Method:** We performed a retrospective analysis of NAS scores of all IHCA and OHCA patients admitted to our ICU from October 2012 until September 2015 during the first 48 hours of stay. The NAS was recorded per patient per nursing shift. We furthermore recorded patient characteristics and vital parameters.

**Results:** During this period 386 patients, 273 males and 113 females, were admitted to the ICU after cardiac arrest. The mean age at admission was 63.4 years (SD = 14.8). The mean NAS at admission was 71.4 (SD = 22.4). Patients admitted in the evening shift had a significantly higher NAS compared to patients admitted in the night shift (74.3; SD = 21.2 vs 67.4; SD = 22.3 (p = 0.037)), but no significant difference was found with the day shift (mean NAS day shift: 71.7; SD = 23.3). After admission the workload decreased in all patients by a mean of 18.3 points (SD = 23.9; p < 0.001). A higher SOFA score, a higher PEEP and a lower pH at admittance resulted in a higher NAS score on average over time (p < 0.001).

**Conclusions:** The nursing workload at admission of patients after cardiac arrest is fairly predictable, with no clinically significant difference between shifts , necessitating a nurse-to-patient ratio of at least 1:1. After the first shift it is almost always possible to decrease the nurse-to-patient ratio to 1:2.

The NAS was influenced by severity of illness. These results can be used to assess the needed nursing staff for the treatment of these patients for the first days after admittance.

**References**

Armstrong E et al. Using Nursing Activities Score to Assess Nursing Workload on a Medium Care Unit. Anesthesia and Analgesia. 2015;121:1274–1280

McGahan M et al. Nurse staffing levels and the incidence of mortality and morbidity in the adult intensive care unit: A literature review. Australian Critical Care.2012;25:64–77

Grillo Padilha K et al. Nursing Activities Score in the intensive care unit: Analysis of related factors. Intensive and Critical Care Nursing. 2008;24:197–204

**Grant acknowledgement**

None.

### A870 A comparison of the epidemiology, pathophysiology and clinical management of intubated patients treated in a central london emergency department resuscitation room with major trauma versus severe sepsis/septic shock

#### P. Riozzi^1^, S. Helyar^1^, H. Cotton^1^, G. Hallows^1^, A. Noon^1^, C. Bell^2^, K. Peters^2^, A. Feehan^2^, J. Keep^1^, P.A. Hopkins^1^

##### ^1^King's College Hospital, ACET Research Team, London, United Kingdom; ^2^King's College Hospital, Audit Team, King's Critical Care, London, United Kingdom

###### **Correspondence:** P. Riozzi – King's College Hospital, ACET Research Team, London, United Kingdom

**Introduction:** Major trauma and severe sepsis are both leading causes of admission to the resuscitation rooms in emergency departments across the world. Despite obvious differences in precipitating mechanism, there are surprising similarities between subsequent pathophysiology: both disorders lead to disorders of the macrocirculation, microcirculation and host inflammatory response (1,2).

**Objectives:** Here we compare the baseline epidemiology, pathophysiology, operational and clinical management of intubated resuscitation room patients with these two critical illness syndromes (Major Trauma/Septic shock). The results will be used to facilitate the design and planning of a study to test the feasibility/effectiveness of advanced monitoring systems (thromboelastography, oesophageal doppler flow monitoring, echocardiography, and microcirculatory monitoring) in the resuscitation room management of critically ill patients with these conditions.

**Methods:** Institutional approval was gained to collect anonymised patient data over a 6-month period from a mixture of written and electronic records. Where appropriate, significance was tested by Mann Whitney U (Sigmaplot 11.0).

**Fig. 18 (abstract A870).**
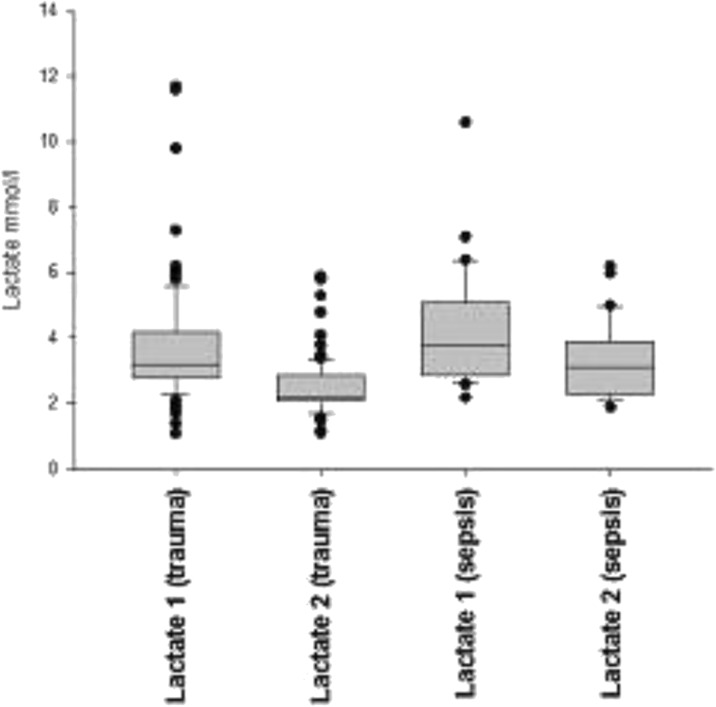
Lactate (Sepsis vs Trauma)

**Results:** 162 patients, intubated pre-hospital or in ED resus, were identified with trauma or sepsis diagnoses. Trauma patients were commoner (n = 128; 79 %) and more likely to be intubated pre-hospital (107/128; 83.6 % vs 27/34; 79 %). Lactate profiles were similar in the two groups at start and end of resus episode (Figure 18). Patients with major trauma were more hypertensive but equally tachycardic when compared with patients with severe sepsis/septic shock. Patients with septic shock/severe sepsis (median 202 minutes vs 128 minutes) spent longer in resuscitation room, but received less documented consultant-level review (90.6 % vs 20.6 %). Imaging of major trauma patients was with CT (128/128; 100 %) and ultrasound (44/128; 34.3 %) in contrast to septic patients (11/34 CT; 32.4 %; 2/34, 1 % US). ICU and hospital mortality was higher in patients with sepsis (38.2 % vs 14.8 %), but death in resus only occurred in the trauma population. Only two patients, both with facial trauma, would have had a relative contraindication to the proposed advanced monitoring.

**Conclusions:** This novel preliminary work has highlighted some important differences between the epidemiology, outcomes, pathophysiology and clinical/operational management of intubated patients with severe sepsis versus major trauma. These will influence the conduct and outcome measures of any trial of advanced monitoring in this setting. However, contraindications to any of the advanced monitoring technologies being considered were rare and no obvious barriers to the planned study of advanced monitoring were identified.

**References**

(1) Lord JM et al. 2014. The systemic immune response to trauma: an overview of pathophysiology and treatment. Lancet 384: 1455–65.

(2) Dewar D et al. 2009. Post injury multiple organ failure. Injury 40: 912–8.

### A871 Impact of an education programme for novice nurses on staff work engagement in a UK ICU

#### K. Churchill^1^, K. Hawkins^1^, R. Brook^1^, N. Paver^1^, R. Endacott^2,3^

##### ^1^Royal Devon and Exeter Hospital, ICU, Exeter, United Kingdom; ^2^Plymouth University/Royal Devon and exeter Hospital Clinical School, Exeter, United Kingdom; ^3^Monash University, School of Nursing and Midwifery, Melbourne, Australia

###### **Correspondence:** K. Churchill – Royal Devon and Exeter Hospital, ICU, Exeter, United Kingdom

**Introduction:** In our 15-bedded GICU, demand for beds has increased while recruitment of ICU trained nurses has decreased. There is enduring evidence of links between workload and stress [1, 2], with high levels of burnout reported in ICU nurses [3]. There has been a shift towards measuring what makes people positive and engaged [4], rather than why people reach the extreme state of burn out. It is important to understand factors that affect work engagement to develop strategies that enhance nurse retention and improve the quality of ICU patient care.

**Objectives:** To examine the impact of an education initiative for novice ICU nurses on work engagement for the ICU nursing staff and organisational resource use.

**Methods:** A pre - post design was used to collect data from all ICU nurses at the start of the education programme and at 6 months following he intervention. Work engagement was measured using the self-report 17 item Utrecht Work Engagement Scale (UWES) [4] with an open question to capture staff experiences. Organisational impact was measured using levels of sick leave, % staff turnover, use of agency nurses and staff recruitment.

**Results:** Fifty three ICU nurses completed the pre-intervention survey (61 % response) and 42 completed the post-intervention survey (46 % response). Respondents had reasonable years of ICU experience (Mean7.57, SD 8.1) and time in current post (Mean6.21, SD 8.2). Internal consistency for the UWES was high (alpha .91). Levels of work engagement (Mean [SD]) increased (3.94, [0.64] vs 4.03 [0.69]) but did not reach significance and remained in the 'average' band as judged by the scale authors [4]. When examined by senior and junior nurses, the increase was similar. Organisational measures showed decrease in sick leave, turnover, agency use and increase in recruitment of experienced ICU nurses. Qualitative feedback was positive, with perceptions of improvement in unit morale due to time being invested in the individual and reduced stress and workload for shift leaders.

**Conclusions:** Providing education for the newest ICU recruits can have benefits for the whole ICU team. However, it is important to examine how work engagement might be further improved.

**References**

1. Schaufeli W, et al. In : Miranda DR, et al. (eds) *Organisation and Management of Intensive Care : a prospective study in 12 European countries*. 1998; Springer, Berlin

2. Gurses AP et al. Impact of performance obstacles on intensive care nurses' workload, perceived quality and safety of care and quality of working life. *Health Services Research* 2009; 44(2): 422–443

3. Poncet MC et al. Burnout syndrome in critical care nursing staff. *Am J Resp Crit Care Med* 2007; 175: 698–704

4. Bakker, A. B. et al. Work engagement: An emerging concept in occupational health psychology. *Work and Stress* 2008, 22(3), 187–200.

**Grant acknowledgement**

None

### A872 The incidence of silent aspiration on intensive care

#### N. Maistry

##### Royal Brompton and Harefield NHS Foundation Trust, Rehabilitation and Therapies, London, United Kingdom

**Introduction:** The incidence of Dysphagia on intensive care is an area of growing research. Dysphagia is associated with aspiration pneumonia and increased ICU bed days. In general, Speech and Language Therapy (SLT), makes recommendations based on the results of a clinical bedside swallowing evaluation, despite the unreliability of this method 1. This is largely due to the difficulty accessing gold standard assessment methods such as Videofluoroscopy (VF) and Fibreoptic Endoscopic Evaluation of Swallowing (FEES)3. Referral for these assessment methods are based on a local defined criteria. This study evaluates the incidence of silent aspiration identified by VF and FEES in a 20 bedded tertiary Cardio-Respiratory Intensive Care Unit.

**Objectives:** To determine the incidence of silent aspiration, defined as “aspiration before, during, or after swallowing in the absence of cough or visible signs of choking and distress 2," in ICU patients assessed by VF or FEES between July 2014 and June 2015.

**Method:** Data was retrospectively reviewed for 12 month period from all ICU referrals made to SLT for swallowing evaluation. All patients received a clinical bedside swallowing evaluation. The results are presented as percentages and counts for patients receiving VF and FEES that silently aspirated.

**Results:** A total of 97 patients were referred for swallowing assessment and 35 % (34/97) had a VF or FEES. There were 27 males and ages were 44.1 ± 15.7 years. In this group, 22 patients had Videofluoroscopic assessments and 12 patients had FEES. In the VF group 77 % silently aspirated whilst in the FEES group the values were 83 %. 27 patients (79 %) silently aspirated during objective assessment, impacting on how and when oral feeding was commenced.

**Conclusion:** This study suggests that silent aspiration is highly prevalent in this population group. Consequently, VF and FEES should be part of standard routine assessment in the management of critically ill patients.

**References**

1. Romano M, Schultz T, Tai A. Diagnostic Accuracy of clinical swallow assessment for oropharyngeal aspiration: a systematic review. JBI Library of Systematic Reviews, 2012 Vol 10

2. Bruce K. Rubin. The Cruelest Lies Are Often Told in Silence. Chest September 2011, Vol 140, No. 3

3. Jeronimo, B. Critical Review: Is the endoscopic swallowing assessment more sensitive than the videofluoroscopic swallowing assessment at identifying penetration or aspiration in adults with dysphagia 2013

### A873 Delerium related incidents at the ICU and nursing aspects

#### A. van Wijk^1^, N. Rouw^1^, T. van Galen^2^, S. Evelein-Brugman^3^

##### ^1^Senior ICU Nurse Quality and Safety VU University Medical Center, Amsterdam, Netherlands; ^2^ICU Nursing,VU University Medical Center, Amsterdam, Netherlands; ^3^VU University Medical Center, Amsterdam, Netherlands

###### **Correspondence:** A. van Wijk – Senior ICU Nurse Quality and Safety VU University Medical Center, Amsterdam, Netherlands

**Introduction:** At the ICU of VU University Medical Center (VUmc) nurses are frequently confronted with delirium^1,2^. Delirium is known to be present in 60-80 % of mechanical ventilated patients and 20-50 % in non-ventilated patients. Immediate consequences are falling incidents or for patients to remove tubes and IV lines that are necessary for treatment. In literature, this is stated as a result of treatment, but often data is missing. Consequences of removal are increased risk of complications^3^, prolonged mechanical ventilation, LOS and increased morbidity/mortality^4^.

**Objectives:** To measure the frequency of removing tubes, lines and falling incidents related to delirium.

**Methods:** A multidisciplinary focus group was formed (2014) in order to properly diagnose, prevent and/or treat delirium due to the high prevalence. The first steps were increasing awareness and implementing the CAM-ICU score. To clarify delirium-related incidents a one year period was set in which the dedicated senior nurse informed and trained the nursing staff regarding delirium and potential risks. To register delirium-related incidents a modified report button was built in the EPR (Metavision, IMD Soft) and used beside the regular incident reporting system^5^.

**Results:** After one year, 52 individual patient incidents were reported concerning falling or tube or IV line removal. This included 14 gastric tubes, 15 airway tubes, 14 IV/CVC/arterial lines, 5 other lines and 4 fall incidents. In 83 % of the cases the patient was diagnosed with delirium. 4 out of 5 patients received medication or were fixated before the incident despite a 40 % CAM-ICU registration rate. Because the focus group doubted about underreporting 10 nurses were interviewed if the results corresponded with their experience. They were unanimous that there was hardly any underreporting.

**Discussion:** Despite therapy or fixation delirium-related incidents occur on a weekly basis at our ICU, causes harm and increases nursing workload. Although the incidence rate is presumed to be low, there is no feeling of satisfaction. Further improvement is necessary due to the high risks for the patient. Therefore, we need to be able to diagnose incidents faster so we can start treatment sooner. Although the CAM-ICU score was implemented, compliance is insufficient. Increasing compliance is the first step to further improvement. The follow-up question is whether delirium-related injury can be reduced when CAM-ICU compliance improves. Second step is to investigate the effectiveness of our fixation protocol.

### A874 Prospective study to determine the predictors of extubation success

#### A. Taggu^1^, B. Krishna^2^, S. Sampath^3^

##### ^1^St. Johns Medical College Hospital, Critical Care Medicine, Bangalore, India; ^2^St. Johns Medical College Hospital, Bangalore, India; ^3^St. Johns Medical College, Bangalore, India

###### **Correspondence:** A. Taggu – St. Johns Medical College Hospital, Critical Care Medicine, Bangalore, India

**Introduction:** Timely extubation is crucial in critically ill patients. Traditional indices like rapid shallow breathing index are considered as accurate during the spontaneous breathing trial. Multiple other proposed parameters like diaphragm thickness, fluid balance and cardiac indices have been shown to predict succesful extubation in the recent years.

**Objectives:** To assess the reliability of the parameters in predicting successful extubation.

**Methods:** A prospective observational study done on 220 adult patients eligible for extubation as decided by the attending intensivists.

**Exclusion criteria:** Pregnant and tracheostomised patients. Along with baseline parameters, following measurements were taken pre and post extubation.

1. Cardiac parameters including Simpsons method for ejection fraction, E/A, E/e' (lateral) for diastolic function, TAPSE and TAD for Right ventricular function. All recordings were taken just before extubation and within six hours post extubation.
2. Just before extubation,high frequency linear ultrasound probe was used to measure the right sided DT at the zone of apposition (ZOA) between 8^th^ to 10^th^ intercostal spaces in mid-axillary line.The change in DT fraction(Δdtfrac_pre%) was calculated as DT(end-inspiration)-DT(end-expiration)/DT (end-expiration)x100.RSBI was simultaneously recorded.
3. Fluid balance 24 hours were recorded.

**Statistics:** Logistic regression was used to develop a model with extubation failure as the outcome and change in DT(delta fraction), RSBI, E/e', E/A , fluid balance 24 hrs and other patient covariates as predictors. Extubation failure was defined as re-intubation within 48 hrs of extubation.

**Results:** In the logistic model, delta fraction was the only significant predictor of extubation success among all covariates (const 8.985, Coef.-0.2715, p value 0.00). The model showed very good discrimination (receiver operating curve, ROC area of 0.94.4) but poor calibration (Hosmer- Lemeshow chi2 (3) = 206.53, Prob > chi2 = 0.000).

**Conclusions:** Among all the parameters studied, diaphragm thickness change just before extubation reliably predicted the extubation success.

**References**

Matamis D, Soilemezi E, Tsagourias M, Akoumianaki E, Dimassi S, Boroli F, Richard JC, Brochard L (2013) Sonographic evaluation of the diaphragm in critically ill patients. Technique and clinical applications. Intensive Care Med 39:801–810

**Table 17 (abstract A874).** Baseline characteristics

Parameters	Extubation success n = 186	Extubation failure n = 34	P value
Age (years)	52.40(sD 16.14)	56.10((sD 16.20)	0.56
Male	108/78	25/9	0.60
Rapid shallow breathing index- median( IQR)	48(39–82)	54(40–82)	0.06
Mean arterial pressure	69.4((sD2.46)	67.56((sD2.78)	0.34
Mechanical ventilation days-median (IQR)	4(6–11)	7(9–14)	0.49
Fluid balance 24 hrs-median (IQR) ml	110(1200–760)	320(900–1500)	0.05
E/e’ (lateral)	7.5((sD 3.8)	9.2((sD3.16)	0.001
E/A	1.92((sD1.2)	1.10((sD0.98)	0.001
Delta fraction of diaphragm thickness	46.35((sD11.7)	30.4((sD12.4)	0.000

**Table 18 (abstract A874).** Logistic regression diaphragm thickness fraction

Delta fraction (Diaphragm thickness change)	Extubation success	Extubation failure	Significance
>25 %	183(98.4 %)	2(5.8 %)	Fisher’s exact test
<25 %	3(1.6 %)	32(94.2 %)	p = 0.000
Total	186	34	220

**Fig. 19 (abstract A874).**
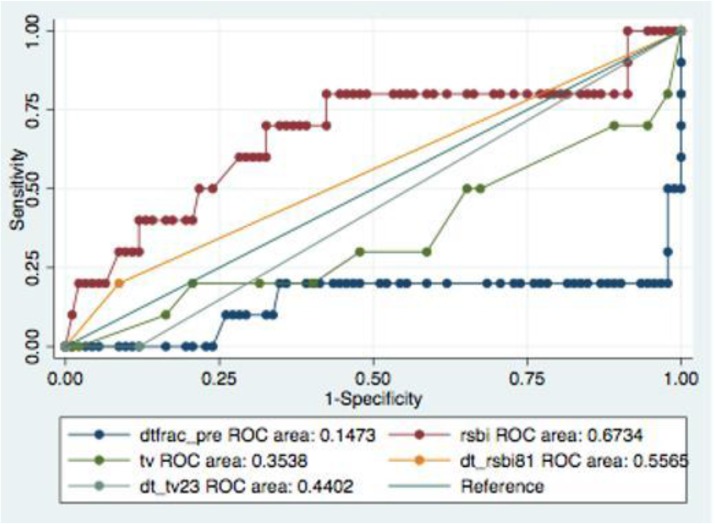
ROC curves of variables predicting extubation

**Grant acknowledgement**

None

## ADVANCES IN GENERAL INTENSIVE CARE

### A875 Blood purification with continuous venovenous hemofiltration in septic and ARDS patients: a meta-analysis of randomized trials

#### A. Putzu^1^, M. Fang^2,3^, M. Boscolo Berto^1^, A. Belletti^2^, T. Cassina^1^, L. Cabrini^2^

##### ^1^Fondazione Cardiocentro Ticino, Cardiac Anesthesia and Intensive Care, Lugano, Switzerland; ^2^IRCCS San Raffaele Scientific Institute, Department of Anesthesia and Intensive Care, Milan, Italy; ^3^3rd Hospital of HeBei Medical University, Department of Intensive Care Medicine, Shi Jiazhuang, China

###### **Correspondence:** A. Putzu – Fondazione Cardiocentro Ticino, Cardiac Anesthesia and Intensive Care, Lugano, Switzerland

**Introduction:** Severe inflammatory conditions, as sepsis and acute respiratory distress syndrome (ARDS), are common among critically ill patients. Despite healthcare improvement, mortality still remains high and few therapies are nowadays available. Attenuating inflammation through direct removal of inflammatory mediators from circulation appears to be intuitively. Theoretically, continuous venovenous hemofiltration (CVVH) could remove part of the inflammatory mediators and/or bacterial toxins.

**Objectives:** The objective of this systematic review and meta-analysis of randomized trials was to evaluate if CVVH reduces mortality in critically ill patients with sepsis or ARDS.

**Methods:** PubMed, EMBASE, and the Cochrane Central Register were searched for pertinent studies (last updated February 1^st^, 2016). We included randomized trials on the use of CVVH as blood purification technique in comparison to standard therapy in adult critical ill patients with sepsis or ARDS. Trials in which primary indication for CVVH was acute kidney injury needing renal replacement therapy were excluded. Primary endpoint was mortality at longest follow-up available.

**Results:** Ten studies published between 2002 and 2015, randomizing 623 patients, were included in the analysis. The included trials had moderate to high risk of bias.

**Fig. 20 (abstract A875).**
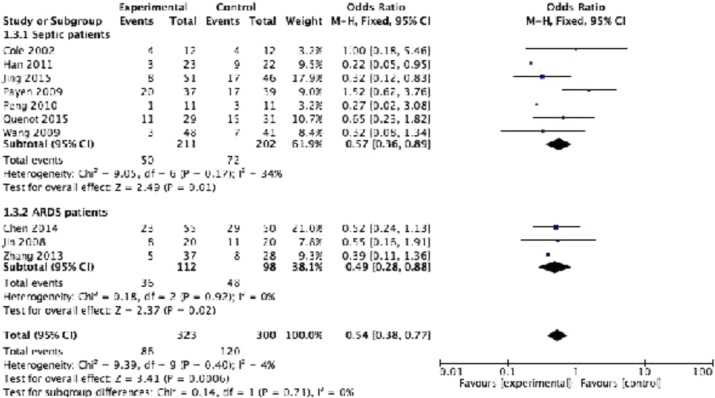
Forest plot for the risk of mortality at longest follow up available

Patients who received blood purification with CVVH showed significantly lower mortality compared with those who received conventional therapy (86/323 [26.63 %] patients in the CVVH group vs. 120/300 [40 %] in the conventional therapy group, OR 0.54 [95 % CI, 0.38, 0.77], *p* for effect = 0.0006, I^2^ = 4 %, Number Needed to Treat = 8) (Figure 20).

The results were confirmed when including only studies performed in a septic setting (7 trials and 413 patients, OR 0.57 [95 % CI, 0.36, 0.89], *p* for effect = 0.01, I^2^ = 34 %) or in ARDS patients (3 trials and 210 patients, OR 0.49 [95 % CI, 0.28, 0.88], *p* for effect = 0.02, I^2^ = 0 %) (Figure 20). We found no significant difference on mortality when limiting the results just to trial employing CVVH at hemofiltration rate lower or higher then 50 ml kg^−1^ h^−1^.

**Conclusions:** Blood purification with CVVH might be associated with a significant reduction in mortality when performed in patients with sepsis or ARDS. This is the first meta-analysis suggesting beneficial effects of CVVH on mortality and we could suppose that the beneficial effects of CVVH in these inflammatory conditions could arise from the immunomodulatory properties of hemofiltration. Further high-quality randomized controlled trials adequate powered for mortality are needed to clarify the impact of CVVH on these inflammatory conditions.

**Grant acknowledgement**

The authors declare no support or funding and no potential conflict of interest.

### A876 Clinical significance of circulating nucleosomes in disseminated intravascular coagulation

#### M. Mistry^1^, Y. Alhamdi^2^, I. Welters^2,3^, S.T. Abrams^2^, C.H. Toh^2^

##### ^1^University of Liverpool, Institute of Ageing and Chronic Disease, Liverpool, United Kingdom; ^2^University of Liverpool, Institute of Infection and Global Health, Liverpool, United Kingdom; ^3^Royal Liverpool University Hospital, Liverpool, United Kingdom

###### **Correspondence:** M. Mistry – University of Liverpool, Institute of Ageing and Chronic Disease, Liverpool, United Kingdom

**Introduction:** Disseminated intravascular coagulation (DIC) is a life-threatening condition that occurs secondary to many underlying diseases which initiate a massive inflammatory response. This damages the microvascular endothelium and ultimately leads to increased cellular damage. During cell death, chromatin is cleaved into nucleosomes, which are released extracellularly and further degraded into individual histones and free DNA (1). Recent studies have shown circulating histones can be extremely toxic by increasing thrombin generation, platelet aggregation and endothelial damage. Nucleosomes are also elevated in numerous critical illnesses associated with the development of DIC.

**Objectives:** This study was designed to elucidate the role of nucleosomes in DIC and to analyse their potential value for diagnosing DIC and predicting patient outcome.

**Methods:** A retrospective analysis of 170 patients admitted to the intensive care unit (ICU) of Royal Liverpool University Hospital (Liverpool, UK) was performed. Citrated blood samples were collected for four days after ICU admission and centrifuged to obtain plasma. Routine coagulation markers were measured. Nucleosomes were measured by enzyme-linked immunosorbent assay. Patients with a score of ≥5 by the ISTH Sub-committee scoring system were diagnosed as having overt-DIC. Statistical analysis was performed using SPSS 22.0 for Windows. Two sided p-values of < 0.05 were considered to be statistically significant.

**Results:** According to the ISTH DIC scoring criteria, 28 out of the total 106 patients (26.4 %) were diagnosed with overt-DIC and 26 patients (24.5 %) were classified as non survivors. The circulating levels of nucleosomes over the four days from intensive care unit (ICU) admission were not significantly elevated in overt-DIC patients compared to ICU controls (*P* = 0.58). There was also no significant difference observed in nucleosome levels between survivors and non survivors, indicating no prognostic value. Nucleosomes did not appear to correlate with any of the clotting tests or variables predictive for disease severity or patient outcome. Nucleosomes also showed little predictive value in the diagnosis of DIC

(AUC = 0.535 (95 % CI 0.405-0.666, *P* = 0.583))

**Conclusions:** Patients with overt DIC do not have elevated plasma levels of nucleosomes. Nucleosomes levels did not appear to change significantly in patients with overt DIC, showing no independent prognostic significance or predictive value in DIC. These findings provide an interesting insight into the differing roles of histones and nucleosomes in DIC pathogenesis; histones may not be toxic when bound in a complex as nucleosomes.

**References**

(1) Holdenrieder S, Stieber P, Bodenmuller H, Fertig G, Furst H, Schmeller N, et al. Nucleosomes in serum as a marker for cell death. Clinical chemistry and laboratory medicine. 2001;39(7):596–605.

### A877 Which factor dose affect the recovery from extreme hyperbilirubinemia in critically ill patients?

#### H.-S. Han^1^, E.M. Gil^2^, D.-S. Lee^2^, C.-M. Park^1^

##### ^1^Samsung Medical Center, Surgery, Seoul, Republic of Korea; ^2^Samsung Medical Center, Critical Care Medicine, Seoul, Republic of Korea

###### **Correspondence:** H.-S. Han – Samsung Medical Center, Surgery, Seoul, Republic of Korea

**Introduction:** Extreme hyperbilirubinemia occurs rarely in critically ill patients. It is usually caused due to hepatic failure or cholestasis with various causes and has a dreadful clinical course without recovery. However, only a few reports are available regarding the clinical course or prognostic factors of extreme hyperbilirubinemia in critically ill patients.

**Objectives:** We evaluated the clinical course and various factors affecting the recovery and survival from extreme hyperbilirubinemia in critically ill patients.

**Methods:** A retrospective study was performed at a single center from 2006 to 2015. We defined extreme hyperbilirubinemia as a state of total bilirubin above 20 mg/dl and selected all patients whose serum total bilirubin increased above 20 mg/dl at least once during their stay in the intensive care unit. We investigated the overall clinical course of the patients and compared the differences between one group with normalization of total bilirubin (recovery group) and the other group without normalization (non-recovery group). Furthermore, we evaluated the association between prognosis and various clinical factors, including the peak total bilirubin levels, increasing rate of total bilirubin (Vi), results of laboratory analyses related to hepatic function, and clinical features at the time of extreme hyperbilirubinemia. These data were analyzed using Chi-square test and Cox and logistic regression analyses.

**Results:** In total, 610 patients experienced extreme hyperbilirubinemia. The mean age was 56.4 ± 14.0 years. The number of recovery group was 46 (7.5 %) and non-recovery group was 564 (92.5 %). A high Vi, young age, and use of hepatotonic agents were identified as influential factors for the recovery from extreme hyperbilirubinemia, whereas obesity, diabetes mellitus, hypertension, and dyslipidemia were the unfavorable factors. The mortality rate was 86.89 % of total patients, and there were no significant differences in mortality rate according to the highest level of total bilirubin. The Vi (95 % CI 1.070-1.221, *p* < 0.001), serum level of aspartate aminotransferase (95 % CI 1.000-1.001, *p* = 0.005) and lactate (95 % CI 1.125-1.298, *p* < 0.001), and use of vasoactive drugs (95 % CI 1.014-1.943, *p* = 0.04) were identified as significant risk factors for mortality, whereas the use of hepatotonic agents decreased the mortality risk (95 % CI 0.610-0.887, *p* = 0.001).

**Conclusions:** The mortality rate of patients with extreme hyperbilirubinemia was exceedingly high. This study identified the various factors related to the recovery and mortality from extreme hyperbilirubinemia. We expect that the results of this study will be helpful in treating and predicting the prognosis of patients with extreme hyperbilirubinemia.

**References**

1. Vishal Bansal, MD. et al. Jaundice in the Intensive Care Unit. Surg Clin N Am 86 2006;1495–1502

**Grant acknowledgement**

Thanks for H-R Jung, W-S Han, S-H Jung

### A878 Are intravascular thermoregulation devices associated with the development of venous thrombo-embolic phenomenon?

#### S. Winder-Rhodes, R. Lotay, J. Doyle

##### Royal Surrey County Hospital NHS Foundation Trust, Department of Intensive Care Medicine and Surrey Peri-Operative Anaesthesia and Critical Care Collaborative Research Group (SPACER), Guildford, United Kingdom

###### **Correspondence:** S. Winder-Rhodes – Royal Surrey County Hospital NHS Foundation Trust, Department of Intensive Care Medicine and Surrey Peri-Operative Anaesthesia and Critical Care Collaborative Research Group (SPACER), Guildford, United Kingdom

**Introduction:** Intravascular thermoregulation devices (ITDs) have become increasingly popular in recent years and have proved effective in critically ill patients for achieving targeted temperature management^1^. Previous reports, however, have suggested an association between ITDs and the development of venous thrombosis^2^.

**Objectives:** The aim of this quality improvement initiative was to determine the prevalence of venous thrombosis amongst patients in the 28-bed Intensive Care Unit at the Royal Surrey County Hospital (RSCH, UK) who had received ITDs (Thermoguard XP temperature management system^3^).

**Methods:** Patients with ITDs inserted on the Intensive Care Unit between April 2014 and April 2016 were identified retrospectively from a departmental record of procedures. The electronic patient records, discharge summaries and radiological imaging for each patient were reviewed for evidence of a venous thrombus following ITD insertion.

**Results:** Of twenty-five patients who received ITDs between April 2014 and April 2016, three had subsequent radiological evidence of venous thrombosis. Of these, one had the ITD inserted to achieve therapeutic hypothermia (target 33 °C) and the others, for the prevention of pyrexia (target 37.5 - 38 °C). In all three patients, ITDs were sited in femoral veins. Thrombi were identified in the femoral vein local to the site of ITD placement in one patient and involved the IVC in the other two; one patient later had evidence of pulmonary embolism. Two of the three cases had ITDs in situ for more than 72 hours.

**Conclusions:** At least 12 % of patients managed with ITDs in the Intensive Care Unit at the RSCH (UK) had radiological evidence of venous thrombosis. This association justifies a high index of clinical suspicion and low threshold for investigating venous thrombosis in patients with ITDs, and warrants further exploration in larger patient groups.

**References**

1. Cooling techniques for targeted temperature management post-cardiac arrest. Vaity C, Al-Subaie N, Cecconi M. Critics Care. 2015; 19(1): 103.

2. Thermoregulatory catheter-associated inferior vena cava thrombus. Gierman JL, Shutze WP Sr, Pearl GJ, Foreman ML, Hohmann SE, Shutze WP Jr. Proc (Bayl Univ Med Cent). 2013;26(2):100–2.

3. IVTM Intravascular Temperature Management Catheter Specifications. http://www.zoll.com/uploadedFiles/Public_Site/Products/Catheters/Catheters_spec_sheet.pdf. Accessed April 2016.

### A879 Pro-inflammatory macrophage regulates MMP1 and MMP10 expression in pulmonary arterial hypertension via the ERK and JNK signal pathways

#### M.-W. Ke^1^, W.-C. Huang^1^, C.-H. Chiang^2^, W.-T. Hung^1^, C.-C. Cheng^2^, K.-C. Lin^1^, S.-C. Lin^1^, K.-R. Chiou^2^, S.-R. Wann^1^, C.-W. Shu^3^, P.-L. Kang^2^, G.-Y. Mar^2^, C.-P. Liu^2^

##### ^1^Kaohsiung Veterans General Hospital, Critical Care Division, Kaohsiung City, Taiwan, Province of China; ^2^Kaohsiung Veterans General Hospital, Cardiovascular Division, Kaohsiung City, Taiwan, Province of China; ^3^Kaohsiung Veterans General Hospital, Department of Medical Education and Research, Kaohsiung City, Taiwan, Province of China

###### **Correspondence:** C.-H. Chiang – Kaohsiung Veterans General Hospital, Cardiovascular Division, Kaohsiung City, Taiwan, Province of China

**Introduction:** Pulmonary arterial hypertension (PAH) is a fatal disease characterized by vascular remodeling of pulmonary arteries. The remodeling is associated with abnormal proliferation of pulmonary arterial smooth muscle cells (PASMCs), deposition of extracellular matrix proteins and perivascular inflammation. Matrix metalloproteinases (MMPs) are critical to the maintenance of the homeostasis of extracellular environment.

**Objectives:** The goal of this study is to explore the role of MMP1 and MMP10 in PAH, and to investigate the molecular mechanisms involved in the regulation of MMP1 and MMP10 expression.

**Methods:** This study first compared the expression levels of MMP1 and MMP10 in the serum between healthy donors and PAH patients. The primary cultured human monocyte-derived macrophages from PAH patients or healthy donors were subjected to the induction of MMP1 or MMP10 expression by LPS/IFNg stimulation. The expression of mRNA and protein of MMP1 or MMP10 was analyzed by semi-quantitative PCR and western blot, respectively. The human monocyte cell line, THP-1, in combined with chemical inhibitors, including PD98059, LY294002, SB203580 and SP600125, were used to investigate the signaling pathways involved in the regulation of MMP1 and MMP10 expression. For *in vivo* study, the lung specimens from monocrotaline (MCT) induced PAH rat were examined for the expression of MMP1 and MMP10 by semi-qPCR and immunohistochemistry, respectively.

**Results:** In human serum, the level of MMP1 and MMP10 is significantly higher in PAH patients. The elevated expression MMP1 and MMP10 was confirmed by *in vitro* cultured primary human macrophages. Under the stimulation of LPS/IFNg, macrophages derived from PAH patients showed robust upregulated MMP1 and MMP10 expression compared to healthy donors. The human THP-1 cell line, which was polarized by LPS/IFNg stimulation, provided more convincible data of the up-regulation of MMP1 and MMP10 expression in pro-inflammatory macrophages. Following the treatment of chemical inhibitors, the expression of MMP1 and MMP10 was significantly suppressed when the ERK signaling or JNK signaling was interfered, while the inhibition of p38 or PI3K signaling had no effects. For *in vivo* evidence, the expression level of MMP1 and MMP10 protein was critically elevated in the lung tissue of MCT-induced PAH rats compared to the PBS control group. Immunohistochemical staining further revealed the co-localization of MMP1 or MMP10 molecule with the COX-2-positive macrophages, which meant the expression of MMP1 and MMP10 was highly associated with the pro-inflammatory macrophage phenotype.

**Conclusions:** MMP1 and MMP10 were significantly up-regulated in pro-inflammatory macrophages *in vitro* and *in vivo* in both primary cultured human macrophages, THP-1 cell line and MCT-induced PAH rat model. Blockade of JNK and ERK signaling pathways may be a novel strategy for the treatment of PAH.

**Grant acknowledgement**

None.

### A880 An early rise in central venous pressure (CVP) predicts weaning failure during a spontaneous breathing trial: preliminary results of the 4P study

#### S. Dubó^1^, A. Aquevedo^2^, M. Jibaja^3^, D. Berrutti^4^, C. Labra^5^, R. Lagos^5^, M.F. García^3^, V. Ramirez^3^, M. Tobar^3^, F. Picoita^3^, C. Peláez^3^, D. Carpio^5^, L. Alegría^5^, C. Hidalgo^6^, K. Godoy^6^, J. Bakker^5^, G. Hernández^5^

##### ^1^Universidad de Concepción, Facultad de Medicina, Departamento de Kinesiología, Concepción, Chile; ^2^Hospital Dr. Sótero del Río, Unidad de Pacientes Críticos, Santiago, Chile; ^3^Hospital Eugenio Espejo, Unidad de Cuidados Intensivos, Quito, Ecuador; ^4^Universidad de la República, Hospital de Clínicas, Centro de Terapia Intensiva, Montevideo, Uruguay; ^5^Pontificia Universidad Catolica de Chile, Facultad de Medicina, Departamento de Medicina Intensiva, Santiago, Chile; ^6^Hospital Guillermo Grant Benavente, Concepción, Chile

###### **Correspondence:** G. Hernández – Pontificia Universidad Catolica de Chile, Facultad de Medicina, Departamento de Medicina Intensiva, Santiago, Chile

**Background:** Prediction of weaning failure through a spontaneous breathing trial (SBT) is a major task for intensivists since a failed weaning attempt exposes the patient to the risk of cardiovascular and respiratory complications, among others, that could increase morbidity or mortality. Several tests have been used to assess risk incorporating ventilatory mechanics and drive, and some cardiovascular parameters with acceptable predictive values. However, all have limitations and in general are more centered in ventilatory aspects. We hypothesized that a multimodal monitoring during a SBT addressing the capacity of the heart to manage increased venous return as reflected by CVP changes, metabolic effort as reflected by changes in central venous O_2_ saturation (ScvO_2_), and adrenergic stress as reflected by changes in capillary refill time (CRT) during a 1 or 2 h T-tube trial could predict weaning failure in critically ill patients.

**Objective** The aim of the study was to evaluate changes in CVP, ScvO_2_ and CRT during a SBT and their association to weaning failure.

**Methods:** Prospective multicenter observational study including patients in mechanical ventilation for at least 24 h for any cause, excluding only neurological or DNR patients. Usual clinical parameters and the study variables were recorded at baseline, 2, 30, and 120 min during the T-tube trial. Peripheral perfusion was evaluated with CRT and mottling score.

Attending physicians were informed about changes in CVP, ScvO_2_ and CRT during SBT but decisions about extubation after the test were taken by them without any written guideline and based only in their usual practice. Weaning failure was defined as need of reintubation in less than 48 h after extubation. The results of a first interim analysis after completing half of the sample size calculation are presented here. Statistical analysis included t-test.

**Results:** A total of 114 patients from 5 centers were enrolled (age: 53 ± 22 y, APACHE II: 19.7 ± 9.6), and 93 pts were extubated after the SBT. Weaning failure occurred occurred in 7 (8 %). Usual clinical parameters such as HR, RR, SBP, and FiO_2_ were not different between successful vs failed weaning procedures. Reintubated patients presented an early (2 min) significant increase in CVP (9 to 11.8 mmHg; p 0.007) and a late (120 min) decrease in ScvO_2_ (60 % vs 43 %; p 0.05), as compared to succesfully weaned pts.

**Conclusions:** An early rise in central venous pressure appears to predict weaning failure during a spontaneous breathing trial.

### A881 Disseminated intravascular coagulation during early phase of out-of-hospital cardiac arrest and resuscitation belongs to the fibrinolytic phenotype

#### Y. Sadamoto, K. Katabami, T. Wada, Y. Ono, K. Maekawa, M. Hayakawa, A. Sawamura, S. Gando

##### Hokkaido University Graduate School of Medicine, Emergency and Critical Care Medicine, Sapporo, Japan

###### **Correspondence:** Y. Sadamoto – Hokkaido University Graduate School of Medicine, Emergency and Critical Care Medicine, Sapporo, Japan

**Background:** Hypoxia and ischemia influence blood coagulation and fibrinolysis, if sufficiently severe like cardiac arrest and resuscitation, cause disseminated intravascular coagulation (DIC). We investigated characteristics of DIC diagnosed during the first 24 hours after cardiac arrest and return of spontaneous circulation.

**Methods:** Patients with established out-of-hospital cardiac arrest (OHCA) who underwent cardiopulmonary resuscitation with subsequent return of spontaneous circulation were retrospectively enrolled. Patients were divided two subgroups with DIC (208) and without DIC (180) by the Japanese Association for Acute Medicine (JAAM) DIC criteria. Platelet count, global markers of coagulation and fibrinolysis were measured 4 times after admission to emergency department (T1, 0–6; T2, 6–12; T3, 12–18; T4, 18–24 hr). A FDP/D-dimer ratio was used as a surrogate marker of fibrin(ogen)olysis.

**Results:** DIC patients showed significantly lower platelet counts, fibrinogen and antithrombin levels, and more prolonged prothrombin time throughout the study period. FDP and D-dimer levels as well as FDP/D-dimer ratio of DIC patients were extremely higher than those without DIC. Mean values of FDP/D-dimer ratio of DIC patients exceed 2.0, which suggests fibrin(ogen)olysis. Higher sequential organ failure assessment score (SOFA) (9 vs. 6, p < 0.001) associated with increased prevalence of multiple organ dysfunction (23.6 % vs. 3.9 %, p < 0.001) in DIC patients were observed, which leads to higher hospital mortality of DIC patients (54.8 % vs. 23.9 %, p < 0.001).

**Conclusion:** DIC immediately after cardiac arrest and resuscitation shows increased fibrin(ogen)olysis, which suggest that the DIC belongs to fibrinolytic phenotype. This type of DIC may affects OHCA patient's outcome.

### A882 Experience in circulatory and respiratory support with extracorporeal membrane oxigenation (ECMO) in complicated postoperative course after pulmonary endarterectomy

#### H. Marin-Mateos, J.L. Perez-Vela, R. Garcia-Gigorro, M.A. Corres Peiretti, M.J. Lopez-Gude, S. Chacon-Alves, E. Renes-Carreño, J.C. Montejo-González

##### Hospital 12 de Octubre, Medicina Intensiva, Madrid, Spain

###### **Correspondence:** H. Marin-Mateos – Hospital 12 de Octubre, Medicina Intensiva, Madrid, Spain

**Introduction:** Pulmonary endarterectomy is an effective solution for chronic thromboembolic pulmonary hypertension. However outcome at the ICU is not always easy due to important changes in respiratory and cardiovascular systems after surgery.

**Objectives:** To describe our experience in circulatory and respiratory assistance with ECMO in complicated postoperative course after bilateral thromboendarterectomy in patients with pulmonary hipertension cause by Thromboembolic Chronic Disease.

**Methods:** An observational research about a prospective data basis is made. All patients with this type of assistance requirements, admitted in the ICU for five years, from January 2010 to December 2015 are included. Variables as demographic and hemodynamics data, resource consumption in days of ICU stay, hours of mechanical ventilation, hours of ECMO therapy, complications, outcomes and mortality are analysed.

**Results:** In total, eight patients requiered ECMO support in the postoperative course after endarterctomy. The median age was 54 years (ICR 39–67), 62,5 % women. Peripheral cannulation in 100 % cases. The type of asisstance was veno-venous in 3 cases and veno-arterial in 5 cases. Indication for assistance was respiratory falilure alone in 37 % of patients, and combination of respiratory and cardiogenic shock (mainly caused by right ventricle failure) in 63 %. Underlying patology for respiratory and circulatory failure was reperfusión pulmonary edema alone 25 %, combination of reperfusión edema and right ventricle failure 50 %, anda diffuse alveolar haemorrhage in 25 % cases. A median of 204 hours (ICR 151–334) of therapy with ECMO is observed. Three patients presented bleeding complication (two minor pericannula bleeding and one major complication with intracraneal bleeding). Five patients developed acute renal failure, four of them with renal replacement therapy requirement. 62 % patients required norepinefrine and dobutamine infusion before cannulation. Hours of mechanical ventilation median 624 (ICR 451–1110), days of ICU stay median 26.5 (ICR 12–51) and in-hospital stay median 31 days (ICR 13–82). Mortality in ICU was 50 % of cases. Two-years survival was 100 % in patients where succesful weaning from ECMO therapy was performed.

**Conclusions:** Sometimes, pulmonary endarterectomy may present a complicated postoperative course with severe respiratory insufficiency and/or right ventricle failure (cardiogenic shock), that implies high morbidity and mortality, and high resource consumption. In these patients ECMO assistance is a useful support for ICU management and patients survival.

### A883 Lung ultrasound detects pulmonary complications earlier than chest x-ray in patients after cardiac surgery: a prospective single centre observational study

#### K.L. Parlevliet^1^, H.R.W. Touw^2^, M. Beerepoot^1^, C. Boer^2^, P.W.G. Elbers^1^, P.R. Tuinman^1^

##### ^1^VU University Medical Center, Intensive Care Medicine, Amsterdam, Netherlands; ^2^VU University Medical Center Amsterdam, Anaesthesiology, Amsterdam, Netherlands

###### **Correspondence:** K.L. Parlevliet – VU University Medical Center, Intensive Care Medicine, Amsterdam, Netherlands

**Introduction:** Postoperative pulmonary complications (PPCs) are common after cardiac surgery and especially clinically relevant PPCs (cr-PPCs) are associated with adverse outcomes. Early detection is important as it may lead to improved outcome (1). The accuracy of chest X-rays (CXRs) in the detection of PPCs is limited. Lung ultrasound (LUS) is emerging as an accurate diagnostic tool for pulmonary pathology within the intensive care unit (ICU) (2, 3). In literature little is known about the value of LUS for the detection of PPCs in patients after cardiac surgery.

**Objectives:** The primary aim of this study was to assess whether routine LUS can detect cr-PPCs earlier than routine CXR in patients after cardiac surgery.

**Methods:** This is a prospective observational single centre study performed in a tertiary ICU. 40 cardiac surgical patients were included. LUS was performed on the same days as routine CXR; at ICU admission (day 0), day 2 and day 3 post-operative. CXR and LUS findings on day 0, 2 and 3 were compared to cr-PPCs. Cr-PPCs were defined as PPCs requiring an intervention by the attending ICU-physician and considered the composite reference standard. Physicians were blinded for LUS findings. To analyse earlier detection of cr-PPCs the Wilcoxon Signed-Rank test was used. Detection of a cr-PPC was ranked per day and rankings between methods were compared per patient.

**Results:** Overall PPC (≥1) incidence was 37 (92.5 %) for CXR and 40 (100 %) for LUS. Fifteen cr-PPCs were identified in 14 patients (35 %). Patients with a cr-PPC had a prolonged ICU stay (82 ± 45 vs. 53 ± 24 hours; *p* = 0.004) and a lower PaO_2_/FiO_2_ ratio (199 ± 56 vs. 277 ± 97 mmHg; *p* = 0.010) compared to patients without cr-PPCs. CXR detected 5 (33.3 %) and LUS detected 11 (78.6 %) cr-PPCs. These cr-PPCs were detected earlier with LUS than CXR as presented in Table 19 (*p* = 0.024).

**Conclusions:** LUS detects the more PPCs and cr-PPCs than CXR and in an earlier stage. Further research needs to determine which specific LUS findings are associated with the development of clinically relevant pulmonary complications.

**References**

1. Canet J, Gallart L, Gomar C, Paluzie G, Vallès J, Castillo J, et al. Prediction of postoperative pulmonary complications in a population-based surgical cohort. Anesthesiology. 2010 Dec;113(6):1338–50.

2. Touw HRW, Tuinman PR, Gelissen HPMM, Lust E, Elbers PWG. Lung ultrasound: routine practice for the next generation of internists. Neth J Med. 2015;73(3):100–7.

3. Lichtenstein DA. Relevance of Lung Ultrasound in the Diagnosis of Acute Respiratory Failure*. CHEST J. 2008 Jul 1;134(1):117.

**Grant acknowledgement**

This study is not supported by any grant.

**Table 19 (abstract A883).** Cumulative number of detected cr-PPCs by CXR, LUS and the composite reference standard (cr-PPC) per day after cardiac surgery

	Day 0			Day 2			Day 3		
	CXR	LUS	cr-PPC	CXR	LUS	cr-PPC	CXR	LUS	cr-PPC
Atelectasis (n = 2)	1	2	2	1	2	2	1	2	2
Pulmonary edema (n = 9)	1	6	0	2	7	4	3	7	9
Other (n = 4)	1	2	1	1	2	4	1	2	4
Total (n = 15)	3	10	3	4	11	10	5	11	15

### A884 Predictors of severe hypotension in ICU patients sedated with propofol

#### S.A. Abdelmonem^1^, T.A. Helmy^1^, I. El Sayed^2^, S. Ghazal^1^

##### ^1^University of Alexandria, Critical Care, Alexandria, Egypt; ^2^Medical Research Institute, Biomedical Informatics & Medical Statistics, Alexandria, Egypt

###### **Correspondence:** S.A. Abdelmonem – University of Alexandria, Critical Care, Alexandria, Egypt

**Introduction:** Propofol is widely used in critical care sedation due to its pharmacological properties which allow serial neurological examination^(1)^.Hypo tension is a common side effect of propofol infusion, which affect patient outcome.

**Objectives:** Aimed to identify predictors of severe hypotension in patients sedated with propofol

**Methods:** This prospective study included 105 patientsthat were randomly selected by simple random sampling from population of patients admitted to the critical care medicine department in Alexandria Main University Hospital (49 normotensiveand 56 hypotensive).Bivariate analyses were performed to identify risk factors of severe hypotension during propofol infusion.Multivariate logistic regression analysis was performed to identify variables independently associated with hypotension after adjustment of other factors

**Results:** Multivariable logistic regression identified age, baseline MAP, Serum bicarbonate and serum creatinine as factors independently associated with and account for 86 % of the hypotension variance.The odds of hypotension increases by 1.17(95 % CI: 1.06 to 1.29) per one year increase in age. One unit increase in HCO3 results in decrease chance to develop hypotension by 30 % (OR:.705; 95 % CI:.523 to .951). One unit increase in MAP at beginning of treatment decrease the odds of hypotension by 7.6 % (OR:.924; 95 % CI:.869 to .982). Both Age and serum biacarbonate have fair diagnostic accuracy to differentiate cases with and without hypertension (AUC (SE): .746(.050), .785 (.047)) respectively. Baseline MAP < 98 mmHg has good diagnostic accuracy to differentiate cases with hypotension (AUC (SE): .837(.040)).

**Conclusions:** Age and serum bicarbonate can predict hypotension during propofol infusion. We should use propofol with caution in elderly and patients with baseline MAP < 98 mmHg.

**References**

1- Shah NK, Harris M, Govindugari K, Rangaswamy HB, Jeon H. Effect of propofol titration v/s bolus during induction of anesthesia on hemodynamics and bispectral index. Middle East J Anesthesiol. 2011;21(2):275–81. 2.

**Grant acknowledgement**

Thanks to all staff of the Department of Critical Care Medicine, University of Alexandria

### A885 Interlukins and post coronary artery bypass delirium in intensive care unit, an experimental clinical study

#### S.H. Akhlagh^1^, M. Masjedi^1,2^, K. Hozhabri^1^, E. Kamali^3^

##### ^1^Shiraz University of Medical Sciences, Anesthesia and Intensive Care Department, Shiraz, Islamic Republic of Iran; ^2^Anesthesiology and Critical Care Research Center, Shiraz University of Medical Sciences, Shiraz, Islamic Republic of Iran; ^3^Shiraz University of Medical Sciences, Shiraz, Islamic Republic of Iran

###### **Correspondence:** M. Masjedi – Shiraz University of Medical Sciences, Anesthesia and Intensive Care Department, Shiraz, Islamic Republic of Iran

**Introduction:** Delirium is a common Complication in elderly patients after Cardiac surgery. The pathophysiology of delirium remains poorly understood. Several conditions associated with delirium are characterized by activation of inflammatory Cascade.

**Objectives:** The purpose of this study was to find out the correlation between serum interleukins 6 and 10 with delirium in intensive care unit following coronary artery bypass surgery.

**Methods:** In an exploratory observational study we included 20 patients with delirium that has undergone coronary artery bypass surgery (CABG). Serum samples were taken, postoperatively, before, during and after development of delirium in cardiac intensive care unit . Delirium was diagnosed by using the Confusion- Assessment Method- ICU (CAM- ICU). IL_6_ and IL_10_ were determined by Enzyme Linked Immunosorbent Assay.

**Results:** Delirium was detected in 20 of 380 patients (5.2 %) which were admitted in cardiac ICU during a 17 month period. In delirious patients Plasma IL_6_ Level were higher in delirious stage than post delirium period (p = 0/002). Patients with the hyperactive Subtype of delirium had higher IL_6_ Levels in delirious stage than post delirium period (p = 0/009). There was no difference in Serum IL_10_ levels in delirious and post delirious period.

**Conclusions:** Interleukin 6 may contribute to the pathogenesis of post coronary artery bypass delirium. Its role is more prominent in the hyperactive behavior of delirium. No correlation was found between serum level of interleukin10 and delirium. Further studies are needed to elucidate the relationship of other cytokines with the pathogenesis of delirium.

**References**

1. De Rooij SE, Van Munster BC, Korevaar JC, Levi M. Cytokines and acute phase response in delirium. J Psychosom Res. 2007 May; 62 (5): 521–5.

2. Boogaard M, Kox M, Quinn K, Achterberg T, Hoeven J, Schoonhoven L, et al. Biomarkers associated with delirium in critically ill patients and their relation with long- term subjective cognitive dysfunction; indications for different pathways governing delirium in inflamed and noninflamed patients. Crit Care. 2011; 15 (6): R 297.

3. Miyazaki S, Yoshitani K, Miura N, Irie T, Inatomi Y, Ohnishi Y, et al. Risk factors of stroke and delirium after off- pump Coronary artery bypass surgery. Interact Cardiovasc Thorac Surg. 2011 Mar; 12 (3): 379–83.

**Grant acknowledgement**

This study was financially supported by the vice chancellery for research affairs of Shiraz university of medical sciences, Shiraz, Iran.

Many thanks to all nurses of cardiac ICU of Nemazee hospital , Shiraz , Iran.

### A886 Assessment of hemostasis in patients undergoing therapeutic plasma exchange by rotation thrombelastometry

#### I. Zýková, B. Paldusová, P. Sedlák, D. Morman

##### Regional Hospital Liberec, Department of Anesthesia and intensive Care, Liberec, Czech Republic

###### **Correspondence:** I. Zýková – Regional Hospital Liberec, Department of Anesthesia and intensive Care, Liberec, Czech Republic

**Introduction:** Therapeutic plasma exchange (TPE) is a therapeutic option in a variety of diseases. In most indications albumin is recommended as a replacement fluid. During TPE coagulation factors are removed and a depletion of coagulation factors results. We have found no recommendations for the assessment of hemostasis and management of a bleeding risk in patients undergoing TPE protocol. The increased bleeding risk is important in the management of these patients. The assesment of hemostasis by rotational thrombelastometry (ROTEM) is a fast bedside method, which can be used in assessment of these patients.The only published data about assessment of hemostasis by rotational thrombelastometry in patients after TPE are in patients using TPE before cadaveric donor kidney transplant (1). We found no data of ROTEM use in patients, where TPE protocols with series of TPE were used.

**Objectives:** To assess the usage of rotational thrombelastometry in patients undergoing series of TPE.

**Methods:** We have performed rotational thrombelastometry (ROTEM) in 4 patients with Guillain Barré syndrome undergoing TPE therapy. All patients had a series of TPE, the interval between TPE was 24–48 hours. Albumin was used as a replacement fluid. We performed standard coagulation tests and rotational thrombelastometry before and after each TPE.

**Results:** TPE reduced fibrinogen levels both in a standard laboratory test and according to FIBTEM (MCF). Clotting time (CT) and clot formation time (CFT) were prolonged both in INTEM and EXTEM. Standard coagulation tests were also prolonged (aPTT, INR). The trombelastometry results reflected a serious coagulopathy which corrected spontaneously between each TPE. We used thrombelastometry for timing of invasive procedures. We did not correct coagulopathy in patients without clinical bleeding. We corrected coagulopathy only in patients with bleeding or undergoing invasive procedures. Thrombelastometry was a fast and easy bedside test for the assessment of bleeding risk in these patients.

**Fig. 21 (abstract A886).**
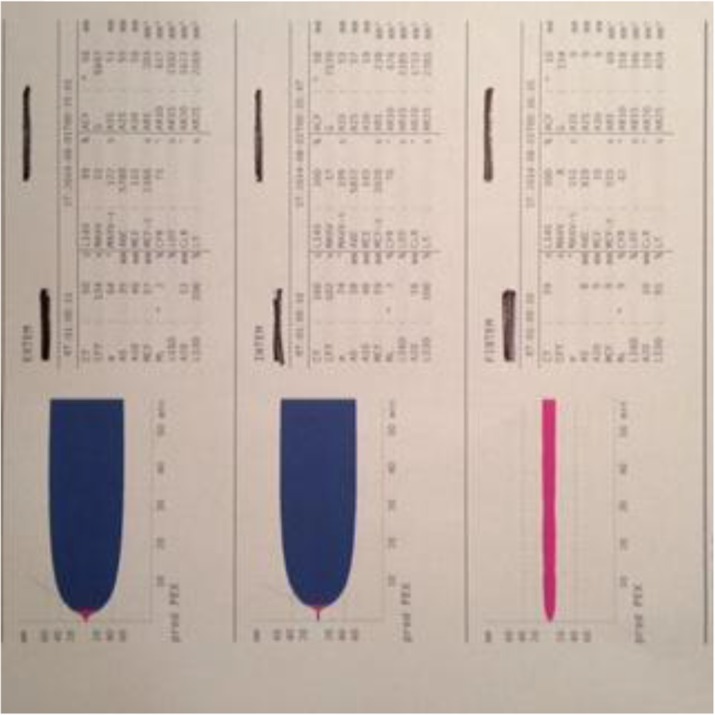
ROTEM before TPE

**Fig. 22 (abstract A886).**
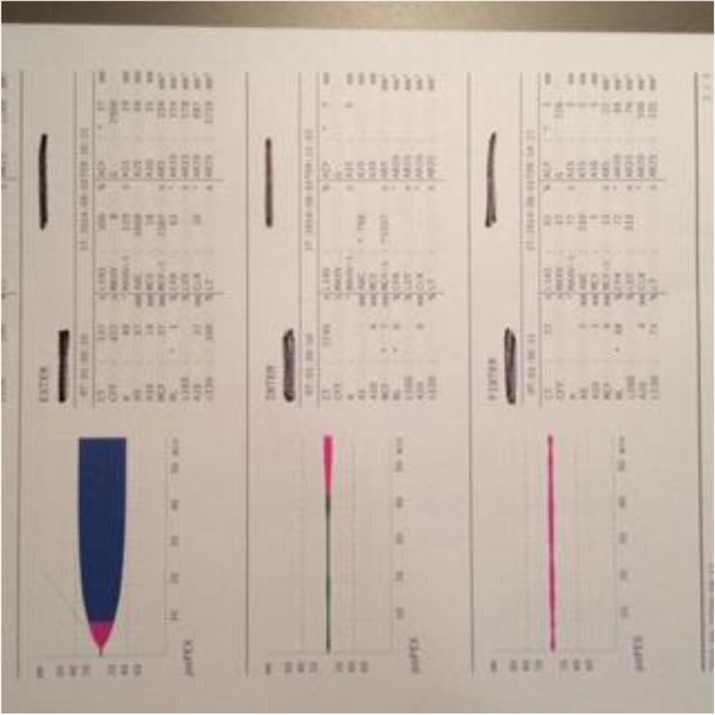
ROTEM after TPE

**Conclusions:** Hemostasis was severely infuenced by TPE, the resulting coagulopathy corrected spontaneously before next TPE. Assesment of hemostasis should always be done in patients on TPE using albumin as a replacement fluid. The bleeding risk should be monitored and measured. The timing of invasive procedures such as central vein cannulation and tracheostomy should be guided by coagulation studies. Rotational thrombelastometry can be used to asses hemostasis and bleeding risk. In patients with Guillain-Barre syndrome spinal tap is performed. Caution should be given to the timing of spinal tap and TPE.

**References**

1. Tholking G, Mesters R, Dittrich R et al. Assessment of Hemostasis after Plasma Exchange Using Rotational Thrombelastometry (ROTEM).PLoS ONE 10(6):e0130402.doi:10.1371

### A887 Heated carrier fluids in decreasing propofol injection pain: a randomized controlled trial

#### A.M. Youn

##### Chungnam National University Hospital, Anesthesiology and Pain Medicine, Daejon, Republic of Korea

**Introduction:** Propofol is a common intravenous drug used during anesthetic induction and sedation because of its rapid onset and short duration. Its downfall, however, is that patients experience injection pain so severe that they recall induction as the most painful part of the sedation process. Among numerous reports in efforts to decrease propofol injection pain, the most effective combination of drug and non-drug intervention evaluated through a quantitative systematic review revealed to be pretreatment with 0.5 mg/kg lidocaine in combination with a tourniquet for venous occlusion. The majority of these reports conclude that a single method is insufficient in eliminating propofol injection pain.

**Objectives:** We evaluated the effect of heated carrier fluids (40 °C) in decreasing propofol injection pain.

**Methods:** A randomized controlled clinical trial was conducted in 90 patients (ASA 1 or 2), ages 18 to 65. Patients were allocated into 3 groups (n = 30) each. Group W received 200 ml of heated carrier fluids for 20 minutes prior to 2 mg/kg propofol injection; group L received 200 ml of heated carrier fluids for 20 minutes prior to lidocaine pretreatment and 2 mg/kg propofol injection: and group C (control group) received 200 ml of room temperature fluids prior to 2 mg/kg propofol injection. Propofol injection pain was evaluated using the verbal pain score (VPS).

**Results:** Group W and L showed significant reduction in the incidence and severity of injection pain compared to group C (P < 0.05). VPS was significantly lower in group W (p = 0.018) and L (p = 0.036) compared to group C. There was no statistical difference between group W and group L (p = 0.3). There was statistically significant difference in mean blood pressures measured after 2 mg/kg propofol injection among groups.

**Conclusions:** Both heated carrier fluids and combination of lidocaine pretreatment effectively reduced propofol injection pain.

**References**

1. Jalota L, Kalira V, George E, Shi YY, Hornuss C, Radke O, et al. Prevention of pain on injection of propofol: systematic review and meta-analysis. BMJ 2011; 342: d1110.

2. Picard P, Tramer MR. Prevention of pain on injection with propofol: a quantitative systematic review. Anesth Analg 2000; 90: 963–9.

### A888 In-hospital adverse events among surgical patients in japan: the JET study

#### Y. Ohta^1^, M. Sakuma^2^, D. Bates^3^, T. Morimoto^2^

##### ^1^Hyogo College of Medicine, General Internal Medicine, Nishinomiya, Japan; ^2^Hyogo College of Medicine, Clinical Epidemiology, Nishinomiya, Japan; ^3^Brigham and Women's Hospital, General Internal Medicine, Boston, United States

###### **Correspondence:** Y. Ohta – Hyogo College of Medicine, General Internal Medicine, Nishinomiya, Japan

**Introduction:** The epidemiology and the nature of adverse events (AEs) were mainly reported in Western countries and were limited in the outside Western countries. However, the local epidemiological data is important to extrapolate the effects of established preventive strategy of AEs in Western countries into other countries.

**Objectives:** We conducted a prospective cohort study to clarify the epidemiology and the nature of AEs in surgical inpatients in Japan.

**Methods:** The Japan adverse EvenT (JET) study was a prospective cohort study which had evaluated AEs and medical errors (MEs) at 2 tertiary care hospitals. The 38 medical and surgical wards were stratified according to hospital and whether they were medical or surgical wards, and study wards were randomly selected. Intensive care units (ICUs) were all included. We included all adult patients aged > = 15 years old who were admitted to any of the selected 23 study wards (10 medical, 11 surgical, and 2 ICUs) over a 2-month period. The primary outcome of this study was the epidemiology and the nature of AEs and MEs in the patients who had operation during the study period. Trained nurses placed at each participating hospital reviewed all charts daily on weekends, along with laboratories, incident reports, and prescription queries to collect any potential event. They also collected the characteristics of the patients in the cohort. Independent physician reviewers evaluated all potential events and classified to whether they were AEs or MEs, as well as to their classification, severity and preventability.

**Results:** The JET study reported that 1,130 patients enrolled with 19,180 patient-days. We included 392 patients with 7046 patient-days. Among 392 patients, 227 patients (58 %) were male and the median of age at admission was 68 years old {Interquartile range (IQR): 55, 78}. Nine patients (2 %) died during hospital stay and the median hospital stay was 9 days (IQR: 5, 20). We found 489 AEs in 202 patients, for the incidence of 69.4 per 1000 patient-days and 124.7 per 100 admissions, and 208 MEs in 108 patients, for the incidence of 29.5 per 1000 patient-days and 53 per 100 admissions.

We classified AEs into following type with duplicate count: surgical operations consisted of 35 % events, followed by drug 31 %, procedure 15 %, nursing 10 %, and others 8 %. In terms of severity of AEs, 1 % were fatal; 5 % were life-threatening; 31 % were serious; and 63 % were significant events. Among the 489 AEs, 119 (24 %) were associated with error and preventable.

**Conclusions:** We found a significant high incidence of AEs and MEs among surgical inpatient in Japan. We also showed operation related AEs were the most frequent AEs.

## Oral Sessions. PEEP AND ALVEOLAR RECRUITMENT

### A889 Positive end-expiratory pressure selection in patients with acute respiratory distress syndrome: alveolar recruitment versus electrical impedance tomography based lung collapsibility

#### P.-L. Su^1^, W.-Y. Chang^1^, W.-C. Lin^1^, C.-W. Chen^1,2^

##### ^1^National Cheng Kung University Hospital, Department of Internal Medicine, Tainan, Taiwan, Province of China; ^2^National Cheng Kung University, Medical Device Innovation Center, Tainan, Taiwan, Province of China

###### **Correspondence:** P.-L. Su – National Cheng Kung University Hospital, Department of Internal Medicine, Tainan, Taiwan, Province of China

**Introduction:** Appropriate PEEP selection is crucial in the ventilator management of ARDS patients. Yet information of alveolar recruitment or degree of lung collapse in the selection of PEEP is unknown.

**Objectives:** Selection of PEEP based on best dorsal(dependent) compliance through electrical impedance monitoring during decremental PEEP titration^1^ and its relationship to alveolar recruitment and lung collapsibility.

**Methods:** Twenty-three patients with ARDS received decremental PEEP titration at 5 levels of PEEP (25, 20, 15, 11, 7 cmH_2_O) following a short cycle of recruitment maneuver. At PEEP level of 15, 11, 7 cmH_2_O, end-expiratory lung volume(EELV) was measured with nitrogen washout/washin technique and estimation of PEEP-induced alveolar recruitment was determined after subtraction of the minimum predicted increase in lung volume (△PEEP X Compliance _rs_ at PEEP = 7 cmH_2_O). Estimation of lung collapsibility was obtained via regional lung compliance changes obtained from EIT at five PEEP levels according to the method of Costa et al^2^. The PEEP level with the best dorsal compliance is defined as the selected PEEP.

**Results:** Data are presented as median [interquartile range] or mean ± SD. PEEP induced alveolar recruitment was 183.8 ml [22.1 - 340.3](PEEP level of 15 cmH_2_O to 7 cmH_2_O) and alveolar recruitment equal to or greater than the median (183.8 ml) as high recruiter (HR), otherwise it is low recruiter(LR). Degree of lung collapsibility between HR and LR at the PEEP level of 7 cmH_2_O was not significantly differed (25.2 ± 11.4 % in HR vs 21.4 ± 12.7 % in LR) and the final PEEP selected was not differed too. However, for patients with final selected PEEP level of 20 cmH_2_O/15 cmH2O/≤ 11cmH2O, the corresponding lung collapsibility at PEEP level of 7 cmH_2_O was 41.2 ± 2.7 %/23.4 ± 8.6 %/11.6 ± 4.5 % (P = 0.0004 between 3 groups). At selected PEEP level, lung collapsibility was 1.5 ± 1.8 % for HR and 1.3 ± 1.9 % for LR.

**Conclusion:** The information of alveolar recruitment did not aid in our final determination of PEEP level in ARDS patients while information about lung collapsibility by EIT may be of help. EIT based lung collapsibility may be considered for the determination of PEEP level in ARDS patients.

**References**

1. Wolf GK, Gomez-Laberge C, Rettig JS, Vargas SO, Smallwood CD, Prabhu SP, Vitali SH, Zurakowski D, Arnold JH, (2013) Mechanical ventilation guided by electrical impedance tomography in experimental acute lung injury. Critical care medicine 41: 1296–1304

2. Costa EL, Borges JB, Melo A, Suarez-Sipmann F, Toufen C, Jr., Bohm SH, Amato MB, (2009) Bedside estimation of recruitable alveolar collapse and hyperdistension by electrical impedance tomography. Intensive care medicine 35: 1132–1137

**Grant acknowledgement**

National Science Concil

National Cheng Kung University Hospital

### A890 ULTRAPEEP: lung ultrasound for the assessment of lung recruitment during esophageal pressure-guided PEEP in ARDS

#### F. Facchin^1^, F. Zarantonello^1^, G. Panciera^1^, A. De Cassai^1^, A. Venrdramin^1^, A. Ballin^1^, T. Tonetti^1^, P. Persona^2^, C. Ori^1^, L. Del Sorbo^3^, S. Rossi^2^

##### ^1^University of Padova, Department of Medicine (DIMED), Padova, Italy; ^2^Azienda Ospedaliera di Padova, Emergency Department, Padova, Italy; ^3^University of Toronto, Interdepartmental Division of Critical Care Medicine, Toronto, Canada

###### **Correspondence:** F. Facchin – University of Padova, Department of Medicine (DIMED), Padova, Italy

**Introduction:** Whereas the importance of low tidal volume to avoid ventilator-induced lung injury (VILI) in patients with ARDS is well known, several uncertainties still exist regarding how to set positive end-expiratory pressure (PEEP). Many approaches have been considered, but no one showed a clear effectiveness in terms of outcome. Recently a ventilator strategy using esophageal pressure to estimate the transpulmonary pressure has been proposed by Talmor and colleagues^1.^ Although they found an improvement in arterial oxygenation, it was not explored whether the increase in oxygenation was due to lung recruitment.

**Objectives:** The aim of this study was to assess whether the PEEP set to maintain a positive end-expiratory transpulmonary pressure (P_L_) is associated with an increase in lung recruitment estimated by lung ultrasound score (LUS)^2^.

**Methods:** 12 patients with moderate and severe ARDS were enrolled. For the first 2 hours, PEEP was set according to the Acute Respiratory Distress Syndrome Network standard-of-care recommendations (phase A). It was then adjusted according to measurements of esophageal pressure for the following 2 hours (phase B) to maintain a positive P_L_ at the end of expiration. The primary end point was the improvement in lung recruitment assessed with lung ultrasound.

**Results:** Lower end-expiratory P_L_ was found during phase A compared to phase B [median −3.5 cmH_2_O [−4.5 to −2] vs 3 cmH_2_O [1 to 4.25], p = 0.002]. During phase B PEEP has been increased from a median of 10 cmH_2_O to 17.5 cmH_2_O (p = 0.002). Arterial oxygenation improved in phase B compared to phase A, (median PaO_2_/FiO_2:_ phase A 146.5 mmHg [117 to 166] vs phase B 164.5 [155 to 180], p = 0.037). The median LUS was 19.5 during phase A (IQR 11.75 to 28) and decreased to 17.5 (IQR 10.25 to 19.75) during phase B (p = 0.028). A decrease in LUS ≥4 (indicating lung recruitment) was found in 8 of 12 patients.

**Conclusions:** In patients with moderate-severe ARDS, PEEP-induced LUS reduction and increase in oxygenation appears to indicate that setting PEEP according to the esophageal pressure-guided method results in a greater alveolar recruitment than setting PEEP according to ARDSNet strategy. Further investigations are needed to assess the potential impact of PEEP-induced alveolar overdistension.

**References**

1. Talmor D, et al. Mechanical ventilation guided by esophageal pressure in acute lung injury. N Engl J Med. 2008 Nov 13;359(20):2095–104.

2. Bouhemad B, et al. Ultrasound assessment of antibiotic-induced pulmonary reaeration in ventilator-associated pneumonia. Crit Care Med. 2010 Jan;38(1):84–92.

### A891 Inspiratory recruitment in acute respiratory distress syndrome

#### G. Vergani^1^, M. Cressoni^1^, D. Chiumello^2,3^, C. Chiurazzi^4^, M. Brioni^1^, I. Algieri^1^, T. Tonetti^5^, M. Guanziroli^1^, A. Colombo^2^, I. Tomic^6^, A. Colombo^1^, F. Crimella^1^, E. Carlesso^1^, V. Gasparovic^6^, L. Gattinoni^5^

##### ^1^Università degli Studi di Milano, Dipartimento di Fisiopatologia Medico Chirurgica e dei Trapianti, Milano, Italy; ^2^Policlinico Di Milano, Dipartimento di Anestesia, Rianimazione, Urgenza ed Emergenza, Milano, Italy; ^3^Plug Working Group, Milan, Italy; ^4^Università degli Studi di Milano, Dipartimento di Fisiopatologia Medico-Chirurgica e dei Trapianti, Milano, Italy; ^5^Georg-August-University Goettingen, Anesthesiology and Intensive Care Medicine, Goettingen, Germany; ^6^Univesity of Zagreb, Department of Intensive Care Medicine, Rebro, Croatia

###### **Correspondence:** C. Chiurazzi – Università degli Studi di Milano, Dipartimento di Fisiopatologia Medico-Chirurgica e dei Trapianti, Milano, Italy

**Introduction:** In ARDS lung protective strategy implies a fully open lung. Recruitment maneuvers are used in ARDS (Acute Respiratory Distress Syndrome) patients to reinflate the lung. Two studies described the distribution of airway opening pressures in ARDS patients. [1][2] No data are available on the relationship between opening pressures and disease severity.

**Objectives:** To describe lung recruitment as a function of the transpulmonary pressure in mild, moderate and severe ARDS.

**Methods:** ARDS patients underwent a low-dose end-expiratory CT scan at PEEP 5 cmH_2_O and three end-inspiratory CT scans at the plateau pressures reached starting from PEEP 5 cmH_2_O, 30 cmH_2_O and 45 cmH_2_O. In each of the CT slices, lung profiles were manually delineated, excluding hilar structures. Thereafter, quantitative analysis of CT scan images was performed and the gas and tissue fractions were computed. We defined the recruitability as the difference of not inflated tissue between 45 and 5 cmH_2_O, that we arbitrarily assumed to be the “full recruitment”. [3] The grams of recruited tissue were computed across the pressure intervals at which the CT scan were performed, as the differences of not aerated tissue. Airway and esophageal pressures were continuously measured and transpulmonary pressure was computed as: Driving airway pressure (cmH_2_O) - (esophageal plateau pressure (cmH_2_O) - esophageal end-expiratory pressure at PEEP (cmH_2_O)[1].

**Fig. 23 (abstract A891).**
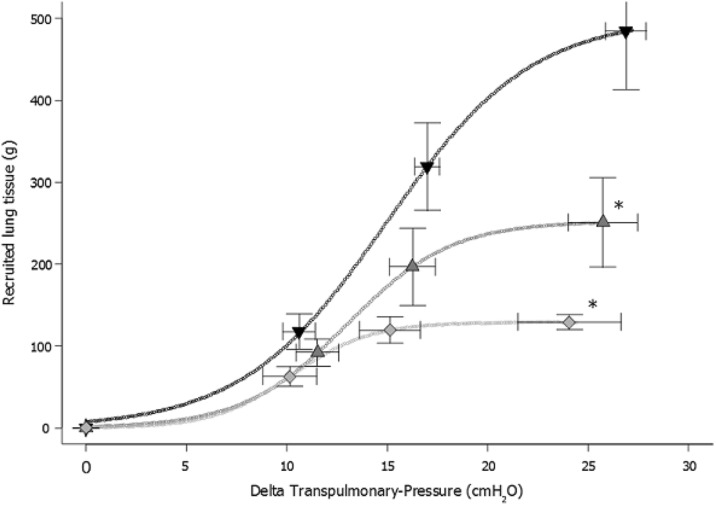
Recruitment as function of applied TP pressures

**Results:** Thirty-three patients were studied, 5 with mild, 10 with moderate and 18 with severe ARDS, according to the Berlin definition.[2] As reported in the Table 20 and Fig. 23, the amount of tissue which can be opened between 30 and 45 cmH_2_O was 8 %, 17 % and 37 % respectively in mild, moderate and severe ARDS). Mild ARDS patients nearly completed recruitment at approximately 15 cmH_2_O transpulmonary pressure while in moderate and severe ARDS recruitment continues up to 26 cmH_2_O transpulmonary pressure.

**Conclusions:** At the clinically recommended plateau pressure of 30 cmH_2_O, in severe ARDS, up to 1/3 of the lung tissue recruitable at 45 cmH_2_O, stays always closed. Beyond contributing to the gas exchange impairment (depending on the perfusion), these “always” collapsed regions may also act as stress risers at their interface with aerated regions, though they are theoretically protected from the mechanical ventilation.

**References**

[1] Crotti S, *Am J Respir Crit Care Med* 2001.

[2] Borges JB, *Am J Respir Crit Care Med* 2006.

[3] Gattinoni L, *N Engl J Med* 2006.

[4] Chiumello D,*Am J Respir Crit Care Med* 2008.

[5] ARDS Definition Task Force, *JAMA J Am Med Assoc* 2012.

**Table 20 (abstract A891).** Recruited tissue

	Experimental step		
	6 ml/kg IBW from PEEP 5 cmH2O	30 cmH2O set from PEEP 5 cmH2O	45 cmH2O set from PEEP 5 cmH2O
Mild ARDS			
Recruited tissue, g (% of recruitability)	63 ± 26 (51 ± 23 %)	56 ± 50 (41 ± 33 %)	10 ± 29 (8 ± 21 %)
Moderate ARDS			
Recruited tissue, g (% of recruitability)	92 ± 53 (42 ± 20)	105 ± 129* (41 ± 22 %)	54 ± 86* (17 ± 27 %)
Severe ARDS			
Recruited tissue, g (% of recruitability)	123 ± 94 (26 ± 17 %)	209 ± 180* (36 ± 22 %)	173 ± 112*$ (37 ± 18 %)

### A892 Associations between ventilator settings during extracorporeal membrane oxygenation and outcome in patients with acute respiratory distress syndrome: a pooled individual patient data analysis

#### A. Serpa Neto^1,2^, M. Schmidt^3,4^, T. Pham^5,6,7^, A. Combes^4,8^, M. Gama de Abreu^9^, P. Pelosi^10^, M.J. Schultz^2^, for the ReVA Research Network and the PROVE Network Investigators

##### ^1^Hospital Israelita Albert Einstein, Critical Care Medicine, São Paulo, Brazil; ^2^Academic Medical Center, University of Amsterdam, Intensive Care, Amsterdam, Netherlands; ^3^Australian and New Zealand Intensive Care Research Centre; School of Public Health, Monash University, Department of Epidemiology and Preventive Medicine, Melbourne, Australia; ^4^Institute of Cardiometabolism and Nutrition (iCAN); Hôpital de la Pitié-Salpêtrière, Assistance Publique-Hôpitaux de Paris, Medical-Surgical Intensive Care Unit, Paris, France; ^5^Groupe Hospitalier Henri Mondor; Assistance Publique-Hôpitaux de Paris, Service de Réanimation Médicale, Créteil, France; ^6^Inserm, Sorbonne Paris Cité, ECSTRA Team; Université Paris Diderot, UMR 1153, Paris, France; ^7^Inserm; Université Paris Est Créteil, UMR 915, Créteil, France; ^8^Université Pierre et Marie Curie-Paris VI, Service de Réanimation Médicale, Paris, France; ^9^University Hospital Carl Gustav Carus, Technische Universität Dresden, 21Pulmonary Engineering Group, Department of Anesthesiology and Intensive Care Medicine, Dresden, Germany; ^10^IRCCS San Martino IST, University of Genoa, Surgical Sciences and Integrated Diagnostics, Genoa, Italy

###### **Correspondence:** A. Serpa Neto – Hospital Israelita Albert Einstein, Critical Care Medicine, São Paulo, Brazil

**Introduction:** Extracorporeal membrane oxygenation (ECMO) is a rescue therapy for patients with acute respiratory distress syndrome (ARDS) by providing additional oxygenation, and removing carbon dioxide thus permitting less injurious mechanical ventilation settings that have been shown to protect the lungs from additional injury.

**Objectives:** To evaluate associations between distinct ventilator settings during ECMO, and outcome of ARDS patients.

**Methods:** Individual patient data analysis of observational studies in adult ARDS patients receiving ECMO for refractory hypoxemia. Multilevel multivariable logistic regression models and Cox-proportional hazards models were used to determine which settings and parameters had an independent association with the primary endpoint all-cause mortality.

**Fig. 24 (abstract A892).**
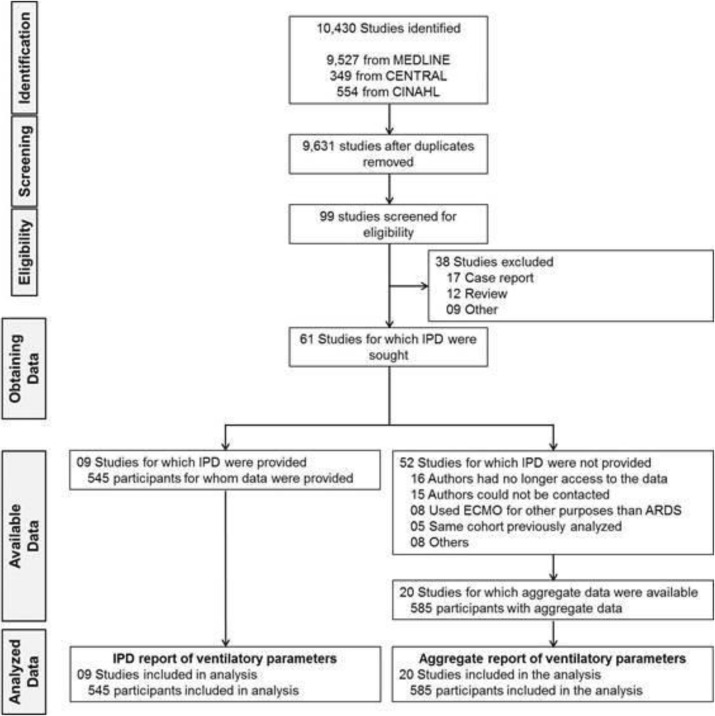
PRISMA-IPD flow diagram

**Fig. 25 (abstract A892).**
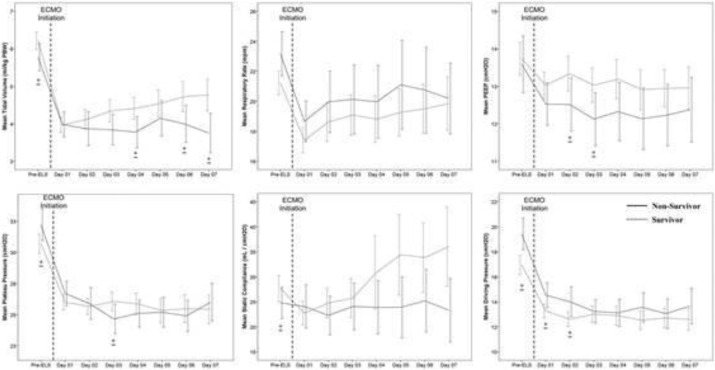
Ventilatory parameters during ECMO

**Fig. 26 (abstract A892).**
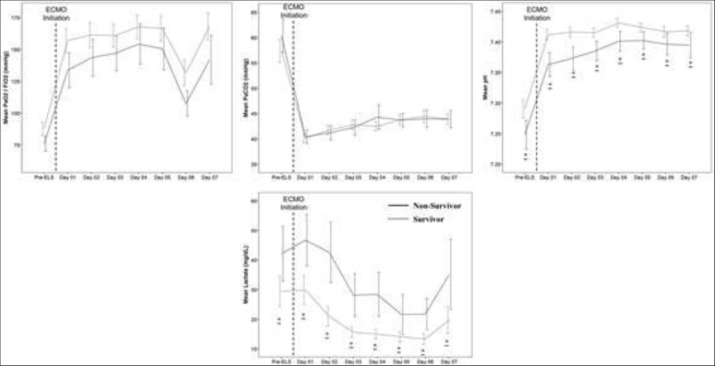
Laboratory parameters during ECMO

**Results:** Nine studies with 545 patients were selected (Figure 24). Initiation of ECMO was accompanied by significant decreases in tidal volume, positive end-expiratory pressure (PEEP), plateau pressure (Pplat), and driving pressure (ΔP = Pplat - PEEP), respiratory rate and minute volume (Figure 25), and resulted in higher PaO_2_ to FiO_2_ ratios, higher arterial pH and lower PaCO_2_ (Figure 26). Higher age, lower body mass index, and higher lactate were associated with all-cause mortality after multivariable adjustment. ΔP, both before and during the first three days of ECMO, demonstrated an independent association with all-cause mortality (adjusted OR, 1.17 [95 % CI 1.06-1.29] for ΔP before ECMO and adjusted OR, 1.13 [1.02-1.27] for ΔP during the first three days of ECMO). Ventilator FiO_2_ during the first three days of ECMO was independently associated with all-cause mortality (adjusted OR, 1.04 [95 % CI 1.01-1.08]).

**Conclusions:** In this series of ARDS patients receiving ECMO for refractory hypoxia, ΔP and FiO_2_ were the only ventilatory variables that had an independent association with outcome. These findings indicate the potential for improvement in the management of patients with ARDS undergoing ECMO.

**References**

Schmidt M, et al. Crit Care Med 2015;43:654–64.

Marhong JD, et al. Intensive Care Med 2015;41:994–1003.

### A893 Deflation injury following release of sustained PEEP

#### B.H. Katira^1,2,3^, D. Engelberts^1^, R.E. Giesinger^4^, C. Ackerley^5^, T. Yoshida^1,2,3^, D. Zabini^6^, G. Otulakowski^1^, M. Post^1^, W.M. Kuebler^6,7^, P.J. McNamara^4^, B.P. Kavanagh^1,2,3^

##### ^1^Hospital for Sick Children, Physiology and Experimental Medicine, Toronto, Canada; ^2^Hospital for Sick Children, University of Toronto, Critical Care Medicine and Anesthesiology, Toronto, Canada; ^3^University of Toronto, Interdepartmental Division of Critical Care Medicine, Toronto, Canada; ^4^Hospital for Sick Children, University of Toronto, Neonatal Medicine, Toronto, Canada; ^5^Hospital for Sick Children, Division of Electron Microscopy, Toronto, Canada; ^6^Keenan Research Centre, Li Ka Shing Knowledge Institute, St. Michael's Hospital, Toronto, Canada; ^7^University of Toronto, Surgery and Physiology, Toronto, Canada

###### **Correspondence:** B.H. Katira – Hospital for Sick Children, Physiology and Experimental Medicine, Toronto, Canada

**Introduction:** ARDS continues to be a significant problem with high mortality. Sustained inflation of lungs is routinely used to recruit the alveoli in ARDS but multiple RCTs of sustained inflation have been negative despite the physiological benefits. This, we think is because of a hidden form of injury offsetting the positive effects of lung inflation - injury induced by lung deflation.

**Aim:** To determine if sudden deflation of normal lungs after sustained inflation causes lung injury, its nature, timing, ultrastructural impact, and the possible hemodynamic mechanisms responsible for this injury.

**Methods:** Healthy Sprague Dawley rats were anaesthetised, ventilated (tidal volume 6 mL/kg), and randomized to Intervention or Control. Intervention was incremental increases in PEEP (3 to 11 cmH_2_O (over 70 min), abrupt deflation to ZEEP and ventilation for 30 min; Control was ventilation for 100 min (PEEP 3cmH_2_O).

Lungs were analysed for wet-to-dry ratio, BAL protein, static compliance, SpO_2_ and histology. To detect the timing of injury, rats received Evans Blue dye (EBD-30 mg/kg IV) at the initiation and were euthanized immediately before lung deflation or at 2, 5, 10 or 20 min afterwards (4/group). Terminal BAL analysed for EBD absorbance. Ultrastructural impact was studied by electron microscopy on lungs sampled from rats euthanized before deflation, and at 1 and 5 min after deflation. Hemodynamic data was obtained by ECHO performed at baseline (PEEP 3 cmH_2_O), immediately before and after deflation, and at 30mins after deflation. RV pressure was measured with a Millar catheter.

**Results:** Wet-to-dry ratio (6.1 ± 0.6 vs 4.6 ± 0.4; P = 0.00) and BAL protein (3.9 ± 4.0 vs 1.5 ± 0.7; P = 0.18) was higher; and static compliance (0.48 ± 0.97 vs 0.82 ± 0.2; P = 0.00) and SpO_2_ (67 ± 23.5 vs 91 ± 4.4; P = 0.04) were lower in Intervention vs Control. Histology revealed collapse, hemorrhage and neutrophil accumulation in the intervention group. BAL Evans Blue demonstrated that microvascular leak was absent before deflation and was maximal by 10 min of deflation.

Ultrastructural analysis showed that sustained inflation caused minimal swelling of epithelium and endothelium before deflation; deflation resulted in major cellular and interstitial edema, and endothelial injury. Hemodynamic data showed that RV and LV were under-filled during inflation. Upon deflation, RV output, pulmonary vascular resistance, RV systolic transmural and diastolic pressures increased precipitously. RV/LV ratio increased progressively.

**Conclusion:** Sudden deflation after sustained inflation with PEEP causes protein leak, inflammation, hypoxemia, reduced compliance, endothelial injury and RV failure. The mechanism appears to be endothelial injury resulting in microvascular leakage, pulmonary hypertension and RV failure.

**Significance:** Deflation injury may be an important entity to prevent when using sustained inflation manoeuvres and may explain -in part- why several important RCTs in ARDS have been negative.

**Fig. 27 (abstract A893).**
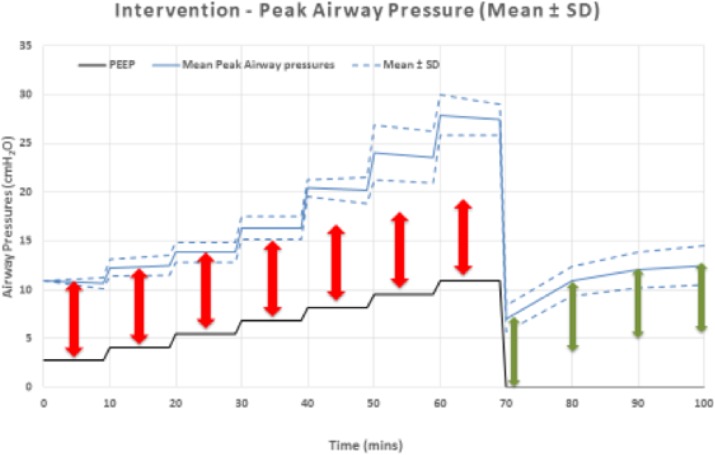
Airway Pressures in Intervention Group

**Fig. 28 (abstract A893).**
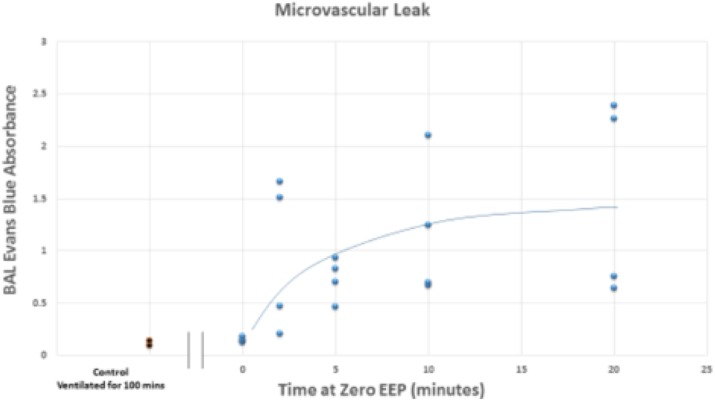
Microvascular leak

**Fig. 29 (abstract A893).**
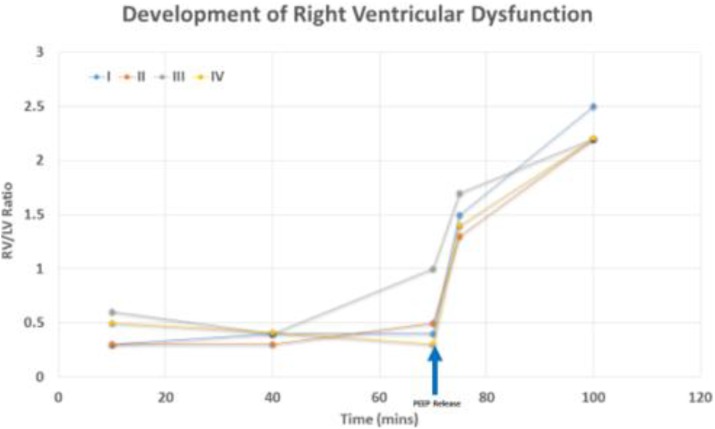
Development of right ventricular dysfunction

## SEPSIS THERAPEUTICS

### A894 Low dose steroids reduce short term mortality in septic shock patients: results of an individual patient data meta-analysis

#### R. Pirracchio^1,2^, M. Resche Rigon^3^, M. Carone^4^, S. Chevret^3^, D. Annane^5^

##### ^1^Hopital Europeen Georges Pompidou, Anesthesiology and Intensive Care Medicine, Paris, France; ^2^University of California Berkeley, Berkeley, United States; ^3^Université Paris Diderot, Paris, France; ^4^University of Washington, Seattle, United States; ^5^Université de Versailles Saint-Quentin-en-Yvelines, Garches, France

###### **Correspondence:** R. Pirracchio – Hopital Europeen Georges Pompidou, Anesthesiology and Intensive Care Medicine, Paris, France

**Introduction:** Previous research has suggested that the use of low dose steroids may be beneficial during septic shock. However subsequent inconsistent results explain the lack of consensus amongst doctors around the world about whether treatment with low dose steroids does improve the overall recovery and survival in patients with septic shock. We hypothetize that the lack of consistent evidence on the effect of low-dose steroids on short term mortality may be related to underpower. treated for septic shock.

**Objectives:** The primary objective of the present study was to estimate the effect of three different therapeutic regimens (hydrocortisone alone, hydrocortisone plus fludrocortisone, neither hydrocortisone nor fludrocortisone) on 28-day mortality in patients treated for septic shock using an individual patient data meta-analysis.

**Methods:** Individual patient data meta-analysis including the 3 major recent randomized controlled trials comparing early low-dose short course hydrocortisone and fludrocortisone to placebo (GER-inf (1)), hydrocortisone alone to placebo (CORTICUS (2)) or hydrocortisone to hydrocortisone and fludrocortisone (COIITSS (3)) in septic shock patients. The primary outcome measure was all cause 28-day mortality. Secondary outcomes measures were 90-day mortality, resolution of organ dysfunction (as measured by the time to reach a Sequential Organ Failure Assessement score < 8), time to vasopressor and mechanical ventilation discontinuation, intensive care unit and hospital lengths of stay as well as the rate of superinfection. Treatment effect on the primary outcome was quantified using relative risk and estimated using targeted maximum likelihood estimation.

**Fig. 30 (abstract A894).**
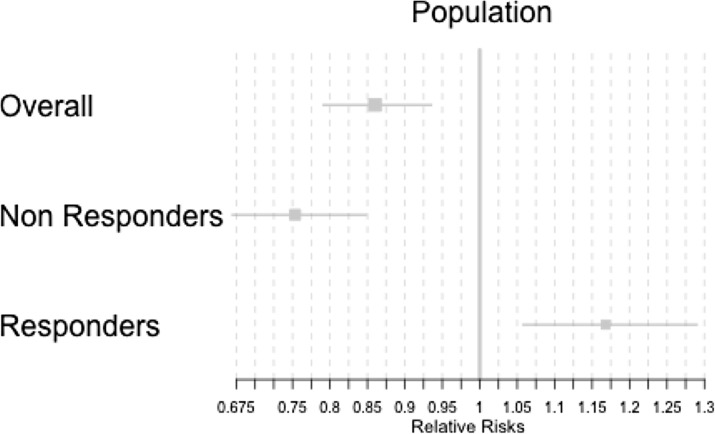
Effect of hydro + fludro vs placebo or hydro alone

**Results:** A total of 1,307 patients were enrolled in the 3 trials. When compared to the placebo, hydrocortisone + fludrocortisone significantly reduced 28-day mortality (RR = 0.73, 95%CI = 0.67-0.79, p < 0.001). Hydrocortisone + fludrocortisone was also superior when compared to the placebo and hydrocortisone pooled together (RR = 0.86, 95%CI = 0.79- 0.94, p = 0.002). Hydrocortisone + fludrocortisone significantly decreased 28-day mortality (RR = 0.75, 95%CI = 0.67-0.85, p < 0.001) in the nonresponders, while it was associated with an increase in 28-day mortality in the responders (RR = 1.17, 95%CI = 1.06-1.29, p = 0.002) (Figure 30). Hydrocortisone + fludrocortisone was also superior when considering secondary outcomes such as vasopressor discontinuation or lengths of stay.

**Conclusions:** In this individual patient data meta-analysis including the 3 major randomized controlled trials on the subject, we found that an early short course of low-dose hydrocortisone and fludrocortisone decreases 28-day mortality and improves recovery from organ failure in septic shock patients non responding to a corticotropin stimulation test.

**References**

(1) **JAMA** 2002 21;288(7):862–71;

(2) NEJM 2008 10;358(2):111–24;

(3) **JAMA** 2010 27;303(4):341–8

### A895 The clinical outcome of simvastatin plus standard therapy versus standard therapy alone in critically ill septic patients: randomized controlled clinical trial

#### S. Eladawy^1^, M. El-Hamamsy^1^, N. Bazan^2^, M. Elgendy^3^

##### ^1^Faculty of Pharmacy-Ain Shams University, Cairo, Egypt; ^2^Critical Care Medicine Department Cairo University Hospitals, Cairo, Egypt; ^3^Faculty of Medicine - Ain Shams University, Cairo, Egypt

###### **Correspondence:** S. Eladawy – Faculty of Pharmacy-Ain Shams University, Cairo, Egypt

**Introduction:** Statin therapy during intensive care unit (ICU) stay has been associated with a reduction in all-cause hospital mortality in some studies. This association was especially noted in septic patients. However, potential benefit needs to be validated in randomized, controlled trials.

**Objectives:** The purpose of this study was to compare the effect of simvastatin plus standard therapy on mortality and total ICU length of stay (LOS) to that of standard therapy alone in critically ill septic patients.

**Methods:** A prospective randomized, open label, controlled pilot clinical trial was conducted on patients diagnosed with sepsis/severe sepsis as defined by the American College of Chest Physicians (ACCP). Hundred patients met the study criteria and were randomized into two groups; a standard group who received standard treatment and simvastatin group who received the standard treatment plus 40 mg simvastatin. Primary outcomes were 28 days ICU mortality and total ICU LOS. Plasma C-reactive protein (CRP), total creatine kinase (CK) and liver enzymes [alanine aminotransferase (ALT) and aspartate aminotransferase (AST)] were measured as secondary outcome measures.

**Results:** A total of 72 patients completed the study. Simvastatin was well tolerated, with no increase in adverse events between the two groups. Total ICU LOS was significantly lower in the simvastatin group. However, the number of patients with 28 days ICU mortality in the simvastatin group was lower compared to standard group; but survival failed to reach statistical significance. Similarly, plasma C-reactive protein failed to reach statistical significance between the two groups

**Conclusions:** Treatment with simvastatin 40 mg in patients with sepsis/severe sepsis is safe and associated with an improvement in number of deaths and ICU LOS but without subsequent improvement in survival.

### A896 The use of anapnoguard 100 system in intubated critically ill patients a randomized controlled study

#### G. De Pascale, M.S. Vallecoccia, S.L. Cutuli, V. Di Gravio, M.A. Pennisi, G. Conti, M. Antonelli

##### Sacro Cuore Catholic University, A. Gemelli Hospital, Department of Anesthesiology and Intensive Care, Rome, Italy

###### **Correspondence:** G. De Pascale – Sacro Cuore Catholic University, A. Gemelli Hospital, Department of Anesthesiology and Intensive Care, Rome, Italy

**Introduction:** The AnapnoGuard 100 system (AG) (Hospitech Respiration LTD., Petach-Tikva, Israel) is an innovative respiratory guard system that continuously monitors and controls the cuff pressure by measurements of CO2 levels above the cuff, and allowing simultaneous rinsing and aspiration of subglottic secretions.

**Objectives:** To determine the safety and clinical efficacy of AG 100 system compared with usual care in critically ill patients.

**Methods:** Prospective, single centre, open-label, randomized, controlled feasibility and safety trial. Sixty patients, without pneumonia, were randomized to be intubated with the AG tube and connected to the system (n = 30) or with a conventional tube (n = 30) combined with subglottic secretion drainage and manually control of tracheal cuff pressure (P_cuff_). Primary outcome was the rate of adverse events. Other outcomes included the rate of mechanical complications, the level of ICU staff satisfaction, the incidence of ventilator-associated pneumonia (VAP), the quality of P_cuff_ control, and the amount of SS drained.

**Results:** Out of 60 patients enrolled in the study, 56 were included in the analysis (28 per each group). Both groups were similar at randomization in demographic characteristics, ICU admission diagnosis, main comorbidities and severity of illness. No device-related adverse events occurred in any of the two groups. No differences were detected using AG system vs conventional tubes in terms of post-extubation throat pain level (0[0–2] vs. 0[0–3]; *p* = 0.7), hoarseness (42.9 % vs. 75 %; p = 0.55) and tracheal mucosa oedema (16.7 % vs*.* 10 %; *p* = 0.65). On the basis of a predefined questionnaire (0–5 grading scale), ICU staff satisfaction level was high. The use of AG system was associated with a significantly higher percentage of P_cuff_ determinations in the safety range (97.3 % vs. 71.2 %; *p* < 0.001) and with a trend to a better daily subglottic secretions drainage (67.8 [20–88.7] ml vs. 50 [18.7-62] ml; *p* = 0.11). Patients enrolled in the AG group showed a lower percentage of ventilator-associated pneumonia (14.8 % vs. 40 %; *p* = 0.06) which were more frequently monomicrobial (25 % vs. 70 %; *p* = 0.2). No statistically significant between-group differences were observed in durations of mechanical ventilation, ICU stay, and mortality.

**Conclusions:** The use AG 100 system in critically ill intubated patients is safe and effective in P_cuff_ control ad SS drainage. The observed promising role as a tool to prevent VAP needs to be confirmed in a larger, adequately powered, randomized trial.

**Clinical trial** registered with www.clinicaltrials.gov (NCT01550978).

**References**

1. Vallecoccia MS, De Pascale G, Cutuli SL, Di Gravio V, Pennisi MA, Antonelli M. Endotracheal tubes cuff pressure control: does the CO2 matter? Minerva Anestesiol. 2015 Mar;81(3):352–3.

### A897 Survival impact of β-blockade in a long-term model of fluid-resuscitated sepsis depends on prognostic risk

#### D.T. Andreis^1,2^, W. Khaliq^1^, M. Singer^1^

##### ^1^Bloomsbury Institute of Intensive Care Medicine, University College London, London, United Kingdom; ^2^Dipartimento di Fisiopatologia Medico-Chirurgica e dei Trapianti, Milan, Italy

###### **Correspondence:** D.T. Andreis – Bloomsbury Institute of Intensive Care Medicine, University College London, London, United Kingdom

**Introduction:** During sepsis, intrinsic stress responses may become maladaptive and contribute to poor outcomes. Targeted intervention with β-blockade to 'de-stress' such patients may be beneficial. We developed a 72-h rodent model of fluid-resuscitated faecal peritonitis in which mortality (occurring between 18 and 42 h) can be predicted at 6 h by a low stroke volume (AUROC 0.87), and where survivors are clinically improving by study end.^[1]^

**Objectives:** To investigate the impact of β-blockade on outcomes in predicted survivors and nonsurvivors of faecal peritonitis.

**Methods:** Instrumented, fluid resuscitated, male Wistar rats (300–400 g) had sepsis induced by intraperitoneal injection of faecal slurry (8.5 ml/kg). At 6 h, under brief isoflurane sedation, echocardiography was performed to differentiate predicted survivors from nonsurvivors based on a stroke volume cut-off of 0.20 ml. Rats in each prognostic group were then randomised to receive either esmolol (500 μg/kg over 1 min followed by 75 μg/kg/min infusion) or matching placebo (0.9 % NaCl) until 24 h. Animals were observed for up to 72 h, and time of death was recorded. The study was powered to detect a mortality reduction in predicted nonsurvivors from 90 % to 45 % with esmolol, with a power of 0.80 and type-1 error of 0.05.

**Results:** 64 rats were randomised after prognostication to receive either esmolol or placebo. At 6 h, predicted survivors and nonsurvivors were clinically indistinguishable (both groups appeared only mildly unwell), though predicted nonsurvivors (stroke volume < 0.20 ml) had lower cardiac output (86 ± 9 vs. 130 ± 25 ml/min), higher heart rate (487 ± 36 vs. 459 ± 24 bpm) and blood pressure (131 ± 12 vs. 125 ± 10 mmHg) and more haemoconcentration (haemoglobin 16.9 ± 1.6 vs. 15.0 ± 1.3 g/dl) (all p < 0.05). Survival was significantly improved by esmolol in predicted nonsurvivors (p = 0.004), but worsened in predicted survivors (p = 0.06).

**Conclusions:** Mortality was approximately halved in predicted nonsurvivors by esmolol, but doubled in predicted survivors. Early prognostication appears key in identifying the subset(s) of animals (and, potentially, patients) who might benefit from additional treatment, while avoiding iatrogenic harm in those that would naturally survive. Mechanisms by which esmolol impact upon mortality are under investigation.

**References**

[1] Rudiger A et al. *Clin Sci* 2013; 124: 391–401.

**Grant acknowledgement**

ESICM Basic Science Award, UK Intensive Care Society Young Investigator Award, NIHR.

**Fig. 31 (abstract A897).**
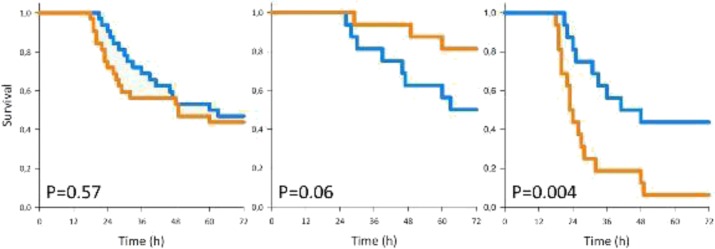
Effect of esmolol (blue) vs. placebo (orange) on mortality in the overall population (left, n = 32 per group), predicted survivors (centre, n = 16 per group) and predicted nonsurvivors (right, n = 16 per group). P values refer to log rank test

### A898 Polymyxin coated cytokine adsorbent for supportive treatment of sepsis

#### J. Hartmann, S. Harm

##### Danube University, Department for Health Sciences and Biomedicine, Krems, Austria

###### **Correspondence:** S. Harm – Danube University, Department for Health Sciences and Biomedicine, Krems, Austria

**Introduction:** Endotoxins (lipopolysaccharides, LPS) have become interesting targets in extracorporeal therapies. LPS is a major constituent of the outer cell wall of gram-negative bacteria and strongly triggers inflammatory responses in humans at concentrations as low as 1 ng/kg body weight. Although the elimination of LPS is promising for the supportive therapy of sepsis and liver failure, endotoxin neutralization using endotoxin adsorbents is controversial.

**Objectives:** We could recently show that endotoxin inactivation by low-dose Polymyxin B (PMB; 200 ng/ml) could be applied for endotoxin inactivation in blood [1]. Aim of this study was to establish an adsorbent-based system which combines constant PMB release for endotoxin inactivation and effective cytokine adsorption during extracorporeal treatment.

**Methods:** We established an adsorbent-based PMB release system which ensures a constant PMB level in plasma during extracorporeal therapies. A polystyrene-divinylbenzene based cytokine adsorbent (CG161c) with nanostructured pores was coated with a defined amount of PMB by hydrophobic interactions. The endotoxin inactivation and cytokine adsorption was tested in an *in vitro* model using fresh donated blood which was stimulated with 1 ng/ml lipopolysaccaride from *E. coli*.

**Results:** In plasma or blood an equilibration between the free and bound form of PMB will lead to a constant PMB level in plasma. The PMB release experiments in plasma clearly show that the adsorption and desorption is a function of the ratio PMB concentration: adsorbent surface. Furthermore the PMB release depends on the protein concentration of the plasma. It makes a big difference whether the PMB coated adsorbent is used in plasma or in fractionated plasma where the hydrophobicity is much lower. The experiments suggest that the PMB coating of the CG161c adsorbent doesn´t influence the cytokine removal which can take place in parallel. The ability of LPS inactivation by the PMB coated CG161c adsorbents was similar to PMB which was infused directly into the plasma.

**Conclusions:** Our *in vitro* model shows that the combination of cytokine removal and controlled PMB release by the same adsorbent results in a strong suppression of inflammatory effects in blood.

**References**

[1] Harm S, Hartmann J: Low-Dose Polymyxin: an Option for Therapy of Gram-Negative Sepsis. Innate Immunity, 2016, accepted for publication.

**Grant acknowledgement**

This work was supported by the government of Lower Austria within the project ID WST3-T-91/036-2014).

## Cardivascular Challenges In The ICU

### A899 Local intraarterial thrombolysis for the management of patients with hemodynamically stable pulmonary embolism and right ventricular dysfunction

#### S. Alcantara Carmona^1^, P. Matia Almudevar^1^, A. Naharro Abellán^1^, J. Veganzones Ramos^1^, L. Pérez Pérez, B. Lobo Valbuena, N. Martínez Sanz, I. Fernández Simón

##### Hospital Universtario Puerta de Hierro Majadahonda, Intensive Care Unit, Madrid, Spain

###### **Correspondence:** S. Alcantara Carmona –Hospital Universtario Puerta de Hierro Majadahonda, Intensive Care Unit, Madrid, Spain

**Objective:** Management of hemodynamically stable pulmonary embolism (PE) with right ventricular (RV) dysfunction is still controversial. The objective of our study is to evaluate the effectiveness of local intraarterial thrombolysis (LIT) in this group of patients and analyze its complications.

**Patients and methods:** Prospective study (January 2008-December 2105). Patients included had been diagnosed of PE by computed tomography (CT), were hemodynamically stable [systolic arterial pressure (SAP) > 90 mmHg] and had a clinical suspicion of RV dysfunction (biventricular quotient in CT > 1 or elevated levels of troponin I), that was confirmed afterwards by the presence of at least one of the following findings in the echocardiogram: subjective alteration of RV contractility, RV basal diameter (four chamber view) > 40 mm, tricuspid annular plane systolic excursion (TAPSE) < 15 mm or estimated systolic pulmonary artery pressure (SPAP) > 30 mmHg. LIT was done with a urokinase infusion (bolus dose of 200.000 UI followed by a perfusion of 100.000 UI/h) administered thru a pulmonary artery catheter, placed with radiological guidance, using an antecubital puncture. Patients received simultaneous systemic anticoagulation with unfractionated heparin. After 48–72 h of treatment, and before ending the urokinase infusion, a radiological control was done using angiography or CT. Within the seven days after LIT, patients underwent a follow-up echocardiogram. Statistical analysis was performed with Student´s T test for parametric paired data, Wilcoxon´s Test for non parametric and Stuart-Maxwell for qualitative values.

**Fig. 32 (abstract A899).**
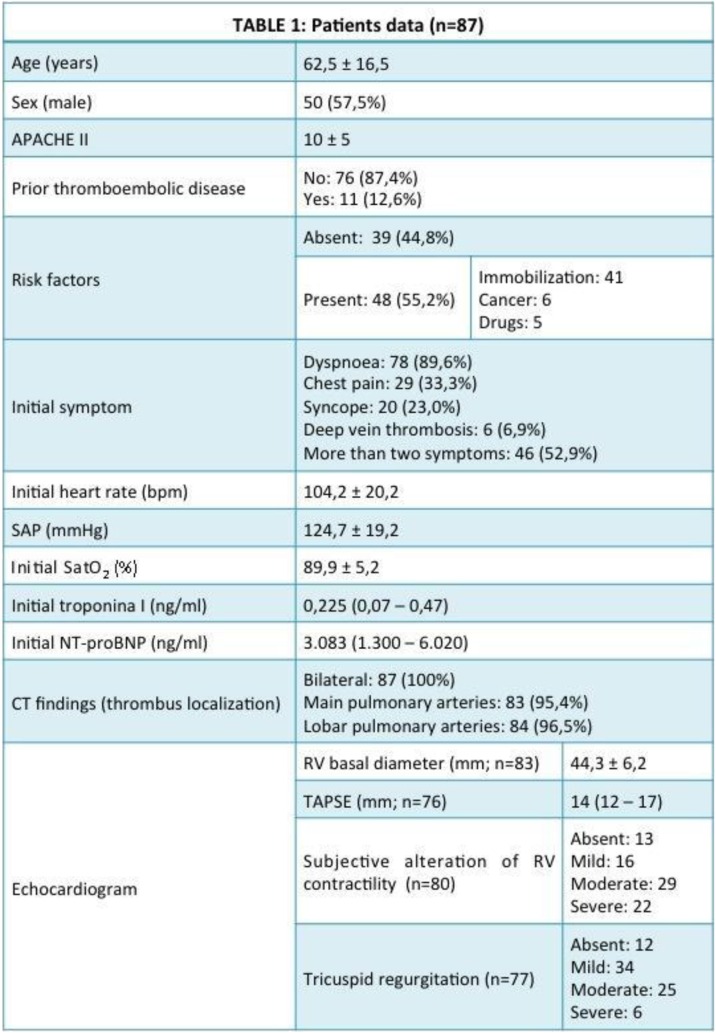
ᅟ

**Fig. 33 (abstract A899).**
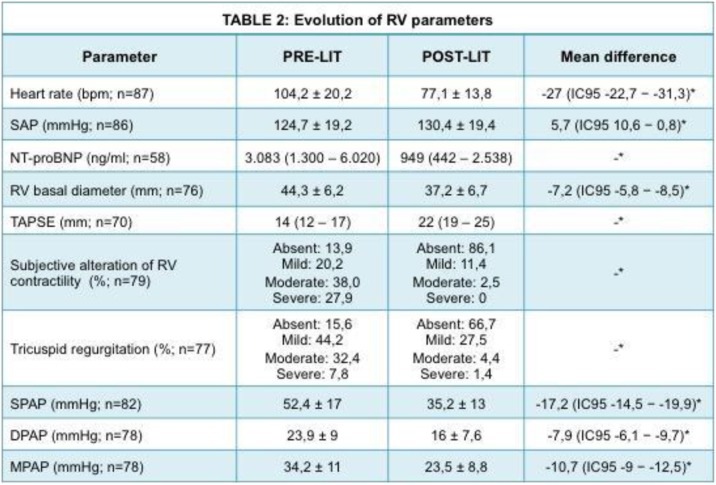
ᅟ

**Results:** Eighty-seven patients were included and their general data are detailed in Fig. 32. Mean treatment time was 56,3 ± 15,5 h. Ninety percent of patients experienced a radiological improvement (50.5 % a complete/almost complete resolution and, 37.8 % a significant improvement). Only 5,7 % didn´t improve radiologically. The evolutions of the different RV parameters studied are shown in Fig. 33. Minimum fibrinogen and platelet values where 251,9 ± 91,8 mg/dl and 134 x 10^3^ ± 44.6 x 10^3^ cells/mm^3^. Eighteen patients (20,7 %) suffered form hemorrhagic complications that, in 13 cases, where puncture site hematomas and, in six occasions (6,9 %) required an early interruption of the treatment. Three patients (3,4 %) received a blood cell transfusion of ≤ 2 blood units. Mean ICU and hospital stays where 4 ± 1,5 and 14 ± 9,2 days. All patients survived.

**Conclusion:** In our group of patients, LIT rapidly improved the function and decreased the hemodynamic strain of the RV, while being associated with a low incidence of major complications.

### A900 New-onset atrial fibrillation has detrimental effects on in-hospital but not on long-term survival in critically ill patients: a report from the FROG-ICU study

#### M. Arrigo^1^, E. Feliot^1^, N. Deye^1^, A. Cariou^2^, B. Guidet^3^, S. Jaber^4^, M. Leone^5^, M. Resche-Rigon^6^, A. Vieillard Baron^7^, M. Legrand^1^, E. Gayat^1^, A. Mebazaa^1^, from the FROG ICU Investigators

##### ^1^AP-HP, Saint Louis and Lariboisière University Hospitals, Department of Anesthesiology and Critical Care Medicine, Paris, France; ^2^AP-HP, Hôpital Cochin, Medical Intensive Care Unit, Paris, France; ^3^AP-HP, Hôpital Saint-Antoine, Medical Intensive Care Unit, Paris, France; ^4^Saint-Eloi University Hospital, Department of Anesthesiology and Critical Care Medicine, Montpellier, France; ^5^AP-HM, Hôpital Nord, Department of Anesthesiology and Critical Care Medicine, Marseille, France; ^6^AP-HP, Saint Louis University Hospital, Biostatistics and Clinical Epidemiology Research Unit Department, Paris, France; ^7^AP-HP, Hôpital Ambroise Paré, Department of Anesthesiology and Critical Care Medicine, Paris, France

###### **Correspondence:** M. Arrigo – AP-HP, Saint Louis and Lariboisière University Hospitals, Department of Anesthesiology and Critical Care Medicine, Paris, France

**Introduction:** Atrial fibrillation (AFib) is associated with higher short-term mortality in critical illness, but it is still uncertain whether AFib independently contributes to unfavorable outcome.

**Objectives:** The aim of this study was to test the hypothesis that AFib during critical illness is independently associated with increased in-hospital and long-term risk of death.

**Methods:** The FROG-ICU study was a prospective, observational, multi-center cohort study designed to investigate outcome of critically ill patients. Heart rhythm was assessed at inclusion and during ICU stay with digital ECG recordings. Among patients who had any AFib during ICU stay, new-onset and recurrent AFib were diagnosed in patients without and with previous history of AFib, respectively. Primary endpoints were in-hospital and 1-year mortality. Covariate adjusted logistic regression models and Cox proportional hazards models were used to evaluate the association between AFib and in-hospital mortality or 1-year mortality, respectively. In-hospital mortality was adjusted for 7 independent covariates (age, gender, Simplified Acute Physiology Score (SAPS II), treatment with inotropes or vasopressors, serum lactate level, high-sensitive troponin I, B-type natriuretic peptide), 1-year mortality was adjusted for 8 covariates (age, gender, SAPS II, history of congestive heart failure, treatment with inotropes or vasopressors, serum lactate level, C-reactive protein and serum creatinine).

**Results:** The study included 2087 critically ill patients. The study population consisted of 1841 patients for whom data about heart rhythm during ICU stay was available. AFib occurred in 343 patients (19 %). New-onset AFib (n = 212) had higher in-hospital mortality (47 %) compared to no AFib (23 %, *P* < 0.001) or recurrent AFib (34 %, *P* = 0.032). New-onset AFib showed increased in-hospital risk of death after multivariable adjustment compared to no AFib (OR 1.6, 95 % CI 1.2-2.2, *P* = 0.003) or recurrent AFib (OR 1.8, 95 % CI 1.1-2.9, *P* = 0.02). Among the 1464 ICU-survivors, new-onset AFib during ICU stay showed higher 1-year mortality compared to no AFib (log-rank *P* < 0.001), but similar to recurrent AFib. After multivariable adjustment, new-onset AFib showed similar 1-year risk of death compared to no AFib (HR 1.3, 95 % CI 0.9-1.8, *P* = 0.17) or recurrent AFib (HR 1.1, 95 % CI 0.7-1.7, *P* = 0.81).

**Conclusions:** New-onset AFib independently increases in-hospital but not long-term risk of death of critically ill patients.

**Grant acknowledgement**

The FROG-ICU study was supported by the national PHRC and the SFAR

### A901 Antiarrhythmic therapy for supraventricular arrhythmias in septic shock

#### M. Balik^1^, I. Kolnikova^1^, M. Maly^1^, P. Waldauf^2^, G. Tavazzi^3^, J. Kristof^1^

##### ^1^1st Medical Faculty, Charles University, Prague, Czech Republic; ^2^3rd Medical Faculty, Charles University, Prague, Czech Republic; ^3^University of Pavia, Pavia, Italy

###### **Correspondence:** M. Balik – 1st Medical Faculty, Charles University, Prague, Czech Republic

**Introduction:** The incidence of the supraventricular arrhythmias is increased in septic shock patient, and it is associated with worse short and long term prognosis.

**Objective:** To test that propafenon could be a feasible antiarrhythmic in the absence of contraindications.

**Methods:** Patients with septic shock who received antiarrhythmic drugs for supraventricular arrhythmias were included over 24 months. The patients were divided into the three groups according to antiarrhythmic agent: amiodarone (Group1), propafenon (Group2) and metoprolol (Group3). In the first 24 h the type of arrhythmia, dosages, cardioversion rates, demographic, haemodynamic, laboratory parameters were recorded. Mortality was compared between the groups and between the cardioverted vs those remaining in acute and chronic arrhythmias.

**Results:** 234 (17.3 % of all ICU) patients with rates of IPPV 99.1 % were included, 14.5 % had chronic atrial fibrillation (AF). Prevailing arrhythmia was acute onset AF(69.7 %). The rates of additional electric cardioversion to secure sinus rhythm were not different (23.7 % Group1, 35.5 % Group2, ns). Except for the dosage of noradrenaline the mortality predictors were not different between the three groups.

ICU (30.9 %) and 28-day (38.8 %) mortalities of cardioverted patients were not significantly different from mortality of chronic AF patients (ICU 38.2 %, OR 1.38, 28-day 41.2 % OR 1.11) and both were lower than the ICU (54.3 %, OR 2.64 vs sinus, OR 1.92 vs chronic AF) and 28-day mortality (65 %, OR 2.98 vs sinus, OR 2.69 vs chronic AF) of those remaining in an acute arrhythmia.

**Conclusions:** A chance to cardiovert a ventilated septic shock patient seems to be higher under propafenon than in amiodarone with a promising impact upon ICU and 28-day mortality. The patients remaining in acute onset arrhythmia demonstrated significantly higher ICU and 28-day mortality compared to those succesfully cardioverted or to those having chronic atrial fibrillation.

**Table 21 (abstract A901).** ᅟ

Logistic regression: ICU and 28-day mortality	Group1: Amiodarone (n = 142)	Group2: Propafenon (n = 78)	Group3: Metoprolol (n = 14)
APACHE II: OR 1.74 and 1.9 (p < 0.001)	26 (16–33)	23.5 (17.5–33.5)	20 (6–28)
SOFA: OR 3.79 and 2.88 (p < 0.001)	10 (8–14)	11 (7.5–14.5)	9 (4–11)
EF_LV [%]	45 (30–60)	50 (40–60) (ns)	48(35–58) (ns)
NAD [ug/kg.min]: OR 2.02 and 1.7 (p < 0.001)	0.35 (0.14–0.80)	0.25 (0.08–0.50) p < 0.01 vs Group1	0.08(0.05–0.23) p < 0.05 vs Group2, p < 0.01 vs Group1
CRRT: OR 2.57 and 3.28 (p < 0.001)	27.2 %	32.4 % (ns)	15.4 % (ns)
PCT [ng/ml]:OR 1.01,ns	3.20 (1.18–10.42)	1.2 (0.43–8.17) (ns)	7.2 (1.22–7.49) (ns)
Cardioversion rate	73.5 %	86.1 % (ns)	92.3 % (ns)
ICU mortality	40 % (OR 1.58 vs Group2, ns)	30.3 % (ns)	23.1 % (ns)
28-day mortality	49 % (OR 1.72 vs Group2, ns)	39.7 % (ns)	23.1 % (ns)

### A902 Comparison of heart rate control with intravenous ivabradine or esmolol perfusion in a large animal model of septic shock

#### A. Herpain^1,2^, F. Su^2^, E. Post^2^, F. Taccone^1,2^, J.-L. Vincent^1,2^, J. Creteur^1,2^

##### ^1^Erasme University Hospital, Université Libre de Bruxelles, Intensive Care Unit, Bruxelles, Belgium; ^2^Experimental Laboratory of Intensive Care, Université Libre de Bruxelles, Bruxelles, Belgium

###### **Correspondence:** A. Herpain – Erasme University Hospital, Université Libre de Bruxelles, Intensive Care Unit, Bruxelles, Belgium

**Introduction:** In septic shock, tachycardia and hyperdynamic hemodynamic status have been associated with a worse outcome in clinical studies; presumably due to an impairment of myocardial oxygenation and ventricular filling. A randomised control trial of heart rate (HR) control in septic shock showed an increase of survival for the patients receiving esmolol^1^. An animal study observed a similar improvement of survival and an increase in left ventricular (LV) contractility when esmolol was associated with norepinephrine (NE)^2^. However beta-blockers therapy in sepsis is still debated considering its negative inotropic side effect. Ivabradine, a pure bradycardic agent, blocking selectively the If channels in the sinus node, could represent a safer option for HR control.

**Objectives:** Compare the hemodynamic tolerance of HR control either with intravenous (IV) ivabradine or esmolol perfusion, in a large animal model of septic shock.

**Methods:** We used a closed chest swine model of fecal peritonitis. Analgesia and sedation were provided by sufentanil and sevoflurane. Hemodynamic monitoring included arterial blood pressure (ABP); continuous cardiac output (CCO); LV maximum rate of pressure (dP/dTmax) and LV elastance (E-LV); mixed venous oxygen saturation (SVO2) and arterial lactate (Lac). After the development of septic shock, fluid resuscitation was started and animals were randomised in 3 groups of 6 pigs: ivabradine (IVB), esmolol (ESM) or control. Ivabradine was administered with an IV bolus of 0,1 mg/kg that could be repeated at 0,3 mg/kg, aiming an HR between 80 and 90 beats per minute (BPM). Continuous IV perfusion of esmolol was started at 1 mg/kg/h and adapted to reach the same HR range. After 5 hours of HR control, a fixed dose of 0,3 mcg/kg/min NE was introduced in all groups.

**Results:** All animals developed an hyperdynamic distributive shock, including tachycardia above 110 bpm. HR control between 80 and 90 bpm was successful in both IVB and ESM groups. IVB administration didn't affect ABP, CCO, dP/dTmax, E-LV, SVO2 or Lac. ESM perfusion tended to decrease ABP, CCO and SVO2; E-LV and Lac were unaffected but dP/dTmax decreased markedly. Under NE perfusion, E-LV was similar in all groups but dP/dTmax was lower in ESM group.

**Conclusions:** In septic shock, HR control with an IV administration of ivabradine doesn´t alter global organs perfusion and cardiac function. Esmolol perfusion, in order to achieve the same goal, reduces LV dP/dTmax and didn´t enhance LV contractility in association with NE.

**References:**

1. Morelli A. *Effect of heart rate control with esmolol on hemodynamic and clinical outcomes in patients with septic shock: a randomized clinical trial.* JAMA. 2013; 310(16):1683–91.

2. Kimmoun A. *Beta1-Adrenergic Inhibition Improves Cardiac and Vascular Function in Experimental Septic Shock.* Crit Care Med. 2015; 43(9):e332-40.

**Grant acknowledgment**

Fonds Erasme pour la Recherche Scientifique.

Fonds pour la Chirurgie Cardiaque.

### A903 Hypotension probability algorithm accuracy on MIMIC II ICU patients

#### C. Lee^1,2^, F. Hatib^1^, Z. Jian^1^, S. Buddi^1^, M. Cannesson^3^

##### ^1^Edwards Lifesciences, Critical Care, Irvine, United States; ^2^University of California Irvine, Biomedical Engineering, Irvine, United States; ^3^University of California Los Angeles, Anesthesiology, Los Angeles, United States

###### **Correspondence:** C. Lee – Edwards Lifesciences, Critical Care, Irvine, United States

**Introduction:** Patients in critical care settings are often at risk of developing hypotension, which can lead to poor outcomes such as increased morbidity and mortality. Current hemodynamic parameters for monitoring such hypotension often exhibit pronounced changes only when the hypotensive event is already occurring or when it is too late. We have developed a hypotension probability indicator (HPI™) to predict hypotensive episodes based on machine learning techniques. The HPI™ model was trained on ~3000 ICU and OR patients. The objective of this study is two-fold: 1) To test the accuracy of HPI™ to predict events on a completely independent test data set of ICU patients, not used in the development of the algorithm; and 2) To compare timing of interventions in response to an event to the timing of detection of an event by HPI™.

**Methods:** Data used in this study came from the MIMIC II MIT Research Database. Arterial pressure waveforms of 326 patients were analyzed for HPI™ and then tested for event detection and prediction accuracy. All features of the HPI™ as well as other hemodynamic parameters for comparison were calculated using FloTrac (Edwards Lifesciences, Irvine, CA). A hypotensive event was defined as any time period where MAP < 65 mmHg for at least 1 minute. An ROC analysis was performed to assess AUC, sensitivity, and specificity of the HPI™ to identify an event during the event, and 5, 10, and 15 minutes prior to the start of event.

Next, clinical records of the 326 patients were reviewed for any drug or fluid interventions during start of event to 5 minutes after an event and the elapsed time from start of event to intervention time was calculated. A drug or fluid intervention was defined as any bolus or IV infusion start. In addition, the time at which HPI™ probability of event > 0.85 prior to the start of an event was also calculated for comparison. Data are presented in median [25- 75^th^ percentiles].

**Fig. 34 (abstract A903).**
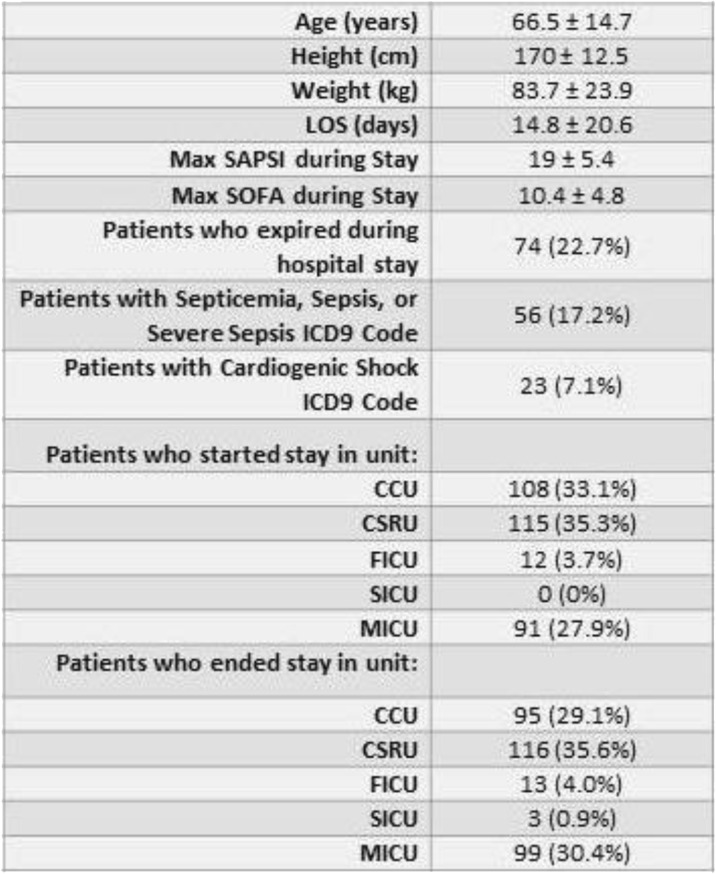
Patient demographics (n = 236). Data represented as #(% of total patients) OR mean ± standard deviation. SAPSI and SOFA scores were calculated by the MIMIC II MIT Database. CCU = Coronary Care Unit; CSRU = Cardiac Surgery Recovery Unit; FICU = Finard Medical Surgical ICU; SICU = Surgical ICU; MICU = Medical ICU. It should also be noted that 60 patients had empty ICD9 records.

**Fig. 35 (abstract A903).**
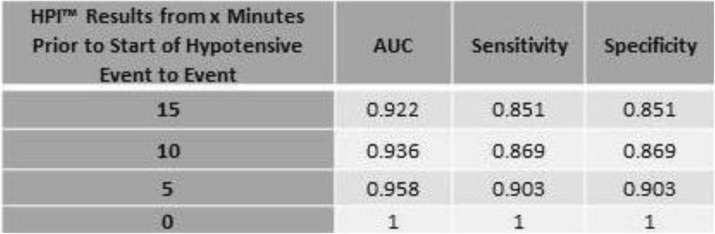
AUC, Sensitivity, and Specificty Results for the hypotension probability indicator (HPI^TM^) on MIMIC II ICU Patients. Results were calculated for the HPI‘s^TM^ ability to correctly detect a hypotensive event 15 minutes prior to the start of a defined event, 10 minutes, 5 minutes, and 0 minutes

**Results:** Patient demographics are presented in Fig. 34. Overall, hypotensive events had a duration of 2.0 [0.7-6.3] minutes. HPI™ can accurately detect an event up to 15 minutes prior to the start of an event with high sensitivity and specificity (>0.85) (Fig. 35). In addition, out of 26,021 total number of events, 15.4 % of events had a drug or fluid intervention start sometime between the start of an event and 5 minutes after the end of an event. Of these interventions, the median start time of intervention was 4.7 [2.3-10.2] minutes after the start of an event. In comparison, HPI™ detected 99.99 % of events and detected at 17.3 [4.7-53.7] minutes before the start of an event.

**Conclusion:** In conclusion, HPI™ can accurately detect an event up to 15 minutes prior. HPI™ may serve as a useful addition in the care of critically ill patients by potentially facilitating earlier intervention either in response to an event or serve as a decision support and direct a physician's attention to potential oncoming events when HPI™ is high.

## Post-Cardiac Arrest Care

### A904 An estimation of the number of successful resuscitations after in hospital cardiac arrest in one single country and possibilities for improvement

#### F. Hessulf^1^, P. Lundgren^2^, J. Herlitz^2,3^, J. Engdahl^2,4^

##### ^1^Halland Hospital Halmstad, Department of Intensive Care Medicine, Halmstad, Sweden; ^2^Sahlgrenska University Hospital, Inst of Internal Medicine, Department of Molecular and Clinical Medicine, Gothenburg, Sweden; ^3^University of Borås, The Center for Prehospital Care in Western Sweden, Borås, Sweden; ^4^Halland Hospital Halmstad, Department of Internal Medicine, Halmstad, Sweden

###### **Correspondence:** F. Hessulf – Halland Hospital Halmstad, Department of Intensive Care Medicine, Halmstad, Sweden

**Introduction:** Despite the development of the chain of survival, the majority of patients suffering a cardiac arrest (CA) do not survive. This statement is valid for both in-hospital as well as out-of-hospital cardiac arrest. Regardless of the location of the cardiac arrest, there are at least four factors that appear to be of major importance for survival. The first is the time from collapse to delivery of treatment; the second is the quality of cardiopulmonary resuscitation (CPR); the third is the patient's co-morbidity and the fourth is the aetiology of the CA and the presenting rhythm. The present study will focus on the first three parts of the chain of survival, time from collapse to call/CPR/defibrillation.

**Objectives:** To describe the number of survivors following in-hospital cardiac arrest (IHCA) in Sweden during one year and, based on estimations and assumptions, calculate the potential number of additional lives saved following improvements in the chain of survival.

**Methods:** A retrospective register study based on the Swedish Register of Cardiopulmonary resuscitation 2005–2014. The inclusion criterions were: a confirmed in-hospital cardiac arrest where resuscitation was initiated among patiens aged >18 years. Based on the chance of survival in relation to the delay from collapse to call for the rescue team and start of treatment, we estimated the potential number of lives that could be saved if the delay to call and treatment was reduced to the treatment recommendations by the Swedish Resuscitation Council.

**Results:** We estimate that in 2014 resuscitation was initiated on 2850 adult patients (>18 years) suffering IHCA. This corresponds to 14 IHCA cases per 100 hospital beds and year. 30-day survival was 31 % i.e. 900 survivors (four survivors per 100 hospital beds). There was a strong inverse relation between delay to call for the rescue team and delay to treatment and survival. If delay from collapse to a/call and, b/start of CPR were reduced to < 1 minute in patients with a longer delay than that and if c/time from collapse to defibrillation was reduced to < 3 minutes among those with a longer delay than that: a/57; b/32; and c/35 further lives could potentially be saved. We speculate that about 100 additional lives (one per 200 hospital beds each year) could theoretically be saved by improved adherence to guidelines regarding the first three components in the chain of survival in Swedish hospitals yearly.

**Conclusions:** In 2014, approximately 900 patients (four per 100 hospital beds) were successfully resuscitated following IHCA in Sweden. There was a strong negative relation between collapse and call for rescue team/CPR/defibrillation and 30-day survival. With reduced delay times a further 100 lives (one per 200 hospital beds) could theoretically be saved each year in Sweden.

**Grant acknowledgment**

The study was supported by grants from the Laerdal Foundation of Acute Medicine in Norway (JH) and the Scientific Council of Halland (FH).

### A905 Prophylactic versus clinically-driven antibiotics in comatose survivors of out-of-hospital cardiac arrest - a pilot study

#### S. Fileković^1^, M. Turel^2^, R. Knafelj^3^, V. Gorjup^3^, R. Stanić^1^, P. Gradišek^1^, O. Cerović^1^, T. Mirković^1^, M. Noč^3^

##### ^1^University Medical Centre Ljubljana, Department of Anaesthesiology and Surgical Intensive Therapy, Ljubljana, Slovenia; ^2^University Medical Center Ljubljana, Department of Pulmonary Diseases and Allergy, Ljubljana, Slovenia; ^3^University Medical Center Ljubljana, Center for Intensive Internal Medicin, Ljubljana, Slovenia

###### **Correspondence:** S. Fileković – University Medical Centre Ljubljana, Department of Anaesthesiology and Surgical Intensive Therapy, Ljubljana, Slovenia

**Introduction:** Benefit of prophylactic antibiotics, which may suppress development of postresuscitation infection and especially early onset pneumonia and thereby decrease the severity of postresuscitation systemic inflammatory response, is controversial.

**Objectives:** We investigated potential benefits of prophylactic antibiotic treatment in comatose survivors of out-of-hospital cardiac arrest (OHCA).

**Methods:** Among 83 patients undergoing admission bronchoscopy from September 2013 to February 2015, 60 patients without tracheobronchial aspiration were randomized to prophylactic (Amoxicillin-Clavulanic acid 1.2 g every 8 h) or clinically-driven antibiotics administered if signs of infection developed.

**Results:** Proportion of patients on antibiotics was significantly greater from day 1 to 5 in prophylactic group while there was no difference on days 6 to 7. Peak C-reactive protein in prophylactic group was significantly smaller (186 ± 61 vs. 229 ± 60 mg/L; p = 0.04). There was no difference in peak white blood cell count (14.6 ± 6.6 vs. 16.6 ± 6.2; p = 0.24), procalcitonin (4.02 ± 10.12 vs. 4.84 ± 8.5 microg/L; p = 0.80) and CD 64. Except for positive mini BAL on day 3 (7 % vs. 42 %; p < 0.01), there was no significant impact on other microbiological samples and X-ray signs of pneumonia (50 % in each group). Use vasopressors/inotropes (93 % in each groups), duration of mechanical ventilation (5.4 ± 3.7 vs. 5.2 ± 3.1 days), tracheal intubation (6.5 ± 4.6 vs. 5.9 ± 4.3 days), ICU stay (7.7 ± 5.2 vs. 6.9 ± 4.5 days), survival (73 % vs. 73 %) and survival with good neurological outcome (50 % vs. 40 %) were also comparable.

**Conclusions:** Tracheobronchial aspiration was documented in more than a quarter of comatose survivors of OHCA using bronchoscopy on admission. In the absence of aspiration, prophylactic antibiotics reduced peak CRP and the incidence of positive mini-BAL on day 3 and had no significant impact on other microbiological samples, incidence of pneumonia, ICU treatment and outcome.

**References**

1. Tomte O, Andersen GO, Jacobsen D, Draegni T et al. (2011) Strong and weak aspects of an established post-resuscitation treatment protocol - A five year observational study. Resuscitation 82:1186–93

2. Stub D, Hengel C, Chan W, Jackson D, Sanders K et al. (2011) Usefulness of cooling and coronary catheterization to improve survival in out-of-hospital cardiac arrest. Am J Cardiol 107:522–7

3. Nielsen N, Wetterslev J, Cronberg T et al. (2013) Targeted temperature management at 33 C versus 36 C after cardiac arrest. N Engl J Med 369: 2197–206

4. Kocjancic ST, Jazbec A, Noc M (2014) Impact of intensified postresuscitation treatment on outcome of comatose survivors of out-of-hospital cardiac arrest according to initial rhythm. Resuscitation 85:1364–9

**Grant acknowledgment**

Suada Fileković received educational grant "Innovative scheme to co-finance doctoral studies" number 291–499 by Ministry of higher education, science and technology, Republic of Slovenia.

### A906 Aetiology of cardiac arrests in hospital general wards

#### J. Tirkkonen^1,2^, H. Hellevuo^3^, K.T. Olkkola^4^, S. Hoppu^2^

##### ^1^Seinäjoki Central Hospital, Department of Anaesthesiology and Intensive Care Medicine, Seinäjoki, Finland; ^2^Tampere University Hospital, Department of Intensive Care Medicine, Tampere, Finland; ^3^Tampere University Hospital, Department of Emergency Medicine, Tampere, Finland; ^4^Helsinki University Hospital, Department of Anaesthesiology, Intensive Care, Emergency Care and Pain Medicine, Helsinki, Finland

###### Correspondence: J. Tirkkonen – Seinäjoki Central Hospital, Department of Anaesthesiology and Intensive Care Medicine, Seinäjoki, Finland

**Introduction:** Survival to discharge after in-hospital cardiac arrest (IHCA) is poor (10 − 20 %) and has not improved despite developments in modern medicine.^1^ Data on the aetiology of in-hospital cardiac arrests is very limited, and conducted studies include IHCA patients resuscitated in emergency departments, intensive care units and high dependency units.

**Objectives:** To determine the underlying causes of IHCAs occurring on general wards and investigate, whether the aetiology is independently associated with six months survival.

**Methods:** A prospective observational study between 2009–2011 in a Finnish university hospital. We included all adult IHCA patients on general wards who were attended by ICU´s medical emergency team. Definite aetiology was determined from the autopsy records and medical records. No autopsies were conducted solely for study purposes. The local Ethics Committee approved the study protocol (Approval no: R08116).

**Results:** The cohort consisted of 279 patients, of which 185 (66 %) were male. Median age of the patients was 72 (64, 80) years. Altogether 178 (64 %) IHCAs were monitored/witnessed, first rhythm was shockable in 42 (15 %) cases and 53 (19 %) patients survived six months. Autopsy was conducted in 153 (55 %) cases. Aetiology was determined as cardiac in 141 events, 73 of which were due to acute myocardial infarction and 26 due to acute myocardial ischaemia without infarction. Congestive heart failure was the third most prevalent reason in cardiac sub cohort (16). Altogether 138 IHCAs were considered non-cardiac; most common causes were pneumonia (39), exsanguination (16), pulmonary embolism (12) and peritonitis (11). Cardiac IHCAs were more commonly preceded by subjective symptoms (e.g. chest pain, respiratory distress) than non-cardiac IHCAs (47 % vs. 32 %, *p* = 0.022), while objective vital dysfunctions preceded IHCAs as often in both sub cohorts (40 % vs. 44 %, *p* = 0.448). In a multivariate logistic regression model monitored/witnessed event, shockable primary rhythm and low age-adjusted Charlson comorbidity index score were factors independently associated with 180-day survival, but the aetiology (cardiac vs. non-cardiac) was not.

**Conclusions:** Aetiology of IHCAs on general wards is cardiac in 50 % of the events. Ischaemic reasons for IHCAs were twice as common as shockable primary rhythms in this study. Subjective symptoms and objective vital dysfunctions often precede general ward IHCAs. However, neither the aetiology nor the presence of antecedents, but low comorbidity, observed arrest and shockable primary rhythm are factors associated with a favorable outcome.

**References**

1. Nolan J, Soar J, Smith GB, et al. Incidence and outcome of in-hospital cardiac arrest in the United Kingdom National Cardiac Arrest Audit. Resuscitation 2014;85:987–92.

### A907 Reducing in-hospital cardiac arrest by implementation of innovative early warning information system in a tertiary medical center

#### K.-C. Lin^1^, W.-T. Hung^1^, C.-C. Chiang^2^, W.-C. Huang^1^, W.-C. Juan^3^, S.-C. Lin^1^, C.-C. Cheng^4^, P.-H. Lin^3^, K.-Y. Fong^3^, D.-S. Hou^3^, P.-L. Kang^4^, S.-R. Wann^3^, Y.-S. Chen^5^, G.-Y. Mar^4^, C.-P. Liu^2^

##### ^1^Kaohsiung Veterans General Hospital, Department of Critical Care Medicine, Kaohsiung City, Taiwan, Province of China; ^2^Kaohsiung Veterans General Hospital, Cardiovascular division, Kaohsiung City, Taiwan, Province of China; ^3^Kaohsiung Veterans General Hospital, Department of Emergency, Kaohsiung City, Taiwan, Province of China; ^4^Kaohsiung Veterans General Hospital, Cardiovascular Division, Kaohsiung City, Taiwan, Province of China; ^5^Kaohsiung Veterans General Hospital, Department of Internal Medicine, Kaohsiung City, Taiwan, Province of China

###### **Correspondence:** W.-T. Hung – Kaohsiung Veterans General Hospital, Department of Critical Care Medicine, Kaohsiung City, Taiwan, Province of China

**Introduction:** In-hospital cardiac arrest (IHCA) is a common and high-risk issue with less than 20 % surviving to hospital discharge. Most patients show signs of clinical deterioration in the hours before IHCA. As a result, the development of vital sign-based early warning system was designed to detect early signs of clinical deterioration before IHCA attack in order to trigger early intensive care.

**Objectives:** In this study, we investigate the impact of the implementation of an innovative early warning information system on the rate of IHCA and survival rate in IHCA patients.

**Methods:** A multidisciplinary team among intensivists, cardiologists, emergency physicians, and nursing staffs in a tertiary medical center was organized since May 2015. The key interventions include automatic national early warning score (NEWS) calculating information system, nurses and physicians computer-based reminding alarm if NEWS ≥ 7 or more than highest scores among previous 3 measurements, real time early warning screen saver and electric board, in service education and early warning monitor team. All patients admitted between January 2013 and January 2016 were enrolled. Total 143,450 patients were divided into three groups: pre-interventional group from Jan 2013 to April 2015 (n = 107,437), Interventional group from May to June 2015 (n = 7,923) and post-interventional group from July 2015 to Jan 2016 (n = 28,090). The definition of In-hospital cardiac arrest is the number of In-hospital cardiac arrest per thousand admitted patients. We compared the rates of IHCA, 48 hours survival rate and discharge survival rate in IHCA patients among these 3 groups.

**Results:** The rate of In-hospital cardiac arrest improved from 2.53‰ in pre-interventional group, to 2.15‰ in interventional group and to 1.32‰ in post-interventional group (p < 0.05). The 48 hours survival rate in IHCA patients increased from 34.4 % in pre-interventional group, to 41.2 % in interventional group and to 45.4 % in post-interventional group (p < 0.05). The discharge survival rate in IHCA patients also increased from 15.6 % in pre-interventional group, to 29.4 % in interventional group and to 36.4 % in post-interventional group (p < 0.05).

**Conclusions:** The study demonstrated that implementation of early warning information system and innovative strategies could attenuate the rate of IHCA, 48 hours survival rate and discharge survival rate in IHCA patients.

**Grant acknowledgment**

None

### A908 Delayed awakening after cardiac arrest: prevalence and risk factors in the Parisian registry

#### M. Paul^1^, W. Bougouin^1^, G. Geri^1^, F. Dumas^2^, B. Champigneulle^1^, S. Legriel^3^, J. Charpentier^1^, J.-P. Mira^1^, C. Sandroni^4^, A. Cariou^1^

##### ^1^ICU Cochin Hospital, Paris, France; ^2^Emergency Department, Cochin Hospital, Paris, France; ^3^ICU Mignot Hospital, Versailles, France; ^4^Anesthesiology and Intensive Care, Catholic University School of Medicine, Rome, Italy

###### **Correspondence:** M. Paul – ICU Cochin Hospital, Paris, France

**Introduction:** Although prolonged unconsciousness after cardiac arrest (CA) is a sign of poor neurological outcome, limited evidence shows that a late recovery may occur in a minority of patients.

**Objectives:** We investigated the prevalence and the predictive factors of delayed awakening in comatose CA survivors treated with targeted temperature management (TTM).

**Methods:** Retrospective analysis of the Parisian Region Out-of-Hospital CA Registry (2008–2013). In adult comatose CA survivors treated with TTM, sedated with midazolam and fentanyl, time to awakening was measured starting from discontinuation of sedation at the end of rewarming. Awakening was defined as delayed when it occurred after more than 48 h.

**Results:** A total of 326 patients (71 % male, mean age 59 ± 16 years) were included, among whom 194 awoke. Delayed awakening occurred in 56/194 (29 %) patients, at a median time of 93 h (IQR 70–117) from discontinuation of sedation. In 5/56 (9 %) late awakeners, pupillary reflex and motor response were both absent 48 h after sedation discontinuation. In multivariate analysis, age over 59 years (OR 2.1, 95 % CI 1.0-4.3), post-resuscitation shock (OR 2.6 [1.3-5.2]), and renal insufficiency at admission (OR 3.1 [1.4- 6.8]) were associated with significantly higher rates of delayed awakening.

**Conclusions:** Delayed awakening is common among patients recovering from coma after CA. Renal insufficiency, older age, and post-resuscitation shock were independent predictors of delayed awakening. Presence of unfavorable neurological signs at 48 h after rewarming from TTM and discontinuation of sedation did not rule out recovery of consciousness in late awakeners.

**Grant acknowledgment**

None

**Note:** This abstract has been previously published and is available at [1]. It is included here as a complete record of the abstracts from the conference.

**References**

1. Paul M, Bougoun W, Geri G, Dumas F, Chapigneulle B, Legriet S, Charpentier J, Mira JP, Sandroni C, Cariou A (2016) Delayed awakening after cardiac arrest: prevalence and risk factors in the Parisian registry. Intensive Care Med 42(7) 1128–1136.

## Host Response In Sepsis

### A909 Discovery and validation of a host immune response gene expression signature for viral systemic inflammation

#### J. Zimmerman^1^, E. Sullivan^1^, M. Noursadeghi^2^, B. Fox^3^, D. Sampson^3^, L. McHugh^3^, T. Yager^3^, S. Cermelli^3^, T. Seldon^3^, S. Bhide^3^, R.A. Brandon^3^, R.B. Brandon^3^

##### ^1^University of Washington School of Medicine, Seattle Children's Hospital, Pediatric Critical Care Medicine, Seattle, United States; ^2^University College London, Faculty of Medical Sciences, Division of Infection & Immunity, London, United Kingdom; ^3^Immunexpress, Seattle, United States

###### **Correspondence:** J. Zimmerman – University of Washington School of Medicine, Seattle Children's Hospital, Pediatric Critical Care Medicine, Seattle, United States

**Introduction:** Viral infections play a key role in preventable deaths of children globally, and can be antecedents to bacterial pneumonia and sepsis. Diagnosis of viral infection is often problematic due to non-specific clinical presentation. We developed a host immune response gene expression signature to distinguish systemic inflammation due to viral infection vs. bacterial or non-infectious causes.

**Objectives:** To define and validate the host immune response gene expression signature against multiple independent datasets.

**Methods:** Four public GEO datasets describing transcriptomic responses to viral infection were used to identify biomarkers, ranked by AUC, which could separate affected from unaffected subjects. Biomarkers that also responded (AUC > 0.80) to non-viral causes of systemic inflammation were removed. Remaining biomarkers were then ranked for performance in 10 other GEO transcriptomic datasets for viral infection; those with mean AUC >0.75 were retained. Next, a greedy search was applied to the merged (4 + 10) viral GEO datasets to identify the best combinations of biomarkers for discrimination of viral infection. The signature was then validated using independent datasets.

**Fig. 36 (abstract A909).**
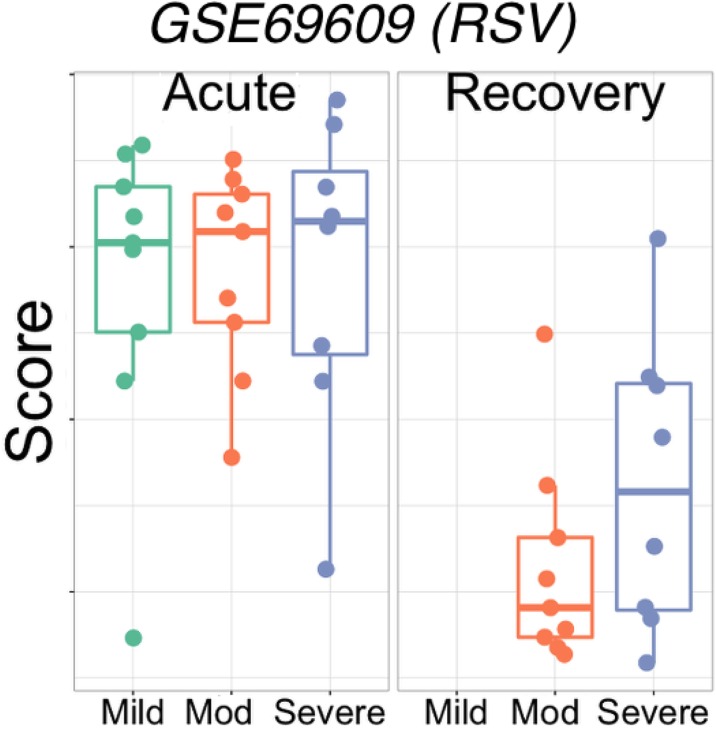
Performance of viral signature in Geo GSE69609 (RSV infection)

**Fig. 37 (abstract A909).**
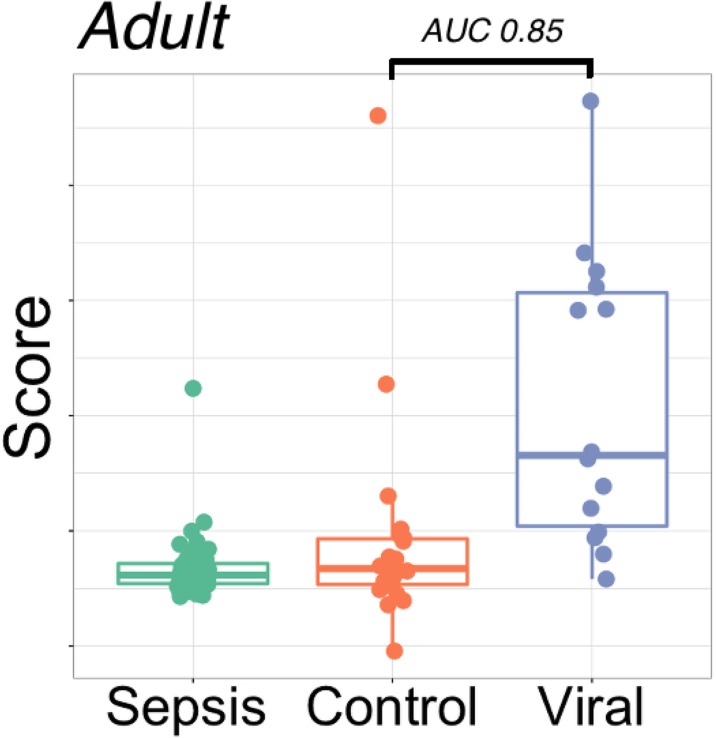
Performance of viral signature in adults from the emergency department

**Fig. 38 (abstract A909).**
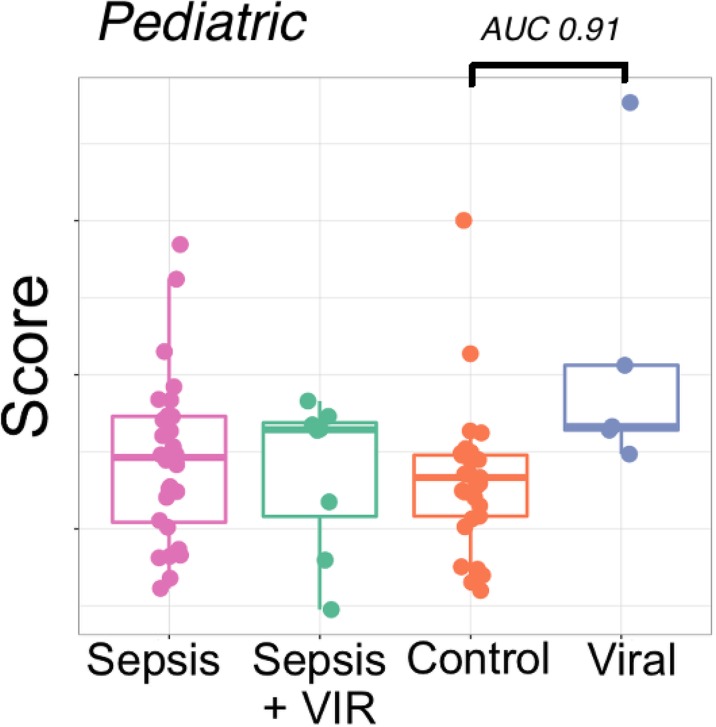
Performance of viral signature in children from intensive care

**Results:** A 4-gene signature (comprised of *ISG15, IL16*, *OASL*, ADGRE5) had AUC 0.94 across the merged (4 + 10) viral GEO datasets. This signature was validated in 11 additional GEO datasets covering a wide variety of viral pathogens including a time-course study of respiratory syncytial virus (RSV) in children (Fig 36), and in two independent datasets of our own: adults from the emergency department (Fig 37) and children from intensive care (Fig 38). Comparative performance: AUC = 0.85 (95 % CI 0.72-0.99) for adults *(*Fig 37*, 22 control vs. 14 viral)*, and AUC = 0.91 (95 % CI 0.80-1.00) for children *(*Fig 38*, 29 control vs. 5 viral),* when comparing patients retrospectively diagnosed with either a viral infection or no infection. In summary, the signature performs well in GEO datasets for HIV-1, fluA, RSV, measles, herpes viruses, hepatitis C and E, and Marburg virus; across five different mammalian species; and in human pediatric and adult populations with systemic inflammation.

**Conclusions:** We have discovered and validated a 4-gene host-immune response signature for differentiating systemic inflammation driven by viral vs. other causes. Following further validation, the signature may have clinical utility in combination with other host response signatures for diagnosing and managing patients with systemic inflammation.

**Grant acknowledgment**

Funded by Immunexpress, Seattle Children's Research Institute, and the UK National Institute for Health Research.

### A910 Remote ischemic preconditioning does not influence the innate immune response during human endotoxemia

#### J. Zwaag, R. Beunders, P. Pickkers, M. Kox

##### Radboud University Medical Center, Intensive Care Research, Nijmegen, Netherlands

###### **Correspondence:** J. Zwaag – Radboud University Medical Center, Intensive Care Research, Nijmegen, Netherlands

**Introduction:** Using a tourniquet to temporary cut off blood supply to the arm (Remote Ischemic Preconditioning - RIPC) has been shown to result in myocardial protection and reduced incidence of AKI in patients undergoing cardiac surgery. However, a recently performed large multi-center trial in CABG patients showed no beneficial effects on clinically relevant endpoints [1]. Animal studies have shown an `early window of protection' in the 1–2 hours after RIPC as well as a `late window of protection` 12–24 hours after RIPC. Several mechanisms have been suggested to mediate the protective effects of RIPC, of which attenuation of the immune response is an important candidate, although this has hitherto also only been shown in animal studies [2].

**Objectives:** To determine the effect of single and repeated RIPC, thereby investigating both the early and late windows of protection, on the inflammatory response during endotoxemia, a standardized, controlled model of systemic inflammation in humans in vivo.

**Methods:** We performed a randomized controlled study in 30 healthy non-smoking male volunteers. Subjects were assigned to either the single-dose RIPC group, multiple-dose RIPC group, or the control group (n = 10 per group). The single-dose RIPC group received 1 dose of RIPC, consisting of 4 cycles of 5-minute ischemia of the arm followed by 5 minutes of reperfusion just before administration of 2 ng/kg lipopolysaccharide (LPS). The multiple-dose RIPC group received one dose of RIPC per day on the 6 days before the endotoxemia experiment day, and 1 dose just before LPS administration.

**Fig. 39 (abstract A910).**
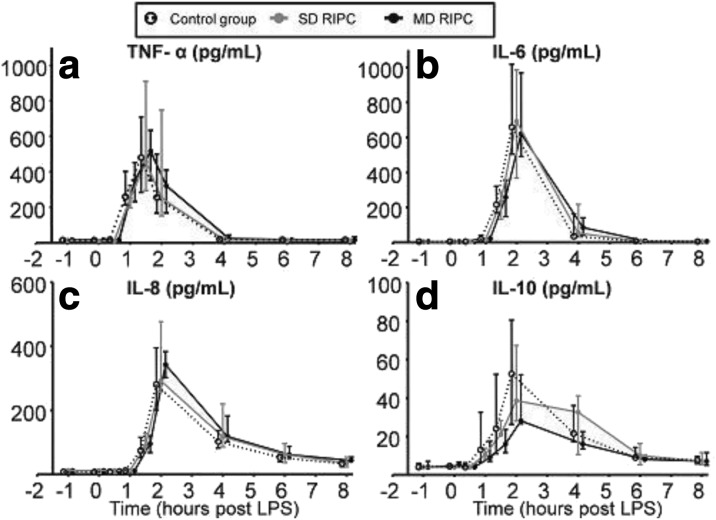
Plasma concentrations of (A) tumor necrosis factor (TNF)α, (B) interleukin (IL)-6, (C) IL-8 and (D) IL-10 during LPS-induced systemic inflammation. Data are expressed as medians with IQR. Differences between groups were evaluated using 2-way ANOVAs on log-transformed data. No significant differences were found. RIPC: Remote Ischemic Preconditioning, SD: Single Dose, MD: Multiple Dose

**Results:** LPS administration resulted in a typical increase in body temperature, flu-like symptoms, and hemodynamic changes, with no differences between groups. Administration of LPS resulted in a sharp increase in plasma levels of the pro-inflammatory cytokines TNF-α, IL-6, and IL-8 as well as the anti-inflammatory cytokine IL-10. No differences in plasma levels of these cytokines were observed between the different groups (Figure 39).

**Conclusions:** In the present study, we demonstrate that RIPC does not affect the in vivo inflammatory response induced by administration of endotoxin in humans. These results implicate that RIPC does not exert direct anti-inflammatory effects and that the previously observed protective effects are mediated through other mechanisms. Furthermore, the absence of immunomodulatory effects of RIPC in the present study tempers expectations of using RIPC as an immunomodulatory treatment strategy in patients.

**References**

1. Meybohm, P., et al., A Multicenter Trial of Remote Ischemic Preconditioning for Heart Surgery. N Engl J Med, 2015. 373(15): p. 1397–407.

2. Cai, Z.P., et al., Remote ischemic preconditioning confers late protection against myocardial ischemia-reperfusion injury in mice by upregulating interleukin-10. Basic Res Cardiol, 2012. 107(4): p. 277.

### A911 Does stem cell applications in sepsis can promote the regulatory t cell mediated immune response?

#### F. Gul^1^, M.K. Arslantas^1^, D. Genc^2^, N. Zibandah^2^, L. Topcu^1^, T. Akkoc^2^, I. Cinel^1^

##### ^1^Marmara University, Anaesthesiology and Reanimation, Istanbul, Turkey; ^2^Marmara University, Pediatric Immunology, Istanbul, Turkey

###### **Correspondence:** F. Gul – Marmara University, Anaesthesiology and Reanimation, Istanbul, Turkey

**Introduction:** Sepsis-induced immune alterations are associated with secondary infections and increased risk of death **(1)**. Mesenchymal Stem Cells (MSCs) have been described as a novel therapeutic strategy for the treatment of diseases related to inflammation and tissue injury with their potent modulatory effects on immune system **(2).**

**Objectives:** In this study, we evaluated the immune-modulatory effects of Human Dental Follicle Mesenchymal Stem Cells (HD-MSCs) on lymphocytes which are isolated from peripheral blood samples of sepsis and septic shock patients.

**Methods:** According to the International Sepsis Definitions Conference**(3)**, patients divided into two groups as sepsis (Group I, n = 10) and septic shock (Group II, n = 10). Peripheral Blood Mononuclear Cells (PBMCs) were isolated from venous blood samples of Group I, Group II and healthy subjects named as Group III, n = 10. Anti-CD3/CD28 PBMCs were co-cultured with DF-MSCs, IFN-g stimulated DF-MSCs and with no MSCs about 72 hour. CD4 + CD25 + FoxP3+ T cells levels (Treg), lymphocyte proliferation and apoptosis were evaluated with the flow cytometry.

**Results:** DF-MSCs and IFN-g induced DF-MSCs cultures significantly supressed proliferation in sepsis group when compare to septic shock group(p < 0,005).

**Conclusions:** MSCs demonstrate their effects on immune system by increasing the number and activity of regulatory T cells (Treg) (**4)** .In our study, MSCs suppressed lymphocyte proliferation and apoptosis but increased the rate of Treg cells in sepsis co-cultures. This effect was more obvious with IFN -g stimulation. These responses were not seen in septic shock patients´ blood samples and might be explained with anergy. Our findings revealed that DF-MSCs application has immunoregulatory effects in sepsis. This approach opened a new area to work how will MSCs be used to reduce organ dysfunctions and mortality in the clinical practice.

**References**

1) Jesús FBM, David A, Raquel A,Eduardo T. Defining immunological dysfunction in sepsis: A requisite tool for precision medicine. Journal of Infection; 2016.doi:10.1016/j.jinf.2016.01.010 2)

2) Shirley HJM, Jack JH, Claudia CD, et all. Mesenchymal Stem Cells Reduce Inflammation while Enhancing Bacterial Clearance and Improving Survival in Sepsis. American Journal of Respiratory and Critical Care Medicine, 2010; 8:1047–1057.

3) Levy MM, Fink MP,Marshall CJ et al., 2001SCCM/ESICM/ACCP/ATS/SIS International Sepsis Definitions Conference. Intensive Care Medicine, 2003; 29:530–538

4) Wang Y ,Zhang A ,Ye Z,et all Bone Marrow Derived Mesenchymal Stem Cells Inhibit Acute Rejection of Rat Liver Allografts in Association With Regulatory T-Cell Expansion. Transplantation, 2009; 41:10, 4352–4356

**Fig. 40 (abstract A911).**
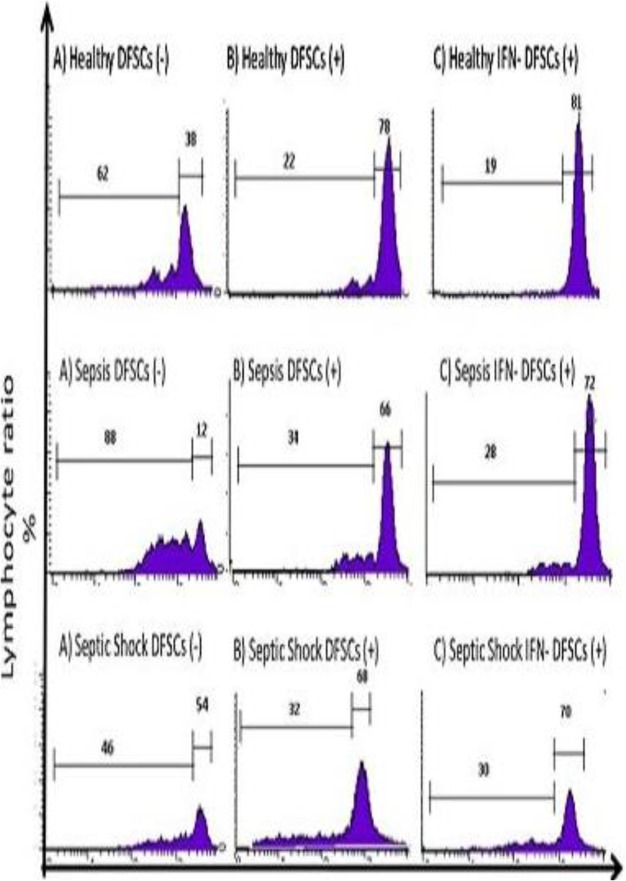
DF-MSCs and IFN-g induced DF-MSCs cultures significantly increased Treg ratio in sepsis but this effect was not observed in septic shock group

**Fig. 41 (abstract A911).**
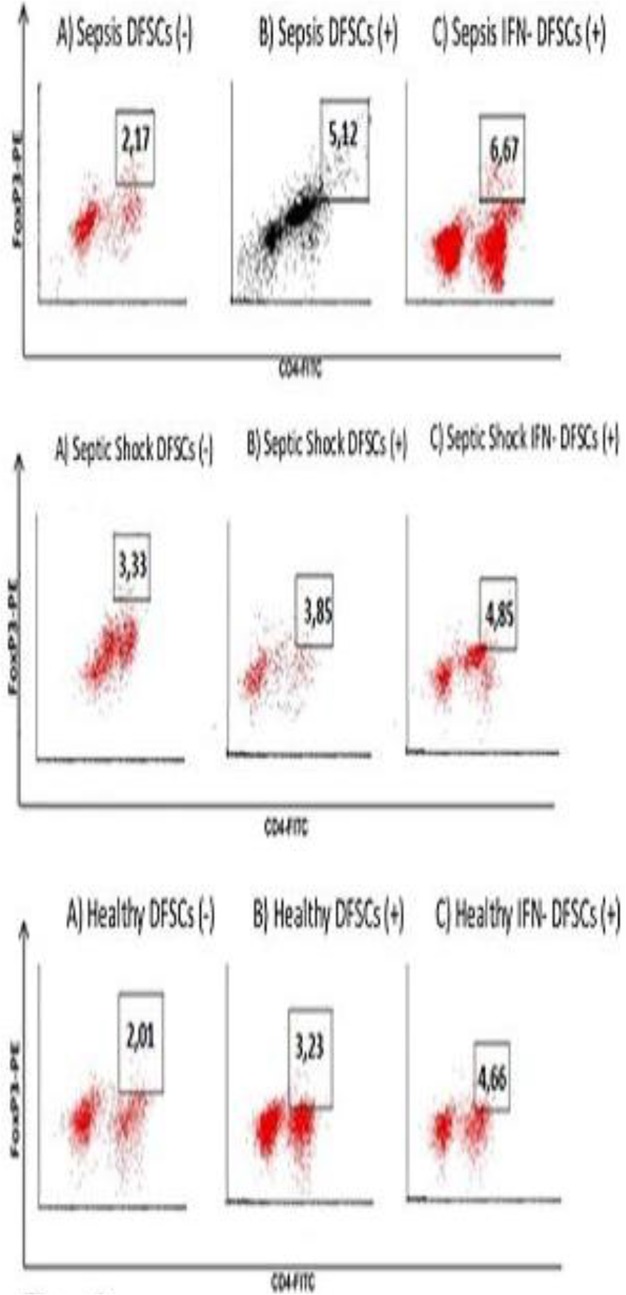
Reduction of lymphocyte apoptosis was observed in both sepsis and septic shock groups

**Fig. 42 (abstract A911).**
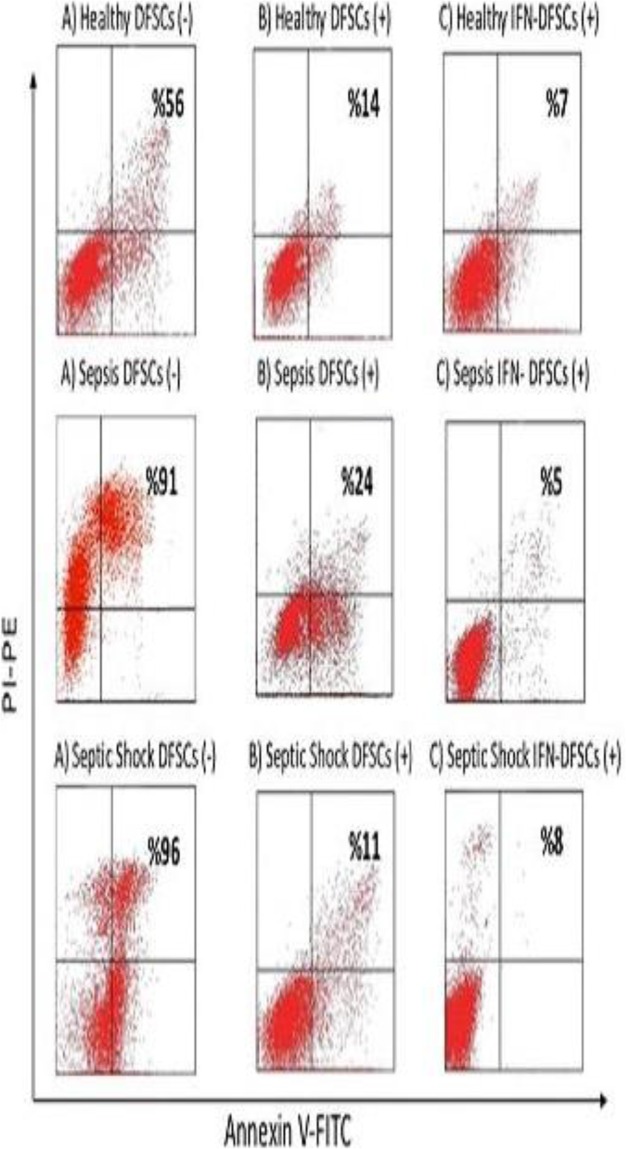
DFSCs and IFN-pre-stimulated DFSCs were significantly reduced CD4^+^ lymphocyte apoptosis in Sepsis and Septic Shock

### A912 Inhibition of mitochondrial complex i by metformin recapitulates the blunted response to exogenous mitochondrial uncoupling seen in sepsis

#### E. Greco, M.P. Lauretta, D.T. Andreis, M. Singer

##### Bloomsbury Institute of Intensive Care Medicine, University College London, London, United Kingdom

###### **Correspondence:** E. Greco – Bloomsbury Institute of Intensive Care Medicine, University College London, London, United Kingdom

**Introduction:** Inhibition of mitochondrial Complex I is described in human and animal sepsis.^1,2^ This may be responsible, at least in part, for the decrease in mitochondrial functionality seen in sepsis. We have recently demonstrated that the mitochondrial uncoupling agent, dinitrophenol (DNP) failed to increase body temperature and oxygen consumption (VO_2_) in septic rats, as was seen in healthy controls. This suggests that uncoupling is active in sepsis and can contribute to fever. We further postulated that the blunted effects of DNP in sepsis may be related in part to upstream mitochondrial inhibition.

**Objectives:** To determine if complex I inhibition by metformin in healthy rats can prevent the increment in temperature and oxygen consumption (VO_2_) by DNP, and thus mimic the pattern seen in sepsis.

**Methods:** VO_2_ was measured in awake, cannulated male Wistar rats (approx 300 g body weight) in metabolic cages (Oxymax, Columbus Instruments). Sepsis was induced with an intraperitoneal injection of faecal slurry at time 0. Sham control animals received no slurry. Fluid resuscitation (10 ml/kg/h crystalloid) was started at 2 hours and continued throughout the whole experiment. Half the septic and sham animals were treated with an IV infusion of metformin (186 mg/kg) between hours 2–6. At 6 and 24 hours, all animals received iv DNP (30 mg/kg). Arterial blood gases, echocardiography and core temperature were measured at times 0, 6 and 8, and 24 and 26 hours (i.e. before and after the two doses of DNP). Mean arterial pressure was recorded continuously. Wilcoxon Rank Sum test was used to compare groups and two-way ANOVA to compare changes in continuous variables from baseline between groups. p values < 0.05 were considered statistically significant.

**Fig. 43 (abstract A912).**
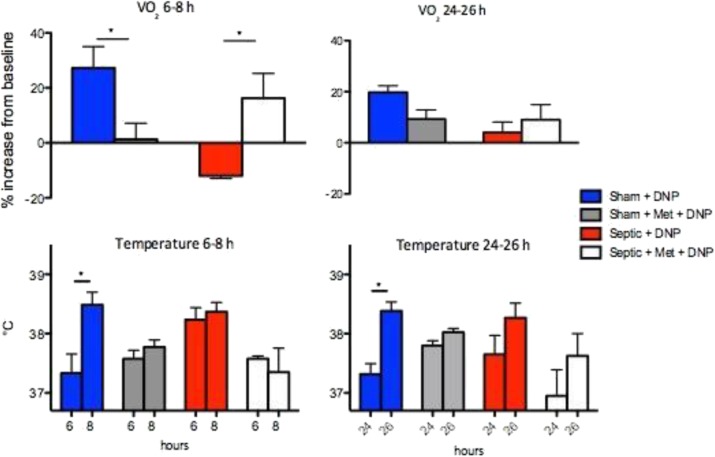
Met = Metformin, VO2 = oxygen consumption

**Results:** Pretreatment with metformin completely prevented the increase in temperature and VO_2_ induced by DNP in sham animals at 6 hours and reflected that seen in non-metformin treated septic rats (Figure 43). The reduction in myocardial contractility (stroke volume and Vmax) seen in the septic animals treated with DNP was prevented by Complex I inhibition at 24 h. Metformin was metabolically well tolerated, with no increase in blood lactate.

**Conclusions:** Inhibiting complex I with metformin prevents the uncoupling effect of DNP in sham animals. This mimics the pattern seen in septic animals and confirms that both Complex I inhibition and pre-existing mitochondrial uncoupling could be active in septic rats.

**References**

1. Brealey D, et al. Lancet. 2002;360:219–23, ^2^Brealey D, et al. Am J Physiol Regul Integr Comp Physiol. 2004;286:R491-7

### A913 Behavior and prognostic value of nlrp3 inflammasome in sepsis and septic shock

#### I. Palacios Garcia, M. Cordero, A. Diaz Martin, T. Aldabó Pallás, J. Garnacho Montero, J. Revuelto Rey, L. Roman Malo

##### Hospital Virgen del Rocio, Sevilla, Spain

###### Correspondence: I. Palacios Garcia – Hospital Virgen del Rocio, Sevilla, Spain

**Objectives:** The inflammasome is a multiprotein complex that stimulates cytokines release such as interleukin-1β (IL-1β) and IL-18, involved in the inflammatory response. Our aim is to quantify the state of activation of the inflammasome complex in septic patients, as well as to study possible differences in the cytokines levels in sepsis and septic shock, its temporary evolution, and its prognostic value.

**Methods:** Prospective study including patients admitted to the ICU with sepsis or septic shock during 15 months. On days 1, 3 and 7, IL 1-β serum levels and real-time expression of NLRP3 inflammasome (nucleotide- binding oligomerization domain, leucine rich repeat domain containing protein and Pyrin) were determined by Elisa and Real time-PCR respectively. Demographic variables, severity scores on ICU admission (APACHE II and SOFA), sepsis focus and mortality were collected. Statistical analysis: T-Student, Kruskal-Wallis and U-Mann-Whitney test as appropriate.

**Results:** There were included 31 patients (severe sepsis 14 and septic shock 18). Overall mortality was 29 % (9 patients). The levels of IL-1β on day 1 (16.5 ± 2.6 vs 14.1 ± 2.4 pg/mL; p < 0.05) and NLRP3-inflammasome (8.5 ± 1.3 vs 7.5 ± 1 mRNA arbitrary units; p < 0.05) were significantly higher in septic shock patients than in sepsis, with no differences in the following days set (3 and 7). The IL-1β and NLRP3 inflammasome levels decreased significantly on days 3 and 7 compared to first day (p < 0.001), without differences between survivors and deceased patients.

**Conclusions:** In septic patients, inflammasome activation complex occurs, with higher levels detected in septic shock. Decreased levels of IL-1β and NLRP3 inflammasome in septic process have been observed during evolution, actually without relation with mortality.

## Weaning And Noninvasive Ventilation

### A914 Diaphragm thickening fraction multiplied by rapid shallow breathing index could be the best parameter for extubation

#### A.A. Tanaka Montoya^1^, A.D.C. Amador Martinez^2^, L.Y. Delgado Ayala^2^, E. Monares Zepeda^2^, J. Franco Granillo^2^, J. Aguirre Sanchez^2^, G. Camarena Alejo^2^, A. Rugerio Cabrera^2^, A. Pedraza Montenegro^2^

##### ^1^ABC Medical Center, Critical Care Unit, Mexico, Mexico; ^2^ABC Medical Center, Mexico, Mexico

###### **Correspondence:** A.A. Tanaka Montoya – ABC Medical Center, Critical Care Unit, Mexico, Mexico

**Introduction:** Measuring diaphragm thickening fraction (DTF) multiplied by Rapid shallow breathing index (RSBI), it can be used as a measure to predict the success or failure extubation.

**Objectives:** the usefulness of a new formula DTF*RSBI as a parameter to withdrawal mechanical ventilation.

**Methods:** 65 patients with invasive mechanical ventilation were recruited prospectively at the Department of Critical Care Medicine Medical Center ABC in a period of 9 months on August 2015 April 2016. The DTF was measured in the area of apposition of the diaphragm to the chest, using an ultrasound transducer 4 MHz and was performed by physician radiologist. It proved difficult to visualize left hemidiaphragm, the reason RSBI*DTF were not performed on left side. Enrolled patients underwent with following weaning criteria: patient awake without continuous infusion of sedatives, SpO2 ≥ 90 % with FiO2 ≤ 50 % and PEEP ≤ 5 cmH2O, and no need for vasopressors. The percentage change in DTF (the end of inspiration-end expiration between the end of expiration) in pressure support (PS) ventilation mode with ventilatory progression purposes extubation was calculated. PS around 7 cm H2O without PEEP. A successful extubation was defined as spontaneous breathing > 48 hrs without ventilatory support.

**Results:** We included data obtained from 65 patients, 23(35.4 %) women and 42 (64.6 %) men, mean Body Mass Index (BMI) of 25.83 (SD ± 4.19), The frequency of extubation failure was 21.5 %, and mortality 24.6 %. RSBI with a mean of 57.0 ± 12.3 points. USG measurements were: Right thickness at end expiration (RTEEx) 0.28 ± .05 cm, Right Thickness at end inspiration (RTEIs) 0.21 ± .05 cm, Right Diaphragm Thickening fraction (RDTF) 23.1 ± 10.7 %. Mechanical ventilation time in median of 4 days (IQR 3–6) vs 8.5 (IQR 7 to 11), p < 0.001 and RSBI 68.2 +/− 9.6 vs 53.9 +/− 11.1, p < 0.001. Variables able to discriminate failures and their predictive yield were as follows: RSBI with ROC = 0.82 (CI 95 % 0.70-0.94) with a cutoff point of > =68, sensibility 0.64, specificity 0.84 Positive Likelihood ratio (LR+) =4.09 Negative Likelihood Ratio (LR-) =0.4; DTF with ROC = 0.85 (CI 95 % 0.71-0.99) cutoff of > =31, sensibility 0.71, specificity 0.92 LR+ =9.1, LR- =0.31; DTF*RSBI with ROC = 0.90 (CI95% 0.79 - 0.99) cutoff point of > =19.5, sensibility 0.85, specificity 0.92, LR+ =10.92, LR- =0.15. All comparisons with a p < 0.05.

**Conclusions:** There is no relationship between COPD patients, BMI, age and extubation failure. This new formula combine 2 parameters, DTF*RSBI, as a good parameter for extubation.

**References**

1. Giovanni Ferrari et al.Diaphragm ultrasound as a new index of discontinuation from mechanical ventilation critical ultrasound journal 2014

2. Gayan-Ramirez G.Ventilator-induced diaphragm dysfunction: time for (contr)action! Eur Respir J. 2013;42:12–5

3. Schepens T. et al.The course of diaphragm atrophy in ventilated patients assessed with ultrasound: a longitudinal cohort study Critical Care (2015) 19:422

### A915 Patients extubated without any spontaneous breathing trial. A sub-analysis of the wind study

#### T. Pham^1,2,3^, G. Beduneau^4,5^, F. Schortgen^6^, L. Piquilloud^7^, E. Zogheib^8,9^, M. Jonas^10^, F. Grelon^11^, I. Runge^12^, N. Terzi^13,14^, S. Grangé^4^, G. Barberet^15^, P.-G. Guitard^16^, J.-P. Frat^17,18^, A. Constan^19^, J.-M. Chrétien^20^, J. Mancebo^21^, A. Mercat^22^, J.-C.M. Richard^23^, L. Brochard^3^, The WIND study group

##### ^1^Hôpital Tenon, APHP, Medical and Surgical ICU, Paris, France; ^2^Université Paris Diderot, Sorbonne Paris Cité, UMR 1153, Paris, France; ^3^University of Toronto Saint Michael's Hospital and Keenan Research Centre, Interdepartmental Division of Critical Care, Toronto, Canada; ^4^Rouen University Hospital, Medical Intensive Care, Rouen, France; ^5^Rouen University Hospital, UPRES EA3830 IRIB, Rouen, France; ^6^CHU Henri Mondor, Medical ICU, Créteil, France; ^7^University Hospital of Lausanne, Intensive Care and Burn Unit, Lausanne, Switzerland; ^8^CHU d'Amiens, Cardiothoracic and Vascular ICU, Amiens, France; ^9^Université Jules Verne, Picardie, INSERM U1088 - CURS, Amiens, France; ^10^CHU Hotel Dieu, Medical ICU, Nantes, France; ^11^Hospital Le Mans, Intensive Care Unit, Le Mans, France; ^12^CHR d'Orleans, Medical ICU, Orleans, France; ^13^CHU Grenoble Alpes, Medical ICU, Grenoble, France; ^14^Université Grenoble-Alpes, Inserm U1042, Grenoble, France, ^15^CHR de Mulhouse, Medical ICU, Mulhouse, France; ^16^Rouen University Hospital, Surgical Intensive Care, Rouen, France; ^17^CHU de Poitiers, Medical ICU, Poitiers, France; ^18^Université de Poitiers, INSERM, CIC- 1402, Équipe 5 ALIVE, Poitiers, France; ^19^CHU Henri Mondor and ReVA Network, Medical ICU, Créteil, France; ^20^CHU d'Angers, Clinical Research Institute, Angers, France; ^21^Hospital de Sant Pau, Servei de Medicina Intensiva, Barcelona, Spain; ^22^CHU d'Angers, Medical ICU, Angers, France; ^23^Annecy Genevois General Hospital, Annecy, France

###### **Correspondence:** G. Beduneau – Rouen University Hospital, Medical Intensive Care, Rouen, France

**Introduction:** According the consensus conference on weaning from mechanical ventilation, intubated patients should pass a spontaneous breathing trial (SBT) to assess their readiness to be extubated.

**Objectives:** To characterize patients who are extubated without any SBT and to compare them to patients who had at least 1 SBT during their weaning period.

**Methods:** The prospective multicentre observational WIND (Weaning accordIng New Definition) study was performed from April to August 2013. Ventilation and weaning modalities were daily assessed until discharge in all intubated patients admitted to the participating ICUs. We defined 1) weaning attempt (WA) as a spontaneous breathing trial (SBT) or an extubation (with or without SBT), 2) successful weaning as an extubation without death or invasive mechanical ventilation within 7 days. Variables are presented as mean ± standard deviation, median [interquartile range] or number (percentage). Comparisons were made using Chi2 test, exact Fisher tests, Student t-test or Wilcoxon rank sum test as appropriate. All statistical tests were two-sided and P value ≤ 0.05 were considered significant.

**Results:** Among the 2729 patients included, 2051 patients had at least 1 WA comprising 1669 patients whose first WA was a SBT and 382 who had another type of first WA. These 382 patients with no SBT had a total of 454 WA: 252 (55.6 %) planned extubation without SBT, 124 self-extubations (27.3 %), 30 WA while tracheostomized (6.6 %) and 48 SBT after their first WA (10.6 %). The majority of patients with self-extubation had a successful weaning not requiring reintubation (75.8 %). Almost a quarter (n = 95) of the patients who were extubated without any SBT had a decision of withholding or withdrawing invasive mechanical ventilation, representing 89.7 % (N = 78) of the 87 deceased patients.

We then excluded patients with a decision of limitation and patients with a self extubation to compare patients who had a planned extubation with or without SBT as first WA (Table 22).

Patients with no SBT were younger, less severe and were more often admitted for unplanned surgery: they had an easier weaning with a lower (but non significative) rate of reintubation, a shorter duration of invasive mechanical ventilation and a shorter length of stay in the ICU.

**Conclusion:** Patients who are extubated without SBT seem to belong to three different groups: self-extubation, terminal extubation and patients in whom physicians anticipate an uneventful weaning and extubation. Among the patients with a planned extubation and without any limitation decision, clinical judgment regarding weanability appears to be effective as this group of patients had a good outcome with a low reintubation rate.

**Grant acknowledgment**

This study benefited of a grant of the non-profit Association Départementale des Insuffisants Respiratoires (ADIR) of the Haute Normandie, France

**Table 22 (abstract A915).** Mean ± SD, median[IQR]

	Patient with no limitation and no self extubation N = 1666	Patients with planned extubation without SBT N = 177	Patients with planned extubation after a SBT N = 1489	p-value
Age, y	60 ± 16	53 ± 16	61 ± 16	<0.001
SAPS II at admission, points	45 ± 17	41 ± 16	46 ± 17	<0.001
SOFA at admission, points	6.7 ± 3.5	5.9 ± 3.2	6.7 ± 3.5	<0.001
Admission: Medical/Planned/Unplanned surgery, n (%)	1163 (69.8)/247 (14.8)/256 (15.4)	107 (60.5)/27 (15.3)/43 (24.3)	1056 (70.9)/220 (14.8)/213 (9.9)	0.004/0.86/<0.001
Reintubations, n(%)	159 (9.5)	11 (6.3)	148 (11.7)	0.11
Total number of days of invasive MV, days	4 [2;8]	2 [1;6]	4 [2;9]	<0.001
Delay from intubation to 1st WA, days	3 [2;6]	2 [1;5]	3 [2;6]	<0.001
Length of stay in the ICU, days	7 [4;13]	4 [3;12]	7 [4;13]	<0.001
Death, n (%)	46 (2.8)	5 (2.8)	41 (2.8)	0.85

### A916 Maximal relaxation rate (MRR) of the diaphragm: could diaphragmatic sonograpy be a reliable alternative to transdiaphragmatic pressure (PDI) measurements?

#### E. Soilemezi, E. Koco, S. Savvidou, C. Nouris, D. Matamis, Plug Working Group

##### 'Papageorgiou' General Hospital, ICU, Thessaloniki, Greece

###### **Correspondence:** E. Soilemezi – 'Papageorgiou' General Hospital, ICU, Thessaloniki, Greece

**Introduction:** Decrease in diaphragmatic maximal relaxation rate (MRR) occurs early in the process of diaphragmatic fatigue and well before the diaphragm fails as a force generator; its measurement would, therefore, be especially valuable in ICU patients during a weaning trial. However, the use of oesophageal pressure catheters for that purpose impedes wide clinical use. On the contrary, M-mode sonography, allows non-invasive, real-time measurement of the speed of the diaphragmatic motion.

**Objective:** Purpose of our study was to investigate a possible correlation between diaphragmatic MRR traditionally acquired with transdiaphragmatic pressure (Pdi) catheters (MRR-Pdi) and an ECHO equivalent MRR (MRR-ECHO) acquired during different breathing conditions.

**Methods:** The slope of MRR was measured from the initial steepest part of the descending Pdi curve simultaneously with the slope of the initial steepest descending part of diaphragmatic excursion with M-mode sonography. The protocol entrained four consecutive stages: i) breathing spontaneously during T-piece trial, ii) breathing spontaneously with performance of sniff-like maneuvers, iii) breathing with resistances of 40cmH_2_O/L, and iv) breathing with resistances of 40cmH_2_O/L with performance of sniff-like maneuvers. Statistical comparisons between slope recordings from the two methods were performed with Pearson correlation, while Bland and Altman plots were obtained in order to demonstrate reliable agreement between methods at each different breathing condition.

**Results:** A total of 512 separate breaths during the four previously reported breathing conditions from six ICU patients were recorded. Table 23 summarizes the slopes measured from MRR-Pdi and MRR-ECHO as means ± standard deviations (SD), and their linear correlations with p values.

Statistical significant correlations were observed in all four stages; i) Pearson correlation coefficient r = 0.474, p < 0.001, R^2^ = 0.2247, ii) r = 0.834, p < 0.001, R^2^ = 0.6950, iii) r = 0.653, p < 0.001, R^2^ = 0.4269, and iv) r = 0.794, p < 0.001, R^2^ = 0.6302. Bland and Altman plots demonstrating differences of measurements against means, as well as confidence intervals (means of differences ± 2SD) were obtained for each breathing condition. Graph 1 represents the Bland and Altman plot for spontaneous breathing with sniff-like maneuvers without resistances.

High R^2^ indexes, indicating high agreement between the two methods were noted: i) 0.9915, ii) 0.9986, iii) 0.9941, and iv) 0.9990.

**Conclusions:** The results of our study suggest a statistical significant correlation and reliability between diaphragmatic MRR measured from Pdi tracings and the assumed diaphragmatic relaxation rate calculated from simultaneous M-mode sonographic recordings. Clinical studies are required to confirm the potential of this non-invasive index of diaphragmatic MRR to be used as a predictor for weaning success.

**Grant acknolwedgement**

None declared.

**Table 23 (abstract A916).** ᅟ

	Slope MRR-Pdi	Slope MRR-ECHO	Linear correlations	p values
	(Y axis, in cmH_2_O/sec)	(X axis, in cm/sec)		
i)Spontaneous breathing	77.06 ± 15.83	3.03 ± 0.97	Y = 45 + 9X	<0.0001
ii)Sniff-like maneuver	140.48 ± 48.15	3.77 ± 1.58	Y = 45 + 25X	<0.0001
iii)Resistances	167.49 ± 41.49	4.89 ± 2.21	Y = 110 + 12X	<0.0001
iv)Sniff with resistances	221.64 ± 60.29	5.67 ± 1.62	Y = 53 + 30X	<0.0001

**Fig. 44 (abstract A916).**
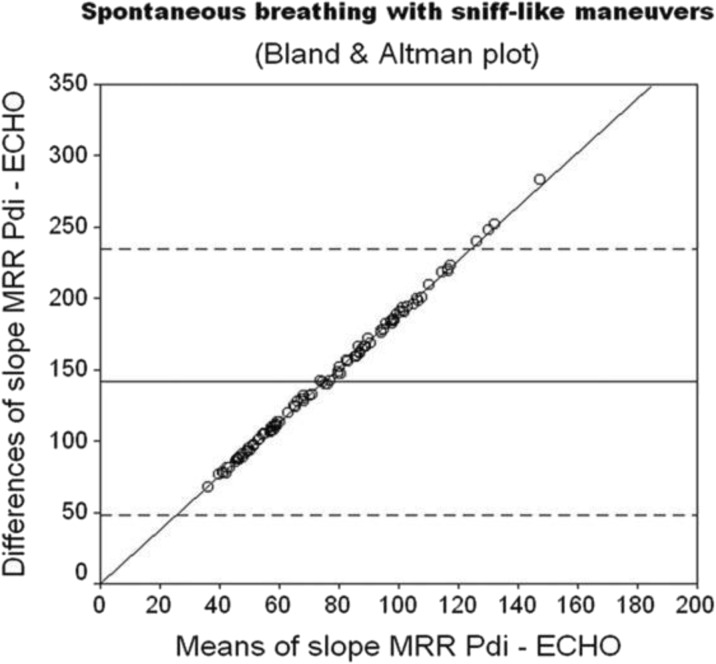
Spontaneous breathing with sniff-like maneuvers

### A917 Effects of high flow nasal cannula oxygen on diaphragmatic electrical activity in the post extubation period

#### R. Di Mussi^1^, S. Spadaro^2^, C.A. Volta^2^, M. Mariani^1^, A. Colaprico^1^, C. Antonio^1^, F. Bruno^1^, S. Grasso^1^

##### ^1^Dipartimento dell'Emergenza e dei Trapianti d'Organo, Università degli Studi “Aldo Moro”, Bari, Italy; ^2^Ferrara University, Ferrara, Italy

###### **Correspondence:** R. Di Mussi – Dipartimento dell'Emergenza e dei Trapianti d'Organo, Università degli Studi “Aldo Moro”, Bari, Italy

**Introduction:** High flow nasal cannula oxygen therapy (HF OXY) has been recently shown to decrease re-intubation rate, as compared with low flow oxygen therapy (LF OXY).[1], [2]

**Objectives:** To assess the effects of HF OXY as compared with LF OXY on diaphragmatic electrical activity (Eadi), respiratory rate (RR), tidal volume (VT) and gas exchange in the post extubation period. Our hypothesis was that HF OXY, as compared with LF OXY, would improve gas exchange and decrease EAdi.

**Methods:** 10 patients underwent a crossover study immediately after extubation. Each patient was submitted to three consecutive steps of 1 hour each, according to an ON-OFF design: 1) HF OXY; 2) LF OXY; 3) HF OXY. Oxygen fraction was maintained stable throughout the study. The Eadi was continuously monitored through Eadi cathether (Maquet, Solna Sweden). The heated and humidified HF OXY was delivered through nasal cannula at flow rates of 50–70 L/min, (F&P, Auckland New Zealand).

**Fig. 45 (abstract A917).**
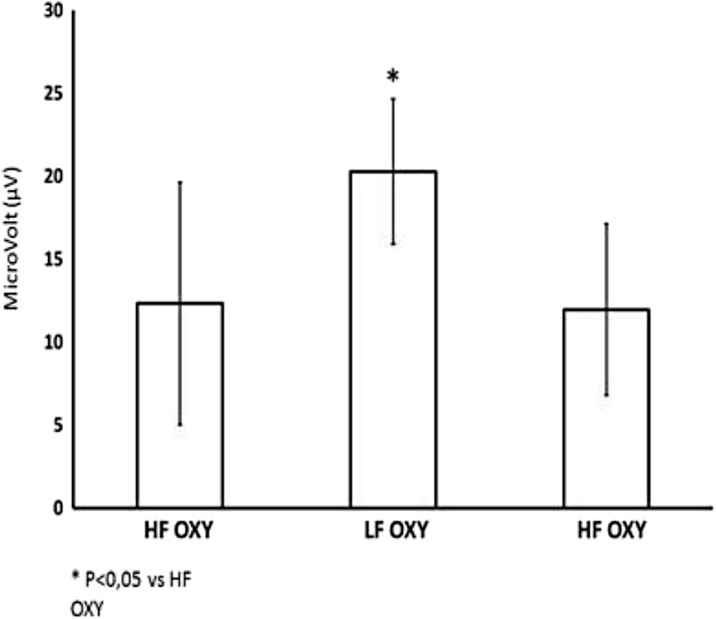
Diaphragmatic activity: LF OXY vs HF oxy

**Results:** RR remained similar throughout the study, VT was significantly higher during the LF OXY step as compared with the HF OXY steps. Oxygenation significantly improved during the HF period, whereas PaCO_2_ remained unchanged throughout the study (Table 24). EAdi was significantly higher during LF OXY (Figure 45)

**Conclusions:** Since the EAdi is correlated to work of breathing, our physiological data suggest that HF OXY significantly reduces WOB while improving oxygenation in the post extubation period. Further studies are required to define if diaphragm unloading may explain the favourable results of HF OXY in clinical trials.

**References**

1. Hernández G, Vaquero C, González P, Subira C, Frutos-Vivar F, Rialp G, Laborda C, Colinas L, Cuena R,Fernández R. - Effect of Postextubation High-Flow Nasal Cannula vs Conventional Oxygen Therapyon Reintubation in Low-Risk Patients: A Randomized Clinical Trial.- JAMA 2016 Apr 5;315(13):1354–61.

2. Maggiore SM, Idone FA, Vaschetto R, Festa R, Cataldo A, Antonicelli F, Montini L, De Gaetano A, Navalesi P, Antonelli M. - Nasal high-flow versus Venturi mask oxygen therapy after extubation. Effects on oxygenation, comfort, and clinical outcome - Am J Respir Crit Care Med. 2014 Aug 1;190(3):282–8

**Table 24 (abstract A917).** Breathing pattern parameters

	LF OXY	HF OXY	P value
RR (breaths/min.)	27.76 ± 2.32	26.34 ± 1.84	0.1
VT (L)	0.47 ± 0.12	0.36 ± 0.09	0.04
PaCO2 (mmHg)	49.24 ± 2.82	47.84 ± 1.64	0.09

Data are expressed as mean ± standard deviation (SD)

### A918 Non-invasive mechanical ventilation in acute respiratory failure in critically ill patients with confirmed influenza infection: a chaid decision-tree analysis

#### A. Rodriguez^1,2^, I. Martín-Loeches^3^, E. Díaz^4^, J.R. Masclans^5^, F. Gordo^6^, J. Solé-Violán^7^, M. Bodí^1,2^, F.X. Avilés-Jurado^2,8^, S. Trefler^1,2^, M. Magret^1,2^, L.F. Reyes^9^, J. Marín-Corral^5^, J.C. Yebenes^10^, A. Esteban^11^, A. Anzueto^9^, S. Aliberti^12^, M.I. Restrepo^9^, GETGAG/SEMICYUC

##### ^1^Hospital Universitari Joan XXIII, Critical Care Medicine, Tarragona, Spain; ^2^Universitat Rovira i Virgili/IISPV, Tarragona, Spain; ^3^St James's University Hospital. Trinity Center for Health Sciences, Anaesthesia and Critical Care, Dublin, Ireland; ^4^Hospital Parc Tauli, Critical Care Medicine, Sabadell, Spain; ^5^Hospital del Mar/CIBERES/UPF, Critical Care Medicine, Barcelona, Spain; ^6^Hospital del Henares, Critical Care Medicine, Madrid, Spain; ^7^Hospital Dr. Negrín, Critical Care Medicine, Las Palmas de Gran Canaria, Spain; ^8^Hospital Universitari Joan XXIII, ORL, Tarragona, Spain; ^9^UT Heatlh Science Center at San Antonio and South Texas Veterans Health Care System, Pulmonary Disease and Critical Care, San Antonio, United States; ^10^Hospital de Mataró, Critical Care Medicine, Mataró, Spain; ^11^Hospital de Getafe, Critical Care Medicine, Madrid, Spain; ^12^University of Milan - Biocca San Genaro Hospital, School of Medicine and Surgery, Monza, Italy

###### **Correspondence:** A. Rodriguez – Hospital Universitari Joan XXIII, Critical Care Medicine, Tarragona, Spain

**Introduction:** I Non-invasive mechanical ventilation (NIV) has been seen to play a major role in decreasing intubation rates in patients with severe exacerbation of chronic obstructive pulmonary disease and congestive heart failure. Unsuccessful NIV has been found to be independently associated with increased mortality in patients with ARF. The NIV failure and their impact on mortality in patients with inlfuenza infection is unknown.

**Objectives:**

1. to describe non-invasive ventilation failure (NIVf) rate,
2. to identify risk factor for NIVf using CHAID (Chi-square Automatic Interaction Detection) and
3. to determine if NIVf is associated with ICU-mortality.

**Methods:** Secondary analysis in 1,898 patients with influenza requiring mechanical ventilation(MV). Three groups were considered:

1. patients with NIV who failed (Group A);
2. patients with NIV who succeeded (Group B); and
3. patients with invasive MV (Group C). Cox analysis was used to assess survival. Risk factors for NIVf were obtained using CHAID.

**Results:** From a total study population of 1898, 806 patients underwent NIV and 1092 received IMV (Group C). Among NIV patients, 56.8 % (Group A) failed and had higher (p < 0.01) APACHE II (17 vs. 14), SOFA (7 vs. 4), infiltrates in chest x-ray (CXR = 3 vs. 2) and ICU-mortality (38.4 % vs. 6.3 %) than Group B. SOFA(S) was the variable most associated with NIVf by CHAID and 2 cut-offs were determined. Patients with S > 5 had more risk of NIVf (OR = 3.3,95%CI:2.4-4.5). Among patients with S < 5, 40.3 % failed and chronic obstructive pulmonary disease (COPD) was associated with low risk of NIVf (OR = 0.59,95%CI:0.41-0.83). NIVf was independently associated with ICU-mortality (OR = 11.4,95%CI:6.5-20.1). ICU-mortality was higher for Group A (38.4 %) than Group C (31.3 %, p < 0.01). A trend of high adjusted ICU-mortality was observed for Group A patients versus Group C (HR = 1.19;95%CI:0.99-1.44;p = 0.07).

**Conclusions:** NIV failure is frequent and independently associated with ICU-mortality in patients with influenza. CHAID analysis might be a promising tool to assist in clinical decision-making.

**References**

1. Masclans JR, et al. Early non-invasive ventilation treatment for severe influenza pneumonia. Clin Microbiol Infect 2013;19: 249–2562.

2. Nicolini A, et al. Effectiveness and predictors of success of noninvasive ventilation during H1M1 pandemics: a multicenter study. Minerva Anestesiol 2012; 78: 1333–40

3. Nin N, et al. Clinical characteristics and outcome of patients with 2009 influenza A(H1N1) virus infection with respiratory failure requiring mechanical ventilation. J Crit Care 2011; 26: 186–192

**Grant acknowledgment**

GETGAG/SEMICYUC

## AKI: OUTCOMES AND IMPROVING MANAGEMENT

### A919 Renal perfusion, function and oxygenation in the early postoperative period after liver transplantation

#### J. Skytte Larsson^1^, B. Redfors^2^, S.-E. Ricksten^1^

##### ^1^University of Gothenburg, Department of Anesthesia and Intensive Care, Sahlgrenska University Hospital, Göteborg, Sweden; ^2^University of Gothenburg, Department of Cardiothoracic Anesthesia and Intensive Care, Sahlgrenska University Hospital, Göteborg, Sweden

###### **Correspondence:** J. Skytte Larsson – University of Gothenburg, Department of Anesthesia and Intensive Care, Sahlgrenska University Hospital, Göteborg, Sweden

**Introduction:** Acute kidney injury (AKI) after liver transplantation is a common complication with an incidence of approximately 50 % [1], resulting in high morbidity and mortality. To increase the possibilities to prevent or treat AKI after liver transplantation, it is essential to increase the knowledge on changes in renal physiology after liver transplantation.

**Objectives:** The aim of this study was to gain insights into renal perfusion, filtration and oxygenation in the immediate postoperative period in patients undergoing liver transplantation and to compare these data to those obtained from a group of patients undergoing major surgery with no postoperative renal impairment.

**Methods:** Informed consent was obtained preoperatively from twelve patients with normal renal function accepted for liver transplantation. Glomerular filtration rate (GFR) was measured preoperatively by plasma clearance of Cr-EDTA. The patients were studied after liver transplantation in the ICU in the immediate postoperative period, sedated and mechanically ventilated. Systemic haemodynamics and renal variables where obtained during two 30-min periods. Renal blood flow (RBF) and GFR were measured by the renal vein retrograde thermodilution technique and by renal extraction of Cr-EDTA (=filtration fraction, FF), respectively. Arterial (a) and renal vein (rv) blood samples were taken for measurements of arterial (CaO_2_) and renal vein (CrvO_2_) oxygen contents. Renal oxygen consumption [RVO_2_ = RBF x (CaO_2_-CrvO_2_)], renal oxygen delivery (RDO_2_ = RBF x CaO_2_) and renal oxygen extraction [RO_2_Ex = (CaO_2_-CrvO_2_)/CaO_2_)] were calculated. Sixty-three patients undergoing uneventful cardiac surgery with no postoperative renal impairment served as controls.

**Results:** Cardiac index (65 %) and systemic oxygen delivery index (62 %) were higher and systemic vascular resistance index was lower (−38 %) in the liver transplant group compared to controls (p < 0.001). RBF was 17 % higher and renal vascular resistance was 16 % lower compared to controls (p < 0.05). In the liver transplanted group, GFR was 35 % lower compared to the preoperative value (p = 0.016), accompanied by a 41 % increase in serum creatinine (p < 0.05). After surgery, when compared to controls, GFR and FF was 23 % and 40 % lower, respectively (p < 0.05, p < 0.01), and RVO_2_ and RO_2_Ex were 42 % and 24 % higher, respectively, in the liver transplanted patients (p < 0.01, p < 0.05).

**Conclusions:** Despite the hyperdynamic systemic circulation, GFR is considerably reduced immediately after liver transplantation, most likely caused by a post-glomerular renal vasodilation decreasing upstream glomerular filtration pressure. Renal oxygenation is impaired after liver transplantation due to the high RVO_2_, which was not met by a proportional increase in RDO_2_.

**References**

1. Hilmi IA et al. Acute kidney injury following orthotopic liver transplantation: incidence, risk factors, and effects on patient and graft outcomes. *Brit J Anaesth* 2015, 114:919.

### A920 Association between aki stages and long-term mortality

#### R. Haines, J. Powell-Tuck, H. Leonard, M. Ostermann

##### Guy’s & St Thomas Hospital, London, United Kingdom

###### **Correspondence:** M. Ostermann – Guy's & St Thomas Hospital, London, United Kingdom

**Introduction:** Acute kidney injury is common in critically ill patients and associated with increased short and long-term mortality. Most published studies have focussed on patients with severe AKI. Little is known about the long-term outcome of patients with less severe AKI.

**Objectives:**

Our objective was to determine the outcome of patients with different stages of AKI at 5 and 7 years after admission to the Intensive Care Unit (ICU).

**Methods:** We retrospectively analysed the data of all adult patients admitted to a multi-disciplinary ICU in a teaching hospital in the UK between March 2004 - May 2009. Patients with chronic dialysis dependent renal failure were excluded. Patients were categorised according to their maximum stage of AKI during stay in ICU as defined by the serum creatinine criteria of the KDIGO classification. APACHE II and SOFA scores were used to describe severity of illness on admission to ICU. In patients with >1 admission to ICU, we only included the first admission in the analysis.

**Results:** Data of 3094 adult patients were analysed of whom 53 % had AKI during their stay in ICU.

Patients with any degree of AKI had a higher mortality at 5 and 7 years but they were also sicker on admission to ICU.

**Conclusions:** Any stage of AKI during critical illness is associated with an increased risk of mortality at 5 and 7 years. Mortality is highest in patients with AKI II and III. More work is necessary to explore the relationship between AKI and long-term outcome and to identify independent risk factors for mortality.

**Table 25 (abstract A920).** AKI stages and outcome

	No AKI	AKI I	AKI II	AKI III
Number of patients	1457 (47 %)	776 (25 %)	166 (5.4 %)	695 (22.5 %)
Age, mean (SD)	53 (18)	62 (17)	60 (17)	62 (16)
Male gender	59 %	68 %	61 %	64 %
APACHE II on admission to ICU, mean (SD)	12.9 (5.0)	16 (5.4)	16.6 (5.6)	19.8 (5.9)
SOFA score on admission to ICU, mean (SD)	3.5 (2.4)	5.3 (2.7)	6.1 (2.8)	7.5 (3.1)
1-year mortality	13.2 %	18.9 %	27.7 %	22.3 %
5-year mortality	27.3 %	34.3 %	42.2 %	40.1 %
7-year mortality	30.8 %	38.1 %	45.2 %	46.2 %

### A921 Forced fluid removal vs. usual care in intensive care patients with high-risk acute kidney injury and severe fluid overload (FFAKI) - a randomized clinical pilot trial

#### R.E. Berthelsen^1^, T.S. Itenov^1^, A. Perner^2^, J.U. Jensen^3^, M. Ibsen^1^, A.E.K. Jensen^4^, M.H. Bestle^1^

##### ^1^Nordsjællands Hospital, Dept. of Anaesthesiology and Intensive Care, Hillerød, Denmark; ^2^Rigshospitalet, Copenhagen University Hospital, Dept. of intensive Care, Copenhagen, Denmark; ^3^Rigshospitalet, Copenhagen University Hospital, CHIP & PERSIMUNE, Dept. of Infectious Diseases, Copenhagen, Denmark; ^4^University of Copenhagen, Dept. of Biostatistics, Copenhagen, Denmark

###### **Correspondence:** R.E. Berthelsen – Nordsjællands Hospital, Dept. of Anaesthesiology and Intensive Care, Hillerød, Denmark

**Introduction and objective:** Observational studies of intensive care unit (ICU) patients with acute kidney injury have shown a negative correlation between accumulation of fluids and survival [1]. It is unknown whether rapid removal of accumulated fluids is feasible and beneficial. Therefore we wish to perform a pilot trial of forced fluid removal vs. standard care in critically ill patients with high-risk acute kidney injury and severe fluid overload.

**Methods:** The FFAKI-trial is a pilot, multicenter, randomized clinical trial recruiting adult intensive care patients with high-risk acute kidney injury and fluid overload defined as > 10 % of ideal bodyweight. To reduce the signal-to-noise ratio we only wish to include patients with a high baseline risk of persistent renal failure. Baseline risk will be calculated using a newly developed model, the renal recovery score (RRS), to predict the chance of recovering renal function within 28 days. In- and exclusion criteria are shown in Tables 26 and 27.

**Fig. 46 (abstract A921).**
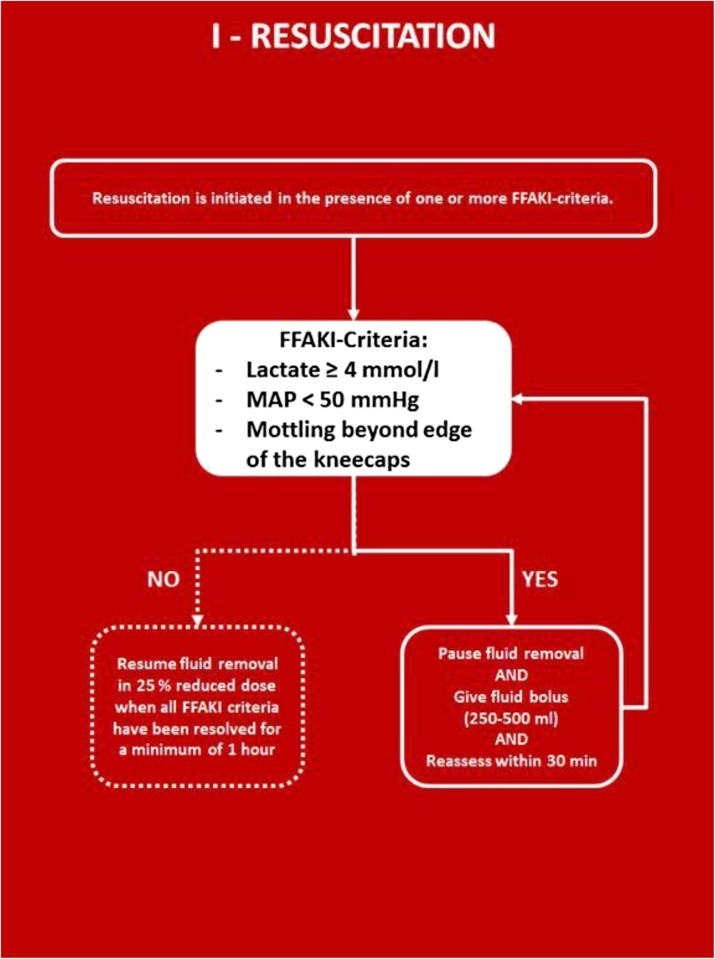
FFAKI algorithm - Rescucitation

**Fig. 47 (abstract A921).**
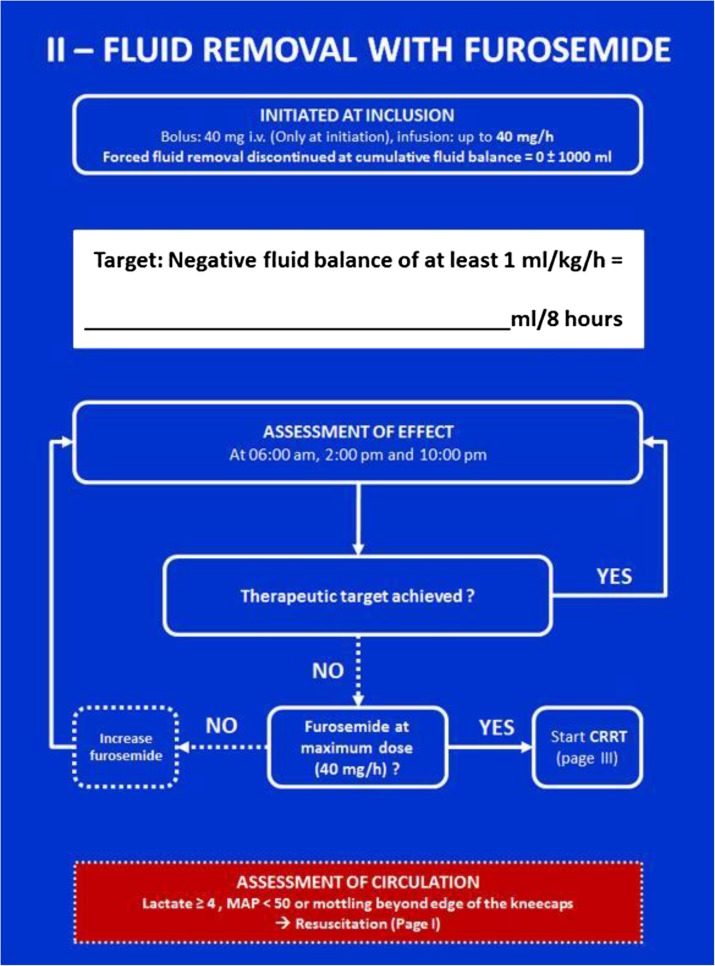
FFAKI algorithm - Furosemide

**Fig. 48 (abstract A921).**
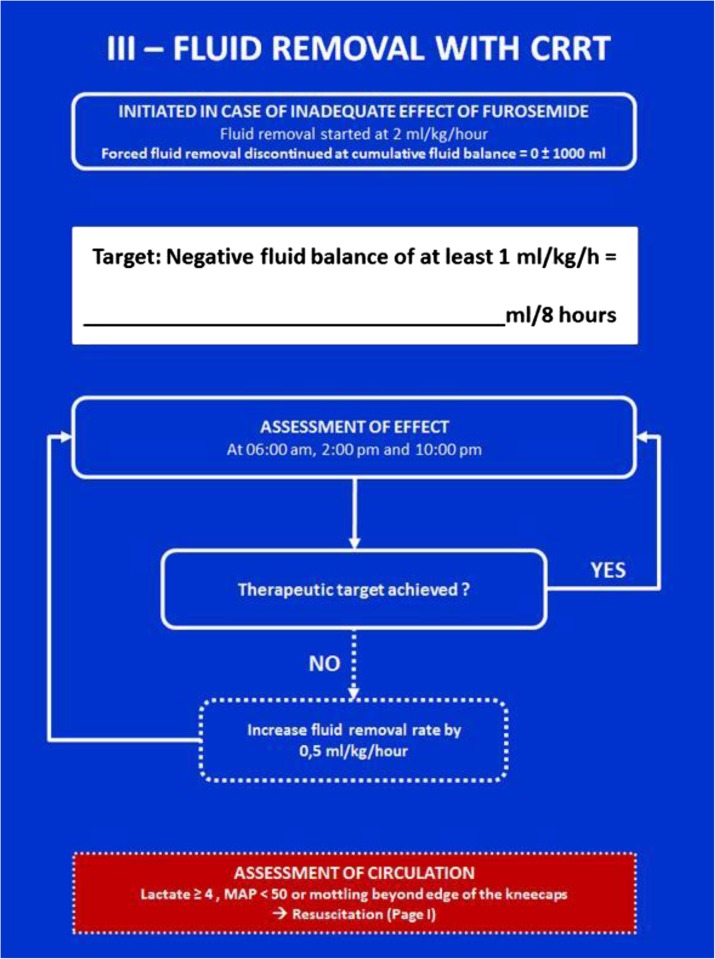
FFAKI algorithm - CRRT

Patients are randomized to either forced fluid removal or standard care for the entire ICU stay. Forced fluid removal is done by infusion of furosemide and/or fluid removal with continuous renal replacement therapy. The fluid removal rate is adjusted 3 times daily to achieve a therapeutic goal of net negative fluid balance ≥ 1 ml/kg/h. Physiologic tolerance to fluid removal is continually evaluated according to predefined criteria of hypoperfusion: Lactate ≥ 4 mmol/l, mean arterial pressure < 50 mmHg or mottling beyond the edge of the kneecaps. In case of hypoperfusion, fluid removal is suspended until all criteria have been resolved for a minimum of 1 hour. The flow chart for the experimental FFAKI-treatment is seen in Figure 46, 47, 48.

The primary outcome is cumulative fluid balance 5 days after randomization. By inclusion of 50 patients we are able to detect a difference of 5.9 L between groups (α = 0.05 and β = 0.80). Secondary outcomes include mean daily fluid balances, fluid balance at discharge from ICU, time to neutral fluid balance, number of serious adverse reactions and number of protocol violations. All patients are followed for 90 days.

**Conclusions:** The FFAKI trial started in October 2015 and will, when completed, provide data to evaluate if a larger trial of forced fluid removal in ICU patients is feasible. Our primary outcome will show if the experimental intervention leads to a clinically relevant difference in fluid balance which would warrant a definitive trial powered for survival at 90 days.

**Grant acknowledgment**

The FFAKI-trial is funded by the Research Department and the Dept. of Anaesthesiology at Nordsjællands Hospital, Denmark

**Trial registration.** EudraCT: 2015-001701-13, ClinicalTrials.gov: NCT02458157

**References**

[1] Zhang L, Chen Z et al. Associations of fluid overload with mortality and kidney recovery in patients with acute kidney injury: a systematic review and meta-analysis. J Crit Care 2015;30:860.e7-860.e13

**Table 26 (abstract A921).** Inclusion criteria

Age ≥ 18 years	
Acute Kidney Injury defined according to the KDIGO criteria.	
Renal Recovery Score ≤ 60 %.	
Fluid overload defined as a positive fluid balance ≥ 10 % of ideal body weight.	
Able to undergo randomization within 12 hours of fulfilling the other inclusion criteria

**Table 27 (abstract A921).** Exclusion criteria

Known allergy to furosemide or sulphonamides	
Known pre-hospitalization advanced chronic kidney disease. (eGFR < 30 mL/minute/1.73 m2 or chronic RRT)	
Severe hypoxic respiratory failure (use of invasive ventilation and FiO2 > 80 % and PEEP > 10 cm H2O)	
Severe burn injury (≥10 % TBSA)	
Severe dysnatremia (<120 or > 155 mmol/l)	
Hepatic coma	
Mentally disabled undergoing forced treatment.	
Pregnancy/breast feeding	
Lack of commitment for on-going life support including RRT	
Lack of informed consent

### A922 Sequential measurement of 1 hour creatinine clearance (1-CRCL) in critically ill patients at risk of acute kidney injury (AKI)

#### T. Bucknall^1^, J. Dixon^2^, F. Boa^3^, I. MacPhee^4^, B.J. Philips^5^, AKI Research Group, St George's University of London

##### ^1^St George's, University of London, London, United Kingdom; ^2^St Georges NHS Foundation Trust, General Intensive Care, London, United Kingdom; ^3^St Georges NHS Foundation Trust, Clinical Blood Sciences, London, United Kingdom; ^4^St George's, University of London, Renal Medicine, London, United Kingdom; ^5^St George's, University of London, General Intensive Care, London, United Kingdom

###### **Correspondence:** T. Bucknall – St George's, University of London, London, United Kingdom

**Introduction:** Measuring renal function in acute kidney injury (AKI) is difficult. The criteria on which a clinical diagnosis of AKI is made are broad and open to interpretation. More objective measures remain elusive. A truly robust biomarker has not been discovered and exogenous markers are difficult to use in critically ill patients. We have previously used measures of renal clearance of Iohexol (rICl) to evaluate AKI (data submitted) and have shown that 1-hour renal creatinine clearances (1-CrCl) correlate well with rICl (correlation r = 0.99; Bland Altman bias −5.6, SD 24.1 %) performing better than 4 hourly CrCl measurements (bias 11.4 % SD 56.7 %).

**Objective:** To use sequential 1-CrCl measurements to measure renal function in critically ill patients with or at risk of AKI over 72 hours and to compare this with traditional methods of assessing AKI. This was part of a BSc project.

**Methods:** Consent was obtained from the next of kin according to ethical review (NREC: 15/LO/1720). 1-hour urine samples were collected at 7 am and 7 pm. Serum creatinine (SCr) samples were taken 30 min after the urine collections were started. SCr and urine creatinine (UCr) concentrations were measured using the enzymatic method by the dedicated researcher. 1-CrCl was calculated by the equation: $CrCl=\left(UCr\times \mathrm{vol}\right)1.73/\left(\right(SCr$/min)xBSA). 1-CrCls were compared with the ´kidney disease improving global outcomes´ (KDIGO) criteria for AKI and estimated glomerular filtration rates (eGFR) calculated by the Cockcroft-Gault (CG), modification of diet in renal disease 7 (MDRD7) and chronic kidney disease epidemiology collaboration (CKI-EPI) methods. Kruskal Wallis, Spearman correlation and the Bland-Altman method were used (IBM® SPSS® version 22) in analysis.

**Fig. 49 (abstract A922).**
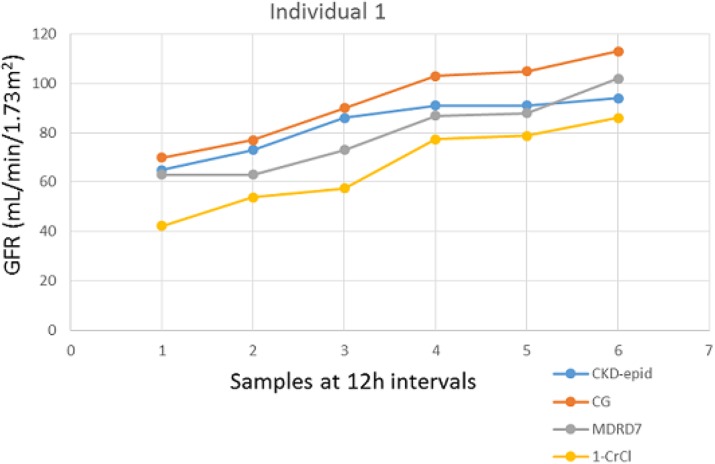
Individual example 1

**Fig. 50 (abstract A922).**
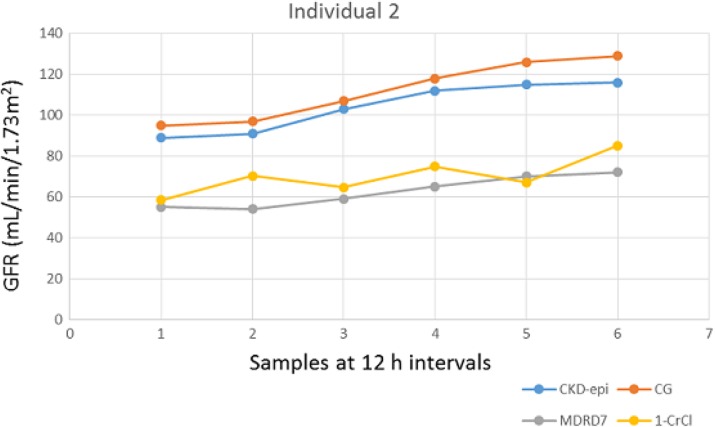
Individual example 2

**Fig. 51 (abstract A922).**
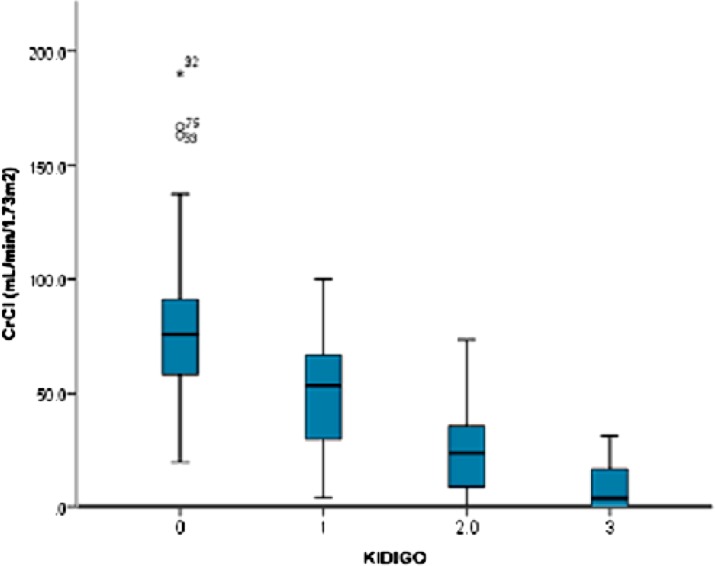
Boxplot of KDIGO and 1 hour CrCl

**Results:** 17 patients were included with a total of 87 1-CrCl measurements. Median (IQR) age was 67 (50–76) and admission APACHE II score 22 (22–32). 14 patients were medical admissions and 3 had emergency surgery. Observed overtime the 1-CrCl changed in similar patterns to the calculated eGFR (Figs. 49 and 50, examples) and correlations were good (1-CrCl vs CG r = 0.857, 1-CrCl vs CKD-epi = 0.861, 1-CrCl vs MDRD7 = 0.845). However the eGFR did not predict actual values. The Bland Altman comparisons for CKD-epi and MDRD7 are in Table 28. Fig 51 compares 1-CrCl with the KDIGO criteria

**Conclusions:** Despite good correlations, the eGFR calculated values were not good measures of actual renal function. 1-CrCl was often although not always markedly lower than the eGFR. The KDIGO criteria did reflect the measured 1-CrCl but there was marked overlap in all categories.

**References**

1. Endre ZH et al., *Nat Rev Nephrol*, 2014; 10: 683–385.

2. Bagshaw SM et al.*, Nephrol Dial Transplant* 2008, 23:1569–74

3. Kirwan CJ et al., *Critical care research and practice* 2013, 1–8.

4. Dixon J et al.*, J Transl Med*, 2015; 12:

**Table 28 (abstract A922).** Bland Altman comparison of 1-CrCl with eGFR

Comparison	Bias (%)	SD(%)	Limits of Agreement (%)
1CrCl vs CKD-epi	−6.5	21.3	−39.4 to 44.1
1CrCl vs MDRD7	2.4	20.5	−46.5 to 33.9

### A923 Does the implementation of a quality improvement care bundle reduce the incidence of acute kidney injury in those undergoing an emergency laparotomy

#### J. Doyle^1^, F. Saadat^1^, T. Samuels^1^, S. Huddart^2^, B. McCormick^3^, R. DeBrunnar^3^, J. Preece^3^, M. Swart^4^, C. Peden^5^, S. Richardson^6^, L. Forni^1^

##### ^1^Department of Intensive Care Medicine and Surrey Peri-Operative Anaesthesia and Critical Care Collaborative Research Group (SPACER), Guildford, United Kingdom; ^2^Department of Anaesthesia, Royal Surrey County Hospital NHS Foundation Trust, Guildford, United Kingdom; ^3^Royal Devon and Exeter Hospital, Exeter, United Kingdom; ^4^South Devon Healthcare NHS Foundation Trust, Torquay, United Kingdom; ^5^Department of Anaesthesia and Intensive Care, Royal United Hospital Bath NHS Trust, Bath, United Kingdom; ^6^Department of Surgery, Royal United Hospital Bath NHS Trust, Bath, United Kingdom

###### **Correspondence:** J. Doyle – Department of Intensive Care Medicine and Surrey Peri-Operative Anaesthesia and Critical Care Collaborative Research Group (SPACER), Guildford, United Kingdom

**Introduction:** Enhanced recovery pathways have been a focus for patient optimisation of morbidiy and mortality in the post-operative patient. Significant mortality improvement was seen following the implementation of the emergency laparotomy pathway quality improvement care (ELPQuiC) bundle with an adjusted risk of death from 15.6 % to 9.6 % (1). The first national emergency laparotomy audit (NELA) has since been published demonstrating a 30-day mortality of 11 % and recommending access to pathways that identify need to escalate care (2). However acute kidney injury (AKI) in critically unwell patients remains a major source of mortality, of up to 60 %, and morbidity (3). It is not yet clear whether enhanced recovery pathways, specifically those that utilise early goal directed therapy, affect the incidence of AKI.

**Objectives:** To determine if there was a difference in incidence of combined AKI pre and post implementation of an enhanced recovery protocol, one that had already demonstrated a significant mortality benefit.

**Methods:** A subgroup analysis of the data gathered via the ELPQuiC bundle was performed (1). We obtained buy-in from the 4 participating centres and requested an extrapolation of values from their raw data. If required further data was obtained via the hospital's electronic path system. All data was reviewed by a second investigator.

We defined the baseline creatinine as the best available preoperative creatinine from the past 1 year. The data recorded included creatinine at baseline, post-op, worse recorded creatinine between day1 and day30, MAKE 30, P-Possum and 30-day mortality data.

CKD stage was identified via MDRD equation with age, gender and baseline creatinine. Patients with AKI were stratified according to KIDGO stages of AKIN.

Primary outcome was the incidence of AKI in each of combined pre and post ELQUIC patient population. Secondary outcome included the stage specific incidence of AKI.

**Results:** There was no significant difference between the cumulative incidence of AKIN pre and post ELQUIC implementation on day 1 post-op (18.4 % vs 19.8 %, P = 0.6920) or day 30 post-op (14.3 % vs 8.6 %, P = 0.0647).

**Conclusion:** This multi-centre cohort subgroup analysis demonstrates that the implementation of a quality improvement care bundle does not affect the incidence of AKI. This is in contrast to the clear mortality benefit that such a care bundle has provided and provides stimulus to discover what factors may yet improve AKI, and so further improve these patients outcome.

**References**

1. Huddart S, Peden CJ, Swart M, et al. Use of a pathway quality improvement care bundle to reduce mortality after emergency laparotomy. Br J Surg. 2015;102(1):57

2. The First Patient Report of the National Emergency Laparotomy Audit - Oct 2015.

3. Chang CH, Fan PC, Chang MY, et al. Acute kidney injury enhances outcome prediction ability of sequential organ failure assessment score in critically ill patients. PLoS One. 2014;9(10):e109649

**Grants** - Nil received.

## QUALITY OF LIFE ISSUES IN THE ICU

### A924 Reduction of self-perceived discomforts in critically ill patients in french intensive care units: a cluster-randomized controlled trial

#### P. Kalfon^1^, K. Baumstarck^2^, P. Estagnasie^3^, M.-A. Geantot^4^, A. Berric^5^, G. Simon^6^, B. Floccard^7^, T. Signouret^8^, M. Boucekine^2^, M. Fromentin^9^, M. Nyunga^10^, A. Sossou^11^, M. Venot^12^, R. Robert^13^, A. Follin^14^, A. Renault^15^, M. Garrouste^16^, O. Collange^17^, Q. Levrat^18^, I. Villard^19^, D. Thévenin^20^, J. Pottecher^21^, R.-G. Patrigeon^22^, N. Revel^23^, C. Vigne^24^, O. Mimoz^25^, P. Auquier^2^, IPREA Study Group

##### ^1^CH Chartres, Réanimation, Chartres, France; ^2^Aix-Marseille Université, Unité de Recherche EA 3279, Marseille, France; ^3^Clinique Ambroise Paré, Réanimation, Neuilly/Seine, France; ^4^CHU Dijon Bourgogne, Anesthésie Réanimation, Dijon, France; ^5^CHI Toulon/La Seyne sur Mer, Réanimation Polyvalente, Toulon, France; ^6^CH Troyes, Réanimation, Troyes, France; ^7^CHU Edouard Herriot, Hospices Civils de Lyon, Réanimation Polyvalente, Lyon, France; ^8^Hôpital Européen de Marseille, Réanimation, Marseille, France; ^9^CHU Cochin, AP-HP, Réanimation Chirurgicale, Paris, France; ^10^CH Victor Provo, Réanimation Polyvalente, Roubaix, France; ^11^CH Emile Roux, Réanimation, Le Puy-en-Velay, France; ^12^CHU Saint-Louis, Réanimation Médicale, Paris, France; ^13^CHU La Milétrie, Réanimation Médicale, Poitiers, France; ^14^Hôpital Européen Georges Pompidou, AP-HP, Réanimation Chirugicale, Paris, France; ^15^CHU Brest, Réanimation Médicale, Brest, France; ^16^Groupe Hospitalier Paris Saint-Joseph, Médecine Intensive et Réanimation, Paris, France; ^17^CHU Strasbourg, Hôpital Civil, Réanimation Chirugicale Polyvalente, Strasbourg, France; ^18^Groupe Hospitalier de La Rochelle - Ré - Aunis, Réanimation, La Rochelle, France; ^19^CHU Beaujon, AP-HP, Anesthésie Réanimation, Clichy, France; ^20^CH Lens, Réanimation, Lens, France; ^21^CHU Strasbourg, Hôpital Hautepierre, Réanimation Chirugicale, Strasbourg, France; ^22^CH Auxerre, Réanimation, Auxerre, France; ^23^CHU Nice, Hôpital Pasteur, Réanimation Médico-Chirurgicale, Nice, France; ^24^CHU Hôpital Nord, AP-HM, Réanimation Chirugicale, Marseille, France; ^25^CHU La Milétrie, Réanimation Chirugicale, Poitiers, France

###### **Correspondence:** P. Kalfon – CH Chartres, Réanimation, Chartres, France

**Introduction:** It is now well documented that critically ill patients are exposed to stressful conditions and experience discomforts from multiple sources. Improved identification of the discomforts of patients in intensive care units (ICUs) may have implications for managing their care, including consideration of ethical issues, and may assist clinicians in choosing the most appropriate interventions.

**Objectives:** The primary objective of this study was to assess the effectiveness of a multicomponent program (MCP) of discomfort reduction in critically ill patients. The secondary objectives were to assess the sustainability of the impact of the program and the potential seasonality effect.

**Methods:** We conducted a multicenter, cluster-randomized, controlled, single (patient)-blind study involving 34 French adult ICUs. The experimental intervention was the implementation of the MCP including the following steps: identification of discomforts, immediate feedback to the healthcare team, and implementation of targeted interventions under control of local champions who received monthly feedback and organized monthly meetings with their healthcare team. All ICUs started with a 1-month period with no intervention, and then they were randomized to one of two groups: 17 ICUs with MCP implemented during a 6-month period (experimental group) and 17 ICUs without any programm during the same period (control group). To assess the sustainabilty of the impact of the MCP, the study was completed with a second 6-month period during which the MCP was no longer applied in the experimental group. The primary endpoint was the monthly overall score of self-reported discomfort from the French 16-item questionnaire on discomforts in ICU patients (IPREA)^1^(range from 0 to 100, the lowest possible level of discomfort to the highest). The secondary endpoints were the scores of each item of IPREA.

**Results:** At the end of the 6-month period, taking into account the clustering design, the monthly overall discomfort score was lower in the experimental group (median 16.7, IQR [14.6-18.7], N = 398] in comparison with the control group (median 23.7, IQR [21.6-25.7], N = 360]) ; D = −7.3, *p* < 0.001. This finding is confirmed after adjustment on age, gender, and IGS2: beta (95 % IC) = −6.6 (4.09;9.04), *p* < 0.001. For each item of IPREA, except pain, the score was lower in the experimental group in comparison with the control group.

**Conclusions:** The MCP of discomfort reduction decreased discomfort perceived by unselected ICU patients and may pave the way for a new strategy in the management of care for ICU patients.

**References**

1. Kalfon P, Mimoz O, Auquier P, Loundou A, Gauzit R, Lepape A, et al. Development and validation of a questionnaire for quantitative assessment of perceived discomforts in critically ill patients. Intensive Care Med. 2010;36(10):1751–8.

**Grant acknowledgment**

Supported by institutional grants from the French 2012 Programme Hospitalier Recherche Clinique National.

### A925 Evaluation of cognitive and emotional states during multidisciplinary clinical simulation sessions

#### S. Pawar^1^, T. Jacques^2^, K. Deshpande^2^, R. Pusapati^2^, B. Wood^2^

##### St George Hospital, Intensive Care, Sydney, Australia; ^2^St George Hospital, Sydney, Australia

###### **Correspondence:** S. Pawar – St George Hospital, Intensive Care, Sydney, Australia

**Introduction:** Simulation is increasingly used in healthcare to teach management of critical and life-threatening situations. The heightened emotions and cognitive overload caused by interaction with high-fidelity simulators may impact on participants' learning ability. We conducted this study to assess emotion during simulation and to explore its association with cognitive load.

**Design:** Prospective observational study.

**Setting.** Intensive Care Unit (ICU), St George Hospital, Sydney.

**Methods:** We enrolled medical and nursing staff with varying levels of ICU experience after obtaining informed consent. We used a standardised clinical scenario and the same human patient simulator for all simulation sessions. The actions of the participants determined the outcome of the scenario. We assessed the emotional state of each participant before and after the completion of the scenario using an eight-item scale containing bipolar oppositional descriptors of emotion.

1. We asked the participants to rate their emotion on a 5-point Likert scale with anchors at −2 and +2 representing the bipolar opposites. We assessed the cognitive load of each participant after the completion of the scenario using a validated subjective rating tool2. We asked the participants to rate their cognitive load on a scale of 1 to 9, with higher scores indicating higher mental effort.

**Results:** A total of 103 medical and nursing staff were studied. Compared to pre-simulation, the participants felt more relaxed (−0.28 ± 1.15 vs. 0.14 ± 1, p < 0.005; d = 0.39), excited (0.25 ± 0.89 vs. 0.55 ± 0.92, P < 0.005, d = 0.35) and alert (0.85 ± 0.87 vs. 1.28 ± 0.73, P < 0.00001, d = 0.54) following simulation. There was no difference in the mean scores for the remaining 5 items. The mean cognitive load for all participants was high (6.67 ± 1.41) and was not different in medical and nursing staff. There was no association between pre or post-simulation emotional state and cognitive load.

**Conclusion:** Simulation had a positive effect on the emotional state despite high cognitive load. Further studies are needed to determine their relationship with learning ability.

**References**

1. Fraser K, Huffman J. The emotional and cognitive impact of unexpected simulated patient death. CHEST 2014; 145(5):958–963.

2. Paas FGWC, Van Merriënboer JJG. The efficiency of instructional conditions: an approach to combine mental effort and performance measures. Hum Factors. 1993; 35 (4): 737–743.

### A926 Quality of life of children 12 months after emergency admission to intensive care

#### R.A. Pulham^1^, J. Wray^1^, K. Brown^1^, C. Pierce^2^, S. Nadel^3^, P. Ramnarayan^4^

##### ^1^Great Ormond Street Hospital, Critical Care and Cardiorespiratory Division, London, United Kingdom; ^2^Great Ormond Street Hospital, Paediatric Intensive Care Unit, London, United Kingdom; ^3^St Mary's Hospital and Charing Cross Hospital Imperial College NHS trust, Paediatric Intensive Care Unit, London, United Kingdom; ^4^Great Ormond Street Hospital, Children's Acute Transport Service, London, United Kingdom

###### **Correspondence:** R.A. Pulham – Great Ormond Street Hospital, Critical Care and Cardiorespiratory Division, London, United Kingdom

**Introduction:** Increasing numbers of children are now surviving after an intensive care admission but a proportion of these have significant morbidities. Although there is a growing body of evidence concerning the impact of intensive care stay on children and families, little is known specifically about the long-term status of children who are admitted to intensive care in an emergency.

**Objectives:** The aim of this study was to collect data from parents about their child's quality of life 12 months after an emergency intensive care admission.

**Methods:** Eligible children were those retrieved from district hospitals to 2 paediatric intensive care (PICU) units in the UK. Parents were asked to consent to being contacted 12 months after discharge, at which point they were asked to complete the PedsQL, a generic measure of quality of life. The PedsQL enables a total score, physical health summary score and psychosocial health summary score to be calculated, with possible scores ranging from 0–100 and higher scores equating to better quality of life.

**Results:** Parents of 60 children aged 1–16.5 years (median age: 2.1 years; 37 (62 %) males), the majority of whom had had an emergency PICU admission due to sepsis (n = 25, 42 %) or respiratory problems (n = 18; 30 %), completed the PedsQL 12 months after discharge from PICU. For the group overall the total score was 78.07 (SD 20.89), physical health summary score was 81.18 (SD 21.93) and psychosocial health summary score 77.60 (SD 21.63). Babies aged 13–23 months (n = 25) had total scores (m = 85.80, SD =12.39) comparable to those of healthy norms (m = 85.55, SD = 8.74). However older children in all age groups had lower total scores than healthy norms. Whilst 25 % (6/25) of babies had scores of more than one standard deviation below the score of healthy norms, which is recognised as being of clinical significance, this rose to 54 % (7/15) of children aged 5–18 years and 60 % (12/20) of children aged 2–4 years. Of note is that 6 children (30 %) aged 2–4 years had been admitted to PICU for reasons related to trauma or neurological concerns whereas no child aged 13–24 months had been admitted for those reasons.

**Conclusions:** Children who have had an emergency admission to PICU are at risk for impaired quality of life 12 months after discharge. The risk appears to be greater for children of 2 years and older which is likely to be at least partly attributable to the underlying reason for their admission. Evaluating quality of life outcomes in the longer term after PICU discharge is warranted and identification of potential risk factors will enable interventions to be targeted to optimise outcomes after an emergency admission to PICU.

### A927 Long-term cognitive outcomes in survivors of critical illness

#### J.R. Azevedo^1^, W.S. Montenegro^1^, D.P. Rodrigues^2^, S.C. Sousa^1^, V.F. Araujo^1^, A.L. Leitao^1^, P.H. Prazeres^1^, A.V. Mendonca^1^, M.P. Paula^2^

##### ^1^Hospital Sao Domingos, ICU, Sao Luis, Brazil; ^2^Hospital Sao Domingos, Sao Luis, Brazil

###### **Correspondence:** J.R. Azevedo – Hospital Sao Domingos, ICU, Sao Luis, Brazil

**Introduction:** Cognitive dysfunction is an important long-term complication of critical illness associated with reduced quality of life, increase in healthcare costs and institutionalization. Delirium, an acute form of brain dysfunction that is common during critical illness has been shown to be associated with long-term cognitive dysfunction(1).

**Objectives:** The aim of this prospective cohort study was to estimate the prevalence and severity of cognitive dysfunction in survivors of critical illness and to evaluate if delirium duration is an independent determinant of the severity of cognitive dysfunction.

**Methods:** Included were all adult patients admitted to a 45-bed medical surgical ICU over a 12-month period(from March 2014 to February 2015).We excluded patients with preexisting cognitive dysfunction; those that in the evaluation by the psychologist on admission to the ICU had evidence of impaired cognition through the Mini Mental State Examination and patients who could not be reliably assessed for delirium owing to blindness, deafness or language deficit and patients for whom informed consent could not be obtained. After at least 3 months of hospital discharge patients were assessed for cognition using a validated battery of tests including:1)the Digit Span, forward and backward;2) the Rey Auditory Verbal Learning Test (RAVLT);3) the Clock Drawing Test (CDT);4) the Verbal Fluency Test; and the Mini Mental State Examination. We classified patients as having mild or moderate impairment if they had either two cognitive test scores 1.5 standard deviation (SD) below the mean or one cognitive test score 2 SD below the mean; we classified patients as having severe cognitive impairment if they had 3 or more cognitive test scores 1.5 SD below the mean or two or more cognitive test scores 2 SD below the mean.

**Fig. 52 (abstract A927).**
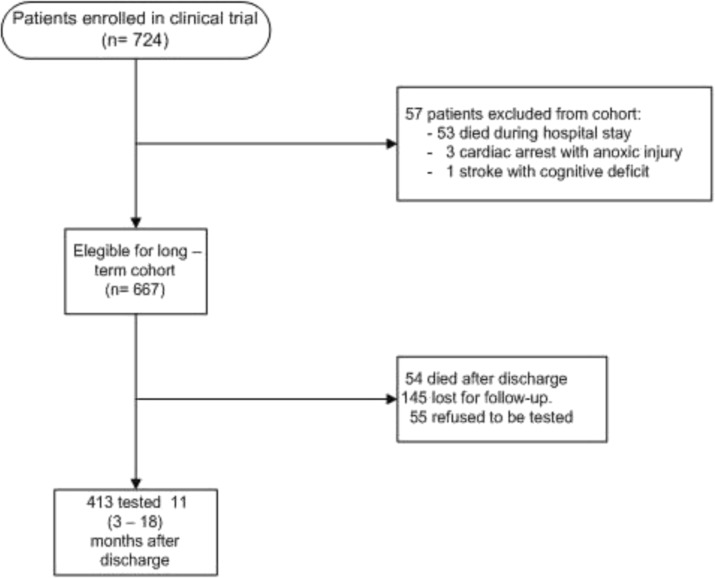
Enrollement and follow-up

**Results:** Enrolled in the clinical trial were 724 patients and 667 patients were eligible for the cohort (Fig. 52).

Four hundred and thirteen patients were tested 11 (03–18) months after discharge. Table 29 shows demographic and clinical data of these patients.

Cognitive impairment was identified in 206(49.9 %) patients; 120 (29.1 %) had mild or moderate and 86(20 %) severe cognitive dysfunction (Table 30).

Eleven(34.3 %) patients with delirium for 3 days or more presented severe cognitive dysfunction. In logistic regression analysis the duration of delirium for 3 days or more was not an independent predictor of cognitive dysfunction(p = 0.76).

**Conclusions:** This investigation in an unselected population of critically ill medical and surgical patients demonstrates that cognitive dysfunction is a frequent and severe long-term complication in survivors of critical illness. On the other hand, unlike other studies we couldn't demonstrate that the duration of delirium is an independent determinant of cognitive impairment.

**References**

1. Pandharipande PP,Girard TD,Jackson JC, et al. Long-term cognitive impairment after critical illness.N Engl J Med 2013;369:1306–1316.

**Table 29 (abstract A927).** Demograhic and Clinical Characteristics

	In - Hospital Cohort (n = 724)	Follow-up Cohort (n = 413)
Age, Yr Median (IQR)	59 (47–73)	57 (46–72)
Male sex n(%)	374 (51.7)	206 (50.1)
Education, yr Median (IQR)	11 (11–14)	11 (11–14)
APACHE IV Score Median (IQR)	35 (23–53)	32 (21–48)
SOFA Score at enrollment Median (IQR)	1 (0–3)	1 (1–3)
Delirium N° of Pacients (%)	80 (11.6)	55 (13.3)
Delirium N° of days Median (IQR)	4 (2–5)	3 (2–5)
Duration of hospital stay, days Median (IQR)	11 (6–23)	10 (5–19)

IQR = Interquatile Range; APACHE = Acute Physiology and Chronic Health Evaluation; SOFA = Sequential Organ Failure Assessment;

**Table 30 (abstract A927).** Diagnosis at admission

Diagnosis at admission n(%)	In-Hospital Cohort (n = 724)	Follow-up Cohort (n = 413)
AMI, CHF, Arrhythmia	146 (20.2)	98 (23.7)
Acute respiratory failure^a^	109 (15.1)	58 (14.0)
Other surgical procedures^b^	99 (13.7)	56 (13.6)
Neurologic disease	93 (12.8)	53 (12.8)
Severe sepsis, septic shock	77 (10.6)	37 (9.0)
Digestive Surgery	69 (9.6)	37 (9.0)
Digestive disease	67 (9.3)	39 (9.4)
Other disease	64 (8.7)	35 (8.5)

*AMI* Acute Myocardial Infarction, *CHF* Congestive Heart Failure

^a^Acute respiratory failure included acute respiratory distress syndrome, pneumonia, acute exacerbation of chronic pulmonary disease, asthma, pulmonary edema, embolism

^b^Other surgical procedures included orthopedic, vascular, urologic surgery

**Table 31 (abstract A927).** Cognitive outcomes during follow-up

	Follow-up Asjessment
	(n = 413)
No Impairment n(%)	207 (50.1)
Mild/Moderate n(%)	120 (29.1)
Severe Impairment n (%)	86 (20)

### A928 Predictors of long-term quality of life after ICU discharge: the Argentinian Caviuci study (evaluation of quality of life after ICU in Argentine)

#### A. Das Neves^1^, C.I. Loudet^1^, M. Busico^2^, D. Vazquez^3^, D. Villalba^4^, A. Lischinsky^5^, M. Veronesi^6^, M. Emmerich^7^, E. Descotte^8^, A. Juliarena^9^, M. Carboni Bisso^10^, M. Grando^11^, A. Tapia^12^, M. Camargo^13^, D. Villani Ulla^14^, L. Corzo^15^, H. Placido dos Santos^16^, A. Ramos^17^, J.A. Doglia^18^, E. Estenssoro^1^

##### ^1^HIGA Gral San Martin, La Plata, Argentina; ^2^Hospital de Trauma F Abete, Municipio Islas Malvinas, Argentina; ^3^Sanatorio Anchorena, Buenos Aires, Argentina; ^4^Hospital Municipal Chivilcoy, Chivilcoy, Argentina; ^5^Instituto De Neurología Cognitiva-INECO, Buenos Aires, Argentina; ^6^Instituto Fleni, Escobar, Argentina; ^7^Sanatorio Güemes, Buenos Aires, Argentina; ^8^Hospital Británico de Buenos Aires, Buenos Aires, Argentina; ^9^Hospital Austral, Pilar, Argentina; ^10^Hospital San Antonio, Gualeguay, Argentina; ^11^Clinica Bazterrica, Buenos Aires, Argentina; ^12^Hospital Domingo Funes, Santa Maria de Punilla, Argentina; ^13^Hospital San Luis, San Luis, Argentina; ^14^HIGA San José, Pergamino, Argentina; ^15^Hospital Artemides Zatti, Viedma, Argentina; ^16^Hospital de Gastroenterologia Udaondo, Buenos Aires, Argentina; ^17^Sanatorio Parque, Rosario, Argentina; ^18^Hospital Dr. Carlos Macías, Mar de Ajó, Argentina

###### **Correspondence:** A. Das Neves – HIGA Gral San Martin, La Plata, Argentina

**Introduction:** Long-term impact of critical illness on quality of life has been scarcely studied in Latin-America.

**Objectives:** To describe health-related quality of life (HRQOL) after ICU discharge and to identify its predictors.

**Methods:** The Argentinian Society of Critical Care launched a multicenter cohort study which enrolled patients receiving mechanical ventilation (MV) >72 hr.(3/5-8/31/2014). Epidemiological data and events were collected. Pre-ICU, and at 2, 6 and 12 months after discharge, HRQOL was recorded with the EQ-5D questionnaire (EQ-Index and EQ-EVA) together with relevant physical, neuropsychological and other variables. Values at different time points were compared versus pre ICU time with Wilcoxon signed rank test. We performed longitudinal data analysis to evaluate the effect of time on EQ-Index adjusting for important covariates, building a marginal model with generalized estimating equations.

**Fig. 53 (abstract A928).**
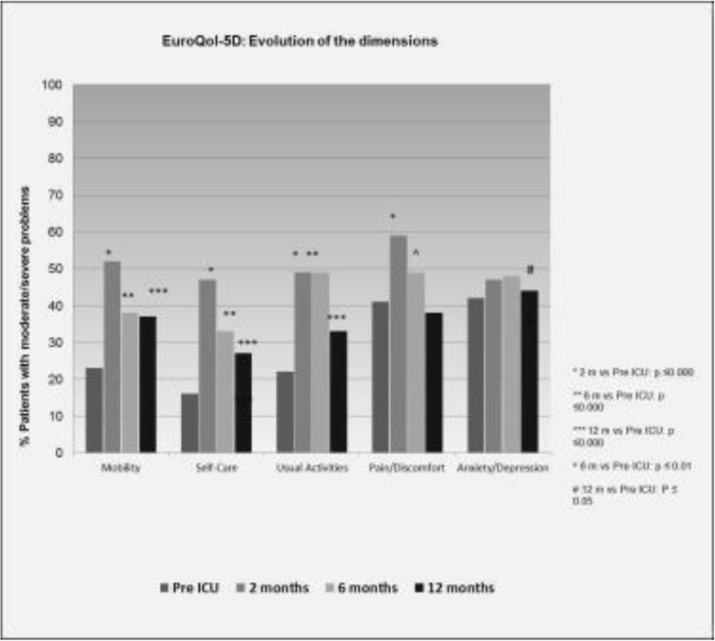
Evolution of dimensions of EuroQol-5D

**Fig. 54 (abstract A928).**
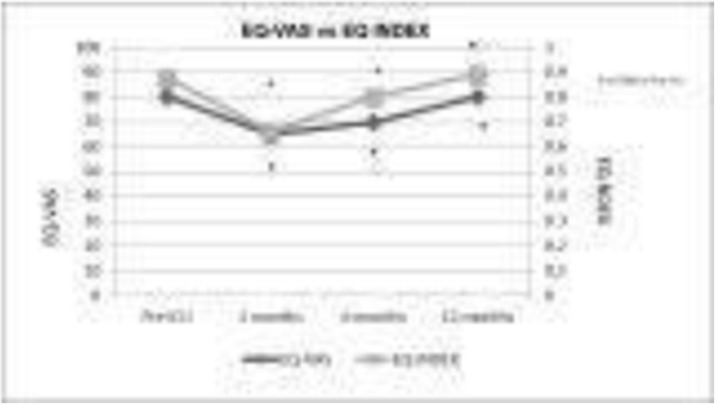
EQ-VAS vs EQ Index

**Results:** 320 patients from 26 ICUs were included:age 50[29–66], 41 % had Charlson score ≥ 2, 48 % married; 51 % occupied; 65 % from public hospitals; APACHE II 18 ± 7, SOFA24hs 7 ± 4; 51 % medical admissions; 23 % trauma; 51 % shock, 45 % ARDS. 1-year mortality was 22 %. EQ-index dimensions and evolution in time of EQ-index and EQ-EVA are shown in Figs. 53 and 54. Symptoms are displayed in Table 32

Positive determinants of the evolution of the EQ-index were time and admission Glasgow score (p 0.032 and 0.005 respectively) while age, duration of MV and weakness were negatively associated (p 0.000, 0.014 and 0.000) ]. EQ-EVA paralleled EQ-index changes.

**Conclusions:** After ICU discharge, patients suffered frequent long-term consequences that negatively affect their HRQOL. Alterations in mobility, daily activities and personal care exhibited the greatest deterioration.

Prevalence of pain, anxiety and depression was high even before ICU admission, aggravated after 1-year post-discharge (40 % of patients)

Duration of VM was the only intra-ICU variable that affected HRQOL. Pre-ICU conditions as age and the extent of neurological injury and, after ICU, time and weakness, were also independent determinants.

**Grant acknowledgment**

The present study was supported by the Argentinian Society of Critical Care (SATI)

**Table 32 (abstract A928).** Follow-up questionnaire

	Pre ICU	2 months	6 months	12 months	p
Caregiver	66 %	50 %	50 %	39 %	6 m vs. 2 m: p ≤ 0.000 12 m vs 2: p ≤ 0.000
Married/partnership	73 %	73 %	75 %	68 %	2 m vs. 0: p = 0.9 6 m vs 0: p = 0.9 12 m vs 0: p = 0.33
Weight	75 ± 18	69 ± 18	72 ± 16	73 ± 17	2 m vs. 0: p ≤ 0.000 6 m vs. 0: p ≤ 0.000 12 m vs 0: p ≤ 0.001
Paresia/paralysis		48 %	36 %	33 %	2 m vs. 6: p ≤ 0.000 2 m vs. 12: p < 0.000
Weakness		53 %	34 %	27 %	6 m vs. 2: p ≤ 0.000 12 m vs. 2: p < 0.000
Mood disorders		44 %	35 %	35 %	6 m vs. 2: p ≤ 0.05 12 m vs 2: p = 0.07
Psychological/Psychiatric therapy		24 %	25 %	24 %	6 vs. 2: p = 0.4 12 vs 2: p = 0.36
Memory problems		40 %	33 %	31 %	6 m vs 2: p = 0.16 12 m vs 2: p = 0.29
Occupation (Works or studies)	54 %	22 %	31 %	44 %	Pre UCI vs 2 m: p ≤ 0.000 Pre UCI vs 6 m: p ≤ 0.000 Pre UCI vs 12 m:p ≤ 0.001

## NON-INASIVE NEUROMONITORING

### A929 Longitudinal whole-brain analysis of white matter integrity in severe traumatic brain injury patients

#### M. Carbonara^1^, S. Magnoni^2^, C.L. Mac Donald^3^, J.S. Shimony^4^, V. Conte^2^, F. Triulzi^5^, F. Stretti^6^, M. Macrì^6^, A.Z. Snyder^4^, N. Stocchetti^2,6^, D.L. Brody^7^

##### ^1^Fondazione IRCCS Cà Granda-Ospedale Maggiore Policlinico, Department of Anaesthesiology and Intensive Care, Milan, Italy; ^2^Fondazione IRCCS Cà Granda-Ospedale Maggiore Policlinico, Department of Anaesthesiology and Intensive Care, Milano, Italy; ^3^University of Washington School of Medicine, Department of Neurological Surgery, Seattle, United States; ^4^Washington University, Mallinckrodt Institute of Radiology, St. Louis, United States; ^5^Fondazione IRCCS Cà Granda-Ospedale Maggiore Policlinico, Department of Neuroradiology, Milano, Italy; ^6^Milan University, Milano, Italy, ^7^Washington University, Department of Neurology, St. Louis, United States

###### **Correspondence:** M. Carbonara – Fondazione IRCCS Cà Granda-Ospedale Maggiore Policlinico, Department of Anaesthesiology and Intensive Care, Milan, Italy

**Introduction:** Diffuse axonal injury (DAI) is a common event following traumatic brain injury (TBI), which is likely related to worst long term outcome. Diffusion tensor imaging (DTI), a magnetic resonance imaging (MRI) technique that investigates white matter integrity, is recognized as a useful tool to quantify DAI extent in TBI and possibly predict outcome. Few studies explored whole brain longitudinal changes of DTI-derived parameters in single subjects following TBI.

**Methods:** Patients with severe TBI underwent brain MRI including DTI (32 directions, b = 1000, voxel size 2x2x2) 2–3 weeks and 1 year after trauma. 31 age-matched healthy controls underwent the same DTI protocol. We used region of interest (ROI) automated analysis (www.mristudio.org) covering the entire brain to quantify white matter integrity. The ROI fractional anisotropy (FA), mean diffusivity (MD), axial diffusivity (AD) and radial diffusivity (RD) were extracted. Abnormalities were defined as DTI values more than 2 standard deviations below or above the mean values of controls for each ROI.

**Results:** 14 TBI patients with a median age of 34 (IQR 20–38) and a median GCS score of 5 (IQR 4–7) were included. 11 had diffuse injury according to Marshall classification.

Regions with increased MD and reduced FA were more than expected in both early and late scan (p < 0.001 binomial test), while AD and RD abnormalities were less common. More than 50 % of the patients had increased MD in the early scan in the fronto-basal girae, corona radiata and thalami; in late scans MD abnormalities were larger and more diffuse, affecting also all frontal and temporal girae and corpus callosum. FA was frequently reduced in the corpus callosum, internal capsule and fronto-basal girae in early scan, while in late phase reductions were similar but more widespread, also including the central girae, cerebellum and inferior longitudinal fascicles.

The number of regions with abnormal MD increased over time (p < 0.01 Mann-Whitney), whereas for FA it was not statistically different.

An inverse correlation between the number of ROI with altered MD at early scan and outcome evaluated with GOSE was found (p < 0.05, Spearman r).

**Conclusion:** The present results indicate that early alterations of mean diffusivity and fractional anisotropy persist or worsen (for MD) at 1 year after TBI, suggesting an ongoing loss of white matter integrity and gliosis. The more frequently affected regions were the frontal girae, corpus callosum, corona radiata, inferior longitudinal fascicles and cerebellum. The number of ROI with early abnormal mean diffusivity is inversely correlated with outcome.

### A930 Application of evoked potentials for predicting swallowing disorders in early period after posterior fossa surgery

#### V. Podlepich, V. Shimanskiy, I. Savin, K. Lapteva, A. Chumaev

##### Burdenko Neurosurgery Institute, Moscow, Russian Federation

###### **Correspondence:** V. Podlepich – Burdenko Neurosurgery Institute, Moscow, Russian Federation

**Introduction:** Neurogenic dysphagia after removal of fossa posterior tumors (FPT) appears in 17 % of cases. The necessary level of upper airway protection depends on the severity of dysphagia. In our neuro-ICU, the clinical screening test (CST) and fibrooptic evaluation of swallowing test (FEST) are performed to determine the severity of swallowing disorders. The CST and FEST, however, require the patient´s collaboration during examination.

**Objectives:** We have evaluated evoked potentials using different modes (SSEP, AEP, TMS) to predict swallowing disorders in early FPT post-operative period in sedated patients.

**Methods:** The prospective study was carried out during the period from December 2013 to June 2014 and included 132 patients operated for non infiltrative paraxial fossa posterior tumors. All patients underwent SSEP, AEP and TMS the day before operation. After operation, all patients were delivered to ICU intubated and mechanically ventilated. 111 patients demonstrated full recovery from anesthesia with regaining consciousness, passed spontaneous breathing test (SBT) and gained 6 points on CST without deficiency. These patients had none or low level of dysphagia and were successfully extubated after operation. These patients formed 1st group. 21 patients had a neurogenic dysphagia and formed 2nd group. We performed SSEP, AEP and TMS on all patients immediately after admission to ICU.

**Results:** We revealed no clinical or electrophysiological points that could have predicted neurogenic dysphagia before operation. In our research, we found the EP values which were different for the first group and for the second group. The AEP and TMS data were not informative. We found instrumental the SSEP values that reflected perioperative CCT dynamics, lat p45, amp n13, AUC n11-n13, AUC n13-n18. These SSEP values were used to create a prognostic rule through logistic regression and ROC-curves. As a result, we were able to predict neurogenic dysphagia in early postoperative period in sedated patients with sensitivity 78,6 % and specificity 85 %.

**Conclusions:** Our study allows to predicting swallowing disorders in sedation in patients with paraxial FPT immediately after surgery through SSEP.

**References**

1. Goryachev A.S., Savin I.A., Putsillo M.V.; Scale evaluation and therapeutic strategy in violation of swallowing in patients with damage to the brain stem. Voprosy neyrokhirurgii №4 2006 str. 24–28 (in Russian).

2. Kang De-Zhi, Wu Zan-Yi, Lan; Combined monitoring of evoked potentials during microsurgery for lesions adjacent to the brainstem and intracranial aneurysms Chinese Medical Journal 2007; 120(18):1567–1573

3. Georg Neuloh, M.D., And Johannes Schramm, M.D; Monitoring of motor evoked potentials compared with somatosensory evoked potentials and microvascular Doppler ultrasonography in cerebral aneurysm surgery. J Neurosurg 100:389–399, 2004

### A931 The revised cerebral recovery index for prediction of neurological outcome after cardiac arrest at the bedside

#### M.C. Tjepkema-Cloostermans^1^, J. Hofmeijer^2,3^, A. Beishuizen^4^, H. Hom^4^, M.J. Blans^5^, M.J.A.M. van Putten^1,3^

##### ^1^Medisch Spectrum Twente, Clinical Neurophysiology and Neurology, Enschede, Netherlands; ^2^Rijnstate Hospital, Neurology, Arnhem, Netherlands; ^3^University of Twente, Clinical Neurophysiology, Enschede, Netherlands; ^4^Medisch Spectrum Twente, Intensive Care Medicine, Enschede, Netherlands; ^5^Rijnstate Hospital, Intensive Care Medicine, Arnhem, Netherlands

###### **Correspondence:** M.C. Tjepkema-Cloostermans – Medisch Spectrum Twente, Clinical Neurophysiology and Neurology, Enschede, Netherlands

**Introduction:** EEG monitoring during the first 24 hours robustly contributes to the prediction of either poor or good outcome in comatose patients after cardiac arrest [1]. Quantitative EEG (qEEG) measures can be useful to visualize evolution of the EEG over hours. We recently proposed the Cerebral Recovery Index (CRI), an index based on a combination of five qEEG measures grading the severity of hypoxic brain damage on a scale from zero to one to facilitate prognostication [2].

**Objectives:** To evaluate the prognostic accuracy of a revised CRI, after optimalization by the use of a random forest classifier instead of a manually chosen feature combination and the addition of four qEEG measures, resuscitation parameters and patient characteristics.

**Methods:** In this prospective cohort study, 283 consecutive comatose patients after cardiac arrest were included in two intensive care units. Continuous EEG was recorded during the first three days. Outcome at 6 months was dichotomized as good (CPC 1–2) or poor (CPC 3–5). Nine qEEG measures were extracted: alpha to delta ratio, signal power, Shannon entropy, delta coherence, regularity, the number of burst/min, mean and max burst correlation, and fraction of burst correlation >0.8. These measures were combined with patient characteristics and resuscitation data, including sex, age, initial heart rhythm, in- versus out-of-hospital-cardiac-arrest, and presumed cause of cardiac arrest.

Patients were randomly divided over a training and a validation set of respectively 143 and 140 patients. Within the training set, a random forest classifier was fitted for each hour after cardiac arrest. Based on results in the test set, two thresholds were chosen: one for predicting poor neurological outcome and one for predicting good neurological outcome. Subsequently, the revised CRI was evaluated in the validation set.

**Results:** Poor outcome could reliable be predicted with the revised CRI (with 100 % specificity) in the validation set with a sensitivity of 67 and 37 % at respectively 12 and 24 hours after cardiac arrest. Good neurological outcome could be predicted with a sensitivity of 60 and 62 % at a specificity of 94 and 93 %.

**Conclusions:** Here we show that a combination of qEEG and clinical measures, extracted and combined by a random forest classifier, provides reliable, objective prognostic information. This revised CRI can be used for the prediction of both poor and good neurological outcome, thereby poor outcome can be reliable predicted (without false positives) with relatively high sensitivity. The revised CRI is expressed as a single index between 0 and 1, which can be used in real time at the bedside, even by professionals who are not trained in EEG interpretation.

**References**

[1] Hofmeijer et al., Neurology, 2015

[2] Tjepkema-Cloostermans et al., Crit Care, 2013

**Table 33 (abstract A931).** Sensitivity and specificity of the revised CRI

	Predicting poor outcome		Predicting good outcome	
	Sensitivity	Specificity	Sensitivity	Specificity
At 12 h after CA	0.67	1.00	0.60	0.94
At 24 h after CA	0.37	1.00	0.62	0.93

### A932 From observation to monitoring: implementation of quantitative electroencephalography (QEEG) in neurointensive care

#### L. Longhi^1^, B. Frigeni^2^, M. Curinga^3^, D. Mingone^4^, S. Beretta^5^, A. Patruno^6^, L. Gandini^6^, A. Vargiolu^6^, F. Ferri^1^, R. Ceriani^1^, M.R. Rottoli^2^, L. Lorini^1^, G. Citerio^4,6^

##### ^1^Azienda Socio Sanitaria Territoriale Papa Giovanni XXIII, Anesthesia and Critical Care Medicine, Bergamo, Italy; ^2^Azienda Socio Sanitaria Territoriale Papa Giovanni XXIII, Neurology, Bergamo, Italy; ^3^University of Milan, Milano, Italy; ^4^University of Milan - Bicocca, Monza, Italy; ^5^Ospedale San Gerardo, Neurology, Monza, Italy; ^6^Ospedale San Gerardo, Anesthesia and Critical Care Medicine, Monza, Italy

###### **Correspondence:** L. Longhi – Azienda Socio Sanitaria Territoriale Papa Giovanni XXIII, Anesthesia and Critical Care Medicine, Bergamo, Italy

**Introduction:** Continuous electroencephalography (cEEG) allows real-time monitoring critically-ill patients neurophysiology and to detect non-clinical seizures in comatose patients, delayed cerebral ischemia after subarachnoid haemorrhage, and guide therapies for status epilepticus. The application of cEEG is still limited because it requires awkward analysis by experienced neurophysiologists of huge amount of EEG tracings. Quantitative EEG (qEEG) techniques, i.e.amplitude integrated EEG (aEEG) and Density Spectra Array (DSA), have been developed to simplify the complexity of EEG interpretation, to allow rapid evaluation of cerebral background electrical activity and the power spectrum of the EEG frequencies derived from raw data EEG. These developments offer the potentiality to transform an instrument interpreted by neurophysiologist afterwards in a monitoring tool useful to ICU staff.

**Objectives:** To test the hypothesis that EEG-nonexpert neurointensivists can obtain real-time reliable information from qEEG after training under the supervision of an in-house neurophysiologist. To describe the implementation of qEEG monitoring in 2 neurointensive care units.

**Methods:** The implementation occurred in sequential phases. cEEG was recorded using 8 surface electrodes according to the international 10–20 system, on a bipolar longitudinal montage in patients with brain injury. qEEG-naïve neurointensivists, after a short training from a neurophysiologist followed by daily supervision for the study period, were subjected to a baseline test evaluating aEEG and DSA traces. Each panel consisted of raw EEG data and 3 qEEG tools: the color density spectral array (DSA), amplitude integrated EEG (aEEG) and the burst suppression rate (BSR). After this evaluation, daily qEEG evaluation was performed by the neurointensivists and reviewed by the neurophysiologist.

**Results:** From July 2015 to April 2016 we monitored 77 patients (57 ± 17 years, 34 male) admitted for brain trauma (41 %), stroke (7 %), intracerebral hemorrhage (19 %), subarachnoid hemorrhage (18 %) and other neurological conditions (15 %). Median admission motor Glasgow coma scale (GCS) was 5 (range 1–6). Indications for cEEG were: unexplained clinical status (69 %), status epilepticus management (17 %) and titrating sedation for ICP control (14 %). cEEG monitoring was done for a median time of 2 days (range 1–8). EEG seizures were detected in 17 % of 45 patients with unexplained clinical status despite they received sedation with Propofol and/or antiseizures prophylaxis. qEEG monitoring guided patients management in 68 % of cases.

**Conclusions:** qEEG monitoring can be efficiently implemented as a real-time monitoring tool by EEG-nonexpert neurointensivists after proper training and under the supervision of a dedicated neurophysiologist. Further work is on-going to evaluate the learning curve of qEEG by naive neurointensivists.

### A933 Early suspect of cerebral vasospasm using transcranial color coded sonography monitoring in patients with subarachnoid haemorrhage

#### S. Pifferi^1^, M. Battistini^1^, V. Cordolcini^1^, A. Agarossi^2^, R. Di Rosso^2^, F. Ortolano^1^, N. Stocchetti^1,2^

##### ^1^Fondazione IRCCS Ca' Granda Ospedale Maggiore Policlinico, Neuroscience Intensive Care Unit, Milan, Italy; ^2^Università degli Studi di Milano, Milan, Italy

###### **Correspondence:** S. Pifferi – Fondazione IRCCS Ca' Granda Ospedale Maggiore Policlinico, Neuroscience Intensive Care Unit, Milan, Italy

**Introduction:** Cerebral vasospasm (VSP) is the narrowing of cerebral vessels. It occurs in up to 67 % of patients with aneurysmal subarachnoid haemorrhage (aSAH) and contributes to delayed cerebral ischemia (DCI) and neurological deterioration (symptomatic VSP = sVSP) in 30 % of patients. Transcranial color coded sonography (TCCS) is a non-invasive bedside technique that allows the measure of mean blood flow velocity (mFV) of cerebral arteries. The range of normality is 55–85 cm/sec, whereas mFV >120 cm/s is currently used as a threshold to define TCCS vasospasm (1).

**Objectives:** To analyse TCCS mFV variation over time in patients with aSAH and evaluate the prognostic utility of a daily TCCS monitoring.

**Methods:** All consecutive patients admitted to our ICU after aSAH were enrolled in the study. Mean blood flow velocity (mFV) of middle cerebral arteries on either side was obtained every 1–2 day for 14 days after the bleeding. We defined sVSP as a new neurological deterioration associated to radiological VSP evaluated with MRI angiography.

**Results:** 80 patients (age 26–80 years old) were included; 714 TCCS exams were performed. Only 13 patients (16 %) had normal TCCS mFV throughout the study period. 67 patients (84 %) presented mFV increase over time, with the highest values between 8 and 12 days after the bleeding (median 9, IQR 6–11). Among these, 49 patients (61 %) had mFV ≥120 cm/s and 21 (26 %) had mFV ≥200 cm/s. Symptomatic VSP occurred in 20 patients (25 %), the median time of neurological deterioration was 7 days (IQR 5–10) after the bleeding. Patients who developed sVSP (sVSP yes) displayed significantly higher mFV starting from day 5 up to day 14 compared to patients without sVSP (sVSP no); the median time of TCCS vasospasm (mFV >120 cm/s) was 4 days (IQR 3–6).

Accuracy of TCCS in diagnosing sVSP is displayed in the Table 34.

**Conclusions:** MeanFV time variation is significantly different in patients with or without sVSP. Daily TCCS monitoring with a threshold of 120 cm/s permits to identify 95 % of patients who will have symptomatic VSP but specificity is low. Patients at greater risk of sVSP are those with early mFV increase (>120 cm/s within 9 days), rapid mFV increase (>50 cm/s in 48 hours) or higher mFV absolute values (>200 cm/sec).

**References**

1) Zipper S et al. Clinical application of transcranial colour-coded duplex sonography - a review. Eur J Neurology 2002,9:1 ± 8

**Fig. 55 (abstract A933).**
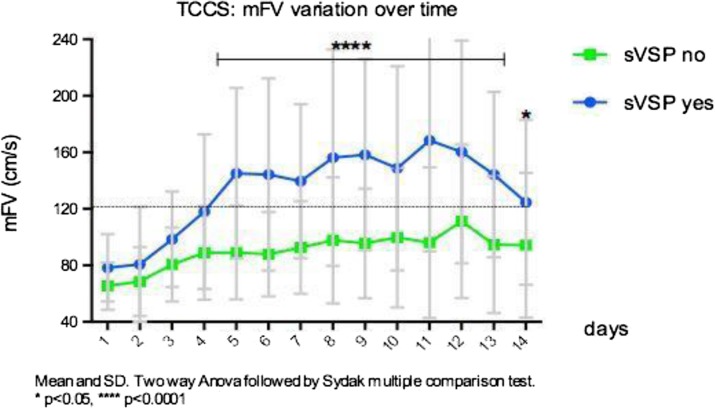
mFV time variation in the middle cerebral arteries

**Table 34 (abstract A933).** TCCS accuracy in predicting symptomatic vasospasm

TCCS thresholds	SENSITIVITY (CI 95 %)	SPECIFICITY (CI 95 %)	AUC
mFV >120 cm/s	95 (75–100)	50 (37–63)	0.90
mFV >120 cm/s (day 4–6)	70 (48–88)	68 (54–79)	0.80
mFV >120 cm/s (day 7–9)	85 (62–97)	70 (57–81)	0.86
mFV >200 cm/s	75 (51–91)	90 (79–96)	0.90
ΔmFV >50 cm/s in 48 hrs	90 (68–99)	63 (50–75)	0.90

## DIFFERENT ASPECTS ON OUTCOME IN THE ICU

### A934 Development and validation of a model to evaluate the application of human resources practices of high commitment, guided to the quality of service from the perspective of ICU healthcare staff

#### C. Mora Lourido^1^, J.L. Santana Cabrera^1^, J.D. Martín Santana^2^, L. Melián Alzola^2^, C. García del Rosario^1^, H. Rodríguez Pérez^1^, R. Lorenzo Torrent^1^

##### ^1^Hospital Insular Las Palmas GC, Las Palmas de Gran Canaria, Spain; ^2^Hospital Insular Las Palmas GC, Department of Economy, University of Las Palmas de Gran Canaria, Las Palmas de Gran Canaria, Spain

###### **Correspondence:** H. Rodríguez Pérez – Hospital Insular Las Palmas GC, Las Palmas de Gran Canaria, Spain

**Introduction:** It has been noted the importance of job satisfaction in healthcare services and the consequences resulting therefrom, such as increasing the quality of care services provided and satisfaction of their users.

**Objectives:** To develop a model of influence of human resource management directed to the quality management and organizational excellence in the organizational results, from the perspective of healthcare staff.

**Methods:** We carried out a research study, of a transversal nature, whose study population were a total of 248 (12,9 % physicians, 49,2 % nurses and 37,9 % nurse assistants) ICU staff. A personal questionnaire was used to measure, through Likert scales of 7 points, the application of human resource practices of high commitment (HR), the quality of service provided to the patient (QUALITY), the satisfaction with the capacity of the service (CAPACITY), the personal satisfaction with the work done (SATISFACTION) and the affective commitment with the organization (COMMITMENT).

**Results:** The measure models of these five constructs were validated by confirmatory factorial analysis, whose results were satisfactory. The measurement model of HR is a second order construct which is formed by 4 dimensions (training, participation, recognition, and communication). The QUALITY model is a model of second order formed by six dimensions (personal attention, ability of response, waiting room, tangibility, sensory experience, and meals). The CAPACITY model is a construct of second order formed by six dimensions referred to the satisfaction of different aspects (information, communication, facilities, additional services, decision maker's staff and non-decision maker's staff). The results of the PATH model indicate that it is an excellent model (CFI > 0,95 and RMFEA < 0,08). From the causal relationships of this model we can tell that (1) the HR influences positively and significantly on the QUALITY; (2) the HR and the QUALITY influence positively and significantly on the CAPACITY; (3) the RH and QUALITY influence on the SATISFACTION and (4) the CAPACITY and SATISFACTION influence positively and significantly on the COMMITMENT.

**Conclusions:** The results of this model indicates that the implementation of a HR strategy supported by a quality management significantly influences on the performance of the ICUs in terms of staff satisfaction, just as with the personal satisfaction and the commitment of healthcare workers.

**References**

1. Carrillo García C, Martínez Roche M, Gómez García CI y Meseguer de Pedro M. Satisfacción laboral de los profesionales sanitarios de un Hospital Universitario: análisis general y categorías laborales. Anales de Psicología 2015; 31 (2):645–650.

2. Newman K, Maylor U, Chansarkar B. “The nurse satisfaction, service quality and nurse retention chain: Implications for management of recruitment and retention. Journal of Management in Medicine 2002; 16 (4/5):271–291.

### A935 Sequential implementation changes affect glucose regulation in the ICU

#### S. Eslami^1,2^, A. Dalhuisen^3^, T. Fiks^3^, M.J. Schultz^4^, A. Abu Hanna^1^, P.E. Spronk^3,4^

##### ^1^Academic Medical Center, University of Amsterdam, Medical Informatics, Amsterdam, Netherlands; ^2^Mashhad University of Medical Sciences, Pharmaceutical Research Center, Tehran, Islamic Republic of Iran; ^3^Gelre Hospitals Apeldoorn, Intensive Care Medicine, Apeldoorn, Netherlands; ^4^Academic Medical Center, University of Amsterdam, Intensive Care Medicine, Amsterdam, Netherlands

###### **Correspondence:** P.E. Spronk – Academic Medical Center, University of Amsterdam, Medical Informatics, Amsterdam, Netherlands

**Introduction:** In the period between 2007–2010, a successful implementation project was finished aimed at strict blood glucose level (BGL) regulation in the Intensive Care Unit (ICU) [1]. We hypothesized that glucose control would afterwards slack and that implementing other measures to modify behavior would be required to regain adequate glucose control.

**Methods:** A prospective study was performed in a 12-bed mixed medical-surgical ICU of a university affiliated teaching hospital. All BGL values were extracted from the ICU database in 4 years following the implementation project until December 2014. Following the project, BGL targets were set at a range of 80–135 mg/dl, Nurses' instructions for keeping BGL values in target were not changed. After 3.5 years, an automated warning system was implemented in the patient data management system that triggered a centrally placed monitor with feedback about the need for obtaining a BGL value, based on the actual value compared to the previous one. The primary outcome measure was mean BGL. Secondary endpoints were sampling frequency, BGL within pre-defined targets, incidences of severe hypoglycemia, and hyperglycemia. The analysis was restricted to patients with at least two blood glucose measurements. These indicators were analyzed over the course of time using the XMR control chart, a tool belonging to Statistical Process Control.

**Fig. 56 (abstract A935).**
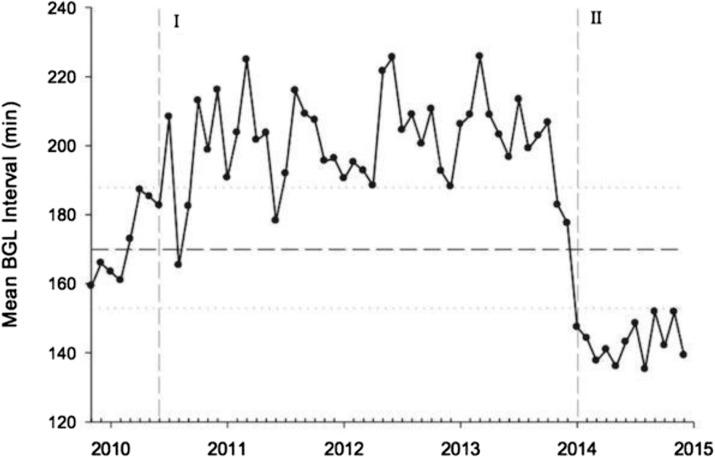
ᅟ

**Results:** Data of 3760 patient admissions were evaluated, which corresponded to 117,080 BGL measurements. The BGL sampling interval (Figure 56), mean BGL and percentage of severe hypoglycemia all increased after introducing nurses' instruction and decreased significantly after monitoring feedback (p < 0.05). Percentage of severe hypoglycemia events, which is associated tosafety, decreased with some delay after nurses' instruction and remained unchanged (0.18 % on average) and stable after introducing monitoring feedback. Percentage of “in range” measurements of both normoglycemia (80–110) and protocol recommended (80–135) decreased after nurses' instructions and then increased after feedback monitoring. Mean of per patient's standard deviation as a measurement of variability remained unchanged and stable after nurses' instruction and even decreased after monitoring feedback.

**Conclusion:** even after successful implementation of a BGL control system, behavior changed within 3 months with inherent worsening of BGL control. An automated warning monitor in a central location was able to restore BGL control in the ICU.

**References**

1. Schultz MJ et al. Minerva Anestesiol 2012; 78:982–995

### A936 Using robotic technology to precisely define the neurocognitive phenotype of ICU survivors

#### M. Wood^1^, D. Maslove^2^, J. Muscedere^2^, S.H. Scott^1^, T. Saha^3^, A. Hamilton^4^, D. Petsikas^4^, D. Payne^4^, J.G. Boyd^1,5^

##### ^1^Queen's University, Centre for Neuroscience Studies, Kingston, Canada; ^2^Queen's University, Medicine and Critical Care, Kingston, Canada; ^3^Queen's University, Anaesthesia and Perioperative Medicine, Kingston, Canada; ^4^Queen's University, Surgery, Kingston, Canada; ^5^Queen's University, Medicine (Neurology) and Critical Care, Kingston, Canada

###### **Correspondence:** J.G. Boyd – Queen’s University, Centre for Neuroscience Studies, Kingston, Canada

**Introduction:** Long-term cognitive impairment is common among ICU survivors, and is an important component of the post-ICU syndrome. There is mounting evidence that conventional screening tests are insufficient to capture the degree of cognitive dysfunction experienced by ICU survivors. The KINARM robot provides robust, objective, and quantitative metrics of the sensorimotor and neurocognitive impairments in humans. It has been used to describe impairments post stroke and importantly, deficits identified with the KINARM correlate well with reductions in quality of life.

**Objective:** The overall objective of this research program is to use the KINARM to define the neurocognitive phenotype of ICU survivors (i.e. required invasive mechanical ventilation and/or vasoactive agents for hemodynamic support). This group is compared to healthy age- and gender-matched controls, as well as 3 active control groups. These active control groups were patients 1) pre- and 2) post-cardiac surgery, and 3) patients post-cardiac arrest.

**Methods:** Participants performed 7 tasks on the KINARM that ranged from simple sensorimotor tasks to more complex executive tasks. For each task, 6–12 performance metrics were recorded. These metrics were compared to a normative database of age- and gender-matched controls and z-scores were generated. A composite score for each task was generated using a score derived from Maholanobis distance, with increasing scores representing worse performance. Cluster analysis was applied to these performance metrics using Euclidian distance.

**Results:** From February 2014-February 2016, we have performed 41 KINARM assessments (12 pre-cardiac surgery, 12 post-cardiac surgery, 10 post critical illness, 7 post cardiac arrest). Compared to all groups, survivors of critical illness performed worse on several tasks that assess the visuomotor control of the upper limb, interlimb coordination, proprioception, and visuospatial processing. Impairments in executive tasks were noted across all participants. While the KINARM tasks tended to cluster together (i.e. visuospatial, executive), the patient cohorts did not.

**Conclusions:** Robotic technology can provide precise and quantitative metrics of the sensorimotor and cognitive deficits after critical illness. These strategies may be able to define a specific neurocognitive phenotype of the ICU survivor.

**Grant Acknowledgement**

This work is supported by Physician Services Incorporated and the Southeastern Ontario Medical Associations' New Clinician-Scientist Program.

### A937 Relationship between changes in rectus femoris/vastus intermedius muscle layer thickness and rectus femoris cross-sectional area in critical illness

#### Z.A. Puthucheary^1,2,3^, A.S. McNelly^2,4^, J. Rawal^4^, B. Connolly^5,6^, M.J. McPhail^7,8^, P. Sidhu^9^, A. Rowlerson^10^, J. Moxham^11^, S.D. Harridge^10^, N. Hart^6,11^, H.E. Montgomery^2,4^

##### ^1^University College London Hospitals NHS Foundation Trust, Division of Critical Care, London, United Kingdom; ^2^University College London Hospitals, NIHR Biomedical Research Centre, London, United Kingdom; ^3^National University Health System, Division of Respiratory and Critical Care, Singapore, Singapore; ^4^UCL/University College London Hospitals NHS Foundation Trust, Institute of Sport, Exercise and Health, London, United Kingdom; ^5^Guy's & St Thomas' NHS Foundation Trust and King's College London, NIHR Biomedical Research Centre, London, United Kingdom; ^6^Guy's & St Thomas' NHS Foundation Trust, Lane Fox Clinical Respiratory Physiology Research Centre, London, United Kingdom; ^7^St Mary's Hospital and Imperial College London, Hepatology & Gastroenterology, London, United Kingdom; ^8^Kings College Hospital NHS Foundation Trust, Institute of Liver Studies, London, United Kingdom; ^9^Kings College Hospital NHS Foundation Trust, Department of Radiology, London, United Kingdom; ^10^Kings College London, Centre of Human and Aerospace Physiological Sciences, London, United Kingdom; ^11^King's College London, Department of Asthma, Allergy and Lung Biology, London, United Kingdom

###### **Correspondence:** Z.A. Puthucheary – University College London Hospitals NHS Foundation Trust, Division of Critical Care, London, United Kingdom

**Background.** Muscle wasting impairs function in Intensive Care Unit (ICU) survivors. Limitations to its non-invasive evaluation hamper the development of preventative or therapeutic interventions.

**Aims and objectives:***To assess the utility and validity of two ultrasound methods in indicating loss of muscle bulk and function.* Quadriceps Femoris muscle (histological) fibre cross sectional area (F_CSA_) and (biochemical) protein/DNA ratio are 'gold standard' indices of muscle mass. We compared changes in these measures with those in ultrasound-assessed thickness of the Rectus Femoris and Vastus Intermedius muscles (muscle layer thickness, MLT), Rectus Femoris cross sectional area (RF_CSA_), and Quadriceps Femoris strength during a 10-day ICU stay.

**Methods: Subjects.** drawn from the Musculoskeletal Ultrasound Study in Critical Care: Longitudinal Evaluation Study (NCT01106300), invasively ventilated for >48 hours and on ICU >7 days.^1^ Protein/DNA ratio, F_CSA_: from Vastus Lateralis muscle biopsies.^1,2^ MLT, RF_CSA_: by ultrasound. ^3^ Knee extensor (KE) strength: from Medical Research Council (MRC) Score.^4,5^

**Fig. 57 (abstract A937).**
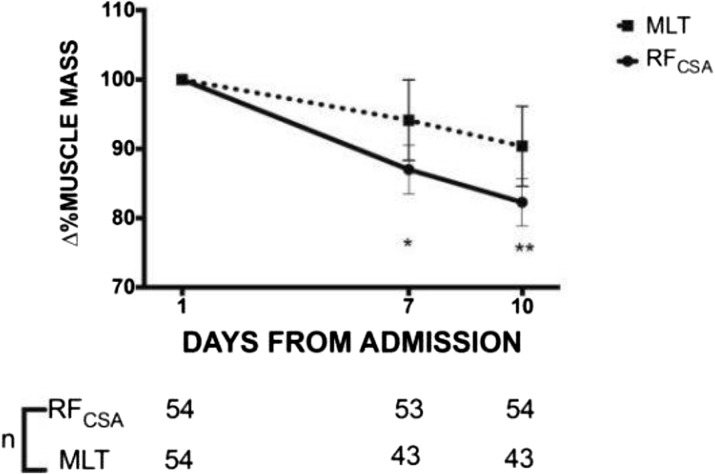
Change in Rectus Femoris Cross Sectional Area (RF_CSA_) and Muscle Layer Thickness (MLT) over 10 days of critical illness

**Results:** Data were collected from 54 patients (63 % male; mean age: 55.6 [95%CI 49.8 - 59.3] years). MLT underestimated muscle wasting at Day 7 assessed by F_CSA_ (−4.6 % [95%CI −14.2-5.0] vs. -16.4 % [95%CI −32.0- -0.7]; p = 0.025) and protein/DNA ratio (−4.6 % [95%CI −14.2-5.0] vs. -30.9 % [95%CI −51.2- -10.6]; p = 0.019) respectively [n = 19]. RF_CSA_ is better related than MLT to F_CSA_ (−10.3 % [95%CI −14.5- -6.1] vs. -17.5 % [95%CI −29.3- -5.8]) and protein/DNA ratio (−10.3 % [95%CI −14.5- -6.1] vs. -29.5 % [95%CI −45.6- -13.4]) respectively [n = 28]. MLT thus underestimated muscle wasting assessed by RF_CSA_ at Day 7 (−5.9 % [95%CI −11.7- -0.1] vs. -13.0 % [95%CI −16.5- -9.5]; p = 0.031*) and Day 10 (−9.4 % [95%CI −15.4- -3.8] vs. -17.7 % [95%CI −21.2- -14.3]; p = 0.004**) (Fig. 57).

**Fig. 58 (abstract A937).**
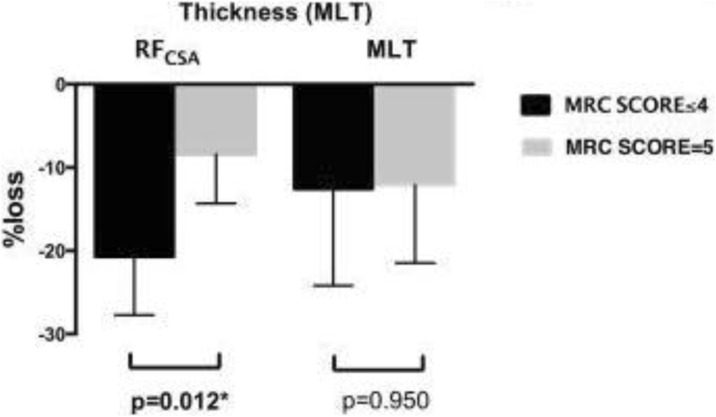
Knee Extensor Medical Research Council (MRC) score and loss of muscle mass as measured by Rectus Femoris Cross Sectional Area (RF_CSA_) and Muscle Layer Thickness (MLT)

Change in RF_CSA_ was associated with reduced MRC KE score of ≤4 (OR 1.1 [95%CI 1.0-1.2]; p = 0.027) but that in MLT was not (OR 1.0 [95%CI 0.1-1.0]; p = 0.947) [n = 27] (Fig. 58).

**Conclusions:** Serial MLT measurements significantly underestimate muscle wasting in critical illness and are not related to development of muscle weakness. In comparison, changes in RF_CSA_ reflect changes in 'gold standard' methods of assessing muscle mass, and are related to loss of muscle mass and function in critically ill patients.

**References**

1. Puthucheary Z et al. *JAMA*. 2013;310(15):1591–1600.

2. Wilkes E et al. *Am J Clin Nutr*. 2009;90(5):1343–1350.

3. Seymour J et al. *Thorax*. 2009;64(5):418–423.

4. Kleyweg R et al. Muscle Nerve 1991,14:1103–1109.

5. Connolly B et al. *Crit Care*. 2013;17(5):R229.

**Grant Acknowledgement**

AM: Moulton Foundation; NIHR BRC University College London Hospitals. ZP: NIHR. HM: UCL; NIHR BRC University College London Hospitals. Additional funding: ESCIM; NIHR Clinical Research Facility Guy's and St Thomas' Hospital; NIHR BRC Guy's and St Thomas' Hospital/KCL; Whittington Hospital.

### A938 One year outcomes of multidisciplinary intervention to improve medicines safety on ICU

#### T. Jovaisa^1^, B. Thomas^1^, D. Gupta^2^, D.S. Wijayatilake^1^

##### ^1^Queen's Hospital, Critical Care Department, Romford, United Kingdom; ^2^Queen's Hospital, Pharmacy Service, Romford, United Kingdom

###### **Correspondence:** T. Jovaisa – Queen's Hospital, Critical Care Department, Romford, United Kingdom

**Introduction:** Critically ill patients are at increased risk of iatrogenic complications due to frequent and complex interventions, multiple prescriptions and potentially higher degree of harm. Previous studies demonstrate that majority of medical errors in ICU are related to medicines management^1^. Clinical practice review of our unit in March 2015 revealed a number of errors and inconsistencies in medicines management. Majority of issues were related to prescriptions of continuous intravenous infusions, other major categories were validity of prescription, administration of medication against invalid prescription and omitted doses.

There is significant evidence that electronic prescribing can significantly reduce the errors, however implementation of it is a long term project and is not feasible in attempt to improve medicines safety over short period of time. Therefore we aimed to improve safety of a current paper based system. Multidisciplinary intervention was chosen as this approach has been previously demonstrated to reduce medication errors on ICU^2^.

**Objectives:** Evaluate effect of multidisciplinary intervention to improve medicines safety.

**Methods:** Over the course of 12 months following interventions were introduced: development and implementation of new ICU specific IV infusion chart, prescription checks during nursing handover, introduction of daily pharmacy handover and on-site feedback, additional medicines training for current staff and new medicines safety induction module for new-starters.

Outcome data was based on monthly spot audits carried out by pharmacy staff. Comparison is made between Quarter 2 and Quarter 4 after the start of intervention. Chi-square test was used to compare the two datasets.

**Fig. 59 (abstract A938).**
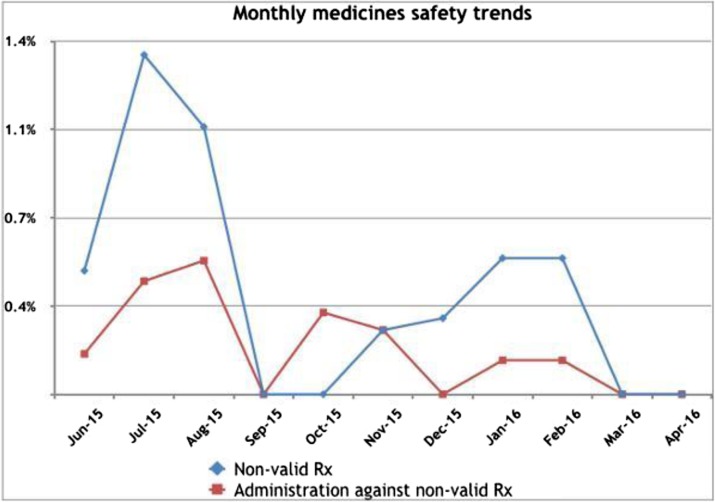
Monthly medicines safety trends

**Results:** There were 1847 prescriptions analysed in Q2 and 2293 in Q4. We observed a five-fold reduction in prescription validity errors from 0.97 % to 0.17 % (p < 0.001). And nearly ten-fold reduction in administration of medicines against non-valid prescriptions from 0.38 % to 0.4 % (p < 0.001). Pre-printed ICU specific IV infusion chart eliminated errors related to variable dilutions, choice of diluent, incorrect or inconsistent infusion rates. Month-by-month trends are presented in Figure 59.

**Conclusion:** Multidisciplinary intervention has resulted in significant improvement in medicines safety.

**References**

1. Rothschild J, et al. The Critical Care Safety Study: The incidence and nature of adverse events and serious medical errors in intensive care. *Critical Care Medicine*. 2005; 33 (8)1694-1700

2. Romero C, et al. Effects of the implementation of a preventive interventions program on the reduction of medication errors in critically ill adult patients. *Journal of Critical Care*. 2013; 28 (4) 451–60

## Poster Corner Sessions. QUALITY IMPROVEMENT & AGE

### A939 Two years outcomes of elderly receiving intensive care

#### H.P. Shum^1^, H.S. King^1^, K.C. Chan^2^, K.B. Tang^1^, W.W. Yan^1^

##### ^1^Pamela Youde Nethersole Eastern Hospital, Hong Kong, Hong Kong, China; ^2^Tuen Mun Hospital, Hong Kong, Hong Kong, China

###### **Correspondence:** H.P. Shum – Pamela Youde Nethersole Eastern Hospital, Hong Kong, Hong Kong, China

**Introduction:** As the general population ages, an increasing number of patients > =80 years are being admitted to intensive care unit (ICU). Their medium team outcomes are not clear when compared with patients between 60–79 years old.

**Objectives:** To evaluate the clinical outcome (2-year mortality) of very elderly critically ill patients (> = 80 years old) and compared with those between 60 and 79 years old.

**Methods:** Retrospective analysis of administrative data of patients admitted between 1/1/2009 and 31/12/2013 to an ICU of a regional hospital in Hong Kong.

**Results:** Over 5 years, 4226 patients aged ≥60 years old were admitted (55.5 % total ICU admission) and 32.8 % with age ≥80 years old. Proportion of patients > =80 years old increased over 5 years (16.2 % in 2009, 18.9 % in 2010, 16.0 % in 2011, 20.3 % in 2012, and 19.4 % in 2013; P = 0.006).. Those > =80 years old carried more significant co-morbidities and with higher disease severity. They required more ventilatory support, less likely to receive renal replacement therapy and with higher ICU/hospital/180-day and 2-year mortality when compared with those aged 60–79. 71.8 % could be discharged home while 47.6 % can survive 2 years from ICU admission. Cox regression analysis revealed that age, APACHEIV-minus-Age score, gender, medical or neurosurgical cases, presence of significant co-morbidities (chronic renal failure, metastatic carcinoma, lymphoma, leukemia or myeloma), regular use of immunosuppressant and requirement of mechanical ventilation or renal replacement therapy during ICU stay independently predicted 2-year mortality.

**Conclusions:** The proportion of critically ill patients with age > =80 years old increased over 5 years. They had more significant comorbidities, greater disease severity, higher ICU/hospital/180-day and 2-year mortality when compared with those aged 60–79. After adjustment with disease severity and other significant factors, their 2-years mortality is 1.8 times higher than those aged 60–69. Less than half (47.6 %) of them survived 2 years from ICU admission.

### A940 Outcomes in elderly patients admitted to ICU

#### C. Castro Arias, J. Latorre, A. Suárez De La Rica, E. Maseda Garrido, A. Montero Feijoo, C. Hernández Gancedo, A. López Tofiño, F. Gilsanz Rodríguez

##### Hospital Universitario La Paz, Madrid, Spain

###### **Correspondence:** C. Castro Arias – Hospital Universitario La Paz, Madrid, Spain

**Introduction:** It is quite frequent for elderly patients to die in the hospital after prolonged stay in the ICU with life-sustaining interventions (LSI).

In many cases we prolong survival, reducing their quality at the end of life.

**Objectives:** The aim of this study is to evaluate outcomes in patients ≥ 80 years old admitted to the surgical ICU, and also the need for LSI.

**Methods:** This is an observational retrospective study. All the patients older than 80 years admitted to our surgical ICU between June 2012 and June 2015 have been included.

We have studied the mortality of these patients both in the ICU and in the hospital, the length of stay and the need for LSI.

We also have analysed the risk factors associated to both ICU and hospital mortality.

**Results:** 299 patients were included (of a total ICU population of 2492 patients in that period). Average age of our patients was 84.43 ± 3.55 years. Mean SAPS II was 45.48 ± 14.59.

Mean length of stay in ICU and in hospital were 5.47 ± 7.61 and 18.15 ± 15.27 days, respectively.

Mortality in ICU has resulted in 18.1 %, whilst in the hospital, in global terms, it has resulted in 26.8 %.

Regarding the need for LSI, 10.0 % underwent renal replacement therapy, 50.2 % required vasopressors and 34.8 % required mechanical ventilation during more than 24 hours.

Patients who received vasopressors had a hospital mortality of 46.0 %, those receiving mechanical ventilation during more than 24 h had a mortality of 58.7 %, and patients receiving renal replacement therapy had a mortality of 80.0 %.

Of the patients who died in the hospital, 86.2 % were treated with vasopressors, 76.2 % received mechanical ventilation during more than 24 hours and 30.0 % underwent renal replacement therapy.

50 % of patients dying in the ICU died at least 17 days after admission. 25 % of the patients died at least 42 days after ICU admission.

Factors independently associated with hospital mortality were age (OR 1.12, 95%CI = 1.054-1.192), SOFA score (OR 1.154 , 95 % CI = 1.079-1.235), need for renal replacement therapy (OR 1.924, 95%CI: 1.121-3.302) and need for mechanical ventilation during more than 24 hours (OR 3.144 , 95 % CI: 1.771-5.584).

**Conclusions:** The decision on the limitation of LSI is not always easy and it is critical to maintain a good doctor-patient communication.

By stopping extraordinary treatments when appropriately indicated, life quality at the final stages of elderly patients would improve.

We consider that is important to raise ethical issues for health-related professionals on the utilization of aggressive life-sustaining interventions at the final stages of elderly patients' life at surgical ICUs.

**References**

1. Heyland D et al. The Very Elderly Admitted to ICU: A Quality Finish? Crit Care Med. 2015 Jul;43(7):1352–60.

### A941 Frailty in intensive care: a retrospective review

#### L.K. Gemmell, R. Campbell, P. Doherty, A. MacKay

##### Queen Elizabeth University Hospital, Anaesthetics and Intensive Care, Glasgow, United Kingdom

###### **Correspondence:** L.K. Gemmell – Queen Elizabeth University Hospital, Anaesthetics and Intensive Care, Glasgow, United Kingdom

**Introduction:** The concept of frailty has been defined as a multidimensional syndrome characterised by the loss of physical and cognitive reserve that predisposes to adverse events. The prevalence of frailty amongst the critically ill is unknown, however it is probably increasing. This audit aimed to look retrospectively at our admissions to Intensive Care, to categorise them into frail or non frail, and evaluate how frailty correlated with ICU length of stay and mortality

**Methods:** A retrospective case note review of all patients admitted to Intensive Care over a six month period in the Victoria Infirmary and then Queen Elizabeth University hospital in Glasgow. Classification of frail or non-frail was done using a combination of the Clinical Frailty Score (CFS) and Edmonton frailty scale.[1,2]. Once classified into frail and non-frail we looked at ICU outcome, length of stay, APACHE, weight on admission, lowest albumin and admission haemoglobin and compared the frail population to the non-frail population.

**Results:** Two hundred and eighty four patients were admitted to Intensive Care in this time period. Of those, 102 were over the age of 65 years. Of the 102 patients, 68patients were deemed to be frail, and 34 were deemed to be non-frail using the CFS. Approximately 40 % of the patients admitted to Intensive Care are over the age of 65. There was no significant difference found in mortality, ICU length of stay or hospital stay, APACHE or weight between the two groups. [see Table 35]

**Conclusions:** We know that the utilisation of intensive care resources by older people is rising. Our data shows that almost 40 % of those admitted to ICU are over the age of 65. Interestingly, there is no significant difference between the non frail and frail groups of patients admitted to intensive care. This may be because of small sample size. The length of stay of the frail patient is shorter and this may be because as intensivists we are better at treatment limitation in this group of patients. No difference in overall mortality suggests that the patients we deem suitable for intensive care who are frail do as well as the non-frail cohort as the selection process for admission has been adequate. Patients deemed to be frail are more likely to be dependant on care if they survive, with 50 % requiring some sort of support on discharge.

Most studies show that frailty is associated with increased mortality so it is indeed interesting that this audit has shown no difference between the two groups.

**References**

1. Rockwood, Song, McKnight. A global clinical measure of fitness and frailty in elderly people.CMAJ: 2005, vol 173 no.5

2. The Edmonton Frailty Scale. Age and Ageing, volume 35.

**Table 35 (abstract A941).** Frailty

	Frail	Non-frail	p-value
Age (years)	75.1 +/− 1.7	73 +/− 2.1	0.14
Mortality (%)	55.1 %	54.3 %	0.94
ICU days	5.1 +/− 1.3	7.6 +/− 3.7	0.128
Total LOS (days)	22.7 +/− 5.5	22.9 +/− 8.9	0.98
APACHE II	24.6 +/− 2.1	24.4 +/− 2.7	0.94
Pred. mortality (%)	50.3 +/− 6.3	49.5 +/− 9.4	0.881
Weight (kgs)	70.2 +/− 5.1	75.6 +/− 7.1	0.224
Albumin (g/dl)	25 +/− 2	27 +/− 3	0.154
Hb (g/dl)	108 +/− 6.5	120 +/− 7.4	0.044

### A942 One year survival data for patients aged over 80 admitted to two British general intensive care units

#### N. Singh

##### Royal Berkshire Hospital, Critical Care Unit, Reading, United Kingdom

**Introduction:** Over the last decade increasing numbers of elderly patients are being admitted to intensive care units (ITU). This reflects an aging population and increasing expectations of care delivered to the elderly. However it remains unclear if admission to ITU changes the eventual outcome in this patient group. Several studies have shown poor outcomes in elderly patients. de Rooij et al. reported one year survival of 57 % in those over 80 admitted after planned surgery, compared with only 11 % survival in those admitted after unplanned surgery or from medical causes^1^. Biston et al. reported one year mortality of 97 % in patients aged over 85 treated for circulatory failure^2^.

**Objectives:** This service evaluation assesses 1 year survival of patients over the age of 80 admitted to a tertiary centre and a large district general hospital ITU in Britain in 2013 to allow comparison to published data.

**Methods:** Admission patient data was obtained from the ITU clinical information system for 2013. The hospital electronic patient record was used to determine the last point of patient contact with the hospital. If this was less than a year after initial admission and the patient was documented as alive they were assumed to have lived to a year for the purpose of data analysis.

**Results:** 1884 patients were admitted to the ITUs, of which 208 were aged 80 or above. Data was not available for one patient. Of the 207 patients for whom data was available, the mean age was 84.3 years. Data for 23 of these patients was not available to a year. Of the 133 surgical patients, 49(36.8 %) were admitted after elective and 84(63.2 %) after emergency surgery. At one year, 39 of the elective patients were alive and 31 of the emergency patients. Of the 74 medical patients only 24(32.4 %) patients survived to 1 year. Within both surgical and medical groups 61(29.5 %) patients were admitted with sepsis and 42(68.9 %) were dead after 1 year. Of the 207 patients, 94(45.4 %) survived to one year or more and 113(55.6 %) did not. If the patients who were admitted after elective surgery were excluded that left 158 unplanned admissions in total, with only 55(34.8 %) patients surviving for more than one year.

**Conclusions:** Although this data shows better survival for unplanned admissions than previous studies, outcomes are still poor for elderly patients who are admitted to ITU. This should inform discussions with patients and their relatives relating to admission to ITU and escalation of care.

**References**

1. de Rooij SE, Govers AC, Korevaar JC, Giesbers AW, Levi M, de Jonge E. Cognitive, functional, and quality-of-life outcomes of patients aged 80 and older who survived at least 1 year after planned or unplanned surgery or medical intensive care treatment. J Am Ger Soc. 2008;56(5):816–822.

2. Biston P, Aldecoa C, Devriendt J, Madl C, Chochrad D, Vincent JL, De Backer D. Outcome of elderly patients with circulatory failure. Int Care Med. 2014 Jan;40(1):50–6.

**Grant acknowledgment**

None.

### A943 The implantation of an antimicrobial stewardship programme in intensive care units is also possible. Analysis of the results of the first year in a secondary Andalusian hospital

#### S. Vitaller, H. Nagib, J. Prieto, A. Del Arco, B. Zayas, C. Gomez

##### Hospital Costa del Sol Marbella, Malaga, Spain

###### **Correspondence:** S. Vitaller – Hospital Costa del Sol Marbella, Malaga, Spain

**Background:** Despite the fact that there is evidence of results of the stewardship programme in conventional hospitalization, results in intensive care units are more limited. The aim of this review is to analyse the results of the first year of stewardship programme implantation in the intensive care unit of an andalusian second level hospital.

**Material/methods:** HCS is a secondary hospital, which provides 350 beds. It has a general ICU with 12 beds. Since 2014, there is a program to optimize antibiotic therapy in ICU, where the services of microbiology, pharmacy, infectious diseases and ICU are involved. The pharmacy department sends a daily report with the broad spectrum antibiotics prescribed in the ICU and they are contrasted with the available microbiological data of all patients admitted to the unit. The main objective is the reduction of antibacterial spectrum when feasible and the duration of treatment. In this paper, data from 2014 is contrasted with data from 2013. Antimicrobial consumption, impact on infections of special microorganisms, and savings in annual drug spending are evaluated.

Throughout 2014, the ICU team evaluated 108 cases of patients admitted to the unit with broad-spectrum antibiotic treatment. In 46 cases (42.5 %), a change to narrower antibiotic spectrum guided by antibiogram was performed. In 62 cases (57,4 %), the initial treatment was maintained. Carbapenems (IMP or MER) explained 42 cases (38.9 %), aztreonam 5 cases (4.6 %), linezolid 41 cases (37.9 %), daptomycin 5 cases (4.6 %) anidalafungin 7 cases (6.48 %). Comparing 2014 with 2013, DDD/1000 admissions of imipenem was 149 against 155, and DDD/1000 hospital stay was 56 versus 68; to meropenem DDD/1000 admissions was 189 against 343 and DDD/1000 hospital stay was 71 versus 110. The number of isolates of *Pseudomonas aeruginosa* in first sample was 15 cases in 2013 compared to 11 in 2014. The strains with resistance or intermediate sensitivity to IMP were 5/15 in 2013 (33 %), while it was only 1/11 (9 %) in 2014. First samples of MRSA, in 2013, were 3, whereas there was only one in 2014. Two cases of *Klebsiella pneumoniae* ESBL were identified in 2013 and 3 cases in 2014. There was one case of *Clostridium difficile* in 2013 and none in 2014. Expenses in antimicrobials, antifungals included, decreased by 19 % (11,000 euros).

**Conclusions:**

1. Implementation of an ICU antimicrobial stewardship programme produced an optimization of antimicrobial therapy in 42.5 % of cases, mainly by reducing the antibacterial spectrum.
2. Use of carbapenems (IMP/MER) was significantly reduced (DDD difference of −17 % and −34 % respectively), confirming a tendency in growth in the susceptibility profile of *Pseudomonas aeruginosa*.
3. Overall, the optimization of all groups of antibiotics and antifungals leads to saving 19 % of the expenses.

### A944 Glycemic variability and mortality in elderly patients in medical intensive care unit

#### S. Tirumala, S.A. Pasha, B.K. Kumari

##### NRI Medical College and Hospital, Department of Critical Care, Guntur, India

###### **Correspondence:** S. Tirumala – NRI Medical College and Hospital, Department of Critical Care, Guntur, India

**Introduction:** Significant glucose variability affects the outcome of critically ill patients, but has not been well studied in the elderly patients in the ICU

**Objectives:** To study the impact of glycemic variability in elderly (age ≥60 years) in Indian setup

**Methods:** We conducted a retrospective analysis of prospectively collected data from 700 patients admitted to a 14 bedded medical ICU in 1000 bedded teaching hospital in south India over a period of 18 months. Patients whose blood glucose measured every 4 hours/day during their ICU stay were included. Patients whose stay is less than 48 hours and those who underwent surgical intervention were excluded. Measures of glycemic variability (Mean Blood Glucose (MBG) and Glycemic lability Index (GLI)) were calculated. Multivariate logistic regression analysis was performed on variables found to be significantly associated with mortality. Age (Exp(B) = 1.735) ; APACHE Score (Exp(B) = 1.298) and Glycemic lability index (Exp(B) = 1.001) were significantly associated with mortality.

The variables were subsequently compared beween the elderly patients (≥60 years) and those ≤ 60 years age

**Results:** Multivariate logistic regression analysis was performed on variables found to be significantly associated with mortality. Age (Exp(B) = 1.735) ; APACHE Score (Exp(B) = 1.298) and Glycemic lability index (Exp(B) = 1.001) were significantly associated with mortality.

Upon comparison of variables between elderly patients (≥60 years) and those ≤ 60 years age it was found that ICU LOS (7.22 ± 3.0 vs 6.57 ± 2.6 , P = 0.004), and GLI 116.20 ± 158.3vs 143.12 ± 159.85, P = 0.037 were significantly associated with mortality in elderly (66.42 ± 6.47 vs 36.34 ± 12.70, P = 0.000) whereas APACHE 11, MV days, Mean blood Glucose (MBG) were not

**Conclusions:** For the similar APACHE 2 score, elderly patients have greater glycemic variability , which could have contributed to the increased mortality. Amongst the glycemic variability indices tested, GLI was found to be better predictor than MBG.

**References**

1. Subhash Todi and Mahuya Bhattacharya Glycemic variability and outcome in critically ill Indian J Crit Care Med. 2014 May; 18(5): 285–290.

2. Krinsley JS. Glycemic variability: A strong independent predictor of mortality in critically ill patients. Crit Care Med. 2008; 36:3008–13.

### A945 Clinical characteristics and outcome of elderly patients (older than 80 years) admitted in an intensive care unit

#### P. Martinez-Lopez, A. Puerto-Morlán, P. Nuevo-Ortega

##### Hospital Universitario Virgen de la Victoria, Malaga, Spain

###### **Correspondence:** P. Martinez-Lopez – Hospital Universitario Virgen de la Victoria, Malaga, Spain

**Introduction:** The aging of the population has increased the demand for healthcare resources. The number of patients aged 80 years and older admitted to the intensive care unit (ICU) increased during the past decade. The aim of this study was to analyze characteristics and prognosis factors of very elderly patients admitted to an intensive care unit (ICU)

**Objectives:** To describe the characteristics and outcome of patients aged 80 or older who were admitted in the ICU of the Virgen de la Victoria Hospital of Malaga (a polyvalent ICU equipped with 18 beds

**Methods:** We performed a retrospective study in which we analysed data that were prospectively collected during 15 consecutive months. All patients ≥ 80 years who were admitted during this period were analysed. Patient´s outcome was followed until home discharge. The main variables collected were: Demographic data, diagnosis at the moment of admission, mortality predicted by APACHE II Score, life-sustaining treatments (LSTs), decision of limitations in LSTs, hospital length of stay, and in-hospital mortality rate.

**Results:** During this period 1474 patients were admitted in our intensive care unit, 131 of them, were ≥ 80 years ( 8,8 %) with a mean age of 82,74+/−2. 92 patients (70,2 %) were admitted after an emergency department admission, 15 (11,5 %) were previously hospitalized in a general ward, and 21 (16 %) were admitted after unscheduled surgery. Patients who had a medical or coronary pathology had better prognosis (only 11 % and 28 % of mortality) compared with post-operated patients who had a mortality of 55,5 % (p: 0,002). The mean of hospital length of stay was 9+/−15 days. Limitations in LSTs was decided in 19 (14,5 %) patients. We observed an in-hospital mortality rate of 27,5 % (36 patients). Multivariate analysis showed than the mechanical ventilation was an independent variable related with mortality (p < 0,05).

**Conclusions:** In our study we have observed that a total of 72 % of elderly patients admitted in our intensive care unit were alive after discharge from hospital, so that we think that there is a group of these patients who can be benefited of ICU admission.

**References**

1. Rummel NE, Balas MC, Morandi A, Ferrante LE, Gill TM, Ely EW. Understanding and reducing disability in older adults following critical illness. Crit Care Med. 2015 Jun;43(6):1265–75.

2. Ely EW, Evans GW, Haponik EF. Mechanical ventilation in a cohort of elderly patients admitted to an intensive care unit. Ann Intern Med. 1999 Jul 20;131(2):96–104.

### A946 Evolution of the number of admissions, age, scales of severity, length of stay and mortality in a general intensive care unit of a university hospital over 15 years

#### L. Martinez Pujol, R. Algarte Dolset, B. Sánchez González, S. Quintana Riera, J. Trenado Álvarez

##### Hospital Universitari Mutua Terrassa, Intensive Care Medicine, Terrassa, Spain

###### **Correspondence:** L. Martinez Pujol – Hospital Universitari Mutua Terrassa, Intensive Care Medicine, Terrassa, Spain

**Introduction:** Several reasons, such as technologic and scientific advances, are influencing in terms of health and quality of life worldwide, so life expectancy is longer. In the aftermath of this, in the last years, the number of admissions of elderly patients has remarkably increased in hospitals. This phenomenon is not indifferent to Intensive Care Units (ICUs).

A single database (GESPAC©) with homogeneous criteria has allowed to us to analize in our ICU the evolution of the admissions for the last 15 years.

**Objectives:** To describe the evolution of the admissions in the ICU of a University Hospital in a 15 years period (2000–2014), in terms of number of admissions, severity scores, ICU lenght of stay (LOS) and mortality, according to age groups.

**Methods:** A retrospective, single-center and descriptive study was conducted from 2000 to 2014. All patients admitted consecutively were included. Patients with ICU LOS less than 24 hours were excluded. They were divided into 3 periods of five years each one. Demographic data (age and gender) were collected. In each patient, scales of severity estimated by MPM0, MPM24, SAPS2 and APACHEIII were applied and registered, as well as lenght of stay and mortality at discharge of the ICU. The population was divided into 4 age groups according to age quartiles (<50 years, 50–64 years, 65–74 years and > 74 years) and the evolution of the number of admissions, severity scales, mortality and ICU LOS were analyzed per age groups and 5-year period.

**Results:** Qualitative variables are expressed as percentages and compared using the X2-test; quantitative ones are expressed as means and standard deviations (± S.D) or ranges, and they are analysed using Student´s t-test or ANOVA. Repeated Measures Analysis (MANOVA) were also used. The level of significance was placed at p < 0.05. The statistical analysis was performed using specific software ( IBM SPSS Statistics for Windows, Version 19.0. Armonk, NY: IBM Corp).

**Fig. 60 (abstract A946).**
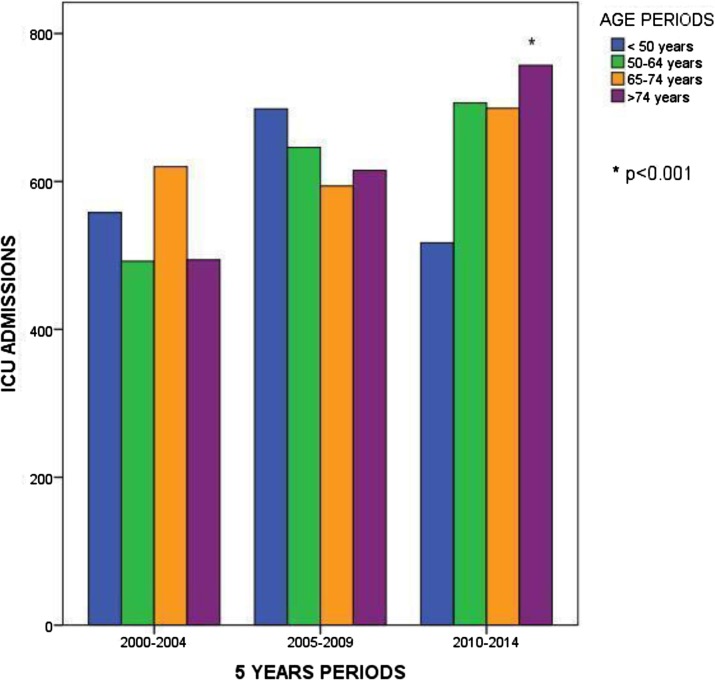
ᅟ

**Fig. 61 (abstract A946).**
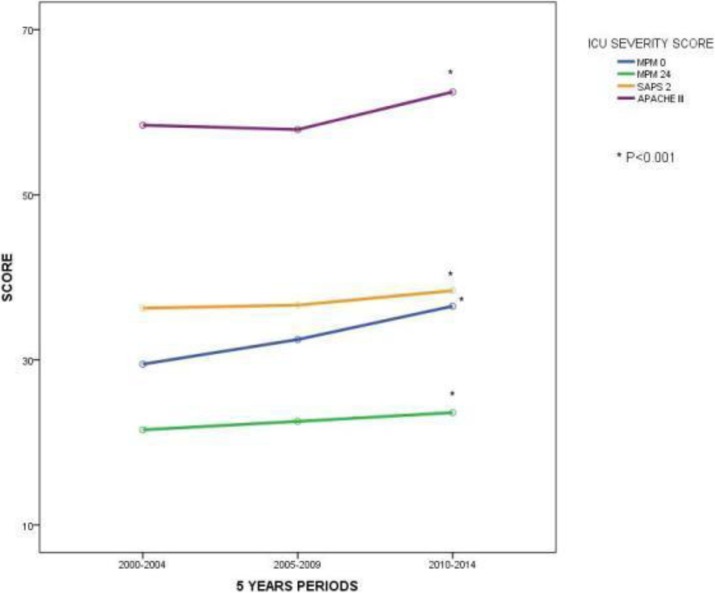
ᅟ

**Fig. 62 (abstract A946).**
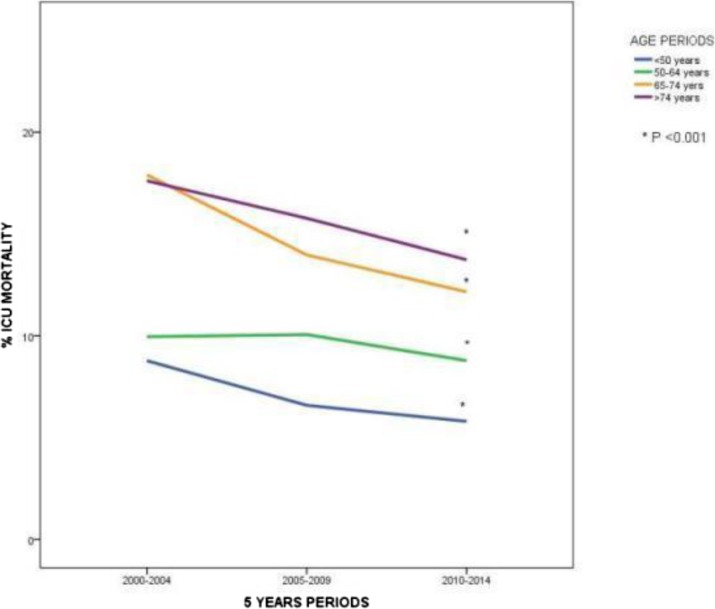
ᅟ

7396 patients were analyzed. The mean age was 61.6 years (SD 16.6). 67 % were men. The number of admissions per five-year period increased from 2164 in the first one to 2679 at the latest. Figure 60 shows a significant increase in admissions among the elder groups along the five-year periods. The severity scores increased significantly as shown in Figure 61 (p < 0.001). ICU lenght stay also decreased significantly (Table 36) along the different periods. Mortality decreased significantly in all age groups , especially in older, over periods Figure 62 (p < 0.001).

**Conclusions:** In our ICU, it has been an increase in the number of annual admisions and in the severity scores. However, ICU LOS and mortality have decreased. These results are particularly noteworthy in patients older than 75 years.

**Table 36 (abstract A946).** ᅟ

5 YEARS PERIOD	LOS ICU	LOS HOSPITAL
1ST PERIOD	7.2 (SD 10.1)	26.3 (SD 28.9)
2ND PERIOD	6.4 (SD 9.1)	27.6 (SD 35.3)
3RD PERIOD	6.1 (SD 9.6)	22.7 (SD 26.6)

### A947 Effectiveness of severity scales for age and type of patient in a polyvalent intensive care unit during the last 15 years

#### S. Quintana, L. Martínez, R. Algarte, B. Sánchez, J. Trenado

##### Hospital Universitari Mutua Terrassa, Critical Care Department, Terrassa, Spain

###### **Correspondence:** L. Martínez – Hospital Universitari Mutua Terrassa, Critical Care Department, Terrassa, Spain

**Introduction:** In our Intensive Care Medicine (ICU) department we used a database (GESPAC) with uniform and quality data for all admissions from 2000 to 2014, which allows us to study the evolution of severity scales and the clinical activity by age and type of patient.

**Objectives:** To describe the effectiveness of severity scales used in our ICU over 15 years by age and type of patient.

**Methods:** A retrospective, single-center and descriptive study was conducted from 2000 to 2014. All patients admitted consecutively were included. Patients with lenght of stay less than 24 hours were excluded.The severity scales we analyzed were MPM0, MPM24, SAPS2, APACHEIII. Patients were divided in 4 groups of age by quartiles (<50 years, 51–64 years, 65–74 years, > 74 years). The type of patient was classified in medical and urgent or scheduled surgery. We used descriptive statistics. Qualitative variables are expressed as percentages and quantitative variables are expressed as means and standard deviations (± SD) and ROC curves for the analysis of discrimination. We used SPSS v19.

**Fig. 63 (abstract A947).**
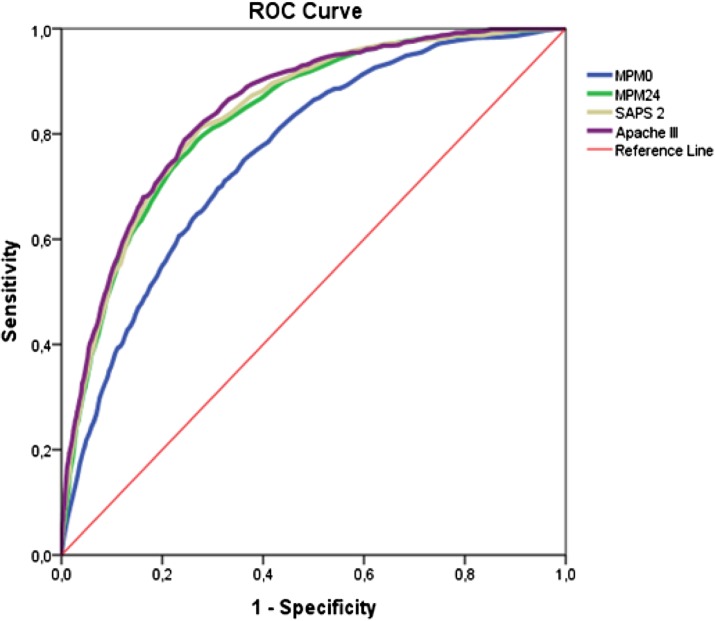
Curve ROC. Severity scales

**Results:** We included 7396 patients, 4955 were men (67 %), mean age was 61.6 years (SD 16.6). ICU mortality was 11.7 %. In Figure 63 we show 4 curve ROC corresponding to the 4 severity scales for all patients, MPM0 has a significantly worse discrimination respect to the other 3 scales. MPM 24, SAPS 2 and APACHE III have a similar behavior. In Table 37 we show the severity scales effectiveness by age groups. In Table 38 we show the severity scales effectiveness by type of patients.

**Conclusions:** We observed a decrease of effectiveness of severity scales over time, however this effectiveness remains optimal in all the severity scales except for MPM0.

**Table 37 (abstract A947).** Curve ROC.Severity scales for Age

	<51 years old	51–64 years old	65–74 years old	>74 years old
MPM0 AUC (95 % CI)	0.841 (0.805–0.877)	0.791 (0.757–0.825)	0.696 (0.663–0.729)	0.703 (0.670–0.736)
MPM24 AUC (95 % CI)	0.898 (0.870–0.926)	0.830 (0.798–0.861)	0.805 (0.779–0.831)	0.783 (0.753–0.813)
SAPS2 AUC (95 % CI)	0.869 (0.839–0.99)	0.849 (0.820–0.878)	0.813 (0.787–0.839)	0.807 (0.779–0.834)
APACHE III AUC (95 % CI)	0.877 (0.851–0.902)	0.846 (0.817–0.874)	0.841 (0.816–0.865)	0.813 (0.786–0.839)

**Table 38 (abstract A947).** Curve ROC.Severity scales type of patient

	MEDICAL	SCHEDULED SURGERY	URGENT SURGERY
MPM0. AUC (95 % CI)	0.761 (0.742–0.779)	0.755 (0.692–0.819)	0.718 (0.679–0.756)
MPM24 AUC (95 % CI)	0.826 (0.809–0.842)	0.888 (0.852–0.924)	0.814 (0.781–0.847)
SAPS2 AUC (95 % CI)	0.837 (0.821–0.853)	0.853 (0.806–0.900)	0.814 (0.781–0.846)
APACHE III AUC (95 % CI)	0.843 (0.827–0.858)	0.878 (0.840–0.915)	0.827 (0.796–0.858)

### A948 Family members´ satisfaction with care in an Angolan ICU

#### E. Tomas, N. Brock, E. Viegas, E. Filipe

##### Clinica Sagrada Esperança, Luanda, Angola

###### **Correspondence:** E. Tomas – Clinica Sagrada Esperança, Luanda, Angola

**Introduction:** In the intensive care unit (ICU), patient-centered care includes family-centered care. Since most ICU patients cannot make decisions for themselves, families are often involved as surrogate decision makers. Therefore, the perspectives of family is especially important in the critical care setting, and family satisfaction is an important outcome measure.

Objective: To determine satisfaction amongst relatives of patients on our ICU.

**Methods:** We distributed a modified version of an FS-ICU published in US and Canadian studies to up to two family members per patient. We only enrolled relatives of patients who had survived to ICU discharge. Four-point Likert scale responses were linearly transformed to give percentage scores. Higher values represented a greater degree of satisfaction. The study was conducted at Sagrada Esperança Clinic, a 250-bed tertiary care hospital. The hospital has 8 critical care beds.

**Results:** We received and analysed 79 completed surveys. Overall care in the ICU, 97 per cent. Courtesy, respect and compassion to the patient (99 %); symptom control such as pain, breathlessness and agitation (92 %, 93 %, 95 %, respectively); emotional support (97 %); ninety-eight per cent felt the care provided by doctors was very good or good, compared with 96 % by nurses and 95 % by physiotherapists.

**Conclusions:** Overall, most families in this study were satisfied with care provided to them and their critically ill relative; however, opportunities for improvement exist. Measuring and understanding satisfaction with care provided in ICUs will likely enhance the effectiveness of resources used in this critically ill patient population.

**References**

1) Heyland, et al. Crit Care Med 2002 Vol. 30, No. 7

2) Wall, *et al. Crit Care Med* 2007, 35: 271–279

**Fig. 64 (abstract A948).**
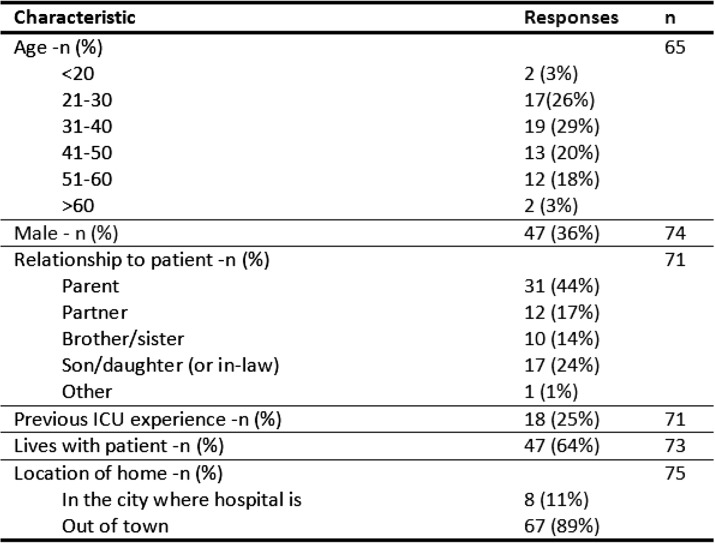
Demographic characteristics of respondents

**Fig. 65 (abstract A948).**
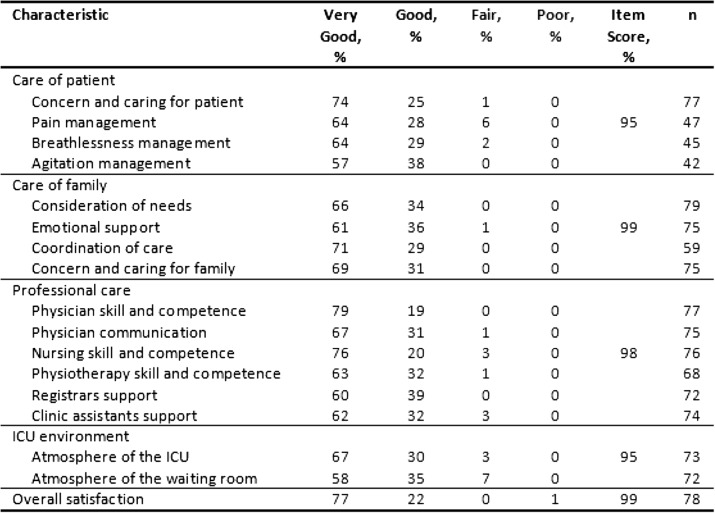
Responses to questionnaire: Family satisfaction wi

### A949 Evaluating documentation in an electronic patient record

#### D. Cottle, T. Traynor

##### Lancashire Teaching Hospitals Trust, Critical Care, Preston, United Kingdom

###### **Correspondence:** D. Cottle – Lancashire Teaching Hospitals Trust, Critical Care, Preston, United Kingdom

**Introduction:** We implemented a Critical Care EPR using the Quadramed system on the 30th Sept 2015. Our objective was to evaluate whether the EPR had improved the quality of our documention and the responsiveness of our notes.

**Methods:** We evaluated the patient record from 21 hospital days prior to the implentation of the EPR and 21 hospital days 6 months after using and refining the system.

**Results:** The proportion of completed nursing risk assessments did not change after implementation of the EPR. They depend on the user to shedule their completion. Safety checks for arterial and CVC lines were well established and changed little. There was an improvement in the percentage of shift checks completed when they were automatically sheduled.

The system provides a date, time and audit trail for each entry. The user traceability in the medical notes increased. The presence of the author´s name improved from 88 % to 100 %, the date from 96 % to 100 %, the time from 76 % to 100 %, and signature from 77 % to 100 %. Legibility improved from 89 % to 100 %. The proportion of entries with a contact number dropped from 23 % to 18 %.

The nursing care plans in the paper notes were better completed than the medical notes, but still improved. The presence of the nurse´s name increased from 97 % to 100 %, the date from 94 % to 100 %, the time from 95 % to 100 % and the signature from 95 to 100 %. Legibility was 100 % in both groups.

The Quadramed system provides automatic calculations of early warning scores and fluid balances.The more complicated the calculation, the greater the improvement.

Integration of data:

· The increase in data points that cross populate is: +350

· The allergy advice populates ALL the sheets compared to an average of 1 on paper (excluding the drug chart).

· The average number of scheduled events (that instruct staff to perform functions) has gone from 5 to 35.

**Conclusions:**

1. The largest improvement came in the accessibility of the notes. They can now be accessed within one minute from any PC in the Trust. Previously a standard time to deliver notes was two days, reducing to one day in an emergency.
2. Correct filing of the EPR notes and the search facility reduced the average time to complete the audit by 16 minutes per patient.
3. The user audit trail and traceability improved in both medical and nursing paperwork, more so in the former. This is explained by a baseline of lower documentation standards in the medical group.
4. The typed out notes are now legible.
5. There was a large improvement in the quality of data calculations that are now up to 100 %.
6. There was a large increase in the number of scheduled events, but this these only lead to an improvement in documentation when they were automatically scheduled by the computer. There was no improvement when user scheduling was required.

**Table 39 (abstract A949).** Change in the percentage of nursing tasks complete

Audit Outcome	Average for the paper notes	Average for the EPR	Difference
% of risk assessments completed	77	77	0
% of care plans completed	100	98	−2
% of arterial line assessments completed	95	100	+5
% of CVC lines completed	100	100	0
% of tracheostomy forms completed	100	100	0
% shift checks complete	81	97	+16

**Table 40 (abstract A949).** The change in times to access the notes

Audit outcome	Average time for paper notes	Average time for the EPR	Difference
Time to access the notes	Up to 1 day	1 min	Up to 24 hours
Time to audit the notes	48 min	32 min	16 min

**Table 41 (abstract A949).** The accuracy following EPR implementation

Audit outcome	Average for the paper notes	Average for the EPR	Difference
% Early warning scores correct	89	100	+11
% Fluid chart in correct	83	100	+17
% Fluid chart out correct	100	100	0
% Total balance correct	67	100	+23

### A950 E-ICU: are new technologies a useful tool to take care for the family of the critically ill patients?

#### M.V. Trasmonte Martínez^1^, M. Pérez Márquez^1,2^, L. Colino Gómez^1^, N. Arias Martínez^1^, J.M. Milicua Muñoz^1,2^, B. Quesada Bellver^1,2^, M. Muñoz Varea^1^, M.Á. Alcalá Llorente^1^, C. Pérez Calvo^1^

##### ^1^Fundación Jiménez Díaz, Madrid, Spain; ^2^Hospital Rey Juan Carlos, Madrid, Spain

###### **Correspondence:** M.V. Trasmonte Martínez – Fundación Jiménez Díaz, Madrid, Spain

**Introduction:** the quality of communication with family members in ICUs is a focus of interest for clinical care improvement.[1] Relatives of patients admitted experience stressful situations where timely honest support provided can be vital to the phychosocial health and satisfaction.[2] We stablished an electronic communication portal (ECP) to supplement daily evolution provided by physicians as well as telephonic information related to developments in clinical status, and to inform about the request of therapeutic/diagnostic procedures.

**Objectives:** to describe the level of satisfaction of the family members regarding the implementation of the ECP and to highlight areas of improvement.

**Methods:** we surveyed 41 family members of adult patients admitted to ICU during the first 8 months after the implementation of the electronic portal. We adapted the Family Satisfaction Intensive Care survey (FS-ICU 34). Selection criteria are described in Table 42.

**Results:** 34 relatives (67,6 % females) took part (44 % son/daughter, 35 % husband/wife) . Severity scores APACHE II 17 +/− 8,6, SAPS II 38,5+/−16,6. Average stay 21,2+/−18,4 days. Mortality 20 %.

We noticed very high levels of satisfaction regarding the professional care (frequency of communication, physician skill and competence, understanding information, honesty and facilities of getting information) and overall with care. Satisfaction was even higher when we considered the usefulness of the ECP. Every respondents supported it as a complement to daily information but it was only supposed to replace verbal information in 11,8 %.

17 % did not access the website because of sufficient verbal information or cultural or age-related difficulties. The access was mostly via computer (47,1 %) followed by smarthphone (23,5 %).

Particularly desired were daily updates, an established timetable and more detailed information. There were no statistically significant differences in the need of web access among families living near the hospital and not or prior experience with ICU familiar admission.

**Conclusions:** ECP appears to decrease the level of anxiety of families, improves perceived quality and can help to combine patient care with their work and personal responsibilities without replacing the daily evolution provided by physicians.

**References**

1. Brown SM, Bell SK, Roche SD et al. Preferences of Current and Potential Patients and Family Members Regarding Implementation of Electronic Comunication Portalls in Intensive Care Units. Ann Am Thorac Soc. 2016 Mar;13(3):391–400.

2. Kirchhoff KT, Song MK. Caring for the family of the critically ill patient. Crit Care Clin. 2004 Jul;20(3):453–66.

**Table 42 (abstract A950).** Family selection criteria

Absence of advance directives	
ICU complex process: − Prolonged stay - Clinical course with probability of severe complications - Complex therapeutic/diagnostic procedures - Possibility of functional/organic sequelae - Possibility of LET and/or exitus	
Internet knowledge	
Informed consent	
Structured family	

### A951 Withdrawn

### A952 Radiological review of central line placement in intensive care unit patients

#### H. Hendra, N. Lawrence

##### Broomfield Hospital, Intensive Care Unit, Chelmsford, United Kingdom

###### **Correspondence:** H. Hendra – Broomfield Hospital, Intensive Care Unit, Chelmsford, United Kingdom

**Introduction:** Correct placement of central venous catheter (CVC) as an essential venous access in most ITU patients can be confirmed by chest radiograph. The carina has been increasingly recognised as a safe radiological landmark to ensure tip placement outside the right atrium. Studies suggest that right-sided central line tip should be located above the carina while left-sided catheter tip be sited below the carina.^1^ An angle of CVC tip to superior vena cava (SVC) wall > 40° has been associated with higher risk of eroding through the vessel wall.

**Objectives:** Our aim was to investigate the position of central line tips in relation to the carina in patients admitted to our ITU as well as assessing complications which occurred as a result of tip misplacement.

**Methods:** A total of 77 patients admitted to ITU in November 2015 were analysed retrospectively. Patients with no central line and femoral line were excluded from the study; 55 patients with internal jugular (IJ) line were studied further. Vertical distances between catheter tip and carina as well as the angle of CVC tip to vertical were calculated.

**Fig. 66 (abstract A952).**
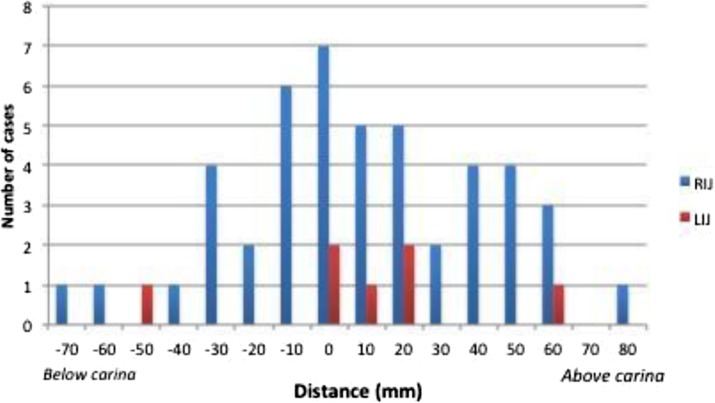
Distance between line tip and carina

**Results:** Out of 55 internal jugular CVC, 48 lines (87 %) were inserted on the right side and 7 (13 %) were located on the left side. CXR was not done in 2 cases. Documentation of post-procedure CXR review by the team was found in 14/53 cases (26 %) and confirmation of line position was documented in 12/53 cases (23 %). Summary of distance measured between line tip and carina can be found in the graph below (Fig. 66).

Among cases with distance > 30 mm above or below the carina, correction of placement only occurred in 1 case. While none of the tips were at steep angle (>40°) to the vertical for the right-sided catheter, 3 cases (43 %) from the left-sided placement had this issue. No intervention was done to correct the position. Length of catheter inserted was only documented in 1/7 patients with left IJ catheter placement. Complications in our cohort included an apical pneumothorax (2 %) and a leak (2 %).

**Conclusions:** Our audit highlighted important issues such as poor documentation of post-procedure CXR review as well as minimal effort in correcting the CVC placement despite evidence of suboptimal position. A high percentage (43 %) of left-sided catheter cohorts had acute angle > 40° between tip & vertical and unfortunately the documentation of the catheter length used is relatively poor; therefore the cause of this remains uncertain. We have introduced a mandatory system for documentation, confirmation of CXR review & actions taken post review as well as teaching sessions for CVC insertion & optimal line position to address these issues. A re-audit will be done after full implementation of these.

**References**

1. Stonelake P.A., et al. The carina as a radiological landmark for central venous catheter tip position. British Journal of Anaesthesia 96(3): 335–40, 2006

## OUTCOME PREDICTION OF POST-CARDIAC ARREST PATIENTS

### 0953 Impact of age on the relationship between resuscitation duration and neurologically intact survival after out-of-hospital cardiac arrest

#### K. Maekawa, M. Hayakawa, Y. Ono, A. Kodate, Y. Sadamoto, N. Tominaga, A. Mizugaki, H. Murakami, T. Yoshida, K. Katabami, T. Wada, A. Sawamura, S. Gando

##### Hokkaido University Hospital, Emergency and Critical Care Center, Sapporo, Japan

###### **Correspondence:** K. Maekawa – Hokkaido University Hospital, Emergency and Critical Care Center, Sapporo, Japan

**Introduction:** Recent studies suggest that prolonged cardiopulmonary resuscitation (CPR) might be beneficial for some patients with cardiac arrest. In practice, children and young adults are often received longer resuscitation efforts than elderly patients, however, the impact of age on the relationship between CPR duration and outcome is unknown.

**Objectives:** To estimate the impact of age on the relationship between CPR duration and neurological outcome among patients with out-of-hospital cardiac arrest (OHCA) in Japan.

**Methods:** We performed retrospective analysis using data from Japan's nationwide OHCA registry, which includes all patients with OHCA transported to the hospital by emergency medical services from January 2005 through December 2012. Patients were stratified into 3 groups: children and adolescents (0–17 yr), adults (18–65 yr) and elderly patients (>66 yr). CPR duration was calculated as time interval from any resuscitation effort start to return of spontaneous circulation (ROSC). We used multivariable logistic regression models to assess the relationship of CPR duration on neurologically intact survival at 1 month after cardiac arrest.

**Results:** Of the eligible 62208 patients with OHCA and ROSC before hospital arrival, 1175 were enrolled in children and adolescents, 20696 in adults and 40337 in elderly patients. Adjusted probabilities of intact survival were 42.2 % for 0–9 min of CPR duration, 25.8 % for 10–19 min, 9.8 % for 20–29 min and 3.2 % for > 30 min in all age groups. Compared with adults, children and adolescents had higher odds and elderly patients had lower odds of intact survival through entire CPR duration. Adjusted odds of intact survival were 2.6 (95%CI, 2.1-3.1) for 0–9 min, 2.1 (1.6-2.7) for 10–19 min, 1.6 (0.9-2.8) for 20–29 min and 2.4 (1.1-5.3) for > 30 min in children and adolescents. Similarly, adjusted odds were 0.6 (0.6-0.7), 0.5 (0.4-0.5), 0.5 (0.4-0.5) and 0.4 (0.3-0.5) in elderly patients.

**Conclusions:** Age affected the relationship between CPR duration and neurological outcome. Even after prolonged resuscitation efforts > 30 min, children and adolescents with OHCA could achieve successful outcome compared with adult and elderly patients.

**References**

1. Goldberger ZD, Chan PS, Berg RA, et al. Duration of resuscitation efforts and survival after in-hospital cardiac arrest: an observational study. Lancet. 2012;27;380:1473–81.

2. Matos RI, Watson RS, Nadkarni VM, et al. Duration of cardiopulmonary resuscitation and illness category impact survival and neurologic outcomes for in-hospital pediatric cardiac arrests. Circulation. 2013;127:442–51.

3. Nagao K, Nonogi H, Yonemoto N, et al. Duration of Prehospital Resuscitation Efforts After Out-of-Hospital Cardiac Arrest. Circulation. 2016;133:1386–96.

### A954 Cortical and subcortical grey matter morphometry for neuroprognostication after cardiac arrest

#### S. Silva^1,2^, L. Kerhuel^3^, B. Malagurski^2^, G. Citerio^4^, R. Chabanne^5^, S. Laureys^6^, L. Puybasset^7^

##### ^1^University Teaching Hospital of Purpan, Critical Care Medicine, Toulouse, France; ^2^INSERM 1214, Toulouse NeuroImaging Center (TONIC), Toulouse, France; ^3^University Teaching Hospital of Purpan, Toulouse, France; ^4^School of Medicine and Surgery, University Milano Bicocca and Hospital San Gerardo, Department of Anaesthesiology and Critical Care, Monza, Italy; ^5^University Hospital of Clermont-Ferrand, Critical Care Unit, Clermont-Ferrand, France; ^6^University Hospital and University of Liège, Cyclotron Research Center and Department of Neurology, Liège, Belgium; ^7^Groupe Hospitalier Pitié-Salpétrière, APHP, Critical Care and Anaesthesiology Department, Paris, France

###### **Correspondence:** S. Silva – University Teaching Hospital of Purpan, Critical Care Medicine, Toulouse, France

**Introduction:** Neuroimaging shows promise for determining early prognosis after cardiac arrest (CA). Nevertheless, conventional MRI sequences, as T1-weightened sequences, are currently considered not precise enough to detect brain structural anomalies in this context, and therefore are supposed to be unable to accurately predict outcome^1^.

**Objectives:** We hypothesize that the combined use of cortical thickness measurement and subcortical grey matter volumetry could provide an early and accurate in vivo assessment of the structural impact of cardiac arrest (CA), and therefore could be used for long-term neuroprognostication in this setting.

**Fig. 67 (abstract A954).**
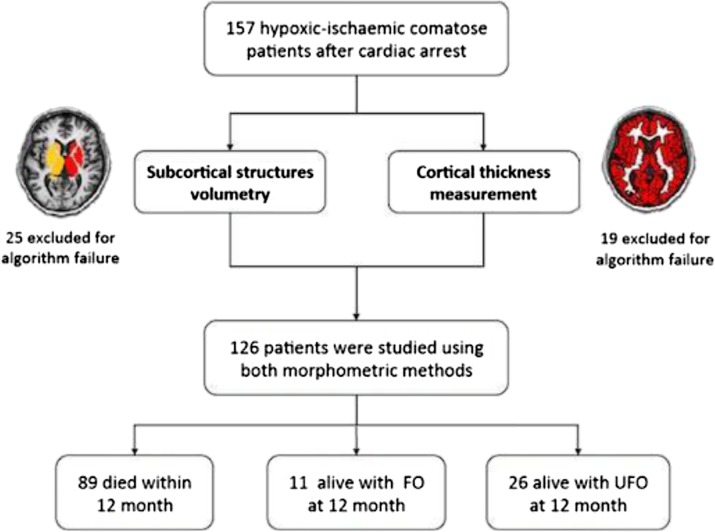
Study flowchart

**Methods:** Prospective study undertaken in five Intensive Critical Care Units affiliated to the University in Toulouse (France), Paris (France), Clermont-Ferrand (France), Liège (Belgium) and Monza (Italy). High-resolution anatomical T1-weighted images were acquired in 157 anoxic coma patients (16 +/− 8 days after CA) and 70 matched controls. Patients were followed up one year after CA. Cortical thickness was computed on the whole cortical ribbon and deep grey matter volumetry was performed after automatic segmentation^2^. Brain morphometric data was employed to create multivariate predictive models using learning machine techniques (Figure 67).

**Fig. 68 (abstract A954).**
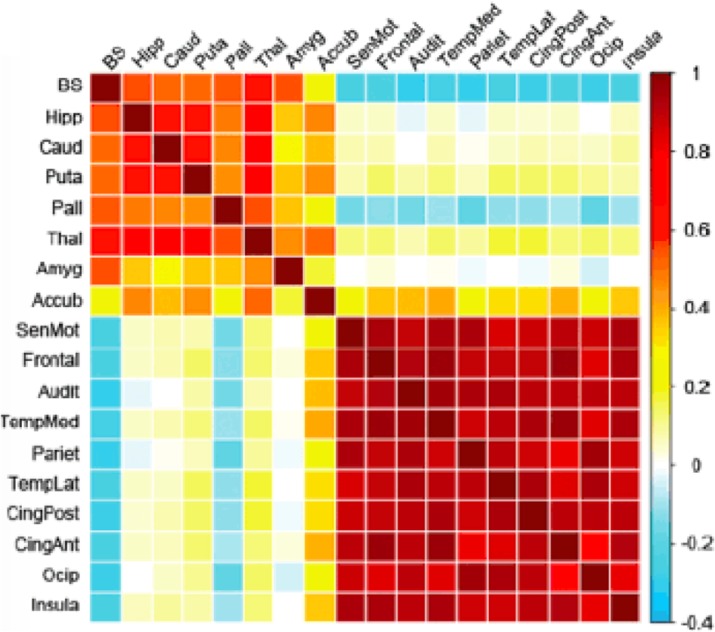
Whole-brain correlation matrix

**Fig. 69 (abstract A954).**
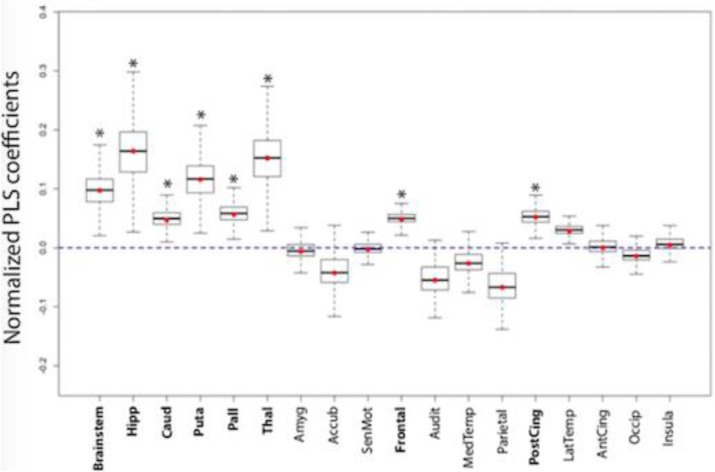
Normalized PLS coefficients

**Results:** Patients displayed significantly extensive cortical and subcortical brain volumes atrophy compared to controls. A dissociated vulnerability to anoxic insult was observed: subcortical volumes were related to CA duration and cortical thickness values were linked to the time to MRI acquisition (Figure 68) The accuracy of a predictive classifier, encompassing cortical and subcortical components has a significant discriminative power (AUC = 0.87). The anatomical regions which volume changes were significantly related to patient's outcome were: frontal cortex, posterior cingulate cortex, thalamus, putamen, pallidum, caudate, hippocampus and brainstem (Figure 69)

**Conclusions:** These findings are consistent with the hypothesis of pathological disconnection within a striatopallidal-thalamo-cortical mesocircuit induced by CA^4^ and pave the way for the use of combined brain quantitative morphometry in this setting.

**References**

1. Young GB. Clinical practice. Neurologic prognosis after cardiac arrest. N Engl J Med. 2009;361(6):605–611.

2. Peran P, Cherubini A, Assogna F, et al. Magnetic resonance imaging markers of Parkinson´s disease nigrostriatal signature. Brain. 2010;133(11):3423–3433.

3. Silva S, de Pasquale F, Vuillaume C, et al. Disruption of posteromedial large-scale neural communication predicts recovery from coma. Neurology. 2015;85(23):2036–2044

**Grant acknowledgment**

University Teaching Hospital of Toulouse, James McDonnell Foundation, the Belgian American Education Foundation, University Milano Bicocca.

### A955 Clinical and electrophysiological correlates of absent somatosensory evoked potentials after post-anoxic brain damage: a multicentre cohort study

#### L. Nobile^1^, E.R. Pognuz^2^, A.O. Rossetti^3^, F. Verginella^2^, N. Gaspard^4^, J. Creteur^1^, N. Ben-Hamouda^5^, M. Oddo^5^, F.S. Taccone^1^

##### ^1^Hôpital Erasme, Université Libre de Bruxelles, Department of Intensive Care, Brussels, Belgium; ^2^Ospedali Riuniti, Department of Perioperative Medicine, Trieste, Italy; ^3^CHUV, Department of Neurology, Lausanne, Switzerland; ^4^Hopital Erasme, Université Libre de Bruxelles, Department of Neurology, Brussels, Belgium; ^5^CHUV, Department of Intensive Care, Lausanne, Switzerland

###### **Correspondence:** L. Nobile – Hôpital Erasme, Université Libre de Bruxelles, Department of Intensive Care, Brussels, Belgium

**Introduction:** Multimodal assessment is recommended for prognostication of coma after cardiac arrest (CA). Bilateral absence of cortical response (N20) to somatosensory evoked potentials (SSEPs) has very high predictive value for poor prognosis, however the exact correlates of absent N20 with other clinical, electrophysiological and biochemical outcome prognosticators are unclear.

Objective: The aim of this study was to evaluate clinical and electrophysiological characteristics of patients with absent N20.

**Methods:** Retrospective analysis of institutional databases (2007–2014) in three academic ICUs including all adult comatose CA patients treated with targeted temperature management (TTM, for 24 hours) who underwent multimodal assessment including SSEPs (48–72 hours after CA) as part of standard care. Additional data included clinical examination (absence of pupillary reflexes, absent or posturing motor response and myoclonus on day 2–3), EEG (absence of reactivity to painful stimuli; presence of a malignant pattern, such as burst-suppression or suppressed background; seizures or status epilepticus) during the first 48 hours and peak NSE levels over the first 72 hours from CA. Neurological outcome was assessed at 3 months using the Cerebral Performance Categories (3–5 = poor outcome; 1–2 = good outcome).

**Results:** A total of 532 patients with SSEPs data were analyzed, of whom 143 (27 %) had bilateral absent N20 (N20_ABS_); 198 (37 %) patients had a good outcome and all had present N20 (N20+). Median time to SSEPs was 72 [IQRs: 48–72] hours after arrest. In N20_ABS_ patients, the occurrence of absent pupillary reflexes (74 % vs. 17 % in N20+; p < 0.001), absent or extension motor response (94 % vs. 38 %; p < 0.001), myoclonus (38 % vs. 8 %; p < 0.001) malignant EEG patterns (51 % vs. 21 %; p < 0.001) and non-reactive EEG (90 % vs. 32 %; p < 0.001) was more frequent than others; also higher NSE (77.2 [37.5-120.9] - n = 64 vs. 21.0 mcg/L [14.9-32.7] - n = 238; p < 0.001] was found in N20_ABS_ patients. N20_ABS_ was concomitantly associated with at least one EEG finding (non-reactive background, seizures or malignant pattern) and one clinical sign (bilateral absence of pupillary reflexes or myoclonus) of poor prognosis in 112/143 (78 % vs. 58/389 (15 %) in N20+, p < 0.001).

**Conclusions:** Our data confirm that bilateral absence of N20 reflects severe post-anoxic cerebral damage and therefore frequently correlates with concordant clinical and EEG signs of poor outcome. However, our study also identified a subset of patients with discordant signs, in whom clinical examination and/or EEG were reactive despite bilaterally absent N20. Our findings raise further questions on outcome prognostication after CA and underline the importance of multimodal assessment in this setting.

**References**

1. Sandroni C, Geocoding RG Neurological prognostication after cardiac arrest. Curr Opin Crit Care. 2015;2:209–14

### A956 The response time threshold for predicting favorable neurological outcomes in patients with bystander-witnessed out-of-hospital cardiac arrest

#### Y. Ono^1^, M. Hayakawa^1^, H. Iijima^2^, K. Maekawa^1^, A. Kodate^1^, Y. Sadamoto^1^, A. Mizugaki^1^, H. Murakami^1^, K. Katabami^1^, T. Wada^1^, A. Sawamura^1^, S. Gando^1^

##### ^1^Hokkaido University Hospital, Emergency and Critical Care Medicine, Sapporo, Japan; ^2^Hokkaido University Hospital, Clinical Research and Medical Innovation Center, Sapporo, Japan

###### **Correspondence:** Y. Ono – Hokkaido University Hospital, Emergency and Critical Care Medicine, Sapporo, Japan

**Introduction:** It is well established that the period of time between when a call in made to emergency medical service (EMS) to the point when EMS arrive at the scene (i.e., the response time) affects the survival outcomes in out-of-hospital cardiac arrest (OHCA) patients. However, the relationship between response time and favorable neurological outcomes remains unclear. We therefore aimed to determine a response time threshold in bystander-witnessed OHCA patients that is associated with positive neurological outcomes and to assess the relationship between the neurological outcomes and response time in OHCA patient.

**Methods:** This study was a retrospective, observational analysis of data from 204,277 episodes of bystander-witnessed OHCA between 2006 and 2012 in Japan. We used classification and regression trees (CARTs) and receiver operating characteristic (ROC) curve analysis to determine the threshold of response time associated with favorable neurological outcomes (Cerebral Performance Category 1 or 2) one month after cardiac arrest.

**Results:** Both CARTs and ROC analyses indicated that a threshold of 6.5 min was associated with improved neurological outcomes in all bystander-witnessed OHCA events from cardiac origin. Furthermore, bystander cardiopulmonary resuscitation (CPR) prolonged the threshold of response time by 1 min (to 7.5 min). The adjusted odds ratios for favorable neurological outcomes in OHCA patients who received care within ≤ 6.5 min was 1.935 (95 % confidential interval: 1.834-2.041, P < 0.001).

**Conclusions:** A response time ≤ 6.5 min was closely associated with favorable neurological outcomes in all bystander-witnessed OHCA patients. Bystander CPR prolonged the response time threshold by 1 min.

**References**

1. Eisenberg MS, Bergner L, Hallstrom A. Cardiac resuscitation in the community. Importance of rapid provision and implications for program planning. *JAMA*. 1979;241:1905–1907. doi: 10.1001/jama.1979.03290440027022.

2. Mullie A, Van Hoeyweghen R, Quets A. Influence of time intervals on outcome of CPR. The Cerebral Resuscitation Study Group. *Resuscitation*. 1989;17:S23-33. doi: 10.1016/0300-9572(89)90088-9.

3. Chen TT, Ma MH, Chen FJ, Hu FC, Lu YC, Chiang WC, Ko PC. The relationship between survival after out-of-hospital cardiac arrest and process measures for emergency medical service ambulance team performance. *Resuscitation*. 2015;97:55–60. doi: 10.1016/j-resuscitation. 2015.04.035.

4. Sladjana A, Gordana P, Ana S. Emergency response time after out-of-hospital cardiac arrest. *Eur J Intern Med*. 2011;22:386–393. doi: 10.1016/j.ejim.2011.04.003.

**Grant acknowledgment**

None

### A957 Disseminated intravascular coagulation with the fibrinolytic phenotype predicts the outcome of patients with out-of-hospital cardiac arrest

#### A. Kodate, K. Katabami, T. Wada, Y. Ono, K. Maekawa, M. Hayakawa, A. Sawamura, S. Gando

##### Hokkaido University Graduate School of Medicine, Division of Acute and Critical Care Medicine, Sapporo, Japan

###### **Correspondence:** A. Kodate – Hokkaido University Graduate School of Medicine, Division of Acute and Critical Care Medicine, Sapporo, Japan

**Background:** We tested the hypothesis that disseminated intravascular coagulation (DIC) with the fibrinolytic phenotype during the early phase of post-cardiopulmonary resuscitation (CPR) causes systemic inflammatory response syndrome (SIRS), multiple organ dysfunction syndrome (MODS) and affects the outcome of out-of-hospital cardiac arrest (OHCA) patients.

**Methods:** Patients with established out-of-hospital cardiac arrest (OHCA) who underwent cardiopulmonary resuscitation with subsequent return of spontaneous circulation were retrospectively enrolled. Two hundred and eight DIC patients diagnosed by the Japanese Association for Acute Medicine (JAAM) DIC criteria were divided into two subgroups with hyperfibrinolysis (73) and without hyperfibrinolysis (135). The definition of hyperfibrinolysis was made by a FDP level > 100 μg/mL. Platelet count, global markers of coagulation and fibrinolysis were measured 4 times after admission to emergency department (T1, 0–6; T2, 6–12; T3, 12–18; T4, 18–24 hr). The outcome measure was the hospital all-cause mortality.

**Results:** Patients with hyperfibrinolysis had higher DIC, SIRS, and sequential organ failure assessment (SOFA) scores associated with higher prevalence of MODS, leading to a higher mortality rate of 68.5 % in comparison to patients without hyperfibrinolysis (41.7 %). Stepwise logistic regression analyses confirmed that DIC, SOFA scores, and lactate levels are independent predictors of patient death. Hyperfibrinolysis also predicted patient death. Tissue hypoperfusion (as indicated by lactate level) is a main determinant of hyperfibrinolysis. Receiver operating characteristic curves showed a significant discriminative performance of DIC scores for patient death. Kaplan-Meier curves showed that DIC, especially DIC with hyperfibrinolysis, significantly affected patient death.

**Conclusions:** DIC with the fibrinolytic phenotype during the early phase of post-CPR more frequently results in SIRS and MODS, and affects the outcome of OHCA patients. Hypoxia/ischemia during cardiac arrest and CPR are considered to be the cause of increased fibrin(ogen)olysis.

### A958 The association between tracheal intubation during pediatric in-hospital cardiac arrest and survival

#### L.W. Andersen^1,2^, T. Raymond^3^, R. Berg^4^, V. Nadkarni^4^, A. Grossestreuer^5^, T. Kurth^6^, M. Donnino^2,7^

##### ^1^Aarhus University Hospital, Anesthesiology, Aarhus, Denmark; ^2^Beth Israel Deaconess Medical Center, Harvard Medical School, Emergency Medicine, Boston, United States; ^3^Medical City Children's Hospital, Dallas, United States; ^4^The Children's Hospital of Philadelphia, Philadelphia, United States; ^5^University of Pennsylvania, Philadelphia, United States; ^6^Institute of Public Health, Charité - Universitätsmedizin Berlin, Berlin, Germany; ^7^Beth Israel Deaconess Medical Center, Harvard Medical School, Critical Care, Boston, United States

###### **Correspondence:** L.W. Andersen – Aarhus University Hospital, Anesthesiology, Aarhus, Denmark

**Introduction:** Tracheal intubation is common during pediatric in-hospital cardiac arrest, although the relationship between intubation during cardiac arrest and outcomes is unknown.

Objective: To determine if intubation during pediatric in-hospital cardiac arrest is associated with improved outcomes.

**Methods:** This was an observational study of prospectively collected data from United States hospitals participating in the Get With the Guidelines - Resuscitation registry. We included pediatric patients (age < 18 years) with index in-hospital cardiac arrest. We excluded patients who were receiving invasive mechanical ventilation and/or had an invasive airway in place at the time chest compressions were initiated. The exposure was tracheal intubation during the cardiac arrest. The primary outcome was survival to hospital discharge. Secondary outcomes included return of spontaneous circulation and neurological outcome. A favorable neurological outcome was defined as a score of 1–2 on the pediatric cerebral performance category score. Patients being intubated at any given minute (from 0 to 15 minutes) were matched with patients at risk of being intubated within the same minute (i.e. still receiving resuscitation) based on a time-dependent propensity score calculated from multiple patient, event, and hospital characteristics. Modified Poisson regression with adjustment for matching and clustering were then performed to obtain risk ratios.

**Results:** 2294 patients were included. Of these, 1555 (68 %) were intubated during the cardiac arrest. In the time-dependent propensity score-matched cohort (n = 2270), survival was lower in those intubated compared to those not intubated during cardiac arrest: 411/1135 (36 %) vs. 460/1135 (41 %), risk ratio: 0.89 (95%CI: 0.81, 0.99), p = 0.03. There was no significant difference in return of spontaneous circulation (770/1135 [68 %] vs. 771/1135 [68 %], risk ratio: 1.00 [95%CI: 0.95, 1.06], p = 0.96) or favorable neurologic outcome (185/987 [19 %] vs. 211/983 [21 %], risk ratio: 0.87 [95 %: 0.75, 1.02], p = 0.08) between those intubated and not intubated during cardiac arrest. The association between intubation and decreased survival remained in the majority of our sensitivity and subgroup analyses

**Conclusions:** Tracheal intubation during in-hospital pediatric cardiac arrest was associated with decreased survival to hospital discharge. These findings challenge the present resuscitation paradigm for pediatric in-hospital cardiac arrest.

**Fig. 70 (abstract A958).**
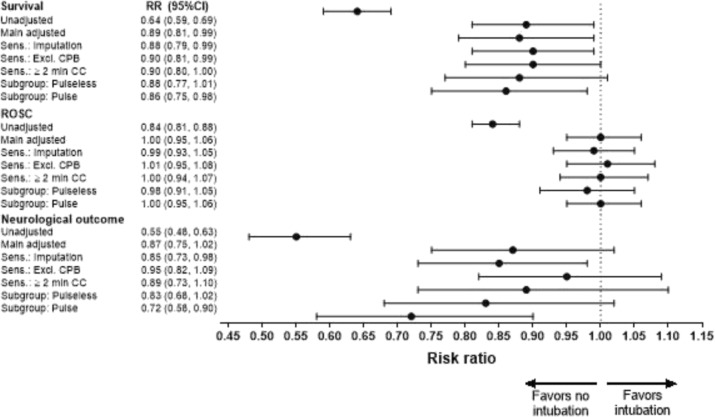
Forest plot

### A959 Prediction of mortality in the patients with refractory cardiac arrest and extracorporeal life support

#### A. Krüger, P. Ostadal, M. Janotka, D. Vondrakova

##### Homolka Hospital, Cardiology, Prague, Czech Republic

###### **Correspondence:** A. Krüger – Homolka Hospital, Cardiology, Prague, Czech Republic

**Introduction:** Substantial proportion of patients who suffered cardiac arrest do not respond to conventional cardiopulmonary resuscitation. Recently, extracorporeal cardiopulmonary resuscitation (ECPR) has been introduced as a potentially life-saving procedure in refractory cardiac arrest.

Objectives. The aim of our study was to evaluate the relation between ECPR survival, lactate levels and blood pH.

**Methods:** Eligible patients for this analysis had to undergo ECPR after at least ten minutes of unsuccessful cardiopulmonary resuscitation with a minimum of three defibrillation attempts. For extracorporeal life support (ECLS) we used Cardiohelp system (Maquet, Germany) or Levitronix CentriMag blood pump (Levitronix, USA). LUCAS II system (PhysioControl, Sweden) was used for chest compressions during ECLS insertion and cannulas were placed with percutaneous puncture under fluoroscopy or ultrasound control. Blood lactate and pH levels measured before ECLS insertion and after 24 hours were used for this study.

**Results:** We analyzed data from 29 patients treated with ECPR for refractory cardiac arrest. The mean age of our patients was 57 years (31–81). Out-of-hospital cardiac arrest occurred in 16 patients, 13 patients suffered from in-hospital arrest. Thirty-day mortality in our group was 57 % and 32 % of patients recovered with good neurological outcome. Percutaneous coronary intervention was performed in 18 (67 %) patients. Baseline value of lactate was 11.52 ± 5.42 mmol/l, initial pH 6.97 ± 0.21. In comparison with survivors, patients who died had significantly higher initial lactate levels (15.05 ± 1.56 vs. 10.01 ± 1.03; P < 0.05) and lower baseline pH (6.87 ± 0.06 vs 7.04 ± 0.04; P < 0.05). Moreover, survivors had significantly lower lactate levels after 24 hours.

**Conclusions:** ECPR represents virtually the last chance to survive refractory cardiac arrest. The levels of blood lactate and pH are significantly associated with clinical outcomes of ECPR.

**Grant acknowledgment**

The study was supported by an Institutional grant MH CZ - DRO (Nemocnice Na Homolce - NNH, 00023884)

### A960 Neurological prognostication in comatose survivors from cardiac arrest treated with therapeutic hypothermia

#### N. Kongpolprom^1,2^, J. Cholkraisuwat^2,3^

##### ^1^Chulalongkorn University, Pulmonary and Critical Care Medicine, Bangkok, Thailand; ^2^King Chulalongkorn Memmorial Hospital, Bangkok, Thailand; ^3^Chulalongkorn University, Medicine, Bangkok, Thailand

###### **Correspondence:** N. Kongpolprom – Chulalongkorn University, Pulmonary and Critical Care Medicine, Bangkok, Thailand

**Introduction:** Post-cardiac arrest survivors treated with therapeutic hypothermia (TH) remain comatose after rewarming. In contrast to survivors without TH, neurological prognostication is imprecise due to a persistent sedative effect[1].

**Objectives:** We aimed to evaluate clinical signs and findings that could predict neurological recovery and determined the optimal time for prognosis.

**Methods:** We retrospectively reviewed database of 51 post-arrest patients treated with TH in our hospital from 2006 to 2014. Cerebral performance category (CPC), neurological signs and findings in EEG and brain CT were evaluated. Neurological recovery was scored as favorable neurological outcome, namely normal cerebral function(CPC1) and moderate disability(CPC2) or unfavorable neurological outcome, namely severe disability(CPC3), vegetative state(CPC4) and death(CPC5). Neurological signs and findings in EEG and brain CT, which possibly predicted neurological recovery, and the optimal time to evaluate neurological status were analyzed.

**Results:** TH was performed in 51 post-arrest patients. Approximately 53 % (27/51) of TH-patients survived and 33 % of the survivors had favorable neurological outcome. Findings predicting unfavorable outcome at discharge were lack of pupillary response and/or Gag reflex after rewarming, and the absence of at least one of the brainstem reflexes, no eye opening or motor response worse than pain withdrawal (M ≤ 3) on the seventh day. (Table 43) Myoclonus and seizure could not be used to indicate poor prognosis. One of 17 survivors with myoclonus had full recovery and 50 % of the survivors with seizures regained consciousness upon discharge. Findings of EEG and brain CT showed that the patients with burst-suppression EEG pattern or brain swelling became vegetative or died, but the prognostic values of these findings were inconclusive.

**Conclusions:** Our study showed that the simple neurological signs helped predict short-term neurological prognosis of comatose survivors undergoing TH. The most reliable signs which determined unfavorable outcome were the lack of the pupillary light response and Gag reflex. The optimal time to assess prognosis was either at 48 to 72 hours or 7 days after return of spontaneous circulation. Physicians can use these neurological signs to evaluate the prognosis of post-cardic arrest survivors treated with TH.

**References**

1. Samaniego E, Mlynash M, Caulfield A, Eyngorn I, Wijman CC. Sedation Confounds Outcome Prediction in Cardiac Arrest Survivors Treated with Hypothermia. Neurocrit Care. 2011;15(1):113–9.

**Table 43 (abstract A960).** Signs for prediction of unfavorable outcome

Test Predictor	after rewarming Sensitivity% (95 % CI)	after rewarming FPR % (95 % CI)	after rewarming PPV % (95 % CI)	on 7th day Sensitivity% (95 % CI)	on 7th day FPR % (95 % CI)	on 7th day PPV % (95 % CI)
No eye opening	90.48 (77.4–97.3)	22.22 (2.8–60.0)	95 (83.1–99.4)	88.1 (74.4–96.0)	0 (0–33.6)	100 (90.5–100)
M ≤ 3	85.71 (71.5–94.5)	22.22 (2.8–60.0)	94.74 (82.1–99.4)	88.1 (74.4–96.0)	0 (0–33.6)	100 (90.5–100)
V ≤ 3	NA	NA	NA	100 (92–100)	33.3 (7.5–70.1)	93.33 (81.7–98.6)
Absent PLR	21.43 (10.3–36.8)	0 (0–33.6)	100 (66.4–100)	35.71 (21.6–52)	0 (0–33.6)	100 (78.2–100)
Absent Gag reflex	28.57 (15.7–44.6)	0 (0–33.6)	100 (73.5–100)	53.66 (37.4–69.3)	0 (0–33.6)	100 (84.6–100)
Absent Corneal	33.33 (19.6–49.6)	11.1 (0.28–48.3)	93.33 (68.1–99.8)	41.46 (26.3–57.9)	0 (0–41)	100 (80.5–100)
Absent Doll's eye	28.57 (15.7–44.6)	11.1 (0.28–48.3)	92.31 (64–99.8)	46.34 (30.7–62.6)	0 (0–33.6)	100 (82.4–100)
Myoclonus	35.71 (21.6–52)	22.2 (2.81–60.0)	88.24 (63.6–98.5)			
Seizure	16.67 (7–31.4)	11.11 (0.28–48.3)	87. 5 (47.4–99.7)			

### A961 Procalcitonin but not presepsin predicts poor outcome in the intensive care unit after out-of hospital cardiac arrest

#### P.T. Pekkarinen^1^, G. Ristagno^2^, S. Masson^2^, R. Latini^2^, S. Bendel^3^, T. Ala-Kokko^4^, T. Varpula^1^, J. Vaahersalo^1^, S. Hoppu^5^, M. Tiainen^6^, M.M. Mion^7^, M. Plebani^7^, V. Pettilä^1^, M.B. Skrifvars^1^, FINNRESUSCI Study Group

##### ^1^University of Helsinki and Helsinki University Hospital, Division of Intensive Care Medicine, Department of Anaesthesiology, Intensive Care and Pain Medicine, Helsinki, Finland; ^2^IRCCS - Istituto di Ricerche Farmacologiche 'Mario Negri', Department of Cardiovascular Research, Milan, Italy; ^3^Kuopio University Hospital, Division of Intensive Care Medicine, Kuopio, Finland; ^4^Oulu University Hospital, Medical Research Center Oulu, Department of Anaesthesiology, University of Oulu and Division of Intensive Care Medicine, Oulu, Finland; ^5^Tampere University Hospital, Department of Intensive Care, Tampere, Finland; ^6^Helsinki University Hospital, Department of Neurology, Helsinki, Finland; ^7^University-Hospital of Padova, Department of Laboratory Medicine, Padova, Italy

###### **Correspondence:** P.T. Pekkarinen – University of Helsinki and Helsinki University Hospital, Division of Intensive Care Medicine, Department of Anaesthesiology, Intensive Care and Pain Medicine, Helsinki, Finland

**Introduction:** Patients resuscitated from cardiac arrest often develop a fulminant inflammatory response called post-cardiac arrest syndrome and clinically resembling septic shock.

**Objectives:** Procalcitonin (PCT) and presepsin are biomarkers associated with severe infections. We asked, if they could be used to reflect the severity of the post-cardiac arrest syndrome and to predict poor outcome.

**Methods:** Plasma was obtained from 278 patients resuscitated from out of hospital cardiac arrest. PCT and presepsin levels were measured at ICU admission and 24, 48 and 96 hours later. Poor outcome was defined as 12-month Cerebral Performance Category (CPC) 3–5. Associations of biomarker levels with poor outcome were tested with repeated measures analysis of variance, logistic regression and receiver operating characteristic (ROC) curves with area under the curve (AUC) analysis.

Both biomarkers were assayed using commercially available chemiluminescent immunoassays: “PATHFAST™ Presepsin” for presepsin and “LIAISON® BRAHMS PCT® II GEN” for procalcitonin.

**Results:** Plasma PCT and presepsin were both significantly higher in those patients eventually presenting a poor 12-month outcome (median admission PCT 0.094 vs. 0.357 μg/L, p < 0.001; 24 h PCT 0.412 vs. 2.24 μg/L, p < 0.001; 48 h PCT 0.385 vs. 1.33 μg/L, p < 0.001; 96 h PCT 0.238 vs. 0.988 μg/L, p < 0.001; admission presepsin 269 vs. 539 ng/L, p < 0.001; 24 h presepsin 261 vs. 316 ng/L, p < 0.05; 48 h presepsin 352 vs. 508 ng/L, p < 0.01; 96 h presepsin 327 vs. 507 ng/L, p < 0.01; favorable vs. poor outcome, respectively, p-values from Mann-Whitney U test for difference between groups).

In a multivariate logistic regression model with age, ROSC-delay and type of rhythm (shockable or not), PCT at 24 h and 96 h was independently associated with poor outcome (OR 1.067; 95 % CI 1.001-1.138 and OR 1.348; 95 % CI 1.090-1.666, respectively) and in a ROC-curve 24 h PCT had an AUC 0.702 (95 % CI 0.635-0.768) and 96 h PCT an AUC 0.755 (95 % CI 0.679-0.831). In the same multivariate logistic regression model admission presepsin and 24 h presepsin both had an OR 1.000 and 95 % CI 1.000-1.001 (not statistically significant, p = 0.06 and p = 0.28, respectively) and in a ROC-curve admission presepsin had an AUC 0.717 (95 % CI 0.653-0.782) and 24 h presepsin an AUC 0.590 (95 % CI 0.518-0.663).

A significantly greater increase in procalcitonin from admission to 24 h was observed in patients with eventual poor outcome compared to those with a favorable one (p < 0.001). Presepsin levels were on average constantly higher in patients with poor outcome but did not show any statistically significant changes in repeated measures analysis of variance.

**Conclusions:** Plasma procalcitonin may be a useful tool for the evaluation of long-term outcome of out-of hospital cardiac arrest patients at the ICU. On the contrary, presepsin did not provide clinically relevant additional predictive value in the study setting.

### A962 Serum levels of high-density lipoprotein and apolipoprotein A-1 at admission can predict the neurologic outcomes in patients with cardiac arrest

#### Y. Son, K.S. Kim, G.J. Suh, W.Y. Kwon, J.I. Ko, M.J. Park

##### Seoul National University Hospital, Emergency Medicine, Seoul, Republic of Korea

###### **Correspondence:** Y. Son – Seoul National University Hospital, Emergency Medicine, Seoul, Republic of Korea

**Introduction:** Prognosis of cardiac arrest survivor is mainly determined by ischemic brain injury. Post-cardiac arrest state is characterized by elevated circulating cytokines and hemodynamic instability, called as a sepsis-like syndrome. In many critical ill diseases such as acute pancreatitis and sepsis, a low serum level of high-density lipoprotein (HDL) and apolipoprotein A-1 (ApoA1) were associated with poor outcomes.

**Objectives:** In this study, we examined whether a serum level of HDL and ApoA1 at intensive care unit (ICU) admission is associated with a neurologic outcomes in cardiac arrest survivors.

**Methods:** This study was a retrospective observational study conducted in a single tertiary urban hospital ICU. All admitted patients following cardiac arrest were screened during from March 2013 to December 2015. Patients younger than 15 years and without admission lipid panel were excluded. Neurologic outcome was determined by hospital discharge cerebral performance categories (CPC). Good neurologic outcome was defined as CPC 1 and 2.

**Fig. 71 (abstract A962).**
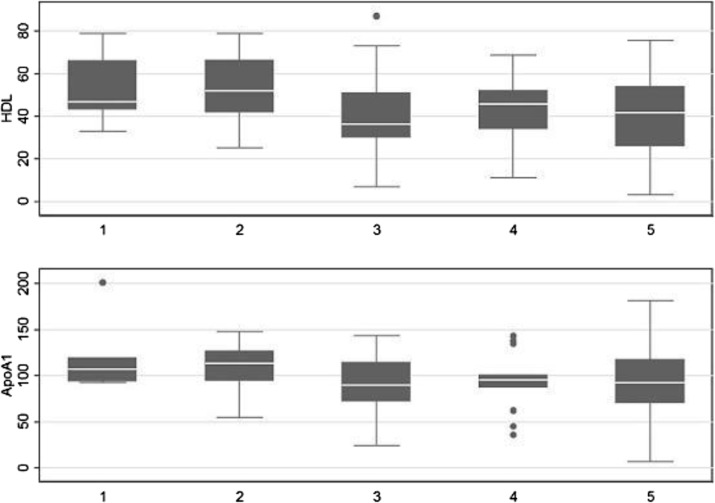
HDL and ApoA1 levels according to CPC

**Results:** During the study period, 93 patients were admitted following cardiac arrest. Among them, 19 patients without lipid panel were excluded. Among 74 patients enrolled, 18 (24.3 %) had a good neurologic outcome. Serum levels of HDL and ApoA1 were significantly higher in patients with good discharge CPC (Table 44 and Fig 71).

However, serum levels of total cholesterol, triglycerides, and apolipoprotein B were not associated with neurologic outcomes. Ares under the receiver operating curves (AUC) of HDL and ApoA1 to predict good neurologic outcome were 0.672 (0.534-0.809) and 0.667 (0.528-0.806)

**Conclusions:** High serum levels of HDL and ApoA1 were associated with good discharge neurologic outcomes in patients with cardiac arrest.

**Table 44 (abstract A962).** Lipid profiles according to outcomes

Variables	Total N = 74	Good CPC n = 18	Bad CPC n = 56	p value
Total cholesterol	143.1 (135.6–154.2)	153.5 (135.6–171.4)	139.7 (126.0–153.5)	0.294
Triglycerides	149.8 (113.7–185.8)	121.2 (38.9–203.6)	159.0 (118.1–199.9)	0.663
High density lipoprotein	43.7 (39.4–48.0)	52.8 (44.8–60.8)	40.8 (35.8–45.7)	0.016
Apolipoprotein A–1	98.1 (89.8–106.5)	113.7 (97.7–129.6)	93.2 (83.4–102.9)	0.036
Apolipoprotein B	67.2 (60.9–73.5)	74.0 (63.4–84.6)	65.0 (57.3–72.7)	0.228

### A963 Association between hemoglobin levels and clinical outcomes after cardiac arrest

#### F. Zama Cavicchi, E. Iesu, L. Nobile, J.-L. Vincent, J. Creteur, F.S. Taccone

##### Université Libre de Bruxelles - Erasme University Hospital, Department of Intensive Care, Bruxelles, Belgium

###### **Correspondence:** F. Zama Cavicchi – Université Libre de Bruxelles - Erasme University Hospital, Department of Intensive Care, Bruxelles, Belgium

**Introduction:** Hemoglobin (Hb) is a main determinant of tissue oxygen delivery, and anemia could be particularly dreadful in post-cardiac arrest (CA) brain injury.

**Aim:** To evaluate the association of Hb levels with neurological outcome patients after CA.

**Methods:** Analysis of an adult CA patients database, admitted to our Department of Intensive Care from January 2012 through December 2015, after exclusion of the 38 patients who died within the first 24 hours. We retrieved all data concerning CA characteristics as well as Hb during the first 3 days since injury as well as the need for red blood cells transfusions (RBCT). Minimum hemoglobin concentration was recorded. Anemia was defined by at least one Hb measurement < 9 g/dL. Neurological outcome was evaluated 3 months after CA (assessed during follow-up visits or by telephone interview with the general practitioner). Favourable neurological outcome (FO) was defined as a Cerebral Performance Categories (CPC) score of 1–2.

**Results:** We treated 145 patients (age 63 [52–72] years; male gender 109/145), including 78 (54 %) patients with an out-of-hospital CA (OHCA) and 58 (40 %) with a shockable initial rhythm. Median Hb concentration on admission was 11.7 [9.3-13.5] g/dL and the lowest Hb concentration was 9.2 [8.0-11.5] g/dL. Anemia was present in 66 (45 %) patients; 51 patients (35 %) received at least one RBCT and 49 of them was considered as anemic during the study period. Hemoglobin on admission was significantly lower in patients with in-hospital CA than OHCA (9.6 [8.6-11.1] vs. 13.0 [11.7-14.0] g/dL; p < 0.01). Patients with FO (n = 51) had a higher Hb on admission than those with PO (13.0 [9.45-14.0] vs. 11.1 [9.3-12.9] g/dL; p =0.04); however the proportion of patients who received a RBCT was similar (15/51 vs. 36/94; p = 0.35, respectively). The proportion of patients with FO significantly increased from the lowest range (5.5-8.9 g/dL, n = 29) to the highest range of Hb (>14.0 g/dL, n = 29) on admission (p = 0.02).

**Conclusions:** Anemia is frequent after CA, in particular after IHCA; low Hb concentrations on admission were associated with poor outcome.

### A964 Factors associated with ventilator weaning after targeted temperature management for cardiac arrest patients in Japan

#### H. Tanaka^1^, N. Otani^2^, S. Ode^2^, S. Ishimatsu^2^

##### ^1^St Luke's International Hospital, Emergency, Tokyo, Japan; ^2^St Luke's International Hospital, Tokyo, Japan

###### **Correspondence:** H. Tanaka – St Luke's International Hospital, Emergency, Tokyo, Japan

**Introduction:** Out of hospital cardiac arrest (OHCA) patients treated with targeted temperature management (TTM) may have substantial difficulty in ventilator weaning due to multiple organ failure, including post-TTM neurologic injury. Ability to predict the clinical course of TTM patients regarding duration of ventilation is important for clinical decision-making for both safer extubation and planning early tracheostomy. However, predictive factors of ventilator weaning after TTM remain unclear. Hypothesizing that weaning difficulty is associated with admission resuscitation conditions, we explore whether failure to wean may also be predicted at admission.

**Objectives:** The purpose of this study is to examine which factors predict ventilator weaning difficulty.

**Methods:** We performed a retrospective cohort study of OHCA patients brought to the emergency room at St. Luke's International Hospital in Tokyo, Japan, who underwent TTM between January 2006 and July 2015. Primary outcome was days to weaning from admission to the intensive care unit. Using the electronic medical record, we collected patient characteristics, resuscitation conditions, and examination data at admission. After characterizing weaning success using descriptive statistics, the relationship between ventilator weaning during hospitalization and resuscitation conditions were assessed with Cox regression.

**Results:** Of 115 OHCA patients who completed TTM, median time to weaning from ventilation was 6 days (4–8: IQR). 98 patients (85 %) were weaned within 2 weeks after admission. Earlier weaning was significantly associated with age (HR, 0.96; 95 % CI, 0.94 to 0.97) and motor response on admission (HR, 1.56; 95 % CI, 1.01 to 2.41). Though not significantly associated with weaning, ventricular fibrillation and asystole showed a trend towards early and later weaning, respectively, compared to Pulseless Electrical Activity.

**Conclusions:** A large majority of TTM patients can be weaned from ventilation within 2 weeks. Older age and absence of motor response at admission are related with prolonged mechanical ventilation. Early tracheostomy planning should be considered for patients after achievement of TTM who are older and does not show motor response in ER.

**Note:** This abstract has been previously published and is available at [2]. It is included here as a complete record of the abstracts from the conference.

**References**

1. N Engl J Med 2013; 369:2197–2206

2. Tanaka H, Otani N, Ode S, Ishimatsu S (2016) Factors associated with ventilator weaning after targeted temperature management for cardiac arrest patients in Japan. Critical Care 20(Suppl 2):94.

**Grant acknowledgment**

None

### A965 Factors associated to the prognosis of intra-hospital cardiac arrest in a university hospital

#### L. Martínez^1^, R. Algarte^1^, B. Sánchez^1^, I. Romero^2^, F. Martínez^1^, S. Quintana^1^, J. Trenado^1^

##### ^1^Hospital Universitari Mutua Terrassa, Critical Care Department, Terrassa, Spain; ^2^Hospital Universitari Mutua Terrassa, Terrassa, Spain

###### **Correspondence:** L. Martínez – Hospital Universitari Mutua Terrassa, Critical Care Department, Terrassa, Spain

**Introduction:** Currently there is a insufficient information about intra-hospital cardiac arrest (IHCA) in the literature, recommendations are based mainly in extra-hospital cardiac arrest knowlledge, however both the causes and the prognosis of PCRIH are possibly different, as well as the assistance and structural features for its attention, closely linked to the idiosyncrasies of each hospital.^1^

**Objectives:** To analyze the characteristics of patients attended by the IHCA team, their prognosis and related factors.

**Methods:** A retrospective, single-center and descriptive study was conducted during 2014 and 2015. We analyzed all patients admitted on hospital ward that were assisted by the IHCA team. Patients admitted less than 24 hours on Ward and patients not eligible for resuscitation were excluded.

Demographic data (age and gender) were collected. We analyzed the type of patient (medical or surgical), the schedule in which the IHCA happens (weekdays from 8 hours am to 8 hours pm and the rest, every day from 8 hours pm to 8 hours am, weekend and holidays), IHCA witnessed, the IHCA team time reaction, IHCA established or not at IHCA team´s arrival, return of spontaneous circulation (ROSC) and Hospital mortality.

Statistics:Qualitative variables are expressed as percentages and compared using the X2-test; quantitative ones are expressed as means and standard deviations (± S.D), and analyzed using Student´s t-test. Multivariate logistic regression was performed, with hospital mortality as the dependent variable. The level of significance was placed at p < 0.05. The statistical analysis was performed using specific software ( IBM SPSS Statistics for Windows, Version 19.0. Armonk, NY: IBM Corp).

**Fig. 72 (abstract A965).**
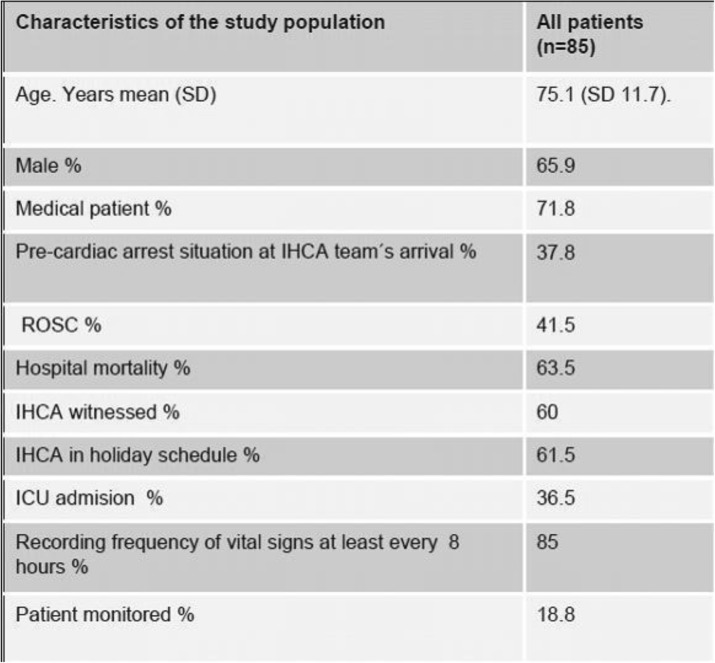
Characteristics of the study population

**Results:** 120 patients were assisted by the IHCA team and 85 patients were included. In Figure 72 we described the characteristics of the study population. The beginning of cardiopulmonary resuscitation (CPR) maneuvers were immediate on ward, according to IHCA protocol. The arrival of IHCA team was less than 5 minutes in all cases. Table 45 shows an assosciation between hospital mortality and the capability of anticipation of IHCA situations (schedule, witness, pre-cardiac arrest …).

Table 46 shows the persistence in the multivariate analysis of the relationship of these factors with the hospital mortality.

**Conclusions:** The number of activations of IHCA team is remarkable, mortality of these patients is very high despite being patients on ward without a bad expected outcome. The improvement in the factors associated with the capability of anticipation of IHCA situations (schedule, witness, pre-cardiac arrest …) could lead to an improvement in the prognosis of IHCA.

**References**

1- Morrison LJ, et al. Strategies for Improving Survival After In-Hospital Cardiac Arrest in the United States: 2013 Consensus Recommendations: A Consensus Statement From the American Heart Association. Circulation. 2013 Apr 9;127(14):1538–63.

**Table 45 (abstract A965).** Analysis of the factors associated with h

Overall hospital mortality (n85)	Death (n = 54)	Alive (n = 31)	p
Age.Years (SD)	75.6 (11.4)	74.3 (12.3)	0.61
Sex.Male %	74	71	0.42
Medical patient %	77.8	61.3	0.08
Pre-cardiac arrest situation at IHCA team´s arrival%	5.6	77.4	<0.001
Witnessed IHCA	50	85.7	0.004
IHCA in holiday schedule %	77.8	45.2	0.003
ICU admision %	31.5	45.2	0.15
Patient monitored %	18.9	28.6	0.26

**Table 46 (abstract A965).** Multivariate analisys of factors associate

Factor	Odd Ratio (95 % CI)	p Value
IHCA in holiday schedule	0.21 (0.04-0.39)	0.02
IHCA not set at IHCA team´s arrival	−0.73 [(−0.92)–(−0.54)]	<0.001
Witnessed IHCA	−0.18 [(−0.34)–(−0.02)]	0.03

### A966 Predictive value of neuron-specific enolase and procalcitonin for long-term clinical outcome in cardiac arrest survivors depends on the time of sample collection: results of a prospective study

#### D. Vondrakova, P. Ostadal, A. Kruger, M. Janotka, F. Malek, P. Neuzil

##### Na Homolce Hospital, Prague, Czech Republic

###### **Correspondence:** D. Vondrakova – Na Homolce Hospital, Prague, Czech Republic

**Introduction:** Despite marked advances in intensive cardiology care, current options for outcome prediction in cardiac arrest survivors remain significantly limited.

**Objectives:** The aim of our study was, therefore, to compare the day-specific predictive values of neuron-specific enolase (NSE) and procalcitonin in cardiac arrest survivors treated with endovascular hypothermia.

**Methods:** Eligible patients were out-of-hospital cardiac arrest survivors alive for more than 24 hours from hospital admission. All were treated with endovascular hypothermia (33 °C for 24 hours). NSE was measured using immunoturbidimetric and procalcitonin with immunoradiometric assay. Samples were collected at day 1, 2, 3, and 4, respectively, after hospital admission. Thirty-day neurological outcomes according to the Cerebral Performance Category (CPC) and 12 months mortality were evaluated as clinical endpoints.

**Results:** One-hundred-and-fifty-three cardiac arrest survivors (mean age 64.1 years) were enrolled in the study. Using ROC analysis, optimal cut-off values of NSE for prediction of CPC 1–2 at respective days were determined as: Day 1: ≤ 20.4 mcg/l (sensitivity 82.1; specificity 63.3; P = 0.0002), Day 2: ≤ 29.0 mcg/l (sensitivity 94.4; specificity 72.5; P < 0.0001), Day 3: ≤ 20.7 mcg/l (sensitivity 86.7; specificity 94.4; P < 0.0001), and Day 4: ≤ 19.4 mcg/l (sensitivity 91.0; specificity 93.5; P < 0.0001). Values > 57.1 mcg/l measured at any time predicted poor outcome (CPC 3–5) with 100 % specificity. Moreover, NSE measured at all individual days predicted also 12 month survival (P < 0.001); NSE values > 365 mcg/l were associated with death at 12 months with 100 % specificity. Procalcitonin levels predicted neurological outcome (only at days 2, 3, and 4; P < 0.05) but not 12-month mortality.

**Conclusions:** Our results indicate that NSE estimation might be useful for neurological outcome and long-term mortality prediction in cardiac arrest survivors treated with endovascular hypothermia. The highest predictive values of NSE measurement were observed at Day 3 and Day 4 after cardiac arrest. Predictive value of procalcitonin levels was only modest.

**Grant acknowledgment**

This study was supported by an Institutional grant MH CZ - DRO (Nemocnice Na Homolce - NNH, 00023884)

## VENO-ARTERIAL ECMO PRACTICE

### A967 Investigation of microcirculation in patients with venoarterial extracorporeal membrane oxygenation life support

#### Y.-C. Yeh^1^, Y.-S. Chen^2^, C.-H. Wang^2^, C.-H. Huang^1^, A. Chao^1^, C.T. Lee^1^, C.-H. Lai^2^, W.-S. Chan^3^, Y.-J. Cheng^1^, W.-Z. Sun^1^

##### ^1^National Taiwan University Hospital, Department of Anesthesiology, Taipei, Taiwan, Province of China; ^2^National Taiwan University Hospital, Department of Surgery, Taipei, Taiwan, Province of China; ^3^Far Eastern Memorial Hospital, Department of Anesthesiology, New Taipei, Taiwan, Province of China

###### **Correspondence:** Y.-C. Yeh – National Taiwan University Hospital, Department of Anesthesiology, Taipei, Taiwan, Province of China

**Introduction:** Extra-corporeal membrane oxygenation (ECMO) life support system can provide both cardiac and respiratory support to patients with heart and respiratory failure.(1) However, many patients still died in spite of ECMO support, and we suggest that the adequacy of microcirculation might be one of the major unresolved clinical problem in these patients.

**Objectives:** The aim of this study is to investigate the relationship between microcirculation and prognosis of patients with venoarterial ECMO life support system.

**Methods:** This is a prospective observational clinical trial (ClinicalTrials.gov ID: NCT02393274). Patients who received venoarterial ECMO life support system were enrolled in this study. The sublingual microcirculation was measured with an incident dark field imaging videomicroscope (Cytocam) within 6 hours after placement of ECMO, at 24 h, and other specific time points. The images were analyzed by a software tool (Automated Vascular Analysis V.3.0), and vessels with diameter smaller than 20 μm were defined as small vessel. Serum level of endothelial cell specific molecule-1(endocan) was measured at specific time points. The hemodynamic parameters, the inotropic equivalent score, and prognosis of the patients were recorded.

**Results:** 20 patients were iinvestigated in this preliminary report. They were equally divided into two groups (Survival and Non-survival) according to 28-day mortality. The baseline patient characteristics were not significantly between the two groups.

The perfused small vessel density and proportion of perfused vessels at 24 h were higher in the Survival group than in the Non-survival group.

The endocan level were higher in the Non-survival group than in the Survival group, but the difference was not significant.

**Conclusions:** Our results revealed that the perfused small vessel density were higher in the Survival group than in the Non-survival group. It encourages further studies to investigate whether aiming to improve microcirculation can improve outcomes in patient with venoarterial ECMO life support system.

**References**

1. Chen YS, Lin JW, Yu HY, et al. Cardiopulmonary resuscitation with assisted extracorporeal life-support versus conventional cardiopulmonary resuscitation in adults with in-hospital cardiac arrest: an observational study and propensity analysis. Lancet 2008;372(9638):554–561.

**Grant acknowledgment**

Supported, in part, by research grant MOST104-2314-B-002-045 from the Ministry of Science and Technology, R.O.C. and NTUH 105-A125 from the National Taiwan University Hospital.

**Table 47 (abstract A967).** Patient Characteristics and Hemodynamic Parameters

28-day	Survival (n = 10)	Non-Survival (n = 10)	P value
Age (y/o)	55(14)	55(10)	0.20
Body weight (kg)	63(12)	66(14)	0.65
Body Height (cm)	164(6)	152(32)	0.39
APACHE II at enrollment	21(8)	20(8)	0.80
Mean arterial pressure_within 6 h (mm Hg)	79(18)	76(23)	0.77
Mean arterial pressure_24h (mm Hg)	84(9)	71(20)	0.08
Inotropic equivalent score_within 6 h	31(35)	47(21)	0.36
Inotropic equivalent score_24h	20(32)	44(19)	0.16

**Table 48 (abstract A967).** Microcirculation and Endocan Level

28-day	Survival (n = 10)	Non-survival (n = 10)	P value
Total small vessel density_within 6 h (mm/mm2)	22.1(2.8)	22.7(3.8)	0.72
Total small vessel density_24h (mm/mm2)	24.7(3.5)	23.2(4.2)	0.38
Perfused small vessel density_within 6 h (mm/mm2)	19.3(2.0)	17.4(3.6)	0.16
Perfused small vessel density_24h (mm/mm2)	21.9(5.1)	12.3(10.2)	0.02
Proportion of perfused small vessels_within 6 h (%)	89(13)	78(17)	0.14
Proportion of perfused small vessels_24h (%	89(16)	52(39)	0.02
Endocan level_within 6 h (ng/mL)	26 (20)	85 (100)	0.10
Endocan level_24h (ng/mL)	74 (62)	105 (133)	0.52

### A968 Extracorporeal life support in patients with severe pulmonary embolism

#### S. Kaese^1^, C. Horstmann^2^, P. Lebiedz^2^

##### ^1^Division of Electrophysiology, Department of Cardiovascular Medicine, University of Münster, Münster, Germany; ^2^Division of Cardiology, Department of Cardiovascular Medicine, University of Münster, Münster, Germany

###### **Correspondence:** S. Kaese – Division of Electrophysiology, Department of Cardiovascular Medicine, University of Münster, Münster, Germany

**Introduction:** Extracorporeal life support (ECLS) is used in patients with cardiac arrest and may prolong the period of time for treatment of reversible causes of cardiac arrest. Studies evaluating the usefulness of ECLS in patients with severe pulmonary embolism (PE) and cardiogenic shock are lacking. Therefore, current guidelines suggest that ECLS can be an effective procedure in patients with massive PE, but only based on experimental evidence or case reports^1^.

**Objectives:** This study evaluated the usefulness of ECLS in patients with cardiac arrest or right heart failure due to severe PE. Further, we determined occurrence of complications.

**Methods:** All patients admitted to our intensive care unit (ICU) from November 2011 to January 2016 with right heart failure or cardiac arrest due to PE and ECLS therapy were included in the study. We evaluated the duration of ECLS therapy, need for CPR and complication rate due to lysis or ECLS therapy.

**Results:** The study includes 11 patients (3 female) with a mean age of 45.0 ± 12.1 years. Mean duration of ICU-stay was 22.5 ± 24.0 days. The emergency medical service admitted 5 patients to the ICU. 4 of these patients suffered from out of hospital cardiac arrest. In 1 patient, return of spontaneous circulation (ROSC) was achieved out of hospital by the emergency medical service. The remaining 3 patients were transported to the ICU during ongoing CPR. In two of them, ECLS was realized during continuous CPR with subsequent ROSC after ECLS implementation. Additional 5 patients were initially treated in other hospitals and subsequently transferred to our ICU due to severe PE. One patient presented herself at our emergency department. All patients received systemic intravenous thrombolysis before implementation of ECLS. Prior to ECLS therapy, 10 of 11 patients suffered from cardiac arrest with need for CPR of different duration. Further, one patient received ECLS therapy due to severe right heart failure. Arterial and venous cannula were inserted into femoral vessels in 9 (81.8 %) patients. In 2 (18.2 %) patients, arterial cannula was placed surgically into the arteria subclavia dextra by using a patch. After systemic lysis and subsequent implementation of ECLS cannulation, bleeding complications occurred in 4 (36.4 %) patients and 2 (18.2 %) had vascular injury with need for surgical treatment. ECLS therapy was performed with a mean duration of 5.6 ± 6.8 days. 6 of 11 patients (54.5 %) died during treatment. 5 patients died due to multi-organ failure (45.5 %) and 1 patient (9.1 %) due to sclerosing cholangitis. 5 of 11 patients (45.5 %) were discharged from hospital.

**Conclusions:** ECLS therapy may be useful and beneficial in patients with cardiac arrest and right heart failure due to severe PE as a bridge to recovery. Despite preceding systemic thrombolysis, ECLS cannulation can be performed with an acceptable complication rate.

**References**

1. Konstantinides SV et al.: European Heart Journal 2014

**Grant acknowledgment**

No grant

### A969 Extracorporeal membrane oxygenation versus impella for cardiogenic shock caused by myocardial infarction: retrospective, observational, single-center cohort study

#### M. Mourad, P. Gaudard, J. Eliet, N. Zeroual, P. Colson

##### CHU Montpellier, Montpellier, France

###### **Correspondence:** M. Mourad – CHU Montpellier, Montpellier, France

**Introduction:** Mechanical circulatory support (MCS) is increasingly used for cardiogenic shock (CS) caused by acute myocardial infarction (AMI) but scientific evidences for determining the optimal implantation timing and which is the appropriate device, are missing^123^.

**Objectives:** To compare extracorporeal membrane oxygenation (ECMO) and Impella as first MCS for CS occurring early after AMI.

**Methods:** Retrospective, observational, single-center cohort study

**Setting:** 14-bed surgical ICU in Academic Hospital

Patients: Patients admitted in ICU for MCS shortly (<48 hours) after onset of CS occurring at the early phase (<72 hours) after AMI, excluding persistent cardiac arrest before MCS.

**Results:** From January 2009 to April 2015, among 88 patients supported with MCS after AMI, 42 met the study criteria. MCS implantation occurred mainly (93 %) during or just after percutaneous coronary intervention (PCI): 23 patients were first treated with ECMO, 19 with Impella. Cardiac management including PCI was no significantly different between the groups. ECMO patients were sicker (higher vasoactive-inotrope and ENCOURAGE scores, higher blood lactate level) than Impella patients (p £ 0.02). Secondary combination of both techniques was needed in 10 patients, either for insufficient circulatory support with Impella (21 %) or for left ventricle overload with ECMO (26 %). Both strategies allowed a rapid improvement of organ perfusion over a median MCS duration of 8 days (1–12) and a 6-month survival rate of 55 % (p = 0.95 between groups).

**Conclusions:** ECMO is a very effective circulatory support but exposes to LV overload. Impella devices, especially percutaneous models, may be not enough to get appropriate circulatory support. Interestingly, the combination of the techniques may overcome the limits inherent to each device. Impella, which is a less demanding technique, might help to anticipate serious adverse events during high-risk PCI situations like AMI CS without compromising a more effective MCS with ECMO if needed afterwards.

**References**

1. Sheu J-J, Tsai T-H, Lee F-Y, et al.: Early extracorporeal membrane oxygenator-assisted primary percutaneous coronary intervention improved 30-day clinical outcomes in patients with ST-segment elevation myocardial infarction complicated with profound cardiogenic shock. *Crit Care Med* 2010; 38:1810–1817

2. O'Neill WW, Schreiber T, Wohns DHW, et al.: The current use of Impella 2.5 in acute myocardial infarction complicated by cardiogenic shock: results from the USpella Registry. *J Intervent Cardiol* 2014; 27:1–11

3. Werdan K, Gielen S, Ebelt H, et al.: Mechanical circulatory support in cardiogenic shock. *Eur Heart J* 2014; 35:156–167

### A970 Synchronized pulsatile extracorporeal life support preserves left ventricular function and coronary flow in a porcine model of cardiogenic shock

#### P. Ostadal^1^, M. Mlcek^2^, M. Hrachovina^2^, A. Kruger^1^, D. Vondrakova^1^, M. Janotka^1^, M. Mates^1^, P. Hala^1^, O. Kittnar^2^, P. Neuzil^1^

##### ^1^Na Homolce Hospital, Prague, Czech Republic; ^2^Charles University in Prague, Prague, Czech Republic

###### **Correspondence:** P. Ostadal – Na Homolce Hospital, Prague, Czech Republic

**Introduction:** Veno-arterial extracorporeal life support (ECLS) is increasingly being used to treat rapidly progressing or severe cardiogenic shock. However, it has been repeatedly shown that increased afterload associated with ECLS significantly diminishes left ventricular (LV) performance. A new electrocardiogram (ECG)-synchronized pulsatile cardiac assist system, which offers full circulatory support with increased diastolic and decreased systolic extracorporeal flow, has recently been introduced.

**Objectives:** To compare the parameters of LV function and coronary flow during standard continuous-flow ECLS support and ECG-synchronized pulsatile ECLS flow in a porcine model of cardiogenic shock.

**Methods:** Sixteen female swine (mean body weight 45 kg) underwent ECLS implantation under general anesthesia and artificial ventilation. Subsequently, acute cardiogenic shock, with documented signs of tissue hypoperfusion, was induced by initiating global myocardial hypoxia. Hemodynamic cardiac performance parameters and coronary flow were then measured at different rates of continuous or pulsatile ECLS flow (ranging from 1 L/min to 4 L/min) using arterial and venous catheters, a pulmonary artery catheter, an LV pressure-volume loop catheter and a Doppler coronary catheter.

**Results:** Myocardial hypoxia resulted in declines in mean cardiac output (CO) to 2.2 ± 0.8 L/min, systolic blood pressure to 60 ± 5 mmHg and LV ejection fraction (LVEF) to 22 ± 5 %. Synchronized pulsatile flow was associated with a significant reduction in LV end-systolic volume by 6.2 mL (6.7 %), an increase in LV stroke volume by 5.0 mL (17.4 %), higher LVEF by 4.5 % (18.8 % relative), CO by 0.37 L/min (17.1 %) and mean arterial pressure by 3.0 mmHg (5.5 %) at all ECLS flow rates when compared with continuous ECLS flow (P < 0.05). At selected ECLS flow rates, pulsatile flow also reduced LV end-diastolic pressure, end-diastolic volume and systolic pressure. ECG-synchronized pulsatile flow was also associated with significantly increased (7 % to 22 %) coronary flow at all ECLS flow rates.

**Conclusions:** The results suggest that ECG-synchronized pulsatile ECLS flow preserved LV function and coronary flow compared with standard continuous-flow ECLS in this particular setting of cardiogenic shock.

**Grant acknowledgment**

The study was supported by an Institutional grant MH CZ - DRO (Nemocnice Na Homolce - NNH, 00023884) and by SVV266503/2013.

### A971 Success of ecls weaning with levosimendan

#### A. Jacky^1^, A. Rudiger^2^, D.R. Spahn^2^, D.A. Bettex^2^

##### ^1^Cantonal Hospital Winterthur, Winterthur, Switzerland; ^2^University Hospital Zurich, Institute for Anaesthesiology, Cardio-surgical Intensive Care Unit, Zurich, Switzerland

###### **Correspondence:** A. Jacky – Cantonal Hospital Winterthur, Winterthur, Switzerland

**Introduction:** Veno-arterial extracorporeal life support (ECLS) is used in selected patients with cardiogenic shock after cardiovascular surgery. Weaning from ECLS requires temporary pharmacological support and/or the use of an intra-aortic balloon pump (IABP). Recent insights have demonstrated potential harm for both. Levosimendan might be a valuable alternative to support circulation during and after the weaning phase. There is currently a paucity of evidence on the feasibility and success of such an approach.

**Objectives:** To investigate effectiveness and safety of levosimendan for ECLS weaning.

**Methods:** In 2011 levosimendan was introduced for ECLS-weaning. We retrospectively studied all patients under ECLS after cardiac surgery between 2007 and 2013. Patients were excluded if they were under the age of 18 or had not undergone cardiac surgery. Results are given as median (interquartile range) or numbers (%). Group comparisons between patients with (L) and without (C) levosimendan were made with the Mann-Whitney U test or the Chi-Square test as appropriate.

**Results:** Of 64 patients, 26 (41 %) received levosimendan. They were treated more recently (2012 (2012–2013) vs 2009 (2007–2010), p < 0.001). No significant differences were found for age (65 (57–71) years, p = 0.65), gender (50 (78 %) male, p = 0.77), type of surgery (30 (47 %) elective procedures, p = 0.80). There was a trend toward higher disease severity and mortality scores (SAPS II 53 (42–62) vs 49 (40–60), p = 0.30; EuroScore II 9 (6–12) vs 8 (3–13), p = 0.41), and multiorgan failure was diagnosed more often in group L (16 (62 %) vs 10 (26 %), p = 0.009). Successful weaning was achieved in 24 (92 %) patients in group L and in 30 (79 %) in group C, p = 0.18. In group L duration of weaning was longer (36 (23–61) vs 27 (14–46) hours, p = 0.013), and IABP was used less often (2 (7.7 %) vs 15 (40 %), p = 0.008). Needs of beta mimetic agents are presented in the table. Mortality at 28 days (35 % vs 40 %, p = 0.79) and 180 days (50 % vs 44 %, p = 0.80) was similar in both groups.

**Conclusions:** ECLS after cardiac surgery is associated with high morbidity and mortality. The introduction of levosimendan for ECLS weaning reduced the need for IABP and milrinone, without increasing the noradrenaline requirements. Despite higher severity of illness, patients treated with levosimendan had a high success rate of ECLS weaning.

**Fig. 73 (abstract A971).**
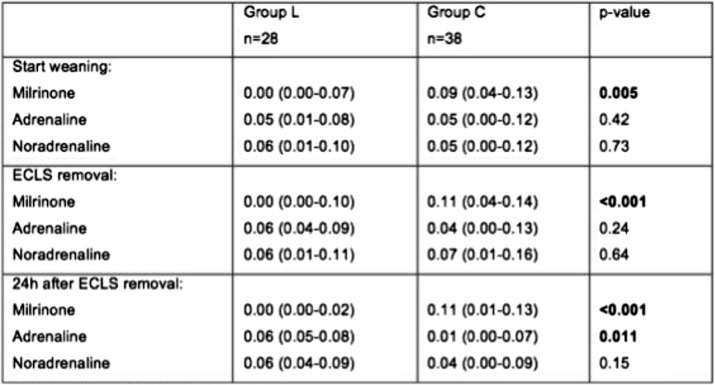
ᅟ

### A972 Prediction of veno-arterial extracorporeal membrane oxygenation (VA-ECMO) outcome by microcirculatory monitoring in patients with cardiogenic shock

#### A. Kara, S. Akin, D. Dos reis Miranda, A. Struijs, K. Caliskan, R.J. van Thiel, E.A. Dubois, W. de Wilde, F. Zijlstra, D. Gommers, C. Ince

##### Erasmus Medical Center, Rotterdam, Netherlands

###### **Correspondence:** A. Kara – Erasmus Medical Center, Rotterdam, Netherlands

**Objectives:** Veno-arterial extracorporeal membrane oxygenation (VA-ECMO) is an effective technique for providing emergency mechanical circulatory support for patients with cardiogenic shock. VA-ECMO enable us a rapid restoration of global systemic organ perfusion, it has not been found to always have a parallel improvement of the microcirculation. We hypothesized in this study that the response of the microcirculation to the initiation of VA-ECMO might identify patients with ICU survival.

**Methods and results:** Twenty-four patients were included in the study. Sublingual microcirculation measurements were performed using the CytoCam-IDF (Incident-Dark-Field) imaging device (CytoCam, Braedius Medical, Huizen the Netherlands). Microcirculatory measurements were performed at: baseline, after VA-ECMO insertion (T1); 48-72 h after initiation VA-ECMO (T2); 5-6days after (T3); 9–10 days after (T4); and within 24 hours explantation of VA-ECMO.

Of the 24 patients included in the study population, 15 survived and 9 died while on VA-ECMO. There was no significant difference between the systemic global hemodynamic variables at initiation of VA-ECMO between the survivors or non-survivors. There was, however, a significant difference in the microcirculatory parameters of both small and large vessels at all time points between the survivors and non-survivors. Perfused vessel density (PVD) at baseline (survivor vs. nonsurvivor; 19.21, 13.78 mm/mm2 p = 0.001) was able to predict ICU survival on initiation of VA-ECMO, areas under the ROC (within a 95 % confidence interval, gave values of 0.911 (0.792-1.0).

**Conclusion:** Perfused vessel density of the sublingual microcirculation at initiation of VA-ECMO can be used to predict ICU survival in patients with cardiogenic shock. yes

**Grant acknowledgment**

None.

### A973 Tolerance to enteral feeding in patients treated with extracorporeal membrane oxygenation: a matched cohort study

#### L. Marca, A. Xini, W. Mongkolpun, C.P.R. Cordeiro, R.T. Leite, O. Lheureux, A. Bader, L. Rincon, C. Santacruz, J.-C. Preiser

##### University Hospital Erasme, Université Libre de Bruxelles, Critical Care, Brussels, Belgium

###### **Correspondence:** W. Mongkolpun – University Hospital Erasme, Université Libre de Bruxelles, Critical Care, Brussels, Belgium

**Introduction:** Extracorporeal membrane oxygenation (ECMO) is widely used for severe cardiogenic shock and acute respiratory distress syndrome (ARDS). Patients with ECMO also require sedation (midazolam), vasopressor agents (noradrenalin, NA). The effects of ECMO and associated treatments on gastrointestinal function are unknown.

**Objective:** To evaluate gastrointestinal intolerance evaluated by gastric residual volume (GRV) in patients treated with ECMO and in a matched cohort of patients with ARDS not treated with ECMO.

**Method:** 31 patients with veno-arterial (VA) ECMO, 30 patients with veno-venous (VV) ECMO and 34 ARDS patients who stayed in ICU ≥ 5 days from 2011 to 2015, were enrolled. Demographic data, sedation and vasopressor dose were recorded. Enteral feeding was started as soon as possible. The cumulative GRV was recorded up to a maximum of 7 days, with a cut-off value of 250 ml used to define intolerance to enteral feeding (IEF). All data is presented as median(p25-p75.) Statistical analysis was performed by STATS13.0 with Mann-Whitney U test and Chi-square test.

**Results:** Data from 95 patients were recorded. Baseline demographic data were similar in the 3 groups. The average GRV and the doses of midazolam were the highest in patients with VV ECMO, while the number of days with IEF and the doses of NA were the highest in the VA ECMO group (Table 49). Overall, GRV and number of days with IEF tended to be higher in survivors (n = 63, GRV 112 (0–360), days with IEF 3(2–5)) than in non-survivors (n = 33, GRV 364 (100–488, days with IEF 4(3–6)).

**Conclusion:** Early enteral feeding is feasible during ECMO, in spite of impairments of gastrointestinal function potentially related to sedation and/or vasopressor treatment.

**Table 49 (abstract A973).** Demographic data and average gastric residual volu

	VV ECMO (n = 30)	VA ECMO (n = 31)	All ECMO (n = 61)	P	ARDS (n = 34)	Total (n = 95)	p
ICU mortality (n%)	9(33 %)	15(48 %)	24(39 %)	0.17	10(29 %)	34(35 %)	0.30
Age (years)	54(38–62)	57(43–67)	55(41–65)	0.20	58(48–64)	56(43–64)	0.55
APACHE II score	23(20–25)	24(21–30)	23(21–27)	0.23	19(15–25)	22(18–27)	0.06
Average gastric residual volume(ml/day)	257(183–384)	133(23–230)	189(87–298)	0.007	190(0–390)	189(54–366)	0.60
Total day intolerance(days)	3(1–5)	5(4–7)	4(2–6)	0.06	4(2–6)	4(2–6)	0.5
Average Midazolam(mg/day)	127(100–127)	99 (33–127)	130(65–177)	0.003	22(9–79)	70(23–177)	0.01
Average Noradrenaline(mcg/day)	4(0–67)	141(30–260)	77(8–135)	0.003	11(3–54)	51(5–150)	0.01

### A974 Extracorporeal membrane oxygenation for refractory cardiogenic shock in patients with peripartum cardiomyopathy

#### A. Chao^1^, A.-S. Chao^2^, Y.-S. Chen^3^

##### ^1^National Taiwan University Hospital, Taipei, Taiwan, Province of China; ^2^Chang Gung Memorial Hospital, Linkou, Taoyaun, Taiwan, Province of China; ^3^National Taiwan University Hospital, Department of Surgery, Taipei, Taiwan, Province of China

###### **Correspondence:** A. Chao – National Taiwan University Hospital, Taipei, Taiwan, Province of China

**Introduction:** Peripartum cardiomyopathy (PPCM) is a rare complication of pregnancy with unknown etiology. The clinical course of PPCM is highly variable; some patients develop unresponsive cardiogenic shock requiring mechanical circulatory support. PPCM accounted for the second most common etiology of cardiomyopathy-related transplant in women in the United States. (1) Extracorporeal membrane oxygenation (ECMO) has been widely used to rescue patients with cardiopulmonary arrest. (2) But its clinical utility in severe PPCM remains unclear.

**Objectives:** We investigate a case series of six patients presenting with refractory shock due to PPCM who were rescued by veno-arterial ECMO.

**Methods:** This is a retrospective study (No 201512232RIND) approved by our Institute Review Board. Patient data was extracted from our ECMO registry between the year of 1995 and 2015.

**Results:** Six patients with confirmed PPCM were found. Two (33 %) patients died of neurological consequences (cerebral infarct and hypoxic encephalopathy) and their left ventricular (LV) ejection fraction remained about 20 %. One patient underwent heart transplantation. The other three patients weaned off ECMO and their LV function began to improve on day 3. They were discharged uneventfully.

**Conclusions:** ECMO can provide an effective and simple treatment for critical PPCM with a satisfactory result. Patients supported by ECMO whose heart function did not begin to recover on day 3 and had neurological complications had a poor prognosis.

**References**

1. Rasmusson KD, Stehlik J, Brown RN, et al. Cardiac Transplantation Research Database Group, Long-term Outcomes of Cardiac Transplantation for Peripartum Cardiomyopathy: A Multiinstitutional Analysis. J Heart Lung Transplant 2007;26:1097–104.

2. Chen YS, Lin JW, Yu HY, et al. Cardiopulmonary Resuscitation with Assisted Extracorporeal Life Support versus Conventional Cardiopulmonary Resuscitation in Adults with In-hospital Cardiac Arrest: An Observational Study and Propensity Analysis. Lancet 2008;372:554–61.

**Grant acknowledgment**

Supported, in part, by research grants NTUH 105-S3181 and MG-213 from the National Taiwan University Hospital.

**Table 50 (abstract A974).** Patient data

Patient Patient Year	1 1995	2 2007	3 2008	4 2008	5 2010	6 2012
Age	37	24	29	32	26	33
Obstetric history and Gestational age, weeks	G5P1SA3 35 (twins)	G3P1AA1 Term	G1P0 34	G2P2 Term	G1P0 30	G1P0 30 (twins)
Medical history	Nil	Thalassemia	ALL, complete remission	Thalassemia	Nil	Lymphoma, complete remission
Time onset	Shortly after delivery	3rd trimester	34th week pregnancy	2 months after delivery	30th week pregnancy	29th week pregnancy
Clinical pictures	Acute pulmonary edema, Ventricular tachycardia	Ventricular tachycardia	Acute pulmonary edema	Acute pulmonary edema	APE, atrial tachycardia	Acute pulmonary edema

**Table 51 (abstract A974).** Clinical course

Patient	1	2	3	4	5	6
Cardiac echo On ECMO (LVEF) Day 3 ECMO	25 39 50	21 19	18 27	17 69 (post-transplantation)	14 21	24 20
Other interventions	Nil	Left ventricular assist device	Femoral arterial endarterectomy, Femoral-Femoral bypass after ECMO	Heart transplantation	Radio-frequency catheter ablation for SVT, CAVH	CAVH
ECMO duration (hour)	96	194	203	36	146	143
ICU LOS, day	10	8	16	6	10	5
Hospital LOS, since ECMO, day	25	8	21	32	15	5
Maternal outcome	Survived	Dead, brain death	Survived	Survived	Survived	Dead, multiple brain infarct, MOF
Fetal outcome	Not affected	Not affected	Good condition	Not affected	Fetal loss	One in good condition, another died.

### A975 Efficacy of extracorporeal versus conventional cardiopulmonary resuscitation for adult cardiac arrest patients: a systematic review and meta-analysis

#### W. Kim^1^, C. Ahn^2^, Y. Cho^1^, T.H. Lim^2^, J. Oh^2^, K.-S. Choi^3^, B.-H. Jang^4^, J.K. Ha^1^

##### ^1^Hallym University, Emergency Medicine, Seoul, Republic of Korea; ^2^Hanyang University, Emergency Medicine, Seoul, Republic of Korea; ^3^Hanyang University, Neurosurgery, Seoul, Republic of Korea; ^4^Kyung Hee University, Preventive Medicine, Seoul, Republic of Korea

###### **Correspondence:** W. Kim – Hallym University, Emergency Medicine, Seoul, Republic of Korea

**Introduction:** To increase the survival rate post-CPR, the application of extracorporeal membrane oxygenation during CPR, referred to as extracorporeal CPR (ECPR), has been selectively attempted on patients who experience cardiac arrest due to reversible etiologies.

**Objectives:** To assess the impact of extracorporeal cardiopulmonary resuscitation (ECPR) as compared to conventional cardiopulmonary resuscitation (CCPR) in adult patients who experience cardiac arrest of cardiac origin.

**Methods: Data sources.** We performed a comprehensive search according to criteria set forth in a predefined protocol. The study was conducted based on the Meta-analysis of Observational Studies in Epidemiology (MOOSE) group and the Preferred Reporting Items for Systematic Reviews and Meta-analysis (PRISMA) group procedures.

**Study selection.** Report inclusion criteria were adult patients with cardiac arrest of cardiac origin in whom ECPR was compared to CCPR, and available survival and neurological outcomes. Exclusion criteria were non-cardiac origin arrest, and review articles, editorials, or nonhuman studies.

**Data extraction.** Two reviewers screened abstracts; a third reviewer arbitrated when necessary.

The efficacy of ECPR was compared to CCPR in terms of survival and neurological outcome as follows: 1) survival to discharge from hospital or 28 days, and 2) good neurologic outcome (Cerebral Performance Category 1–2 or Glasgow Outcome Scale 1) maintained until discharge from hospital or 28 days or 90 days. Odds ratios (ORs) were calculated using a random effects model.

**Results:** A total of 38,366 patients from 8 studies were included. ECPR showed equal survival (OR 1.03, 95 % confidence interval [CI] 0.41-2.56) and neurologic outcomes (OR 2.68, 95 % CI 0.62-11.57) to CCPR in out-of hospital cardiac arrest (OHCA) patients. However, in in-hospital cardiac arrest (IHCA) patients, ECPR showed significantly better survival (OR 3.07, 95 % CI 1.52-6.20) and neurologic outcomes (OR 3.40, 95 % CI 1.49-7.74) than CCPR.

**Conclusions:** ECPR is associated with improved survival and neurologic outcome than CCPR in cardiac origin IHCA patients. However, there are no such benefits for ECPR in OHCA patients.

**References**

1. Kouwenhoven WB, Jude JR, Knickerbocker GG: Closed-chest cardiac massage. *JAMA* 1960;173:1064–1067.

2. Acosta P, Varon J, Sternbach GL, et al.: Resuscitation great. Kouwenhoven, Jude and Knickerbocker: The introduction of defibrillation and external chest compressions into modern resuscitation. *Resuscitation* 2005;64:139–143.

3. Mégarbane B, Leprince P, Deye N, et al.: Emergency feasibility in medical intensive care unit of extracorporeal life support for refractory cardiac arrest*. Intensive Care Med* 2007;33:758–764.

**Grant acknowledgment**

This research was supported by the Civil research projects for solving social problems through the National Research Foundation of Korea (NRF) funded by the Ministry of Science, ICT, and Future Planning (NRF-2015M3C8A7A02027410).

### A976 The incidence of bleeding complications in patients after cardiac arrest treated with va-ecmo and targeted temperature management

#### A. Mecklenburg^1^, J. Stamm^1^, G. Soeffker^1^, M. Kubik^1^, K. Sydow^2^, H. Reichenspurner^3^, S. Kluge^1^, S. Braune^4^

##### ^1^University Medical Center Hamburg-Eppendorf, Intensive Care Medicine, Hamburg, Germany; ^2^University Heart Center Hamburg-Eppendorf, General and Interventional Cardiology, Hamburg, Germany; ^3^University Heart Center Hamburg-Eppendorf, Cardiovascular Surgery, Hamburg, Germany; ^4^St. Franziskus Hospital Münster, Pneumology and Intensive Care Medicine, Münster, Germany

###### **Correspondence:** A. Mecklenburg – University Medical Center Hamburg-Eppendorf, Intensive Care Medicine, Hamburg, Germany

**Introduction:** The use of venoarterial extracorporeal membrane oxygenation (va-ECMO) for prolonged cardiopulmonary resuscitation (CPR) and severe cardiogenic shock after CPR has widely increased (1). Bleeding complications, due to necessary therapeutic anticoagulation and CPR/SIRS induced coagulopathy are common (2). Targeted temperature management (TTM) has shown positive effects on neurological outcome after CPR. Although optimal target temperature is not exactly known, TTM remains a recommended approach in patients after CPR (3).

**Objectives:** To determine the incidence of bleeding complications in patients after CPR, who are on va-ECMO and treated with TTM (target temperature 34 °C) simultaneously.

**Methods:** We conducted a retrospective observational study from Jan 2009 to Dec 2015 and extracted relevant clinical data from electronically medical records. Outcomes of interest were 28d-mortality and incidence of bleeding complications within 36 hrs of CPR. Demographic data, (anti-)coagulation status and need for transfusion were also analyzed.

**Results:** A total of 258 patients received va-ECMO during the study period of which 183 patients (70,9 %) underwent CPR before ECMO. Of these, 44 patients (24 %) were treated with TTM. The median age was 49 yrs (range 22–87 yrs) and 31 patients were male (71 %). SOFA score on admission was 15 (3–20). Patients received CPR mainly because of either acute myocardial ischemia (MI) (19; 43 %) or malignant dysrhythmias not attributable to acute MI (11; 25 %). ECMO implantation was performed within 3 hrs (0.5-26 hrs) of CPR and ECMO duration was 92.5 hrs (6–648 hrs). TTM was implemented within 2 hrs (0–7 hrs) of CPR and the duration of simultaneous treatment with va-ECMO and TTM was 21.3 hrs (2–48 hrs). 40 patients (91 %) received heparin and 25 patients (57 %) additional anticoagulants (Aspirin, ADP-receptor-antagonists, GIIa/IIIb-receptor-antagonists). The 28d-mortality rate was 66 %. Within the first 36 hrs of CPR 37 patients (84 %) experienced bleeding complications on different sites and of variable degrees. Epistaxis and bleeding on cannulation site were the most common bleeding complications with 27 % (12) and 44 % (19), respectively. Two patients (4,5 %) experienced intracerebral bleeds and fatal bleeding occurred in 9 patients (20,5 %).

**Conclusions:** Bleeding complications were very common in our study population of patients treated with va-ECMO and TTM simultaneously. Our preliminary data suggest that great caution should be applied to the management of (anti)coagulation in patients treated with va-ECMO and TTM. Further prospective studies are needed to better understand the attributable risk of TTM on the occurrence of bleeding complications in va-ECMO patients.

**References**

(1) Hryniewicz, ASAIO, 2016

(2) Gray, ASAIO, 2015

(3) Nolan, Intensive Care Medicine, 2015

**Grant acknowledgment**

None.

### A977 Extracorporeal low flow CO2 removal as a bridge to urgent lung transplantation: a single center experience

#### B. Bergantino^1^, F. Ruberto^1^, E. Magnanimi^1^, E. Privato^1^, V. Zullino^1^, K. Bruno^1^, F. Pugliese^1^

##### ^1^Università La Sapienza di Roma, UOD Terapia Intensiva nei Trapianti d'Organo, Policlinico Umberto I, Roma, Italy

###### **Correspondence:** B. Bergantino – Università La Sapienza di Roma, UOD Terapia Intensiva nei Trapianti d'Organo, Policlinico Umberto I, Roma, Italy

**Introduction:** Lung transplant is the last therapy for patients with end stage respiratory failure. Patients on waiting list may need mechanical ventilation and extracorporeal support right before the lung transplant, due to sudden and rapid worsening of their lung disease. In these cases an urgent lung transplantation is necessary. ECMO as a bridge to lung transplant is widely used: survival of pretransplant ECMO patients improved over the last years, but ECMO treatment is related to severe complications, such as bleeding and intracranial hemorrhages, with a decline in survival for treatments longer than 2 weeks. Today low flow venovenous extracorporeal CO2 removal (ECCO2R) devices have been available. They remove CO2 using a mini-invasive system (blood flow < 450 ml/min), requiring lower anticoagulation than ECMO and easier management.

**Methods:** The study is a retrospective analysis: we analyzed all patients admitted to our Transplant Center Intensive Care Unit (ICU), listed for an urgent lung transplantation. We included patients presenting respiratory acidosis not responding to mechanical ventilation with PaCO2 > 75 mmHg and pH < 7.20, mild hypoxia and stable hemodynamic conditions, who received ECCO2R treatment (Decapsmart®, or ProLung ®). Patients on ECMO were excluded.

We analyzed ECCO2R devices feasibility as a bridge to lung transplantation, examining blood gasses values, hemodynamic parameters and mechanical ventilation setting during treatment.

**Results:** From January 2010 to March 2016, 18 patients were admitted to Transplant ICU, listed for an urgent lung transplantation. 8 patients underwent ECMO. 10 patients were supported with ECCO2R for severe respiratory acidosis, so their data were analyzed.

Mean treatment duration was 76 ± 44 hours; ECCO2R efficiently removed CO2 reducing the severe acute hypercapnia adverse events; within the 20^th^ hour, pH and blood gasses improved, with a significant mechanical ventilation support reduction and mean tidal volume increasing.

1 patient was bridged to lung transplant successfully without ECMO. In 4 patients, because of severe hypoxia, ECCO2R treatment was rapidly replaced by veno-venous ECMO, bridging them all to transplant, too. 5 patients died in ICU waiting for an available organ.

**Conclusions:** Nowadays in Italy, the main problem for patients awaiting for a lung transplantation is the real low organ availability and the very long time on the waiting list. ECCO2R can provide further options for hypercapnic respiratory disease, but because systemic oxygenation improvement is slight and there's no hemodynamic support, actually in our experience, it cannot substitute standard ECMO as a bridge to lung transplant, especially if extracorporeal support is required for a long time. But for selected patients, its simpler management and minimal requirement for anticoagulation can allow us to gain time to avoid ECMO and its severe related complications.

### A978 Comparison between picco and pac methods to evaluate the cardiac index during veno-venous extracorporeal membrane oxygenation. A prospective observational study

#### G. Sales^1^, V. Girotto^1^, F. Vittone^2^, L. Brazzi^1,2^

##### ^1^University of Turin, Dept. of Surgical Sciences, Turin, Italy; ^2^Città della Salute e della Scienza Hospital, Dept. of Anesthesiology, Turin, Italy

###### **Correspondence:** V. Girotto – University of Turin, Dept. of Surgical Sciences, Turin, Italy

**Introduction:** Cardiac index (CI) monitoring in patients with severe acute respiratory distress syndrome (ARDS) is important to optimize organ perfusion, avoiding pulmonary fluid-overload. [1] CI can be measured using both pulmonary artery catheter (PAC) method and less invasive transpulmonary thermodilution-based PiCCO system. [2] However, the accuracy of PiCCO during extracorporeal membrane oxygenation (ECMO) is still unconfirmed.

**Objectives:** To compare the effectiveness of PAC and PiCCO methods to measure CI during venovenous ECMO.

**Methods:** Data were collected in two patients treated with femoro-femoral veno-venous ECMO for severe ARDS. In both cases, continuous cardiac output PAC catheters (Edwards Lifesciences, CA) were placed in the internal jugular veins, while PiCCO catheters (Pulsion Medical Systems, DE) were introduced in the brachial arteries. With extracorporeal blood flows in the range of 3-4 L/min and mean arterial blood pressure >70 mmHg, CI determinations were performed twice daily in both patients (at 10.00 AM and at 6.00 PM) simultaneously with PAC and PiCCO systems (CI_PAC_ and CI_PiCCO_). Mean values between three thermodilutions were recorded for each measurement. Statistics were performed according to the regression linear analysis and the Bland-Altman method. Significance was assumed at a p-value < 0.05.

**Fig. 74 (abstract A978).**
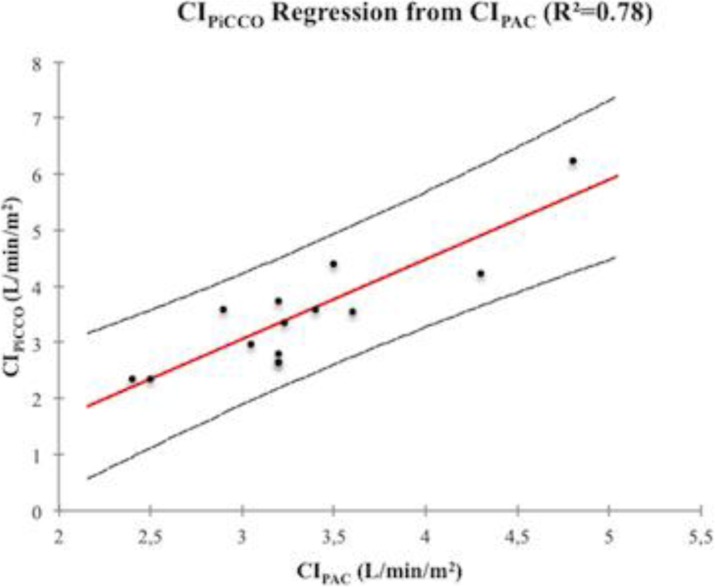
CI_PiCCO_ Regression from CI_PAC_ (R^2^ = 0.78)

**Fig. 75 (abstract A978).**
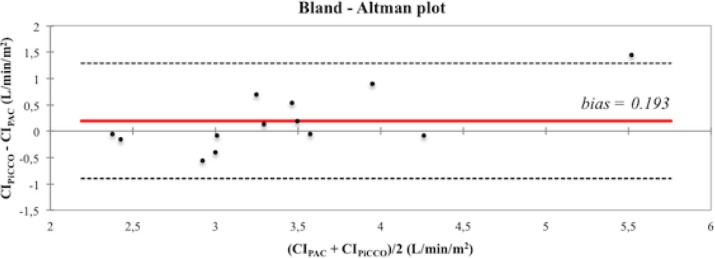
Bland-Altman plot

**Results:** A total of 13 parallel CI measurements were recorded over three days (one patient underwent three determinations on day 3). CI_PAC_ ranged from 2.4 to 4.8 L/min/m^2^ and CI_PiCCO_ from 2.4 to 6.2 L/min/m^2^. Linear regression analysis between CI_PAC_ and CI_PiCCO_ revealed R^2^ = 0.78, p < 0.0001 (Figure 74). From Bland-Altman analysis, the mean differences (m) and SD between CI_PAC_ and CI_PiCCO_ resulted in m = 0.193 L/min/m^2^ and SD = 0.557 L/min/m^2^ (Figure 75).

**Conclusions:** We observed a strong correlation between CI_PAC_ and CI_PiCCO_ (R = 0.89) during veno-venous ECMO, but also a small CI overestimation (0.193 L/min/m^2^; 95%CI: −0.89 - 1.28) of PiCCO over PAC. Due to the wide confidential interval, it follows that PiCCO system can not be considered accurate, at least during ECMO. Further investigations are needed to confirm this preliminary observation.

**References**

[1] Grissom CK, et al. Crit Care Med 2015, 43:288–95;

[2] Della Rocca G, et al. Br J Anaesth 2002, 88:350–6.

### A979 High *versus* low blood-pressure target in experimental ischemic refractory cardiac arrest treated with ECLS

#### C. Fritz^1,2^, A. Kimmoun^1,2^, F. Vanhuyse^2^, B. Trifan^3^, S. Orlowski^1^, E. Albuisson^4^, N. Tran^3^, B. Levy^1,2^

##### ^1^INSERM, U1116, Vandoeuvre-les-Nancy, France; ^2^Ecole de Chirurgie de Nancy, Vandoeuvre-les-Nancy, France; ^3^Ecole de Chirurgie de Nancy, Vandoeuvre les Nancy, France; ^4^CHU de Nancy, Plateforme d'Aide à la Recherche Clinique, Vandoeuvre-les-Nancy, France

###### **Correspondence:** A. Kimmoun – INSERM, U1116, Vandoeuvre-les-Nancy, France

**Introduction:** During initial resuscitation of patients with shock, last consensus recommend to target a mean arterial pressure of at least 65 mmHg. However, there is no recommendation for the mean arterial pressure target in in the particular setting of Extra Corporeal Cardio Pulmonary Resuscitation patients in the first hours following a refractory cardiac arrest.

**Objectives:** Therefore, we conducted an experimental study to assess the effects of two different levels of mean arterial pressure for macrocirculatory, microcirculatory and metabolic functions.

**Methods:** Randomized animal study in university research laboratory. In fourteen male pigs, a myocardial infarction was induced by a surgical ligature of the inter-ventricular coronary artery, triggering a refractory ventricular fibrillation. After twenty minutes of standardised cardiopulmonary resuscitation, Extra Corporeal Life Support was initiated to restore the circulatory flow. Then, animals were randomly allocated to a high mean arterial pressure group (High MAP, 80–85 mmHg) or to a standard mean arterial pressure group (Standard-MAP, 65–70 mmHg). Evaluations at baseline, just before and six hours after ECLS initiation were focused on 1) lactate, 2) amount of fluid infused and 3) microcirculatory parameters (Sidestream Darkfield Imaging, renal and liver functions).

**Results:** The two groups were similar at baseline and also at time of ECSL initiation including for the lactate levels (High MAP : 8.8 [6.7-12.9] *vs* Standard MAP: 9.6 [9.1-9.8] mmol.l^−1^, *p = 0.779*). Lactate levels were also similar between the two groups at six hours (8.9 [4.3-11.1] *vs* 3.3 [2.4-11] mmol.l^−1^, *p = 0.603*). Fluid volume infused was not significantly different between the two groups (4000 [3500–12000] *vs* 5000 [2500–18000] ml, *p = 0.977)*. No significant difference between the two groups was also found for renal and liver functions, sublingual capillary microvascular flow index and the percent of perfused capillaries assessed by Sidestream Darkfield Imaging.

**Conclusions:** Compared to a standard mean arterial pressure regimen, targeting a high mean arterial pressure in the first six hours of an experimental ECPR model, did not result in any hemodynamic, metabolic, microcirculatory improvement or decrease in the amount of fluid infused.

**Grant acknowledgment**

Dr. Fritz received support for article research from “Bourse année recherche” and « Bourse Master II » respectively from the French Ministry of Health and from the French Intensive Care Society (FICS).

### A980 Percutaneous cannulation by intensivists for veno-arterial extracorporeal life support in refractory cardiac arrest: a pilot study

#### V. Chhor, J. Joachim, A. Follin, B. Champigneulle, J. Chatelon, G. Fave, J. Mantz, R. Pirracchio

##### Hopital Europeen Georges Pompidou, Paris, France

###### **Correspondence:** V. Chhor – Hopital Europeen Georges Pompidou, Paris, France

**Introduction:** Refractory cardiac arrest (CA) may benefit from veno-arterial extracorporeal life support (ECLS). While femoral vessels cannulation is usually performed by surgeons, we hypothesized that ultrasound-guided percutaneous technique is also feasible by intensivists (1).

**Objectives:** The goal of our study was to assess the factors associated with percutaneous cannulation success or failure during CA.

**Methods:** This was a prospective observational monocentric study conducted between 2014 May and 2016 February including all consecutive patients with CA (no return to spontaneous circulation after 30 minutes of cardio-pulmonary resuscitation) and an indication of ECLS (low-flow below 90 minutes). Femoro-femoral cannulation (17Fr for arterial cannula and 19Fr for venous cannula, Maquet®) was performed using the Seldinger technique under ultrasound (US) guidance. Patient characteristics, physician's a priori about cannulation conditions (ranging from 1 (expected very easy) to 5 (expected very difficult)) and US measures of femoral vessels diameter were recorded. The primary endpoint was the time to ECLS initiation (ICU admission - ECLS running) and was analyzed using a stepwise multivariable linear regression. As a secondary analysis, we also explored the differences between the patients with a time to ECLS initiation < 20 min and all others (>20 minutes or cannulation failure).

**Results:** 35 patients were included in the study. ECLS cannulation was successfully performed in 29 patients and failed in 6 patients. Median time to ECLS initiation was 20 min [IQR = 17-25].

In the 29 successful cannulations, the time to ECLS initiation was associated with epinephrine total dose (ß = 0.65 95 % CI [0.18 ; 1.12]), use of the Zoll® automatic chest compression system (ß = 18.09 95 % CI [9.09 ; 27.09]), physician's impression (ß = −2.43 95 % CI [−4.33 ; −0.53]) and femoral artery size (ß = −1.18 95 % CI [−2.26 ; −0.10]).

There were 16 patients with a time to ECLS initiation < 20 min and 19 with a time to ECLS initiation >20 min or a cannulation failure. Median time ECLS initiation was 17 min [15–20] and 25 min [24–30] in the 2 groups respectively. The size of the femoral artery was the only significant difference between the 2 groups (8.5 [7.0-9.0] mm *vs* 7.0 [5.0-8.0] mm, p = 0.02).

**Conclusions:** In our cohort of limited size, we have shown that US-guided percutaneous femoral cannulation by intensivists is an effective technique for patients in refractory CA. Epinephrine total dose, physician's impression about cannulation conditions, the type of the automatic chest compression device and the size of femoral artery are associated with the time to ECLS initiation.

**References**

(1) Conrad SA et al. *Crit Care Med. 2015;43:1010–5*

## INTERVENTIONS AND ORGANISATIONAL ISSUES IN ICU

### A981 Outcome of patients with oncological diseases admitted to an ICU

#### D. Díaz Diaz, M. Villanova, M. Aguirregabyria, G. Andrade, L. López, E. Palencia

##### Hospital Universitario Infanta Leonor, Intensive Care Unit, Madrid, Spain

###### **Correspondence:** D. Díaz Diaz – Hospital Universitario Infanta Leonor, Intensive Care Unit, Madrid, Spain

**Objectives:** To analyse the evolution and outcome of patients with oncological diseases admitted to an ICU.

**Methods:** Retrospective study including consecutive patients with prior diagnosis of oncological diseases. We register type of cancer, location, degree of extension, quality of life by means of the Eastern Cooperative Oncology Group (ECOG) scale, severity at admission (SAPS3, APACHE-II, SOFA), days of stay ad the ICU, treatments administered and mortality.

**Results:** We included 50 patients (males 62 %; mean age 68.7 years). 90 % was presenting a solid tumour, being the most frequent those of digestive origin (n = 23; 46 %). Among the hematologic tumours the most frequent was the lymphoma (n = 4; 90 %). A total of 42 patients (84 %) were in phase I (potentially treatable) and 6 in phase II (palliative care). Fifteen patients (30 %) presented with metastasis. The main reason for ICU admission was the surgical treatment of the tumour (n = 19; 38 %), followed by septic shock (n = 12; 24 %). Clinical severity at ICU admission: SAPS3 63 (SD14), APACHE-II 19 (SD 7), SOFA 4 (SD 3). A total of 32 patients (64 %) needed mechanical ventilation. The average stay in the ICU was 4.1 days (SD 5.6), and the in-hospital total length of stay was 24 days (SD 23.2). Five patients (10 %) died at the ICU and other three during their stay in the hospital. The factors related to the mortality were: respiratory chronic disease, neutropenia and septic shock. Mean ECOG at hospital discharge was 2 (independence for the majority of personal needs, without being able to recover job activity due to the presence of symptoms needing to remain at bed less than 50 % of the day).

**Conclusions:** The main reason for admission of patients with oncological diseases to an ICU is the surgical treatment of the tumour. They have low mortality rates and an acceptable quality of life at hospital discharge. The factors related to the mortality were: respiratory chronic disease, neutropenia and septic shock.

### A982 Quantifying baby and mother contact during advanced maternity care for critically unwell mothers

#### G. John, R. Cowan, R. Hart, K. Lake, K. Litchfield

##### Glasgow Royal Infirmary, Anaesthesia, Glasgow, United Kingdom

###### **Correspondence:** G. John – Glasgow Royal Infirmary, Anaesthesia, Glasgow, United Kingdom

**Introduction:** Postpartum contact between neonates and their mothers is an important part of early development and is strongly advocated by neonatal and obstetric practitioners. Unfortunately, critical illness and admission to critical care areas of a hospital can interrupt this contact. In the United Kingdom the minimum standards of Advanced Maternity Care (AMC) are approaching completion. One of these standards is expected to be baby-mother contact. In Scotland, many Obstetric units are joining the Scottish Intensive Care Society Audit Group database “Wardwatcher” to better benchmark critical care performance against nationally agreed standards.

**Objectives:** Quantify baby being with mother postpartum, what level of care is provided, if level of care affects the proportion of babies with their mothers and examine maternal outcome overall.

**Method.** Princess Royal Maternity unit at Glasgow Royal Infirmary, has kept Wardwatcher data since 5th July 2015. A local addition to the Wardwatcher database to document newborns being with their mother when already an AMC patient has been made. Data extracted 8^th^ April 2016 and analysed using a combination of R and Excel statistical software.

**Results:** 157 admissions with 332 patient calendar days.

Pregnancy status: 27 antepartum, 4 delivered during AMC and 126 postpartum.

Baby with mum per admission: delivered during AMC, 2/4 (50 %); postpartum admission, 87/126 (69.0 %); overall, 89/130 (68.5 %).

Level of AMC and baby with mum per admission: level 0, 7/12 (58.3 %); level 1, 26/42 (61.9 %); level 2, 56/76 (73.7 %).

Maternal outcome overall: improvement, 121/130 (93.1 %); no change, 6/130 (4.6 %); worse, 3/130 (2.3 %). No deaths.

Analysis of Baby with mother combined with maternal outcome:

- out of 89 admissions with baby-mother contact, outcome: improved, 86 (96.6 %); no change, 1 (1.1 %).
- out of 41 admissions without baby-mother contact, outcome: improved, 35 (85.4 %); no change 5 (12.2 %).

Analysed per patient calendar day: patients delivering during admission, 10 days, 8 after delivery, 4 with baby (50 %); postpartum patients 156/258 days (60.5 %).

**Conclusions:** Our data suggests that the level of care patients receive does not affect baby being with its mother. However, level 3 patients are admitted to a general critical care unit and not captured in this review. The results also suggest that maternal well-being is affected by and/or affects whether baby is with her, however the 95 % confidence intervals overlap. The above data does not take into account babies being in Special Care Baby Units and therefore not able to join their mothers at their bedside (this data will be recorded in the future). When this data is recorded it is expected that a high proportion of babies who are able to be at their mother's bedside during AMC will be there.

### A983 The change of clinical outcomes in patients with advanced lung cancer who admitted to medical intensive care unit after intensivist referral

#### J.W. Song, Y.J. Lee, Y.-J. Cho, S. Choi

##### Seoul National University Bundang Hospital, Internal Medicine, Seongnam, Republic of Korea

###### **Correspondence:** Y.-J. Cho – Seoul National University Bundang Hospital, Internal Medicine, Seongnam, Republic of Korea

**Introduction:** Lung cancer is the leading cause of intensive care unit (ICU) admission in patients with advanced solid tumor.

**Objectives:** This study was aimed to elucidate the clinical factors associated with ICU mortality of advanced lung cancer patients and the effect of intensivist's contribution on their clinical outcomes since the introduction of organized intensive care.

**Methods:** From 2003 to 2015, patients with advanced lung cancer including non-small cell lung cancer (NSCLC) with stage IIIB or IV and small cell lung cancer (SCLC) with extensive stage who admitted to ICU were included and reviewed. Multivariate logistic regression analysis was performed to find the variables including intensivist referral associated with 30-day ICU mortality.

**Results:** Respiratory failure was the primary cause of ICU admission in advanced lung cancer patients (n = 167, 86 %). 30-day ICU mortality and hospital mortality were lower in the group of intensivist referral, but no statistically significant difference between two groups. Age, gender adjusted multivariate analysis, intensivist referral [OR 0.32, 95 % CI 0.13 - 0.77, *p*-value = 0.01] was associated with low 30-day ICU mortality. Also in sepsis group (n = 58) [OR 0.00, 95 % CI 0.00 - 0.41, *p*-value = 0.03] and acute respiratory failure group (n = 167) [OR 0.36, 95 % CI 0.14-0.92, *p*-value = 0.03], intensivist referral was statistically significant in reduction of 30-day ICU mortality.

**Conclusions:** In patients with advanced lung cancer, the organized care by intensivists could be contributable to improve clinical outcomes. In the subgroup of sepsis and acute respiratory failure, the intensivist referral was associated with lower mortality.

**References**

1. Puxty K, McLoone P, Quasim T, Kinsella J, Morrison D. Survival in solid cancer patients following intensive care unit admission. *Intensive Care Med.* 2014;40(10):1409–1428.

2. Kim YJ, Kim MJ, Cho YJ, et al. Who should be admitted to the intensive care unit? The outcome of intensive care unit admission in stage IIIB-IV lung cancer patients. *Medical oncology (Northwood, London, England).* 2014;31(3):847.

3. Azevedo LCP, Caruso P, Silva UVA, et al. Outcomes for patients with cancer admitted to the icu requiring ventilatory support: Results from a prospective multicenter study. *Chest.* 2014;146(2):257–266.

4. Soubani AO, Ruckdeschel JC. The outcome of medical intensive care for lung cancer patients: the case for optimism. *Journal of thoracic oncology : official publication of the International Association for the Study of Lung Cancer.* 2011;6(3):633–638.

**Grant acknowledgment**

None

### A984 Communication satisfaction and job satisfaction among critical care nurses and the impact on burnout and turnover intention

#### P. Vermeir^1^, D. Vandijck^2^, S. Blot^3^, A. Mariman^4^, R. Verhaeghe^2^, M. Deveugele^5^, D. Vogelaers^6^

##### ^1^Ghent University Hospital, General Internal Medicin, Gent, Belgium; ^2^Ghent University, Public Health, Gent, Belgium; ^3^Ghent University, Internal Medicin, Gent, Belgium; ^4^Ghent University, General Internal Medicine, Gent, Belgium; ^5^Ghent University, Family Medicine and Primary Health Care, Gent, Belgium; ^6^Ghent University Hospital, Internal Medicine, Gent, Belgium

###### **Correspondence:** P. Vermeir – Ghent University Hospital, General Internal Medicin, Gent, Belgium

**Introduction:** High levels of job satisfaction are associated with decreased turnover intention, burnout incidence and absenteeism among health care professionals. Moreover, turnover and burnout negatively impact on quality of care and healthcare costs. As the intensive care unit (ICU) represents a highly complex and stressful environment, prevention of conflicts among team members as well as improvement of communication and job satisfaction can as such reduce burnout risk.

**Objectives:** This study explores the relationship between communication - and job satisfaction and the impact on burnout and turnover intention among ICU nurses.

**Methods:** In a multicentre study, ICU nurses of three hospitals were included (N = 303). Data included the Communication Satisfaction Questionnaire (Downs & Hazen, 1977) (translated in Dutch and validated by a factor analysis and pilot tested), the scale 'Turnover intention' of the Questionnaire on the Experience and Evaluation of Labour (Van Veldhoven & Meijman, 1994) and the Maslach Burnout Inventory (Maslach et al., 1996). To measure job satisfaction a visual analogue scale was used.

**Results:** 77.6 % (235/303) of the respondents were female. The ICU nurses had an average age of 38.31. Their mean work experience was 15.28 years. The majority worked fulltime (60.1 %, 182/303). An average job satisfaction of 7.66/10 was found. 5.33 % (16/300) had a score ≤ 5 on job satisfaction, indicating significant dissatisfaction. ICU nurses were most satisfied with the trust received from their supervisor (76.6 %) and least with the information about accomplishments and/or failures of the organization (21.8 %). 49.5 %, (150/290) had a low, 39.6 % (120/290) an average and only 6.6 % (20/290) a high turnover intention. 3 % of the ICU nurses had an indication for burnout. 23.7 % of the nurses had a low experience of their personal accomplishment.

**Conclusions:** In this survey ICU nurses had a reasonable job satisfaction. They are most satisfied with the trust received from supervisors. Despite a low indication for burnout risk, a quarter of ICU nurses report low personal accomplishment. This may represent a particular focus for both preventive and interventional actions, which should preferably be developed through and in conjunction with the supervising staff.

**References**

1. Aiken, L. H., Sermeus, W., Van den Heede, K., Sloane, D. M., Busse, R., McKee, M., … & Tishelman, C. (2012). Patient safety, satisfaction, and quality of hospital care: cross sectional surveys of nurses and patients in 12 countries in Europe and the United States. Bmj, 344, e1717.

2. McHugh, M. D., Kutney-Lee, A., Cimiotti, J. P., Sloane, D. M., & Aiken, L. H. (2011). Nurses' widespread job dissatisfaction, burnout, and frustration with health benefits signal problems for patient care. Health Affairs, 30(2), 202–210.

### A985 Effect of diagnosis related groups implementation on the intensive care unit of a Swiss tertiary hospital: a cohort study

#### L. Chok^1^, E.B. Bachli^2^, D. Bettex^3^, S.R. Cottini^4^, E. Keller^5^, M. Maggiorini^6^, R. Schuepbach^7^

##### ^1^University Hospital Zurich, University Zurich, Zürich, Switzerland, ^2^Hospital Uster, Department of Internal Medicine, Uster, Switzerland, ^3^University Hospital Zurich, University Zurich, Heart and Vascular Surgical Intensive Care Unit, Zurich, Switzerland, ^4^University Hospital Zurich, University Zurich, Surgical Intensive Care Unit, Zurich, Switzerland, ^5^University Hospital Zurich, University Zurich, Neurosurgical Intensive Care Unit, Zurich, Switzerland, ^6^University Hospital Zurich, University Zurich, Medical Intensive Care Unit, Zurich, Switzerland, ^7^University Hospital Zurich, University Zurich, Surgical Intensive Care Unit, Zürich, Switzerland

###### **Correspondence:** R. Schuepbach – University Hospital Zurich, University Zurich, Surgical Intensive Care Unit, Zürich, Switzerland

**Introduction:** In 2013 the Swiss Diagnosis Related Groups ((Swiss)-DRG)) was implemented in Intensive Care Units (ICU). Its impact on hospitalizations has not yet been thoroughly examined. We compared the number of ICU admissions according to clinical severity and referring institution, screened whether implementation of SwissDRG affected admission policy, ICU length-of-stay (LOS) or ICU mortality.

**Methods:** Retrospective single center cohort study conducted at the University Hospital Zurich, Switzerland between January 2009 and end of 2013. Demographic and clinical data were retrieved from a quality assurance data base.

**Results:** Admissions (17231) before introduction of SwissDRG were used to model expected admissions after DRG and compared to admissions observed. Forecasting matched observations in patients with a high clinical severity admitted from internal units and external hospitals (admitted/predicted: 709/703, [95 % CI, 658–748] and 302/332, [95 % CI, 269–365] respectively). In low severity of disease patients in-house admissions become more frequent than expected whereas external admission were less frequent (admitted/predicted: 1972/1910, [95 % CI, 1898–1940] and 436/518, [95 % CI, 482–554] respectively). In regard to LOS and ICU mortality DRG could not be linked to make significant changes.

**Conclusions:** DRG introduction had not affected ICU admissions policy, except for an increase of in-house patients with a low clinical severity of disease. DRG had neither affect ICU mortality nor ICU LOS.

### A986 Interactive gaming as part of mobilisation programs is feasible in the ICU, but specific explanation about the usefulness of these games to patients is crucial for improving motivation and engagement

#### T. Fiks^1^, C. Stiphout^1^, M. Grevelink^1^, I. Vaneker^1^, A. Ruijter^2^, M. Buise^3^, P.E. Spronk^1,4^

##### ^1^Gelre Hospitals Apeldoorn, Intensive Care Medicine, Apeldoorn, Netherlands; ^2^Gelre Hospitals Apeldoorn, Physiotherapy, Apeldoorn, Netherlands; ^3^Catharina Hospital, Intensive Care Medicine, Eindhoven, Netherlands; ^4^Academic Medical Center, University of Amsterdam, Intensive Care Medicine, Amsterdam, Netherlands

###### **Correspondence:** P.E. Spronk – Gelre Hospitals Apeldoorn, Intensive Care Medicine, Apeldoorn, Netherlands

**Introduction:** In recent years, light sedation has gained attention as part of standard daily care in the intensive care unit (ICU). Consequently, patients are increasingly engaged in their rehabilitation process. Particularly early mobilization is associated with shorter time on the ventilator, shorter ICU length of stay and better survival [1]. Interactive gaming may be a challenging way of engaging the patient in his own rehabilitation program. Few data are available for the use of these interactive games in the ICU envirnoment as part of daily routine physiotherapy, although one study showed that it was safe [2].

We developed a trolley with a Wii^(TM)^ device that can be easily used when the patient is mobilized in a chair. We hypothesized that this would be associated with increased motivation to participate in interactive gaming by our patients.

**Methods:** The Wii device was used with 4 different games. Participating patients were offered to play 2 games of their own choice as part of the mobilisation program to improve their strength and coordination. No extensive explanation about the potential usefulness of these interactive games was given to the patients. After finishing the games, a specific survey was administered addressing motivation and affects on mental health. Scores were obtained using a Likert scale (range 1–7). Results are shown as median and interquartile range {P_25_-P_75_]

**Results:** At the time of abstract submission, 13 participating patients had finished a cycle of 2 games. Some of the patients liked to use the Wii device, particularly because a choice in games made it more interesting to use. Other patients, however, felt they were required to participate. Tennis, bowling and boxing were most frequently used. The use of the Wii was programmed in the daily mobilization schedule together with a physiotherapist, or just with the attending ICU nurse. In general, patients were not that enthusiastic about the Wii-games (median score 2 [1–2]), were not convinced that playing these games improved their well-being (median score 2 [1–4]), and most felt that they did not have a choice but to participate (median score 6 [5–7]).

**Conclusion:** Interactive gaming with the Wii-device is feasible in ICU patients. However, thorough explanation of the potential usefulness of these games is required to engage and motivate patients to participate.

**References**

1. Early physical and occupational therapy in mechanically ventilated, critically ill patients: a randomised controlled trial. Schweickert WD, Pohlman MC, Pohlman AS, Nigos C, Pawlik AJ, Esbrook CL, Spears L, Miller M, Franczyk M, Deprizio D, Schmidt GA, Bowman A, Barr R, McCallister KE, Hall JB, Kress JP. Lancet. 2009 May 30;373(9678):1874–82

2. Feasibility and observed safety of interactive video games for physical rehabilitation in the intensive care unit: a case series. Kho ME, Damluji A, Zanni JM, Needham DM. J Crit Care. 2012 Apr;27(2):219.e1-6

### A987 Hospital mortality reduction after the establishment of rapid response team (RRT)

#### S. Altaba Tena, L. Galarza Barrachina, J.H. Rodriguez Portillo, G. Pagés Aznar, L. Mateu Campos, M.D. Ferrándiz Sellés, M. Arlandis Tomás, A. Belenguer Muncharaz

##### General University Hospital of Castellon, Castellón, Spain

###### **Correspondence:** S. Altaba Tena – General University Hospital of Castellon, Castellón, Spain

**Objective:** Determine if the implantation of Rapid Response Team (RRT) reduce the hospital mortality after ICU discharge.

**Methods:** Descriptive and retrospective study. We include all patients admitted in ICU during 2012 (previous to RRT establishment) and 2015 (year of RRT establishment), and who were discharged to the ward. We analysed the ICU and hospital mortality in both groups.

**Results:** In 2012 we admitted 1296 patients (17,3 % from the ward and 82,7 % from the other places like Emergency, other ICU or other hospitals), with a ICU mortality of 10,4 % and hospital mortality of 15,55 %, with a hospital mortality after ICU discharge of 5,69 %. In 2015 we admitted 1307 patients (14,6 % from the ward and 85,4 % from the other places), with a ICU mortality of 12,6 % and hospital mortality of 15,68 %, and a hospital mortality after ICU discharge of 3,5 % (p = 0.03).

**Conclusions:** After the first year of RRT establishment in our hospital, we appreciate that the continuation of patients after ICU discharge, decrease the hospital mortality ( 5,69 % versus 3,5 %, p = 0.03). The number of patients admitted in ICU from the ward decrease in 2015 (14,6 % versus 17,3 %), maybe because we did a previous assessment of this patients, with a stabilization in the ward and avoiding the ICU admission.

### A988 Outcomes and prognostic factors in haematological malignancy patients admitted to a general intensive care unit over a 5-year period

#### L. Skinner^1^, S. Monsalvo^2^, E. Olavarria^2^, R. Stümpfle^1^

##### ^1^Imperial College Healthcare NHS Trust, General Intensive Care Unit, London, United Kingdom; ^2^Imperial College Healthcare NHS Trust, Haematology, London, United Kingdom

###### **Correspondence:** R. Stümpfle – Imperial College Healthcare NHS Trust, General Intensive Care Unit, London, United Kingdom

**Introduction:** Critically ill haematological malignancy (HM) patients admitted to intensive care units (ICU) are known to have poor prognoses. Mechanical ventilation, multiple organ failures and severe acute critical illness are factors associated with mortality. Current literature suggests review of ceilings-of-care on day 5 of ICU admission^1^.

**Objectives:** To identify outcomes and prognostic factors in HM patients admitted over 5-years to a general intensive care unit in a specialist haematology centre.

**Methods:** We retrospectively reviewed HM patients admitted to ICU at our institution between 2010–2015. Data were available on demographics, reason for ICU admission, APACHE II, SOFA and SAPS II scores, underlying HM and treatment: autologous, allogenic transplant (HSCT) or chemotherapy. Laboratory values and organ support requirements were recorded on ICU admission. Univariate anaylsis was used to compare outcome groups.

**Fig. 76 (abstract A988).**
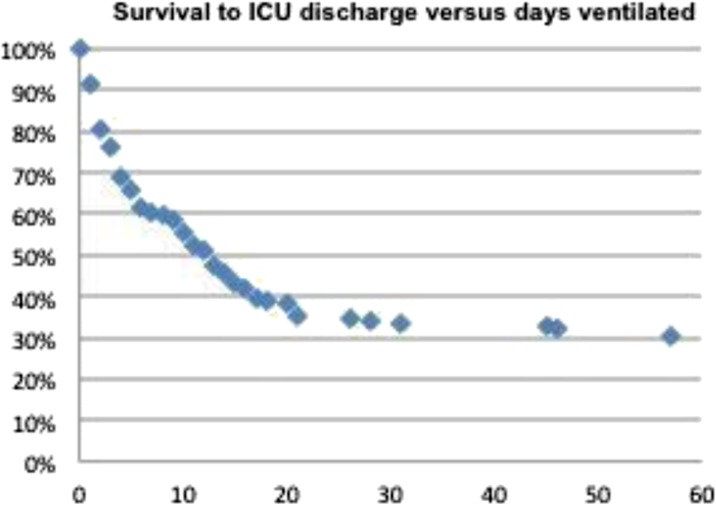
Survival to ICU discharge in ventilated patients

**Results:** We identified 191 ICU admissions from a cohort of 180 HM patients. The median age was 55 years (range 22–85 years). 40.5 % were female. Average duration of organ support was 8.69, 5.97, 1.96 days for respiratory, renal and cardiovascular support, respectively. Average ICU stay was 12.8 days. Reasons for admission were: sepsis, 73 %; cardiac insufficiency, 4 %; renal failure, 3.3 %; haemorrhage, 1.7 %; status epilepticus 1.1 %; cardiac arrest, 1.1 %; intervention, 1 %; and other, 15 %. 33 % were post-HSCT: 8 % autologous, 25 % allogenic. ICU mortality was 61 % and in-hospital mortality was 72 %. 13 % of patients had >1 ICU admissions. In-hospital mortality in ventilated patients was significantly greater than that of non-ventilated patients (p < 0.0001; 82 % vs. 36 %), OR 8.6 [4.2 - 16.9; CI 95 %]. ICU mortality was 83 % in those ventilated for 1–4 days and 69 % in those ventilated for ≥5 days (Figure 76). Patients with 3–4 organ failures had significantly worse outcomes than those with 1–2 organ failures (p < 0.0001) OR 13.4 [6.7 - 25.0, CI 95 %]. Median APACHE II, SOFA and SAPS II scores were 24, 10 and 48, respectively. Median APACHE II (p < 0.05), SOFA (p < 0.04) and SAPS II (p < 0.001) scores were greater in those ventilated for 1–4 days vs. ≥5 days.

**Conclusions:** APACHE II scores and mortality were greater than described in similar HM populations. Given the severity of critical illness in our cohort, we suggest that admission to ICU earlier in the acute illness may improve outcomes. Poorer outcomes were observed in those with >2 organ failures and in ventilated patients. The survival of 1/3^rd^ of patients on ICU for ≥5 days to hospital discharge suggests that 5-day trials of ICU in HM patients are unlikely to reliably distinguish between survivors and non-survivors.

**References**

1. Wise M, Barnes R, Baudouin S et al. Guidelines on the management and admission to intensive care of critically ill adult patients with haematological malignancy in the UK. British Journal of Haematology. 2015;171(2):179–188

### A989 An association between a cardiac intensivist and mortality in adult cardiac intensive care unit

#### S.J. Na, J. Park, C.R. Chung, C.M. Park, G.Y. Suh, J.H. Yang

##### Samsung Medical Center, Seoul, Republic of Korea

###### **Correspondence:** S.J. Na – Samsung Medical Center, Seoul, Republic of Korea

**Background:** Dedicated intensive care unit (ICU) physician staffing was associated with a reduction of ICU mortality in the general medical and surgical ICU. However, limited data were available on the role of a cardiac intensivist in the cardiac intensive care unit (CCU). We compared the clinical outcomes in adult patients admitted to CCU before and after implementing the cardiac intensivist-directed care.

**Methods:** We enrolled 2,923 consecutive patients admitted to a CCU at Samsung Medical Center, from January 2012 to December 2015. In January 2013, CCU was changed from a low-intensity staffing model to high-intensity staffing model which managed by a dedicated cardiac intensivist. We divided eligible patients into low-intensity group (n = 616) and high-intensity group (n = 1,815). The primary outcome was CCU mortality.

**Results:** High-intensity group had significantly lower CCU (8.9 % vs 4.1 %; p < 0.001) and hospital (10.9 % vs 6.1 %; p < 0.001) mortality compared to the low-intensity group. The decrease in CCU (54.5 % vs 22.5 %; p = 0.001) and hospital (57.6 % vs 29.4 %; p = 0.003) mortality in high-intensity group were consistent in 135 (low-intensity group 33, high-intensity group 102) patient with profound cardiogenic shock treated with extracorporeal membrane oxygenation. Kaplan-Meier survival curve showed significant higher cumulative survival rates in high-intensity group at 1 year follow-up (Log rank test, p < 0.001). CCU (7.6 % vs 5.8 %; p = 0.101) and hospital re-admission rate (7.5 % vs 6.0 %; p = 0.179) were decreased as well after conversion to high-intensity although these results were not statistically significant.

**Conclusions:** Dedicated cardiac intensivist was associated with reductions of CCU mortality in patient with cardiovascular disease requiring critical care.

### A990 Prolonged chronic use of opioids: a comparison between the critically ill and surgical populations

#### T. Witter^1,2^, C. Brousseau^3^, M.B. Butler^1^, M. Erdogan^4^, P.C. Mac Dougall^2^, R.S. Green^1,4^

##### ^1^Dalhousie University, Critical Care Medicine, Halifax, Canada; ^2^Dalhousie University, Anesthesiology, Pain Management and Perioperative Medicine, Halifax, Canada; ^3^Dalhousie University, Respiratory Therapy, Halifax, Canada; ^4^Dalhousie University, Emergency Medicine, Halifax, Canada

###### **Correspondence:** T. Witter – Dalhousie University, Critical Care Medicine, Halifax, Canada

**Introduction:** Opioids are commonly given to alleviate pain and distress in patients admitted to the intensive care unit (ICU) patients or undergoing major surgery. Previous studies have shown that patients who are already taking opioids prior to surgery or ICU admission are more likely to experience an extended duration of opioid use postoperatively or post-discharge (1). However, it is unknown whether patterns of opioid usage differ between patients who are admitted to the ICU and those undergoing a surgical procedure.

**Objectives:** The objective of this study was to describe opioid use in critically ill patients before and after ICU admission and to compare it with preoperative and postoperative opioid use in a surgical population.

**Methods:** Retrospective review and comparison of adult patients admitted to the ICU or undergoing surgery at a tertiary care center between January 1, 2006 and December 31, 2008. We divided the populations based on their degree of opioid use into “non-user”, “intermittent”, and “chronic” opioid users as previously described (1). We assessed opioid use at 3 months prior to ICU admission or surgery, at discharge, and monthly for 6 months thereafter. Patients admitted to ICU who had surgery were categorized under the ICU population. To assess for risk of monthly chronic opioid use, a Cox-Proportional Hazards model was postulated that allowed for recurrent events to account for patients irregularly requiring opioids over the course of the study period.

**Results:** A total of 1684 ICU patients and 16736 surgical patients were included in the analysis. Prior to admission, the ICU group included 1274 (75.7 %) non-users, 390 (23.2 %) intermittent users, and 20 (1.2 %) chronic opioid users. Prior to their procedure, the surgical group included 12057 (72.0 %) non-users, 3611 (21.6 %) intermittent users, and 1068 (6.4 %) chronic opioid users. At discharge, 3.3 % (55/1684) of ICU patients and 5.7 % (956/16736) of surgery patients were chronically using opioids. At 6 months post-discharge, the number of chronic opioid users in the ICU population increased to 91 (5.4 %), while the number of chronic users in the surgical group increased to 1062 (6.2 %). The model showed that the risk of chronic opioid use was 52.2 times greater for those with prior chronic opioid use compared to patients who were non-users. There was no difference in risk of chronic opioid use between the ICU and surgery group.

**Conclusions:** Our findings suggest that ICU and surgical patients have similar risk of prolonged chronic opioid use post-discharge. Chronic opioid use prior to ICU admission or surgery is the strongest predictor of chronic opioid usage at and after discharge.

**References**

1. Yaffe P., Green R., Butler M, Witter T. Is Admission to the Intensive Care Unit Associated With Chronic Opioid Use? A 4-Year Follow-Up of Intensive Care Unit Survivors. J Intensive Care Med. 2015 Nov 25

### A991 A single-centre cohort study of national early warning score (NEWS) and blood gas derived biomarkers in patients with acute medical illness

#### T.E.F. Abbott^1^, H.D.T. Torrance^1^, N. Cron^2^, N. Vaid^3^, J. Emmanuel^4^

##### ^1^Queen Mary University of London, William Harvey Research Institute, London, United Kingdom; ^2^London School of Economics, London, United Kingdom; ^3^Northwick Park Hospital, London, United Kingdom; ^4^Barts Health NHS Trust, London, United Kingdom

###### **Correspondence:** T.E.F. Abbott – Queen Mary University of London, William Harvey Research Institute, London, United Kingdom

**Introduction:** The utility of an early warning score may be improved when used with near patient testing.^1^ However, this has not yet been investigated for National Early Warning Score (NEWS).

**Objectives:** We hypothesised that the combination of NEWS and blood gas variables (lactate, glucose or base-excess) was more strongly associated with clinical outcome compared to NEWS alone.

**Methods:** This was a prospective cohort study of adult medical admissions to a single UK centre over 20-days. Blood gas results and physiological observations were recorded at admission. NEWS was calculated retrospectively and combined with the biomarkers in multivariable logistic regression models. The primary outcome was a composite of mortality or critical care escalation within 2 days of hospital admission. The secondary outcome was hospital length of stay. The study was reviewed and approved by the National Research Ethics Service (12/LO/1985).

**Results:** After accounting for missing data, 15 patients out of 330 (4.5 %) died or were escalated to the critical care unit. The median length of stay was 4 (IQR 1–8) days. When combined with lactate NEWS was associated with the primary outcome (OR 1.13, p = 0.03). However, NEWS alone was more strongly associated with the primary outcome measure (OR 1.34, p < 0.01). Combinations of NEWS with glucose or base excess were not associated with the primary outcome. Neither NEWS nor any combination of NEWS and a biomarker were associated with hospital length of stay.

**Conclusions:** Admission NEWS is more strongly associated with death or critical care unit admission within 2 days of hospital stay, compared to combinations of NEWS and blood-gas derived biomarkers.

**References**

1. Jo S, Lee JB, Jin YH, Jeong TO, Yoon JC, Jun YK, et al. Modified early warning score with rapid lactate level in critically ill medical patients: the ViEWS-L score. Emergency medicine journal : EMJ. 2013;30:123–9.

**Grant acknowledgment**

TA is a MRC/BJA Clinical Research Training Fellow.

### A992 Evaluation and validation of the four scoring systems; the acute physiology and chronic health evaluation (APACHE) IV, simplified acute physiology score (SAPS) 3, mortality probability model (MPM) 0-II and ICU cancer mortality model (ICMM) in critically ill cancer patients

#### S.S. Siddiqui, N. Prabu, H.K. Chaudhari, V.P. Patil, J.V. Divatia, S. Solanki, A.P. Kulkarni

##### Tata Memorial Hospital, Anaesthesia, Critical Care and Pain, Mumbai, India

###### **Correspondence:** S.S. Siddiqui – Tata Memorial Hospital, Anaesthesia, Critical Care and Pain, Mumbai, India

**Introduction:** Data on performance of recent versions of ICU mortality prediction scores in critically ill cancer patients is scarce.

**Objectives:** To evaluate and validate four prognostic scoring systems namely, the Simplified Acute Physiology Score (SAPS) 3, the Mortality Probability Model II at 0 hours (MPM_0_ II), Acute Physiology and Chronic Health Evaluation IV and ICMM [1], in a prospective cohort of critically ill cancer patients.

**Methods:** The study was performed in a 14 bed combined medical-surgical ICU of a tertiary care cancer centre of India, from July 2014 to June 2015. All adult patients (>18 years age) with cancer who stayed in ICU for > 24 hours were included. The most recent admission was considered in case of multiple hospital admissions and in patients requiring readmission to the ICU during the same hospital stay, only the first ICU admission was considered. Patients with burn injury, acute coronary syndrome, bone marrow transplant or those free of cancer for > 5 years and those with ICU stay < 24 hrs were excluded. Variables relevant to aforementioned scores were collected at admission within ±1 hour and over the first 24 hours of ICU admission. Score performance was judged by assessing discrimination and calibration and using the area under receiver operating characteristics (ROC) curve and by the Hosmer Lemeshow goodness-of-fit test, respectively.

**Results:** 322 patients (243 solid organ and 79 haematolymphoid malignancies) were included in the study. Predominantly were admitted to ICU for medical 218 (67.7 %) reasons. Most common reason for ICU admission was acute respiratory failure 85 (26.4 %).Invasive mechanical ventilation (IMV) was required in 228 (70.8 %), non-invasive ventilation (NIV) was required in 74 (23 %) and 40 (12.4 %) patients needed intubation due to failure of NIV. Vasopressors were required in 161 (50 %) and dialysis in 18 (5.6 %) patients; most patients received multiple ICU interventions. ICU and hospital mortality were 37.9 % and 41.6 % respectively. The mean SAPS 3 and APACHE IV scores were 59.8 ± 12.76 and 71.53 ± 24.97 respectively. Predicted mortality and SMR of each score is given in Table 52. The area under ROC curve for SAPS 3, MPM0 II, ICMM and APACHE IV models were 0.70, 0.673. 0.675 and 0.74 respectively (Graph 1). Calibration as calculated by Hosmer and Lemeshow analysis type C statistics, found that APACHE IV, SAPS 3, MPM0 II and ICMM show good calibration with Chi square values of 10.437, 10.148, 7.7 and 11.087 and p values of 0.236, 0.255, 0.463 and 0.197 respectively.

**Conclusions:** APACHE IV, SAPS 3, ICMM and MPM0 II had moderate discrimination and good calibration. However, none of the mortality prediction models could accurately discriminate between survivors and non-survivors in our patient population. Specifically designed model like ICMM did not seem to have any advantage over other general ICU mortality prediction models.

**References**

1. Groeger JS et al. J Clin Oncol 1998; 16: 761–770.

**Table 52 (abstract A992).** Predicted Mortality and SMR

	Predicted mortality	Lower limit of 95 % confidence interval	Upper limit of 95 % confidence interval	Standardised Mortality Ratios (SMR)
MPM0 II	95.16	1.179	1.667	1.408
SAPS 3	118.94	0.944	1.334	1.127
ICMM	144.97	0.774	1.095	0.9243
APACHE IV	96.24	1.167	1.649	1.392

**Fig. 77 (abstract A992).**
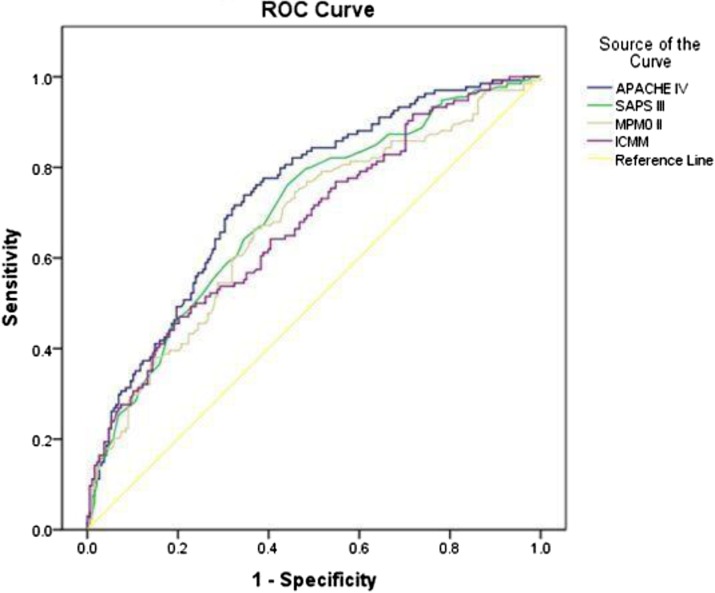
Receiver operating characteristics (ROC) curves

### A993 Validation of save score in more than 100 patients treated with VA-ECMO

#### L.A. Rincon Gutierrez^1,2^, A. Bader^1^, A. Brasseur^1^, O. Lheureux^1^, J.L. Vincent^1^, J. Creteur^1^, F.S. Taccone^1^

##### ^1^Erasme University Hospital, Université Libre de Bruxelles, Department of Intensive Care, Brussels, Belgium; ^2^Insituto Mexicano del Seguro Social, Internal Medicine, Celaya, Mexico

###### **Correspondence:** L.A. Rincon Gutierrez – Erasme University Hospital, Université Libre de Bruxelles, Department of Intensive Care, Brussels, Belgium

**Background:** Veno-Arterial Extracorporeal Membrane Oxygenation (VA-ECMO) is commonly used in some centers to treat refractory cardiogenic shock. Considering the costs and resources, selection is important. Recently, the SAVE score has been developed to predict mortality in VA-ECMO patients but has been validated only in one cohort of patients (European Heart Journal (2015) 36, 2246–2256).

**Objective:** To compare the usefulness of SAVE score with the MELD-XI, SAPS II, APACHE II and SOFA scores in VA-ECMO patients.

**Methods:** We reviewed our institutional database of VA-ECMO from November 2008 to September 2015 (n = 179), including those with age more than 18 years old and baseline available data. We collected patients´ demographics and clinical data as well as additional scores (SAPS II, APACHE II, SOFA and MELD-XI scores) on the day of ECMO insertion. Also, blood lactate levels and the cardiovascular SOFA score, were collected on the onset of ECMO therapy. The sensitivity and specificity of variables retained by a multivariable analysis as being associated with neurological outcome was evaluated using receiving operating characteristic (ROC) curves with the corresponding area under the curve (AUC).

**Results:** Of the 179 patients, 62 were excluded (out-of-hospital cardiac arrest -n = 19; early death - n = 43; already on ECMO from another hospital - n = 5) and 112 were analyzed (age 55[46–63] years; 77/112 male). Overall mortality was 66/112 (59 %). Median SAVE score was −5 [−7 to −2], and APACHE II, SAPS II, SOFA and MELD-XI scores were 27 [24–31], 71 [64–80], 13 [11–14] and 15 [11–20], respectively. Lactate levels were 5.3 [2.8-9.3] mmol/L. SAVE scores, SAPS II, APACHE II scores and baseline lactate levels had the highest AUCs to predict mortality (0.74 [0.65-0.83] vs. 0.77 [0.69-0.86] vs. 0.68 [0.58-0.78] vs. 0.70 [0.61-0.80, respectively). AUCs were significantly lower for cardiovascular SOFA score and MELD-IX.

**Conclusions:** The SAVE-score had similar predictive value for mortality in VA-ECMO patients than other non-specific scores or baseline lactate levels. The combination of SAVE score with other clinical relevant variables should be further evaluated.

### A994 Two-point-compression ultrasound to diagnose DVT a randomized, controlled educational trial

#### D. Hempel^1^, N. Stauffert^2^, F. Recker^3^, T. Schröder^4^, S. Reusch^5^, J. Schleifer^3^, R. Breitkreutz^6^

##### ^1^University Hospital Jena, Department of Internal Medicine IV, Jena, Germany; ^2^University of Frankfurt, Frankfurt, Germany; ^3^University Bonn, Bonn, Germany; ^4^Hospital Frankfurt Hoechst, Frankfurt, Germany; ^5^KfH Bad Soden, Bad Soden, Germany; ^6^Ultrasound Network in Acute and Critical Care, Frankfurt, Germany

###### **Correspondence:** D. Hempel – University Hospital Jena, Department of Internal Medicine IV, Jena, Germany

**Introduction:** Two-point compression ultrasound (2-pc) is an important imaging modality for diagnosing deep vein thrombosis (DVT). Traditional ultrasound training comprises classroom lectures and hands-on training (HT), both time- and cost-intense.

**Objectives:** We wanted to assess whether 2-pc can be learned without an instructor.

**Methods:** 77 students were recruited for the study. 47 students were randomized to Groups A and B. 30 students to group C.

Group A had access to an e-learning curriculum prior to the course. At the day of the course they watched a live-demo. After the demo all students received the pocket card and went on to the HT without an instructor.

Group B was the control group without e-learning, but with a class-room lecture at the course day and HT directed by an instructor.

Group C received access to the e-learning, a pocket card and HT without instructor. All groups performed a practical test at the day of the course. Groups A and B repeated the test after 4 weeks.

**Results:** 62 students completed the study protocol.

Group B performed significantly better compared to group A (82.7 %, CI 74.7-90.7 vs. 72.6 %, CI 65.3-80.0; p < 0.05) and group C (65.7 %, CI 59.9-72.6). Looking at specific items of the practical test no difference was found regarding the sequence of the exam or the evaluation of the femoral veins. After four weeks there was no difference in the results of groups A and B showing equal retention of knowledge and skills.

**Conclusions:** Self-directed learning using e-learning, live-demonstration and hands-on training leads to acquisition and retention of skills comparable to traditional teaching methods. This is a time-and cost-effective new learning pathway.

**Fig. 78 (abstract A994).**
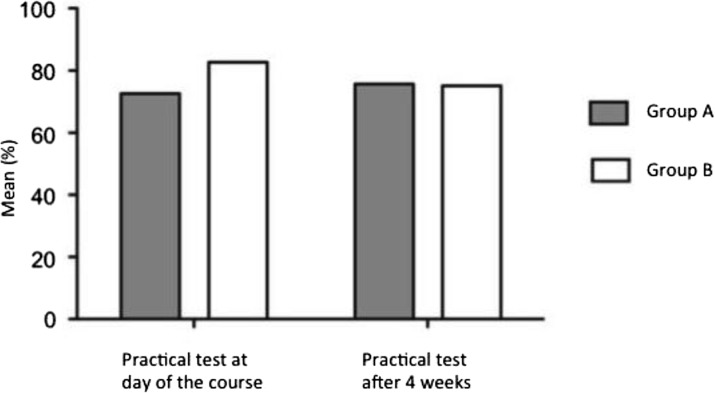
ᅟ

## ANTIBIOTICS AND ANTIBIOTIC RESISTENCE

### A995 Empirical mono- versus combination antibiotic therapy in adult intensive care patients with severe sepsis - a systematic review with meta-analysis and trial sequential analysis

#### F. Sjövall^1,2^, A. Perner^1^, M. Hylander Møller^1^

##### ^1^Rigshospitalet, Copenhagen University Hospital, Intensive Care Unit 4131, Copenhagen, Denmark; ^2^Lund University, Dep. of Clinical Sciences, Lund, Sweden

###### **Correspondence:** F. Sjövall – Rigshospitalet, Copenhagen University Hospital, Intensive Care Unit 4131, Copenhagen, Denmark

**Introduction:** Empirical combination antibiotic therapy for treatment of severe sepsis is a matter of debate. The proposed rationale for using a combination of two or more different antimicrobials is several fold. First, it allows for a broader empirical coverage with a higher likelihood of targeting the causative organism. Second, it may decrease the development of resistance to the antibiotics used. Third, a combination of active drugs potentially cause a synergistic effect increasing the efficacy of bacterial eradication. The Surviving Sepsis Campaign recommends combination therapy in some patient populations and certain type of infections but the quality of the evidence supporting empirical combination antibiotic therapy is weak and does not include high quality randomised clinical trials (RCTs).

**Objectives:** To assess benefits and harms of empirical mono- *vs.* combination antibiotic therapy in adult patients with severe sepsis in the intensive care unit (ICU).

**Methods:** We performed a systematic review according to the Cochrane Collaboration methodology, including meta-analysis, risk of bias assessment and trial sequential analysis (TSA). We included RCTs assessing empirical mono-antibiotic therapy versus a combination of two or more antibiotics in adult ICU patients with severe sepsis. We exclusively assessed patient-important outcomes, including mortality. Two reviewers independently evaluated studies for inclusion, extracted data, and assessed risk of bias. Risk ratios (RRs) with 95 % confidence intervals (CIs) were estimated and the risk of random errors was assessed by TSA.

**Results:** Thirteen RCTs (*n* = 2,633) were included; all were judged as having high risk of bias. There was no difference in mortality (RR 1.11, 95 % CI 0.95 - 1.29; *p* = 0.19) or in any other patient-important outcomes between mono- *vs.* combination therapy. In TSA of mortality, the Z-curve reached the futility area, indicating that a 20 % relative risk difference in mortality may be excluded between the two groups. For the other outcomes, TSA indicated lack of data and high risk of random errors.

**Conclusions:** This systematic review of RCTs with meta-analysis and TSA demonstrated no differences in mortality or other patient-important outcomes between empirical mono- *vs.* combination antibiotic therapy in adult ICU patients with severe sepsis. The quantity and quality of data was low without firm evidence for benefit or harm of combination therapy.

### A996 De-escalation, antimicrobial adequacy and culture positivity in septic patients in a middle income country: observational study

#### R.B. Moraes^1,2^, F.K. Borges^3^, J.A.V. Guillen^3^, W.J.C. Zabaletta^3^, PICS- HCPA: Programa Intrahospitalar de Combate à Sepse do Hospital de Clínicas de Porto Alegre

##### ^1^Hospital de Clínicas de Porto Alegre, Intensive Care Unit, Porto Alegre, Brazil; ^2^Hospital Femina Grupo Hopsitalar Conceição, Intensive Care, Porto Alegre, Brazil; ^3^Hospital de Clínicas de Porto Alegre, Internal Medicine, Porto Alegre, Brazil

###### **Correspondence:** R.B. Moraes – Hospital de Clínicas de Porto Alegre, Intensive Care Unit, Porto Alegre, Brazil

**Introduction:** De-escalation antibiotic in sepsis is associated with reduced costs and bacterial resistance. However, often it is not done.

**Objectives:** We designed this study with the primary objective to evaluate the prevalence of de-escalation in patients with severe sepsis or septic shock in an academic public hospital in south Brazil. Secondarily we evaluated antibiotic adequacy and cultures positivity.

**Methods:** We analyzed prevalence of de-escalation, antibiotic adequacy and culture positivity in severe sepsis and septic shock patients in an Intensive Care Unit.

**Results:** Of the 224 patients included, de-escalation could have been performed in 29 % of cases (66 patients), but was implemented in only 19 % of cases (44 patients). Among patients who received de-escalation, half was for antimicrobial spectrum narrowing. The mortality was not different between patients with or without de-escalation (56.8 % versus 56.1 %, p = 0.999). Empirical antimicrobial therapy was adequate in 89 % of cases. Pathogens were isolate in 30 % of all cultures and 26.3 % of blood cultures.

**Conclusion:** The rate of empiric antibiotic adequacy was high, reflecting active institutional policy of monitoring the epidemiological profile and institutional protocols of antimicrobial use. However, the antimicrobial de-escalation could have been higher than reported. De-escalation did not impact mortality. There are few data in the literature regarding the care of severe sepsis patients in developing countries. This data can contribute to adequate treatment in this scenario.

**References**

1. Silva BN, Andriolo RB, Atallah AN, Salomão R. De-escalation of antimicrobial treatment for adults with sepsis, severe sepsis or septic shock. Cochrane Database Syst Rev. 2013 Mar 28;3:CD007934

2. Garnacho-Montero J1, Gutiérrez-Pizarraya A, Escoresca-Ortega A, Corcia-Palomo Y, Fernández-Delgado E, Herrera-Melero I, Ortiz-Leyba C, Márquez-Vácaro JA. De-escalation of empirical therapy is associated with lower mortality in patients with severe sepsis and septic shock. Intensive Care Med. 2014 Jan;40(1):32–40

**Grant acknowledgment**

FIPE- HCPA

### A997 Impact of amikacin pharmacokinetic/pharmacodynamic parameters on clinical outcome of gram-negative infection in critically ill patients

#### J. Ruiz-Ramos^1^, P. Ramirez^1^, M.R. Marqués-Miñana^2^, E. Villarreal^1^, M. Gordon^1^, M. Sosa^1^, P. Concha^1^, A. Castellanos^1^, R. Menendez^3^

##### ^1^Hospital Universitari i Politècnic la Fe, Intensive Care Unit, Valencia, Spain; ^2^Hospital Universitari i Politècnic la Fe, Pharmacy, Valencia, Spain; ^3^Hospital Universitari i Politècnic la Fe, Pneumology, Valencia, Spain

###### **Correspondence:** P. Ramirez – Hospital Universitari i Politècnic la Fe, Intensive Care Unit, Valencia, Spain

**Introduction:** Despite recent advances, appropriate initial amikacin dose in critically ill patients is still challenging. Relationship between pharmacokinetic/pharmacodynamic (pk/pD) parameter peak concentration (Cmax)/minimum inhibitory concentration (MIC) in critically ill patients is not clear.

**Objectives:** We assessed the impact of amikacin pharmacokinetic and pharmacodynamic parameters on clinical and microbiological outcome in these patients.

**Methods:** Observational prospective study. Adult patients (>18 years) admitted to an intensive care unit (ICU) with a gram negative documented infection and treatment with amikacin were included (Study period: September 2014 - April 2015). Amikacin blood samples were taken 24 to 72 hours after treatment started. Amikacin concentration were determined using Indiko® (Thermo Fisher Scientific), and drug adjustment were based on the recommendations given by the Pharmacokinetics Unit (Pharmacy Service).

Clinical response, defined as sign and symptoms presented at the moment of infection diagnosis (fever, chest radiography alteration, infection biomarkers elevation and hemodynamic instability), was evaluated. Ji-square and U-Mann Whitney test were used to compare results between treatment responders and not-responders.

**Results:** 49 patients were included [(Mean age: 56.0 (SD:2.0) years; Median APACHE-II: 22 (IQT: 22–27)]. 25 patients (51.0 %) presented mechanical ventilated infection, 10(20,4 %) catheter-related infections and 5(10.2 %) sepsis without clear focus. Mean bacterial isolated were *K pneumoniae* (40.8 %), *E Cloacae* (18.3 %) and *P aeruginosa* (17.5 %). Mean initial dose was 1150 mg (SD: 45,9)/day, equivalent to 15.8 (0.6) mg/kg/day. With that dose, 40 patients (83,7 %) reached a Cmax/MIC value higher than 8. Final treatment response was higher for those patients with amikacin Cmax/MIC value >8 (61,0 % vs 12,5 %; p = 0.028). No significant differences were reached in early treatment response (Initial 72 h) (57,5 % vs 37,5 %;p = 0,403) or 30 days mortality (19,5 % vs 37,5 %;p = 0,265). Cmax/MIC values was not associated with toxicity-related treatment discontinuation (17,4 vs 12,5; p = 0,581).

**Conclusions:** Initial Cmax/MIC value is associated with clinical response in those patients treated with amikacin. High initial amikacin dose may be necessary to optimize pk/pD parameters.

### A998 De-escalation in a mixed intensive care unit after four years with selective digestive decontamination significantly decreases ICU mortality

#### C. Sánchez Ramírez^1^, M. Cabrera Santana^1^, L. Caipe Balcázar^1^, S. Hípola Escalada^1^, M.A. Hernández Viera^1^, C.F. Lübbe Vázquez^1^, J.J. Díaz Díaz^1^, F. Artiles Campelo^2^, N. Sangil Monroy^3^, P. Saavedra Santana^4^, S. Ruiz Santana^1^

##### ^1^University Hospital of Gran Canaria Dr. Negrín, Intensive Care Unit, Las Palmas de Gran Canaria, Spain; ^2^University Hospital of Gran Canaria Dr. Negrín, Microbiology Department, Las Palmas de Gran Canaria, Spain; ^3^University Hospital of Gran Canaria Dr. Negrín, Pharmacy Department, Las Palmas de Gran Canaria, Spain; ^4^University of Las Palmas de Gran Canaria, Mathematics and Informatics Deparment, Las Palmas de Gran Canaria, Spain

###### **Correspondence:** C. Sánchez Ramírez – University Hospital of Gran Canaria Dr. Negrín, Intensive Care Unit, Las Palmas de Gran Canaria, Spain

**Objective:** To assess the appropriate use of antibiotics and their de-escalation (DE) to treat nosocomial infections in an ICU after four years with Selective Digestive Decontamination (SDD).

**Method:** In a 30 bed mixed ICU from October 1, 2011 to September 30, 2015 nosocomial infections (pneumonia, urinary tract infections, catheter-related bacteremia (CRB) and secondary nosocomial bacteremia) were prospectively collected. ENVIN-HELICS diagnostic criteria were applied. Etiology, inflammatory response to infection, antibiotic treatment (ATB T) and treatment modifications according to culture results, were analyzed. SDD was applied to all admitted patients requiring endotracheal intubation over 48 hours. For each groups categorical variables were summarized as frequencies and percentages and number in means and standard deviations (SD) or median with interquartile ranges (IQR).Percentages were compared, as appropriate, with the Fisher´s exact test or X^2^ test and medians with the Wilcoxon test for independent samples. For those variables that were associated with DE in the univariate analysis were entered into a logistic multidimensional analysis. The model obtained was expressed by p-values and odd-ratios, which were estimated by confidence intervals at 95 %. A hypothesis test was considered statistically significant when p-value was less than .05.

**Fig. 79 (abstract A998).**
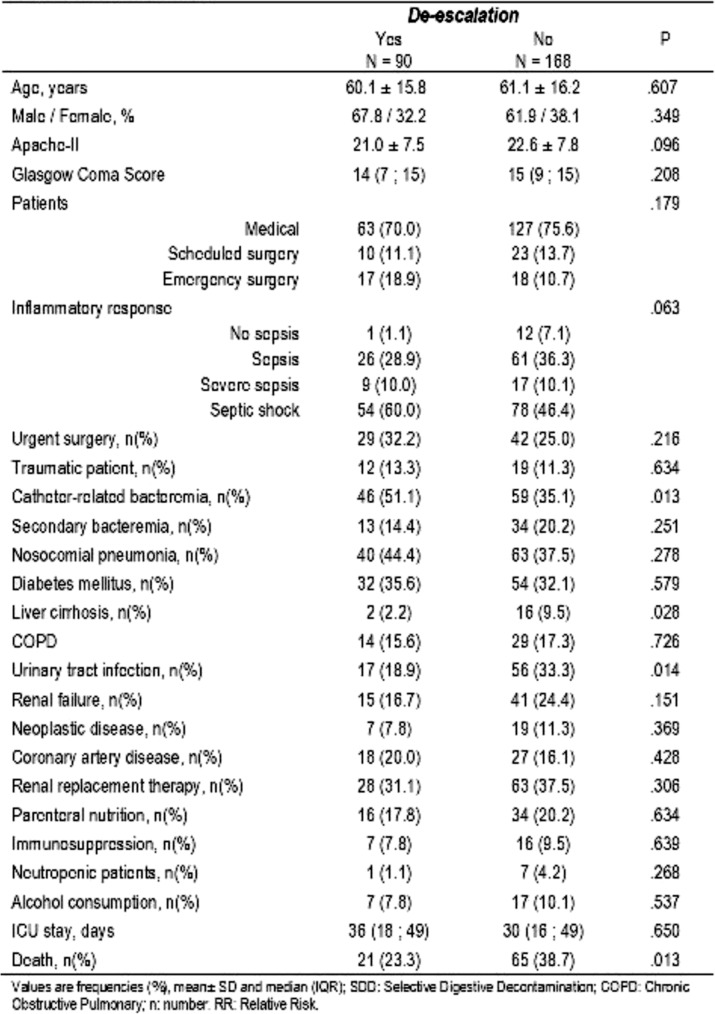
Univariate analysis

**Results:** Ninety patients (34,8 %) had ATB DE and 168 did not. There were no significant differences in demographics or type of admission in both groups (Fig. 79).

**Fig. 80 (abstract A998).**
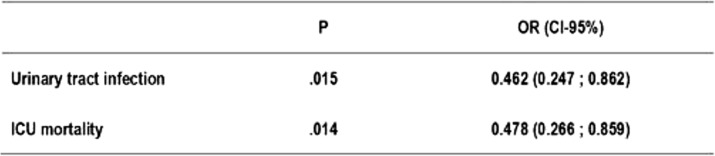
Multivariate analysis

Mortality was lower in patients receiving DE antibiotic (ATB) (23,3 %, p: 0.013). In the multivariate analysis, ICU mortality and urinary tract infection were the only variables found significant (Fig. 80)

DE was performed in 53 out of 105 (50,4 %) with CRB and in 45 out of 103 (43,6 %) who had nosocomial pneumonia. The ATB T was inadequate in 58 out of 364 infections (15,9 %). Targeted therapy was performed in 136 out of 258 patients (52,7 %) and in 162 out of 364 infections, at least once occasion (44,5 %). Finally, 199 ATB were targeted prescribed.

**Fig. 81 (abstract A998).**
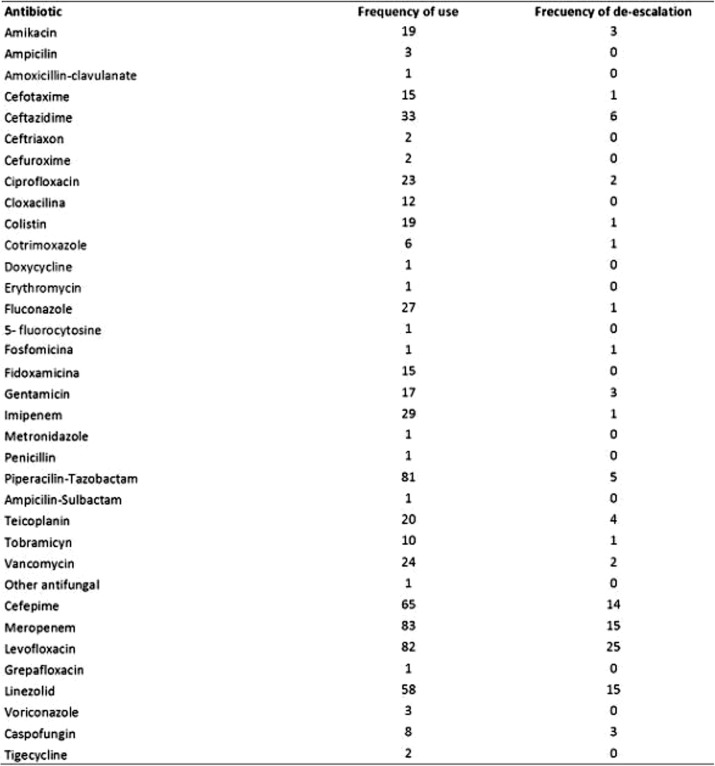
Antibiotics

In all studied patients with DE, this was performed in 72 patients once, in 13 patients twice and in 3 patients three times. The number of antibiotics used was 668 and ATB DE was performed in 104 occasions. Frequency of ATB used and of theirs DE is shown in Fig. 81

Of note, meropenem was DE in 14,4 %.

**Conclusions:** Patients who received ATB DE compared to those that did not had a significant lower ICU mortality. The factors independently associated to DE were ICU mortality and urinary tract infection. Inadequate ATB T in our ICU occurred in 15.9 % of nosocomial infections. ATB DE was performed in 90 patients. Targeted therapy was applied to 44,5 % of infections. The most commonly used antibiotics were meropenem (12,3 %), levofloxacin (12.2 %) and piperacillin-tazobactam (12,05 %). Meropenem, was DE in 14,4 %.

### A999 De-escalation of empirical therapy in septic patients and its positive impact on mortality: a collaborative international cohort

#### A. Gutiérrez-Pizarraya^1^, J. Garnacho-Montero^2^, C. Martin^3^, K. Baumstarck^4^, M. Leone^3^, I. Martín-Loeches^5^

##### ^1^Hospital Virgen del Rocio, Seville, Spain; ^2^Hospital Virgen Macarena, Seville, Spain; ^3^Aix Marseille Université, Assistance Publique-Hôpitaux de Marseille, Hôpital Nord, Service d'Anesthésie et de Réanimation, Marseille, France; ^4^EA 3279 Research Unit, Aix Marseille University & Assistance Publique-Hôpitaux de Marseille, Marseille, France; ^5^Department of Clinical Medicine, Trinity College, Wellcome Trust-HRB Clinical Research Facility, St James Hospital, Dublin, Ireland

###### **Correspondence:** A. Gutiérrez-Pizarraya – Hospital Virgen del Rocio, Seville, Spain

**Introduction:** Antimicrobial prescription represents a major challenge for clinicians in the daily practice especially in certain difficult clinical scenarios. Thus, in critically ill septic patients, prompt and adequate antimicrobial therapy reduces morbidity and mortality

**Objectives:** We set out to assess the impact on in-hospital of antibiotic de-escalation in patients admitted to the ICU with severe sepsis or septic shock.

**Methods:** Collaborative study enrolling patients admitted to the ICU with severe sepsis or septic shock from two different cohorts. The first one, a Spanish prospective and observational cohort and the second one, a multicenter non-blinded, randomized and non inferiority trial conducted in France. Severity was estimated by the use of the predicted mortality rate at ICU admissionfor every included patientby implementing the likelihood of death logit formule defined according to the APACHE II and SAPS II scores criteria and taking this cuantititative variable into account as a confounder factor in the regression model. De-escalation was defined as discontinuation of an antimicrobial agent or change of antibiotic to one with a narrower spectrum once culture results were available. To control for confounding variables we performed a multivariatebinomial logistic regression analysis adjusted by Wald test.

**Results:** Nine hundred and one patients with severe sepsis or septic shock at ICU admission were treated empirically with broad-spectrum antibiotics. Eight hundred and seventeen patients were evaluated (84 died before cultures were available). De-escalation was applied in 274 patients (33.5 %). We found no differences in hospital long of stay between de-escalation group comparedto those who did not received it. We also found a significant lower hospital mortality in de-escalation group in front of the others (25.9 *vs.* 43.1 %; *p* < 0.001). By multivariate analysis (adjusted by severity scores-APACHE and SAPS), factors independently associated with in-hospital mortality were age (Odds-Ratio [OR] 1.02; 95 % confidence interval [CI] 1.01-1.03), and SOFA score at ICU admission (OR 1.17; 95 % CI 1.11-1.23), whereas de-escalation therapy was a protective factor (OR 0.50; 95 % CI 0.35 - 0.71) as well as urinary focus (OR 0.23; 95 % CI 0.12-0.43). Analysis of the 646 patients with etiological diagnosis revealed that the factors associated with mortality were age and SOFA and conversely de-escalation therapy was a protective factor (OR 0.45; 95 % CI 0.30-0.66)

**Conclusions:** De-escalation therapy for septic critically ill patients is a safe strategy associated with a lower mortality. Efforts to increase the frequency of this strategy are indeed justified.

### A1000 Patient-centered estimation of the impact of vancomycin/aminoglycosides on kidney function in septic patients

#### R. Pirracchio^1,2^, M. Legrand^3^, J.L. Mainardi^4^, J. Mantz^4^, B. Cholley^4^, A. Hubbard^5^

##### ^1^Hopital Europeen Georges Pompidou, Anesthesiology and Intensive Care Medicine, Paris, France; ^2^University of California Berkeley, Biostatistics, Berkeley, United States; ^3^Hopital Saint Louis, Paris, France; ^4^Hopital Europeen Georges Pompidou, Paris, France; ^5^University of California Berkeley, Berkeley, United States

###### **Correspondence:** R. Pirracchio – Hopital Europeen Georges Pompidou, Anesthesiology and Intensive Care Medicine, Paris, France

**Introduction:** At the population level, both vancomycin and aminoglycosides are known to be nephrotoxic. The risk of nephrotoxicity might even be higher when combining the 2 agents together (1). Nonetheless, in septic patients, the benefit in terms of sepsis control may outweight the risk of nephrotoxicity. Thus, being able to appraise the risk/benefit ratio at the patient level would be of great interest to better tailor individual treatment.

**Objectives:** Capitalizing on recent statistical innovations in personnalized medicine, our goal was to develop a patient-centered estimation of the impact of the association vancomycin/aminoglycosides on the kidney function.

**Methods:** Our data come from a cohort study performed between 2000 and 2010 in the departments of anesthesia, critical care and cardiovascular surgery at a French teaching hospital. This study included all consecutive patients operated for an acute endocarditis (2). The primary endpoint was postoperative evolution of the kidney function as evaluated by the AKIN score (Stade 1: elevation in serum creatinine (SrCr) ⩾ 26.2 μmol/L or ⩾ 1,5xbaseline; Stade 2: elevation in SrCr ⩾ 2xbaseline; Stade 3: elevation in SrCr ⩾ 3xbaseline or creatinine ⩾ 354 μmol/L with increase >44 μmol/L or need for RRT). The impact of vancomycin/aminoglycosides on kidney function was estimated using targeted maximum likelihood estimation on a risk difference (RD) scale. The association between patient characteristics and the individual effect of the drugs on the kidney function was estimated using conditional recursive partitioning (cTREE).

**Fig. 82 (abstract A1000).**
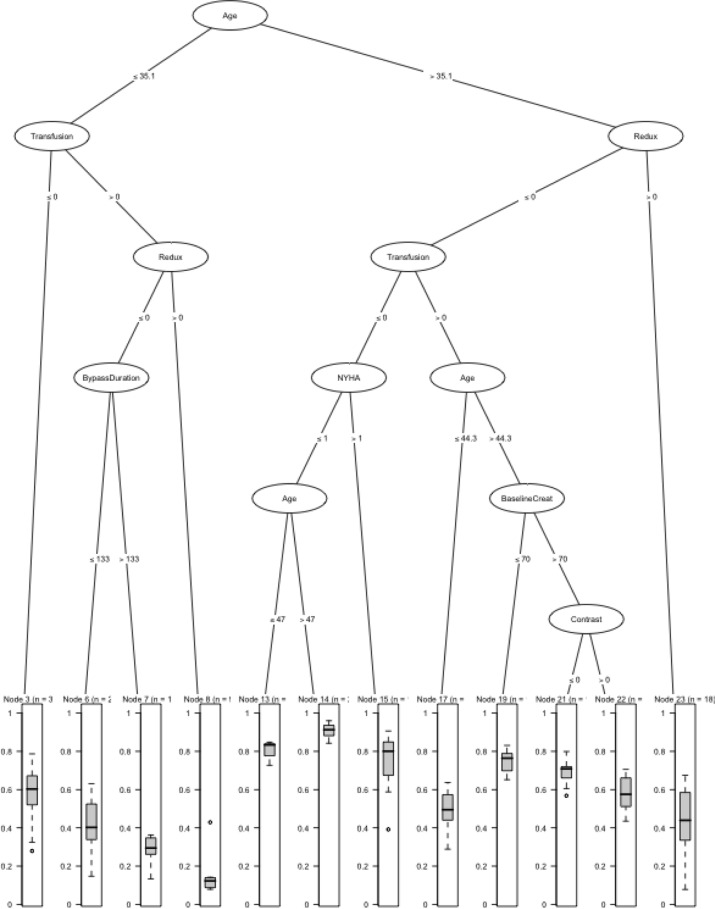
Prediction of individual impact on kidney function

**Results:** 202 patients were included in the study. Their baseline characteristics are described in Table 53. At a population level, we confirmed the strong association between vancomycin + aminoglycosides and the risk of kidney dysfunction (RD = 0.54, 95%CI: 0.47-0.61, p < 0.001). However, at the patient level, this effect was very variable and could be predicted based on patients characteristics (r^2^ = 0.74) (Figure 82).

**Conclusions:** The individual impact of vancomycin + aminoglycosides on kidney function may be very different than the overall effect at the population level. Innovant statistical approaches may be used to identify patients in whom this drug combination is safe, and others in whom it may seriously threaten kidney function.

**References**

(1) Fauconneau, Ren. Fail. 1997Jan;19(1):15–22;

(2) Legrand, Crit. Care 2013 Oct 4;17(5):R220.

**Table 53 (abstract A1000).** Baseline characteristics

Age : median (IQR)	42 (28–59)
Preop serum creatinine (μmol/l)	100 (76–132)
Urgent surgery	27 (13 %)
Aortic valve	104 (51 %)
Mitral	103 (51 %)
Tricuspid	20 (10 %)
Bypass duration (min)	118 (85–163)
aminoglycosides	119(59 %)
vancomycin	36(18 %)
vancomycin + aminoglycosides	94(46 %)

### A1001 Early goal-directed antibiotic therapy in catheter-related bloodstream infections

#### P. Ruiz Frontera^1^, L.M. Claraco Vega^1^, P. Ruiz de Gopegui Miguelena^1^, M.C. Villuendas Usón^2^, A. Rezusta López^2^, E. Aurensanz Clemente^3^, P. Gutiérrez Ibañes^1^, A.L. Ruiz Aguilar^1^

##### ^1^Hospital Universitario Miguel Servet, ICU, Zaragoza, Spain; ^2^Hospital Universitario Miguel Servet, Microbiology, Zaragoza, Spain; ^3^Hospital de Barbastro, Barbastro, Spain

###### **Correspondence:** P. Ruiz Frontera – Hospital Universitario Miguel Servet, ICU, Zaragoza, Spain

**Introduction:** Catheter-related bloodstream infection (CRBSI) is one of the most frequent nosocomial infections in critically ill patients, resulting in a significant increase of morbidity and mortality. That is why it is essential to detect CRBSI precociously so that optimal treatment can be initiated as soon as possible.

**Objective:** Our objective is to evaluate the effectiveness of rapid non-invasive tests that may allow clinicians to detect colonisation of the central venous catheter (CVC) as a source of bloodstream infection in critically ill patients, permitting catheter withdrawal and the initiation of early goal-directed antibiotic therapy.

**Methods:** Over the course of eight months, we selected for evaluation those critically ill patients admitted to our ICU who developed fever (>38 °C) without source, to whom the clinician in charge decided to withdraw the CVC. Before extraction, we obtained a skin smear at the insertion site, in addition to a catheter-hubs smear.

We sent both smears, along with the catheter tip and blood cultures, to the microbiology laboratory. The results of the skin and catheter-hub cultures were obtained within 24 hours. We compared the result of the combination of the smear cultures with the gold standard for diagnosis of CVC colonisation: the culture of the catheter tip using the Maki's technique.

The diagnosis of bloodstream infection was established from the blood culture results, which took at least 72 hours. CRBSI was considered if the same microorganism was detected in both the catheter tip and in the blood cultures.

**Results:** Sixty-two CVCs were removed due to suspected CRBSI. Colonisation was confirmed using the Maki's technique in 20 CVCs (32 %). The combination of skin and catheter-hub cultures showed 24 positive results (four false positives) and 38 negative results (no false negatives). We detected 15 cases of CRBSI (24 %), corresponding to a 62 % of cases with positive results of our test.

These results indicate that our test has a negative predictive value of 100 %. To evaluate the degree of association between our test and the gold standard, we calculated the contingency coefficient, which resulted in 0,65 out of a maximum 0,7(p < 0,001). This shows the strong validity of the combination of skin and catheter-hub cultures as a diagnostic method for CVC colonisation.

**Conclusions:** The combination of skin and catheter-hub cultures is a rapid and very effective method for detecting the colonisation of CVC as a possible source of bloodstream infection in critically ill patients. While a negative result, which can be obtained within 24 h, would prevent the need for CVC withdrawal, a positive result would not only enable the removal of the likely source of infection, but it would also allow for, if the intensivist deemed it necessary, the initiation of early goal-directed antibiotic therapy. This would mean, in more than 60 % of cases, starting optimal treatment at least 48 hours prior to the diagnosis of CRBSI from blood cultures.

### A1002 Carbapenem use in ICU

#### M. Palomar^1^, P. Olaechea^2^, S. Uriona^3^, M. Vallverdu^1^, M. Catalan^4^, X. Nuvials^1^, C. Aragon^5^, F. Alvarez Lerma^6^, ENVIN-HELICS Study Group

##### ^1^HU Arnau de Vilanova, Lleida, Spain; ^2^H Galdakao, Bilbao, Spain; ^3^HU Vall d´Hebron, Barcelona, Spain; ^4^HU Doce de Octubre, Madrid, Spain; ^5^HU Malaga, Malaga, Spain; ^6^Parc de Salut Mar. IMIM (Mar Medical Research Institut), Barcelona, Spain

###### **Correspondence:** M. Palomar – HU Arnau de Vilanova, Lleida, Spain

**Introduction:** The overuse of carbapenems (CBP) facilitates the emergence and spread of Carbapenemases . In Spain, their presence is a real threat since 2012.

**Objectives:** To asses the use of CBP in recent years in the Spanish ICUs.

**Methods:** Analysis of antibiotic treatments (ATB) included in the Spanish register ENVIN-HELICS from 2008 to 2015. We differentiated ATB in CBP and other ATB (O-ATB). Indications (treatment for community, nosocomial or ICU acquired infections (AI) and prophylaxis) and form of use(empiric, directed) were compared. Statistical test: Chi square test.

**Results:** : In 8 years of study, 150,877 patients were recorded: They received a total of 202,867 ATB and 1,251.333 days of treatment (DOT). In 15.50 % of patients, 23,393 CBP (11.53 % of total ATB) were indicated during 178,381 DOT (14.26 % of total ATB DOT). The number of CBP indications increased from 2008 until 2012, (when a national alarm for carbapenemases presence with subsequent decrease was observed although this decrease was only significant on the percentage of CBP- DOT and not on the percentage of indications or the percentage of patients receiving CBP

Differences in the form of use were also observed ; empirical treatment (%): 85.82 CBP vs. 76.63 O-ATB (p < 0.001). Indication (%): Community -AI 30,35 CBP vs. 28,66 O-ATB; nosocomial-AI 35.75 vs. 19.03; ICU-AI 28.3 vs. 21.85 and Prophylaxis/unknown: 30.45 vs. 5.58 (p < 0.001) with variations over the years (less CBP for I-ICU and more for community and nosocomial- AI ).

The microbiological documentation was higher for CBP: 47.37 % vs. 41.66 % (p < 0.001) and in these cases, the treatment was adequate in greater proportion: 28.41 % CBP vs. 38.2 % O-ATB (p < 0.001 ). However, the change of empirical or targeted treatments was similar 22.1 % vs. 22.01 %, although when it was changed, reduction of the spectrum was higher 53.9 % CBP vs. 30.3 % O-ATB (p < 0.001).

**Conclusions:** Increased use of CBP observed until 2012 has begun to reverse. Usage patterns have changed, with fewer indications for ICU-AI. The use is mainly empirical and although few treatments are changed, the main reason is the reduction of the spectrum. , there is still much room for improvement.

**Fig. 83 (abstract A1002).**
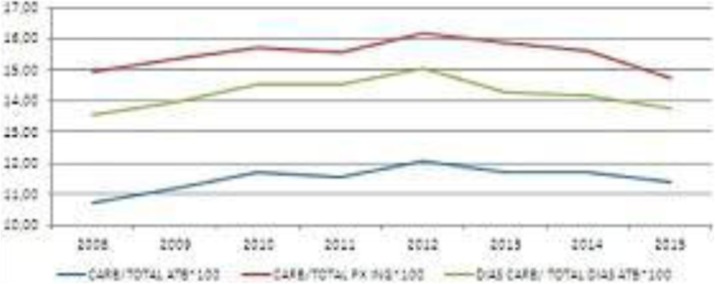
ᅟ

### A1003 The clinical impact of matrix-assisted laser desorption/ionization time-of-flight without antimicrobial stewardship in patients with gram negative bacteremia

#### Y.D. Jeon^1^, W.Y. Jeong^2^, M.H. Kim^2^, I.Y. Jeong^2,3^, M.Y. Ahn^2^, J.Y. Ahn^2^, S.H. Han^2,3^, J.Y. Choi^2,3^, Y.G. Song^2,3^, J.M. Kim^2,3^, N.S. Ku^2,3^

##### ^1^Yonsei Wonju University College of Medicine, Department of Internal Medicine, Wonju, Republic of Korea; ^2^Yonsei University College of Medicine, Department of Internal Medicine, Seoul, Republic of Korea; ^3^Yonsei University College of Medicine, AIDS Research Institute, Seoul, Republic of Korea

###### **Correspondence:** Y.D. Jeon – Yonsei Wonju University College of Medicine, Department of Internal Medicine, Wonju, Republic of Korea

**Introduction:** Several studies evaluated the impact of matrix-assisted laser desorption/ionization time-of-flight (MALDI-TOF) combined with antimicrobial stewardship in patients with bacteremia and showed improved clinical outcomes.^1–3^ But, in many hospitals, antimicrobial stewardship has not been being applied due to restricted medical resources.^4^

**Objectives:** Thus, we investigated the clinical impact of MALDI-TOF without antimicrobial stewardship in patients with gram negative bacteremia.

**Methods:** Retrospectively, medical records from patients (>18 years old) with gram negative bacteremia in two periods, between October to December in 2012 and 2013, were reviewed. Conventional method was used for the detection of bacterial pathogen in 2012 and MALDI-TOF was used in 2013. The clinical outcomes were compared between conventional group and MALDI-TOF group.

**Results:** In total, 324 patients with gram negative bacteremia were enrolled in this study: 181 patients in the conventional group and 143 patients in MALDI-TOF group. MALDI-TOF reduced time to effective antibiotic therapy (21.43 vs 14.1 hours, *P* = 0.035). The length of hospital stay (24.7 vs 21.4 days, *P* = 0.556), recurrent bacteremia (7.5 vs 2.7 %, *p* = 0.051) and 28-day mortality (13.4 vs 8.7 %, *P* = 0.176) were lower in MALDI-TOF group, but not statistically significant.

**Conclusions:** The use of MALDI-TOF might reduce time to effective antibiotic therapy, regardless of antimicrobial stewardship.

**References**

1. Clerc O, Prod´hom G, Vogne C, Bizzini A, Calandra T, Greub G. Impact of matrix-assisted laser desorption ionization time-of-flight mass spectrometry on the clinical management of patients with Gram-negative bacteremia: a prospective observational study. *Clinical infectious diseases : an official publication of the Infectious Diseases Society of America.* 2013;56(8):1101–1107.

2. Huang AM, Newton D, Kunapuli A, et al. Impact of rapid organism identification via matrix-assisted laser desorption/ionization time-of-flight combined with antimicrobial stewardship team intervention in adult patients with bacteremia and candidemia. *Clinical infectious diseases : an official publication of the Infectious Diseases Society of America.* 2013;57(9):1237–1245.

3. Wenzler E, Goff DA, Mangino JE, Reed EE, Wehr A, Bauer KA. Impact of rapid identification of Acinetobacter Baumannii via matrix-assisted laser desorption ionization time-of-flight mass spectrometry combined with antimicrobial stewardship in patients with pneumonia and/or bacteremia. *Diagnostic microbiology and infectious disease.* 2016;84(1):63–68.

4. Howard P, Pulcini C, Levy Hara G, et al. An international cross-sectional survey of antimicrobial stewardship programmes in hospitals. *The Journal of antimicrobial chemotherapy.* 2015;70(4):1245–1255.

**Grant acknowledgment**

This work was supported by a faculty research grant of Yonsei University College of Medicine for 2014(6-2014-0012).

### A1004 Nebulized amikacin/fosfomycin and intravenous meropenem for the treatment of amikacin-resistant meropenem-susceptible *P. aeruginosa* pneumonia: an experimental study

#### G. Li Bassi^1^, E. Aguilera Xiol^1^, T. Senussi^1^, F.A. Idone^1^, A. Motos^1^, C. Chiurazzi^2^, C. Travierso^3^, L. Fernández-Barat^1^, R. Amaro^1^, Y. Hua^1^, O.T. Ranzani^1^, Q. Bobi^1^, M. Rigol^1^, A. Torres^1^

##### ^1^Hospital Clinic, Pulmonary and Critical Care Medicine, Barcelona, Spain; ^2^University of Milan, Fisiopatologia Medico-Chirurgica e dei Trapianti, Milano, Italy; ^3^University of Milan, Fisiopatologia medico-chirurgica e dei trapianti, Milano, Italy

###### **Correspondence:** G. Li Bassi – Hospital Clinic, Pulmonary and Critical Care Medicine, Barcelona, Spain

**Introduction:** Often, in critically ill patients with severe pneumonia, intravenous antibiotics have marginal therapeutic efficacy, due to limited lung penetration. Nebulized antibiotics could be a promising alternative to ameliorate antibacterial efficacy.

**Objectives:** We tested, in an animal model of severe pneumonia, the effects of nebulized and systemic antibiotics on pulmonary function and bacterial burden.

**Methods:** Twenty-four pigs (31.8 ± 1.9 Kg) were anesthetized and on mechanical ventilation (MV) for 78 hours. Multi-lobar pneumonia was induced by *P. aeruginosa* (1), resistant to amikacin (A), fosfomycin (F) and susceptible to meropenem (Mero). Following clinical diagnosis of pneumonia, animals were randomized to receive the following treatments: nebulized saline (control); nebulized AF; nebulized A; nebulized F; AF with intravenous (IV) Mero; IV Mero alone. Nebulization was performed through a vibrating mesh nebulizer (PARI GmbH, Germany). Every 24 h, white blood cells, creatinine, arterial blood gases, respiratory system elastance were assessed. Tracheal secretions were aspirated to quantify *P. aeruginosa* concentration upon clinical diagnosis of pneumonia, and at 48 and 72 h of MV. Upon autopsy, pulmonary lobes were biopsied for quantitative microbiology cultures.

**Results:** Table 54 reports differences among study groups of *P. aeruginosa* tracheal secretions concentrations (p < 0.001) and pulmonary bacterial burden (p < 0.001).

On average, white blood cell were 14.7 ± 8.7 10^9^/L; creatinine was1.05 ± 0.3 mg/dL; pulmonary system elastance 36.8 ± 11.5 cmH_2_O/L, PEEP was 7.1 ± 2.5 cmH_2_O and PaO_2_/FIO_2_ 380 ± 84 mmHg_,_ without differences among study groups for all variables.

**Conclusions:** In a porcine model of AF-resistant *P.aeruginosa* pneumonia, the use of nebulized AF, associated with systemic meropenem, achieves higher bactericidal efficacy than nebulized or systemic antibiotics alone. This does not result in statistically significant improvement in pulmonary function after the first 2 days of treatment.

**References**

1. Luna C et al. Chest 132, 2007: 523–31

**Grant acknowledgment**

Support was provided by Cardeas Pharma Ltd.

**Table 54 (abstract A1004).** Microbiology studies

	Control (4 Animals)	Nebulized Amikacin/Fosfomycin (4 Animals	Nebulized Amikacin (3 Animals)	Nebulized Fosftomycin (4 Animals)	Nebulized Amikacin/Fosfomycin and IV Meropenem (6 Animals)	IV Meropenem (3 Animals)	P-Value
Tracheal Secretions P.aeruginosa Concentration (log cfg/mL)	5.9 ± 1.9	2.9 ± 3.0*	2.4 ± 2.7*	4.7 ± 0.9	2.5 ± 2.8*	5.1 ± 0.9	<0.001
Pulmonary P.aeruginosa Concentration (log cfg/gr)	6.2 ± 1.6	6.3 ± 1.3	6.3 ± 1.0	5.0 ± 2.4	3.8 ± 2.1**	5.3 ± 0.7	<0.001

Data are reported as mean ± SD

*IV* intravenous

*p < 0.05 vs. Fosfomycin, control and IV meropenem group

**p < 0.05 vs. Amikacin/Fosfomycin, Amikacin and control group

### A1005 Is useful the infusion of 8/1 grams of piperacillin-tazobactam in intensive care unit?

#### I. Fuentes Fernández^1^, E. Andreu Soler^1^, A. Pareja Rodríguez de Vera^2^, E. Escudero Pastor^3^, V. Hernandis^3^, J. Ros Martínez^1^, R. Jara Rubio^1^

##### ^1^Hospital Virgen de la Arrixaca, ICU, El Palmar, Spain; ^2^Hospital Virgen de la Arrixaca, Pharmacy, El Palmar, Spain; ^3^University of Murcia, Faculty of Veterinary, Murcia, Spain

###### **Correspondence:** I. Fuentes Fernández – Hospital Virgen de la Arrixaca, ICU, El Palmar, Spain

**Introduction:** Piperacillin/tazobactam (PTZ) is a β-lactam-β-lactamase inhibitor combination with a broad spectrum of antibacterial activity. β-lactams are time-dependent antibiotics and their effectiveness is in association with the duration of free drug concentrations over the minimum inhibitory concentration (t > MIC) of organisms. Prolonged infusion has a pharmacokinetic (PK) advantage compared to intermittent bolus dosing, a continuous infusion lower dose of 8 g PTZ may be as effective as a higher dose of intermittent bolus PTZ.

**Objectives:** In this study we intend to evaluate the continuous infusion of 8/1 grams of PTZ along with a loading dose of 4/0,5 grams in patients admitted in a tertiary ICU.

**Methods:** Between October 2015 and December 2015 eight patients had piperacillin plasma concentration monitored during treatment with continuous PTZ infusion in a monocentric prospective observational study. Patients received a loading dose of 4/0,5 grams of PTZ followed by infusion of 8/1 grams, reconstituted in 90 ml Sodium Chloride 0,9 % and transferred to Braun Space Infusion System®. The pump had a flow rate of ± 4 ml/h.

Blood was always extracted from the contralateral arm to the infusion, over 60 hours at predetermined times. Serum piperacillin/tazobactam concentrations were determined using an HPLC method (2). After extraction, samples (99 μL) were injected into a XBRIDGE C18 column (Waters, Spain) and were scanned by an UV detector at 220 nm with gradient elution. Mobile phase was composed by acetonitrile and a solution of tetrabutylammonium bisulfate (10 g/L). Penicillin G was used as internal standard (Sigma Aldrich, Spain).

**Results:** 8 patients have been examined (4 men and 4 women). The average age was 53 ± 22, the weight was 81 ± 22 kg, the creatinine clearance 122 ± 57 mL/min and the Apache II score 21,7 ± 7,3.

The mean concentrations of PTZ in serum are represented in the next figures.

**Conclusions:** In this ICU patient group, our results suggest that continuous infusion of PTZ at 8/1 g per day is sufficient to obtain therapeutic plasma-concentrations in critical care patients with infections caused by PTZ sensitive bacteria with a MIC lower than 8 mg/dl. However, in our group there were three patients with levels of 9 mg/dl, which are not sufficient for bacteria with MIC lower than 16 mg/dl; these three patients are neurocritical. In conclusion, further studies in this are needed, especially studies regarding the association between piperacillin therapeutic drug monitoring and clinical outcome.

**References**

1. Dulhunty JM et al. Continuous infusion of beta-lactam antibiotics in severe sepsis: a multicenter double-blind, randomized controlled trial. Clin Infect Dis. 2013 Jan;56(2):236–44.

2. Di Giovamberardino et al. HPLC determination of plasma free and total tazobactam and piperacillin. J Chromatograp B 2009, 877: 86–88.

**Grant acknowledgment**

We appreciate the cooperation of ICU nursing service and staff of the University of Murcia

**Table 55 (abstract A1005).** Patient, clinical characteristics

Patient	Sex	Age	CL CR	Weight	BMI	APACHE II	SAPS II	Diagnostic
A	M	74	45	72	26,45	17	40	Cardiac surgery
B	M	58	148	105	34,29	10	32	Hemorraghic stroke
C	M	21	207	85	24,84	22	50	Trauma brain injury
D	F	50	120	58	25,11	31	57	Cerebral aneurysm
E	M	21	188	65	21,22	21	42	Trauma brain injury
F	F	50	140	66	24,54	20	31	Acute respiratory failure
G	F	75	40	80	27,68	20	52	Cardiogenic shock
H	F	78	91	120	44,08	33	62	Acute respiratory failure

**Fig. 84 (abstract A1005).**
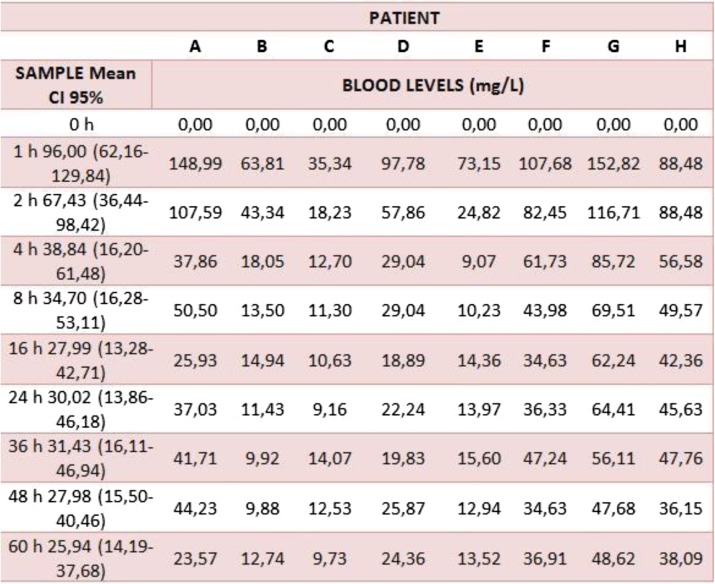
PTZ BLOOD LEVELS

**Fig. 85 (abstract A1005).**
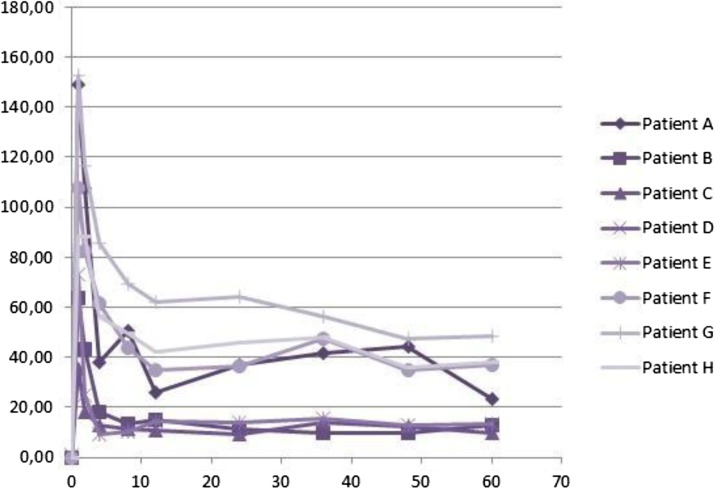
PTZ BLOOD LEVELS

### A1006 Risk factors for infradosification of piperacilin administered in continuous infusion in critically ill patients

#### M. Miralbés Torner^1^, S. Carvalho Brugger^1^, A. Aragones Eroles^2^, S. Iglesias Moles^1^, J. Trujillano Cabello^1^, J.A. Schoenenberger^1^, X. Nuvials Casals^1^, M. Vallverdu Vidal^1^, B. Balsera Garrido^1^, M. Palomar Martinez^1^

##### ^1^Hospital Universitari Arnau de Vilanova, Lleida, Spain; ^2^Institut de Recerca Biomédica de Lleida, Lleida, Spain

###### **Correspondence:** M. Miralbés Torner – Hospital Universitari Arnau de Vilanova, Lleida, Spain

**Introduction:** Piperacilin-tazobactam (TZP) is an extended-spectrum beta-lactam antibiotic (ATB) broadly used in intensive care units. Recently, it has been suggested that standard doses of piperacillin might not be sufficient for critically ill patients.

**Objectives:** Identify risk factors for infra-dosification of piperacillin administered in continuous infusion in critically ill patients.

**Methods:** Open, prospective, single-center study. All consecutive patients in whom treatment with TZP was indicated from October 2014 to March 2016 were included. A 4 g TZP loading dose was given followed by a 16-24 g TZP continuous infusion over 24 hours. Serum concentrations were determined by high-performance liquid chromatography (HLPC) 1 hour, 24 hours and 3–5 days after the start of the infusion, determining maximum (Cmax) and free steady state concentrations (ƒCss). The objective was maintaining ƒCss 4–6 times above the MIC corresponding to the clinical breakpoint for *Pseudomonas aeruginosa* from our hospital database: 8 μg/ml for MER and 16/4 μg/ml for TZP. Here we analyzed Css of TZP during the first 24–48 hours. When the target was not achieved, the dose was adjusted. Univariant, multivariant, logistic regression and ROC analysis were performed. A p value < 0.05 was considered statistically significance.

**Results:** We enrolled 113 patients [73.5 % male (83) and 26.5 % female (30)]. 36 patients (24.1 %) were admitted to ICU after ≥ 7 days of hospitalization. Mean APACHE-II score was 18 ± 8. 73 patients (64.6 %) had septic shock, 27 (23.9 %) severe sepsis, 8 (7.1 %) sepsis and 5 patients (4.4 %) had not systemic inflammatory response. In 20 patients (17.7 %) bacteremia was present. Empiric therapy was administered in 105 cases (92.9 %).

In 30 patients, levels were below target (27.5 %) (Table 56). We did not find statistically significance differences when we compared de dose administrated (p = 0.25). Those patients showing lower levels were younger (p < 0.001) with lower APACHE-II (p < 0.001), did not need mechanical ventilation (p = 0.020) and were hemodinamically stable (p < 0.001). In multivariariate analysis we identified as risk factors for infradosification: low serum creatinine (p < 0.001; OR 1.04), low APACHE-II (p 0.014; OR 1.1) and hemodynamic stability (p 0.041; OR 3.3). The area under the ROC curve was 0.9

**Conclusions:** Even increasing significantly ATB doses, there are 27.5 % patients that remain undertreated. APACHE-II, serum creatinine and hemodynamic stability might be useful to determine optimal ATB doses. We remark the importance of therapeutic drug monitoring and the need to keep studying to identify risk factors for infradosification in order to optimize antibiotic treatment.

Css Piperacilin

**Table 56 (abstract A1006).** Css Piperacilin

	Css < 64	Css 64-96	Css > 96
16 g/day	9 (8.3 %)	7 (6.4 %)	18 (16.5 %)
24 g/day	21 (19.3 %)	18 (16.5 %)	36 (33 %)
TOTAL	30 (27.5 %)	25 (23 %)	54 (49.5 %)

### A1007 Early treatment with polymyxin B improve outcome of patients with gram negative sepsis in hospital wards

#### L. Mirabella, A. Cotoia, L. Tullo, A. Stella, F. Di Bello, A. De Gregorio, M. Dambrosio, G. Cinnella

##### University of Foggia, Anaesthesia and Intensive Care, Foggia, Italy

###### **Correspondence:** L. Mirabella – University of Foggia, Anaesthesia and Intensive Care, Foggia, Italy

**Introduction:** Intra-abdominal sepsis is associated with significantly increased mortality and morbidity both in ICU and Surgical Department. For Gram negative intra-abdominal sepsis the role of endotoxin is a key component and it is known that Direct hemoperfusion (DHP) with an adsorbent column using polymyxin B-immobilized fiber (PMX-F) improve the state of shock.

**Objectives:** The aim of this study is to assess outcome and mortality after the approval of shared protocol with general hospital wards on early treatment with PMX-DHP.

**Methods:** Adult patients with diagnosis of severe sepsis, according to sepsis surviving campaign, Endotoxin Activity Assey (EAA > 0.6), Procalcitonin (PCT > 0.5 ng/ml) and Gram Negative infectious admitted to ICU, Internal Medicine, Surgical Emergency, Cardiology Intensive Care Unit (CICU), and Nephrology Department of University of Foggia from Genuary 2014 to March 2015 were enrolled to two treatment with PMX-DHP.

**Results:** A total of 59 patients were enrolled in the study period, 46 in ICU, 2 in CICU, 2 in Internal Medicine, 7 in Surgical Emergency, 2 in Nephrology Department. Total mortality at 28 days was 79,6 %, ICU mortality was 87,5 % vs 45,5 % in the other hospital wards participating to the study (p < 0.002). During the treatment, 45 patients presented acute kidney injury with necessity of renal support treatment. The surviving patients shows hemodynamic improvement already at 72 hours post treatment with progressive reduction of inotropic support.

**Conclusions:** This study demonstrated the importance of an early treatment with PMX-DHP of Gram negative severe sepsis and the application of shared protocol in hospital wards in reducing 28 days mortality.

**References**

1. Cruz DN , Perazella, M A Bellomo et al.Effectiveness of polymyxin B-immobilized fiber column in sepsis: a systematic review Crit Care. 2007; 11(2): R47

## CRITICAL CARE OUTCOMES II

### A1008 Prognostic factors involved in poor outcome in postoperative resection of brain tumor in the first month

#### L.E. de la Cruz Rosario^1^, S.P. Gómez Lesmes^1^, J.C. García Romero^2^, A.N. García Herrera^1^, E.D. Díaz Pertuz^3^, M.J. Gómez Sánchez^1^, E. Regidor Sanz^1^, J. Barado Hualde^1^, A. Ansotegui Hernández^1^, J. Roldán Ramirez^1^

##### ^1^Complejo Hospitalario de Navarra, Intensive Care Medicine, Pamplona, Navarra, Spain; ^2^Complejo Hospitalario de Navarra, Neurosurgery, Pamplona, Navarra, Spain; ^3^Complejo Hospitalario de Navarra, Neurology, Pamplona, Navarra, Spain

###### **Correspondence:** L.E. de la Cruz Rosario – Complejo Hospitalario de Navarra, Intensive Care Medicine, Pamplona, Navarra, Spain

**Introduction:** Surgical resection of CNS tumors and the postoperative management in the ICU is challenging because of aggravated neurological impairment in the 14 % of patients. We analyzed the factors implicated in these outcomes.

**Objectives:** Analysis of the factors involved in poor outcome in the first month of the scheduled postoperative brain tumor resection

**Methods:** Cohort study, observational and retrospective. analyzed over the past 5 years, all patients underwent elective surgery for resection of brain tumor. We collect risk factors, demographic variables, related to the type of tumor and complications during surgery and in the postoperative ICU. We defined as unfavorable outcome in two points or more of the Canadian´s scale score (CSS). Comparative study by Student´s t-test for quantitative variables and using chi square test and Fisher test for qualitative variables. Variables with a p-value less than 0.1 are included in the multivariate analysis, using logistic regression. Data expressed by the mean, standard deviation (SD) and risk. Comparative study expressed by the mean difference, relative risk (RR), odds ratio, exponential (B) and their corresponding confidence intervals at 95 %.

**Results:** Data are collected from 271 patients. An unfavorable evolution is observed in 14 % of patients, 6.6 % by exitus and 7.4 % decline in score CSS. Factors associated with unfavorable outcome with p-value less than 0.1 are: Age 61.42 (SD 12.22) vs 53.47 (SD 14.87), mean differences 7.95, IC95 3.53 to 12.38, p-value 0.001; APACHE II 10,45 (SD 5,95) vs 6,3 (SD 3,99), mean differences 4,15, CI95 2,13 to 6,17, p-value 0.000; Karnofsky scale 67,89 (SD 14,72) vs 74,21 (SD 12,47), mean differences 6,31, CI95 1,93 to 10,69, p-value 0.005. Tenían Have the following risk: Cardiovascular risk factor (CRF) 17,8 % vs 10,2 %, RR 1,73, CI95 0,93 to 3,19, p-value 0,077; Malignant tumor 18,7 % vs 9,1 %, RR 2,05, CI95 1,08 to 3,91, p-value 0,023; Intraoperative bleeding 27 % vs 11,2 %, RR 2,47, CI95 1,36 to 4,45, p-value 0,003; Severe complications in the ICU 46 % vs 6,8 %, RR 6,75, CI95 3,80 to 11,97, p-value 0,000. Using logistic regression, the factors independently associated with unfavorable evolution are: diagnosis of malignant tumor (Exp (B) 3,318; CI95 1,273-8,653); surgical bleeding (Exp (B) 2,836; CI95 1,047-7,861) and occurrence of serious complications in ICU Exp (B) 10,301; CI95 4,342 to 24,439).

**Conclusions:** The unfavorable outcome after intervention on brain tumors has considerable prevalence. In our environment, the odds ratio of presenting unfavorable evolution is multiplied by at least 1.3 if the tumor is malignant lineage, at least 1.05 if excessive bleeding occurs during surgery and at least 4.3 if complications serious in ICU.

**References**

1. Predicting functional impairment in brain tumor surgery: the Big Five and the Milan Complexity Scale *P. Ferroli, MD ,1 M. Broggi, MD, PhD,1 S. Schiavolin, PsyD,2 et all. Neurosurg Focus 39 (6):E14, 2015

### A1009 An analysis of 3,173 patients transferred by helicopter emergency medical service (HEMS) from remote Tokyo islands, 2002–2014

#### H. Takahashi^1,2^, F. Kazutoshi^1^, Y. Okada^3^, W. Oobayashi^4^, T. Naito^1^

##### ^1^Juntendo University School of Medicine, General Medicine, Tokyo, Japan; ^2^Niijima, Tokyo National Health Insurance Clinic, Tokyo, Japan; ^3^Jichii Medical University, Pediatrics, Tochigi, Japan; ^4^Tokyo Metropolitan Government, Emergency Medical Services and Disaster Response Section, Tokyo, Japan

###### **Correspondence:** H. Takahashi – Juntendo University School of Medicine, General Medicine, Tokyo, Japan

**Introduction:** Helicopter emergency medical service (HEMS) has been well known as a crucial lifeline for people living in rural areas especially on remote islands. Located 200 km and 1,000 km away from the mainland respectively, the Izu islands and the Ogasawara islands are part of Tokyo. These islands are clearly separated into 3 populations; 200–500, 2,000-3,000 and 8,000. Accessibility of medical resources varied depending on the number of residents. Inpatient facilities and surgeries are available in islands where more than 8,000 people live. Despite numerous studies of HEMS, neither distance from the mainland nor accessibility of medical resources has been evaluated as factors influencing the medical condition of HEMS-transferred patients.

**Objectives:** We aimed to identify the influence of distance from the mainland or accessibility of medical resources on medical condition severity of HEMS-transferred patients from remote Tokyo islands.

**Methods:** All patients transferred by HEMS from remote Tokyo islands to hospitals on the mainland between 2002 and 2014 were retrospectively enrolled in this study. Medical conditions of patients were ranked according to severity: mild, moderate, severe, serious or fatal. We classified patients from islands 200 km and 1,000 km away from the mainland as short distance group and long distance group respectively. We also classified patient access to medical resources as easy access group (>8,000 residents) and difficult access group (200–3,000 residents) respectively. We adjusted for age, accessibility of medical resources and distance from the mainland by stratification. Statistical comparisons of severity were done between the mild group (mild + moderate) and severe group (severe + serious + fatal) using the Chi-square test.

**Results:** In total 3,173 patients were transferred by HEMS over the 12-year period. Medical severity of the patients; mild: 20 (0.6 %), moderate: 1,561 (49.2 %), severe: 1,353 (42.6 %), serious: 226 (7.1 %), and fatal: 2 (<0.1 %). In statistical comparison of severity, the severe group composed a significantly larger proportion of the short distance group compared with the long distance group [658/1,437 (45.8 %) vs 121/355 (34.1 %), *p < 0.001*] and likewise in the easy access group compared with the difficult access group [802/1,370 (58.5 %) vs 658/1,437 (45.8 %), *p < 0.0001*].

**Conclusions:** The medical condition severity of the HEMS-transferred patients varied depending on the distance from the mainland and medical resource accessibility. Shorter distance and easier access to medical resources contributed to a shift toward greater medical condition severity in patients transferred by HEMS from remote Tokyo islands.

**Reference**

Taylor CB, et al. Primary scene response by Helicopter Emergency Medical Services in New South Wales Australia 2008–2009. BMC Health Serv Res. 2012;12:402.

**Grant acknowledgment**

I am grateful to Dr. Okada and Dr. Obayashi for their valuable cooperation in my experiments.

**Table 57 (abstract A1009).** Characteristics of the remote Tokyo isilands

Islands characteristics	Total	>8,000 residents (n = 2)	200-3,000 residents (n = 9)	Izu islands (n = 9) (200 km away)	Ogasawara islands (n = 2) (1,000 km away)
Total population (%)	28744 (100.0)	17539 (61.0)	11,205 (39.0)	26,021 (90.5)	2,723 (9.5)
Age (%)					
≦ 14 years	3,540 (12.3)	2,175 (12.4)	1,365 (12.2)	3,143 (12.1)	398 (14.6)
15-64 years	17,397 (60.5)	10,348 (59.0)	7,049 (62.9)	15,306 (58.8)	2,091 (76.8)
≧ 65 years	7,807 (27.2)	5,016 (28.6)	2,791 (24.9)	7,575 (29.1)	2,091 (76.8)
Sex (%)					
Male	14,751 (51.3)	8,669 (49.4)	6,082 (54.3)	13,062 (50.2)	1689 (62.0)

**Table 58 (abstract A1009).** Characteristics of the HEMS-transferred patients

Patients Characteristics	Total	>8,000 residents (n = 2)	200-3,000 residents (n = 9)	Izu islands (n = 9) (200 km away)	Ogasawara islands (n = 2) (1,000 km away)
Number of patients (%)	3,173 (100.0)	1380 (43.5)	1,793 (56.5)	2,818 (88.8)	355 (11.2)
Age (%)					
≦ 14 years	172 (5.4)	62 (4.5)	110 (6.1)	140 (5.0)	32 (9.0)
15–59 years	2,071 (65.3)	922 (66.8)	1,150 (64.1)	1,910 (67.8)	161 (45.4)
≧ 60 years	930 (29.3)	396 (28.7)	533 (29.7)	768 (27.3)	162 (45.6)
Sex (%)					
Male	1,999 (63.0)	857 (62.1)	1,142 (63.7)	1,768 (62.7)	231 (65.1)

**Table 59 (abstract A1009).** Comparison of medical condition severity

	Distance from the mainland			Accessibility of medical resources		
Severity (%)	Short distance group (200 km)	Long distance group (1,000 km)	*p* Value	Easy access group	Difficult access group	*p* Value
Total	1,437	355		1,370	1,437	
Mild	10 (0.6)	3 (0.8)		7 (0.5)	10 (0.6)	
Moderate	769 (53.5)	231 (65.1)		561 (40.9)	769 (53.5)	
Severe	574 (39.9)	108 (30.4)		671 (49.0)	574 (39.9)	
Serious	83 (5.8)	13 (3.7)		130 (9.5)	83 (5.8)	
Fetal	1 (<0.1)	0 (0.0)		1 (<0.1)	1 (<0.1)	
Mild + Moderate (%)	779 (54.2)	234 (65.9)	<0.001	568 (41.5)	779 (54.2)	<0.0001
Severe + Serious + Fetal (%)	658 (45.8)	121 (34.1)		802 (58.5)	658 (45.8)	

### A1010 Comparison of short axis out-of-plane approach vs medial oblique in-plane approach for real time ultrasound guided internal jugular vein cannulation

#### D.K. Baidya, S. Maitra, R.K. Anand, B.R. Ray, M.K. Arora

##### All India Institute of Medical Sciences, Anesthesiology Critical Care and Pain Medicine, New Delhi, India

###### **Correspondence:** D.K. Baidya – All India Institute of Medical Sciences, Anesthesiology Critical Care and Pain Medicine, New Delhi, India

**Introduction:** Ultrasound guided internal jugular vein cannulation is recommended technique in current anaesthesia and intensive care practice. However, classic short axis view has inherent problem of needle visualization during venous access. In contrast, medial oblique view may enhance needle visibility during venipuncture and decrease overlap between IJV and carotid artery and thereby increase the safety of US guided IJV cannulation (1–2).

**Objectives:** To compare the safety and efficacy of medial oblique view and in-plane technique as compared to short axis view and out-of-plane technique during US guided IJV cannulation.

**Methods:** Two hundred patients aged between 18-50 yrs of either sex and American Society of Anesthesiologists' physical status I-II who were undergoing any surgery under general anaesthesia requiring an internal jugular vein cannulation, enrolled for this prospective randomized controlled trial. Three patients were excluded due to US machine malfunction. In patients of group M, IJV cannulation was performed with medial oblique probe position and in plane approach. In patients belonging to group S, IJV cannulation was done in out of plane approach with the US probe in short axis position. Primary outcome was needle and guide-wire visibility during procedure.

**Results:** Needle visibility (entire needle tract and needle tip) was significantly higher during IJV puncture in medial oblique probe position (68 of 98 patients in group M versus 40 of 99 patients in group S; p = 0.00002). Guide wire visibility during insertion was also higher when medial oblique probe position was used (59 of 98 in group M versus 34 out of 99; p = 0.00013). First insertion success rate for IJV puncture, incidence of posterior wall of IJV puncture and time to cannulation were similar both the groups.

No serious complications such as carotid artery puncture, haematoma formation and pneumothorax were reported.

**Conclusions:** Medial oblique view may increase safety of US guided IJV cannulation in comparison to short axis view by increasing needle visibility during puncture.

**References**

1. Dilisio R, Mittnacht AJ. The "medial-oblique" approach to ultrasound-guided central venous cannulation-maximize the view, minimize the risk. J Cardiothorac Vasc Anesth. 2012;**26**:982–4.

2. Baidya DK, Chandralekha, Darlong V, Pandey R, Goswami D, Maitra S. Comparative Sonoanatomy of Classic "Short Axis" Probe Position with a Novel "Medial-oblique" Probe Position for Ultrasound-guided Internal Jugular Vein Cannulation: A Crossover Study. J Emerg Med. 2015;48:590–6.

**Grant acknowledgment**

No grant was obtained.

**Table 60 (abstract A1010).** Characteristics of IJV cannulation

Parameters	Group M (n = 98)	Group S (n = 99)	Significance
Cannulation time (seconds)	128 [96–220]	134 [98–220]	p = 0.165
First insertion success rate	87/98	85/99	p = 0.538
Carotid artery puncture	1/98	0/99	
Posterior wall of IJV puncture	1/98	5/99	

### A1011 Good neurological outcome predictor in patients treated for out of hospital cardiac arrest

#### C. Ruffini^1^, L. Rota^1^, A. Corona^1^, G. Sesana^2^, S. Ravasi^2^, E. Catena^1^

##### ^1^ASST Sacco Fatebenefratelli, Milano, Italy; ^2^ASST Grande Ospedale Metropolitano Niguarda, Milano, Italy

###### **Correspondence:** C. Ruffini – ASST Sacco Fatebenefratelli, Milano, Italy

**Introduction:** Out of Hospital Cardiac Arrest (OHCA) is a time related condition occurring in Europe with a rate of 280,000 events per year. Resuscitation “*philosophy*” shifted from being *heart oriented* towards *brain oriented*^2^ since its delay may increase neurological deficits^1^. Recent studies are highlighting the role of the Bystander-CPR as a critical variable affecting OCHA neurological outcome.^3, 4^

**Objectives:** Two step prospective interventional observational study to assess the role of the Bystander*-*CPR in affecting neurological outcome in our OHCA population.

**Methods:** Data have been collected on the Metropolitan SOREU *(*Emergency & Urgency Regional Operative Room) database, serving the Metropolitan Area (5.000.000 people) of Milan. OHCA occurred in the first quarter of 2014 (step 1 phase) and 2015 (step 2 phase) were recruited. Demographics, causes and rhythms of presentation and neurological outcome - measured with *Cerebral Performance Category* scale - were collected. In between the two steps the the 2^nd^ edition of the Italian National Campaign, (*Settimana Viva!*) was organised to teach BLS-D to the population. Statistical analysis was performed using SPSS 20.

**Results:** A total of 1208 (step 1) and 1371 (step 2) patients experiencing OHCA were recruited. In the step 2 phase an increase of (I) OHCA (+11 %, p = 0.234), (ii) ROSC (+16 %, p = 0.11), (iii) OHCA (+3 %, p = 0.763) witnessed by a Bystander-CPR has been observed. A Cox´s proportional Hazard Model allowed us to assess that the presence of a Bystander-CPR (i) reduces of about one third the risk of extra-hospital death (HR: 0.37, 95 % CI: 0.28-0.51, p < 0.001) and above all (ii) is associate with a significantly better neurological outcome at discharge from hospital (p = 0.027 HR 0.51 0.27-0.93).

**Conclusions:** Despite the limitations, our study suggests that our educational campaign to train population in BLS-D may be efficacious. Actually, in step 2 phase, OHCA related risk of death significantly decreased as well as a better neurological outcome were recorded.

**References**

1. http://www.who.int/whosis/whostat/2010/en/- WHO Statistical Information System (WHOSIS) - World Health Statistics 2010

2. Cummins, R. O., Ornato, J. P., Thies, W. H., & Pepe, P. E. (1991). Improving survival from sudden cardiac arrest: the “chain of survival” concept. Circulation,83(5), 1832–1847

3. Wijdicks EF, Hijdra A, Young GB, Bassetti CL,Wiebe S. Practice parameter: prediction of outcome in comatose survivors after cardiopulmonary resuscitation (an evidence-based review): report of the Quality Standards Subcommittee of the American Academy of Neurology. Neurology 2006;67:203—10

4. Nakahara S., Tomio J., Ichikawa M., Nakamura F. et al.(2015) Association of Bystander Interventions with neurologically intact survival among patients with Bystander-Witnessed Out of Hospital Cardiac Arrest in Japan - JAMA 2015;314(3):247–254

### A1012 The microcirculation can be assessed at the bedside: the point-of-care microcirculation (POEM) grading system

#### D.N. Naumann^1^, C. Mellis^2^, S.L. Husheer^3^, J. Bishop^1^, M.J. Midwinter^1^, S. Hutchings^2^

##### ^1^NIHR Surgical Reconstruction and Microbiology Research Centre, Birmingham, United Kingdom; ^2^Kings College Hospital, London, United Kingdom; ^3^Heartfelt Technologies Ltd, Cambridge, United Kingdom

###### **Correspondence:** D.N. Naumann – NIHR Surgical Reconstruction and Microbiology Research Centre, Birmingham, United Kingdom

**Introduction:** There is a clear rationale for monitoring microcirculatory behaviour during shock since it is the anatomical location of oxygen and substrate exchange, and may not correspond to global haemodynamics. And yet despite over a decade of research and technological advances such monitoring has not reached clinical bedside utility. Analysis of the data is performed offline and too time consuming for clinical use. There is an urgent need for a system to assess the microcirculation at the bedside. We present a novel 5-point grading system (the Point-Of-carE Microcirculation (POEM) scoring system) that can be used at the bedside (using sublingual microcirculatory monitoring).

**Objectives:** To assess the inter-user variability of the novel POEM scoring system amongst doctors and nurses who may use such technology for clinical practice, and to benchmark POEM scores against traditional offline computer analysis.

**Methods:** The POEM score is an ordinal scale from 1 (worst) to 5 (best), and calculated based on assessment of 4 individual video clips. Online calculator found at: www.POEMscore.com. Thirty-two naïve study participants from two UK teaching hospitals (Birmingham and London) participated in a standardised 1-hour interactive training session in how to assign POEM scores based on microcirculatory video clips from sublingual incident dark field (IDF) videomicroscopy imaging. They were then asked to assign scores for 5 different video sequences (each of varying clinical status, played in a random order). They were blinded to clinical status. Inter-user consistency and agreement were assessed using intra-class correlation coefficient (ICC) analysis. Blinded expert POEM scores were also validated against offline computer analysis of the same clips using traditional microcirculatory parameters, and the time taken to assign each was recorded.

**Results:** Raters showed good inter-rater consistency (ICC 0.83, 95 % CI 0.626, 0.976) and agreement (ICC 0.815, 95 % CI 0.602, 0.974) for assigned POEM scores. Expert POEM scores correlated well with offline analysis but took far less time to assign (mean times of 2 minutes *versus* 44 minutes; *p* < 0.001).

**Conclusions:** A new 5-point ordinal scale of microcirculatory function has been tested amongst 'front line' emergency physicians and nurses at two large UK teaching hospitals, and has minimal inter-user variability, even after just 1 hour of training. POEM scores take a matter of minutes to assign, and correspond well to computer-analysis variables. We present for the first time a bedside microcirculatory grading system that is quick, reliable, and gives potentially meaningful clinical parameters that might guide resuscitation. Prospective randomised trials utilising goal directed therapy using the POEM score are required to test its real-life clinical utility.

### A1013 Splenic perfusion assessment by splenic Doppler resistive index during fluid challenge in cardiac surgery patients

#### F. Corradi^1^, C. Brusasco^1^, T. Manca^2^, A. Ramelli^2^, M. Lattuada^1^, F. Nicolini^2^, T. Gherli^2^, A. Vezzani^2^

##### ^1^Ente Ospedaliero Ospedali Galliera, Anestesia e Rianimazione, Genova, Italy; ^2^Azienda Ospedaliero-Universitaria di Parma, Dipartimento Cardio-Nefro-Polmonare, UO di Cardiochirurgia, Parma, Italy

###### **Correspondence:** F. Corradi – Ente Ospedaliero Ospedali Galliera, Anestesia e Rianimazione, Genova, Italy

**Introduction:** To monitor right-sided intravascular volume status and optimize cardiac preload is a major issue especially in critical care medicine.

**Objectives:** To assess splenic Doppler resistive index variations in response to fluid challenge, in a population of cardiac surgery patients receiving mechanical ventilation and requiring a fluid challenge.

**Methods:** Splenic Doppler was used to measure resistive index before and after fluid challenge.

**Results:** Of the 27 patients included, 14 (52 %) met our definition for fluid challenge responsiveness, that is at least a 15 % increase in cardiac index. After fluid challenge, cardiac index increased from 2.3 ± 0.6 L/min/m^2^ to 2.7 ± 0.7 mL L/min/m^2^ (p < 0.001). Cardiac index changes after fluid challenge were +35 % (±19 %) in fluid challenge responders and +6 % (±5 %) in fluid challenge non-responders. Splenic Doppler resistive index was reduced after fluid challenge in responders (0.66 [±0.12] before and 0.56 [±0.12] after fluid challenge; p < 0.001) while was unchanged in non-responders (0.65 [±0.07] before and 0.65 [±0.08] after fluid challenge; p = 0.601). Splenic Doppler Resistive Index changes after fluid challenge were +16 % (±9 %) in fluid challenge responders and +4 % (±3 %) in fluid challenge non-responders.Cardiac index variations (DCI) showed a strong correlation with splenic Doppler resistive index changes (DSDRI) after fluid challenge in the overall population (r = 0.85, p < 0.001).

**Conclusions:** Systemic hemodynamic changes induced by fluid challenge translate into splenic Doppler mechanical ventilated cardiac surgery patients

### A1014 Vocal cord palsy; prevalence and consequences in a cardiothoracic unit

#### A. Young

##### Royal Brompton and Harefield Trust, Speech and Language Therapy, London, United Kingdom

**Introduction:** Vocal cord palsy is a known postoperative complication following cardiothoracic surgery.^1, 2.^ Although the incidence is relatively low its existence cannot be ignored and thus its identification necessary in order to avoid any further complications and maintain patient wellbeing. This study aims to look at the incidence of vocal cord palsy following cardiothoracic surgery in a tertiary referral centre and highlight the importance of the speech and language therapist's role in working with this cohort.

**Objectives:** To measure the incidence of vocal cord palsy post cardiac and thoracic surgery and to identify the consequent effects.

**Methodology.** A retrospective analysis, within a tertiary cardiothoracic centre. Data for all patients who underwent either a cardiac or thoracic surgical procedure between December 2015 and April 2016 and were referred to speech and language therapy (SLT) was collected.

Vocal cord palsy was identified by Fibreoptic Endoscopic Evaluation of Swallowing (FEES) or Bronchoscopy.

**Results:** A total of 25 patients were seen by the SLT. Patients with vocal cord palsy were identified by FEES and Bronchoscopy; 83 % and 17 % respectively. Six patients assessed presented with vocal cord palsy; patients were post cardiac surgery (4/6) and post thoracic surgery (2/6).

The consequence of vocal cord palsy was dysphonia in all the patients and dysphagia in two thirds of patients. The median duration that patients experienced dysphagia was 12 days (range 4–38 days) and dysphonia was 21 days (range 11–38).

**Conclusion:** This review highlights the high prevalence of vocal cord palsy post cardiothoracic surgery. Vocal cord palsy led to high levels of dysphagia and dysphonia. Early identification of these is imperative to ensure patient safety and optimise recovery and quality of life.

**References**

1 Bozinovski; J & Coselli JS. Outcomes and Survival in Surgical Treatment of Descending Thoracic Aorta With Acute Dissection. Presented at the Fifty-third Annual Meeting of the Southern Thoracic Surgical Association, Tucson, AZ, Nov 8–11, 2006

2 Hamdan; AL, Roger V. Moukarbel; RV, Farhat; F & Obeid; M. Vocal cord paralysis after open-heart surgery. Eur J Cardiothorac Surg (2002) 21 (4): 671–674.

### A1015 Tranthoracic lung ultrasound in a neurotraumatologic ICU

#### A. Fernández Carmona, A. Iglesias Santiago, L. Navarro Guillamon, M.J. García Delgado

##### Complejo Hospitalario de Granada, Intensive Care Unit, Granada, Spain

###### **Correspondence:** A. Fernández Carmona – Complejo Hospitalario de Granada, Intensive Care Unit, Granada, Spain

**Introduction:** Pleuropulmonary ultrasound is an increasing important tool in the treatment of critically ill patients, because it is immediately bedside available, its safety and applicability; helping in the detection of pleural effusion, pneumothorax and for the diagnosis and monitoring of lung consolidation and atelectasis. In addition it complements the hemodynamic information given by echocardiography about extravascular lung water, and detects changes in the normally aerated lung in different pathologies. We describe the experience with transthoracic lung ultrasonography (LUS) in a neurotraumatologic ICU of a tertiary care hospital, in terms of purpose, usefulness, more frequent findings and safety of LUS.

**Methods:** A descriptive prospective single-center study of patients admitted to the ICU in whom a LUS was performed and described in clinical history from March 2015 to February 2016.

Examination of the lung and pleura was performed following the international recommendations (8 thoracic areas) in supine position. The equipment used was: Siemens Acuson Cypress (5 MHz Cardiac-probe) and CAPASEE II Toshiba (abdominal-Convex-probe 3.75 MHz), the choice of each device depended on the physician and whether the study was completed with echocardiography and/or abdominal ultrasound. All of these studies were performed by intensivist. All LUS were compared with other radiologic studies (18,8 % by TAC).

**Results:** During this period a total of 86 LUS were performed. Most patients were male (69,4 %) with a mean age of 49,7 ± 18,5 years. The most common reason for ICU admission was multiple trauma (48.8 %) followed by neurocritical pathology (27.9 %). 62,8 % of LUS were performed while patients were under invasive mechanical ventilation and 9,3 % noninvasive mechanical ventilation. 37,0 % of LUS were considered urgent.

The most frequent ultrasound diagnoses were pneumonia (16.5 %), pneumothorax (12,9 %), haemoneumothorax plus pulmonary contusion (12,1 %), and pulmonary contusion alone (10.8 %). In 17,6 % no pathology was detected when LUS was made as a hemodynamic and/or respiratory monitoring or during E-FAST realization.

In 5 cases LUS had higher sensitivity than chest radiography, detecting 3 pneumothoraces, 1 heart failure with LUS congestive lung pattern and ELWI > 10 (PiCCO monitoring) and 1 bibasal pneumonia.

77,0 % of LUS were considered as an useful tool, guiding decision making and patient treatment. In 39,5 % patients decision on pleural drain or not depended on LUS, as well as to guide the insertion of pleural drainage.

**Conclusions:** Pleuropulmonary ultrasound is a useful tool in the management of multi trauma and neurocritical patients in emergency situations. LUS also has become an everyday tool for evaluation and guide clinical practice at intensive care units.

### A1016 Effects of preoperative conditions on health-related quality of life after cardiac surgery

#### M. Delgado-Amaya^1^, E. Curiel-Balsera^1^, L. Rivera-Romero^1^, E. Castillo-Lorente^2^, F. Carrero-Gómez^3^, E. Aguayo-DeHoyos^4^, ARIAM registry of adult cardiac surgery

##### ^1^Hospital Regional Málaga, Málaga, Spain; ^2^Complejo Hospitalario de Jaén, Jaen, Spain; ^3^Hospital Puerta del Mar, Cadiz, Spain; ^4^Hospital Virgen de las Nieves, Granada, Spain

###### **Correspondence:** M. Delgado-Amaya – Hospital Regional Málaga, Málaga, Spain

**Introduction:** Factors influencing the postoperative health-related quality of life (HRQOL) after cardiac surgery have not been well described yet, mainly in the older people.

**Objectives:** To study the relationship between preoperative conditions, type of surgery and the HRQOL in a cohort of 4430 patients, recluted at 11andalusian hospitals.

**Methods:** Observational, prospective and multicenter study of patients included in the ARIAM registry of adult cardiac surgery. We analized clinical variables, surgery data, outcomes and scores (SAPS3 and EuroSCORE). We have interviewed 1382 patients with the SF-36 questionnaire during the follow up (medium 1291+/− 166 days).

**Fig. 86 (abstract A1016).**
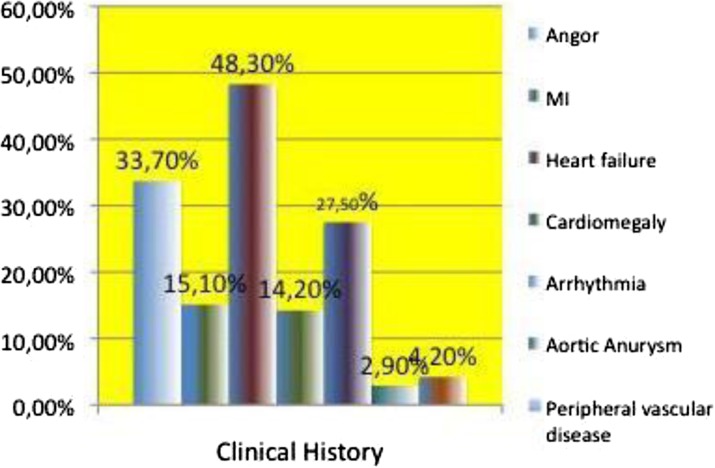
Medical history

**Results:** Medium age was 63,73 years (SD 12,69), 61,2 % were male. Medium weight was 76,6 kg (SD 14,21). BMI was 28,4 (SD 4,7). 20.4 % were over 75 years old at the moment of the surgery. Figure 86 shows previous cardiac clinical history

**Fig. 87 (abstract A1016).**
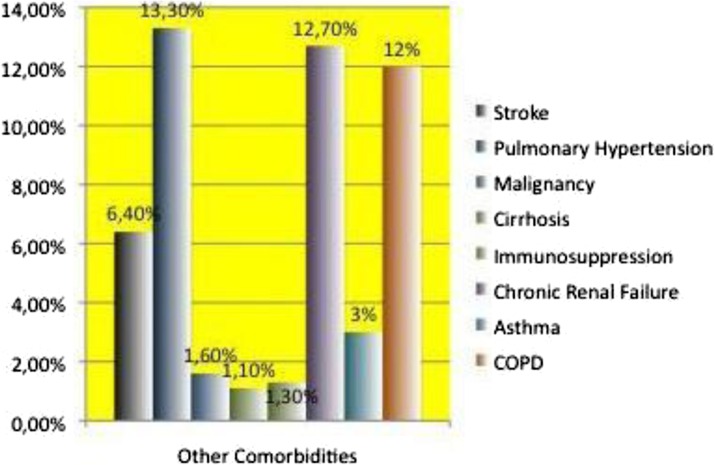
Other comorbidities

Figure 87 shows other relevant comorbidities.

It was first surgery in 90.5 % of the patients. 68,7 % of the cases were valvular surgery (with or without CABG); 21,4 % were isolated CABG; 5,1 % thoracic aortic surgery; 5,4 % were other surgeries (congenital disease surgery, post AMI complications, pericardiectomy). Tables 61 and 62 summarizes the results of the application of SF-36 questionnaire in the 1382 patients interviewed in the follow up

In the comparative analysis we found worse QOL in women than men (p = 0,0001), and a negative correlation between age and QOL (p = 0,0001). Women in our study were significantly older than men (p = 0,0001). We found no differences between the type of surgery and the postoperative QOL, or between surgery or extracorporeal circulation duration and QOL. There was a relationship between NYHA degree during the follow up and the SF 36 Health score

Prognostic scores showed an inverse relationship with QOL, but with a low correlation; Pearson coefficient −0,22 (Euroscore), −0,28 (SAPS 3).

**Conclusions:** In our study, involving 11 hospitals in Andalusia (South Spain), 71,5 % of cardiac surgery patients didn´t show any activity limitation or only a slight limitation before 3 years of follow up. Perceived quality of life decreases as age increases or worsens the functional status of the patients.

**References**

1. Kurfirst V et al. Health-related quality of life after cardiac surgery-the effects of age, preoperative conditions and postoperative complication. Journal of Cardiothoracic Surgery 2014, 9:46

**Grant acknowledgment**

This study has been done thanks to a scholarship of the Progreso y Salud Foundation.The data has been obtained from the ARIAM registry of adult cardiac surgery.

**Table 61 (abstract A1016).** Functional status

	Frecuency	Percentage
Alive, functional degree I. Without activity limitation	661	47,9 %
Alive, functional degree II Slight activity limitation	326	23,6 %
Alive, functional degree III Marked activity limitation	219	15,9 %
Alive, functional degree IV Symptons, pain at rest	175	12,7 %

**Table 62 (abstract A1016).** Statistical data

	D1 Phsysical Functioning	D2 Limited due to physical problems	D3 Limited due emotional problems	D4 Vitality, energy, fatigue	D5 Mental health	D6 Social Role	D7 Pain	D8 General health
Media SD	62,4946 28,08800	45,0579 49,61369	65,5322 47,54353	55,9739 20,30055	68,2171 12,63486	86,1785 22,52858	71,2455 27,72203	48,5807 15,87013

**Fig. 88 (abstract A1016).**
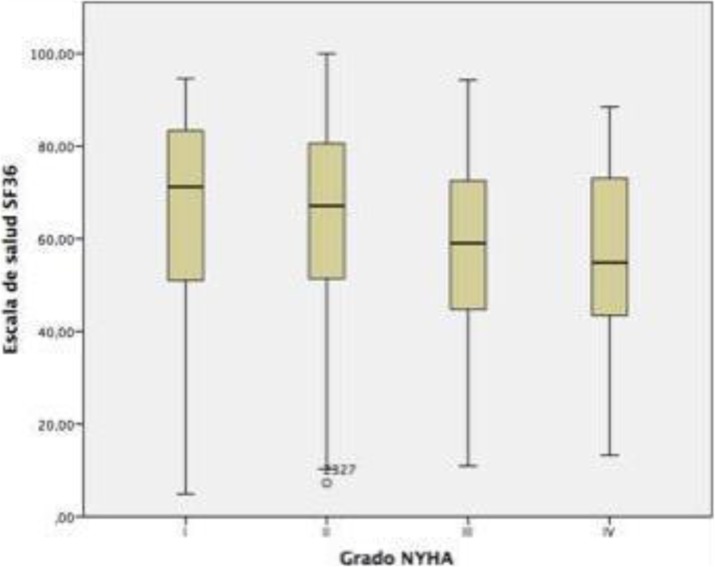
Relationship SF36/NYHA

### A1017 ICU management of severe acute pancreatitis in a tertiary referral HPB centre: a 2 year review supporting the role of early tertiary ITU referral in optimising outcomes

#### A.J. Healey MD FRCSEd(Gen.Surg), C. Cameron MBChB, L.R. Jiao MD FRCS(Gen.Surg), R. Stümpfle MRCP FRCA FFICM

##### Hammersmith Hospital, Imperial College Healthcare NHS Trust, Department of Surgery and Cancer, London, United Kingdom

###### **Correspondence:** A.J. Healey MD FRCSEd(Gen.Surg) – Hammersmith Hospital, Imperial College Healthcare NHS Trust, Department of Surgery and Cancer, London, United Kingdom

**Introduction:** In 2012, an international multidisciplinary consultation formulated a determinant based classification of acute pancreatitis severity to rival or replace the 2012 updated Atlanta classification, based on the actual local and systemic determinants of severity.

**Objectives:** To apply the classification to all ITU admissions at a tertiary HPB referral centre and assess its potential role in determining which patients require centralised specialist HPB ITU care and those who may benefit from local itu support.

**Methods:** A review of patients admitted to ITU with a primary diagnosis of acute pancreatitis at Hammersmith Hospital, Imperial College Healthcare Trust between 1/1/2014 and 31/12/2015 was performed. Demographics such as age, sex, ethnicity and BMI were recorded. Outcome parameters included total ITU stay, time on organ support, time in ITU prior to tertiary unit transfer, (for external referrals) and time to first radiological investigation and first surgical/interventional radiological (IR) intervention.

**Results:** 17 patients were admitted to Hammersmith ITU with severe or critical acute pancreatitis. 17.6 % patients had single, 17.6 % patients had two-organ and 29.4 % patients had three-organ support. 35.3 % had no organ support. All patients received intravenous antibiotics, 70 % more than 1 and 53 % more than 5 different agents. Median ITU stay was 29.79dys, but there was a longer median overall ITU stay when comparing outside ITU-ITU referrals to those admitted directly to the tertiary unit, 58dys (IQR 9-89dys) vs. 22dys (9.9-75dys) respectively, (p > 0.05). Furthermore, all critical acute pancreatitis patients referred from outwith tertiary ITU, had surgical or IR intervention prior to transfer. Mean time lag between referral and transfer to tertiary ITU care was 17 days. All patients transferred severe or critical pancreatitic patients had early CT imaging, admission to ITU unit, prior to intervention and/or transfer.

**Conclusions:** A new acute pancreatitis classification based on actual local and systemic determinants has been adopted since 2012. We reviewed ITU admissions at a Specialist HPB referral centre and found that all patients classified as having severe or critical acute pancreatitis had early complex surgical interventions at the satellite hospital and a longer overall median ITU stay. A greater emphasis on adoption of this classification system and streamlined referral route from 'spoke' ITUs to a central 'hub' subspecialty HPB ITU may facilitate earlier intervention planning in a centralised tertiary MDT in those acute pancreatitic patients and improved outcomes, particularly shorter ITU stay.

**References**

1. Bansal SS, Hodson J, Sutcliffe RS, Marudanayagam R, Muiesan P, Mirza DF, Isaac J, Roberts KJ, Performance of the revised Atlanta and determinant-based classifications for severity in acute pancreatitis., Br J Surg. 2016 Mar;103(4):427–33, Epub 2016 Jan 25

### A1018 CRISTALLOIDS VS STARCH IN THE OUTCOME OF A SEPTIC SURGICAL PATIENTS COHORT

#### A. Pérez, S. Martin, O. Lopez del Moral, S. Toval, J. Rico, C. Aldecoa

##### Hospital Rio Hortega, Valladolid, Spain

###### **Correspondence:** A. Pérez – Hospital Rio Hortega, Valladolid, Spain

**Background and goal of study:** Fluid therapy is one of the most important targets of the resuscitation in septic patients, however, its use is controversial. Our aim is the review of the differences in fluid therapy in patients with sepsis, severe sepsis or septic shock in the intensive care unit among the time. Furthermore, we want to know the relationship between fluid therapy and the mortality, the development of acute kidney injury (AKI) and the use of renal replacement therapy (RRT)

**Materials and methods:** A retrospective, observational study of patients treated in our ICU with criteria of sepsis, severe sepsis and septic shock during the last five years. 273 adult patients were included. Variables were: demographic data, comorbidity, criteria of sepsis, APACHE II and SOFA within 24 hours, AKI, RRT, and the fluid therapy used that was divided in 4 groups: starch, starch + gelatine, crystalloids and crystalloids + albumin. The association between fluid therapy, mortality, AKI and RRT were analysed using test square Chi, with levels of confidence of 95 % and levels of significance of p < 0,05.

**Results:** We observed significant differences (p = 0.03) by associating fluid with kidney failure, 41.63 % developed AKI, the majority being treated with crystalloid + albumin (49.30 %) and crystalloid (48.68 %), followed by the group of starch + gelatine (27 %) and starch (28.89 %).

We obtained significant differences (p = 0.01) relating the fluid and the use of RRT. 21,03 % of patients were treated with RRT of which 34,72 % and 18,75 % received crystalloid + albumin and crystalloid respectively, the starch were 13,04 % and 12.20 % starch + gelatine. Mortality was 16,61 % with no significant differences (p = 0.175) in terms of mortality in our ICU related with type of fluids.

**Conclusion:** Significant differences have been found in correlation with the type of fluid and the development of AKI, also with the use of renal replacement therapy. The group with major incidence of AKI and RRT has been the group with crystalloids and albumin as fluid therapy following by the group of crystalloids. Not significant differences in terms of mortality have been found. Other variables as the fluid balance would be taking into account in a bigger sample size.

**References**

1. Asghar Rastegar. MD. Rational Fluid Therapy for Sepsis and Septic Shock; What Do Recent Studies Tell Us? Review Article. Archives of Iranian Medicine, Volume 18, Number 5, May 2015.

2. R. P. Dellinger, Mitchell M. Levy, Andrew Rhodes, et al. Surviving Sepsis Campaign: International Guidelines for Management of Severe Sepsis and Septic Shock, 2012. Intensive Care Med (2013) 39:165–228.

3. B. Rochwerg, W. Alhazzani, A. Gibson, et al. Fluid type and the use of renal replacement therapy in sepsis: a systematic review and network meta-analysis. Int Care Med, April 2015. DOI. 10.1007/s00134-015-3794

### A1019 Assessment of emergency intensive care unit calls from other departments

#### K. Oguzhan, O. Demirkiran, M. Kirman, S. Bozbay, M.E. Kosuk, G. Asyralyyeva, M. Dilek, M. Duzgun, S. Telli, M. Aydin, F. Yilmazer

##### Istanbul University Cerrahpasa Medical Faculty, Anesthesiology and Intensive Care, Istanbul, Turkey

###### **Correspondence:** O. Demirkiran – Istanbul University Cerrahpasa Medical Faculty, Anesthesiology and Intensive Care, Istanbul, Turkey

**Introduction:** In some hospital intensivits called for the severe cases in the wards. In this study we aimed to determine the emergency calls, reasons for consultation requests, whether the cases need intensive care unit admission or not, and admission rate to our unit.

**Methods:** The clinics in our hospital divided in two part depend on their localization for emergency calls. Our ICU has 6 beds, and close to emergency department, and responsible for these clinics: Emergency medicine, chest medicine, ortopedics, neurology, psychiatry, obstetrics and gynecology, gynecological oncology, physiotherapy, and radiation oncology. The emergency calls for this clinics between 2015–2016 were analyzed.

**Results:** In one year 599 patient (54,9 % male, 45,1 % female) have been consultated by our staff. The mean age was 56,86 year. Departments asking for consultaion were as follow: 42,9 % Emergency Medicine, 25,3 % Emergency Surgery, 11,6 % Chest Medicine, 5,3 % Neurology, 5 % Gynecological Oncology, 9.9 % others.

The most common reasons calling for consultation were respiratory insufficency (31,7 %), sepsis/septic shock (10,2 %), and cardiac arrest (9,1 %).

The interventions performed during consultation were as mostly intubation (44,9 %), and CPR (8,8 %). 57 % of the patient had need intensive care admission, 5,6 % had need surgical operation, and 33,5 % recommended to stay in ward., 4,8 % of the consulted patients died.

The patients who need intensive care transferred to ICU's, 29,4 of them admitted to an ICU in our hospital (20,9 % general ICU, 4,6 % emergency ICU, 3,4 % neuro ICU, 0,2 % pediatric ICU), but 70,6 % of them send to an other hospital for ICU care.

**Conclusion:** Concsultation for the severely ill cases in the wards take very much time of the intensivits. It was seen that consultation request was mostly emergent and due to respiratory problems and sepsis. Maybe we need another system like rapid response team for decrease the insivists work and decrease the mortality amd morbidity. The limitation of the beds in ICU is one of the most important problem, and there must be more empty beds for inhospital emergencies.

## SEPSIS DIAGNOSIS

### A1020 An external validation study of the qSOFA score to predict in-hospital mortality in medical patients with infection and derivation of a new enhanced score using automatically available variables: news-hazard

#### L.E. Hodgson^1,2^, B.D. Dimitrov^1^, C. Stubbs^3^, L.G. Forni^4,5^, R. Venn^2^

##### ^1^University of Southampton, Faculty of Medicine, Southampton, United Kingdom; ^2^Western Sussex Hospitals NHS FT, Worthing, United Kingdom; ^3^University of Brighton, Brighton, United Kingdom; ^4^Royal Surrey County Hospital, Intensive Care, Guildford, United Kingdom; ^5^University of Surrey, Guildford, United Kingdom

###### **Correspondence:** L.E. Hodgson – University of Southampton, Faculty of Medicine, Southampton, United Kingdom

**Introduction:** Early diagnosis of sepsis is crucial. In updated sepsis consensus definitions “Quick SOFA” (qSOFA) score has been proposed as a clinical prediction rule (CPR) to consider sepsis in a patient with suspected infection.^1^ qSOFA combines three physiological variables (tachypnoea, altered mental state and hypotension) and accurately predicts in-patient mortality in original derivation and external validation cohorts.

**Objectives:** Firstly, externally validate performance of qSOFA at admission to hospital to predict mortality using a cohort of medical patients with infection. Secondly, investigate clinical and laboratory parameters at admission that could contribute to improved stratification of patients with severe infection.

**Methods:** A historical cohort study was performed at two UK adult acute medical units (2013–5). All patients were included based on a final coded diagnosis of acute infection and a culture (such as blood or urine) sent for testing at admission. Primary outcome was in-hospital mortality. Alert on the AVPU scale was used rather than altered GCS to calculate qSOFA as GCS is not in routine use at the two hospital sites. Predictive performance of qSOFA was compared to the National Early Warning Score (NEWS). Exclusion criteria included: direct admission from Emergency Department to ICU, a stay < 1 night, age < 18, neutropenia, HIV, metastatic malignancy and palliative care. Additionally, multivariable logistic regression was performed to investigate additional predictors of mortality in the cohort using only variables electronically available at admission (blood parameters, past coded history and physiological observations).

**Results:** Of 24,706 patients, 5,351 met the inclusion criteria for infection. Mortality was 8.7 % (n = 464), 5 % of the patients were escalated to ICU and 6 % had bacteraemia. Discrimination of mortality, assessed by area under the receiver operating characteristic curves (AUC_ROC_) were: qSOFA 0.61 (95 % CI 0.58-0.64) and NEWS 0.65 (0.62-0.68). To predict mortality a cut-off score of 2 points for qSOFA was very specific (98 %) but not sensitive (8 %). Following multivariable logistic regression a new CPR to predict mortality was constructed. NEWS-**HAZARD** combines the NEWS with blood parameters and prior history (HAZARD = **H**eart, **H**epatic failure, **AZ**otaemia, **A**lbumin and **R**ed cell **D**istribution width) with an AUC_ROC_ of 0.77 (0.75-0.79) to predict mortality in this cohort.

**Conclusions:** In a cohort of medical patients with suspected infection, qSOFA score at admission did not accurately discriminate for mortality. A new multivariable CPR (NEWS-HAZARD) combining coded history with physiological and blood parameters improved discrimination for in-hospital mortality.

**References**

1. Seymour CW, et al. JAMA 2016;315:762–74.

**Fig. 89 (abstract A1020).**
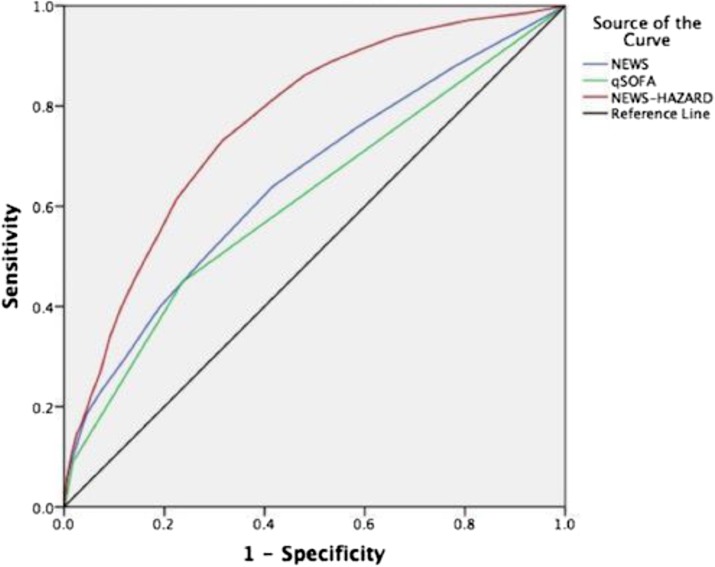
AUCROCs qSOFA, NEWS, NEWS-HAZARD

### A1021 Evaluation of SIRS and qSOFA criteria in predicting outcome for sepsis in a UK hospital

#### D. Vedage, S. Shawaf, P. Naran, N. Sirisena, J. Kinnear

##### Southend University Hospital, Essex, United Kingdom

###### **Correspondence:** D. Vedage – Southend University Hospital, Essex, United Kingdom

**Introduction:** Sepsis has recently been redefined as ´life-threatening organ dysfunction caused by a dysregulated host response to infection´. It is one of the leading causes of mortality internationally. Earlier identification of sepsis means more timely management, reduced length of hospital admission, and prevention of septic shock; ultimately reducing sepsis associated mortality. (1)

There is currently no standard diagnostic test for sepsis. Distinguishing sepsis from alternative, uncomplicated infections is pertinent to ensuring an appropriate clinical approach. Systemic inflammatory response syndrome (SIRS) criteria have been used since 1992 to define sepsis (Sepsis 1.0). However they have been found to lack sensitivity or specificity.

The quick sequential organ failure assessment score (qSOFA) is an emerging initial assessment method that uses three simple bedside criteria to measure organ dysfunction; altered mental status, respiratory rate ≥22 and systolic blood pressure ≤100 mmHg. The aim is to facilitate earlier recognition of sepsis outside of ITU by prompting the clinician to think, and adequately screen for sepsis.

**Objectives:** To compare the efficacy of qSOFA relative to SIRS in screening for sepsis by comparing outcomes in each group, in order to evaluate this as a screening tool.

**Methods:** This is a retrospective study of 341 patients with a diagnosis of sepsis (ICD-10 codes A40 and A41) at an acute UK hospital, between April 2013 and March 2014. Outcomes indicating severity of sepsis including ITU admission, hospital length of stay in surviving patients, organ dysfunction and 30 day mortality were compared with whether they met either SIRS or qSOFA criteria, or both.

**Results:** 19.9 % (68) patients did not meet either criteria and 40.8 % (139) met both. 22.9 % (78) patients were SIRS positive but qSOFA negative, while 16.4 % (56) were qSOFA positive but SIRS negative. 8.33 % of patients who were SIRS negative/qSOFA positive were admitted to ITU compared to 4.76 % who were SIRS positive/qSOFA negative. The length of stay (LOS) in surviving patients was significantly longer (p 0.02) in SIRS negative/qSOFA positive compared to SIRS positive/qSOFA negative cohorts. There is a statistically significant difference in 30 day mortality rates when comparing between all 4 groups (p = 0.000).

**Conclusions:** Our results suggest that qSOFA and SIRS perform differently in identifying the septic patient. Outcomes are worse for patients who are SIRS negative/qSOFA positive compared to SIRS positive/qSOFA negative patients. This suggests qSOFA may be a better indicator in identifying the septic patient with strong predictive validity and provides a quick and reproducible bedside prompt to further investigation and management of sepsis.

**References**

1. Singer, M., et al., The Third International Consensus Definitions for Sepsis and Septic Shock (Sepsis-3). JAMA, 2016. 315(8): p. 801–810

### A1022 Improving stratification of patients with suspected bacteraemia at admission to hospital with a new clinical prediction rule: the BALDUS score

#### B.D. Dimitrov^1^, L.E. Hodgson^1,2^, C. Stubbs^3^, L.G. Forni^4,5^, R. Venn^2^

##### ^1^University of Southampton, Faculty of Medicine, Southampton, United Kingdom; ^2^Western Sussex Hospitals NHS FT, Worthing, United Kingdom; ^3^University of Brighton, Brighton, United Kingdom; ^4^Royal Surrey County Hospital, Intensive Care, Guildford, United Kingdom; ^5^University of Surrey, Guildford, United Kingdom

###### **Correspondence:** L.E. Hodgson – University of Southampton, Faculty of Medicine, Southampton, United Kingdom

**Introduction:** Bacteraemia is associated with high mortality and suspicion of significant infection frequently prompts a blood culture (BC). However, results takes several days and contamination is common. Initial risk stratification for suspected infection has two main aims: (i) promote appropriate intensive interventions in patients at highest risk and (ii) improve microbiological stewardship and reduce unnecessary interventions. To date, only few strategies are described that employ hospital electronic records to automatically help achieve these aims.

**Objectives:** (i) Externally validate the performance of two clinical prediction rules (CPRs), qSOFA and NEWS, to predict bacteraemia at admission. (ii) Further investigate electronically available demographic, clinical and laboratory parameters in patients who had a BC sent at admission that could contribute to improved bacteraemia prediction.

**Methods:** A historical cohort study (2013–15) was performed at two UK adult acute medical units (AMUs). We extracted electronically captured physiological observations, laboratory parameters and previously coded patient history for all patients. Exclusions: direct admission from emergency department to intensive care (ICU), a stay < 1 night, age < 18, metastatic malignancy, neutropenia, HIV and palliative care. Multivariable logistic regression was used to identify independent predictors of bacteraemia. Discrimination performance was assessed by the area under the receiver operating characteristic curve (AUCROC).

**Results:** Of 2,844 patients with BCs drawn, 314 (11 %) had true bacteraemia, with a further 170 considered to have a contamination culture. The most common organisms were *E. Coli* (45 %), *S. Pneumoniae* (14 %) and *S. Aureus* (10 %). The group with bacteraemia had significantly higher mortality, escalation to ICU and length of stay (p < 0.05) than those without bacteraemia. Amongst physiological variables only systolic blood pressure (SBP) and shock index (heart rate/SBP) differed significantly between groups; temperature did not.

**Table:** 

Characteristic	True bacteraemia	No bacteraemia	P value (X ^2^, T-test or Mann-Whitney U)
Age (mean ± SD)	75 ± 15	69 ± 20	0.001
Died	13 %	7.5 %	0.001
Shock index (HR/SBP)	0.75 ± 0.23	0.72 ± 0.20	<0.001
Lymphocytes	0.89 ± 1	1.4 ± 6.3	<0.001
Urea	10.6 ± 7	8.3 ± 7	<0.001
Albumin	30 ± 6	33 ± 6	<0.001
Bilirubin	27 ± 30	19 ± 25	<0.001
Platelets	220 ± 100	256 ± 110	<0.001
Diabetes	26 %	19 %	0.004

*[Demographics & Clinical parameters at admission]*

Discrimination by qSOFA (AUC_ROC_ 0.55 [95 % CIs 0.52-0.59]) and NEWS (0.55 [0.51-0.58]) was poor. Independent predictors (at admission) in the multivariable model were **B**ilirubin/platelet ratio, **A**lbumin, **L**ymphopenia, **D**iabetes, **U**rea, and **S**hock index, which were combined into a new clinical rule (**BALDUS** score). The BALDUS score had a AUC_ROC_ 0.72 (0.69-0.75) and for calibration the Hosmer-Lemeshow test p = 0.442.

**Conclusions:** A combination of laboratory parameters and past history predicted true bacteraemia at hospital admission. The BALDUS score, using only data collected automatically, has the potential to be automated on existing hospital electronic systems, with subsequent ability to flag high-risk patients.

**Fig. 90 (abstract A1022).**
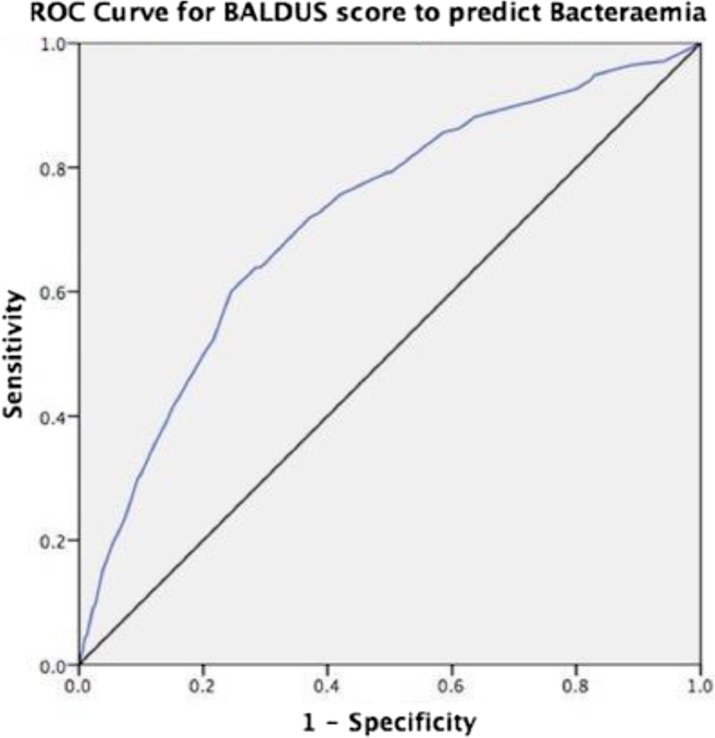
BALDUS_Bacteraemia

### A1023 Quick diagnose of pneumococcal meningitis in adults. Sensitivity and specificity of the *Streptococcus pneumoniae* antigen in CSF

#### J. Gonzalez Londoño^1^, C. Lorencio Cardenas^1^, A. Sánchez Ginés^1^, C. Murcia Gubianas^1^, E. Clapes Sánchez^2^, J.M. Sirvent^1^

##### ^1^Dr Josep Trueta University Hospital, Intensive Care Unit, Girona, Spain; ^2^Dr Josep Trueta University Hospital, Clinical laboratory, Girona, Spain

###### **Correspondence:** J. Gonzalez Londoño – Dr Josep Trueta University Hospital, Intensive Care Unit, Girona, Spain

**Objectives:** Despite not being one of the most prevalent infections in adults, acute meningitis has a high morbidity and mortality, being in many cases a neurological emergency that can end up requiring ICU admission. One of the most common etiological agents is *S. pneumoniae*. Early etiologic diagnosis is important in order to administer a targeted treatment and establish prognosis.

Our goal is to determine the sensitivity and specificity of the detection of *S. pneumoniae* antigen in CSF in the diagnosis of severe pneumococcal meningitis in adults admitted to the ICU.

**Materials and methods:** We present 73 patients with the clinical diagnosis of CNS pathology admitted to the ICU of a tertiary hospital, during the period between 2009 and 2015. In all patients, a pneumococcal antigen by immunochromatography ( BinaxNOW® test ) in CSF was performed. The results of this test, CSF culture, Gram staining and detection of pneumococcal CRP in CSF were analyzed. Pneumococcal meningitis was diagnosed in patients who presented compatible clinical findings and CSF culture and/or positive CRP for *S. pneumoniae*.

In 14 cases, the diagnosis of pneumococcal meningitis by culture or pneumococcal CRP in CSF was confirmed. In all cases, pneumococcus antigen was positive. Therefore, there were no false negatives. In 59 cases pneumococcal meningitis was not diagnosed, being the final diagnosis a non-pneumococcal bacterial meningitis or another pathology. In all cases, pneumococcus antigen was negative. Therefore, there were no false positives. In conclusion, in our sample of 72 patients, the sensitivity and specificity of the test for *S. pneumoniae* antigen in CSF was 100 %. The PPV and NPV were also 100 %.

**Conclusions:** In our series, the sensitivity and specificity of the test for *S. pneumoniae* antigen in CSF by immunochromatography ( BinaxNOW® test ) was 100 %. The VPP and VPN were also 100 %. These results are similar to those reported previously in the literature. Knowing the reliability of this fast, simple and inexpensive test, will allow to remove unnecessary isolation and to establish a more specific treatment and a better prognoses of the disease.

### A1024 Evaluation of sensitivity and specificity of different criteria using for diagnosis of burn sepsis

#### V. Panafidina^1^, I. Shlyk^1^, V. Ilyina^2^

##### ^1^Pavlov First Saint-Petersburg State Medical University, Anesthesiology and Intensive Care, Saint-Petersburg, Russian Federation; ^2^Saint-Petersburg I. I. Dzhanelidze Research institute of Emergency Medicine, Saint-Petersburg, Russian Federation

###### **Correspondence:** V. Panafidina – Pavlov First Saint-Petersburg State Medical University, Anesthesiology and Intensive Care, Saint-Petersburg, Russian Federation

**Introduction:** Difficulties in diagnosis of burn sepsis are associated with early and persistent systemic inflammatory response syndrome (SIRS) formation. Thus, different scales for diagnosis of sepsis in burn patients have been developed.

**Objectives:** Assessment of sensitivity and specificity of scales and criteria using for diagnosis of burn sepsis.ave been developed.

**Methods:** Single-center retrospective study including review of 30 patients who died in burn ICU. Criteria of including: clinical signs of sepsis (bacteremia + invasive wound infection by histology + criteria of The Third International Consensus Definitions for Sepsis and Septic Shock), age 18–65, burns > 10 % of total body surface area, death after 72 hours in ICU. Evaluation of the organ dysfunction by SOFA (Sequential Organ Failure Assessment) score, SIRS criteria, American Burn Association (ABA) sepsis criteria, French Society for Burn Injuries (FSBI) criteria of infection in burns and Chinese definitions of burn sepsis (CDBS) were performed. Patients were divided into two groups: with pathomorphological signs of sepsis (abscesses, microabscesses and/or bacterial emboli; group №1, n = 14) and without these (group №2, n = 16). Logistic regression was performed to identify the independent factors for the prediction of early death (7 days and less). We examined sensitivity and specificity with area under the Receiver Operating Characteristic Curve (ROC AUC).

**Results:** There were no significant differences between two groups for demographics, burn size, inhalation injuries. Fatal outcome came early in the group №2 (mean ICU length of stay 7,5 days vs 10 days, p < 0,05). Organ dysfunction at day 4 was significantly higher in the group №2 (mean SOFA 3,4 vs 2,0; p < 0,05). There were no significant differences in ABA, FSBI and CDBS between two groups, and the highest AUC were for CDBS by the day 6 (AUC 0,687 95 % CI 0,517-0,796 vs 0,567 95 % CI 0,469-0,719 and 0,504 95 % CI 0,315-0,702 for CDBS, ABA and FSBI, respectively). SIRS criteria were significantly higher in the group №2, AUC was 0,745 (95 % CI 0,412-0,889) but specificity and sensitivity was too low (for 2 SIRS criteria is 64,9 % and 12 %, respectively). Independent factors for early death include: more than 20 % immature neutrophils at the day 3, SOFA more than 4 by the day 4, thrombocytopenia less than 100 by the day 4.

**Conclusion:** Patients without specific pathomorphological signs of sepsis have more severe organ dysfunction, greater number signs of systemic inflammation and earlier fatal outcome. Diagnostic model of sepsis by Chinese experts has more sensitivity and specificity for diagnosis of burn sepsis confirmed by autopsy. Immature neutrophils count, thrombocytopenia and SOFA score are stronger risk factors for early death.

### A1025 The value of neutrophil to lymphocyte count ratio in diagnosing blood-stream infection

#### S. Judickas^1^, G. Kezyte^2^, I. Urbanaviciute^2^, M. Serpytis^1^, E. Gaizauskas^1^, J. Sipylaite^1^

##### ^1^Vilnius University, Clinic of Anaesthesiology and Intensive Care, Vilnius, Lithuania; ^2^Vilnius University, Faculty of Medicine, Vilnius, Lithuania

###### **Correspondence:** G. Kezyte – Vilnius University, Faculty of Medicine, Vilnius, Lithuania

**Introduction:** Blood-stream infection (BSI) is serious and life threatening condition that needs immediate recognition and management. Blood culture is the gold standard establishing the diagnosis of BSI, although it takes a lot of time and sometimes is falsely negative or positive. There is a need of alternative diagnostic methods that could lead to faster diagnosis. One of the alternative tests is neutrophil - lymphocyte count ratio (NLCR).

**Objectives:** Evaluate NLCR and define its value in association with other inflammatory markers in diagnosing BSI.

**Methods:** An exploratory cohort study conducted in University hospital from January 2015 until September 2015. Study group consisted of patients with positive blood culture. Laboratory tests of the day of blood culture were analyzed: white blood cell count (WBC), neutrophil count (NC), lymphocyte count (LC), C reactive protein (CRP), procalcitonin (PCT). NLCR was calculated by dividing neutrophils count to lymphocytes count. Laboratory tests were evaluated considering laboratory reference results. Exclusion criteria were: hematological disease and any type of immunosuppression. A control group consisted of patients with suspected BSI but negative blood cultures and no signs of sepsis in later clinical course. ROC curve analysis, negative and positive prognostic values were obtained using statistical analysis.

**Results:** Overall 320 patients were included in the study. 116 were excluded due to missing data, 102 - due to possible contamination according to laboratory information. Final analysis consisted of 103 patients': 20 in control group and 83 in study group. WBC was not significantly different between study 10.69 (1.97-35.94) and control group - 13.79 (4.7-36.83) p = 0.057. LC was significantly lower in sepsis group (0.77 (0.15-4.93) vs 1.49 (0.42-9.66), p = 0.000) while NC did not differ (10 ± 5,09 vs 10.98 ± 5.7, p = 0.456). NLCR was higher in sepsis group (15.17 ± 12.03 vs 7.39 ± 5.03, p = 0.006.). ROC analysis showed high NLCR ability to detect sepsis, compared to complete blood count cells alone - NLRC 0.762 (CI 95 % 0.622; 0.902), WBC - 0.360 (CI 95 % 0.218; 0.503), NC 0.431 (CI 95 % 0.290; 0.571), LC 0.183 (CI 95 % 0.066; 0.299). Threshold value was obtained for NLCR - 15, at this point - NLRC is 34 % sensitive and 89.5 % specific, and its negative prognostic value - 23,6 %, positive prognostic value - 93,3 %.

**Conclusions:** NLCR is elevated in blood stream infection and works better for diagnosing blood stream infections than white blood cell count alone. It is cheap and highly available assay that could be used as an additional test when blood stream infection is suspected.

### A1026 Diagnosis of infection utilizing accellix CD64

#### C.L. Sprung^1^, G. Munteanu^1^, R.C. Morales^1^, H. Kasdan^2^, T. Volker^2^, A. Reiter^2^, Y. Cohen^2^, Y. Himmel^2^, J. Meissonnier^2^

##### ^1^Hadassah Hebrew University Medical Center, Jerusalem, Israel; ^2^LeukoDx, Jerusalem, Israel

###### **Correspondence:** C.L. Sprung – Hadassah Hebrew University Medical Center, Jerusalem, Israel

**Introduction:** Differentiating infected or non- infected patients in the ICU can be very difficult. The Accellix CD64 Assay, identifies whether or not a patient is infected within 25 min. The CD64 marker measures neutrophil activation rapidly thus enabling early treatment. CD64 is constitutively expressed on the cell surface of PMNs at low levels in patients without infections. When pathogens invade the circulation, expression of CD64 on neutrophils increases dramatically at a very early step of the immune host response. The purpose of this study was to evaluate the Accellix CD64 instrument in ICU patients with and without infections.

**Methods:** Infected & non-infected ICU patients (ICU) & normal volunteers (C) had CD64 levels measured by the Accellix CD64 instrument using a sample of patient blood (30–50 μL).Measurements were calculated as CD64 index (ratio between the fluorescence of the PMN population and the fluorescence of control beads). The Accellix system is composed of two main components, a disposable cartridge and a reader. All pre-analytical and analytical processing are performed within the Accellix CD64 cartridge. The user adds a drop of blood into the sample port and places the cartridge into the reader. The cartridge contains all the needed reagents, control material as well as an integrated flow cell. Once the blood is added, cells are labeled with multiple antibodies conjugated with differentiating fluorescent tags. Once the sample processing is complete, the sample flows through a dedicated reading channel where data are acquired. Analytical data processing utilizing proprietary algorithms provide final answers on the screen. This cartridge-based platform provides rapid results, is available 24/7 and is easy to use by any clinical staff and lab technicians.

**Results:** A total of 92 subjects were studied; 54 in the ICU (ICU) and 38 controls (C). CD64 Index levels were higher (mean ± SEM) in the ICU definite infection (2.24 ± 0.48), ICU probable infection patients (1.54 ± 0.7) than ICU no infection (0.65 ± 0.17) and normal control patients (0.53 ± 0.02). Definite vs. no infection: P < 0.001, Definite vs. healthy: P < 0.0001, Probable vs. healthy: P < 0.005. CD64 Index levels were higher (mean ± SEM) in ICU definite infection plus ICU probable infection patients (1.99 ± 0.40) compared to ICU possible infections plus ICU no infection (0.64 ± 0.16), p < 0.005.

**Conclusions:** CD64 index levels are higher in infected than non-infected ICU patients. The simplicity of its handling and rapid turnaround time makes Accellix CD64 a promising assay for differentiating infected from non-infected patients in critical settings such as the emergency room and ICU.

**References**

1. Allen E, Bakke A, Purtzer M, et al. Ann Rheum Dis. 2002; Pierrakos C & Vincent JL. Critical Care 2010; Hoffmann J. Biochem Med 2011; Dimoula A. Clin Infect Dis 2014; Gibot S, Béné MC, Noel R, et al. Am J Respir Crit Care Med 2012; Gerrits, JH. Clin Chemistry & Labo Medicine 2013

### A1027 Acute bacterial meningitis in an adult population admitted in the ICU

#### M.E. Banderas-Bravo, C. Gómez-Jiménez, M.V. García-Martínez, J.F. Martínez-Carmona, J.F. Fernández-Ortega

##### Regional University Hospital in Málaga, Intensive Care Medicine, Málaga, Spain

###### **Correspondence:** C. Gómez-Jiménez – Regional University Hospital in Málaga, Intensive Care Medicine, Málaga, Spain

**Introduction:** The Acute Bacterial Meningitis (ABM) in adults admitted in ICU is an entity that keeps yet a high rate of mortality and neurological sequelae. The development of craniectomy, the increase in the use of ventricular and lumbar catheters and open head injuries have contributed to increase the number of patients with nosocomial ABM.

**Objectives:** To determine the demographic and microbiological profile of patients with ABM admitted in ICU, the mortality and the differences between communitary ABM and nosocomial ABM.

**Methods:** Retrospective study of all consecutive patients admitted in our ICU for ABM since January 2012 to February 2015. We recorded clinical and demographical data, reason for ICU admission, Glasgow score at admission, APACHE II at admission, evolution, microbiological data and antimicrobial therapy. We have performed a descriptive statistics and a comparative analysis between communitary and nosocomial ABM.

**Results:** We present a series of 23 patients, 13 of communitary ABM (56,5 %) and 10 of nosocomial (43,5 %). The mean age was 51,6 ± 18,1. 60,9 % (14) women. Alteration of cranial integrity in 11 patients. The most important reason for ICU admission was coma (74 %). Glasgow at admission was 10,2 ± 3,8. APACHE II 16,4 ± 5,9. The seizures were the main complication (28,57 %). All patients had CT at admission: 13 with hydrocephalus (56,52 %). 10 required a surgical intervention (43,5 %): 8 external ventricular drainage. Lumbar puncture was performed to 22 patients, 18 with positive culture (85,7 %). Involved microorganisms: S. Pneumoniae and N. Meningitidis in communitary ABM, S. Epidermidis and Gram-negative bacilli in nosocomial ABM. When comparing both groups, only we observed difference in the C-reactive protein at admission (98 ± 99,5 in nosocomial ABM vs 177,6 ± 71,1 in communitary, p 0,05). There were differences with other variables but were not statiscally significant. So, we observed a higher mortality in nosocomial ABM group but the difference was not statistically significant (p 0,08). Global ICU mortality was 19 % (4) and hospital mortality was 30,4 % (7).

**Conclusions:** The demographic and bacteriological profiles of patients with Acute Bacterial Meningitis have changed in the last years mainly due to the expansion of neurosurgical procedures. Still has a high morbidity and mortality.

**References**

1. Eur J Clin Microbiol Infect Dis 2009; 28:1317–25. Journal of Critical care 2014; 29:347–50. N Eng J Med 2010; 362:146.

### A1028 The detection of microbial DNA but not cultured bacteria is associated with increased mortality in patients with suspected severe sepsis - a European multi-centre observational study

#### M.J. O'Dwyer^1^, M. Starczewska^2^, M. Wilks^3^, J.-L. Vincent^4^, The Rapid Diagnosis of Infections in the Critically Ill Team

##### ^1^Queen Mary University of London, William Harvey Research Institute, London, United Kingdom; ^2^The Royal London Hospital, Adult Critical Care Unit, London, United Kingdom; ^3^Queen Mary University of London, London, United Kingdom; ^4^Erasme University Hospital, Université Libre de Bruxelles, Brussels, Belgium

###### **Correspondence:** M.J. O'Dwyer – Queen Mary University of London, William Harvey Research Institute, London, United Kingdom

**Introduction:** Sepsis is a leading cause of worldwide mortality. Blood culture results poorly discriminate the mortality risk in critically ill patients with sepsis. Here we aimed to determine whether the detection of microbial DNA in the blood stream of patients with suspected sepsis was associated with mortality.

**Methods:** We performed an analysis of data collected during the Rapid Diagnosis of Infections in the Critically Ill (RADICAL) study (1). Patients were considered eligible for this study if they developed suspected sepsis and were either in or were referred for treatment to one of nine intensive care units (ICUs) in six European countries. When initial blood cultures were taken for clinical indications an additional blood sample was obtained for a culture-independent polymerase chain reaction/electrospray ionization-mass spectrometry (PCR/ESI-MS) assay. The results of the PCR/ESI-MS test were not communicated to the treating clinicians.

**Results:** Of the 529 patients analysed in the original study outcome data, blood culture results and PCR/ESI-MS results were available for 439 patients (Table 63).

**Fig. 91 (abstract A1028).**
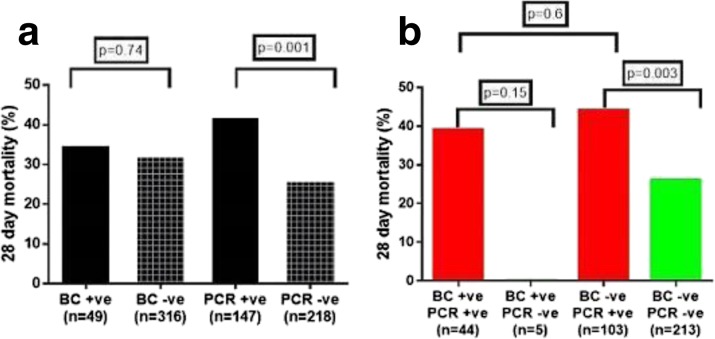
28 day mortality is not different in patients that have positive or negative blood culture results but is increased in patients that have a positive PCR/ESI-MS test result (A). Amongst patients that have a negative blood culture result those with a positive PCR/ESI-MS test result have a higher mortality

13 % (56/439) patients had a positive blood culture and 40 % (177/439) of patients had a positive PCR/ESI-MS result. Patients with either a positive blood culture (p = 0.01) or a positive PCR/ESI-MS (p = 0.005) had higher SOFA scores on study enrolment. At 28 days no mortality difference was observed in patients who had either positive or negative blood culture results (35 % versus 32 %, p = 0.74) but patients with a positive PCR/ESI-MS had a higher mortality than those with a negative result (42 % versus 26 %, p = 0.001) (Figure 91).

**Conclusions:** The detection of microbial DNA during suspected sepsis defines a patient group with a greater risk of mortality. Further work should explore the underlying disease mechanisms relating to the presence of microbial DNA.

**References**

(1) Vincent JL et al. Rapid Diagnosis of Infection in the Critically Ill, a Multicenter Study of Molecular Detection in Bloodstream Infections, Pneumonia, and Sterile Site Infections. *Crit Care Med*. 2015 Nov;43(11):2283–91

**Grant acknowledgement**

Supported, in part, by Ibis Biosciences, Abbott.

**Table 63 (abstract A1028).** Demographic and Clinical Features

	Total Cohort (n = 439)	Blood Culture + ve (n = 56)	Blood Culture -ve (n = 383)	p value	PCR/ESI-MS + ve (n = 177)	PCR/ESI-MS -ve (n = 262)	p value
Median Age (yrs) median (IQR)	65(49–75)	64(48–71)	66(50–76)	0.2	66(54–78)	64(46–72)	0.01
Sex (m%)	66 %	69 %	63 %	0.2	64 %	66 %	0.9
Antimicrobial use within 30 days preceding hospital	11 %	9 %	12 %	0.8	10 %	12 %	0.9
Antimicrobial use in hospital but preceding study enrolment	59 %	57 %	60 %	0.8	58 %	60 %	0.6
SOFA score on enrolment (median/IQR)	7(4–11)	10(6–12)	7(4–11)	0.01	8(5–11)	7(4–10)	0.005
Vasopressor use on enrolment	55 %	68 %	53 %	0.04	62 %	50 %	0.02
Requirement for mechanical ventilation	59 %	54 %	59 %	0.5	66 %	54 %	0.02
Pre-existing Diabetes	24 %	27 %	23 %	0.6	24 %	23 %	0.8
Pre-existing Cancer	29 %	30 %	29 %	0.9	34 %	26 %	0.09

### A1029 Identification of SIRS/sepsis signs by ward nurses reduces 30-days mortality in patients with sepsis

#### M. Torsvik^1^, L.T. Gustad^2,3^, I.L. Bangstad^2^, L.J. Vinje^2^, J.K. Damås^4,5,6^, E. Solligård^6,7,8^, A. Mehl^2,6,9^

##### ^1^Nord University, Faculty of Health Science, Levanger, Norway; ^2^Nord-Trøndelag Hospital Trust, Levanger Hospital, Internal Medicine, Levanger, Norway; ^3^Norwegian University of Technology and Science (NTNU), Department of Neuroscience (INM), Trondheim, Norway; ^4^St Olavs University Hospital, Department of Infectious Diseases, Trondheim, Norway; ^5^Norwegian University of technology and Science (NTNU), Centre of Molecular Inflammation Research, Department of Cancer Research and Molecular Medicine, Trondheim, Norway; ^6^Norwegian University of Technology and Science (NTNU), Mid-Norway Sepsis Research Center, Trondheim, Norway; ^7^St Olavs University Hospital, Clinic of Anesthesia and Intensive Care, Trondheim, Norway; ^8^Norwegian University of Technology and Science (NTNU), Department of Circulation and Medical Imaging, Trondheim, Norway; ^9^Norwegian University of Technology and Science (NTNU), Department of Cancer Research and Molecular Medicine,Unit for Applied Clinical Research, Trondheim, Norway

###### **Correspondence:** M. Torsvik – Nord University, Faculty of Health Science, Levanger, Norway

**Introduction:** SIRS and sepsis is easily identified with observation of vital signs and organ failure, but there is little focus on the effect of better observation and treatment for outcome in patients with suspected infection cared for at the ward level.

**Objectives:** To evaluate if a “bundle” consisting of a SIRS and organ failure (SOF)-triage, flow chart response and alert system, and a SIRS/sepsis training course for all wards nurses improved clinical observations, lead to fewer patients developing severe sepsis, decreased length of stay in the high-level care (LOS) and increased survival.

**Methods:** A before and after intervention study in one emergency and community hospital within the Mid-Norway sepsis study catchment area. All patients with confirmed blood stream infection (BSI) and evidence of sepsis have been prospectively registered continuously since 1994. The severity of sepsis, observation frequency of vital signs, treatment data, LOS and mortality were retrospectively registered from the patients' medical journals until end 2013.

**Results:** The pre-intervention group was patients with confirmed BSI from Jan 2008 to Dec 2010 (n = 472) whilst the post-interventions group was recruited between Nov 2011 to Dec 2013 (n = 409). The nurses' observation frequency of vital signs increased in BSI patients with and without severe organ failure comparing these periods. The post-intervention group had, in average, 2.4 days shorter LOS. Patients admitted without severe organ failure in the post-intervention group had a lower probability of developing severe organ failure (0.7, 95 % CI 0.4-0.9) than the pre-intervention group. Adjusted for differences in disease severity the post-intervention group also had higher odds of surviving 30 days (OR 2.7, 95 % CI 1.6-4.6).

**Conclusion:** A sepsis specific triage-, flow chart alert and treatment system was an effective tool to increase ward nurses recognition and early treatment of patients with confirmed BSI. In addition to increased survival, the shorter LOS is important from a hospital perspective in term of resource utilization.

**Grant acknowledgment**

This study was supported by the liaison committee between Nord-Trøndelag Hospital Trust and Nord University

### A1030 The diagnosis for new definition's sepsis due to gram-negative infection with endotoxin activity assay (EAA)

#### M. Tsunoda, M. Kang, M. saito, N. saito, N. Akizuki, M. Namiki, M. Takeda, J. Yuzawa, A. yaguchi, Tokyo Womens Medical University

##### Tokyo Women's Medical University, Critical Care and Emergency Medicine, Tokyo, Japan

###### **Correspondence:** M. Tsunoda – Tokyo Women's Medical University, Critical Care and Emergency Medicine, Tokyo, Japan

**Introduction:** The Endotoxin Activity Assay (EAA™; Spectral Diagnostics Inc., Toronto, Canada) is a rapid *in vitro* diagnostic test of the neutrophil's reaction to endotoxin and reflects the endotoxemia^1)^. Recently, EAA is used to confirm endotoxemia such as in the EUPHRATES (Evaluating the Use of Polymyxin B Hemoperfusion in a Randomized controlled trial of Adults Treated for Endotoxemia and Septic shock) trial study in North America^2)^. However, EAA has not been routinely used to diagnose sepsis, yet.

**Objectives:** Our hypothesis is EAA is useful to diagnosis for new definition's sepsis due to Gram-negative infection.

**Methods:** The present study is a single-center retrospective observational analysis. Of all adult patients in whom EAA was measured at our medico-surgical ICU from July 2008 to July 2013, patients with new definition's sepsis in 2016 were included in this study. New definition's sepsis is defined as life-threatening organ dysfunction caused by a dysregulated host response to infection which is identified with total SOFA score of 2 or greater^3)^. Patients were divided into two groups, 1) with Gram-negative organisms in some cultures and 2) with no Gram-negative organisms in any cultures. Age, sex, body temperature (BT), WBC, CRP, procalcitonin (PCT), SOFA score, and EAA values were compared between two groups. Values are expressed as mean ± SD. Data was analysed by chi-square test and unpaired Students t-test. P values less than 0.05 were considered significant.

**Results:** Five hundred and twenty seven patients (330 men and 197 women; mean age 66.2 ± 17.3 years) were studied. There were 1) 251 patients with Gram-negative infection and 2) 276 patients with no Gram-negative infection. EAA values and SOFA score were statistically significant differences between Gram-negative infection and no Gram-negative infection (0.40 ± 0.18 vs. 0.37 ± 0.16, p = 0.03, 8.6 ± 4.3 vs. 7.3 ± 4.0, p < 0.0005, respectively). PCT was different but did not reach to statistically differences between two groups (17.5 ± 38.3 vs. 11.8 ± 28.9, p = 0.06) and age, sex, BT, WBC were no significant differences between two groups.

**Conclusions:** EAA values could be useful for diagnosis of sepsis with new definition. Especially, EAA of 6 or greater has been meaningful value in previous studies such as

EUPHRATES. But it should be revalue the cut-off values for diagnosis of sepsis.

**References**

1) JC. Marshall,D Foster, Jean-Louis Vincent et al. JID 2004; 190: 527–534, 2) DJ. Klein, D Foster, CA Schorr et al. Trials 2014; 11: 218, 3) M Singer, CS. Deutschman, CW Seymour et al. JAMA 2016; 315: 801–810

### A1031 Thrombocytopenia, cytokines and markers of endothelial dysfunction in severe sepsis/septic shock: evaluation of a new scoring system of disease severity

#### F. Frantzeskaki^1^, P. Tsirigotis^2^, S. Chondropoulos^2^, E. Paramythiotou^1^, M. Theodorakopoulou^1^, M. Stamouli^2^, K. Gkirkas^2^, I.K. Dimopoulou^3^

##### ^1^Attikon University Hospital, 2nd Department of Critical Care, Athens, Greece; ^2^Attikon University Hospital, 2nd Department of Internal Medicine, Athens, Greece; ^3^National and Kapodistrian University of Athens, Department of Experimental Physiology, Athens, Greece

###### **Correspondence:** F. Frantzeskaki – Attikon University Hospital, 2nd Department of Critical Care, Athens, Greece

**Introduction:** Several scoring system of patients with sepsis have been developed, incorporating the number of platelets and certain cytokines level.

**Objective:** Aim of the study was to describe the incidence and prognostic role of thrombocytopenia in patients with severe sepsis/septic shock. We also investigated the role of serum cytokine levels and of markers of endothelial activation in risk stratification systems.

**Patients and methods:** Mechanically ventilated patients with severe sepsis/septic shock, treated in ICU, were included in this prospective observational study. Exclusion criteria were mechanical ventilation for more than 72 hours prior ICU admission and brain death. Clinical and laboratory data were recorded, on a daily basis. Thrombocytopenia was defined as a PLT below 150X10^3^/μl. Thrombocytopenia was considered as mild (100X10^3^/μl ≤ PLT < 150X10^3^/μl), moderate (50X10^3^/μl ≤ PLT < 100X10^3^/μl), or severe (PLT < 50X10^3^/μl) depending on PLT counts. Serum levels of IFNγ, IL-8, ICAM, VCAM, and soluble urokinase plasminogen activation receptor (SuPAR) were estimated by using Luminex xMAP technology.

**Results:** Fifty-six out of 105 (53 %) patients enrolled in the study were thrombocytopenic at the time of admission in ICU. The overall incidence of thrombocytopenia during ICU hospitalization was 75 %, while mild, moderate, and severe thrombocytopenia developed in 18 (17 %), 15 (14 %), and in 46 (44 %) respectively. Patients with severe thrombocytopenia had higher APACHE score, higher serum ICAM, IL-8 and SUPAR levels, higher incidence of bacteremia and higher probability to present with septic shock as compared with patients with normal platelet counts. Moreover, severe thrombocytopenia was associated with statistically significantly higher hospital mortality. Patients with severe thrombocytopenia showed significant higher serum ICAM (p < 0.001), IL-8 (p = 0.013), and SUPAR (p < 0.001) levels respectively, as compared to patients with normal platelets count, or patients with mild or moderate thrombocytopenia. In multivariate analysis, higher APACHE score, thrombocytopenia, and higher serum SUPAR levels were statistically significantly associated with a higher risk of ICU mortality. Enrolled patients were stratified in different groups according to their APACHE II score (APACHE II > 18), PLT counts (PLT ≤ 128.000) and serum SUPAR levels (SUPAR > 8.1). In multivariate analysis, this new scoring system remained the only and most significant factor associated with statistically significantly increased ICU mortality [OR = 8.41, (95 % CI, 4.19 to 16.9), p < 0.0001].

**Conclusion:** Severity of thrombocytopenia in severe sepsis and septic shock parallels the severity of inflammation and subsequent endothelial dysfunction and is associated with higher mortality.

**References**

1. Fjell C, Thair S, Hsu J, Walley K, Russell J, Boyd J. Cytokines and Signaling Molecules Predict Clinical Outcomes in Sepsis. PLOS ONE, November 2013 | Volume 8 | Issue 11 | e79207

### A1032 The utility of human cytokine ELISA Plate Array I (Chemiluminescence) in sepsis

#### S. Makiko, M. Tsunoda, M. Kang, J. Yuzawa, N. Akiduki, M. Namiki, M. Takeda, A. Yaguchi

##### Tokyo Women's Medical University, Tokyo, Japan

###### **Correspondence:** S. Makiko – Tokyo Women's Medical University, Tokyo, Japan

**Introduction:** The measurements of cytokines are important to assess the severity of sepsis. However, the existing measurement of cytokines take time and are costly, it is quite difficult to routinely use those data at the bedside. Human Cytokine ELISA Plate Array I (Chemiluminescence) (Signosis, Inc., CA, US) is an ELISA kit to measure 31 human cytokines with one plate simultaneously and to provide those results quickly.

**Objectives:** To evaluate 31 human cytokines' values between before and after Polimyxin B immobilized fiber column direct hemoperfusion (PMX-DHP) (Toraymixin^TM^, Toray Medical Co., Ltd., Tokyo, Japan) treatment using with Human Cytokine ELISA Plate Array I in sepsis.

**Methods:** Seven patients (4 men, 3women; mean age 64.1 ± 18.6) who were treated with PMX-DHP were included in this study. PMX-DHP was performed for 2 hours, and then the second PMX-DHP was performed 24 hours after the end of the first treatment. Thirty-one cytokines, which are TNF-α, INF-ɤ, GCSF, CMCSF, IL-1α, IL-8, IP-10, Rantes, VEGF, EFG, IL-6, Resistin, PAI-1, IL-12, IL-13, Eotaxin, PDGF, PIGF, B-NGF, SCF, MCP-1, MIP-1α,IL-2, IL-4, IL-10, FGFb, Leptin, IGF-1, TGF-β, Adiponectin, and IL-17α, were measured and compared between immediately before the first PMX-DHP therapy, before the second PMX-DHP therapy and after PMX-DHP therapy. Human Cytokine ELISA Plate Array I (Chemiluminescence) are used the plate which 31 of cytokine capture antibodies are coated on 31 wells respectively. Each cytokine value is shown as relative light units of luminescence. Values were expressed as mean ± SD. Data were analyzed by Wilcoxon signed-ranks test. A *p* < .05 was considered as statistically significant.

**Results:** All results were provided in about 5 hours after starting this assay. One measurement of all 31 cytokines costed $340.0. TNF-α and MCP-1 values were significantly decreased between immediately before and after the second PMX-DHP therapy (987.7 ± 1194.0 vs. 360.9 ± 426.8, 590.7 ± 486.8 vs. 360.6 ± 368.3, p < .05, respectively). IL-8, IL-12 and MCP-1 were also significantly decreased between before the second and after PMX-DHP therapy (668.6 ± 463.6 vs. 324.9 ± 330.7, 623.7 ± 478.5 vs. 292.3 ± 264.9, 476.1 ± 412.0 vs. 360.6 ± 368.3, p < .05, respectively). There were no statistically significant differences between before and after PMX-DHP therapies in other cytokines.

**Conclusions:** The present study has some limitations because of a retrospective analysis and numbers of patients. However, Human Cytokine ELISA Plate Array I is a fast and low-cost assay compared with the previous ELISA method. And several cytokines are evaluated with one sample at the same time. This assay could be useful especially for the clinical research because small volumes of sample allow to several cytokines' information at the bedside.

### A1033 Involvement of the anabolic and catabolic signalling pathways in critical illness-acquired myopathy varies by muscle type

#### S. Preau^1,2^, M. Ambler^1^, A. Sigurta^1^, S. Saeed^1^, M. Singer^1^

##### ^1^University College London, Faculty of Medical Sciences, London, United Kingdom; ^2^Lille Univ Hospital, Lille, France

###### **Correspondence:** S. Preau – University College London, Faculty of Medical Sciences, London, United Kingdom

**Introduction:** Critical illness-acquired myopathy in rats is characterized by homogeneous muscle atrophy (1). Conversely, histological abnormalities are heterogeneous: oxidative muscles show patchy alterations (myofascitis, necrosis), while glycolytic types demonstrate normal patterns. Akt and mTOR are key proteins of the anabolic pathway, leading to myocyte growth when activated. Conversely, AMPK and FoxO3 are key proteins of the catabolic pathway, leading to myocyte atrophy when activated. Whether anabolic or catabolic pathway activation is dependent on skeletal muscle type (i.e. oxidative and glycolytic) during critical illness is unknown.

**Objectives:** To characterize activation of the anabolic and catabolic signalling pathways in a long-term rat peritonitis model by skeletal muscle type.

**Methods:** Male Wistar rats were followed for up to 2 weeks after intraperitoneal injection of the yeast cell wall constituent, zymosan or n-saline. Soleus (oxidative, slow twitch muscle), and gastrocnemius (mixed glycolytic-oxidative, fast twitch muscle) were harvested from both zymosan and control groups at 2, 7 and 14 days after the insult. Expression of phospho- (p-) and total proteins were assessed by Western blots. Expression of Akt, p-Akt (p-threonine 308, active form), mTOR, p-mTOR (p-serine 2448, active form), AMPK, p-AMPK (p-threonine 172, active form), FoxO3, and p-FoxO3 (p-threonine 32, inactive form) were assessed at all time points.

**Results:** Weight loss was not statistically different in soleus *versus* gastrocnemius in the zymosan group (−26 ± 11 % versus −16 ± 9 %, p = 0.17) at day 2. Gastrocnemius displayed a decrease in p-Akt at day 2, and an increase of p-Akt and p-FoxO3 at day 14. Soleus displayed an increase of p-Akt, p-AMPK, and p-FoxO3 at day 2, and an increase of p-AMPK, and p-FoxO3 at day 7. Results are detailed in the Table 64.

**Conclusions:** In a rodent model of long-term peritonitis, both oxidative and glycolytic muscles display little change in the anabolic signalling pathway. AMPK (an autophagy activator) is activated while FoxO3, (an autophagy and ubiquitin-proteasome system activator) is inhibited up until day 7 in oxidative but not glycolytic muscle.

**References**

1. Hill NE et al. Detailed characterization of a long-term rodent model of critical illness and recovery. Crit Care Med. 2015 Mar;43(3):e84-96.

**Table 64 (abstract A1033).** Anabolic and catabolic signalling pathways

	soleus			gastrocnemius		
Day	2	7	14	2	7	14
AKT	<−>	<−>	+	<−>	<−>	<−>
p-AKT (active)	+	<−>	<−>	-	<−>	+
mTOR	<−>	<−>	<−>	<−>	<−>	<−>
p-mTOR (active)	<−>	<−>	<−>	<−>	<−>	<−>
AMPK	<−>	-	<−>	<−>	<−>	<−>
p-AMPK (active)	+	+	<−>	<−>	<−>	<−>
FoxO3	+	<−>	<−>	-	<−>	<−>
p-FoxO3 (inactive)	+	+	<−>	<−>	<−>	+

## MONITORING THE RESPIRATORY SYSTEM BY ESOPHAGEAL PRESSURE AND LUNG VOLUMES

### 1034 Optimization of peep for alveolar recruitment in ards based on inspiratory transpulmonary pressure

#### S. Jochmans^1,2,3^, J. Chelly^1,2^, L.V.P. Vong^1^, O. Sy^1^, J. Serbource-Goguel^1^, N. Rolin^1^, C.-M. Weyer^1^, R.I. Abdallah^1^, C. Adrie^1,2^, C. Vinsonneau^1,2^, M. Monchi^1,2^

##### ^1^Melun Hospital, Intensive Care Medicine Department, Melun, France; ^2^Melun Hospital, Clinical Research Unit, Melun, France; ^3^Plug Working Group, ESICM, Melun, France

###### **Correspondence:** S. Jochmans – Melun Hospital, Intensive Care Medicine Department, Melun, France

**Introduction:** Esophageal pressure (P_es_) guided setting of PEEP has been described in ARDS patients^1^ either to avoid expiratory alveolar collapse^2^ or to promote maximum inspiratory recruitment^3^. The proportion of ARDS patients that may benefit from maximum recruitment strategy and its effects regarding dead space (V_D_/V_T_), shunt, driving pressure (DP), transpulmonary driving pressure (TPDP) and expiratory transpulmonary pressure (TPP_exp_) remain unclear.

**Methods:** We included moderate and severe ARDS patients under mechanical ventilation and paralyzed, in the first 12 hours after reaching ARDS criteria. Patients were monitored with esophageal balloon catheter and ventilated with EXPRESS study settings for 1 hour after recruitment maneuver. Then PEEP was modified to obtain an inspiratory transpulmonary pressure (TPP_insp-P_) based on P_es_ between 20 and 25 cmH_2_O. Increase in PEEP was limited to 25 cmH_2_O or less in case of severe haemodynamic failure.

**Results:** 22 ARDS patients have been included: 16 (73) male with age 56 [44–61], SAPS2 46 [34–49] and SOFA 9 [7–12]. 19 (86) had pulmonary ARDS with PaO2/FiO2 121 [100–160] at inclusion and PEEP 12 [10–12]. 21 (95) needed vasopressors, 5 (23) renal replacement therapy, 14 (64) prone position and none ECMO or ECCO2R. ICU and hospital lengths of stay were respectively 15 [9–25] and 22 [14–37]. 8 (36) patients died. Only one patient had PEEP limitation due to patent foramen ovale.

Pes measurement distinguished 2 groups of patients (Table 65): in 11 (50) patients (Group A) inspiratory esophageal pressure (P_es-insp_) was 4 [2–7] leading to small modifications in PEEP (< ± 4 cmH_2_O). In the second half of patients P_es-insp_ was high 17 [14–23] allowing PEEP increase > 4 cmH_2_O (Group B).

Higher PEEP in Group B led to higher plateau pressure and TPP_insp-P_, positivation of TPP_exp_ without increase in V_D_/V_T_ (p = 0.97), shunt (p = 0.84), DP (p = 0.16), TPDP (p = 0.09) or oxygen stretch index (p = 1). However agreement between TPP_insp-P_ and TPP_insp_ calculated from respiratory motion equation and chest wall elastance (TPP_insp-E_) was weak with Band-Altman bias (TPP_insp-E_ - TPP_insp-P_) = 4.5 ± 8.9 [95%CI −13;22].

**Conclusions:** P_es_ measurement in moderate to severe ARDS patients distinguishes 2 groups of patients in whom PEEP appears to be taylorized without side-effects. However physiologic studies should assess reliability of transpulmonary measurement based on either P_es_ or chest wall elastance.

**References**

1. Akoumianaki, E. *et al.* The application of esophageal pressure measurement in patients with respiratory failure. *Am. J. Respir. Crit. Care Med.***189,** 520–531 (2014).

2. Talmor, D. *et al.* Mechanical ventilation guided by esophageal pressure in acute lung injury. *N. Engl. J. Med.***359,** 2095–2104 (2008).

3. Grasso, S. *et al.* ECMO criteria for influenza A (H1N1)-associated ARDS: role of transpulmonary pressure. *Intensive Care Med.***38,** 395–403 (2012).

**Table 65 (abstract A1034).** Comparative data between groups and steps

Parameters		EXPRESS Settings	P_es_Guided Settings	p-value
PEEP	Group A	15 [14–17]	14 [12–16]	0.27
	Group B	16 [15–18]	24 [23–25]	0.002
	p-value	0.36	0.001	<0.001
P_plat_	Group A	30 [30–31]	28 [26–30]	0.04
	Group B	28 [25–30]	37 [36–38]	<0.001
	p-value	0.02	<0.001	<0.001
P_es-insp_	Group A	4 [2–7]	4 [1–7]	0.97
	Group B	17 [14–23]	21 [15–25]	0.006
	p-value	<0.001	<0.001	<0.001
P_es-exp_	Group A	1 [(−1)-4]	0 [(−2)-4]	0.97
	Group B	14 [10–17]	17 [14–21]	0.15
	p-value	<0.001	<0.001	<0.001
TPP_insp-P_	Group A	26 [25–29]	24 [22–25]	0.07
	Group B	11 [6–13]	19 [9–21]	0.05
	p-value	<0.001	0.02	<0.001
TPP_insp-E_	Group A	23 [21–25]	22 [19–23]	0.26
	Group B	17 [14–22]	30 [24–32]	0.002
	p-value	0.03	0.009	0.002
TPP_exp_	Group A	16 [10–18]	14 [13–15]	0.17
	Group B	2 [(−3)-5]	6 [4–9]	0.04
	p-value	<0.001	0.004	<0.001
TPDP	Group A	11 [10–13]	10 [9–12]	0.51
	Group B	6 [5–10]	11 [8–12]	0.09
	p-value	0.02	0.74	0.09
DP	Group A	15 [13–16]	15 [12–16]	0.47
	Group B	11 [9–13]	13 [11–16]	0.16
	p-value	0.02	0.62	0.06

### A1035 Impact of high volume paracentesis on respiratory parameters including transpulmonary pressure and on haemodynamics: a prospective study

#### U. Mayr, W. Huber, E. Karsten, T. Lahmer, P. Thies, B. Henschel, G. Fischer, R.M. Schmid

##### Technische Universität München, II. Medizinische Klinik, Munich, Germany

###### **Correspondence:** U. Mayr – Technische Universität München, II. Medizinische Klinik, Munich, Germany

**Introduction:** To optimize mechanical ventilation different targets are used including tidal volume (TV), peak and mean airway pressure and PEEP. However, prevention of alveolar collapse not only depends on *intra-pulmonary*, but also on the *extra-pulmonary pressure* (EPP). EPP can be estimated by measuring esophageal pressure (EP). Ventilator strategies aiming at optimized *trans-pulmonary* pressure TPP (difference between intra- and extra-pulmonary pressure: TPP = TIP-TEP) have been shown to improve outcome. TPP-guided ventilator setting might be useful in patients with liver cirrhosis and ascites. However, the impact of paracentesis on TPP is poorly investigated.

**Objectives:** To investigate the impact of high volume paracentesis (HVP; ≥3000 mL) on TPP and on other parameters of pulmonary and circulatory function.

**Methods:** Analysis of 23 HVP-procedures in 11 patients ventilated with the AVEA Viasys ventilator (CareFusion, USA) capable to measure EP via an esophageal tube. Haemodynamic monitoring with the PiCCO-2-device (Pulsion Medical Systems SE, Feldkirchen, Germany) was available during 17 measurements. Intra-abdominal pressure IAP was determined by intra-peritoneal (IAP_P) and intra-vesical (IAP_V) pressure measurement. High grade esophageal varices had been excluded endoscopically before measurement of EP. Statistics: SPSS 23.

**Results:** 5 male, 6 female patients, aetiology of cirrhosis alcoholic (n = 7), viral (2) and cryptogenic (2). Age 51 ± 15 years, APACHE-II 30 ± 9, SOFA 13 ± 4, MELD 25 ± 10. Paracentesis of 4826 ± 1276 mL resulted in marked increases in inspiratory (17.9 ± 8.9 vs. 5.4 ± 13.4; p < 0.001) and expiratory (−3.0 ± 4.7 vs. -15.9 ± 10.9 cmH_2_O; p < 0.001) TPP. In parallel inspiratory (2.4 ± 8.7 vs. 14.1 ± 14.5 cmH_2_O; p < 0.001) and expiratory (12.4 ± 6.0 vs. 24.9x ± 11.3cmH_2_O; p < 0.001) EP significantly decreased. Paracentesis resulted in decreases in IAP_P (5.2 ± 2.3 vs. 11.7 ± 2.0 mmHg; p < 0.001), IAP_V (8.1 ± 2.3.4 vs. 16.2 ± 6.0 mmHg; p = 0.003) and CVP (15.0 ± 9.7 vs. 21.3 ± 9.3 mmHg; p < 0.001). The marked 30 % decrease in CVP is in contrasts to the unchanged values of MAP (p = 0.139), CI (p = 0.140), SVI (p = 0.635), GEDVI (771 ± 87 vs. 771 ± 104 mL/m^2^; p = 0.979), EVLWI (p = 0.639) and SVRI (p = 0.381). Among respiratory parameters we observed improved pO2/FiO2 (254 ± 54 vs. 222 ± 41 mmHg; p = 0.001), OI (5.1 ± 2.3 vs. 5.8 ± 3.1; p = 0.016), increases in TV (510 ± 100 vs. 452 ± 113 mL; p = 0.009), compliance (47 ± 16 vs. 35 ± 15 mL/cmH_2_O; p < 0.001) and a decrease in respiratory rate (17 ± 7 vs. 20 ± 8 min^−1^; p = 0.013).

**Conclusions:** Paracentesis markedly increases inspiratory and expiratory TPP in parallel with a decrease in IAP. Increased IAP before paracentesis resulted in markedly decreased inspiratory and end-expiratory TPP despite ventilation according to the ARDSnet guidelines. To avoid decreased end-expiratory TPP and alveolar collapse in patients with increased IAP, paracenteses and/or higher PEEP-setting should be used. IAP and its changes markedly confound CVP, but neither GEDVI nor CI.

**References**

**Grant acknowledgment**

### A1036 Transpulmonary pressure guided mechanical ventilation in ARDS. Effect on PEEP and PaO_2_/FiO_2_ ratio

#### O. Ediboglu, S. Ataman, I. Naz, G. Yaman, C. Kirakli

##### Dr. Suat Seren Chest Diseases and Surgery Training Hospital, Intensive Care Unit, Izmir, Turkey

###### **Correspondence:** O. Ediboglu – Dr. Suat Seren Chest Diseases and Surgery Training Hospital, Intensive Care Unit, Izmir, Turkey

**Introduction:** Determining and setting the optimal and safest PEEP levels in ARDS patients often can be challenging for the ICU physicians. Real time transpulmonary pressure (Ptp) measurement by an esophageal balloon catheter and titrating PEEP levels according to the end expiratory Ptp shows promising results in these patients (1,2).

**Objectives:** To assess the feasibility and effect of Ptp guided PEEP titration in ARDS patients.

**Methods:** An esophageal balloon catheter (Cooper Surgical Inc, Trumbull, CT, USA) was inserted to monitor the esophageal pressure in ARDS patients under invasive mechanical ventilation. Patients were connected to a ventilator capable of monitoring airway, esophageal and transpulmonary pressure waveforms real time breath-by-breath (Hamilton G5, Hamilton Medical AG, Bonaduz, Switzerland). PEEP levels were adjusted to achieve a Ptp 0 to 10 cmH_2_O at end expiration

**Results:** Six patients were enrolled. Median age was 34 (26–48) years and body mass index was 25 (20–30). Median PEEP was increased to 20 (16–24) from 9 (7–15) cmH_2_O after titrating PEEP according to the Ptp at end expiration (p = 0.03). This also led to a significant improvement in PaO_2_/FiO_2_ ratio [from 73 (64–91) to 160 (114–250), p = 0.006] and allowed a nonsignificant decrease in FiO_2_ levels [from 1 (0.8-1) to 0.55 (0.47-1)]. Median pH increased from 7.32 (7.24-7.52) to 7.44 (7.39-7.48) and PaCO_2_ decreased from 48 (36–66) to 40 (33–51) mmHg but these changes did not reach statistical significance.

**Conclusions:** Ptp guided PEEP titration can be beneficial especially in ARDS patients who are in need of high PEEP levels.

**References**

1. Talmor D, Sarge T, Malhotra A, O´Donnell CR, Ritz R, Lisbon A, Novack V, Loring SH. Mechanical ventilation guided by esophageal pressure in acute lung injury. N Engl J Med. 2008 Nov 13; 359(20):2095–104. doi: 10.1056/NEJMoa0708638. Epub 2008 Nov 11. PubMed PMID: 19001507; PubMed Central PMCID: PMC3969885.

2. Wu X, Zheng R, Lin H, Zhuang Z, Zhang M, Yan P. [Effect of transpulmonary pressure-directed mechanical ventilation on respiration in severe acute pancreatitis patient with intraabdominal hypertension]. Zhonghua Yi Xue Za Zhi. 2015 Oct 20; 95(39):3168–72. Chinese. PubMed PMID: 26814111.

**Fig. 92 (abstract A1036).**
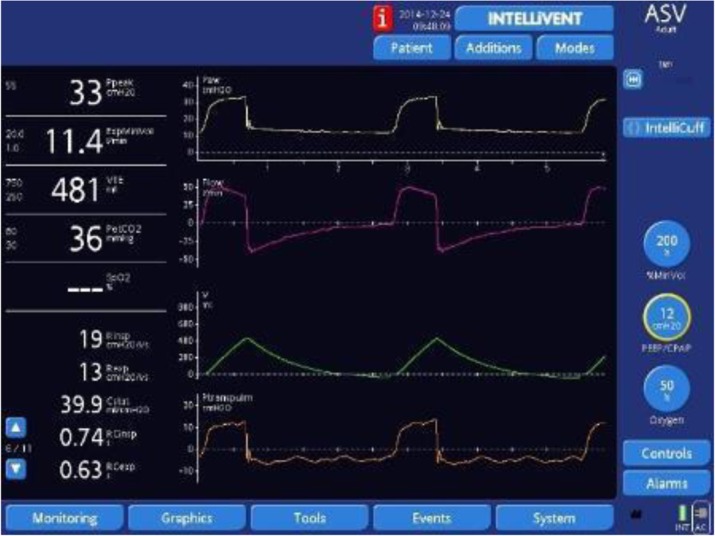
ᅟ

**Fig. 93 (abstract A1036).**
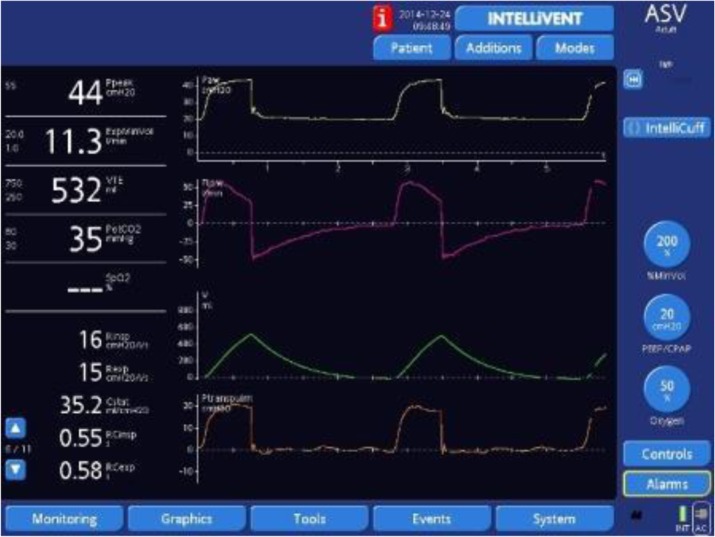
ᅟ

### A1037 A comparative study between muscular pressure-time product estimated from △P (P_AW, PEAK_ - PEEP) and esophageal pressure tracing during proportional assist ventilation

#### P.-L. Su^1^, P.-S. Kou^1^, W.-C. Lin^1^, C.-W. Chen^1,2^

##### ^1^National Cheng Kung University Hospital, Department of Internal Medicine, Tainan, Taiwan, Province of China; ^2^National Cheng Kung University, Medical Device Innovation Center, Tainan, Taiwan, Province of China

###### **Correspondence:** C.-W. Chen – National Cheng Kung University Hospital, Department of Internal Medicine, Tainan, Taiwan, Province of China

**Introduction:** If the proportional assist ventilation(PAV) level is known, then muscular effort can be estimated from the difference between peak airway pressure and PEEP (△P) during PAV. Namely, P_mus, peak, aw_ = (P_aw, peak_ - PEEP) X (100 - Gain)/Gain. Pressure time product estimated from airway (PTP_aw_) = P_mus, peak, aw_ X inspiratory time/2 X respiratory rate[1].

**Objectives:** Validation of this hypothesis by using the esophageal pressure time product calculation.

**Methods:** Eleven mechanically ventilated patients who received esophageal pressure monitoring under PAV were enrolled. Patients were randomly assigned to seven PAV assist levels (20-80 %, PAV20 means 20 % PAV Gain) for 15 minutes. Maximal muscular pressure (P_mus, peak, es_ and P_mus, peak, aw_) and pressure time product (PTP_es_ and PTP_aw_) estimated from △P and esophageal pressure were determined from the last minute of each PAV level.

**Results:** PAV significantly reduced the breathing efforts of patients with increasing PAV gain (PTP_es_ 197.7 ± 75.8 at PAV20 vs. 71.2 ± 47.0 cmH_2_O∙sec/min at PAV80, PTP_es, PEEPi_ 262.7 ± 95.6 at PAV20 vs. 101.2 ± 66.4 cmH_2_O∙sec/min at PAV80, p < 0.0001). P_mus, peak, aw_ overestimate P_mus, peak, es_ in PAV of low gain (PAV20) and underestimate in PAV of moderate to high gain (from PAV40 to PAV80). Linear regression analysis revealed that the slope PTP_es, br_(PTP_es_ per breath)/P_mus, peak,es_ for PTP_es, PEEPi_ is 0.84 (r^2^ = 0.8939), for PTP_es_ is 0.70 (r^2^ = 0.8760), and PTP_aw, br_ (PTP_aw_ per breath)/P_mus, peak, aw_ for PTP_aw_ is 0.56 (r^2^ = 0.9351).

**Conclusions:** Adjustments should be made when extrapolating PTP_aw_ into PTP_es_. An additional 25 % should be added when extrapolating PTP_es_ from P_mus, peak, aw_ and an additional 50 % should be added when extrapolating PTP_es, PEEPi_ from P_mus, peak, aw_, assuming P_mus, peak, es_ and P_mus, peak, aw_ are equal.

**References**

1. Carteaux G, Mancebo J, Mercat A, Dellamonica J, Richard JC, Aguirre-Bermeo H, et al. Bedside adjustment of proportional assist ventilation to target a predefined range of respiratory effort. Crit Care Med. 2013;41(9):2125–32.

**Grant acknowledgment**

National Cheng-Kung University Hospital Grant

### A1038 Assessment effort and work of breathing by airway occlusion pressure versus esophageal pressure

#### J.A. Benítez Lozano^1^, P. Carmona Sánchez^2^, J.E. Barrueco Francioni^3^, F. Ruiz Ferrón^4^, J.M. Serrano Simón^2^

##### ^1^Hospital Quirón de Málaga, Intensive Care Unit, Málaga, Spain; ^2^Hospital Universitario Reina Sofia, Intensive Care Unit, Córdoba, Spain; ^3^Hospital of Manises, Intensive Care Unit, Valencia, Spain, ^4^Complejo Hospitalario de Jaén, Intensive Care Unit, Jaén, Spain

###### **Correspondence:** J.M. Serrano Simón – Hospital Universitario Reina Sofia, Intensive Care Unit, Córdoba, Spain

**Introduction:** Airway occlusion pressure is a noninvasive measure of motor neural output. If the airway is occluded, the change in pressure in the pleural space and at the airway open, both are equivalent

**Objectives:** We studied the similarity of effort and work of breathing measure with Pesophageal (Peso) at regular cycles, versus inadvertent airway pressure (Paw) occluded during end-expiration (Paw_occl).

**Fig. 94 (abstract A1038).**
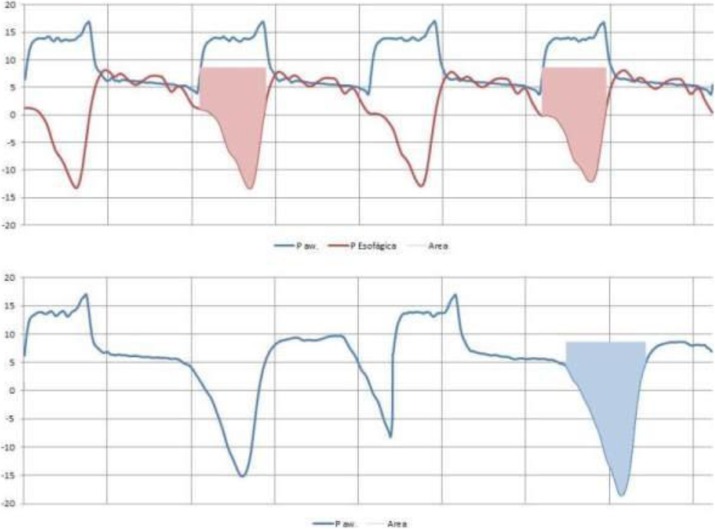
ᅟ

**Fig. 95 (abstract A1038).**
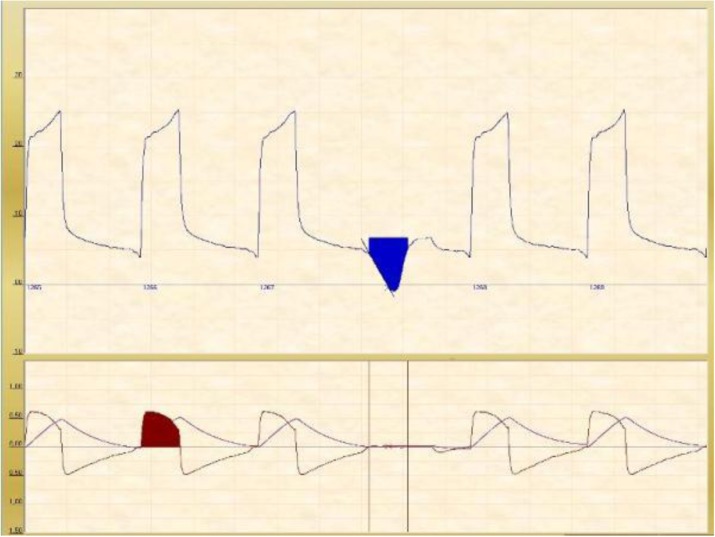
ᅟ

**Methods:** Esophageal, airway pressure and airway flow, sampling 278 Hz, were registered in 10 patients during weaning time, with levels of *sedation Ramsay 2, at* Pressure support ventilation (PSV) with differents levels of assistance (High:15–23 cmH2O, medium: 10–14 cmH2O, low: 5–9 cmH2O). Respiratory effort was quantified using pressure-time product (PTP/min) with esophageal and occluded cycle (Figure 94), and WOB_Occlusion, using the occlusion pressure with the flow of preceding, not occluded, cycles (Figure 95). The work of breathing esophageal referent (WOB_Peso, j/l)) is obtained from integral of Peso versus differential of volume. Also we are calculated Δ Peso and Δ Paw_occl as additional parameter. For all data, the Bland-Altman analysis and linear regression was applied. The results are expressed as mean ± SD, the comparison was made by t-test.

**Results:** A total of 69 paired measures were obtained. The mean comparison of the respiratory effort and work did not show showed statistical differences for all data, except for low assistance (Table 66). A good correlation between both measures methods was observed for PTP and WOB (R = 0,91 and 0,87; respectively). The mean bias was for PTP and WOB: 42,64 (±58,95) cmH2O*sec/min and 0,07 (±0,29) j/l, respectively; and the 95 % limits of agreement were −72,91 to 158,19cmH2O*sec/min and −0,50 to 0,64 j/L, respectively; this indicates wide dispersion.

**Conclusions:** Airway occlusion pressure is a noninvasive procedure that could be useful to assess the effort and work of breathing patients during mechanical ventilation.

**References**

1. W. A. Whitelaw and J.P. Derenne. Airway occlusion pressure. J. Appl. Physiol. 74(4): 1475–1483, 1993.

**Table 66 (abstract 1038).** ᅟ

Cases (N)	PTP_Peso cmH2O*sec/min	PTP_occl cmH2O*sec/min	WOB_Peso j/L	WOB_occl j/L	Δ Peso cmH2O	Δ Paw_occl cmH2O
All Data (69)	233,83 ± 128,45	276,47 ± 138,81	1,01 ± 0,52	1,12 ± 0,55	15,2 ± 8,05	17,81 ± 8,64
	P = 0,063		P = 0,17		P = 0,07	
High PSV (27)	165,02 ± 91,41	180,12 ± 79,16	079 ± 0,39	0,92 ± 0,41	13,07 ± 6,05	16,16 ± 5,85
	P = 0,53		P = 0,26		P = 0,047	
Medium PSV (21)	269,39 ± 151,08	316,510 ± 158,65	0,33 ± 0,15	1,31 ± 0,68	15,46 ± 10,03	18,86 ± 10,43
	P = 0,33		P = 0,53		P = 0,222	
Low PSV (21)	280,00 ± 113,34	350,87 ± 111,50	1,33 ± 0,52	1,25 ± 0,67	19,01 ± 7,03	19,71 ± 8,56
	P = 0,048		P = 0,662		P = 0,747

### A1039 Monitoring of changes in lung and chest wall mechanics in the supine, lateral and prone position during the prone positioning maneuver in ARDS patients

#### Z. Riad^1^, M. Mezidi^1^, M. Aublanc^1^, S. Perinel^1^, F. Lissonde^1^, A. Louf-Durier^1^, H. Yonis^1^, R. Tapponnier^1^, J.-C. Richard^1^, B. Louis^2^, C. Guérin^1,2^, PLUG working group

##### ^1^Réanimation Médicale, Hôpital de la Croix-Rousse, Lyon, France; ^2^IMRB, INSERM 955, Créteil, France

###### **Correspondence:** Z. Riad – Réanimation Médicale, Hôpital de la Croix-Rousse, Lyon, France

**Introduction:** Chest wall elastance (Ecw) is thought to increase in prone (P) as compared to supine (S) position in ARDS patients (1–3). This makes respiratory system elastance (Ers) not reflecting lung elastance (E_L)_. Little is known about the changes of Ecw, E_L_ and lung resistance (R_L_) when moving the patient from the supine to the prone position via the lateral (L) position (4–5).

**Objectives:** The goal of present study was to measure Ecw, E_L_ and R_L_ in ARDS patients in S, L and P position during the proning procedure.

**Methods:** ARDS patients intubated, sedated and paralyzed with an indication of P positioning were included. Mechanical ventilation was delivered in volume controlled mode with constant flow inflation. End-inspiratory pause of 0.100 sec was set during the breathing cycles. Ventilator settings were unaltered during the procedure. Airway and esophageal pressures and airflow were continuously measured during 30 minutes in S, then during 1 minute in L and 30 minutes in P. The side for the lateralization was that selected by routine practice (in the opposite side from central venous line). Prone positioning was performed manually by 3 caregivers. Ecw, E_L_ and R_L_ were obtained by fitting the first order equation model to flow and pressures signals. Values are expressed as mean ± SD.

**Results:** Fifteen patients (8 males) of 66 ± 12 years, SAPS 2 41 ± 12 and SOFA 7 ± 2 were included 2 ± 3 days after ARDS criteria (12 moderate and 3 severe) were met. Tidal volume averaged 6 ± 0.6 ml/kg predicted body weight, PEEP 11 ± 3 cmH2O, FiO2 73 ± 15 %, PaO2/FiO2 ratio 116 ± 19 mmHg. The cause of ARDS was pneumonia in 9 cases, undetermined in 6 cases. Side positioning was the right in 8 and the left in 7 patients. The results are shown in the Table 67.

**Conclusions:** During prone positioning in ARDS patients, as compared to S we observed an increased R_L_ and E_L_ in L and increased Ecw in P.

**References**

(1) Pelosi P, Tubiolo D, Mascheroni D, Vicardi P, Crotti S, Valenza F, et al. Effects of the prone position on respiratory mechanics and gas exchange during acute lung injury. Am J Respir Crit Care Med. 1998;157(2):387–93.

(2) Guerin C, Badet M, Rosselli S, Heyer L, Sab JM, Langevin B, et al. Effects of prone position on alveolar recruitment and oxygenation in acute lung injury. Intensive Care Med. 1999;25(11):1222–30.

(3) Mentzelopoulos SD, Roussos C, Zakynthinos SG. Prone position reduces lung stress and strain in severe acute respiratory distress syndrome. Eur Respir J. 2005;25(3):534–44.

(4) Thomas PJ, Paratz JD, Lipman J, Stanton WR. Lateral positioning of ventilated intensive care patients: A study of oxygenation, respiratory mechanics, hemodynamics, and adverse events. Hear Lung J Acute Crit Care. 2007;36(4):277–86.

(5) Tongyoo S. The Effect of Lateral Position on Oxygenation in ARDS Patients : A Pilot Study. J Med Assoc Thai. 2006;89(6):55–61.

**Table 67 (abstract A1039).** Respiratory mechanics during the proning procedure

	Supine	Lateral	Prone
Ecw (cmH2O/L)	8.9 ± 4.5	11.2 ± 7.4	10.4 ± 5.4*
EL (cmH2O/L)	36.5 ± 17.2	42.6 ± 24.1*	38.3 ± 19.4
RL (cmH2O/L/s)	14.2 ± 4.2	15.8 ± 5.5**	15.0 ± 3.4

*P < 0.05 versus supine

**P < 0.01 versus supine

### A1040 Effect of end-inspiratory plateau pressure duration on driving pressure

#### M. Mezidi^1^, H. Yonis^1^, M. Aublanc^1^, F. Lissonde^1^, A. Louf-Durier^1^, S. Perinel^1^, R. Tapponnier^1^, J.-C. Richard^1^, C. Guérin^1,2^

##### ^1^Hôpital de la Croix-Rousse, Réanimation Médicale, Lyon, France; ^2^INSERM 955, Equipe 13, Créteil, France

###### **Correspondence:** M. Mezidi – Hôpital de la Croix-Rousse, Réanimation Médicale, Lyon, France

**Introduction:** Driving pressure of respiratory system (ΔP) is defined as the difference between plateau pressure (Pplat) and total positive end-expiratory pressure (PEEPtot) measured after end-inspiratory and end-expiratory occlusion, respectively, on airway pressure signal. ΔP has recently been shown as a strong predictor of mortality in patients with acute respiratory distress syndrome (ARDS) (1). Most of the studies involved in this demonstration measured Pplat 0.5 sec after onset of end-inspiratory pause according to the ARMA trial (2) and used PEEP set on the ventilator (PEEPvent) instead of the PEEPtot. This does not take into account slow decay of airway pressure after end-inspiratory occlusion and instrinsic PEEP, respectively.

**Objectives:** The aim of the study was to compare ΔP when Pplat was measured at different times after end-inspiratory pause and whether PEEPtot or PEEPvent were used. Our hypothesis was that ΔP was higher with Pplat measured at 0.5 and PEEPvent than with any other combinations of Pplat and PEEPtot.

**Methods:** A retrospective analysis of patients with ARDS in whom respiratory mechanics was measured. Most of the patients had recordings at two levels of PEEPvent. Data were analyzed with Acqknowledge software. Pplat was measured at 0.5, 1 and 2 seconds after end-inspiratory pause. PEEPtot was measured after a 3-sec end-expiratory pause. The low-PEEP and high-PEEP measures pertained to PEEPvent ≤10cmH20 and PEEPvent > 10 cmH20, respectively. The primary outcome was the comparison of ΔP calculated as Pplat 0.5 sec - PEEPvent : ΔP_reference_ versus ΔP computed as Pplat 2 sec - PEEPtot: ΔP_physiologic_.

The values are expressed as mean ± SD and are compared by using signed rank test for paired values, ANOVA for repeated measures and Bland-Altman.

**Results:** Twenty-three patients were analyzed. ΔP_reference_ was significatively higher than ΔP_physiologic_ in low and high PEEP groups: 13.9(±5.6) vs 10.1(±3.5) cmH2O (p < 10^−5^) and 13.5(±4.2) vs 11(±3.3) (p < 10^−4^), respectively.

**Fig. 96 (abstract A1040).**
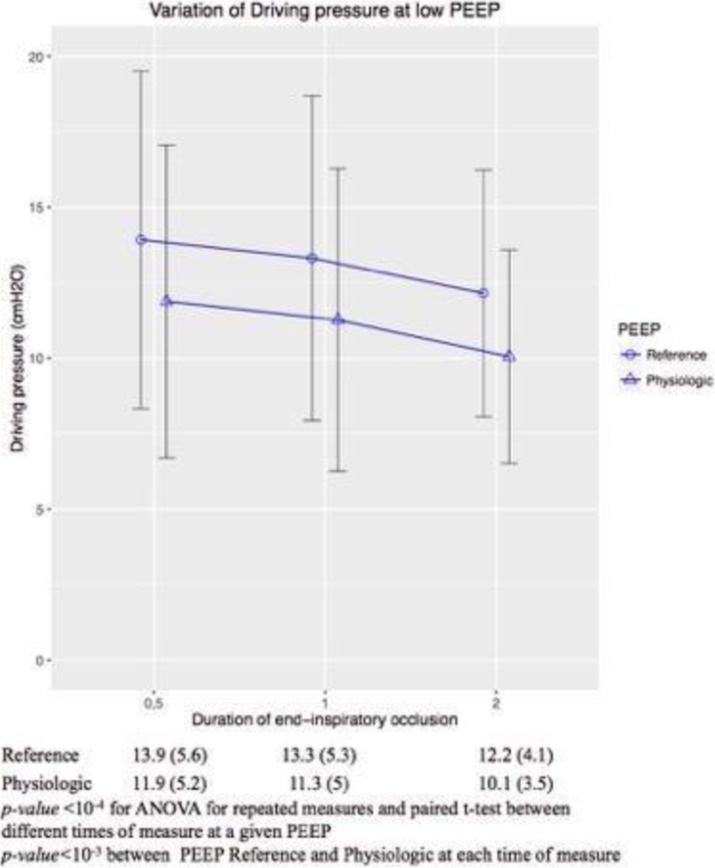
Variation of Driving presure at low PEEP

**Fig. 97 (abstract A1040).**
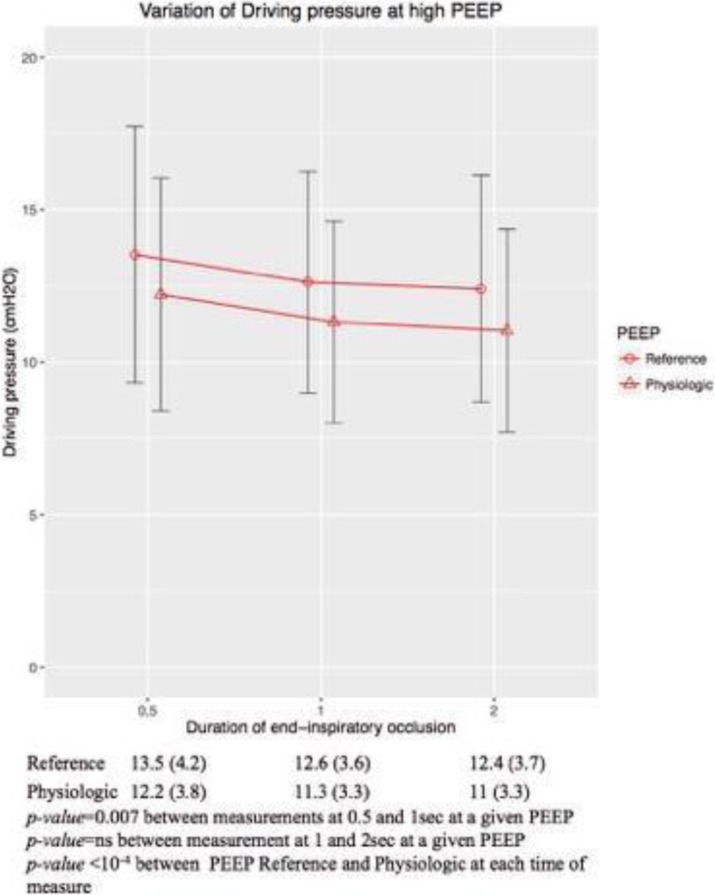
Variation of Driving presure at high PEEP

At low PEEP, ΔP_reference_ and ΔP_physiologic_ values significantly decreased with the length of the end-inspiratory occlusion, whether PEEPvent or PEEPtot were used (Figs. 96 and 97).

We compared ΔP_reference_ and ΔP_physiologic_ through a Bland Altman analysis : at low PEEP the mean difference was 3.3cmH2O (±2.6) IC95% [−1.7 — 8.5], while at high PEEP the mean difference was 2.8cmH20 (±1.9) IC95% [−0.9 — 6.5].

**Conclusions:** ΔP values are significantly altered by the way Pplat and PEEP are measured.

**References**

1. Amato MBP, Meade MO, Slutsky AS, Brochard L, Costa ELV, Schoenfeld DA, et al. Driving Pressure and Survival in the Acute Respiratory Distress Syndrome. http://dx.doi.org/101056/NEJMsa1410639. Massachusetts Medical Society; 2015 Feb 18;372(8):747–55.

2. Ventilation with lower tidal volumes as compared with traditional tidal volumes for acute lung injury and the acute respiratory distress syndrome. The Acute Respiratory Distress Syndrome Network. 2000 May 4;342(18):1301–8.

### A1041 Measuring end expiratory lung volume with two different techniques in mechanically ventilated patients. Technical problems and pitfalls

#### K. Marmanidou^1^, M. Oikonomou^1^, C. Nouris^1^, C. Loizou^2^, E. Soilemezi^1^, D. Matamis^1^

##### ^1^Papageorgiou General Hospital, Intensive Care Unit, Thessaloniki, Greece; ^2^Cyprus University of Technology, Department of Electrical Engineering, Computer Engineering and Informatics, Limassol, Cyprus

###### **Correspondence:** K. Marmanidou – Papageorgiou General Hospital, Intensive Care Unit, Thessaloniki, Greece

**Introduction:** End Expiratory Lung Volume (EELV) is reduced in ventilated patients especially in patients with acute respiratory failure (ARF). Recruitment maneuvers and different levels of PEEP are used to restore EELV and to improve oxygenation. However; no matter how useful is the value of EELV in clinical practice his bedside measurement at baseline, at different levels of PEEP and after recruitment maneuvers is cumbersome. We measured the EELV with two techniques and we noted the technical problems and pitfalls.

**Methods:** We measure the EELV in patients with ARF at two levels of PEEP (8 and 16 cm H2O) with two techniques. With a dilutional Nitrogen wash in and wash out method using the Carescape R860 ventilator (GE)

and by measuring the expired lung volume after a sudden release of PEEP to zero PEEP (ZEEP). Specifically after 10 min of stabilization at each level of PEEP we performed an expiratory hold and we decrease the ventilator frequency to zero to obtain sufficient time for a complete expiration.

Therefore, by releasing the expiratory hold we permit the return of the lung to his passive lung volume at ZEEP(passive FRC).The expired volume was calculated by the integration of the expiratory flow. We call this volume ΔEELV. Correlation and Agreement between the two methods with the Bland and Altmann analysis were performed.

**Results:** 22 mechanically ventilated patients were studied. At ZEEP EELV was low (1276 ml) 43 % of the predicted. Good correlation was found between the two methods (1637 ± 556 and 1816 ± 521 ml respectively) when EELV was measured at 8 cmH2O of PEEP(R = O.92, p < 0.0001). On the contrary at 16 cmH2O of PEEP a wider variability and less agreement was noticed between the EELV values(2040 ± 703 vs. 2411 ± 597, R = 0.86, p < 0.001). Technical problems with Carescape were spontaneous breathing attempts or asynchrony in the patient-ventilator interactions leading to instable VO2-VCO2 measurements. On the contrary with the release of PEEP method high expired volumes from PEEP to ZEEP induced high expired flow exceeding the 1lit/sec, thus affecting the linear sensitivity of the ventilator's pneumotachograph.

**Conclusions:** The measurement of the EELV remains a precious parameter for the ventilatory management of the ICU patients but his measurement is still far away to be accurate by both techniques at bedside.

**Fig. 98 (abstract A1041).**
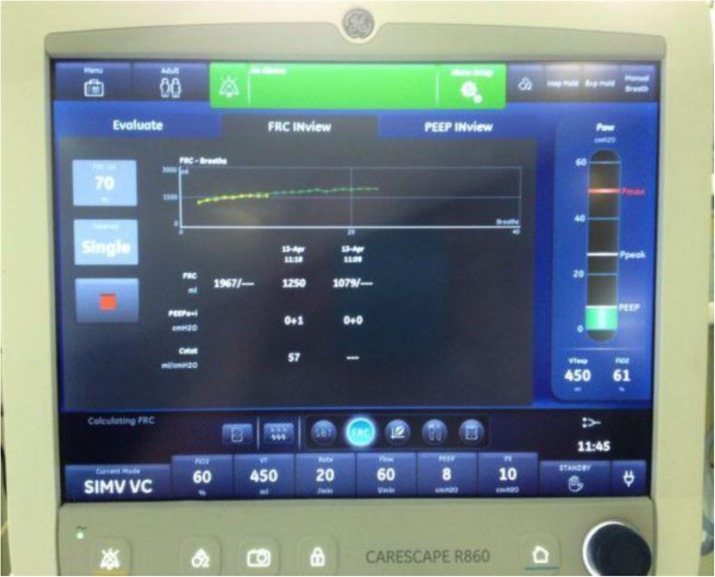
EELV measured with a Nitrogen indirect dilution

**Fig. 99 (abstract A1041).**
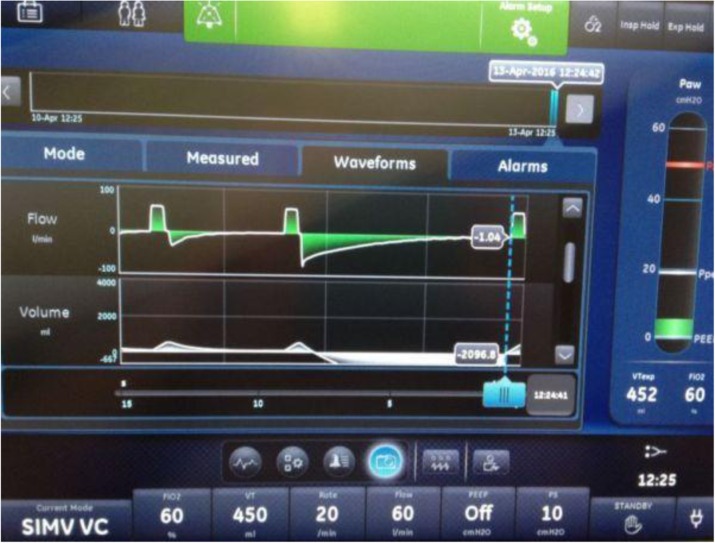
The expired volume at a complete expiration

**Fig. 100 (abstract A1041).**
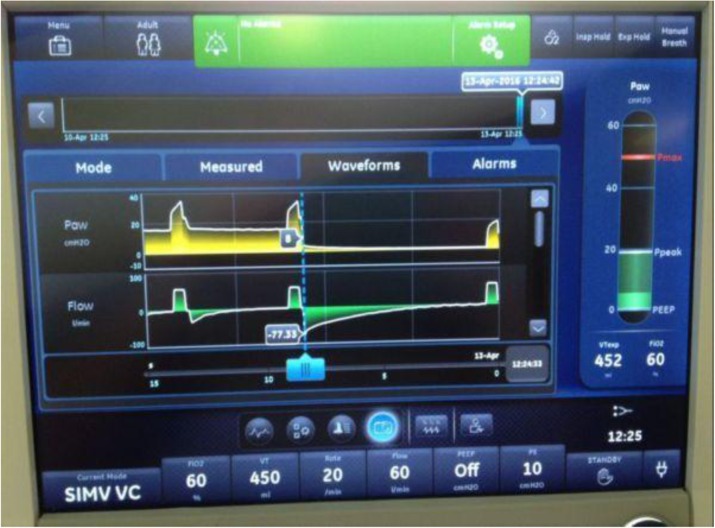
High expired flow exceeding the 1lit/sec

### A1042 Low end-expiratory trans-pulmonary pressure is associated with lung collapse

#### P. Somhorst, D. Gommers

##### Erasmus Medical Center, Intensive Care Adults, Rotterdam, Netherlands

###### **Correspondence:** P. Somhorst – Erasmus Medical Center, Intensive Care Adults, Rotterdam, Netherlands

**Introduction:** The open lung concept aims to reduce lung injury due to cyclic opening and closing of alveoli.^1^ Finding the 'optimal' PEEP to maintain an open lung proofs difficult and patient-specific.^2^ We hypothesize that targeting positive trans-pulmonary pressure (Ptp) at end of expiration (PtpEE) may prevent collapse. We used Electrical Impedance Tomography (EIT) clinically to optimize PEEP, visualizing over-distention and collapse.^3^

**Objectives:** To show the association between collapse and low PtpEE, as visualized by EIT.

**Methods:** We retrospectively analyzed data of ten patients with acute respiratory distress syndrome (ARDS) who underwent measurement of the Ptp and EIT due to clinical considerations. A PEEP trial was performed to identify optimal ventilator settings. Esophageal pressure (Pes) was measured using endo-esophageal pressure balloons (Cooper Surgical, Germany, or Sidam, Italy). The Ptp was calculated as the continuous difference between the airway pressure and Pes. EIT was measured at the 5th/6th intercostal space (Dräger, Germany) and analyzed using specialized software (Dräger, Germany). Collapse is defined as a local decrease in ventilation after a reduction in PEEP; over-distention is a local decrease in ventilation after an increase in PEEP.^4^

**Fig. 101 (abstract A1042).**
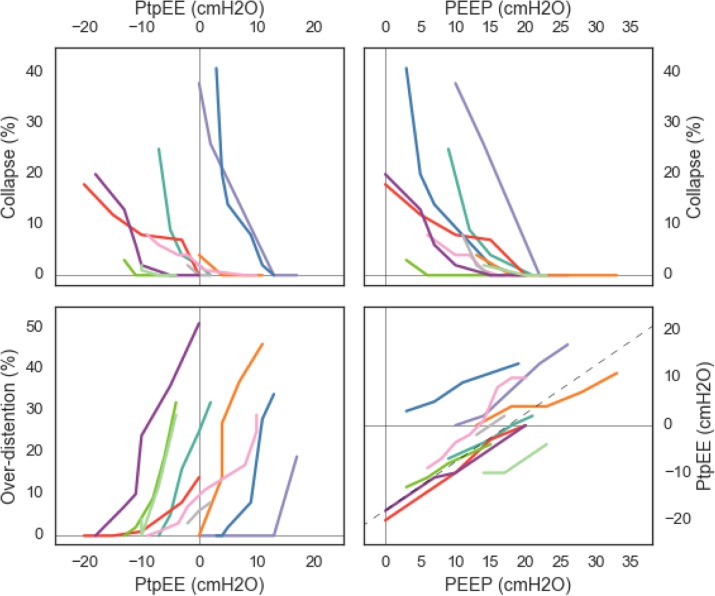
Collapse and over-distention vs. PtpEE and PEEP

**Results:** Collapse was associated with a lower PtpEE at lower PEEP levels. For most patients (8/10), collapse occurred when PtpEE was ≤ 2 cmH2O. Collapse was also present at a PEEP level of 15 cmH2O. Inversely, we showed that collapse and over-distention can occur simultaneously (Fig. 101). The PtpEE was strongly correlated to the PEEP level (r2 = 0.93, p < 0.001; corrected for each individual patient). The overall regression is shown as a dashed line.

**Conclusions:** For most ARDS patients, collapse did not occur when PtpEE was above +2 cmH2O. In addition, PEEP increase in order to prevent collapse may induce over-distention due to heterogeneity in the ventilation distribution in each patient.

**References**

1. Papadakos, P. J. & Lachmann, B. The open lung concept of mechanical ventilation: the role of recruitment and stabilization. Crit. Care Clin. 23, 241–50, ix-x (2007).

2. Gattinoni, L., Carlesso, E. & Cressoni, M. Selecting the 'right' positive end-expiratory pressure level. Curr Opin Crit Care 21, 50–57 (2015).

3. Blankman, P., Hasan, D., Erik, G. & Gommers, D. Detection of 'best' positive end-expiratory pressure derived from electrical impedance tomography parameters during a decremental positive end-expiratory pressure trial. Crit. Care 18, R95 (2014).

4. Costa, E. L. V et al. Bedside estimation of recruitable alveolar collapse and hyperdistension by electrical impedance tomography. Intensive Care Med 35, 1132–1137 (2009).

### A1043 Long forced inspiratory time is associated with high transpulmonary pressure in patients with acute respiratory failure

#### K. Hayashi^1^, T. Hirayama^1^, T. Yumoto^2^, K. Tsukahara^2^, A. Iida^2^, N. Nosaka^2^, K. Sato^2^, T. Ugawa^2^, A. Nakao^2^, Y. Ujike^3^, S. Hirohata^4^

##### ^1^Okayama University Hospital, Department of Clinical Engineering, Okayama City, Japan; ^2^Okayama University Hospital, Advanced Emergency and Critical Care Center, Okayama City, Japan; ^3^Kawasaki Medical School, Department of Acute Care & Primary Care Medicine, Kurashiki City, Japan;^4^Okayama University, Department of Medical Technology Graduate School of Health Sciences, Okayama City, Japan

###### **Correspondence:** K. Hayashi – Okayama University Hospital, Department of Clinical Engineering, Okayama City, Japan

**Introduction:** Medical experts recommend keeping plateau pressure below 30 cm H_2_O to avoid ventilator-induced lung injury in patients with acute respiratory distress syndrome (ARDS). Transpulmonary pressure (Ptp), the difference between alveolar and pleural pressure, has been measured as a surrogate for plateau pressure for lung protective strategies. However, placement of an esophageal balloon catheter is required to measure esophageal pressure.

**Objectives:** We investigated the relationship between Ptp and ventilator waveform parameters according to the strength of spontaneous breathing effort.

**Methods:** Eight patients (four patients with ARDS and four with non-ARDS) mechanically ventilated with AVEA® were included in this study. An esophageal balloon catheter (AVEA® SmartCath® esophageal balloon) was placed to measure esophageal pressure. We evaluated the relationship between ΔPtp (difference between inspiratory and end expiratory Ptp) and peak inspiratory flow, or Ti ratio (percentage of time until peak negative esophageal pressure to total inspiratory time). Spontaneous breathing effort was categorized as strong or weak and was analyzed with inspiratory waveform using VOXR data management software.

**Results:** Although there was no significant relationship between ΔPtp and peak inspiratory flow (R = 0.450, P = 0.080), a significant correlation was found between ΔPtp and Ti ratio (R = 0.720, P = 0.002). Median ΔPtp and Ti ratio were significantly higher in patients with strong spontaneous breathing effort compared with those with weak breathing effort (24.3 vs. 13.4 cmH2O, P = 0.012, and 0.50 vs. 0.21, P = 0.008, respectively). In patients with strong spontaneous breathing effort, median ΔPtp was higher in the ARDS group compared with the non-ARDS group (15.3 vs. 3.5 cmH2O, P = 0.029).

**Conclusions:** Measuring Ti ratio and ventilator waveform parameters may be helpful to estimate Ptp. Appropriate sedatives, analgesics, or muscle relaxants may be required to limit Ptp in cases of higher Ti ratio in patients with ARDS.

**References**

1) Talmor D, Sarge T, O´Donnell CR, Ritz R, Malhotra A, Lisbon A, Loring SH. Esophageal and transpulmonary pressures in acute respiratory failure. Crit Care Med. 2006;34:1389–94.

2) Yoshida T, Uchiyama A, Matsuura N, Mashimo T, Fujino Y. Spontaneous breathing during lung-protective ventilation in an experimental acute lung injury model: high transpulmonary pressure associated with strong spontaneous breathing effort may worsen lung injury. Crit Care Med. 2012;40:1578–85

### A1044 In vivo calibration of the esophageal balloon catheter: a simplified procedure

#### F. Mojoli^1^, F. Torriglia^1^, M. Giannantonio^2^, A. Orlando^1^, S. Bianzina^1^, G. Tavazzi^1^, S. Mongodi^1^, M. Pozzi^1^, G.A. Iotti^1^, A. Braschi^1^, PLUG Working Group

##### ^1^Fondazione IRCCS Policlinico S. Matteo, Anesthesia and Intensive Care, University of Pavia, Pavie, Italy; ^2^University of Chieti, School of residency in Anesthesia, Intensive Care and Pain Therapy, Chieti, Italy

###### **Correspondence:** F. Mojoli – Fondazione IRCCS Policlinico S. Matteo, Anesthesia and Intensive Care, University of Pavia, Pavie, Italy

**Introduction:** A calibration procedure has been recently proposed to obtain reliable esophageal pressure (Pes) measurements in mechanically ventilated patients [1]. This procedure helps optimizing esophageal balloon filling and removing esophageal artifacts, but is time-consuming.

**Objectives:** To test accuracy of a simplified procedure, designed according to average values of esophageal elastance (Ees) and minimum appropriate filling volume (Vmin) previously observed [1].

**Methods:** In 15 patients under Pressure Controlled Ventilation, 35 pairs of end-expiratory and end-inspiratory calibrated Pes values (Pes,cal) were obtained with the standard procedure, consisting in measure of Ees and detection of Vmin and Vbest (filling volume associated with the largest tidal swings of Pes): Pes,cal = Pes - Ees * (Vbest - Vmin). “Simplified” calibrated Pes values (S-Pes,cal) were also obtained with a simplified procedure based on detection of Vbest and on the assumptions that Ees = 1 cmH_2_O/ml and Vmin = 1 ml: S-Pes,cal = Pes - (Vbest - 1). We used the Nutrivent catheter (Sidam, Italy), equipped with an esophageal balloon that is 10 cm long and has a 10 ml nominal volume.

**Fig. 102 (abstract A1044).**
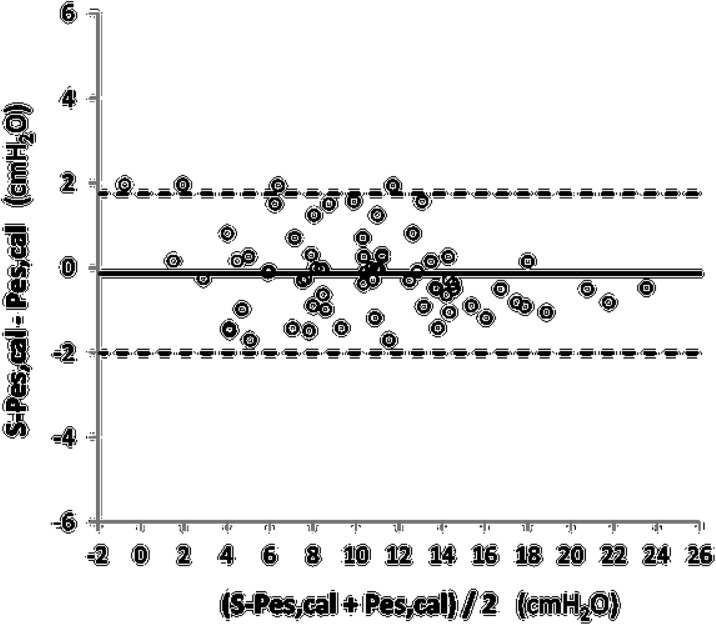
Comparison between simplified and standard procedure for calibration of the esophageal catheter: Bland-Altman analysis. Continuous line refers to mean value and dotted lines mark ± 1.98 SD of S-Pes,cal - Pes,cal difference

**Results:** In the 35 conditions tested, Vmin was 1.4 ± 0.5 ml, Vbest 4.2 ± 1.9 ml and Ees 1.2 ± 0.3 cmH_2_O/ml. At optimal filling volume (Vbest), difference between Pes and Pes,cal was 3.1 ± 1.9 cmH_2_O (range 0.0-8.0). S-Pes,cal strictly correlated with Pes,cal (R^2^ = 0.97; p < 0.0001); difference between S-Pes,cal and Pes,cal was −0.1 ± 0.9 cmH_2_O (Figure 102).

**Conclusions:** When optimal filling of the esophageal balloon is adopted in mechanically ventilated patients, absolute values of Pes are affected by significant esophageal artifact. A simplified calibration procedure seems to be adequately accurate in removing this artifact and suitable for clinical use.

**References**

1) Mojoli et al. Critical Care 2016, 20:98.

**Grant acknowledgment**

None

### A1045 Validation of indices to assess respiratory muscle effort in ventilated ICU patients

#### D. Jansen, S. Gadgil, J. Doorduin, L. Roesthuis, J.G. van der Hoeven, L.M.A. Heunks

##### Radboudumc, Intensive Care, Nijmegen, Netherlands

###### **Correspondence:** D. Jansen – Radboudumc, Intensive Care, Nijmegen, Netherlands

**Introduction:** Mechanical ventilation unloads the inspiratory muscles in case of high work of breathing to prevent development of muscle injury and patient discomfort. On the other hand, over-assist is associated with disuse atrophy and patient-ventilator asynchrony. Two indices for assessing respiratory muscle effort have recently been published. Patient-ventilator breath contribution (PVBC) index provides an estimation for the percentage of the total work of breathing performed by the patient related to the total work of breathing (patient + ventilator). Neuromuscular efficiency (NME) or pressure-electrical activity index (PEi) expresses how much pressure the inspiratory muscles generate (Pmus) for each microvolt of diaphragm electrical activity (EAdi)*.*

**Objectives:** The aim of the current study was to assess the repeatability of both PVBC and NME and investigate how these indices changes in time in ventilated ICU patients.

**Methods:** We included 31 mechanically ventilated adult ICU patients with a dedicated naso-gastric feeding tube for assessing diaphragmatic EMG activity (EAdi catheter). PVBC and NME were calculated at inclusion, after 12, 24 and 72 hrs and repeated 5 times each with a 1-minute interval. PVBC was calculated by (tidal volume no assist/EAdi no assist)/(tidal volume assist/Edi assist). NME was calculated by measuring change in airway pressure divided by amplitude of the electrical activity during end expiratory occlusion (delta Paw/Edi).

**Results:** The repeatability coefficient (RC) of PVBC and NME was 20 % and 0.34 cm H_2_O/uV respectively. Median PVBC at T = 0 was 76 % and decreased until 71 % at T = 72. In the same period, the mean NAVA level decreased (0.88 until 0.73) and mean EAdi_peak_ increased (14.2 until 18.9uV). Five patients had a PVBC index >100 %; four of them had a calculated pressure support (mean EAdi_peak_ x NAVA level) < 5cmH_2_O. The median NME was 0.90H_2_O/uV [IQR; 0.60-1.43 cm] and did not significantly change during the first 72 hrs. The median pressure generated by the diaphragm under mechanical ventilation was 9.3cmH_2_O without a significant change in time (8.7 cmH_2_O, 9.1 cmH_2_O, 10.6 cmH_2_O and 7.3cmH_2_O for T = 0, T = 12, T = 24 and T = 72 respectively).

**Conclusions:** We showed a repeatability of 20 % for PVBC and 0.34cmH_2_O/uV for NME. This means that the absolute difference between two repeated measurements lies between this value with a probability of 95 %. For example, with a calculated PVBC of 70 % it is expected that 95 % of the subsequent measurements will be between 50-90 %. NME was much more heterogeneous which indicates that neuromechanical coupling changes during ICU stay in an unpredictable manner. Pressure developed by the diaphragm in our patients appears within physiological limits.

### A1046 Use of sigmoid regression for determining the optimal balloon volume in esophageal pressure monitoring: a bench and clinical feasibility study

#### G.-Q. Chen, X.-M. Sun, X. He, Y.-L. Yang, Z.-H. Shi, M. Xu, J.-X. Zhou

##### Beijing Tiantan Hospital, Capital Medical University, Department of Critical Care Medicine, Beijing, China

###### **Correspondence:** J.-X. Zhou – Beijing Tiantan Hospital, Capital Medical University, Department of Critical Care Medicine, Beijing, China

**Introduction:** Esophageal pressure (P_ES_), which has been used as a substitute for pleural pressure, is commonly measured by catheter with air-filled balloon. The accuracy of measurement depends on the proper balloon volume (V_B_). Assessment of optimal V_B_ is difficult in clinical settings because the surrounding pressure of the balloon cannot be directly measured. In the present study, we introduced a sigmoid fitting method for determining the optimal V_B_.

**Objectives:** To assess the accuracy of optimal V_B_ measured by sigmoid fitting and to evaluate the feasibility of this method in clinical practice.

**Fig. 103 (abstract A1046).**
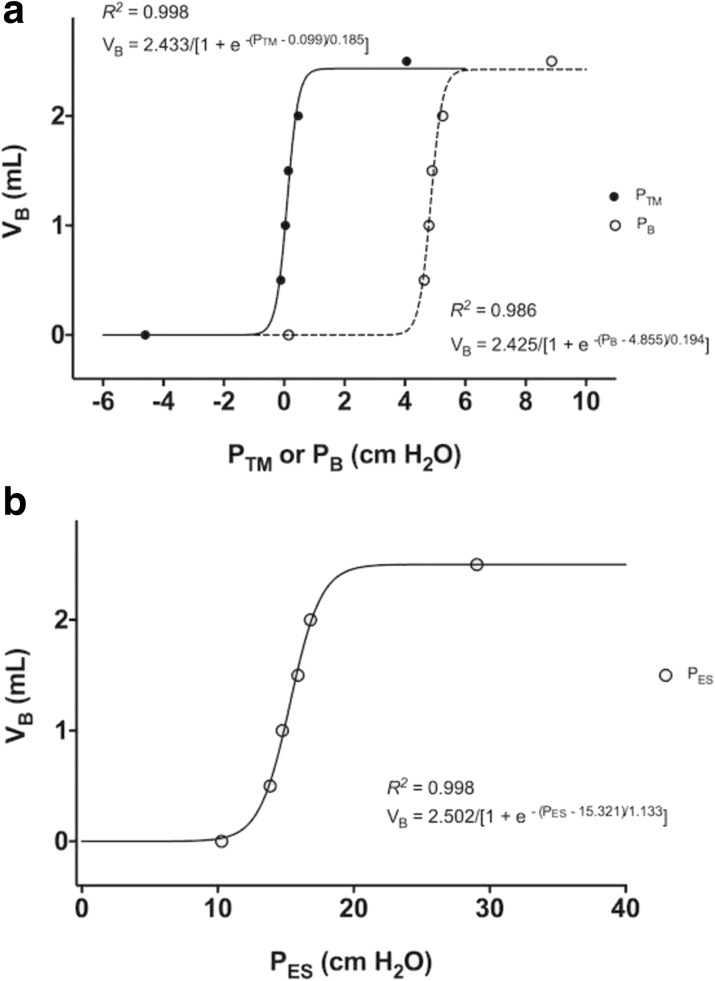
ᅟ

**Methods:** Six randomly selected esophageal balloon catheters (Cooper catheter, Cooper Surgical, USA) were tested in a bench model with the lung and the pleural cavity during simulated mechanical ventilation. The balloon was progressively inflated in 0.5 mL increments from 0 to 2.5 mL, and pressure in the balloon pressure (P_B_) and in the pleural cavity (P_C_) were measured. Balloon transmural pressure (P_TM_) was calculated as P_B_-P_C_. Balloon pressure-volume was fitted by a sigmoid regression: V_B_ = *a*/[1 + e^-(P-*b*)/*c*^], where *a* = the vertical distance of the upper asymptote, *b* = the pressure at the midpoint between zero and *a*, and *c* = the pressure range with the greatest volume change (Figure 103A shows a sample curve). The optimal V_B_ was predicted by zero P_TM_ and zero (P_B_-*b*). Bland-Altman´s analysis was used to assess the accuracy of the optimal V_B_ predicted by P_TM_ and P_B_. The balloon catheter was introduced into lower third of esophagus in 10 patients with mechanical ventilation, and the balloon was inflated as the same sequence as that in the bench study. P_ES_ and V_B_ were also fitted by the sigmoid regression (Figure 103B shows a sample curve) and the optimal V_B_ was predicted by zero (P_ES_-*b*). At each V_B_, dynamic occlusion test was performed, and ratio of changes in P_ES_ and airway pressure (ΔP_ES_/ΔP_AW_) was calculated.

**Results:** In the bench study, the best-fit coefficient *R*^*2*^ of sigmoid regression ranged from 0.918 to > 0.999 with a median (interquartile range, IQR) of 0.989 (0.972, 0.998). The natural logarithmically transformed bias (and lower to upper limit of agreement) in optimal V_B_ predicted by P_TM_ and P_B_ was −0.001 (−0.012 to 0.010). In the clinical study, 20 V_B_ tests were performed. *R*^*2*^ of sigmoid regression ranged from 0.770 to >0.999 with a median (IQR) of 0.986 (0.972, 0.995). The optimal V_B_ was 1.2 (1.2, 1.2) mL. The *b* value (*R* = 0.847, p < 0.001) and predicted optimal V_B_ (*R* = 0.348, p = 0.028) significantly correlated with respective P_ES_ measured at the V_B_ with the best ΔP_ES_/ΔP_AW_ ratio.

**Conclusions:** In the determination of balloon pressure-volume response, the performance of nonlinear sigmoid fitting was excellent in the bench model and acceptable in the clinical situation. P_B_ (P_ES_) alone can be used to predict the optimal V_B_. The sigmoid fitting method might provide a practical method for individual optimal V_B_ determination at the bedside.

### A1047 Optimizing intraoperative mechanical ventilation using electrical impedance tomography (EIT)-titrated PEEP: preliminary results

#### S.M. Pereira^1,2^, M.R. Tucci^2^, B.F.F. Tonelotto^3^, C.M. Simoes^1,3^, C.C.A. Morais^2^, M.S. Pompeo^1^, F.U. Kay^4^, M.B.P. Amato^2^, J.E. Vieira^1^

##### ^1^University of Sao Paulo, Faculty of Medicine, Hospital das Clínicas, Anesthesia, Critical Care and Pain, Sao Paulo, Brazil; ^2^University of Sao Paulo, Faculty of Medicine, Hospital das Clínicas, Pulmonary and Critical Care, Sao Paulo, Brazil; ^3^Hospital Sirio Libanes, Anesthesia, Critical Care and Pain, Sao Paulo, Brazil; ^4^University of Sao Paulo, Faculty of Medicine, Hospital das Clínicas, Radiology, Sao Paulo, Brazil

###### **Correspondence:** S.M. Pereira – University of Sao Paulo, Faculty of Medicine, Hospital das Clínicas, Anesthesia, Critical Care and Pain, Sao Paulo, Brazil

**Introduction:** The use of more physiological tidal volumes (6–8 mL/Kg of ideal body weight) during general anesthesia can minimize the risk of lung injury but may be associated with increased atelectasis. A recent meta-analysis has suggested that high driving pressure and PEEP level changes that result in an increase of driving pressure are associated with more postoperative pulmonary complications (1). There is no consensus, however, on how to tailor the level of PEEP to best suit each patient.

**Objectives:** Our primary objective is to evaluate the variability of PEEP titrated by EIT in healthy patients submitted to elective abdominal surgery. Our secondary objective is to compare the consequences on lung mechanics and on the formation of atelectasis during abdominal surgery in two groups: titrated PEEP or PEEP of 4cmH_2_O.

**Methods:** Forty patients will be allocated into two groups: laparoscopic (n = 20) or open surgery (n = 20). After induction of anesthesia and neuromuscular blockade, and before insufflation of abdominal cavity, all patients will be submitted to a recruitment maneuver (RM) in pressure-controlled ventilation mode for two minutes followed by a decremental PEEP titration starting at PEEP of 20 and diminished in steps of 2 cmH_2_O. Optimal PEEP is defined as that with the best compromise of atelectasis and overdistention as measured by EIT.

Patients in each subgroup will be randomized to one of two ventilatory strategies during intraoperative period:

1. PEEP chosen by the PEEP titration procedure (titrated PEEP);
2. PEEP set at 4 cm of H_2_O (PEEP4). A chest CT will be performed one hour after extubation. A density range of −200 to +100 Hounsfield units (HU) was used to define atelectasis.

**Results:** Thirty nine patients have been recruited. The median of titrated PEEP was 12 (IQ 10–14) (Table 68).

**Fig. 104 (abstract A1047).**
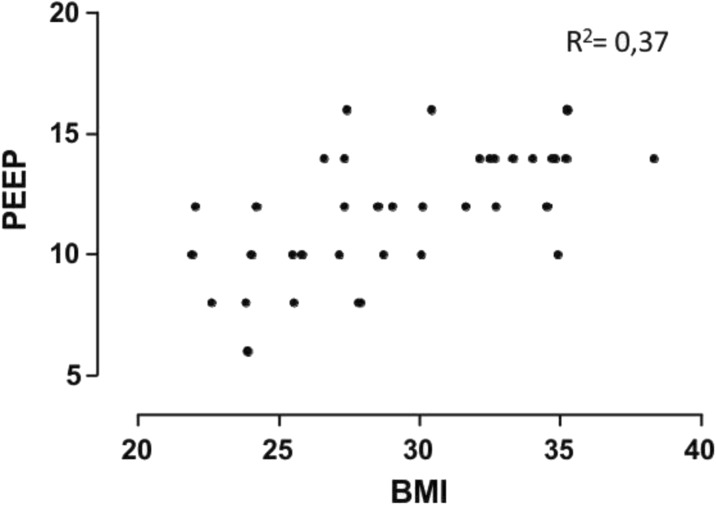
PEEP and BMI

A weak correlation between BMI and titrated PEEP (R^2^ = 0.37) is shown in figure 104.

Lung compliance was significantly lower and driving pressure was significantly higher at baseline, with PEEP = 4 and before RM, when compared to same measures using titrated PEEP during PEEP titration (Table 69).

**Fig. 105 (abstract A1047).**
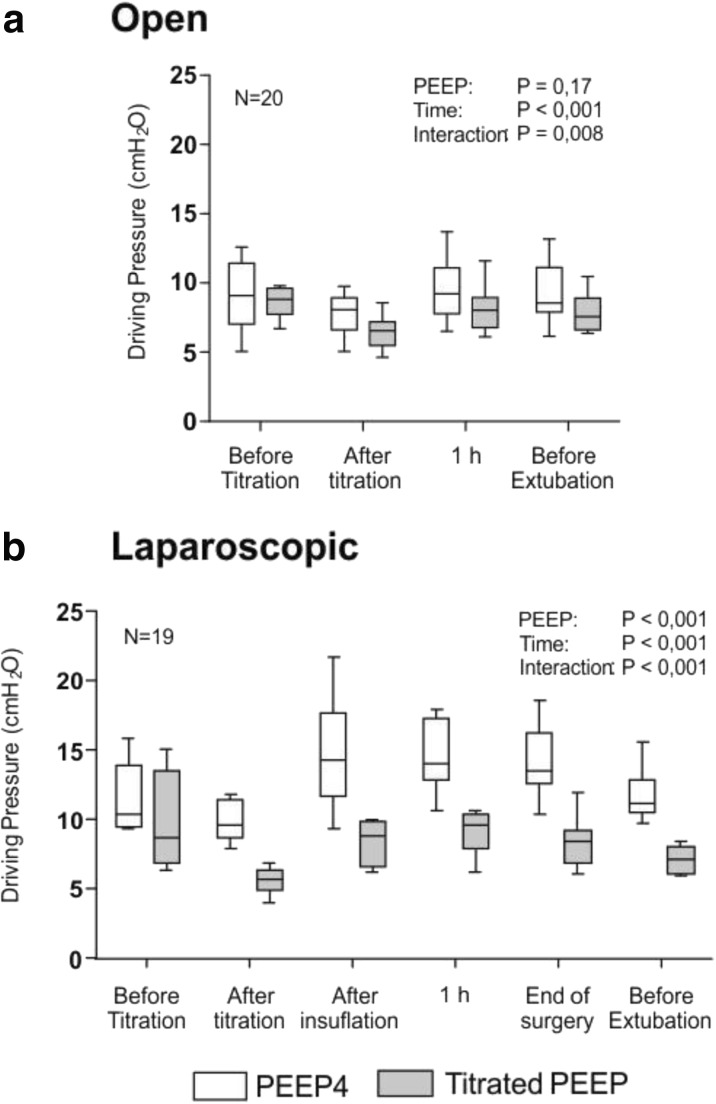
Driving pressure

During surgery, compliance (p < 0,01) and driving pressure (p < 0,01) were also significantly different between PEEP4 and titrated-PEEP group (figure 105).

Lung collapse evaluated through lung CT after extubation presented less non-aerated lung tissue in patients submitted to mechanical ventilation under EIT-titrated PEEP.

**Conclusions:** In this sample of 39 patients, the individualized value of PEEP titrated by EIT had a great variability. PEEP titrated by EIT was able to reduce both lung collapse and driving pressure.

**Table 68 (abstract A1047).** Population and titrated PEEP

	Mean/Median	Min-Max
Age (years)	52.3 ± 11.6	26–74
BMI (kg/m^2^)	29.5 ± 4.3	21.9–38.3
Male Gender (N)	18 (46.2 %)	-
Titrated PEEP (cmH_2_O)	12 (10–14)	6–16

**Table 69 (abstract A1047).** Mechanical ventilation patterns

	Laparoscopic Surgery	Laparoscopic Surgery	Open Abdominal Surgery	Open Abdominal Surgery
	PEEP 4 (N = 10)	Titrated PEEP (N = 9)	PEEP 4 (N = 10)	Titrated PEEP (N = 10)
Body Mass Index (BMI)	33.3 ± 2.4	30.2 ± 4.9	27.9 ± 3.9	26.7 ± 3.1
Ideal Body Weight (kg)	54.1 ± 12.5	55.8 ± 7.5	54.4 ± 7.2	62.4 ± 8.3
Tidal volume (ml)	325 ± 75	335 ± 45	326 ± 43	374 ± 50
Titrated PEEP (cmH_2_O)	14 (12–16)	14 (12–14)	10 (10–12)	10 (8–10)
Lung Compliance with PEEP 4 before recruitment maneuver (mL/cmH_2_O)	33.5 ± 8.1	38.2 ± 10.1	42.2 ± 15.7	43.5 ± 7.9
Lung Compliance with Titrated PEEP (mL/cmH_2_O)	77.1 ± 14	75.4 ± 9.1	75.9 ± 17.9	71.9 ± 15.7
Driving Pressure with PEEP 4 before recruitment maneuver (cmH_2_O)	11.6 ± 2.5	9.9 ± 3.3	9.2 ± 2.5	9 ± 1.9
Driving Pressure with Titrated PEEP (cmH_2_O)	5.3 ± 0.7	5.2 ± 0.7	6 ± 1.3	6.5 ± 1.1

**Fig. 106 (abstract A1047).**
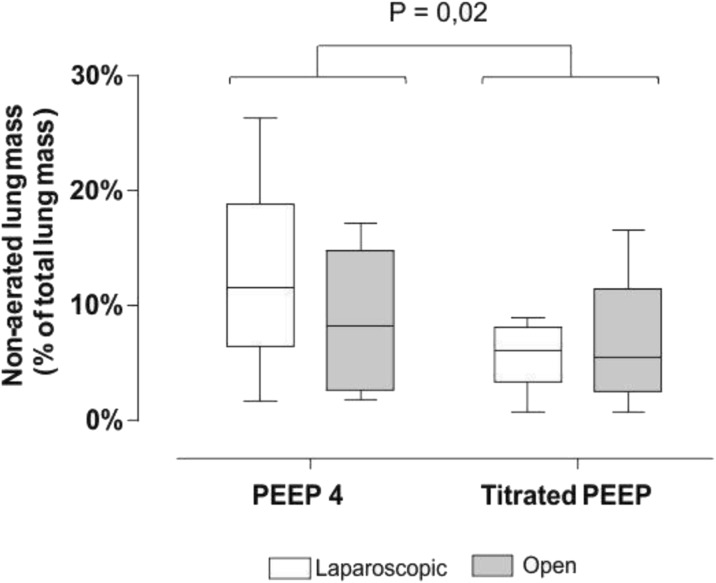
Non-aerated Lung - CT

**References**

1. Serpa Neto A et al. Association between driving pressure and development of postoperative pulmonary complications in patients undergoing mechanical ventilation for general anesthesia: a meta-analysis of individual patient data. Lancet Respir Med 2016.

**Grant acknowledgment**

FAPESP - Fundação de Amparo à Pesquisa do Estado de São Paulo

## PERIOPERATIVE INTENSIVE CARE AND TRANSFUSION

### A1048 Current oxygen management during general anesthesia: a multicenter cross-sectional study

#### S. Suzuki^1^, Y. Mihara^2^, Y. Hikasa^2^, S. Okahara^2^, H. Morimatsu^2^, On behalf of Okayama Research Investigation Organizing Network (ORION) investigators

##### ^1^Okayama University Hospital, Department of Intensive Care, Okayama, Japan; ^2^Okayama University Medical School, Department of Anesthesiology and Resuscitology, Okayama, Japan

###### **Correspondence:** S. Suzuki – Okayama University Hospital, Department of Intensive Care, Okayama, Japan

**Introduction:** Lung protective ventilation strategies could improve clinical outcomes in patients undergoing surgery. These strategies did not include specific goals for oxygenation. There is increasing recognition of potential harmful effect of hyperoxia in critically ill patients. However, little is known about current oxygen management during surgery.

**Objectives:** To describe current oxygen administration during general anesthesia in Japanese hospitals.

**Methods:** A multicenter cross-sectional study was conducted. We screened all consecutive adult patients (≥16 years) who received general anesthesia from 14 to 18 September 2015 or from 9 to 13 November 2015 at the participating hospitals (each participating hospital could choose whichever was more convenient). Ventilator settings and the corresponding vital signs were collected 1 hour after the induction of general anesthesia. We investigated the prevalence and risk factors for excess oxygen exposure (F_I_O_2_ > 0.5 despite SpO_2_ > 92 %). The study was registered at UMIN-CTR (Identifier: UMIN000018884).

**Results:** We enrolled 1,498 patients from 43 centers in Japan. The median age was 65 years [interquartile range (IQR), 48–74]; 732 (51 %) were male. One hour after the induction of general anesthesia, volume control ventilation was used in 778 patients (52 %). The median tidal volume was 8.2 ml/kg of predicted body weight [IQR, 7.3-9.2] and PEEP was applied in 956 patients (64 %). The median F_I_O_2_ was 0.47 [IQR, 0.4-0.6] and only 1 % of patients (n = 13) received F_I_O_2_ of 0.21-0.3. In more than 80 % of patients, SpO_2_ was 98 % or greater despite the corresponding F_I_O_2_ > 0.21. A total of 483 patients (32 %) were exposed to excess oxygen. In multivariate analysis, one-lung ventilation was independently associated with greater exposure to excess oxygen (adjusted odds ratio, 12.9; 95 % confidence interval, [7.19-23.1]).

**Conclusions:** Current intraoperative oxygen management may be suboptimal especially in patients during one-lung ventilation and further investigations are warranted.

### A1049 Pretransplant cystatin c and risk of 30-day cardiovascular events and all-cause mortality in liver transplant recipients with normal serum creatinine levels

#### H.-M. Kwon^1^, Y.-J. Moon^1^, S.-H. Lee^1^, K.-W. Jung^1^, W.-J. Shin^1^, I.-G. Jun^1^, J.-G. Song^1^, G.-S. Hwang^1^

##### ^1^Asan Medical Center, University of Ulsan College of Medicine, Department of Anesthesiology and Pain Medicine, Laboratory for Cardiovascular Dynamics, Seoul, Republic of Korea

###### **Correspondence:** H.-M. Kwon – Asan Medical Center, University of Ulsan College of Medicine, Department of Anesthesiology and Pain Medicine, Laboratory for Cardiovascular Dynamics, Seoul, Republic of Korea

**Background:.** Acute kidney injury (AKI) is common and is associated with significant morbidity and mortality after liver transplantation (LT). Although the creatinine value is highly specific to estimate renal dysfunction, an inadequate sensitivity of creatinine level is demonstrated, particularly in early stage AKI. Cystatin C is founded to be a stronger predictor of the risk of cardiovascular events and death than creatinine. We aimed to determine whether pretransplant serum levels of cystatin C predict 30-day major cardiovascular events (MACE) and all-cause mortality in LT recipients with normal serum creatinine values.

**Methods:** Between May 2010 and October 2015, 1187 consecutive LT recipients (mean age: 53 years; 75 % male; 97 % living-donor LT) who have pretransplant creatinine level < 1.4 mg/dL were retrospectively evaluated. The 30-day MACE was a composite of troponin I > 0.2 pg/mL, arrhythmias, congestive heart failure, death, cerebrovascular accidents.

**Results:** There was a 19.1 % 30-day MACE event and 5.2 % of LT recipients were dead during a median of 2.1 years follow-up. Mean values of cystatin C and creatinine were 0.91 ± 0.37 mg/dL and 0.77 ± 0.21 mg/dL, respectively. The risk for a 30-day MACE event increased significantly with increasing quartiles of cystatin C; hazard ratios ranged from 1.13 to 2.73 for the highest versus the lowest quartile (P < 0.001 for trend). The Kaplan-Meier curves showed that the highest quartile (cystatin C > 1.0 mg/dL) had a significantly worse survival rate than the lowest quartile (cystatin C < 0.7 mg/dL) (log-rank P = 0.023). However, pretransplant creatinine level showed neither increasing MACE event rate nor worse survival rate with increasing quartiles of creatinine values (P = 0.094 for trends, log-rank P = 0.082, respectively).

**Conclusions:** Our results demonstrate that pretransplant cystatin C levels were significantly and progressively associated with 30-day MACE and all-cause mortality in LT recipients with normal serum creatinine values, in contrast, the creatinine levels were not significant and gradual predictor of adverse clinical outcomes.

**Fig. 107 (abstract A1049).**
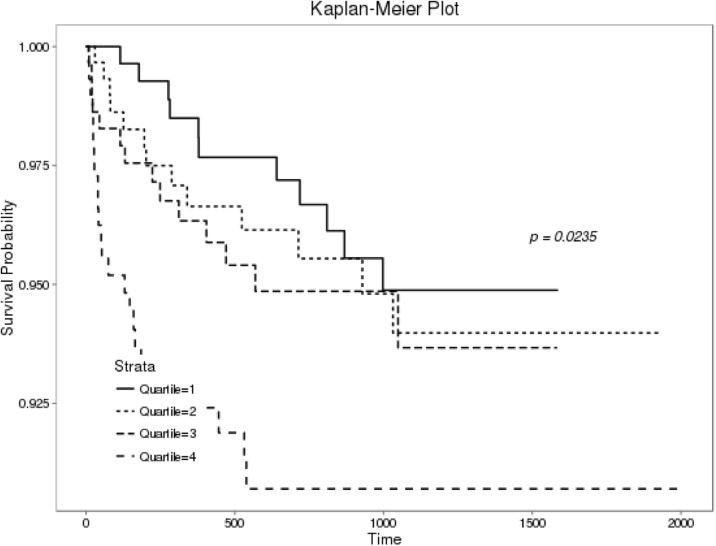
Survival probability

### A1050 Pretransplant arterial blood pressure and risk of postoperative acute kidney injury in liver transplant recipients

#### S. Lee, Y.-J. Moon, H.-M. Kwon, K. Jung, W.-J. Shin, I.-G. Jun, J.-G. Song, G.-S. Hwang

##### Asan Medical Center, University of Ulsan College of Medicine, Department of Anesthesiology and Pain Medicine, Laboratory for Cardiovascular Dynamics, Seoul, Republic of Korea

###### **Correspondence:** S. Lee – Asan Medical Center, University of Ulsan College of Medicine, Department of Anesthesiology and Pain Medicine, Laboratory for Cardiovascular Dynamics, Seoul, Republic of Korea

**Introduction:** The pathophysiology of hepatorenal syndrome is considered to be the extreme underfilling of the arterial circulation secondary to arterial vasodilatation in the splanchnic vascular bed. In the current study, we thus hypothesized that pretransplant low mean arterial pressure (MAP) may be predictive of the development of postoperative acute kidney injury (AKI) in liver transplant (LT) recipients..

**Objectives:** To explore the relationship between pretransplant mean atterial pressure and the development of postopertative acute kidney injury in liver transplant recipients

**Methods:** Of the 1531 patients who have normal pretransplant creatinine level (<1.5 mg/dL) between May 2010 and October 2015, complete electronic medical records of 1200 consecutive LT recipients (mean age: 53 years; 76 % male; 95 % living-donor LT) were retrospectively evaluated. MAP was collected from the average of preoperative daily blood pressure measurements. Postoperative AKI was defined as 2012 KDIGO (Kidney Diseases: Improving Global Outcomes) criteria.

**Results:** The overall prevalence of AKI was 42.5 % in this study cohort. A quartile (Q) analysis of MBP showed range of MAP: 59–75 mmHg (Q1), 76-81 mmHg (Q2), 82–88 mmHg (Q3), 88–122 mmHg (Q4), respectively. With decreasing quartiles of MAP, preoperative mean values of estimated GFR (cystatin C-glomerular filtration rate) were decreased (P < 0.001), and prevalence of postoperative AKI was increased (Q4: 44 %, Q3: 52 %, Q2: 57 %, Q1: 63 %, respectively, P < 0.001). Odds ratios for AKI ranged from 1.39 to 2.16 for the highest versus the lowest quartile (P < 0.001 for trend). On the multivariate logistic analysis, low MAP was an independent risk factor of the postoperative AKI (P < 0.001), after adjusting factors of age, sex, body mass index, diabetes, hypertension, creatinine, QTc interval, MELD score, B-type natriuretic peptide, beta blocker uses, intraoperative red blood cell uses, postreperfusion syndrome, and cyclosporine uses.

**Conclusions:** Our results demonstrate that pretransplant low MAP was significantly and progressively associated with the postoperative AKI in LT recipients with normal serum creatinine values, therefore, our findings may assist in determining the optimal perioperative management of patients to prevent postoperative AKI.

**References**

1. Stadlbauer, V., et al., *Relationship between activation of the sympathetic nervous system and renal blood flow autoregulation in cirrhosis.* Gastroenterology, 2008. **134**(1): p. 111–9.2.

2. Velez, J.C., et al., *Hepatorenal Acute Kidney Injury and the Importance of Raising Mean Arterial Pressure.* Nephron, 2015. **131**(3): p. 191–201.

**Grant acknowledgment**

No conflicts of interest declared. This research was carried out without funding.

### A1051 Evaluation of changes in lung ultrasound patterns after chest physiotherapy in patients undergoing cardiac surgery

#### A. Ramelli^1^, T. Manca^1^, F. Corradi^2^, C. Brusasco^2^, F. Nicolini^1^, T. Gherli^1^, R. Brianti^1^, P. Fanzaghi^1^, A. Vezzani^1^

##### ^1^University Hospital of Parma, Parma, Italy; ^2^E.O. Ospedali Galliera, Genoa, Italy

###### **Correspondence:** A. Ramelli – University Hospital of Parma, Parma, Italy

**Introduction:** Patients undergoing cardiac surgery often develop, in the post operative period, pulmonary impairment and abnormalities gas exchanges (1). Lung ultrasound (LUS) examination may detect main pulmonary abnormalities at the bedside of the patient (2). To increase bronchial drainage and help lung reaeration, physiotherapy treatment is daily applied starting from the first day after cardiac surgery.

**Objectives:** Our study was to evaluate if physiotherapy treatment was able to induce changing in lung ultrasound pattern in the postoperative patients.

**Methods:** n 19 cardiac surgery patients (11males), 6 h after estubation, we performed a LUS evaluation in six areas of each hemithorax. The LUS pattern was evaluated and a score of loss of aeration assigned: no loss of aeration in presence of A lines with lung sliding (point 0)and no more then 2 B lines (point 1), moderate decrease in aeration with several not coalescent B lines (point 2), severe in presence of coalescent B lines (point 3); complete loss of aeration in presence of consolidation with (point 4) or without( point 5) air bronchogram. After chest physiotherapy treatment a new complete LUS evaluation was repeated and the score of loss of aeration re-calculated.

**Results:** In 16 patients we detected consolidations in the bilateral inferior areas: in 4 patients consolidations were monolateral. Severe decrease in aeration or coalescent B lines were identified in 13 patients while moderate decrease in aeration was identified in the anterior areas in all patients. After treatment no change was identified in consolidated areas while an increase of aeration was identified in areas with moderate and severe decrease in aeration.

Compared total loss of aeration, before and after treatment, we identified a significant increase of rearation after physiotherapy (p = 0,046) evaluated with Wilcoxon test.

**Conclusions:** Our results confirm an elevate rate of loss of aereation in patients after cardiac surgery. Physiotherapy may induce increase of reareation when evaluated with LUS even thought it is not able to reduce consolidation.

**References**

1) Urell C, Westerdahl E, Hedenstrom H, Janson C, Emtner M. Lung fuction before and two days after open-heart surgery. Crit Care Res Pract 2012; 2012: 291628

2) Vezzani A, Manca T, et al. Diagnostic value of chest ultrasound after cardiac surgery: a comparison with chest X-ray and auscultation. J Cardiothorac Vasc Anesth. 2014 Dec;28(6):1527–32

### A1052 Increased heat shock protein 27 serum levels in patients undergoing stapler hepatectomy and CUSA resection

#### B.-A. Tudor^1^, D.A. Klaus^1^, D. Lebherz-Eichinger^1^, C. Lechner^1^, C. Schwarz^2^, M. Bodingbauer^2^, R. Seemann^3^, K. Kaczirek^2^, E. Fleischmann^1^, G.A. Roth^1^, C.-G. Krenn^1^

##### ^1^Medical University of Vienna, Department of Anesthesiology, General Intensive Care and Pain Medicine, Vienna, Austria; ^2^Medical University of Vienna, Department of Surgery/Div. of General Surgery, Vienna, Austria; ^3^Medical University of Vienna, Department of Cranio-, Maxillofacial and Oral Surgery, Vienna, Austria

###### **Correspondence:** B.-A. Tudor – Medical University of Vienna, Department of Anesthesiology, General Intensive Care and Pain Medicine, Vienna, Austria

**Introduction:** The therapy of malignant liver diseases has changed over the last 100 years. During this period the frequency of liver resection has increased with great improvement in morbidity, mortality and long-term survival. [1] Thereby, the duration of liver transection and the amount of perioperative blood loss are of great importance for postoperative recovery time and therefore they are measures for choosing the optimal resection method. [2] Furthermore, the release of cytokines, chemokines, and stress hormones correlates with postoperative infection and organ dysfunction [3].

To minimize cell damage and limit apoptotic cell death the so called heat shock response is initialized by various body cells as countermeasure to increased stress levels [4]. Moreover, Pittet et al. showed a positive correlation between the small heat shock protein 27 (HSP72) serum levels and survival after severe trauma. [5]

**Objectives:** Measurement of HSP27 could give an insight about pathological mechanism and their counter regulations of the liver. Furthermore the HSP27 serum level should be correlated with the transection speed of the two resection methods CUSA and Stabler.

**Methods:** Of 40 patients undergoing hepatic resection (CUSA-group: 20 patients, STAPLER-group: 20 patients) we collected serum at following time points: pre-surgery (systemic), pre-resection (systemic, portal vein, hepatic vein), post-resection (systemic, portal vein, hepatic vein), post-surgery (systemic) and on the 1 and 3 post-operative day (systemic). Immediately after collection, samples were aliquoted, snap frozen and stored at −80 °C until further analyzation. To quantify HSP27 in serum commercially available ELISA kits from R&D (DuoSet IC) have been used according to the manufacturer's protocol. Furthermore the duration of transection and the resection surface expressed as cm^2^/s were recorded.

**Results:** During surgery a significant increase in HSP27 levels was detected in patients undergoing Stabler hepatectomy or CUSA resection (n = 40, p < 0.05). During postoperative ICU stay, HSP27 concentrations decline to levels comparable before surgery.

The transection speed was significant faster in patients undergoing stapler resection compared to the CUSA method (p < 0.0001).

The mean length of ICU stay after liver resection was in both groups 2 days.

**Conclusions:** Our data show increased levels in serum of HSP27, which might reflect the body's countermeasure to increased systemic stress levels during hepatectomy. Moreover the HSP27 levels are in both groups equal high during surgery even though the resection conducted with the Stabler is significant faster than CUSA.

**References**

1. Blumgart LH et.al. Elsevier 2007.

2. Taketomi A, et.al. JACS 2007.

3. Kimur a F et.al. JSurg Res 2006.

4. Arya R et.al. JBiosci 2007.

5. Hashiguchi N et.al. JTrauma.

### A1053 Effects of long-term pneumoperitoneum during laparoscopic surgery on respiratory function

#### A. Malyshev, S. Sergey

##### Pirogov Russian National Research Medical University, Anesthesiology and Intensive Care Medicine, Moscow, Russian Federation

###### **Correspondence:** A. Malyshev – Pirogov Russian National Research Medical University, Anesthesiology and Intensive Care Medicine, Moscow, Russian Federation

**Introduction:** Performing laparoscopic surgery using carboxipneumoperitoneum usually accompanied with a moderate increase of the concentration of carbon dioxide at the end of expiration, as well as higher peak airway pressure that easily manages to compensate by the correction of ventilation parameters. In the postoperative period marked a fairly long recovery of baseline respiratory function associated not only with the post-operative pain, but with the restriction of the lung as a result of intra-abdominal hypertension.

**Objectives:** Assess the impact of prolonged pneumoperitoneum during laparoscopic surgery on respiratory function and to follow the dynamic of its rehabilitation.

**Methods:** The study included 94 patients (42 men and 52 women) in the age of 60.3 years (min 32, max 86), operated in Moscow municipal hospital №4. The volume of surgical procedures: gastric resection (n = 18), gastrectomy (n = 5), pancreatoduodenal resection (n = 8), hemicolectomy (n = 39), resection of the sigmoid colon (n = 18), anterior resection of rectum (n = 6). Depending on the surgical access patients were divided into two study groups: 1st - basic - (n = 33) group - laparoscopic procedures, 2nd - control - (n = 61) group of traditional laparotomy. All patients were under equal anesthesia during surgery: combined general anesthesia (sevoflurane + fentanil) and epidural infusion of 0.2 % ropivacaine solution, as well as myoplegia; postoperative multimodal analgesia: nonsteroidal anti-inflammatory drugs, antispasmodics, epidural analgesia. A study of respiratory function was carried out in four stages: 1 - before surgery, 2 - 2nd, 3 - 6th, 4- 11th day after surgery.

**Results:** In patients of both groups to the second stage of study determined a significant reduction of volume parameters of respiratory function (VC, FVC, FEV, FEV1, MEF, MVV etc.). For example VC decreased in patients of group 1 by 41 % against the initial values, and 52 % of patients in group 2 (dynamics presented in the diagram). Similarly changes in VC there is a decrease of all volume parameters: FEV1 for the second phase decreased by 39.1 % in group 1 and 48 % in group 2; MEF decreased by 61.5 % and 74.3 % in the first and second groups, respectively. However, in addition to a statistically smaller decrease in the absolute values of volumetric parameters of respiratory function in the 1st group, we found them more intense recovery.

**Conclusions:** Reducing the volume indicators of respiratory function after extensive laparoscopic surgery is less than after similar in volume laparotomy. Recovery of acquired restrictive respiratory disorders is more intense and after laparoscopic surgery. At the same time in either group studies we have not observed a complete rehabilitation the initial levels of respiratory parameters, even after 11 days after surgery.

**Fig. 108 (abstract A1053).**
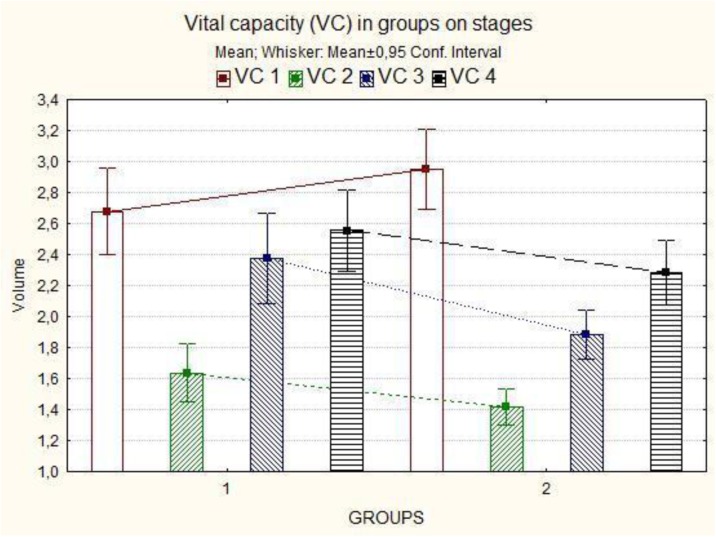
Vital capacity intwo groups on four stages

### A1054 Ultrasonographic assessment of optic nerve sheath diameter during carbondioxide pneumoperitoneum and trendelenburg position

#### Y. Yamaguchi^1^, T. Nomura^1^, E. Yoshitake^2^, M. Idei^1^, T. Yoshida^1^, S. Takaki^1^, O. Yamaguchi^1^, M. Kaneko^2^, T. Goto^1^

##### ^1^Yokohama City University Graduate school of Medicine, Department of Anesthesiology and Critical Care, Yokohama, Japan; ^2^Yamato Municipal Hospital, Department of Anesthesia, Yamato, Japan

###### **Correspondence:** Y. Yamaguchi – Yokohama City University Graduate school of Medicine, Department of Anesthesiology and Critical Care, Yokohama, Japan

**Introduction:** Critically ill patients sometimes need laparoscopic surgery. It has been reported that steeped head-down position could increase intracranial pressure during robotic surgery. But we don´t know whether mild Trendelenburg position and carbon dioxide pneumoperitoneum cause intracranial hypertension. We conducted a prospective observational study.

**Objectives:** The aim of our study was to investigate the change of optic nerve sheath diameter (ONSD) in head-down position during carbon dioxide pneumoperitoneum.

**Methods:** We included patients scheduled to undergo laparoscopic gynecological surgery. Exclusion criteria were ocular disease and central nervous system diseases.

ONSD were measured 3 mm sagittal behind the globe We assessed ONSD after tracheal intubation (T baseline), after pneumoperitoneum and Trendelenburg position (T_0_) and every 30 minutes (T_30_, T_60_, T_90_, T_120_). Anesthetic management were standardized.

**Results:** Twenty seven patients were enrolled in this study. Four patients were excluded from analysis because it was difficult for us to measure ONSD. The degree of head-down angle was 13.8 ± 2.61.

**Fig. 109 (abstract A1054).**
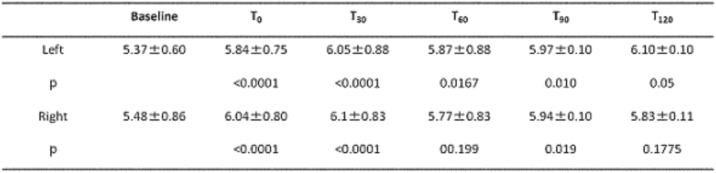
ᅟ

ONSD is significantly higher than baseline after pneumoperitoneum and Trendelenburg position (Figure 109).

**Conclusions:** Carbon dioxide pneumoperitoneum and Trendelenburg position increased intracranial pressure even if the head-down angle was mild.

### A1055 Activated partial thromboplastin time and anti-Xa measurements in heparin monitoring among critical care patients

#### N. Tencé^1^, I. Zaien^1^, M. Wolf^2^, P. Trouiller^1^, F.M. Jacobs^1^

##### ^1^CHU Antoine Béclère APHP, Intensive Care Unit, Clamart, France; ^2^CHU Antoine Béclère APHP, Department of Hematology, Clamart, France

###### **Correspondence:** F.M. Jacobs – CHU Antoine Béclère APHP, Intensive Care Unit, Clamart, France

**Introduction:** Monitoring the anticoagulant effect of unfractionated heparin (UFH) is mandatory. This monitoring can be done by the mean of the activated partial thromboplastin time (aPTT) or by anti-Xa levels measurements. Compared with anti-Xa levels testing, aPTT is more frequently impacted by preanalytic variables and biologic factors (increased levels of acute phase reactants, consumption coagulopathy) often encountered among critically ill patients. We studied the agreement of both tests results in unselected critically ill patients.

**Objectives:** To study the agreement of both tests results in unselected critically ill patients.

**Methods:** aPTT and anti-Xa levels were simultaneously monitored in patients treated by continuous intravenous infusion of UFH. Blood samples were drawn into sodium citrate tubes (Greiner Bio-One SAS, France). aPTT was measured with TriniCLOT Automated aPTT reagent (Tcoag, Ireland) and anti-Xa levels with BIOPHEN HEPARIN (LRT) (HYPHEN Biomed, France). An aPTT of 2–3 times the control and anti-Xa levels between 0.3-0.7 IU/ml were defined as therapeutic.

**Results:** Forty-four patients (mean age 71.13 ± 15.7 years; mean SAPSII 39.1 ± 14.4) were included. Reasons for admission were medical in 31, surgical in 13. The indications for UFH therapy were atrial fibrillation (26), venous thromboembolism/pulmonary embolism (13), thrombophilia (2), acute coronary syndrome (1), arterial thrombosis (2). Paired measurements of aPTT and anti-Xa were performed on 353 samples. Linear regression analysis was used to evaluate the relationship between aPTT and anti-Xa.

The correlation between aPTT and anti-Xa levels was low (r = 0.495 ) Concordant aPTT and anti-Xa values were observed in 213 (60.3 %) data pairs. aPTT was discordantly high in 96 (29.9 %) data pairs and discordantly low in 44 (12.5 %) ones. Considering anti-Xa as gold standard, monitoring anticoagulation treatment by aPTT leads to a high risk of misdosing. aPTT is frequently impacted by biologic factors. Although less commonly, anti-Xa levels can also be influenced by biological cofounders. Poor correlation between aPTT and anti-Xa could result from lterations in FII and FVIII activity.

**Conclusions:** Use of aPTT and anti-Xa levels to guide heparin therapy may lead to different estimates of UFH concentration in the same patient.

Both aPTT and anti-Xa have limitations when used for UFH monitoring and may not accurately assess anticoagulant status. Further investigation (using thromboelastometry or thrombin generation assays) could be useful to determine the optimal anticoagulation testing protocol in critically ill patients.

**Note:** This abstract has been previously published and is available at [1]. It is included here as a complete record of the abstracts from the conference.

**References**

1. Tence N, Zaien I, Wolf M, Troullier P, Jacobs F (2016) Activated partial thomboplastin time and anti-Xa measurements in heparin monitoring among critical care patients. Annals of Intensive Care 6(Suppl 1): P79.

**Table 70 (abstract A1055).** Crosstabulation of aPTT and anti-Xa levels

aPTT below therapeutic range/anti-Xa below therapeutic range	105
aPTT below therapeutic range/anti-Xa in therapeutic range	37
aPTT below therapeutic range/anti-Xa above therapeutic range	2
aPTT in therapeutic range/anti-Xa below therapeutic range	41
aPTT in therapeutic range/anti-Xa in therapeutic range	95
aPTT in therapeutic range/anti-Xa above therapeutic range	5
aPTT above therapeutic range/anti-Xa below therapeutic range	11
aPTT above therapeutic range/anti-Xa in therapeutic range	44
aPTT above therapeutic range/anti-Xa above therapeutic range	13

### A1056 A5 is an acceptable early measure of clot firmness in the bleeding trauma patient and is highly predictive of massive transfusion: retrospective analysis of ROTEM and outcome in 1146 patients

#### J.M. Kelly^1,2^, P. Veigas^2^, S. Hollands^2^, A. Min^3^, S. Rizoli^2,4^

##### ^1^University Hospitals Birmingham NHS Trust, Critical Care, Birmingham, United Kingdom; ^2^University of Toronto, Toronto, Canada; ^3^Royal College of Surgeons in Ireland, Dublin, Ireland; ^4^St Michael's Hospital, Toronto, Canada

###### **Correspondence:** J.M. Kelly – University Hospitals Birmingham NHS Trust, Critical Care, Birmingham, United Kingdom

**Introduction:** Viscohaemoelastic assays such as TEG and ROTEM are increasingly used to guide blood product transfusion decisions. Maximum clot firmness (MCF) is a ROTEM parameter available after 25–29 minutes often used to guide treatment decisions. The DEUCE investigators found clot firmness after 10 minutes (A10) is an acceptable substitute, but not after 5 minutes (A5).

**Objectives:** We aimed to confirm the correlation between A10 and MCF in the largest series of trauma ROTEMs reported, and also to look again at the correlation between A5 and MCF. We further aimed to assess A5, A10 and MCF in terms of prediction of mortality and need for massive transfusion.

**Methods:** We performed a retrospective observational analysis of a consecutive series of 1146 trauma patients with ROTEM performed on admission, in a single centre between 04/08/2011 and 30/03/2013. Firstly, we measured strength of correlation between A5 and MCF, A10 and MCF, and Fibtem A10 and Fibtem MCF using Pearson's coefficient. Secondly, we compared association of A5, A10 and MCF with mortality and massive transfusion by plotting receiver operating curves and calculating the c-statistics.

**Results:** A5, A10 and Fibtem MCF all correlated well with MCF/Fibtem MCF. Pearson correlation coefficients were as follows: A5:MCF 0.92, A10:MCF 0.96, Fibtem A10:Fibtem MCF 0.97.

A5, A10 and MCF correlated with mortality, but not strongly, and c-stat values were similar (0.67, 0.69, 0.69). However they all showed very strong correlation with need for massive transfusion, with little difference in strength: A5 c-stat 0.87, A10 c-stat 0.89, MCF c-stat 0.90.

**Conclusion:** The correlation of A5 is not quite as strong as that of A10 with MCF. However both correlate strongly. Both A5 and A10 showed predictive power almost as high as MCF for massive transfusion. Predictive power does increase marginally as time goes on. However the authors feel that in view of the acceptable correlation with MCF, and very high predictive power for massive transfusion, A5 is an acceptable pragmatic early measure of clot firmness.

We recommend that ROTEM A5 be used to inform early treatment decisions, since we do not see significant benefit in waiting for A10 or MCF.

**References**

1. Reed MJ, Nimmo AF, McGee D, Manson L, Neffendorf AE, Moir L, Donaldson LS. Rotational thrombolelastometry produces potentially clinical useful results within 10 min in bleeding emergency department patients: the DEUCE study. *Eur J Emerg Med*. 2013;20(3):160–6.

2. Meyer AS, Meyer MA, Sørensen AM, Rasmussen LS, Hansen MB, Holcomb JB, Cotton BA, Wade CE, Ostrowski SR, Johansson PI. Thrombelastography and rotational thromboelastometry early amplitudes in 182 trauma patients with clinical suspicion of severe injury. *J Trauma Acute Care Surg*. 2014;76(3):682–90.

**Grant acknowledgment**

Academic Health Sciences Alternative Funding Plan Innovation Fund 2011–2012 (Dr Rizoli)

### A1057 Thromboelastography findings in critically ill patients with sepsis

#### C.M. Coronado Robles, M.A. Montes de Oca Sandoval

##### Universidad Autonoma de México, POSGRADO, Mexico, Mexico

###### **Correspondence:** C.M. Coronado Robles – Universidad Autonoma de México, POSGRADO, Mexico, Mexico

**Introduction:** Thromboelastography (TEG) is a diagnostic method designed for a global evaluation of coagulation. It is frequently used in the clinic for the evaluation of coagulopathies and as guide for intervention. There is insufficient information regarding thromboelastography findings in patients with sepsis and its correlation with Bleeding times.

**Objectives:** To describe the findings on TEG in patients with sepsis in an ICU unit.

**Methods:** Descriptive and transversal study that includes septic patients of any etiology admitted to ABC Medical Center in México City, from March 2014 to February 2016. We evaluate TEG and coagulation tests used routinely, in the first 24 hrs of patient admission and the severity of the disease by means of sequential organ failure assessment (SOFA).

**Results:** We included data from 28 patients, 71.4 % men, 28.6 % female, with a mean age of 68 years, (IQR 61–81), an admission SOFA of 10 (IQR 7–11). Mean C reactive protein was 15.35 mg/dl. Mean procalcitonin was 3.7 ng/ml. Reaction time (R value), was normal in 75 % of patients, increased in 10.7 % (hipocoagulability), and reduced 14.3 % (Hipercoagulability), with a mean 5 (IQR 4–6). Kinetics time (K value) was normal in 85.7 %, and increased in 14.3 % (coagulation factor deficiency, diminished platelet aggregation), none of the patients had a reduced value, with a mean of 2 (IQR 2–4). The alpha Angle was normal in 75 %, increased 7.1 % (platelet hiperagregability or hiperfibrinogenemia), and low 17.9 % (Hipofibrinogenemia or anticoagulation), with a mean 60 (IQR 46–63). Maximal Amplitude (MA) normal 57.1 %, increased 7.1 % (hiperagregability platelet), reduced 35.7 % (low function or diminishing platelets), with a median of 54 (IQR 52–59). Mean for TTPA 32 sec: normal or increased in 70.4 and 29.6 % respectively. TT 18 sec: Normal, increased or low in 75, 14.3 and 7.4 %, respectively. TP 13.20 sec: Normal, increased or reduced in 60.7, 32.1 and 7.1 %. INR 1.16: Normal or increased in 57.1 and 42.9 %, respectively. Fibrinogen: median of 261, normal, increased or reduced in 51, 25, 21.4 %. Platelet count of 119 thousand, which was low in 67.9 % and normal in 32.1 %.

**Conclusions:** TEG detect 10 % of bleeding disorder in septic patients. Should be used to assess clotting disorder and provide specific treatment. Can effectively monitor the change in coagulation in patients with sepsis, and distinguish the hyper-coagulable and hypo-coagulable state. Upcoming research could determine its possible pronostic value and utility in clinical evaluation in septic patients.

**References**

1. Ostrowski SR, Windelov NA, Ibsen M, et al. Consecutive thrombelastography clot strength profiles in patients with severe sepsis and their association with 28-day mortality: a prospective study [J]. J Crit Care, 2013, 28 (3): 317.

2. Mervyn Singer, MD FRCP, Clifford S. Deutschman , MD, MS, et al. The third International Consensus Definitions for Sepsis and Septic Shock (Sepsis-3), JAMA, 2016;315(8):801–810.

### A1058 Using of prothrombin complex concentrate in obstetric massive bleeding

#### O. Tarabrin^1^, D. Gavrychenko^1^, G. Mazurenko^1^, P. Tarabrin^2^

##### ^1^Odessa National Medical University, Anesthesiology and Intensive Care, Odessa, Ukraine; ^2^Odessa National Medical University, Odessa, Ukraine

###### **Correspondence:** O. Tarabrin – Odessa National Medical University, Anesthesiology and Intensive Care, Odessa, Ukraine

**Introduction:** The severe capillary leak-induced respiratory and renal failure limit large-volume resuscitation with crystalloids and blood components. The combined use of low volumes of crystalloids and “damage control resuscitation” (DCR), a blood product resuscitation goal of a 1:1:1 ratio of packed red blood cells (PRBC), fresh frozen plasma (FFP) has recently been applied to obstetric patients in hemorrhagic shock. Another important consideration is the association of FFP with the risk of transfusion-related acute lung injury (TRALI), a major cause of death after transfusion. This risk is not present with the use of prothrombin complex concentrate (PCC) as the antibodies responsible for TRALI are removed during the manufacturing processes.

**Methods:** Our research involved 51 patients with massive bleeding after Cesarean section. Patients were divided into 2 groups: 1^st^ group contained 10 patients as a treatment of massive bleeding with coagulopathy was scheduled PCC in a dose of 1 ml/kg (25 IU/kg), packed red blood cells (PRBC). 2^nd^ group (41 patients) received fresh frozen plasma (FFP) in a dose of 20 ml/kg and PRBC. Basic infusion-transfusion therapy was administered according to the protocols of hemorrhagic shock treatment in obstetrics. Evaluation of the functional state of the hemostasis system was carried out using low-frequency pyezoelectric thromboelastography (LPTEG) on admission to hospital and every 2 hours after the patient´s admission until normalization of hemostasis state.

**Results:** According to LPTEG indicators obstetric patients with massive bleeding has a statistically significant abnormality in all parts of hemostatic system: platelet aggregation - Intensity of contact coagulation (ICC), the coagulation - Intensity of coagulation drive (ICD), clot maximum density (MA) and fibrinolytic activity - Index of retraction and clot lysis (IRCL). ICC in patients with massive bleeding was reduced by 45.64 %, ICD was less than normal at 59.32 %, MA was reduced by 88.15 %, IRCL was 86,16 % above the norm. Patients of 1^st^ group received infusion of PCC with estimation of efficiency by LPTEG, signs of ongoing bleeding and clinical signs of relief hemorrhagic shock. Indicators of platelet hemostasis characterized by persistence of hypoagregation. Patients of 2^nd^ group received FFP have hypoagregation and mild hypocoagulation state with increased active of fibrinolysis. Clinically, patients of the 1^st^ group had reducing signs blood loss, decreased volume of transfusion PRBC for 11 % and decreasing volume of infusion therapy for 19 % compared to patients of 2^nd^ group. There are 1 case of transfusion related lung injury in 2^nd^ group.

**Conclusions:** The use of prothrombin complex concentrate can reduce the level of blood loss, decrease volume of transfusion packed red blood cells and infusion therapy. Reducing the use of blood components in the intensive care unit of massive bleeding can be a method of preventing the development of TRALI-syndrome.

### A1059 Applicability of the thromboelastography in the characterization of coagulation disorders in septic patients

#### I. Palacios Garcia, A. Diaz Martin, M. Casado Mendez, V. Arellano orden, R. Leal Noval

##### Hospital Virgen del Rocio, Sevilla, Spain

###### **Correspondence:** I. Palacios Garcia – Hospital Virgen del Rocio, Sevilla, Spain

**Objectives:** Patients with severe sepsis often have abnormalities of haemostasis. In this prospective study, coagulation disorders are analysed by thromboelastography (TEG) in the early period of severe sepsis.

**Methods:** Prospective study for six months on septic patients, from any source, who required ICU admission. Definitions TEG patterns: 1. Hypercoagulability, R (reaction time) < 5 min [5–10] and/or MA (clot maximum amplitude) >70 mm [50–70]; 2. hypocoagulability, R > 10 min and/or MA < 50 mm; 3. Hyperfibrinolysis, Ly30 (clot lysis at 30 min) >8 [0-8 %] and 4. Hypofibrinolysis if Ly30 < 1 %. Quantitative variables are expressed as median [IQR].

**Results:** There were included 38 patients, 63.2 % were men (24 cases) with a median age of 64 years [58–70]. 65.8 % (25 cases) were in septic shock at admission to the ICU, with median severity scores: SOFA 5 [4–6.25] and APACHE II 8 [7–14]. The most common focus was abdominal in 24 cases (63.2 %), followed by respiratory focus (15.8 %, 6 cases).

The TEG was performed 1-day median [0–3] after ICU admission. The main TEG parameters values were: R 9 min [6.25-11.65], MA 69,80 mm [54.22-73.87] and Ly30 0 % [0–0.8]. TEG patterns were normal in 17 cases (44.7 %), hypercoagulable 8 cases (21.1 %), hypocoagulability 9 cases (23.7 %), fibrinolysis 4 patients (10.5 %) and hypofibrinolysis 24 cases (63.2 %).

The overall mortality was 13.2 % (5 cases), of which 60 % had hypercoagulable TEG pattern and 40 % moderate or severe hypocoagulability. The median time from the TEG to death in ICU was 8.5 days [3.75-13].

**Conclusion:** Over 50 % of septic patients have TEG patterns with coagulation disorders. Further studies are needed to evaluate the efficacy of TEG in these patients.

### A1060 A retrospective review of anaemia in the elderly population in ICU

#### C. McCue^1^, L. Gemmell^2^, A. MacKay^2^

##### ^1^Royal Alexandra Hospital, Anaesthetics and Intensive Care, Glasgow, United Kingdom; ^2^Queen Elizabeth University Hospital, Anaesthetics and Intensive Care, Glasgow, United Kingdom

###### **Correspondence:** C. McCue – Royal Alexandra Hospital, Anaesthetics and Intensive Care, Glasgow, United Kingdom

**Introduction:** Both anaemia and transfusion of red cells (as defined by WHO criteria) (1) have been associated with adverse outcomes, and the potential for anaemia to be a marker of a greater disease burden is frequently raised in discussion. Cohort studies of patients aged >65 years demonstrate that anaemia is associated with increased mortality (2). Anaemia is also associated with a variety of morbidities in older people, being linked with an increase in hospitalisation, poorer physiological, physical and cognitive function, development of Alzheimer´s and Parkinson´s diseases, depression, falls and hip fracture rates. We aimed to investigate whether anaemia was associated with adverse outcomes, increased lengths of stay and increased overall mortality in our ICU cohort. We also thought it would be interesting to know if there was a difference in haemoglobin level depending on the specialty in which the patients were admitted - hereby defining the physiology of their anaemic process.

**Methods:** We conducted a retrospective review of all patients over the age of 65 years that were admitted to the Victoria Infirmary, Glasgow between 01/01/2005 and 31/12/2014 using the Wardwatcher national database. We looked at admitting specialty (Medicine or Surgical), haemoglobin at admission, length of stay and hospital mortality.

**Results:** 1248 patients were included in the analysis, however full data set was available for 934 patients. The patients were more predominantly male with similar numbers in the medical and surgical groups. Medical patients were slightly younger, but with higher physiology scores and mortality. There was no statistical difference between length of stay in Intensive Care between the two groups. Medical patients had a higher admission haemoglobin but this did not trend with outcome or length of stay. (see Table 72)

**Conclusions:** Interestingly, and not as expected, it seems that admission haemoglobin to Intensive Care is not associated with outcome in the elderly population. It is noteworthy that discussion continues in the literature regarding the definition of anaemia in this age group as the population used to generate the WHO criteria did not include any over 65´s. Admission haemoglobin levels did not seem to correlate with APACHE, length of stay or outcome. However, the medical patients with more likely chronic anaemic state had higher APACHE-II scores, were younger with higher mortality than the surgical admission who were older, had better outcomes but were more significantly anaemic on admission to Intensive Care.

**References**

1. www.who.int/topics/anaemia/en

2. Zakai NA, Katz R. A prospective study of anaemia status, haemoglobin concentration and mortality in an elderly cohort. *Archives of Internal Medicine 2005;*165: 2214–2220

**Table 71 (abstract A1060).** ᅟ

	All patients	Medical	Surgical	p-value
Number of patients	934	468	466	
Male gender	54.3 %	55.3 %	53.2 %	0.51
Age (years)	74.4 +/− 0.4	73.6 +/− 0.5	75.5 +/− 0.6	<0.001
APACHE-II	21.6 +/− 0.6	24.1 +/− 0.9	19.1 +/− 0.9	<0.001
Predicted mortality (%)	44 +/− 1.8	47.8 +/− 2.6	40.1 +/− 2.3	<0.001
Hb (g/dl)	10.2 +/− 0.1	10.7 +/− 0.2	9.8 +/− 0.2	<0.001
Length of stay (days)	4.7 +/− 0.4	4.8 +/− 0.6	4.6 +/− 0.6	0.71
Outcome (hosp)	46.1 %	52.4 %	38.5 %	<0.001
ROC (Hb vs mortality) - Area under Curve	0.53	0.51	0.57	

### A1061 Experience with a hospital-wide implementation of a massive transfusion protocol: before and after

#### J. Luján^1^, P. Villa^1^, B. Llorente^1^, R. Molina^2^, L. Alcázar^1^, C. Arenillas Juanas^1^, S. Rogero^1^, T. Pascual^3^, J.A. Cambronero^1^

##### ^1^Hospital Universitario Príncipe de Asturias, Intensive Care Unit, Madrid, Spain; ^2^Hospital Universitario Príncipe de Asturias, Madrid, Spain; ^3^Hospital Universitario Príncipe de Asturias, Hematology, Madrid, Spain

###### **Correspondence:** J. Luján – Hospital Universitario Príncipe de Asturias, Intensive Care Unit, Madrid, Spain

**Introduction:** Because of the substancial morbidity and mortality provoked by massive bleeding, a protocol to guide treatment of this event in each hospital is required.

**Objectives:** The aim of this study was to determine whether implementation of the massive transfusion protocol (MTP) was associated with a change in clinical practice or mortality.

**Methods:** Ambispective observational study in a Level 3 Hospital. A MTP was developed in collaboration with the Departments of Anesthesia and Hematology. After the approval of the protocol by the Quality committee, training in the protocol was conducted in the hospital. MTP was implanted in November 2013. Only Anesthesia and Intensive Care Departments could activated the protocol if needed. All MTP activations from November 2013 to February 2016 were included in the study (post-MTP group). Post-MTP group was compared with a historical group of patients admitted to hospital as haemorragic shock (pre-MTP group) from July 2011 to October 2013. Data acquired included: demographics characteristics, reason of ICU admission, severity-of-illness by APACHE score, amount of blood and hemostatic products and 24 h and 90-day mortality

**Results:** Pre-MTP group included 21 patients and post-MTP group included 45 patients. Median age of 61,33 ± 15,20 vs 57,44 ± 16,95 (p >0,05).Predominance of males in both groups (66,7 % vs 68,9 %, p > 0,05), respectively. There were no statistically significant differences between groups in antiplatelet and anticoagulation previous treatment ( 38,1 % vs 26,7, p > 0,05). Gastrointestinal bleeding was the most frequent reason for ICU admission in both groups (66,7 % vs 37,8 %, respectively (p > 0,050)).Intergroup comparisons did not revealed statistically significant differences in APACHE (pre-MTP 20 ± 8,2 vs post-MTP 16,5 ± 6,7 p > 0,05) neither in the administration of blood and hemostatic products. The median number of RBC units was 8,67 in the pre-MTP group vs 8,18 in the post-MTP group (p > 0,05), units of FFP 3,76 vs 3,80 (p > 0,05) and units of platelets 0,67 vs 1,22 (p = 0,74), respectively. No statistically significant differences were found in 24 h-mortality (33,3 % vs 24,4 %, p > 0,05) or 90 day-mortality ( 38,1 % vs 33,2 %,p > 0,05).

**Conclusions:** The number of patients is greater in post-MTP group and APACHE score is lower in the same group since we are warned of these patients at an early stage. There were no differences in clinical practice regarding the administration of blood and hemostatic products.No change in mortality could be documented using the protocol. We have not found any statistically differences probably in part due to the sample size.

**References**

1. Experience with a massive transfusion protocol in the management of massive haemorrhage. Sinha R, Roxby D, Bersten A. Transfus Med. 2013 Apr;23(2):108–13.

## ETHICS AND ORGAN DONATION

### 1062 Terminal extubation as a form of withdrawing life-sustaining treatments

#### P. Matía Almudévar, J. Palamidessi Domínguez, S. Alcántara Carmona, D. Palacios Castañeda, A. Naharro Abellán, A. Pérez Lucendo, L. Pérez Pérez, R. Fernández Rivas, N. Martínez Sanz, J. Veganzones Ramos, P. Rodríguez Villamizar

##### Hospital Universitario Puerta de Hierro Majadahonda, Madrid, Spain

###### **Correspondence:** P. Matía Almudévar – Hospital Universitario Puerta de Hierro Majadahonda, Madrid, Spain

**Introduction:** Discontinuation of life sustaining treatments (LST) is an accepted approach for certain ICU patients. There are different ways of limiting LST, and while terminal extubation (TE) is one of them, it may lead to dyspnoea and respiratory distress, which can be regarded as morally troublesome.

**Objectives:** To study TE as a way of withdrawing LST and to focus on sedation and analgesia practices.

**Methods:** Retrospective study (January 2012 to March 2016) including all ICU patients with withdrawal of LST by TE. The decision to limit LST was always made in clinical consensus and accepted by the family. We analysed demographic data, diagnosis on ICU admission, ICU length of stay, reason for withdrawing LST, time from TE to cardiac arrest and the use of sedation and analgesia during the process. Statistical analysis was done using the Chi-square test.

**Results:** Sixty-eight patients, mean age 60,84 ± 15,4 years. Forty males (58,8 %). ICU diagnosis upon admission: 39,7 % (n = 27) cardiac arrest due to cardiac cause, 30,9 % (n = 21) cerebral stroke (ischemic or haemorrhagic); 10,3 % (n = 7) respiratory failure. Mean APACHE II 26,09 ± 6,9.

Median ICU stay was 6 days (3–10). Mean SOFA before withdrawing LST was 8,23 ± 3,3. Main causes for limitation of LST: severe non-reversible neurological impairment (83,8 %, n = 57); refractory hypoxemia (10,3 %, n = 7); multiorgan failure (5,9 %, n = 4). Median time from TE to cardiac arrest: 15 minutes (10 to 45). Eighty per cent of patients (n = 65) died in the first hour and 97 % within 48 hours.

**Fig. 110 (abstract A1062).**
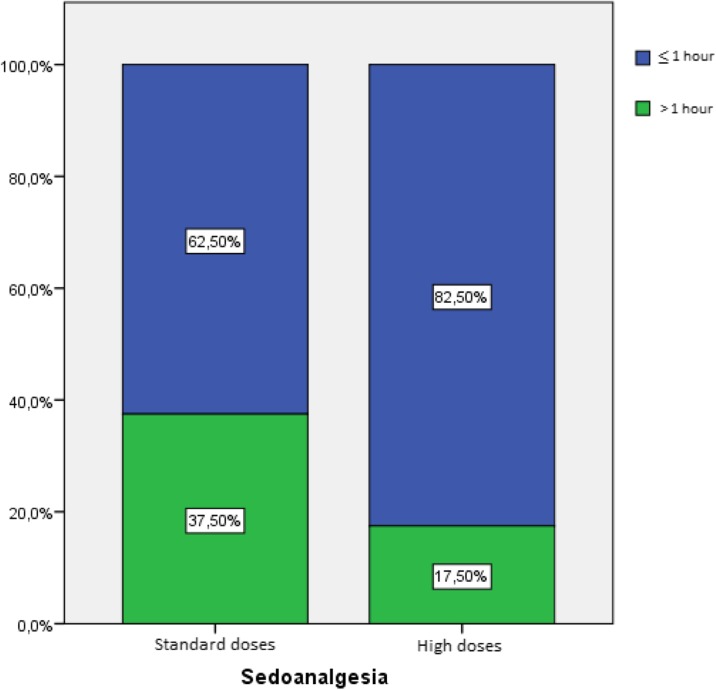
ᅟ

During limitation of LST, analgesic drugs used were Remifentanil (57,1 %), Fentanyl (22,4 %) and Morphine (10,2 %) and sedatives used were Remifentanil (38,8 %), Midazolam (16,3 %), Propofol (6,1 %) or a combination (30,6 %). In 96,9 % of cases (n = 47) sedoanalgesia was increased (bolus administration, increase of the infusion rate or both). High doses of sedoanalgesia, described according the American College of Critical Care recommendations^1^, were used in 83,3 % of cases versus 16,7 % of standard doses. We found that 82,5 % of patients who received high doses of sedoanalgesia died within the first hour vs 62,5 % in the standard dose group (p 0,204). [Fig. 110]

**Conclusions:** TE is a common way of limiting LST in our ICU, being the main reason for it the presence of a severe non-reversible neurological impairment with high APACHE II and SOFA scores.

Increasing sedation and analgesia doses after TE is a standard procedure when withdrawing LST.

We found no differences in the time from TE to cardiac arrest with respect to doses of sedoanalgesia administered. Short times between TE and cardiac arrest regardless of sedative and analgesic treatment used, support the idea that doses needed to palliate patient distress do not hastened the inevitable death.

**References**

1*.* Truog RD et al. Recommendations for end-of-life care in the intensive care unit: a consensus statement by the American College of Critical Care Medicine. *Crit Care Med* 2008; 36(3):953–63

### A1063 Assessing the spiritual health in the intensive care unit nurse

#### S. Javadpour, N. Kalani, T. Amininejad, S. Jamali, S. Sobhanian

##### Jahrom University of Medical Sciences, Jahrom, Islamic Republic of Iran

###### **Correspondence:** S. Javadpour – Jahrom University of Medical Sciences, Jahrom, Islamic Republic of Iran

**Introduction:** Intensive care unit due to critical ill patients and its special situation, require nurses who can take care for patients in their best manner. Spiritual health as one of the important aspects of the health, along with other aspects such as physical, mental and social aspects, has an important role in providing useful and effective care by nurses in intensive care units.

**Objective:** This study was conducted to assess spiritual well-being in the intensive care unit nurses.

**Methods**: In this cross-sectional study, 53 ICU nurses were studied. Census method is done for sampling and study was conducted according to informed consent of intensive care unit nurses of Jahrom University of Medical Sciences in 1394. The data collection tool was a valid and reliable questionnaire of spiritual health (r = 0.8), consisted of 28 questions related to attitude and 20 questions related to the performance. The questionnaire had 5-point Likert scale: absolutely agree (score = 100), almost agree (score = 75 ) , neutral (score = 50), almost disagree (score = 25) and strongly disagree (score = 0). Collected information was analyzed by SPSS software for the descriptive statistics.

**Results:** Of total of 53 nurses, 94.3 % (n = 50) were female, 66 % (n = 35) were married and 98.1 % (52 patients) had nursing bachelor´s degree. The average age of nurses was 32.06 ± 6.23. 49.1 % (26 patients) worked in the ICU and others worked at CCU and dialysis wards. mean score of attitude towards spiritual health was 86.85 and mean score of healthy spiritual performance was 81.53. The total mean score of spiritual health in the population of ICU nurses was 84.19 ± 9.62 of 100

**Conclusion**: The spiritual health score is generally good in the intensive care unit nurses, although due to the difference between spiritual insight and action, special attention should be done to enhance better healthy practice and consequently intensive care unit nurses who are exposed to high stress situation , can benefit the spiritual health and as a result, patients receive better nursing care.

**Grant acknowledgment**

We are appreciate of Medical Ethics Research Center of Jahrom University of Medical Sciences and nurses of all intensive care units.

### A1064 End of life decisions in intensive care units (ICU): the psychological experiences of physicians and nurses

#### A. Laurent^1^, M. Bonnet^1^, R. Rigal^2^, P. Aslanian^2^, P. Hebert^2^, G. Capellier^3^, PS-ICU Group

##### ^1^University of Franche-Comté, Laboratory of Psychology EA 3188, Besançon, France; ^2^CR CHUM, Montreal, Canada; ^3^CHRU Minjoz, Besançon, France

###### **Correspondence:** A. Laurent – University of Franche-Comté, Laboratory of Psychology EA 3188, Besançon, France

**Introduction:** End of life decisions are common issues in Intensive Care Units (ICUs), but literature reviews show that such decisions are a source of conflict and suffering within the medical team (H.I Jensen & al., 2011).

**Objective:** The aim of this study is to identify the psychological experiences of the physicians and nurses confronted with end of life decisions in intensive care units (ICU).

**Design.** Twenty physicians and twenty nurses who work in three ICUs in Montreal (Canada) were interviewed about their approach to withholding or withdrawing life-sustaining medical treatment. All of the interviews were transcribed verbatim and analysed thematically using Interpretative Phenomenological Analysis.

**Setting.** 3 ICUs in the teaching hospitals of Montreal (Canada).

**Results:** End of life decisions are source of psychological stress and uncertainty. For nurses and physicians, their psychological experiences in relation to end of life decisions are very different.

Physicians are responsible for decision-making and they do not base their decisions solely on patient data. They depend on more subjective dimensions such as perceptions, beliefs and culture concerning care, dying and death for both care-givers and relatives.

Nurses suffer from the feelings caused by medical obstinacy. These feelings are a source of psychological stress as they create a sensation of inflicting pain on patients and lead to a lack of understanding of the care required.

**Conclusions:** Collective decisions concerning the end of life decisions are a challenge. An important challenge to quality of care and make ethical decision in this emotional time.

### A1065 Limitation of treatment, what is the opinion of professional?

#### M.R. Diaz Contreras, C. Rodriguez Mejías, F.C. Santiago Ruiz, M. Duro Lombardo, J. Castaño Perez, E. Aguayo de Hoyos

##### Complejo Hospitalario de Granada, Granada, Spain

###### **Correspondence:** C. Rodriguez Mejías – Complejo Hospitalario de Granada, Granada, Spain

**Introduction and objectives:** By limitation of treatment (LET ), we understand clinical decision not start or withdraw life support when a perceived disproportion between the means and therapeutic purposes to be achieved. In our units, we often find patients versus those who have to make a decision LET,recognizing many professionals do not have clear ideas on this and other aspects of clinical ethics.Our objetives is to know the opinion of the medical staff working in the intensive care unit (ICU) of our center, about ethical problems generated around the care of critically ill patients,especially the LET.

**Material and methods:** This survey consisting of 14 questions aimed at all health personnel working in our ICU and answered voluntarily and anonymously. A descriptive study of answered surveys are conducted.

**Results:** 92 surveys are answered, 77 % are women, >45 years 68.5 % and 83.7 % more than 9 years of professional experiencie. Among the professional category: 24 % Doctors, 50 % Nurses and 26 % Nursing Assistant.All but two are in favor of the application of LET, although 79.3 % believe it is not the same not start to withdraw treatment, 13 % related to the passive euthanasia and 20.6 % think that not establishment is passive and withdrawal is active. Only 20.6 % think it is the same not start a treatment that remove. And 5.4 % equates terminal sedation with euthanasia.To highlight that 14.1 % would not take into account the views of the family when taking LET, there unanimously to consider advance directives of the patient. 35.8 % think that the LET should be taken by consensus of the medical unit, 29.3 % by multidisciplinary consensus among all professionals involved in patient care. Almost everyone thinks that if there are discrepancies most important will be the living will of the patient. Despite the importance given, only 8.7 % admit having made a living will.54.3 % of staff do not feel qualified to make a decision LET: 58.4 % nurses, 31.2 % nursing assitan and 10.4 % medical residents. Virtually everyone thinks often falls into the aggressive treatment,even with the best intentions.83.7 % think that LET is not adequately captured in the patient´s history and 71.7 % are not well transmitted to all personnel involved in patient care.

**Conclusions:** While most professionals do not have a clear definition of LET, all of them are in favor of its implementation. When taking such decisions it is important that there is a consensus of the medical unit and in general the multidisciplinary team caring for the patient. Always the living will of the patient must be considered.The decision LET should be adequately reflected in the patient´s history and be transmitted to all professionals involvedIt.

Is important to train professionals from the ethical and moral point of view, since such decisions affect the right to life and a dignified death

**Table 72 (abstract A1065).** LET Survey For Health Professionals UCI

A-Sex: Male/Female B-Age: Under 25 years old/25 to 35 years old/35 to 45 years/More than 45 years C-Professional Category:Doctor/Nurse/Nursing Assistant D-Working years:0-3/3-6/6-9/More than 9.
1-Do you know what the limitation of treatment(LET)?Yes/No 2-For you it is the LET?Active Euthanasia/Passive Euthanasia/The establishment is not passive and active withdrawal/None of the above 3-Are you in favor of the implementation of the LET?Yes/No 4-Do you think it is the same not start a treatment to remove a treatment already in place? Yes/No 5-You consider that palliative or terminal sedation is similar to euthanasia? Yes/No 6-Should take into account the views of the family to implement the LET? Yes/No 7-Should take into account the vital testament when implementing the LET? Yes/No 8-When applying the LET, who do you think should make the decision?The physician responsible for the patient/The staff responsible for the patient (doctor,nurse,assistant..)/Consensus medical unit/Consensus multidisciplinary medical team(as appropriate: intensivitas with cardiac surgeons , hematologists , neurosurgeons, etc.)/After consulting the team of hospital ethics/The patient´s family 9-In case of discrepancies,which opinion must prevail?The responsible physician/Family/Vital testament 10-Do you think that when a decision is taken this appears LET adequately reflected in the patient´s history? Yes/No 11-The decision LET are properly transmmitted to all personnel involved in patient care?Yes/No 12-Do you feel prepared to make a decision about LET?Yes/No 13-Do you think that sometimes falls, even with the best intentions, therapeutic cruelty, needlessly prolonging situations of suffering, both for the patient and his family?Yes/No 14-Do you have a vital testament:Yes/No

### A1066 Refusal of admission to an intensive care unit

#### A. Estella^1^, R. Viciana^2^, L. Perez Fontaiña^1^, T. Rico^1^, V. Perez Madueño^1^, M. Recuerda^1^, L. Fernández^1^

##### ^1^Hospital del SAS de Jerez, Intensive Care Unit, Jerez de la Frontera, Spain; ^2^Hospital del SAS de Jerez, Jerez de la Frontera, Spain

###### **Correspondence:** A. Estella – Hospital del SAS de Jerez, Intensive Care Unit, Jerez de la Frontera, Spain

**Introduction:** Most of studies about limitation of life sustaining therapy not includes decisions of denial of admission in ICU.

**Objectives:** The aim of the present study was:• To identify cases of limitation of life-sustaining treatments (LST) regarding denial of ICU admission.• To compare the clinical characteristics and prognosis of patients not admitted in ICU according the reason for denial admission: non invasive treatment recommendation against LST decisions.

**Methods: DESIGN.** Prospective observational study in a community hospital.Inclusion criteria: patients consulted to intensivist for ICU admission that was denied. Time of study: 6 months.Variables: age, gender, comorbidity Charlson index, APACHE II score, destination and prognosis.Statistic analysis. The data were analyzed using SPSS version 18 for Windows, chi-square test was used to compare qualitative variables and Student t for quantitative variables.

**Results:** 546 patients were admitted in ICU during time of study. In 54 patients ICU admission was refused. 31.5 % of these decisions were due LST. Main reasons for consultation were respiratory failure, 18.5 %, coronary syndrome, 18.5 %, and infectious disease, 13 %. Table 74 shows the characteristics of the patients included.

**Conclusions:** Denial of admission to an intensive care unit due to LST decisions was associated with a high morbidity and mortality.

Mortality, APACHE II and Charlson index were significantly lower in the group of patients refused admission to an ICU with a non invasive treatment recommendation.

**Table 73 (abstract A1066).** Clinical characteristics of patients

	LST (n:17)	Non invasive treatment (n:37)	p value
Age, median	78	67	ns
Sex (male%/female%)	58,8/41,2	70,3/29,7	ns
APACHE II, median	25	12	<0.05
Origin prior ICU Emergency Medical ward Surgical ward Oncohematology	58,8 % 29,4 % 0 % 5,9 %	78,4 % 16,2 % 2,7 % 2,7 %	<0.05
Mortality (%)	70,6	10,8	<0.05
Charlson Index <3 3–5 > 5	5,9 % 5,9 % 88,2 %	29,7 % 32,4 % 37,8 %	<0.05

### A1067 Searching for organ donors outside the icu. A multidisciplinar collaborative model using new technologies in a university hospital

#### A. Sandiumenge^1^, S. Bonet^2^, C. Mazo^3^, M. Rubiera^2^, J.C. Ruiz-Rodríguez^3^, R.M. Gracia^3^, E. Espinel^1^, T. Pont^1^

##### ^1^University Hospital Vall d´Hebron, Transplant Coordination, Barcelona, Spain; ^2^University Hospital Vall d´Hebron, Stroke Unit, Neurology, Barcelona, Spain; ^3^University Hospital Vall d´Hebron, Barcelona, Spain

###### **Correspondence:** A. Sandiumenge – University Hospital Vall d´Hebron, Transplant Coordination, Barcelona, Spain

**Introduction:** Despite an optimized donor detection system set in place in most Spanish hospitals, 44 % of medically suitable possible organ donors outside the ICU are not being referred by the treating physician.[i]

**Objectives:** Descriptive analysis of the implementation of a web-based collaborative system for the identification and management of all possible organ donors outside the ICU at a University Hospital.

**Material and methods:** In 2015 a virtual collaborative system using a cross-platform instant messaging application(Whatsapp®) between neurologists, ICU specialists and donor coordinators(DC) was started. This system was aimed at the early referral of patients suffering from a devastating neurological injury (GCS < 8; NIHSS > 21; ICH > 3; post-anoxic encephalopathy-PAE), in whom all treatment options, including ICU admission, had been deemed futile by the attending team. Once DC established medical suitability and likelihood of progression to brain death (BD), patients (or their relatives) were offered the option to undergo non-therapeutic elective ventilation (NTEV) and ICU admission, to incorporate donation into their end-of life care plan.

**Results:** A total of 46 patients (72.2 ± 11.1; range 46–90)years old; 70 % male) were referred (Mar-Dec 2015) from the stroke (58.6 %), emergency (26.9 %) and coronary (4.3 %) departments after suffering hemorrhagic (58.6 %), ischemic (32.6 %) stroke, subarachnoid hemorrhage (4.3 %) or PAE (4.3 %). GCS at referral was 5.2 ± 3.1 with ICH, NIHSS and Hunt-Hess scores of 3.9 ± 1.0, 26.4 ± 4.2, and 4.5 ± 0.4, respectively. Twenty patients (43.4 %) were discarded as donors due to advanced age (66.6 %), co-morbidities (86.6 %),clinical deterioration (13.3 %) and/or GCS improvement (25.0 %). Sixteen families (61.5 %) accepted the option of organ donation, proceeding to patient NTEV 62.5 %(n = 10) and ICU admission 93.7 %(n = 15). Ten(66.6 %) patients progressed to BD and 9 of them became actual donors(AD). As previously agreed with the family, withdrawal of life-sustaining treatment was applied at a mean of 76.5 ± 21.2 hours following ICU admission in those who did not progress to BD (n = 5), 4 of them becoming actual donors after controlled circulatory death. A total of 14 donors resulted from this collaboration system, accounting for 29.7 % of all AD (47) at the Hospital.

**Conclusions:** The implementation of a virtual community allowed more patients to be presented with the option of donation upon their death leading to the generation of one out of 4 AD in our centre. The utilization of new technologies contributed to early referral of possible donors outside the ICU, promoting collective learning through the provision of immediate feedback while preserving patient confidentiality.

**References**

1. End-of-life care practices in patients dead as a result of a devastating brain injury and organ donation in Spain. Report from the ACCORD-Spain Project. Available at: http://www.ont.es/infesp/Paginas/ProyectosenMarcha.aspx. Last access: April 2016.

### A1068 An observational study on time to death after withdrawal of treatment in potential donors after circulatory death maastricht category III (DCD III)

#### A. Kotsopoulos^1^, N. Jansen^2^, W.F. Abdo^3^

##### ^1^St Elisabeth Twee Steden Hospital, Intensive Care Medicine, Tilburg, Netherlands; ^2^The Dutch Transplant Foundation, Leiden, Netherlands; ^3^Radboud University Nijmegen Medical Centre, Nijmegen, Netherlands

###### **Correspondence:** A. Kotsopoulos – St Elisabeth Twee Steden Hospital, Intensive Care Medicine, Tilburg, Netherlands

**Introduction:** Donation after circulatory death (DCD), refers to the procurement of organs from patients whose death is diagnosed and confirmed after circulatory arrest. In the Netherlands the timeframe for DCD to proceed is set at two hours. A considerable number of potential donors after circulatory death are lost because they do not die within the specified timeframe after withdrawal of life-supporting treatment (WLST). Identification of those dying within 2 hours after WSLT results in efficient utilization of the organ procurement teams, hospital resources and above all fulfillment of family expectations.

**Objectives:** The aim of this study is to determine factors predicting time to cardiac circulatory death after WSLT within 2 hours.

**Methods:** In this single-center study we retrospectively evaluated 92 potential and actual DCD III donors. Patients younger than 16 years of age, and clinically brain dead patients in whom relatives requested a DCD procedure, were excluded. Univariate logistic regression analyses were performed to establish the effect of different predictors.

**Results:** Only 20 (32 %) converted into actual donor partly due to the fact that cardiac death did not occur within 2 hours. Univariate analysis showed an association between the following predicitors and death within 2 hours: absent cough (OR 39.27; 95 % CI 4.95-311.07; p = 0.001), absent corneal (OR 9.214; 95 % CI 3.05-27.77; p < 0.001), and absent pupillary reflex (OR 4.22; 95 % CI 1.64-10.83; p = 0.003). An extensor or absent motor response and a GCS of 3 were not significant predictors. Overall the ventilatory settings were low however the FiO_2,_ peak inspiratory pressures and use of a controlled mode of ventilation were significantly higher in the ≤2 hours group. Significantly less patients in the ≤2 hours group used sedatives (OR 0.078; 95 % CI 0.010-0.616; p = 0.016). The proportion of patients using vasopressors was significantly larger in patients dying within 2 hours (OR 4.28; 95 % CI 1.16-15.8;p = 0.029). Multivariate logistic regression analysis showed that absent cough reflex was the only significantly contributing predictor for death within 2 hours (OR 17.7; 95 % CI 1.62-192.7), p = 0.018). Post WSLT nursing (e.g. use of suction support, May-tube) did not differ between the 2 groups.

**Conclusions:** An absent cough reflex was the single strongest predictor of death within 2 hours. Interestingly patients dying within 2 hours had less sedatives after WLST. Currently, we are performing a prospective multi-center study to develop a multimodal prediction model for bedside use.

**References**

1. A. Rabinstein A, et al. Prediction of potential for organ donation after cardiac death in patients in neurocritical state: a prospective observational study. Lancet Neurol. 2012;3(5):414–419.

**Grant acknowledgment**

NE Jansen, WF Abdo

**Table 74 (abstract A1068).** Logistic Regression Predicting Time to Death

Variable	B	p	exp(B)	Odds Ratio Lower bound	Odds Ratio Upper bound
Absent corneal reflex	1.385	0.113	3.993	0.722	22.092
Absent cough reflex	2.874	0.018	17.70	1.626	192.7
Absent pupillary reflex	0.310	0.691	1.364	0.296	6.287
Motor response (absent or extensor)	0.053	0.965	1.054	0.104	10.65
GCS of 3	0.141	0.899	1.152	0.131	10.13
Controlled mode of ventilation	−0.560	0.517	0.571	0.105	3.114
FiO2	0.340	0.926	1.405	0.001	1844
Peak inspiratory pressure	0.070	0.480	1.073	0.883	1.303
Oxygenation indexa	0.054	0.775	1.056	0.728	1.532

### A1069 Family refusal of organ donation in Clinical Hospital Centre Sestre Milosrdnice, Zagreb, Croatia, from 2005 to 2015

#### A. Gopcevic, Z. Gavranovic, M. Vucic, M. Zlatic Glogoski, L. Videc Penavic, A. Horvat

##### Clinical Hospital Center Sestre Milosrdnice, Anaesthesiology and Intensive Care, Zagreb, Croatia

###### **Correspondence:** Z. Gavranovic – Clinical Hospital Center Sestre Milosrdnice, Anaesthesiology and Intensive Care, Zagreb, Croatia

**Introduction:** Family refusal of organ donation from DBD (donors after brain death) is a limiting factor of the whole donation process and plays an important role in shortage of organs available for transplantation. Although Croatia is a state with presumed consent when it comes to DBD (donors after brain death) organ donation, family is always informed about the possibility of organ donation after it is verified that the deceased is not registered in the non-donor registry. If the family objects organ donation, their decision is always respected.

**Objectives:** To retrospectively analyse rate and reasons of family refusal of organ donation in our hospital in last 10 years.

**Methods:** We retrospectively evaluated data from 255 brain dead persons in our hospital from 2005 to 2015.

**Results:** Out of 255 confirmed brain deaths, organ donation was performed in 193 cases, while in 5 cases there was a medical contraindication for organ donation. Family opposed the organ donation in 57 cases (22 %). Main reasons for refusal were: donors´ unknown wishes or opposition to organ donation in 43 cases (75 %), fear of objection from other members of the family in 8 cases (14 %) and religious beliefs in 6 cases (10 %). There were no refusals due to fear of organ trafficking or disfiguring of the body. We also examined impact of additional education of transplant coordinators on refusal rate. From 2010 it is mandatory that our coordinators regularly participate in at least two educational seminars per year, and the refusal rate in period 2010–2015 was 22 %, while in period 2005–2010 was 21.75 % (p = NS).

**Conclusions:** Main reason for refusal of organ donation in our hospital is unknown wish or opposition of the deceased person. No family refused donation due to fear of organ trafficking which is an encouraging fact. Although refusal rate in our hospital is 22 %, which is higher than Croatian average of 13 %, we could not clearly identify contributing factors. We also could not confirm the hypothesis that additional education of transplant coordinators lowers refusal rate. A more detailed prospective evaluation is needed in order to further reduce refusal rate in our hospital.

**References**

1. Ghorbani F, Khoddami-Vishteh HR, Ghobadi O, Shafaghi S, Louyeh AR, Najafizadeh K. Causes of family refusal for organ donation. Transplant Proc 2011;43:405–6.

2. Zivcic SZ, Busic M, Zupan Z, Pelcic G, Anusic MJ, Jurcic Z, et al. Development of the Croatian model of organ donation and transplantation. Croat Med J 2013;54:65–70.

3. Santiago C, Gomez P, Olivares J, de la Concepcion M. Evaluation of organ procurement in an area under the influence of a training program. Transplant Proc 2005;37:3649–3650.

4. Siminoff LA, Marshall HM, Dumenci L, Bowen G, Swaminathan A, Gordon N. Communicating effectively about donation: an educational intervention to increase consent to donation. Prog Transplant 2009;19:35–43.

**Grant acknowledgment**

None.

### A1070 Non-heart beating donor program: Seville's experience

#### L. Martin-Villen^1,2^, J.J. Egea-Guerero^1,2^, J. Revuelto-Rey^3^, T. Aldabo-Pallas^2^, E. Correa-Chamorro^1^, A.I. Gallego-Corpa^1^, P. Ruiz del Portal-Ruiz Granados^1^

##### ^1^Hospital Universitario Virgen del Rocio, Coordinación de Trasplantes, Seville, Spain; ^2^Hospital Universitario Virgen del Rocio, Critical Care, Seville, Spain; ^3^Hospital Universitario Juan XXII, Critical Care, Tarragona, Spain

###### **Correspondence:** L. Martin-Villen – Hospital Universitario Virgen del Rocio, Coordinación de Trasplantes, Seville, Spain

**Introduction:** Although Spain has the highest rate of donors in the world^1^, it is still insufficient. For this reason, the development of Non-Heart-Beating Donation (NHBD) programs has been one of the measures developed from health institutions in order to meet those needs of organs.

**Objectives:** To evaluate the results of the NHBD program developed in a city of 800,000 inhabitants since it began.

**Material.** Prospective observational study between 2012 and 2015. We included all patients with out-of-hospital cardiac arrest who did not recovered after advanced cardiopulmonary reanimation. When clinical criteria were met those patients were transferred to the hospital as potential donors (PD). The variables analyzed were: total number of queries activation, of PD, of eligible donors (ED) and of real donors (RD). RD attendance times were registered and we defined out-of-hospital time (from Cardiac arrest to hospital arrival), in-of-hospital time (from hospital arrival to cannulation onset), cannulation time (beginning of cannulation to perfusion onset) and perfusion (from perfusion onset to the first organ removal). We registered number and type of valid organs and tissues, number of family members or judicial negative, number of non-real-donors (NRD) and its causes.

Descriptive statistical analysis was performed, presenting the qualitative variables as frequencies and percentages, and quantitative as mean (+/− SD) or median (interquartile range ICR) depending its distribution.

**Results:** In the analysis period 113 queries activation were performed, 55 (48 %) were PD, of which 37 (67 %) were ED and 27 (49 %) RD. RD donated 63 organs of which 40 (63.5 %) were valid for transplantation. Mean rate of organ per donor was 1.48 (+/− 0.27). 88 % of RD were men with a mean age of 40.7 (+/− 11.3). Of the RD, 22 (82 %) were renal donors, 2 (7.2 %) liver donors and 18 (66 %) of any type of tissue (corneal 66.6 %, 66.6 % bones, skin 11.1 % and vascular segments 11.1 %). Regarding attendance times, the median time was 71 (ICR 60–75) minutes for out-of-hospital, 29 (ICR 22–33) minutes for in-of-hospital, 30 (24–35) minutes for cannulation and 135 (107–169)minutes for perfusion. There were 28 NRD, 11 (39.2 %) were for recovery pulse upon arrival, 7 (25 %) for not meeting criteria for starting cannulation, 7 (25 %) perfusion problems and 3 (10.7 %) per family negative. There was no judicial negative. Of these 28 NRD, 7 (25 %) could be tissues donors.

**Conclusions:** NHBD is an effective program with a ratio of 1.5 organs/donor.

NHBD program could help to serve organ and tissues demand.

NHBD program has a low rate of family refusal and null judicial.

**References**

1. Organización Nacional de Trasplantes. Ministerio de Sanidad, Servicios Sociales e Igualdad [Internet]. Madrid, Spain: [Ref 12 Jan 2015]: http://www.ont.es/Documents/Balance_Actividad_2015.pdf

### A1071 Impact of brain injury (BI) and BI with brain death (BD) on circulating immune cells phenotype; role of plasma factors

#### V. Faivre^1^, L. Wildenberg^1^, B. Huot^1,2^, A.C. Lukaszewicz^1,2^, M. Simsir^1^, C. Mengelle^1^, D. Payen^1,2^

##### ^1^Université Paris Diderot, Sorbonne Paris Cité, INSERM UMR1160, Paris, France; ^2^APHP, Lariboisiere University Hospital, Surgical ICU, Paris, France

###### **Correspondence:** V. Faivre – Université Paris Diderot, Sorbonne Paris Cité, INSERM UMR1160, Paris, France

**Introduction:** Renal grafts from BD donors have both reduced lifetime and function. It may result from BD-induced inflammation with graft infiltration by Monocytes (Mo), T Lymphocytes (Ly) and neutrophils (PMN).

**Objectives:** 1- Does BI more than BD induces modifications of the donor immune cell phenotype? 2- Are these modifications resulting from plasma (Pl) micro-environment?

**Methods:** Blood from patients with severe BI (Glasgow < 8) collected during the BD process or at the same delay for BI alone. Data of BI and BD groups compared to healthy volunteers (HV, n = 21): a/White Blood Cells (WBC), PMN and Ly count; b/Expression of immune activation markers (flow cytometry): HLA-DR, CD11b and CD62L (adhesion molecules), CCR2 (chemokine receptor (CR)) on Mo CD16- (classical, infiltrating) and Mo CD16+ (non-classical, patrolling); on PMNs: CD11b, CD62L and CCR2; on CD4 (T4) and CD8 (T8): CTLA-4 and LAG-3 (T Ly anergy markers) c/Spontaneous and stimulated Reactive Oxygen Species (ROS) production (chemiluminescence).

Pl factors: 1- *ex vivo* incubation (6 h) of HV blood cells with pooled Pl from BD and BI patients, vs control Pl pool (Plc, n = 5 HV) collecting the same parameters than in patients, in addition with CXCR4 (CR) and PD-1 (Ly anergy); 2- blood cells from BD patients incubated with Plc (removing patients Pl). Non parametric statistical tests.

Ethical committee: Société de Réanimation de Langue Française - CE n° 13–19.

**Results:** 23 patients (Glasgow 4 [3–7]), 9 BD, 14 BI enrolled. *Compared to HV*, WBC count increased in BI and BD for PMN and Mo, with stable fraction of Mo16+ and 16-. *Activation markers expression were similar in BI and BD patients*: decreased on Mo16- (HLA-DR and CCR2), decreased on PMN (CD62L, CCR2) but no change for CD11b; increased in Mo16+ (CD62L and CD11b) with a decreased in CCR2. On T4 and T8, only BD had a decreased CTLA-4 vs HV. *Spontaneous ROS* increased in BD vs both HV and BI (p < 0,05). The other parameters did not differ between patients groups.

*Plasma environment changes: Pl from patients* induced changes in HV cells phenotype similar to those observed in patients' cells: both BI and BD Pl decreased HLA-DR on Mo16- and 16+, and CCR2 on Mo16-; PMNs CD11b and CCR2 decreased with BI Pl but not with BD Pl, with decreased T4 PD-1 expression. *BD cells incubated in Plc:* increased CD11b and PD-1 expression on Mo16- and 16+; decreased CD62L on Mo16- and PMN, and PD-1 on T8. *Incubation itself* without Pl replacement, increased expression of CXCR4 in Mo16-, Mo16+ and PMN. HLA-DR, CCR2 and ROS remained unchanged in all experimental conditions.

**Conclusions:** More than BD event itself, the initial BI seems to induce the essential of immune phenotype changes leading to innate immunosuppression. Part of these changes depends on Pl micro-environment factors, suggesting a potential benefit to early use extracorporeal adsorption methods.

**Grant acknowledgment**

Ministère de l´Enseignement Supérieur et de la Recherche

### A1072 Promoting controlled donation after circulatory death in Spain through high-fidelity simulation training

#### N. Martinez Sanz, B. Lobo Valbuena, M. Valdivia de la Fuente, P. Matía Almudena, L. Pérez Pérez, S. Alcántara Carmona, A. Navarro Abellán, I. Fernández Simón, J.J. Rubio Muñoz

##### Hospital Universitario Puerta de Hierro Majadahonda, Intensive Care Unit, Madrid, Spain

###### **Correspondence:** N. Martinez Sanz – Hospital Universitario Puerta de Hierro Majadahonda, Intensive Care Unit, Madrid, Spain

**Introduction:** In Spain the ethical framework for the practice of controlled donation after circulatory death (cDCD) was created in 2012. Over the past three years the evolution of cDCD has been astounding, increasing from 23 cDCD donors in 2012 to 211 in 2015, helping to raise Spanish donor activity.

Training is one of the strategies to promote this kind of donation. It is well known that high-fidelity simulation is becoming a useful tool to improve the training of health professionals.

Objective: Analyze the interest and evaluation of a cDCD training course, based on high-fidelity simulation, and its impact to promote cDCD in Spain

**Methods:** Descriptive study analyzing the 17 editions of the course performed at the Simulation Unit of Puerta de Hierro Hospital in Majadahonda in 2013, 2014 and 2015.

The course was aimed to Spanish health professionals (nurses and doctors), with a total duration of 10.5 hours. It consisted of a small theoretical introduction followed by several workshops, which included: donor after circulatory death management protocol through high fidelity simulator, family interview, preservation and perfusion procedures with extracorporeal membrane oxygenation in animal models.

The students sat a pre-course self-assessment 10-question test to evaluate their knowledge about cDCD. At the end of the course they filled out a survey, offering their opinion on different sections: content, usefulness, documentation and educational support, organization, duration and overall assessment. The score ranged between one, the most negative value, to 10. Average score was analyzed.

A survey was sent to students working in different hospitals to evaluate the impact of the course in the cDCD programs at their hospitals.

**Results:** 366 students completed the course, their characteristics are in Table 76. 364 students did the pre-course test, with an average score of 8.64 points. 364 filled out the survey, results plotted in Table 77.

Feedback trough the after-course survey was received from 56 students. 48 % of the students worked at hospitals without a cDCD program, established after completing the course. 89 % of these students considered that the course contributed to the development and implementation of cDCD program. All professionals who worked at centers where there was already a cDCD program felt that the course contributed to its improvement.

**Conclusions:** Despite the fact that there was a high knowledge on the subject among the students, they showed interest and enjoyed the course.

The course had a high impact because it helped improving and developing cDCD programs in several hospitals.

We believe that this course, based on high-fidelity simulaton training, has been one of the factors that has promoted controlled donation after circulatory death in Spain in the last years

**References**

1. Hessheimer AJ, Domínguez-Gil B, Fondevila C, Matesanz R. Controlled donation after circulatory determination of death in Spain. Am J Transplant. 2016 Feb 29.

**Table 75 (abstract A1072).** Workplace and professional position of the student

	Puerta de Hierro de Majadahonda Hospital	Others Hospitals (>40) and National Transplant Organization
Doctors	48	143
Nurses	90	85
STUDENTS 366 (92 worked at National Transplant Organization)

**Table 76 (abstract A1072).** Survey results valuation

Sections to evaluate	Average score
Course content	9.03 points
Usefulness of the course	9.02 points
Documentation and educational support	9.02 points
Organization	8.7 points
Duration	Well adjusted (94.5 %)
Overall Assessment	9.07 points

### A1073 Advance age lung donors. The new frontier for reducing lung shortage?

#### J. Veganzones Ramos, S. Alcantara Carmona, P. Matia Almudevar, A. Naharro Abellan, M.A. Perez Lucendo, L. Perez Perez, J. Palamidessi Dominguez, R. Fernandez Rivas, P. Rodriguez Villamizar

##### Puerta de Hierro Hospital, Intensive Care Unit, Majadahonda, Spain

###### **Correspondence:** J. Veganzones Ramos – Puerta de Hierro Hospital, Intensive Care Unit, Majadahonda, Spain

**Introduction:** The demand for donor lungs thoroughly exceeds the supply.This situation and the application of a strict group of selection criteria, has made donor lung shortage a major problem. To overcome this scarcity, some studies have examined the possibility of using lungs from older donors with mixed results^1–4^

**Objective:** Using as a starting point the favourable clinical evolution of a recipient of a 85 year old lung, we reviewed all of our donors from the last 6 years that were dismissed strictly because advance age (>60 years)

**Methods:** Retrospective (Feb 10-Jan 16) and descriptive study. All donors of a Spanish tertiary hospital were analysed. We selected all patients excluded for donation and reviewed their contraindication to serve as lung donors. Demographic data and comorbidities, reason for ICU admission, ICU length of stay, respiratory parameters, days of mechanical ventilation, respiratory cultures and antimicrobial therapy were collected.

**Results:** During the period studied we identified 133 potential donors that translated into 90 real donors and only 12 of them (13,3 %) served as lung donors. After analysing the 78 (86,6 %) patients that were not considered as lung donors, we identified 5 (5,5 %) that had been dismissed strictly because advance age.

**Fig. 111 (abstract A1073).**
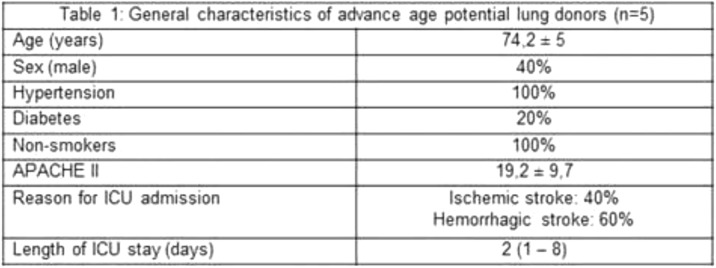
General characteristics of advance age potential lung donors (n = 5)

All 5 patients studied were brain death donors. Demographic data and comorbidities, reason for ICU admission and ICU length of stay are detailed on Figure 111.Last mean pO_2_/FiO_2_ recorded was 325,2 ± 115 (PEEP 5,2 ± 0,44) and last mean pCO_2_ recorded was 44,3 ± 10,7 mmHg. Median days of mechanical ventilation were 2 (1–8). All patients were on antibiotics, 4 of them as part of a selective digestive decontamination program. Three of them had respiratory cultures taken prior to death declaration and in all of them microbiological results were negative

Taking into account this 6-year period, these 5 advance age potential donors would have meant almost a 50 % increase in the lung pool

**Conclusion:** Patients dismissed as lung donors strictly because of advance age had a short length of ICU stay, a short period of mechanical ventilation, pO_2_/FiO_2_ rates >300 and no evidence of respiratory tract infection.

Although the sample is small, we believe that, according to the data obtained, it would be reasonable at least to take into consideration advance age donors, as this would mean a substantial increase in the lung donor pool

**References**

1. Bittle GJ et al.The use of lung donors older than 55 years: a review of the United Network of Organ Sharing database.J Heart Lung Transplant 2013;32:760 2.Aigner C et al.Extended donor criteria for lung transplantation-a clinical reality.Eur J Cardiothorac Surg 2005;27:757

3. Pierre AF et al.Marginal donor lungs: a reassessment.J Thorac Cardiovasc Surg 2002;123:421.

4. Bhorade SM,Vigneswaran W,McCabe MA,Garrity ER.Liberalization of donor criteria may expand the donor pool without adverse consequence in lung transplantation.J Heart Lung Transplant 2000;19:1199

### A1074 Knowledge and attitudes of critical care providers towards human organ transplant in Singapore

#### S. Wee^1^, C. Ong^1^, Y.H. Lau^1^, Y. Wong^2^

##### ^1^Tan Tock Seng Hospital, National Healthcare Group, Anaesthesiology, Pain and Intensive Care, Singapore, Singapore; ^2^Tan Tock Seng Hospital, National Healthcare Group, Singapore, Singapore

###### **Correspondence:** S. Wee – Tan Tock Seng Hospital, National Healthcare Group, Anaesthesiology, Pain and Intensive Care, Singapore, Singapore

**Introduction:** Organ transplantation is the treatment of choice for end-stage organ failure. Despite having the Human Organ Transplant Act (HOTA) in Singapore, an opt-out organ donation scheme that applies to all citizens and permanent resident, there is still a critical shortage of deceased organ donors.

**Objectives:** This study aims to determine the knowledge and attitudes, pertaining to organ donation, of personnel in intensive care units in Singapore and to identify key factors that should be addressed to improve their competency.

**Methods:** A cross sectional study, involving personnel working in the intensive care units in a single tertiary hospital, was conducted. An anonymous-interviewee-administered questionnaire, with both closed and open-ended questions, was administered in English. The questionnaire comprises 24 questions.

**Results:** 172 (90.5 %) out of the 190 distributed questionnaires were completed with equal proportion (43.0 %) of doctors and nurses. 60.5 % had ≥2 years experience in the intensive care unit. 41 (23.8 %) have no religious beliefs. Compared to non-doctors, doctors had better knowledge about the legislation determining patient eligibility for organ donation under HOTA (*p* < 0.05) and were more familiar with clinical and supplementary tests done for brain death certification (*p* < 0.001). 93.0 % had a positive attitude towards organ donation and 92.4 % supported the donation of their own organs under HOTA.

124 (72.9 %) expressed a willingness to play a role in counselling families of potential organ donors; however, only 50 (29.1 %) expressed confidence in doing so. Those who were not confident in counselling cited unfamiliarity with the details surrounding the process of organ donation and brain death certification (n = 81, 66.4 %), inadequate medical knowledge (n = 50; 41.0 %) and lack of appropriate skills in counselling distressed family members (n = 60; 49.2 %). 110 (64.0 %) respondents felt the need for annual or biennial refresher courses.

**Conclusions:** Increasing organ donation rates is a challenging process. However, it is evident that majority of healthcare workers surveyed do not have adequate working knowledge about it. Providing healthcare workers more confidence to handle difficult conversations revolving around organ donation may hopefully increase the number of actualised donors in Singapore.

**References**

1. The Human Organ Transplant Act. Legislative Acts and Guidelines, Ministry of Health, Singapore.

2. Shum E, Chern A. Amendment of the Human Organ Transplant Act. Ann Acad Med Singapore 2006;35:428–32

**Grant acknowledgment**

Nil

### A1075 Organ transplantation from type iii maastricht death cardiac donors: our experience

#### M.E. Banderas-Bravo, V. Olea-Jiménez, J.M. Mora-Ordóñez, C. Gómez-Jiménez, J.L. Muñoz-Muñoz, J. Vallejo-Báez, D. Daga-Ruiz, M. Lebrón-Gallardo

##### Regional University Hospital in Málaga, Intensive Care Medicine, Málaga, Spain

###### **Correspondence:** V. Olea-Jiménez – Regional University Hospital in Málaga, Intensive Care Medicine, Málaga, Spain

**Introduction:** Organ transplants from deceased donors in cardiac arrest are becoming more numerous. In Spain were initially of uncontrolled donors (Maastricht II) but in 2015 the donors died after limiting life-sustaining treatments (LIST) defined as Maastricht III controlled donors, have outnumbered the type II.

**Objectives:** To describe the main features of the Maastricht III donation in our Hospital and the evolution of liver receptors from these donors in cardiac arrest.

**Methods:** Retrospective study of Maastricht III controlled donors in tertiary hospital from Málaga, since January 2012 to December 2015. We recorded the main characteristics of the donors, the most important periods in the process and the evolution of liver receptors admitted to the ICU.

**Results:** 13 patients were admitted. The mean age was 58,69 ± 7,64. Most were male (12/13). Reason for ICU admission more frequent: haemorrhagic stroke (8). The ICU stay until LIST decision was 6,07 ± 3,32 days. The cares at the end of life (LIST) were performed in the first four patients in the UCI, and another patients in the operating room, intervening in all of them intensivists who had participated in the previous treatment. The time from extubation to significant hypoperfusion of organs (SBP < 60 mmHg) was 9 minutes, the time to cardiac arrest was 13,3 minutes, and to the beginning of the cold perfusión was 21,7 minutes. 7 liver transplants were performed without complications in the ICU, and the ICU stay was 2 days (1, 6). The higher ALT level was 2553 ± 1679,53. 6 of the 7 liver transplants are well and with functioning organ today (one died in hospital ward unexpected cardiac arrest). 21 kidneys were obtained from these donors.

**Conclusions:** The Maastricht III donors provide valid organs for transplantation and the intensivists play an important role both in the detection as in the development of care at the end of life. The first transplants had long functional prolonged warm ischemia, which has been reflected in graft function, but the performing of LIST in operating room, the ultrafast extractions and the presence of the receptor in the hospital are improving the viability of organs, so the results of the last donors are better. Thus, Maastricht III donors must be considered today as an additional source of organs for transplantation.

## NONVENTILATORY MANAGEMENT STRATEGIES FOR ACUTE RESPIRATORY FAILURE

### A1076 Effects of acetazolamide on the duration of mechanical ventilation in patients with metabolic alkalosis

#### G. Rialp^1^, J.M. Raurich^2^, I. Morán^3^, M.C. Martín^4^, G. Heras^5^, A. Mas^6^, I. Vallverdú^7^

##### ^1^Hosiptal Son Llàtzer, Intensive Care Department, Palma de Mallorca, Spain; ^2^Hospital Son Espases, Intensive Care Department, Palma de Mallorca, Spain; ^3^Hospital de la Santa Creu i Sant Pau, Intensive Care Department, Barcelona, Spain; ^4^Hospital Torrejón de Ardoz, Intensive Care Department, Madrid, Spain; ^5^Hospital Infanta Leonor, Intensive Care Department, Madrid, Spain, ^6^Hospital de Sant Joan Despí Moissès Broggi, Intensive Care Department, Sant Joan Despí, Spain; ^7^Hospital Sant Joan de Déu, Intensive Care Department, Reus, Spain

###### **Correspondence:** G. Rialp – Hosiptal Son Llàtzer, Intensive Care Department, Palma de Mallorca, Spain

**Introduction:** Metabolic alkalosis (MA) inhibits respiratory center drive and reduces cardiac output and can delay weaning from mechanical ventilation (MV). MA is commonly observed in chronic CO_2_ retainers that need mechanical ventilation due to acute respiratory failure. Acetazolamide (ACTZ) may be used to decrease serum bicarbonate concentration and stimulate central respiratory drive.

**Objectives:** To evaluate the effects of ACTZ on the duration of MV in patients with MA and COPD or obesity hypoventilation syndrome (OHS) intubated due to acute respiratory failure.

**Methods:** Multicenter, randomized, control group, (ACTZ 500 mg vs placebo), double-blind, with COPD or OHS with MV < 72 h and initial bicarbonate > 28 mmol/L and pH > 7.35. Test-treatment was daily administered if pH > 7.35 and bicarbonate > 26 mmol/L. Clinical, respiratory and laboratory parameters were recorded. Chi-square, student-t, and Log-Rank tests and generalized estimating equations were used to compare groups. p ≤ 0.05 was considered significant.

**Results:** 47 patients (36 men) with mean age of 67 ± 10 years were included. There were no significant differences between groups in comorbidities, baseline characteristics or arterial blood gases at inclusion. Median (IQR) days of MV in placebo and acetazolamide group were 8.3 (3.9 to 11.5) and 5.0 (2.4 to 8.3), p = 0.19, respectively. Kaplan-Meier curve did not show significant differences on the duration of MV between groups (Log-rank p = 0.41). Comparison of estimated marginal means (95 %) during MV between placebo and acetazolamide groups were, respectively: PaCO_2_ 55 (51 to 59) vs 48 (47 to 50) mm Hg, p = 0.002; bicarbonate 34 (32 to 35) vs 29 (28 to 30) mmol/L, p < 0.0001; and minute volume 9.7 (8.9 to 10.4) vs. 10.6 (9.2 to 12.0) L/min, p = 0.26. There were no severe adverse effects with ACTZ administration.

**Conclusions:** Among patients with MA and COPD or OHS, early treatment with ACTZ shortened not significantly the duration of mechanical ventilation compared with placebo. The study resulted unpowered to detect significant differences. NCT01499485

**Grant acknowledgment**

DGAVAL. AAEE.

### A1077 A paramedical protocol of neuromuscular blockade management to reduce cisatracurium consumption in ARDS patients: the TOF-ARDS study

#### S. Hraiech^1^, J. Bourenne^2^, C. Guervilly^1^, J.-M. Forel^1^, M. Adda^1^, P. Sylla^1^, A. Mouaci^1^, M. Gainnier^2^, L. Papazian^1^

##### ^1^APHM, CHU Nord, Medical ICU, Marseilles, France; ^2^APHM, CHU Timone, Medical ICU, Marseilles, France

###### **Correspondence:** S. Hraiech – APHM, CHU Nord, Medical ICU, Marseilles, France

**Introduction:** In early acute respiratory distress syndrome (ARDS), neuromuscular blockers (NMBAs) have been shown to improve survival. However, in the ACURASYS study (1), cisatracurium was administered at a constant and high posology without monitoring the depth of neuromuscular blockade.

**Objectives:** To assess if the monitoring of the train-of four (TOF) and the management of cisatracurium posology by nurses according to an algorithm can ensure an effective neuromuscular paralysis and allow to decrease cisatracurium consumption during ARDS

**Methods:** We conducted a prospective study in 2 medical ICUs. All the patients with a PaO2/FiO2 < 120 for more than 6 hours and requiring a continuous perfusion of cisatracurium were included. Neuromuscular blockade was monitored by a TOF at the adductor pollicis. Nurses followed an algorithm of adaptation of cisatracurium posology depending on the TOF with an aim of 0/4. The initial posology was based on the maximal doses recommended in anesthesiology and on the patient ideal body weight. This posology was increased and a bolus done each time that the TOF was >0. The interruption of NMBAs was decided by physicians. The initial and final posology and the daily consumption of cisatracurium, the need of performing boli, the results of TOF and the occurrence of adverse events such as patient/ventilator asynchrony were noted. Effective cisatracurium consumption was compared to the theoretical posology that would have been administered if the patient had been treated according to the ACURASYS study protocol (*i.e.* 37,5 mg/h). We also evaluated the economic impact of the reduction of cisatracurium consumption.

**Results:** From January to December 2015, we included 26 patients (mean age 58 ± 15 years). Mean IGS2 score at ICU admission was 48 ± 18. The main etiologies of ARDS were community acquired pneumonia (35 %), nosocomial pneumonia (31 %) and aspiration (27 %). Mean duration of mechanical ventilation was 20 ± 20 days. The mortality at ICU discharge was 31 %. The mean duration of cisatracurium perfusion was 72 ± 24 hours. The initial posology of cisatracurium was (median [interquartile range]) of 11 [9–12,5] mg/h as compared to 37,5 [37,5-37,5] mg/h in the ACURASYS study (p < 0,001). The final posology of cisatracurium was of 12,5 [11–15] mg/h as compared to 37,5 [37,5-37,5] mg/h in the ACURASYS study (p < 0,001) resulting in a 67 % reduction. In 80 % of patient, there was 0 to 1 modification of cisatracurium posology from the beginning to the end of the study period. Patient/ventilator asynchrony with insufficient neuromuscular blockade was diagnosed in 2 patients. The paramedical algorithm was considered feasible by 86 % of the nurses. The estimated cost-reduction was about 12 000 euros during the study period.

**Conclusions:** A paramedical protocol can help to reduce cisatracurium consumption with an effective neuromuscular blockade and without major adverse events during ARDS

**References**

1. Papazian et al.New England J Medicine 2010

### A1078 Decision-making regarding intubation apropos of sepsis-associated acute respiratory failure: a qualitative study

#### P.R. Bauer^1^, A. Kumbamu^2^, M.E. Wilson^1^, J.K. Pannu^3^, J.S. Egginton^2^, R. Kashyap^4^, O. Gajic^1^

##### ^1^Mayo Clinic, Pulmonary and Critical Care, Rochester, United States; ^2^Mayo Clinic, Center for the Science of Health Care Delivery, Rochester, United States; ^3^Mayo Clinic, Mayo Foundation for Medical Education and Research, Rochester, United States; ^4^Mayo Clinic, Anesthesia Clinical Research, Rochester, United States

###### **Correspondence:** P.R. Bauer – Mayo Clinic, Pulmonary and Critical Care, Rochester, United States

**Introduction:** Sepsis-associated acute respiratory failure is frequent, occurs early and is associated with significant mortality. With the increasing use of noninvasive techniques, timing of intubation can vary and may lead to a difference in outcome.

**Objectives:** The objectives of this study were 1) to draw on practitioners' current practice and perspectives to understand and identify practice variation in intubation and 2) to develop an explanatory theoretical model that demonstrates the relationship of various factors contributing to practice variance.

**Methods:** Between March and July 2015, using a grounded theory approach, we conducted semi-structured interviews with providers involved in intubation and audio recorded them. The interview guide focused on clinicians' perspectives on and practices of intubation in patients with sepsis and impending respiratory failure.

**Results:** Eighteen interviews were conducted with intensivists, fellows, nurse-practitioners, respiratory therapists and registered nurses. Intubation perspective and practice varied dependent on three domains: patient's characteristics, clinician's characteristics, and organizational structure. Patient factors included nature of acute illness, underlying comorbidities, clinical presentation, and patient's values. Clinician factors included background, training, experience and practice style. System factors included of standardized policies and protocols, hierarchy and team dynamics. Although most clinicians agreed that intubation is needed in case of persistent respiratory distress, altered mental status, or shock, they disagreed on when to initiate it. In different contexts, intubation could be considered as preemptive (prophylactic), therapeutic ('just in time'), and as a rescue. Assessment, reassessment, and time-limited trial off noninvasive techniques matter. Based on these results, we propose a model regarding intubation in sepsis consisting of the steps in the decisional process, a classification of the categories of timing of intubation, and decisional context factors that impact the timing of intubation.

**Conclusions:** In patients with sepsis-associated acute respiratory failure, variability of intubation was a natural phenomenon and appeared case-driven. Intubation timing should be adjusted based on explicit consideration of each patient situation, their fitness, the cadence and trajectory of their respiratory failure, the team's proficiency in providing noninvasive and invasive ventilator support, and emphasis on clear, frequent closed-loop communication of the treatment plan and rationale within the entire critical care team.

**References**

1. DeKeyser Ganz et al. Crit Care Med. 2016;44(4):680–9.

2. Iscimen et al. Crit Care Med. 2008;36(5):1518–22.

3. Kangelaris et al. Crit Care Med. 2016;44(1):120–9.

**Grant acknowledgment**

This work was funded by the 2015 Innovation Awards from the Department of Medicine, Mayo Clinic in Rochester, USA.

### A1079 Recombinant human soluble thrombomodulin (RHTM) in septic patients with severe respiratory failure: sub-analysis from JSEPTIC DIC study

#### S. Yoshihiro^1^, M. Sakuraya^2^, M. Hayakawa^3^, A. Hirata^2^, N. Kawamura^2^, T. Tsutui^2^, K. Yoshida^2^, Y. Hashimoto^1^, Japan Septic Disseminated Intravascular Coagulation (JSEPTIC DIC) study group

##### ^1^JA Hiroshima General Hospital, Pharmaceutical Department, Hatsukaiti-shi, Hiroshima, Japan; ^2^JA Hiroshima General Hospital, Division of Emergency and Critical Care Medicine, Hatsukaiti-shi, Hiroshima, Japan; ^3^Hokkaido University Hospital, Emergency and Critical Care Center, Sapporo, Hokkaido, Japan

###### **Correspondence:** S. Yoshihiro – JA Hiroshima General Hospital, Pharmaceutical Department, Hatsukaiti-shi, Hiroshima, Japan

**Introduction:** Recombinant human soluble thrombomodulin (rhTM) is a novel therapeutic agents for Disseminated Intravascular Coagulation (DIC). rhTM might improve clinical outcomes of septic DIC patients with respiratory failure in a observational study ^(1)^.

**Objectives:** To determine the efficacy of rhTM in septic patients with severe respiratory failure.

**Methods:** We performed sub-analysis of a retrospective observational study (Japan Septic Disseminated Intravascular Coagulation study, J-SEPTIC DIC study), which was conducted in 42 intensive care units in Japan. Among 3195 septic patients enrolled in this original trial, we selected septic patients with severe respiratory failure and compared patients based on rhTM treatment (rhTM group and control group). Propensity score analysis was performed between two groups. Outcome was the number of ventilator free days.

**Results:** 1180 patients (rhTM, n = 356, control, n = 824) were analyzed in this trial. After adjusting for baseline imbalances by propensity score analysis, VFDs increased significantly in rhTM group (rhTM group: 11.9 ± 10.3 days vs. control group: 10.3 ± 10.4 days, *P* = 0.03).

**Conclusions:** In this analysis, rhTM improved outcomes in septic patients with severe respiratory failure. We need further evaluation.

**References**

1. Yamakawa K, et al., Recombinant human soluble thrombomodulin in sepsis-induced disseminated intravascular coagulation: a multicenter propensity score analysis, Intensive Care Med 2013, 39, 644–52.

### A1080 Glucocorticoid therapy on mortality in acute respiratory distress syndrome (ARDS) with diffuse alveolar damage (DAD)

#### C.-H. Chang^1^, H.-C. Hu^2^, L.-C. Chiu^2^, C.-Y. Hung^2^, S.-H. Li^2^, K.-C. Kao^2^

##### ^1^Chang Gung Memorial Hospital, Taoyuan, Taiwan, Province of China; ^2^Chang Gung Memorial Hospital, Linkou, Taoyuan City, Taiwan, Province of China

###### **Correspondence:** C.-H. Chang – Chang Gung Memorial Hospital, Taoyuan, Taiwan, Province of China

**Introduction:** Diffuse alveolar damage (DAD) is a typical pathological finding of acute respiratory distress syndrome (ARDS) patients with open lung biopsy. The incidence of DAD finding of ARDS patients in the previous studies was about 50 % and seemed to have poor prognosis than non-DAD patients. The effect of glucocorticoid therapy on mortality in ARDS patients with DAD remains controversial.

**Objectives:** To investigate the survival predictors and impact of glucocorticoid therapy in ARDS patients with pathological finding of DAD.

**Methods:** We retrospectively reviewed all the patients who met the Berlin definition of ARDS and underwent open lung biopsy from January 2002 to June 2015 at Chang Gung Memorial Hospital in Taiwan. Of these patients with open lung biopsy, the pathological finding compatible with DAD were enrolled for analysis. Clinical data including baseline characteristics, severity of ARDS, medication, management and survival outcomes were investigated.

**Results:** During the study period, totally 64 ARDS patients with pathologic diagnosis of DAD were eligible for analysis. These 64 patients were divided as mild (n = 14, 21.9 %), moderate (n = 36, 56.2 %) and severe ARDS (n = 14, 21.9 %) by Berlin definition and the hospital mortality rate were not significantly different between these three groups (64.3 %, 69.4 % and 85.7 %, p = 0.4). According to the etiology, these 64 DAD patients were divided into known etiology group (n = 47, 73.4 %) and unknown etiology group (n = 17, 26.6 %), and the hospital mortality rate had no significant difference (72.3 % vs 70.6 %, p = 0.890). The known etiology group had higher percentage of male and lower PaO2/FiO2 ratio than unknown etiology group (72.3 % vs 41.2 %, p = 0.022; 202.3 ± 84.4 vs 146.3 ± 60.0, p = 0.005). The multivariable logistic regression revealed sequential organ failure assessment (SOFA) score at the time of open lung biopsy was the only predictor of hospital mortality (odds ratio 1.413, 95 % confidence interval 1.127-1.772; p = 0.03). In terms of glucocorticoid treatment, there was no significant difference in glucocorticoid use, timing from ARDS to glucocorticoid use, dose and duration between survival and nonsurvival patients.

**Conclusions:** For the ARDS patients with DAD, SOFA score was the predictor of hospital mortality but glucocorticoid treatment did not improve the survival rate.

### A1081 Comparison of subglottic suction drainage devices versus standard endotracheal tubes in the development of tracheal injury

#### S. Sibley^1^, J. Drover^1^, C. D'Arsigny^1^, C. Parker^1^, D. Howes^1^, S. Moffatt^1^, J. Erb^1^, R. Ilan^1^, D. Messenger^1^, I. Ball^2^, J.G. Boyd^1^, M. Harrison^1^, S. Ridi^1^, J. Muscedere^1^

##### ^1^Queen's University, Critical Care Medicine, Kingston, Canada; ^2^University of Western Ontario, Critical Care Medicine, London, Canada

###### **Correspondence:** S. Sibley – Queen's University, Critical Care Medicine, Kingston, Canada

**Introduction:** Ventilator associated pneumonia (VAP) is a known complication of mechanical ventilation. Aspiration of oropharyngeal secretions results in infection that leads to significant morbidity, mortality and cost^1^. Use of sub-glottic secretion drainage (SSD) devices have been shown to decrease both the incidence of VAP and intensive care unit (ICU) days^2,3^.

There have been safety concerns associated with use of SSD devices and herniation of tracheal tissue into the suction port^4^. A study in sheep showed significant tracheal injury associated with continuous suction^5^. Human studies have shown conflicting results regarding the risk of tracheal injury^6,7^.

**Objectives:** To determine the risk of tracheal injury using an SSD device versus a standard endotracheal tube.

**Methods:** 57 patients undergoing tracheostomy in the ICU were enrolled in the study. Patients were intubated in the ICU, operating or emergency room, pre-hospital, or referring hospitals. Intubation conditions and duration of intubation were documented. At the time of tracheostomy, a bronchoscopy was performed and the presence and degree of tracheal injury were noted. Patients were followed to hospital discharge and decannulation, otolaryngology consults, and discharge or death were recorded.

**Results:** 41 patients were intubated with a Malinckrodt EVAC SSD device and 16 were intubated with a standard endotracheal tube. 33 patients were found to have a tracheal injury ranging from mild erythema to severe ulceration; 23/41 (56 %) in the EVAC group and 10/16 (63 %) in the standard group (RR - 0.89; 95 % CI 0.56 to 1.43). 5/41 (12 %) patients were reported to have injury at the site of the suction port; 2 were reported to have mild edema and erythema and 3 had mild to moderate ulceration. Of the patients with tracheal ulceration at the suction catheter port, 4 were decannulated successfully without further complication and 1 patient died prior to termination of mechanical ventilation.

**Conclusions:** There was no significant difference in the risk of tracheal injury with SSD devices compared to standard endotracheal tubes. The degree of injury was similar in both groups. A small number of patients had ulceration at the site of the suction port but did not suffer any complication as a result.

**References**

1. Muscedere JG, Martin CM, Heyland DK. J Crit Care 2008, **23:** 5–10.

2. Dezfulian C, Shojania K, Collard HR, et al. Am J Med 2005, **118:** 11–18.

3. Lacherade JC, De Jonghe B, Guezennec P, et al. Am J Respir Crit Care Med 2010, 182: 910–917.

4. Dragoumanis CK, Vretzakis GI, Papaioannou VE, et al. Anesth Analg 2007, **105:**1083**–**1085.

5. Berra L, De Marchi L, Panigada M, et al. Crit Care Med 2004, **32:** 2071–2078.

6. Deem S, Treggiari MM. Respir Care 2010, **55:** 1046–1055.

7. Girou E, Buu-Hoi A, Stephan F, et al. Intensive Care Med 2004, **30:** 225–233.

### A1082 Successeful strategy to reduce ventilator-associated pneumonia

#### A.H. Andrade^1^, R.C. Costa^1^, V.A. Souza^1^, V. Gonzalez^1^, V. Amorim^1^, F. Rolla^1^, C.A.C. Abreu Filho^1^, R. Miranda^2^

##### ^1^Hospital Municipal Moyses Deutsch, ICU, São Paulo, Brazil; ^2^Hospital Municipal Moyses Deutsch, Quality Sector, São Paulo, Brazil

###### **Correspondence:** A.H. Andrade – Hospital Municipal Moyses Deutsch, ICU, São Paulo, Brazil

**Introduction:** VAP rates in Brazil are higher than those listed in Europe and EUA.No Municipal Hospital Moyses Deutsch since 2009 implemented the protocol, applied the five strategies to reduce VAP, however we maintained VAP density with little reduction and we never zero target . Objectives.Objective of the study was to examine the effect of Healthcare Improvement ventilation bundle Institute in addition to focusing on three strategies : Oral decontamination with chlorhexidine (ODC), the head elevation and awakening daily in the incidence of VAP in a unit intensive care.

**Methods:** The study was conducted in a 20-bed, medical-surgical ICU. Criteria for nosocomial pneumonia are those from the CDC. Strategy was to implement the IHI's ventilator bundle , focused and optimized in the first three The goals were the ICU team adhesion of 80 % achieved in six month after bundle implementation and 98 % after one year of follow up. These measures included five strategies to prevent ventilator-associated pneumonia: 1- 45° elevation of the head of the bed, 2-adequate sedation level (RASS −1 a −2 ), 3- oral decontamination with chlorhexidine 0.12 %, 4- DVT/PE prevention and 5- peptic ulcer prophylaxis . From January 2012 on, the ICU nursing staff and ICT performed a daily checklist in order to observe the five issues accomplishment. If any item was found to be inadequate it was promptly corrected.

**Results:** In January and December 2012 , adhesion to the whole bundle was 68 % and 100 % respectively. VAP density was proportionally lower to bundle adhesion in the same period, 20 per 1000 ventilation/day and respectively. In 2015 we achieved zero VAP in both semesters.

**Conclusions:** Initial VAP rates were extremely high even for Brazilian benchmarks. Although we could not implement expensive technologies like continuous aspiration of subglottic secretions, ICU team and ICT efforts were crucial for satisfactory results, as well the administrative board support, which turned this issue an institutional priority. Our goals are to reduce even more, implementing ''ventilator bundle—getting to zero'' program, maintaining a continuum effort to sustain these results.

**References**

1. Koeman M, Ven AJAMVD, Hak E, Joore HCA, Kaasjager K, Smet AGA et al. Oral decontamination with chlorhexidine reduces the incidence of ventilator-associated pneumonia. Am J Respir Crit Care Med 2006;173(12):1348–55. 5 Million Lives Campaign. Getting Started Kit: Prevent Ventilator Associated Pneumonia. Cambridge MA: Institute for Healthcare Improvement; 2008. [ 10 ago. 2011]. www.ihi.org.

**Grant acknowledgment**

Without conflict concern

**Fig. 112 (abstract A1082).**
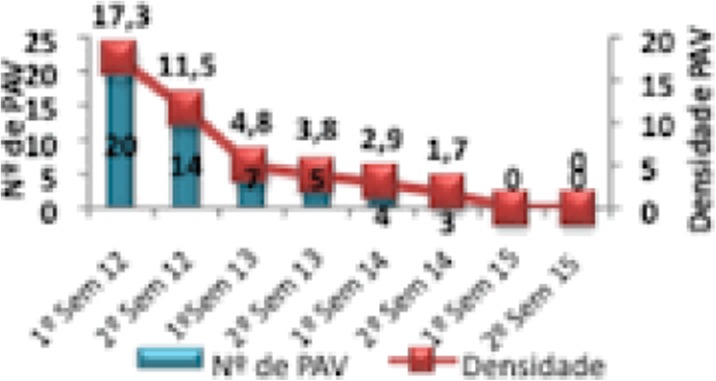
VAP

### A1083 Implementation of chula ventilator bundle to prevent ventilators-associated pneumonia

#### S. Atchasiri, P. Buranavanich, T. Wathanawatthu, S. Suwanpasu

##### KCMH, Nursing Department, Bangkok, Thailand

###### **Correspondence:** S. Atchasiri – KCMH, Nursing Department, Bangkok, Thailand

**Introduction:** Ventilators-Associated pneumonia (VAP) and its prevention is a significant concern for ventilated patients in the acute care.

**Objectives:** To determine if the knowledge and awareness of "ventilator bundle" helped in the prevention of ventilator associated pneumonia in the patients admitted to hospital.

**Methods:** A prospective observational study that evaluated VAP rates from August through October 2015 were evaluated. All the adult medical patients who were intubated and ventilated in 8 medical wards from August through October in the year 2015 were included in the study. During the period of June to July 2015 the staff nurses were educated and made aware about the problem of VAP and the use of ventilator bundle in helping to prevent this VAP. Patients who expired within 24 hrs of admission, who were transferred to intensive care unit within 48 hrs, and those who were diagnosed with pulmonary embolism or metastasis were excluded from this study.

**Intervention.** The concept of "ventilator bundle' was introduced after educating the nursing staff and the medical personnel through group discussions.”CHULA Ventilator bundle" is a package of evidence -based interventions that include:

1. Clean equipment and environment;
2. Hand Hygiene and Elevation of patient's head of bed to 30–45 degrees;
3. Use 0.12 % chlorhexidine as a part of oral care every 4 hour;
4. Labor over weaning and extubation each day;
5. Aspiration precaution protocol.

**Measurement.** Demographic data was collected from the patient data files. VAP was diagnosed when it met the (clinical non-invasive) diagnostic criteria. Incidence of VAP and protocol compliant were calculated.

**Results:** A total of 147 were on mechanical ventilator for a vary period of 0–90 days. Average age was 71.48 ± 15.92 with 59.5 % of male. Introducing the concept of "CHULA Ventilator Bundle to Prevent Ventilators-Associated pneumonia" significantly reduced the VAP rate per 1000 ventilator days from 12 % to 0 % in the medical group (8 medical wards). It significantly reduced the incidence of oral cavity problem (5.72 ± 1.352 vs. 5.23 ± 0.88, p = < 0.001). Ventilator Bundle compliance was 60.14 %.

**Conclusions:** However, Ventilator Bundle compliance was less than 80 %, introducing the concept of "CHULA ventilator bundle" helped us to reduce the incidence of VAP and the incidence of oral cavity problem.

**Grant acknowledgment**

Quality Improvement Center, King Chulalongkorn Memorial Hospital

### A1084 Risk factors and prognosis impact of decreased patient-ventilator asynchrony in mechanically ventilated patients - a prospective study

#### C. Bureau^1^, C. Rolland-Debord^1^, T. Poitou^1^, M. Clavel^2^, S. Perbet^3^, N. Terzi^4^, A. Kouatchet^5^, T. Similowski^1^, A. Demoule^1^

##### ^1^Université Pierre et Marie Curie, UMR_S 1158 and Hôpital Pitié-Salpêtrière, Respiratory Division and Medical ICU, Paris, France; ^2^Hôpital Dupuytren, Limoges, France; ^3^CHU de Clermont-Ferrand and Université d'Auvergne, Clermont-Ferrand, France; ^4^Université Grenoble-Alpes and CHU Grenoble Alpes, Grenoble, France; ^5^CHU d'Angers, Angers, France

###### **Correspondence:** C. Bureau – Université Pierre et Marie Curie, UMR_S 1158 and Hôpital Pitié-Salpêtrière, Respiratory Division and Medical ICU, Paris, France

**Introduction:** Patient-ventilator asynchrony is a mismatch between patient and ventilator inspiratory and expiratory time. It is associated with prolonged duration of mechanical ventilation (MV), increased need for tracheostomy and increased mortality. Five main patterns of asynchrony are described, without universal agreement on definition. Studies on patient ventilator asynchrony have quantified asynchrony at heterogeneous time points and during periods of various durations. In addition, most of these studies were of single centre type.

**Objectives:** The aim of the present study was to evaluate the factors associated with and the prognosis impact of asynchrony, according to two methods of quantification: visual inspection of airway flow and pressure signal and a computerized method integrating electromyographic activity of the diaphragm (EAdi) as a maker of patient inspiratory time at the early phase of weaning.

**Methods:** Ancillary study of a multicentre, randomized controlled trial comparing neurally adjusted ventilator assist to pressure support ventilation at early phase of weaning. Airway flow, pressure and EAdi were recorded during 20 minutes 12, 24, 36 and 48 hours following inclusion. Asynchrony were quantified according to two methods: 1) “flow-and-pressure” based on the visual inspection of the flow and pressure signals 2) “EAdi-based” with analysis of the EAdi signal in addition to the flow and pressure signals. Asynchrony index (AI) was calculated as the number of asynchronous breaths divided by the total number breaths multiplied by 100.

**Results:** 103 patients mechanically ventilated for 5 days (3–9) were included, 72 men (68 %), aged 66 (60–77) years, SAPS II 44 (35–59), 62 % were mechanically ventilated for de novo hypoxemic respiratory failure.

Prevalence of ineffective efforts was higher with flow-and-pressure method than with EAdi-based method. Auto-triggering, double-triggering, premature and late cycling were more frequently observed with EAdi-based method than with flow-and-pressure method. AI and the total prevalence of asynchrony were significantly lower with the flow-and-pressure method than with the EAdi-based method (Table 78).

No significant difference in term of gender, age, SAPS 2, Charlson score or length of MV prior to inclusion was observed with severe asynchrony (AI >10 %)

Severe asynchrony was not associated with difference in term of hospital length of stay, duration of MV and day-28 mortality.

ICU length of stay determined by the flow-and-pressure method was shorter in patients with AI ≥ 10 % (18 (14–28) vs 9 (9–12), p = 0.02).

**Conclusions:** The prevalence of patient ventilator asynchrony varies according the methods and definitions used to quantify asynchrony, which suggests the need for a consensus statement in asynchrony's definition. Patient ventilator asynchrony was not associated with a poorer outcome.

**Table 77 (abstract A1084).** Prevalence of asynchrony

	Flow-and-pressure	EAdi-based	p
Ineffective triggering, min-1	0.02 (0.00–0.11)	0.00 (0.00–0.05)	0.028
Auto triggering, min-1	0.00 (0.00–0.02)	0.47 (0.20–1.03)	<0.0001
Double-triggering, min-1	0.15 (0.05–0.41)	1.12 (0.18–2.57)	<0.0001
Premature cycling, min-1	0.00 (0.00–0.01)	0.67 (0.25–1.62)	<0.0001
Late cycling, min-1	0.00 (0.00–0.01)	1.09 (0.28–2.33)	<0.0001
All asynchrony, min-1	0.30 (0.20–0.80)	4.70 (3.20–7.70)	<0.0001
Asynchrony Index, %	1.00 (1.00–3.25)	18.50 (12.80–31.50)	<0.0001

### A1085 Tracheostomy in critically ill patients: trends, utilization, timing and outcomes

#### P. Diaz, J. Nunes, S. Escórcio, G. Silva, S. Chaves, M. Jardim, M. Câmara, N. Fernandes, R. Duarte, J.J. Jardim, C.A. Pereira, J.J. Nóbrega

##### Hospital Dr. Nélio Mendonça, Serviço de Medicina Intensiva, Funchal, Portugal

###### **Correspondence:** P. Diaz – Hospital Dr. Nélio Mendonça, Serviço de Medicina Intensiva, Funchal, Portugal

**Introduction:** Tracheostomy is a frequent procedure in intensive care units, in the US over the past 2 decades utilization rose substantially, driven by surgical patients [1]. The optimal timing for tracheostomy in critically ill patients remains a topic of debate.

**Objectives:** To analyse tracheostomy utilization and trends in an intensive care unit (ICU) and to determine the impact of tracheostomy timing (early vs late) in critically ill patients on duration of mechanical ventilation, ICU stay, overall hospital stay and mortality.

**Methods:** Retrospective study including all critically ill patients who underwent tracheostomy in an ICU from 2005 to 2015. The sample was stratified in two groups, according to time of invasive mechanical ventilation until tracheostomy: early tracheostomy (≤ 10 days) and late (> 10 days).

**Results:** Over the study period a total of 688 tracheostomies were performed, representing 15,3 % of the admissions in the ICU. Tracheostomy was more common in medical patients (67.4 %). Mean time until tracheostomy was 9 days. There was no tendency in tracheostomy rates and timing over the years. Early and late tracheostomy groups did not differ significantly by gender, age, SOFA score and type of admission. In the early tracheostomy group there was a statistically significant reduction in the length of invasive mechanical ventilation (10 days vs 25 days, *p < 0.001*) and ICU stay (13 days vs 28 days, *p < 0.001*), with impact in ICU and hospital mortality.

**Conclusions:** Early tracheostomy was associated with reduction in invasive mechanical ventilation days and ICU stay, with possible implications in long term morbidity and health care costs. Reinforcing that tracheostomy timing should be considered in the decision process, when evaluating risks and benefits.

**References**

1- Mehta AB, Syeda SN, Bajpayee L, Cooke CR, Walkey AJ, Wiener RS. Trends in Tracheostomy for Mechanically Ventilated Patients in the United States, 1993 – 2012. Am J Respir Crit Care Med. 2015. 192(4):446-54.

### A1086 The impact of liver cirrhosis on long-term outcome after a first-ever mechanical ventilation: a population-based study

#### C.-M. Chen^1,2^, C.-C. Lai^3^, K.-C. Cheng^4^, W. Chou^1,5^

##### ^1^Chia Nan University of Pharmacy and Science, Recreation and Health-Care Management, Tainan, Taiwan, Province of China; ^2^Chi-Mei Medical Center, Intensive Care Medicine, Tainan, Taiwan, Province of China; ^3^Chi-Mei Medical Center, Liouying District, Intensive Care Medicine, Tainan, Taiwan, Province of China; ^4^Chi Mei Medical Center, Internal Medicine, Tainan, Taiwan, Province of China; ^5^Chi-Mei Medical Center, Tainan, Taiwan, Province of China

###### **Correspondence:** C.-M. Chen – Chia Nan University of Pharmacy and Science, Recreation and Health-Care Management, Tainan, Taiwan, Province of China

**Introduction:** This study is to assess the effect of liver cirrhosis (LC) on the poorly understood long-term mortality risk after a first-ever mechanical ventilation (1-MV) for acute respiratory failure.

**Objectives:** All patients given a 1-MV between 1997 and 2013 from the Longitudinal Health Insurance Database 2000 (LHID2000) which randomly selected 1,000,000 beneficiaries in 2000 were identified.

**Methods:** Patients with LC were individually matched to patients without LC (ratio: 1:2) using a propensity-score method. The primary outcome was death after a 1-MV.

**Results:** A total of 16,695 patients were enrolled, including the 5,565 LC patients and 11,130 controls without LC. Patients with LC had more organ failures and more likely to admit to medical department than controls without LC. Patients with LC had a lower survival rate than patients without LC (adjusted hazard ratio [HR] 1.41; 95 % CI: 1.36-1.47). Moreover, the risk of mortality was highest among patients with LC (HR: 1.60, 95 % CI: 1.53-1.67,) followed by patients with cryptogenic LC (HR: 1.08, 95 % CI: 1.01-1.15) and patients without LC.

**Conclusions:** Compared to controls without LC, patients with LC and cryptogenic LC, can increase the mortality risk after a 1-MV.

**References**

1. Levesque, E., Saliba, F., Ichai, P., & Samuel, D.. Outcome of patients with cirrhosis requiring mechanical ventilation in ICU. *J. Hepatol.* 60, 570–578 (2014).

2. Shawcross, D. L. *et al.* The impact of organ dysfunction in cirrhosis: survival at a cost? *J. Hepatol* 56, 1054–1062 (2012).

**Grant acknowledgment**

Grant from Chi Mei Medical Center (CMFHR10535)

### A1087 Drainage of pleural effusion with small bore tube in mechanically ventilated patients

#### S.J. Lee^1^, Y.S. Cha^2^, W.-Y. Lee^1^

##### ^1^Yonsei University Wonju College of Medicine, Internal Medicine, Wonju, Republic of Korea; ^2^Yonsei University Wonju College of Medicine, Emergency Medicine, Wonju, Republic of Korea

###### **Correspondence:** S.J. Lee – Yonsei University Wonju College of Medicine, Internal Medicine, Wonju, Republic of Korea

**Introduction:** Critically ill patients under mechanical ventilation (MV) were often complicated by pleural effusion. Improvement in US-guided procedure brings both early diagnosis and safe management. However, there was limited evidence in the benefit of effusion drainage and the impact on ultimate results.

**Objectives:** We designed this study to investigate effect of effusion drainage in patients under MV and to reveal the prognosis and risk factors for weaning failure.

**Methods:** Following a retrospective review from January to December 2014, 80 patients who underwent drainage of pleural effusion via small bore tube during MV care were enrolled. We compared respiratory rate and parameters of arterial blood gas analysis performed before and after drainage of pleural effusion. Also, risk factors for weaning failure and prognostic parameters were investigated.

**Results:** The median age of the patients was 72 years and 50 % was male. Of total 80 Patients, 30 patients (37.5 %) could discharge from hospital without need for MV. Thirty six patients (45 %) died in our hospital. Respiratory rate decreased after drainage of effusion (22 [IQR 18–29] vs 21 per min [17–26], p = 0.011). Partial oxygen pressure (PaO_2_) increased after drainage (98 [82–125] vs 112 mmHg [81–132], p = 0.021). However, other parameters of arterial blood gas did not show difference between before and after drainage.

Among fourteen (17.5 %) and 39 (51.3 %) patients, MV support level could be decreased within one hour and one day respectively. However, there was no difference in the frequency of lowering the support level between weaning success and weaning failure group (one hour, p = 0.65; one day, p = 0.78). Improvement of PaO_2_ after drainage was also not associated the result of weaning success (60 vs 66 % in weaning success vs failure group, p = 0.59). On the other hand, high score of APACHE II (15 [12–20] vs 24 [17–26], p = 0.003) showed association with a risk of weaning failure. Old age (70 [54–75] vs 74 years [61–79], p = 0.086) and cardiac dysfunction including systolic or diastolic dysfunction and pulmonary hypertension (81 vs 95 %, p = 0.086) showed a trend for association with a risk of weaning failure.

**Conclusions:** In this study, we revealed improvement of respiratory rate and PaO_2_ after effusion drainage via small bore chest tube in the patients under MV treatment. However, increase of PaO_2_ was not associated with weaning success. Instead, we suggest disease severity or heart failure, rather than immediate improvement of PaO_2_ after drainage, may have an association with the risk for weaning failure.

### A1088 ICU-acquired weakness. Does it develop within a week?

#### M. Onodera^1^, E. Nakataki^1^, J. Oto^1^, H. Imanaka^2^, M. Nishimura^1^

##### ^1^Tokushima University Graduate School, Critical Care and Emergency Medicine, Tokushima, Japan; ^2^Takarazuka City Hospital, Intensive Care Unit, Takarazuka, Japan

###### **Correspondence:** M. Onodera – Tokushima University Graduate School, Critical Care and Emergency Medicine, Tokushima, Japan

**Introduction:** Development of critical care medicine has been decreasing mortality of critical illness. However, 60-80 % of survivors suffer functional impairment or ICU-acquired weakness (ICU-AW). In order to address interventions in ICU-AW, it is essential to know when ICU-AW developed in addition to its incidence and risk factors.

**Objectives:** To assess the onset of ICU-AW and its incidence and risk factors in the ICU of Tokushima University Hospital.

**Methods:** Prospective observational study. Critically ill adults were enrolled when they were mechanically ventilated at least 4 days. Patients younger than 20 years old, with neuromuscular diseases, central nervous system disorders, and pregnancy were excluded. After we determined feasibility of communication, medical research council (MRC) sum score was measured as soon as possible. When MRC score was less than 48, we diagnosed patient as ICU-AW. Basic profiles, underlying diseases, APACHE II score, administration of neuromuscular blocking agents (NMBA) and corticosteroids, and laboratory data were recorded. The protocol of the study was approved by the IRB of Tokushima University Hospital.

**Results:** During study period, 460 adults were admitted to the ICU, while 302 were mechanically ventilated. We excluded 279 patients because of impaired consciousness or mechanical ventilation (MV) duration less than 4 days. Among remaining 23 patients, six (26 %) were diagnosed as ICU-AW. Basic profiles and APACHE II score were not different between the patients with and without ICU-AW. NMBA and corticosteroids were administered in 1 and 2 ICU-AW patients, respectively. Blood glucose, electrolytes, creatinine kinase, and albumin levels were not different between the patients with and without ICU-AW. Duration of MV and length of hospital stay were longer in patients with ICU-AW than in patients without ICU-AW (median [IQR], 11.5 [7.3 - 40.8] vs. 4 [4.0 - 7.5] days and 75 [58.8 - 103.3] vs. 37 [25.0 - 72.5] days, respectively). Three patients with ICU-AW were tracheostomized. ICU-AW was diagnosed as early as on 5th MV day. Hospital mortality was 17 % (1/6) in ICU-AW patients and 24 % (4/17) in non-ICU-AW patients.

**Discussion.** The incidence of ICU-AW was 26 % at our ICU, while it reportedly varied 25-100 %. In line with previous reports, duration of MV and length of hospital stay were significantly longer in the patients with ICU-AW. We included the patients who were mechanically ventilated at least 4 days, and one of our patients was diagnosed as ICU-AW as early as on 5th MV day. ICU-AW usually develops in the patients who are mechanically ventilated prolonged periods. The number of enrolled patients in the present study was small, and we could not find any factors related to early onset of ICU-AW.

**Conclusions:** Prolonged MV is a risk factor of ICU-AW. However, we should be aware that ICU-AW can develop in a shorter period than we expect.

**Grant acknowledgment**

Departmental.

### A1089 Assessment of respiratory support methods in patients with intracranial hypertension in severe traumatic brain injury

#### A. Khadjibaev^1^, D. Sabirov^2^, A. Rosstalnaya^2^, R. Akalaev^1^, F. Parpibaev^1^

##### ^1^Uzbekistan Research Center of Emergency Medicine, Tashkent, Uzbekistan; ^2^Tashkent Institute of Postgraduate Medical Education, Tashkent, Uzbekistan

###### **Correspondence:** A. Khadjibaev – Uzbekistan Research Center of Emergency Medicine, Tashkent, Uzbekistan

**Introduction:** Severe traumatic brain injury (STBI) remains as the most significant medical and social problem due to high prevalence and mortality, primarily among young and employable population. The leading problem of intensive care of STBI is the prevention and elimination of intracranial hypertension (ICH). One of the methods of ICH elimination is mechanical ventilation as a component of complex therapy. Among the various methods and modes of mechanical ventilation high-frequency jet ventilation (HFJV) is particularly distinguished, which is enduring “the second birth”. In HFV transpulmonary pressure and the pressure in airways is much lower than one during traditional methods, the negative pressure in pleural cavity is maintained during inspiration phase and spontaneous breathing.

**Objectives:** Comparative assessment of efficacy of different modes of mechanical ventilation in patients with STBI.

**Methods:** We studied the cerebral perfusion during various modes of mechanical ventilation in 70 patients with STBI. Mean age was 32 ± 5. The general status in admission was severe, Glasgow coma score was 7 ± 3. All patients had traditional intensive care with different modes of respiratory support: controlled mechanical ventilation - CMV (n = 10); synchronized intermittent mandatory ventilation - SIMV (n = 20); HFJV (n = 40). The efficacy of all modes were assessed by arterial blood gases analysis (SaO2 - 96-99 %, pCO2 - 34.7-35.2 mmHg). Intracranial pressure were measured invasively and was 20 ± 5 mmHg. All patients regularly had clinical and neurological examination, control of laboratory tests (common blood count, arterial blood gases, arteriovenous gradient of O2 (AVDO2) and oxygen saturation in jugular vein (SjO2). Cerebral hemodynamics was studied by transcranial dopplerography. The registered parameters were mean linear velocity of cerebral blood flow (Vm), pulsatile index (Pi) and overshoot coefficient (OC).

**Results:** There were significant differences in parameters of cerebral hemodynamics in various modes of respiratory support: CMV: Vm - 51.1 ± 1.4 cm/sec, Pi - 1.84 ± 0.1, OC - 1.28 ± 0.01

CMV: Vm - 52.6 ± 4.1 cm/sec, Pi - 1.60 ± 0.1, OC - 1.23 ± 0.02

CMV: Vm - 57.8 ± 7.1 cm/sec, Pi - 1.39 ± 0.2, OC - 1.36 ± 0.01

In HFJV intracranial pressure (ICP) was lower, cerebral perfusion pressure (CPP) was higher and AVDO2 was moderately increased. Besides, mean arterial blood pressure was directly proportional to SjO2 and inversely proportional to AVDO2 in all modes.

CMV: AVDO2 - 58.6 ± 5.5 %, SjO2 - 38.4 ± 3.2 %, ICP - 28.6 ± 2.7 mmHg, CPP 64.6 ± 2.8 mmHg

SIMV: AVDO2 - 45.1 ± 3.3 %, SjO2 - 48.9 ± 3.2 %, ICP - 26.5 ± 2.4 mmHg, CPP 68.0 ± 2.8 mmHg

HFJV: AVDO2 - 59.7 ± 3.4 %, SjO2 - 53.8 ± 3.1 %, ICP - 24.7 ± 2.9 mmHg, CPP 70.1 ± 3.0 mmHg

**Conclusions:** Hence, the analysis results of ICH complex therapy in STBI show the advantages of HFJV over the other modes of mechanical ventilation as it is devoid of negative effects of traditional mechanical ventilation.

## OUTCOME ANALYSIS III

### A1090 Tracheostomy and invasive mechanical ventilation in intermediate care unit: an alternative approach

#### E. Antonucci^1^, P. Rossini^1^, S. Gandolfi^2^, E. Montini^1^, S. Orlando^1^

##### ^1^Guglielmo da Saliceto Hospital, Intermediate Care Unit, Piacenza, Italy; ^2^Guglielmo da Saliceto Hospital, Local Health Care Unit, Piacenza, Italy

###### **Correspondence:** E. Antonucci – Guglielmo da Saliceto Hospital, Intermediate Care Unit, Piacenza, Italy

**Introduction:** Intermediate Care Unit (IMCU) may provide more intensive monitoring and patient management than the general ward but less than are offered in Intensive Care Unit (ICU)^1^. Patients undergoing tracheostomy usually need invasive mechanical ventilation (IMV) and prolonged ICU stay with increased hospital costs and mortality rate.

**Objectives:** We hypothesized that IMCU may represent a valid option to manage critically ill patients undergoing tracheostomy and mechanical ventilation and reduce ICU stay.

**Methods:** We reviewed all patients discharged from Piacenza ICU and directly admitted in our IMCU (from June 2014 to December 2015, 19 months). The inclusion criteria were age ≥ 18 y.o., need of IMV, tracheostomy performing during ICU stay. We collected demographic characteristics, comorbidities, reasons for ICU admission; complications during IMCU stay and timing of tracheostomy (early: ≤ 10 days after tracheal intubation; late: > 10 days after tracheal intubation). We analyzed rate of decannulation, IMCU mortality and mortality at 28 days after IMCU discharge.

**Fig. 113 (abstract A1090).**
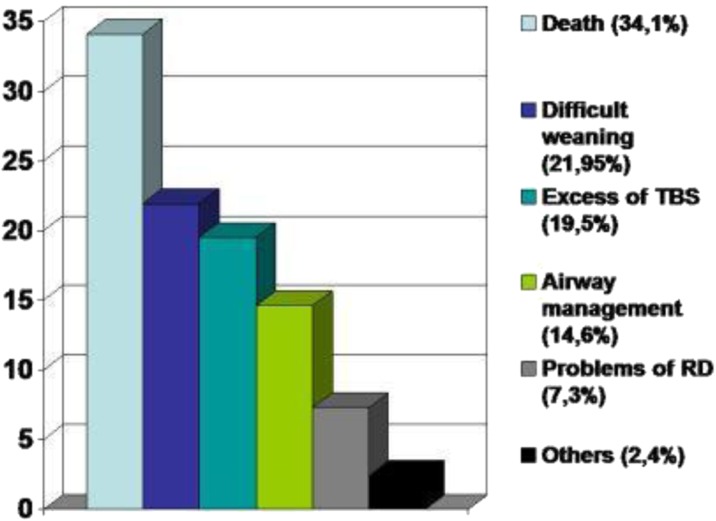
Causes of decannulation failure

**Fig. 114 (abstract A1090).**
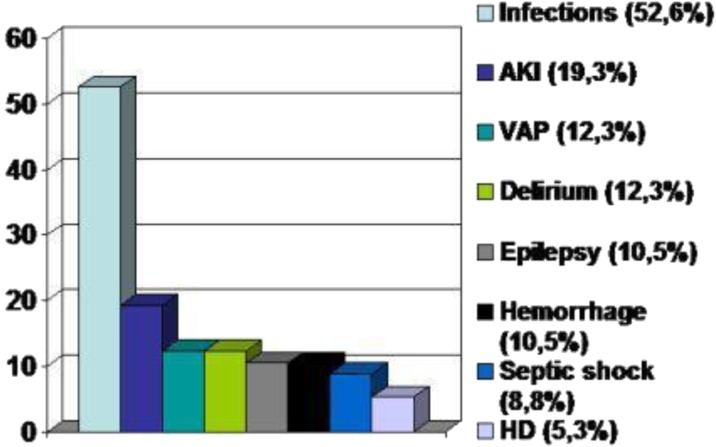
Complications during IMCU stay

**Results:** 57 from a total of 145 analyzed patients (39,3 %) respected inclusion criteria. Patients' characteristics, comorbidities and causes of admission are shown in the tables. Median timing of tracheostomy was 12 days (3–28) with 22/57 patients (38,6 %) undergoing early tracheostomy and 35/57 patients (61,4 %) undergoing late tracheostomy. We decannulated 16/57 patients (28,1 %) in a median time of 15 days (1–42). Figure 113 summarizes causes of decannulation failure, while Figure 114 shows the most common complications occurred during IMCU stay. Death occurred in 13/57 patients (22,8 %) during IMCU stay and in 7/44 patients (15,9 %) at 28 days after IMCU discharge. All decannulated patients survived at 28 days after IMCU discharge. Both decannulated and not decannulated patients had no significant differences in terms of age and BMI. SAPS2 score was significant higher in not decannulated patients (p = 0.01). Ventilator-associated pneumonia (VAP) acquired during IMCU stay seemed correlate with decannulation failure although not in a statistical significant mode (p = 0.078). Timing of tracheostomy was not related with mortality rate at 28 days after IMCU discharge (early 36,4 % vs late 34,3 %) and with decannulation rate (p = 0.48) .

**Conclusions:** Patients undergoing tracheostomy need prolonged hospital stay and usually develop medical complications. IMCU may represent an alternative approach to manage these patients accelerating ICU discharge. Decannulated patients show better survival rate than not decannulated. VAP occurred during IMCU stay seems correlate with decannulation failure but future studies are necessary in this field.

**References**

1. Vincent JL, Rubenfeld GD. Does intermediate care improve patient outcomes or reduce costs? Crit Care 2015

Abbreviations: TBS, tracheobronchial secretions; RD, respiratory drive; AKI, acute kidney injury; HD, hemodialysis

**Table 78 (abstract A1090).** Patients characteristics

Characteristics	IMCU Patients (n = 57)
Male gender, n (%)	40/57 (70,2 %)
Median age (years)	73 (45–89)
Median weight (kg)	74 (45–108)
Previous ICU stay (days)	22 (3–67)
IMCU stay (days)	20 (1–74)
Median SAPS 2 score at IMCU admission	37 (19–94)
Median SOFA score at IMCU admission	4 (1–11)

**Table 79 (abstract A1090).** Comorbidities

Comorbidities	IMCU Patients (n = 57)
Cardiovascular, n (%)	35/57 (61,4 %)
Diabetes, n (%)	22/57 (38,6 %)
Chronic Obstructive Pulmonary Disease, n (%)	18/57 (31,6 %)
Tumors, n (%)	7/57 (12,3 %)
Cronic Kidney Disease, n (%)	7/57 (12,3 %)
Cirrhosis, n (%)	6/57 (10,5 %)

**Table 80 (abstract A1090).** Causes of admission in ICU

Causes of admission in ICU	IMCU Patients (n = 57)
Acute respiratory failure, n (%)	32/57 (56,1 %)
Neurological, n (%)	9/57 (15,8 %)
Major surgery, n (%)	5/57 (8,8 %)
Cardiac arrest, n (%)	4/57 (7 %)
Septic shock, n (%)	3/57 (5,3 %)
Trauma, n (%)	3/57 (5,3 %)
Hemorrage, n (%)	1/57 (1,7 %)

### A1091 Patient perceptions of physiotherapy in ICU: a qualitative study

#### M. van Nes^1^, F. Karachi^1,2^, S. Hanekom^3^

##### ^1^Stellenbosch University, Stellenbsoch, South Africa; ^2^University of the Western Cape, Cape Town, South Africa; ^3^Stellenbosch University, Physiotherapy, Stellenbsoch, South Africa

###### **Correspondence:** S. Hanekom – Stellenbosch University, Physiotherapy, Stellenbsoch, South Africa

**Introduction:** Physiotherapy practice in the intensive care unit (ICU) is changing. Early mobilisation programmes are prioritised and included. Understanding and investigating patient perception and satisfaction with regard to healthcare is vital in both the assessment and improvement of quality of care.

**Objectives:** To describe patient perceptions and satisfaction regarding the physiotherapy care received during their surgical ICU stay.

**Methods:** An interpretive and descriptive qualitative design was used. Patients were included via purposive sampling. Audiotaped, semistructured interviews of 25/60 minutes each were completed within 3 days of discharge from ICU. Data was transcribed and analysed via inductive and interpretive content analysis. Trustworthiness of results was ensured through reflexivity, checking of transcriptions, peer review and member checking.

**Results:** Eighteen patients, ten males, were purposefully selected for the primary study. The median patient age was 44 years and the median ICU length of stay was six days. Twelve themes arose from the data analysis. The importance of clear communication was highlighted. Communication and understanding acted as bridging factors to link the patient´s expectations and the comprehension of physiotherapy. Communication reassured the patients and aided them in knowing what to expect during physiotherapy activities and sessions. This reassurance and communication also assisted in making the patients feel comfortable, thus building a trustworthy relationship with the physiotherapists. Factors that would have decreased satisfaction with the physiotherapy care received were predominantly linked to pain and ultimately poor communication. Most patients found mobilisation to be a positive experience and the beginning of their recovery. However, mobilization was described as a difficult component of the care, mainly due to pain, tiredness and dizziness. Almost all patients commented on the benefits of participation in physiotherapy, which was verified by physical improvements and progression in their abilities. Although most improvements discussed were physical, two patients also described the psychological benefits that occurred in the sessions. They reported that the physiotherapists 'built them up' and encouraged them. One patient described a mind shift that occurred once she had mobilised out of the bed. She described it as being able to see what she was capable of. It was described as a precious and much-needed service, without which some patients felt they may not have survived or recovered as quickly.

**Conclusions:** Physiotherapy in ICU is a valuable experience predominantly perceived positively and satisfactorily by patients. Patient perception and satisfaction in the ICU can be used to ensure quality of care.

**Grant acknowledgment**

MRC-SA NRF-SA

### A1092 Impact mobilize early in critical patients admitted in ICU

#### A.H. Andrade, U.V. Pereira, C.A.C. Abreu Filho, R.C. Costa

##### Hospital Municipal Moyses Deutsch, ICU, Sao Paulo, Brazil

###### **Correspondence:** A.H. Andrade – Hospital Municipal Moyses Deutsch, ICU, Sao Paulo, Brazil

**Introduction:** Authors report that more than one million patients per year require invasive mechanical ventilation considering the evidence of morbidity, mortality and high costs generated in the hospitalization of these patients. In many hospitals in developed countries, physiotherapy and seen as part of the treatment of patients in the ICU, 30 years early mobilization has been shown to reduce the time to weaning from ventilation and is the basis for functional recovery.

**Objectives:** To compare the two groups evaluated the time spent on mechanical ventilation after 48 of hospitalization and number of calls received physical therapy in patients with early mobilization (CMP) with the group without early mobilization (SMP)

**Methods:** This was a retrospective longitudinal study by collecting data from the records of patients treated in the ICU of the Hospital Municipal adult. Participants were patients undergoing mechanical ventilation for 48 hours, were admitted to the ICU between June 29 to August 31, 2012 and January 1 to February 28, 2013 in all clinical situations that characterize stable evolution. With exclusion were patients admitted to the ICU outside the study period, we did not stay for at least 48 h in VMI and clinical instability.

**Results:** The study showed effectiveness in the use of the protocol reducing time VM. The SMP group had an average of 26.7 days while in IMV CMP group had an average of 10.16 days in VMI. We also observed a decrease in the quantity of care SMP group had an average of 73.07 calls while the group CMP 16.48 physiotherapy service.

**Conclusions:** Given the above I am clear that the CMP group had significant values for the proposal that was employed, where we can see the decrease in time to VMI, decrease in number of visits.

**References**

1. INOUE.C, Kelly et al. Dimensionamento da equipe de enfermagem da UTI-adulto de um hospital ensino -Rev. Eletr. Enf. [Internet]. 2009;11(1):55–63

2. CALDEIRA.M.H, Vanessa et al. Critérios para admissão de pacientes na unidade de terapia Intensiva e Mortalidade. Rev. Assoc. Med. Bras. vol.56 no.5 São Paulo 2010

**Grant acknowledgment**

No conflicts of concern

### A1093 Sharps containers clinical waste in a cardiac intensive care

#### M.S.W. Parkin, M. Moore

##### St Georges NHS Foundation Trust, Cardiothoracic Intensive Care, London, United Kingdom

###### **Correspondence:** M. Moore – St Georges NHS Foundation Trust, Cardiothoracic Intensive Care, London, United Kingdom

**Introduction:** A review of sharps containers clinical waste in a Cardiac Intensive Care of a one-week period.

**Objectives:** To weight all sharps containers in a Cardiac Intensive Care over a one-week period. To then review the financial implications of the cost of this waste. To review ways to redistribute this waste.

**Methods:** All closed sharps containers were weighed.

Research the cost implications of different waste types from the Waste Management team within the Trust.

Look into ways of reducing this waste.

**Results:** A total of 62 items where weighed, totaling 123.82 kg, costing £65.26. This can be broken down in six 55 L Chest Drain boxes, weighing 53.98 kg, costing £28.45. Four 22 L sharps bin, weighing 23.04 kg, costing £12.14. 13 7 L sharps bin, weighing 11.62 kg, costing £6.12, and thirty-nine 5 L sharps bins, weighing 35.18 kg, costing £18.54. This would take eight weeks to produce a tonne. Producing around 6.5 tonnes a year costing £3,425.50.

Compared to other types of waste:

Sharps Containers: £527 per tonne

Clinical Waste: £303 per tonne

Domestic Waste: £104 per tonne

Recycling: £30 per tonne

Clinically objects that aren't sharps are placed in sharps bins, for example arterial blood gas syringes, this item could be put into a clinical waste bin. While this suggests education is needed there are other methods to reduce Sharps Bin wastage. These include: having needle only sharps bins, and solidifying chest drain bottles post removal.

**Conclusions:** This Cardiac Intensive Care Unit produces a volume of high-cost waste. A large proportion of this waste can be re-distributed to other types of waste. This could make the unit more efficient, and reduce it's environmental burden. This audit suggests that it should be looked at other types of waste and other departments in the same manner on other units.

### A1094 Successful strategy to reduce infection catheter related bloodstream infections in patients on hemodialysis

#### A.H. Andrade, R.C. Costa, K.V. Silva Carvalho, C.A.C. Abreu Filho

##### Hospital Municipal Moyses Deutsch, ICU, São Paulo, Brazil

###### **Correspondence:** A.H. Andrade – Hospital Municipal Moyses Deutsch, ICU, São Paulo, Brazil

**Introduction:** The bloodstream infections related to the use of central venous catheter (CVC-ICS) are among the leading causes of morbidity and mortality of the Adult Intensive Care Unit patients worldwide, increasing the time and cost of hospitalization. Due to high rate of bloodstream infection related to the use of central venous catheter in the adult intensive care unit was necessary to process review.

In 2014 the bloodstream infection density related to hemodialysis catheter in the Hospital Moyses Deutsch was 13.7, which represents patient 16 in its entirety. Due to this high rate, it was necessary to review all related process through institutionalized and supervised practice, minimizing the risks of hemodialysis procedure and maintenance of the catheter, in order to directly reduce the length of hospital stay, morbidity and hospital costs.

**Objectives:** Objective of this study was to evaluate the application of strategies according to IHI to reduce infection of the bloodstream related to CVC hemodialysis.

**Methods:** The study was conducted in a 20-bed, medical-surgical ICU. Criteria for infection catheter related bloodtream infection are those from the CDC. Strategy was to implement the Permanent education of employees, highlighting the importance of prevention of infections;Training of new employees as the hemodialysis routine and safe and aseptic techniques; Optimize other measures that can reduce the risks, such as early removal of invasive devices.Raise awareness of nursing staff about the importance of their role in the prevention of infection, such as maintenance of the catheter with use of aseptic techniques; Disseminate monthly for teams infection rates; Make benchmarking with other services; The goals were the ICU team adhesion of 46 % achieved in six month after bundle implementation and 100 % after one year of follow up. From june 2012 on, the ICU nursing staff and ICT performed a daily checklist in order to observe the issues accomplishment. If any item was found to be inadequate it was promptly corrected.

**Results:** In June and December 2012 , adhesion to the whole bundle was 46 % and 100 % respectively, infection of the bloodstream related to CVC hemodialysis density was proportionally lower to bundle adhesion in the same period, in 2015 we not had any cases of bloodstream infection related to hemodialysis catheter.

**Conclusions:** Continuing education and staff training are essential to achieve good results and zero number bloodstream infection related to hemodialysis catheter**.** Initial rates were extremely high even for Brazilian benchmarks, ICU team and ICT efforts were crucial for satisfactory results, as well the administrative board support, which turned this issue an institutional priority. Our goals are to reduce even more, implementing getting to zero´´ program, maintaining a continuum effort to sustain these results.

### A1095 Comparison between patient acuity rating and modified early warnings score

#### H.J. Min^1^, H.J. Kim^1,2^, D.S. Lee^1^, Y.Y. Choi^1^, E.Y. Lee^1^, I. Song^1,3^, D.J. Kim^1^, Y.Y. E^2^, J.W. Kim^2^, J.S. Park^2^, Y.J. Cho^1,2^, J.H. Lee^1,4^, J.W. Suh^2^, Y.H. Jo^1,4^, K.S. Kim^1,4^, Y.J. Lee^1,2^

##### ^1^Seoul National University Bundang Hospital, Critical Care Medicine, Seongnam, Republic of Korea; ^2^Seoul National University Bundang Hospital, Internal Medicine, Seongnam, Republic of Korea; ^3^Seoul National University Bundang Hospital, Anesthesiology, Seongnam, Republic of Korea; ^4^Seoul National University Bundang Hospital, Emergency Medicine, Seongnam, Republic of Korea

###### **Correspondence:** H.J. Min – Seoul National University Bundang Hospital, Critical Care Medicine, Seongnam, Republic of Korea

**Introduction:** Despite recent highlight on rapid response systems, current vital sign based scoring have demonstrated limited accuracy predicting hospital mortality and intensive care unit (ICU) admission. Many other prediction models were proposed, however, they are difficult to perform in other facilities.

**Objectives:** Our aim is to propose a more intuitive method predicting the risk among ward patients.

**Methods:** In this retrospective cohort study from January 2015 to March 2016, patients were screened with automated alarm of abnormalities in vital sign and laboratory results. Four well trained, ICU-specialized RRT Nurses visited each patient randomly, and the patients were scored 1 to 7 according to patient acuity rating (PAR). Modified Early Warning Score (MEWS) was calculated simultaneously. ICU admission, mortality, and the composite outcome within 7 days of evaluation were analyzed. PAR and MEWS were compared using the receiver operating characteristic (ROC) curve.

**Results:** Total of 1,134 cases was screened in this study. Within the 7 days of RRT Nurses visit, overall ICU admission, mortality, and the composite outcomes were 231 (20.4 %), 106 (9.3 %), and 310 (27.3 %) respectively. PAR more accurately predicted all outcomes compared to MEWS (area under the receiver operating characteristics curve, 0.754 vs 0.670 for ICU admission; 0.761 vs 0.635 for mortality; and 0.788 vs 0.668 for composite outcomes; P < 0.001 for all comparisons).

**Conclusions:** PAR is simpler and more accurate than MEWS. Implementation of this tool may decrease unexpected in-hospital ICU admission and mortality. Further studies comparing PAR with other objective prediction models will be complementary.

**References**

1. Dana P. Edelson, MD, MS, Elizabeth Retzer, MD, Elizabeth K.Weidman, BA, James Woodruff, MD, Andrew M. Davis, MD MPH, Bruce D. Minsky, MD, William Meadow, MD, PhD, Terry L. Vanden Hoek,MD, David O. Meltzer, MD, PhD. Patient Acuity Rating: Quantifying Clinical Judgment Regarding Inpatient Stability. *Journal of Hospital Medicine. 2011;6:475–479.*

2. Jeroen Ludikhuize MDa, Susanne M. Smorenburg MD, PhDa, Sophia E. de Rooij MD, PhDb, Evert de Jonge MD, PhDc. Identification of deteriorating patients on general wards; measurement of vital parameters and potential effectiveness of the Modified Early Warning Score. *Journal of Critical Care*(2012) 27, 424.e7-424.e13

**Grant acknowledgment**

We would like to thank the rapid response team, Intensive care unit members and general ward members for their enthusiasm and commitment to patient care.

**Keywords.** hospital rapid response team, critical care, intensive care units.

### A1096 Chronic critical illness: a growing fact in the intensive care setting

#### J. Ferrero-Calleja, D. Merino-Vega, A.I. González-Jiménez, M. Sigcha Sigcha, A. Hernández-Tejedor, A. Martin-Vivas, Á. Gabán-Díez, R. Ruiz-de Luna, N. De la Calle-Pedrosa, I. Temprano-Gómez, D. Afonso-Rivero, J.I. Pellin-Ariño, A. Algora-Weber

##### ^1^Intensive Care Unit, Hospital Universitario Fundación Alcorcón, Madrid, Spain

###### **Correspondence:** J. Ferrero-Calleja – Intensive Care Unit, Hospital Universitario Fundación Alcorcón, Madrid, Spain

**Introduction:** The evolution of the techniques used in the intensive care over the past decades has led to better survival rates in patients with acute conditions and severely impaired vital functions. However, it has resulted in a growing number of patients who survive an acute event but who then become dependent on one or more life support techniques. Such patients are called chronically critically ill (CCI) patients. No categorical definition of the disease is currently available, although most patients are characterized by the need for prolonged mechanical ventilation.

**Objectives:** The purpose of our study is to analyze the characteristics of patients requiring prolonged support with slow recovery, as defined by terms of intensive care unit (ICU) stay and need for prolonged mechanical ventilation requiring tracheostomy.

**Methods:** This retrospective study was performed in a 12-bed medical ICU in Spain from 2011 to 2015. All patients admitted to the ICU during this period were included in the study. CCI patients were defined as those with more than 60 days of ICU stay. Data were collected in 3 ways: review of a prospectively elaborated database, review of electronic records, and telephone survey evaluating the functional status of survivors, one year after their discharge from the ICU.

**Results:** During the study period, 46 patients (9 females −20 %) were considered CCI. The characteristics of these patients are shown in Table 82. All the studied patients needed prolonged mechanical ventilation (median 66 days), defined as >6 hours/day of ventilator support for > 21 consecutive days.

**Fig. 115 (abstract A1096).**
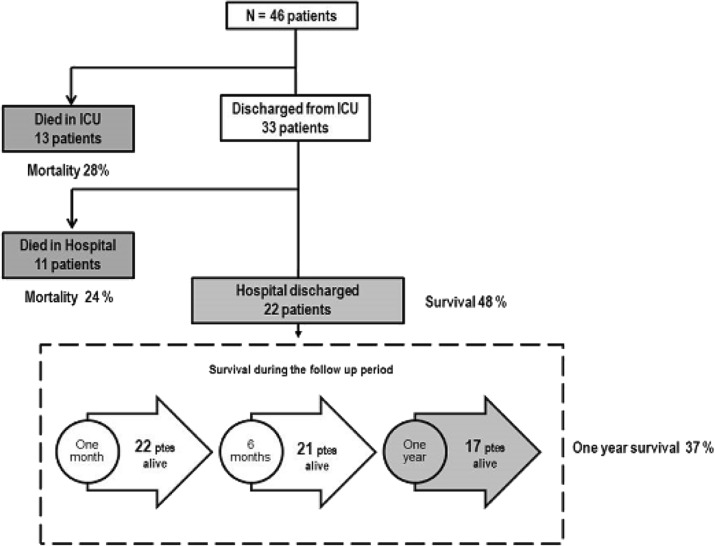
Enrolment and follow-up of patients

The follow-up period is drawn in Figure 115. The in-ICU mortality was 28 %. In the first year, 17 patients (37 %) were alive. Most patients improved their quality of life over a year, with approximately 40 % of them displaying some help for dressing or to performing the transfer of themselves. Symptoms of anxiety and depression improved during the first year, being present in up to 20 % (of the patients), but in 6 % if we refer to the presence of nightmares or hallucinations. 41 % these patients were transferred (discharge) to a rehabilitation center and 47 % needed hospital readmission within the follow-up period.

**Conclusions:** For CCI patients in-hospital mortality rate is still high after discharge from the ICU. However, more than one third of them are alive one year after their Hospital stay and in an almost independent condition. Efforts focused on early specific therapeutic strategies after ICU admission to prevent the progress of the acute disease towards chronic critical illness and to improve the outcome must be explored.

**Table 81 (abstract A1096).** Characteristics of patients with ICU. Median (IQR)

Age (years)	69 (66–75)
LOS (days)	
ICU	84 (69–108)
Hospital (hospital admission to discharge)	101 (88–161)
Admission source	
Hospital general Ward(%)	27 (59)
Emergency department(%)	17 (37)
Other facility (%)	2 (4)
Duration of mechanical ventilation (days)	66 (54–84)
Period with tracheostomy (days)	49 (31–76)

### 1097 Assessment of chronically critically ill patients admitted to the ICU and the impact on their family members

#### R.R.L. Fumis^1^, A.B. Ferraz^2^, J.M. Vieira Junior^3^

##### ^1^Hospital Sírio-Libanês, Intensive Care Unit, São Paulo, Brazil; ^2^Hospital Geral do Grajau, Intensive Care Unit, Sao Paulo, Brazil; ^3^Hospital Sírio Libanes, Intensive Care Unit, São Paulo, Brazil

###### **Correspondence:** R.R.L. Fumis – Hospital Sírio-Libanês, Intensive Care Unit, São Paulo, Brazil

**Introduction:** The chronically critically ill (CCI) comprise a growing population of patients who have survived acute critical illness. There are few studies about their characteristics.^1^ The uncertain trajectory of CCI exposes the patient´s family to heightened levels of psychological distress.^2^

**Objectives:** To assess the CCI Patients and their family members admitted to the Intensive Care Unit and identify their conditions during stay at ICU and at 30 post-ICU.

**Methods:** A prospective study was conducted in a 22-bed adult general ICU. Patients were defined as CCI if they were at least 8 days in ICU plus one of six eligible clinical conditions: Mechanical ventilation for at least 96 hrs; tracheotomy; sepsis; severe wounds; stroke; traumatic brain injury.^1^ Family members completed the Hospital Anxiety and Depression Scale (HADS) at ICU and 30-days after interview and respond the EQ-5D and KATZ index of patients.

**Results:** Between May 2015 and March 2016, 66 patients fulfilled the criteria. Of the total, 62.1 % of patients had more than one criterion for CCI. Sepsis was the most common eligible condition (74.2 %) followed by mechanical ventilation (65.2 %). The mean of length of stay at ICU was 17.75 ± 11.25 days and 48.86 ± 36.73 days in hospital. The ICU mortality was 13.1 % and 30-days cumulative mortality was 30.3 %. The median of SAPS 3 was 54.5 [42.0-64.0]; median of SOFA was 6.00 [2.75-8.00], Glasgow was 14 and median of charlson was 2.00 [1–4]. At ICU, 40.9 % had delirium, 40.9 % needed blood transfusion and 18.2 % renal replacement therapy. Patients worsened in all parameters of the five dimensions of the EQ-5D after 30-days: The extreme problems level increased in the mobility dimension from 12.1 % at ICU to 42.1 %, self-care from 25.8 % to 52.6 %; usual activities from 19.7 % to 68.4 %; pain/discomfort from 7.6 to 13.2 and anxiety/depression from 4.5 % to 7.9 %. The dependence observed in the KATZ index worsened in 30 days when 28.8 % of patients were dependents before ICU admission increasing to 44.7 % after 30 days. About family members, 42.4 % were spouses and 39.4 offspring, their mean of age was 55.89 ± 13.69 years and 84.8 % had previous experience of ICU. We observed that they presented more symptoms of anxiety (50 %) and depression (27.3 %) at ICU when compared 30 days after (21.1 %) and (13.2 %), symptoms of anxiety and depression respectively.

**Conclusions:** The most common eligibility conditions of CCI were sepsis followed by mechanical ventilation. We observed a great mortality on 30 days and among survivors a worsen quality of life with more dependence in their Activities of Daily Living. We also observed that family members suffered more while in ICU stay.

**References**

1. Kahn JM, Le T, Angus DC, et al. The epidemiology of chronic critical illness in the United States. Crit Care Med. 2015;43:282–7.

2. Hickman RL Jr, Douglas SL. Impact of chronic critical illness on the psychological outcomes of family members. AACN Adv Crit Care. 2010;21:80–91.

### A1098 Tracheostomy experience at a turkish university hospital ICU

#### H. Kirca, O. Cakin, M. Unal, H. Mutlu, A. Ramazanoglu, M. Cengiz

##### Akdeniz University, Antalya, Turkey

###### **Correspondence:** H. Kirca – Akdeniz University, Antalya, Turkey

**Introduction:** Tracheostomy is a favored alternative option for providing prolonged mechanical ventilation and safety airway used for more than 50 years. Despite its numerous advantages, tracheostomy may have severe complications as being an invasive method for presenting respiratory tract patency. Besides, the tracheostomized patients usually have prolonged ICU stay, high mortality and morbidity arise from concomitant comorbidities.

**Objectives:** The aim of the study was to evaluate the frequency, patient characteristics, complications and the prognosis related with our percutaneous tracheostomy practice.

**Methods:** Hospital electronic records and ICU files of the patients with percutaneous tracheostomies performed in our 34 bed anesthesiology ICU were evaluated between January 2010 and December 2014. Ethic consent was obtained from local ethic committee. The patients who were discharged with home type mechanical ventilator or their relatives were contacted by phone for getting information about their health status or related complications. Demographic variables of the patients, ICU admission and tracheostomy indications, intubation history, method for tracheostomy procedure, ICU stay or tracheostomy duration, decannulation and/or recannulation status, early and late complications were recorded.

**Results:** There were 442 patients who underwent tracheostomy during their ICU stay in 5 year. Mean age and mortality of the patients were 56 and 55.2 % respectively. Tracheostomy procedures were performed surgically in 29 patients. Mean tracheostomy performance day after ICU admission was 9.9 days. Tracheostomy incidence performed at 7. day or before, between 8 and 20 days and at 21. day or later after ICU admission were 181 (41 %), 250 (54,3 %) and 21 (4.8 %), respectively. Overall complication incidence was 7 % and the major complication was bleeding.

**Conclusions:** The results of our study were in accordance with previous studies investigating tracheostomy timing and complication rates^1–4^. However, the mortality was higher in our study group. The main reason for high mortality may result from the fact that end of life decision is not supported by law in our country. For the reason we performed percutaneous tracheostomy to all patients who were highly predicted to die.

**References**

1. Vargas M, Sutherasan Y, Antonelli I, Brunetti I, Corcione A, Laffey JG, Putensen C, Servillo G, Pelosi P. Tracheostomy procedures in the intensive care unit: an international survey. Crit Care. 2015; 13;19:291.

2. Dempsey GA, Grant CA, Jones TM. Percutaneous tracheostomy: A 6 yr prospective evaluation of the single tapered dilator technique. Br J Anaesth. 2010;105:782–8.

3. D.R. Gerber, A. Chaaya, C.A. Schorr, et al. Can outcomes of intensive care unit patients undergoing tracheostomy be predicted? Respir Care, 54 (2009), pp. 1653–1657

4. Diaz-Reganon G, et al. Safety and complications of percutaneous tracheostomy in a cohort of 800 mixed ICU patients. Anaesthesia 2008; 63: 1198–203

### A1099 The fate of disnatremias in surgical critically ill patients prognosis

#### E.A. Nicolini^1^, F.G.F. Pelisson^2^, R.S. Nunes^2^, S.L. da Silva^2^, M.M. Carreira^2^, F. Bellissimo-Rodrigues^3^, M.A. Ferez^2^, A. Basile-Filho^1^

##### ^1^Ribeirão Preto Medical School, University of São Paulo, Division of Intensive Care, Department of Surgery and Anatomy, Ribeirão Preto, Brazil; ^2^Intensive Care Unit of São Francisco Hospital, Ribeirão Preto, Brazil; ^3^Ribeirão Preto Medical School, University of São Paulo, Social Medicine Department, Ribeirão Preto, Brazil

###### **Correspondence:** S.L. da Silva – Intensive Care Unit of São Francisco Hospital, Ribeirão Preto, Brazil

**Introduction:** The dysnatremias (hypo and hypernatremia) are associated with increased mortality of critically ill patients. Literature suggests that serum sodium level imbalances on admission to the intensive care unit (ICU) may lead to a poor survival rates. The objective of this study was to evaluate the ability of serum sodium levels on admission and prognostic indexes to predict mortality of surgical critically ill patients.

**Methods:** One thousand five hundred and ninety-nine surgical patients (57 % males and 43 % females; mean age of 60.6 ± 14.4 years admitted to the ICU in the post-operative phase were retrospectively studied. The patients were classified according to the blood serum sodium levels (mmol/L) at admission as normonatremia (135–145), hyponatremia (<135) and hypernatremia (>145). Therefore, APACHE II, SAPS III and SOFA were recorded. The capability of each index to predict relative risk of ICU and Hospital mortality of surgical patients was analyzed by multiple logistic regression analysis. Relative Risk (RR) and 95 % confidence interval (CI) were calculated.

**Results:** The mean of APACHE II, SAPS III and SOFA were 13.9 ± 7.9, 39.6 ± 10.9, 4.8 ± 2.5, respectively. The overall ICU mortality was 7.1 %. RR for APACHE II at admission was 1.08 (1.02-1.15), SAPS III 1.03 (1.00-1.05) and SOFA 1.36 (1.21-1.53). RR for hypernatremia and hyponatremia were 2.37 (1.09-5.12) and 0.04 (0.44-2.03), respectively (Tables 83 and 84).

**Conclusions:** The blood serum sodium levels at admission, especially hypernatremia, may also be used as an independent predictor of outcome in the surgical critically ill patients.

**References**

1. Darmon M, Diconne E, Souweine B, et al. Prognostic consequences of borderline dysnatremia: Pay attention to minimal serum sodium change. Crit Care. 2013 Jan;21:17(1):R12.

2. Basile-Filho A, Menegeti MG, Nicolini EA, et al. Are the dysnatremias a permanent threat to the critically ill patient? J Clin Med Res. 2016 Feb;8(2):141–146.

**Table 82 (abstract A1099).** ᅟ

	ICU Mortality Relative Risk	ICU Mortality CI	Hospital Mortality Relative Risk	Hospital Mortality CI
Gender	0.98	0.56–1.72	1.14	0.73–1.79
Age	1.01	0.99–1.03	1.01	1.00–1.03
Apache II	1.08	1.02–1.15	1.07	1.02–1.13
SAPS III	1.03	1.00–1.05	1.04	1.02–1.06
SOFA escore	1.36	1.21–1.53	1.19	1.08–1.30
Hypernatremia	2.37	1.09–5.12	2.62	1.34–5.12

**Table 83 (abstract A1099).** ᅟ

	ICU Mortality Relative Risk	ICU Mortality CI	Hospital Mortality Relative Risk	Hospital Mortality CI
Gender	1.11	0.63–1.94	1.09	0.70–1.69
Age	1.01	0.99–1.03	1.02	1.00–1.35
Apache II	1.19	1.05–1.18	1.08	1.03–1.14
SAPS III	1.03	1.01–1.06	1.04	1.02–1.06
SOFA escore	1.26	1.12–1.41	1.17	1.06–1.28
Hyponatremia	0.04	0.44–2.03	1.34	0.74–2.43

### A1100 Explore the dengue-related risk factors and death factors in Taiwan

#### H.-C. Chao, C.-M. Chen

##### Chi Mei Medical Center, Intensive Care Medicine, Tainan, Taiwan, Province of China

###### **Correspondence:** H.-C. Chao – Chi Mei Medical Center, Intensive Care Medicine, Tainan, Taiwan, Province of China

**Introduction:** WHO estimates that the worldwide dengue fever incidence is about tens of thousands of cases every year. As Taiwan is situated in the high risk subtropical region, dengue fever has virtually become a seasonal infectious disease. Climate warming, demographic movement and the higher probability of increase in intermittent rainfall in recent years have added many factors unfavorable to dengue fever prevention. Years of prevalence and the emergence of different types have also caused the risk of mortality for dengue fever to become relatively high.Of the total 37,224 confirmed dengue fever cases in 2015, there were 174 deaths (with a mortality rate of 4.7 per thousand), marking the largest outbreak over nearly one decade in Taiwan.

**Objectives:** Analysis was conducted on the 93 confirmed severe cases of dengue fever or dengue hemorrhagic fever reported to this hospital over the period between July 20 and September 30, 2015 in terms of gender, age, history of chronic diseases, warning signs and diagnostic criteria for severe conditions.

**Methods:** Retrospective case study was also conducted to identify risk factors in dengue fever and dengue hemorrhagic fever as well as predictors of death among dengue fever cases for statistical analysis.

**Results:** According to the results, those susceptible to infection concentrated on older people aged over 65 (with an average age of 68); in total 73 cases had chronic diseases (with an average rate of 78.5 %), among which hypertension and diabetes constituted the majorities; and based on symptoms, fever accounted for 83.51 % while gastrointestinal bleeding was the most common at 38.7 %. Of the 93 cases, there were 18 deaths, with an average APACHE II score of 17.73 and an average mortality rate of 19.8 %.

**Conclusions:** This study shows that patients with chronic diseases aged over 65 will have 10 times higher risk of death if infected with dengue hemorrhagic fever. It is therefore suggested that older people aged over 65 and patients with chronic diseases who are infected with dengue hemorrhagic fever must be closely monitored in clinical practice to pinpoint the best time for treatment and effectively reduce mortality rates. To sum up, effective use of knowledge about risk factors and prognostic factors in dengue hemorrhagic fever can help epidemic prevention organizations to focus their limited resources on high risk groups and increase the effectiveness of prevention.

**References**

1. Chao DY,Lin TH,Hwang K P.1998 dengue hemorrhagic fever epidemic in Taiwan. Emerg Infect Dis,10,552-4.

2. Pang J, Salim A, Lee VJ, et al. Diabeted with Hypertension as Riak Factor for Adult Dengue Hemorrhagic Fever in a Predominantly Dengue Serotype 2 Epidemic: A Case Study. PLoS Negl Trop Dis. 2012; 6(5):e 1641 1–8.

3. Chang SF, Huang JH, Shu PY. Characteristics of dengue epidemics in Taiwan. Journal of the Formosan Medical Association 2012; 111:297–9.

### A1101 Cardiorespiratory instability risk escalation patterns: an association study with risk factors and length of stay

#### L. Chen^1^, M. Hravnak^2^, G. Clermont^3^, M. Pinsky^3^, A. Dubrawski^1^

##### ^1^Carnegie Mellon University, Robotics Institute, Pittsburgh, United States; ^2^University of Pittsburgh, School of Nursing, Pittsburgh, United States; ^3^University of Pittsburgh, Department of Critical Care Medicine, School of Medicine, Pittsburgh, United States

###### **Correspondence:** L. Chen – Carnegie Mellon University, Robotics Institute, Pittsburgh, United States

**Introduction:** We previously discovered that patients in step down units (SDUs) who developed cardiorespiratory instability (CRI) may follow several distinct risk escalation patterns based on a risk score derived from multi-parameter vital sign (VS) from bedside monitors.

**Objectives:** To assess the association between risk escalation patterns and baseline demographics as well as length of stay (LOS).

**Methods:** Data were collected from 1971 patients admitted to a SDU, including 918 patients who developed at least one episode of CRI. Demographics were collected from the electronic health record (EHR). Continuous bedside monitoring data stream includes heart rate, respiratory rate, and SpO_2_ collected at 1/20 Hz, and intermittently recorded systolic and diastolic blood pressure. We discovered distinct relative risk (RR) escalation patterns among 918 CRI positive patients during the 4 hours immediately before CRI onset, following methods in [1]. We then tested for association of RR escalation patterns with the mean risk levels during the first 4 post-SDU admission hours, and independently with demographics (age, Charlson Comorbidity Index [CCI]), and SDU and hospital LOS) using ANOVA at 0.05 significance level.

**Fig. 116 (abstract A1101).**
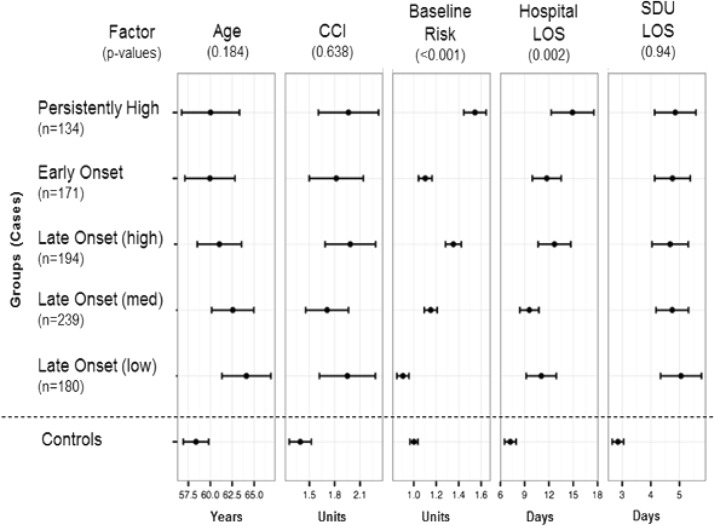
ᅟ

**Results:** We identified 5 RR escalation patterns among 918 CRI positive patients (Fig. 116). 66 % of them belong to “late onset” types whose risk escalated ≤30 minutes before CRI onset, but with different initial RR levels (low, medium and high). 19 % of patients belonged to “early onset” type with gradual escalating risk starting about 3 hours before overt CRI, and 15 % falling into a “persistently high” type. The mean RR during the first 4 hours of SDU stay are 0.9, 1.15 and 1.35 for “late onset” types; 1.10 for “early onset” type, and 1.55 for “persistently high” type, comparing with baseline RR of 1.0 for CRI negative patients. The mean RR derived in the first 4-hours after admission is strongly associated with risk escalation patterns observed (p-value < 0.001), specifically, patients of “persistently high” type were more likely to have higher mean risk levels at SDU admission . Risk escalation patterns were not significantly associated with age, CCI or SDU LOS. However, they are significantly associated with hospital LOS (p = 0.002).

**Conclusions:** There is potential “risk stratification value” of VS collected during initial hours of SDU stay in predicting the CRI risk escalation patterns later on, which may in turn predict hospital LOS. These insights may guide monitoring resource allocation for CRI management.

**References**

[1] Chen L, Dubrawski A, Clermont G, Hravnak M, Pinsky MR. Modelling Risk of Cardio-Respiratory Instability as a Heterogeneous Process. AMIA Annu Symp Proc. 2015 Nov 5;2015:1841–50.

**Grant acknolwedgment**

NIH NINR R01NR013912; NSF 1320347.

### A1102 Survival and functional status after a year from prolonged admission in ICU

#### J. Luján Varas, R. Molina Montero, L. Alcázar Sánchez-Elvira, P. Villa Díaz, C. Pintado Delgado, B. Llorente Ruiz, A. Pardo Guerrero, J.A. Cambronero Galache

##### Hospital Universitario Príncipe de Asturias, Alcalá de Henares, Madrid, Spain

###### **Correspondence:** J. Luján Varas – Hospital Universitario Príncipe de Asturias, Alcalá de Henares, Madrid, Spain

I**Introduction:** Since it is increasingly common to find patients with prolonged ICU hospitalization (defined as greater or equal than 21 days), we conducted a study to assess the survival and functional status of these patients a year later.

**Objective:** Assessment of survival and functional status of patients with ICU hospitalization greater or equal than 21 days a year later.

**Methods:** Prospective and descriptive study. During two years, we included all patients with more than 20 days of stay in a medical-surgical ICU. Previous informed consent , we collected demographics data, baseline functional status (Barthel scale), mortality intraUCI, at hospital and one-year of hospital discharge. The monitoring was conducted by telephone or by personal interview to determine the functional status.

**Results:** A total of 48 patients with prolonged ICU admission were included. Survival to ICU admission is 62.5 % (30/48) and intra-hospital of 43.75 % (21/48). In the follow-up, survival at 12 months of hospital discharge is 31.25 % (15/48).

Regarding to functional status, it is observed that prolonged ICU admission conditioned an important and significant deterioration to functional level with a drastic drop in Barthel Index at hospital discharge compared with baseline Barthel index (p = 0.03). This deterioration is recovered significantly in the next months after hospital discharge, being more noticeable this recovery in the first 6 months (p = 0.04), with rates Barthel at 6 months close to baseline (p = 0.176) being less important subsequent recovery (p = 0.357).

**Conclusions:** One-year survival of patients who had prolonged ICU admission is low (31.25 %). It also represents a significant deterioration in functional status, which is recovered significantly in the next 6 months after hospital discharge.

**References**

1. Nelson JE, Cox CE, Hope AA, Carson SS. Chronic critical illness. Am J Respir Crit Care Med. 2010;182:446–454.

2. Lamas D. Chronic critical illness. N Engl J Med. 2014;370:175–177.

**Fig. 117 (abstract A1102).**
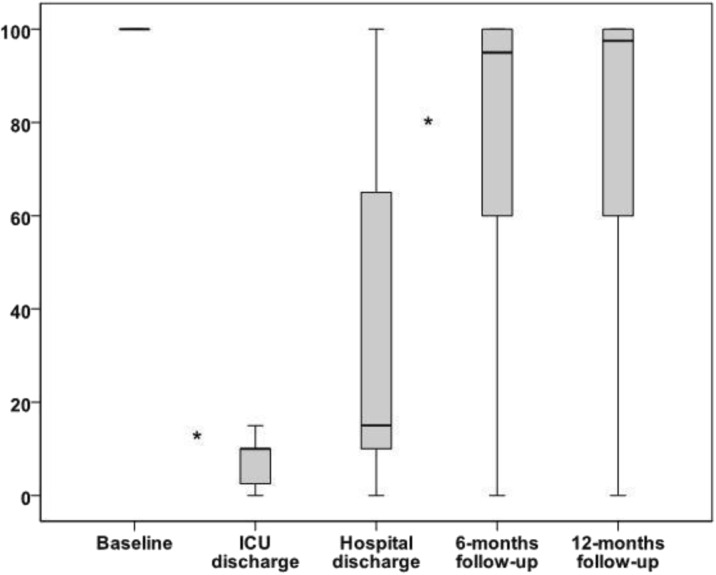
Barthel

**Fig. 118 (abstract A1102).**
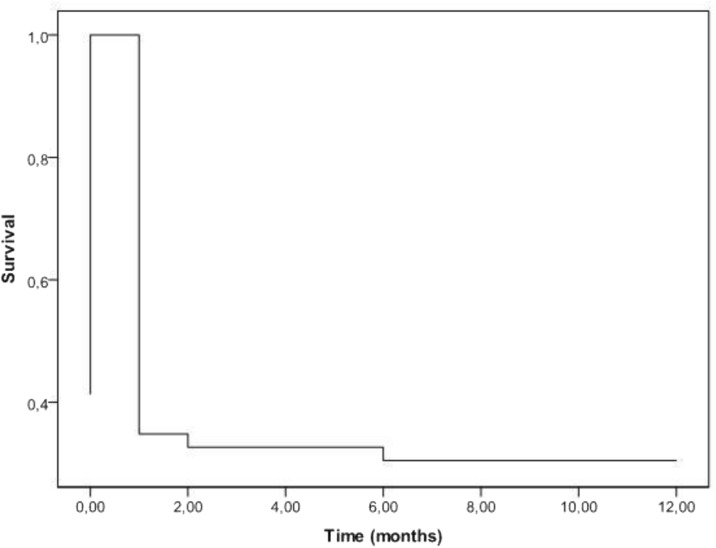
Survival

### A1103 Comparison between 2-dimentional speckle tracking & color-tissue doppler imaging for the assessment of left ventricular global longitudinal systolic strain and strain rate in outcome prediction of sepsis

#### H. Sherif, H. Hassanin, R. El Hossainy, W. Samy

##### Cairo University, Critical Care Medicine, Cairo, Egypt

###### **Correspondence:** H. Sherif – Cairo University, Critical Care Medicine, Cairo, Egypt

**Background:** Strain imaging by either tissue-Doppler imaging (TDI) velocity converted to strain and strain rate, or by digital 2-dimensional speckle tracking echocardiography (STE) analysis have been reported to assess abnormal left ventricular (LV) mechanical activation patterns in sepsis.

**Objective:** Comparison between both approaches of LV strain and strain rate in relation to the survival outcome in sepsis.

**Methods:** Thirty two patients (pts) (43.7 ± 13.7ys, 21 males); 13 pts with sepsis (group-1), 19 pts with severe sepsis/septic shock (group-2) and a subset of 10 controls (36.5 ± 8.7ys, 8 males) were included. APACHE II score was assessed on admission. In the first 24 hours, echocardiography-derived LV dimensions and ejection fraction (%EF) were recorded in sinus rhythm. Color-TDI was performed for 16 segments in the three LV apical views to acquire myocardial peak velocities during systole. Doppler flow profiles were reanalyzed using STE to retrieve LV peak global longitudinal systolic strain (GLSS) and strain rate (GLSSR) which were averaged for the whole segments.

**Results:** Compared to controls, %EF of both groups were comparable, but GLSS showed increased values (−17.5 ± 2.9 vs. -20.2 ± 1.6, *p* < 0.05 by STE, and −14.9 ± 2.6 vs. -19.7 ± 1.8, *p* < 0.001 by TDI) and for GLSSR values (−1.3 ± 0.2 vs. -1.6 ± 0.1, *p* < 0.001 by STE, and −1.1 ± 0.4 vs. -1.6 ± 0.1, *p* < 0.001 by TDI). Compared to group-1, %EF of group-2 showed significantly reduced values (63.1 ± 6.2 % vs. 69 ± 3.9 %, *p* < 0.05), but APACHE II score values were comparable. Compared to group-1, GLSS of group-2 showed increased values (−15.4 ± 1.5 vs. -20.2 ± 2.4, *p* < 0.05 by STE, and −12.7 ± 6.8 vs. -18.1 ± 2.4, *p* < 0.05 by TDI) but for GLSSR, the values were comparable. The total mortality was 31.3 % at 30 days for all pts (n = 10). Compared to the survivors, the non survivors showed lower APACHE II score values (13.1 ± 4.2 vs. 23.5 ± 5.4, *p* < 0.001), but %EF values were comparable. Good correlation could detected between APACHE II score and both STE GLSS and GLSSR values (*r* = 0.79, *p* < 0.001, and *r* = 0.66, *p* < 0.001), moderate correlation was found between %EF and STE GLSS and GLSSR values (*r* = 0.43, *p* < 0.05 and *r* = 0.54, *p* < 0.05), but with TDI the correlation was poor with either APACHE II score or %EF. The area under the curve (AUC) of STE GLSS to predict mortality was 0.9 (95 % CI; 0.32-0.48), with best cutoff value at −16.8 (sensitivity 100 %, specificity 86 %), AUC for STE GLSSR was 0.96 (95 % CI; 0.1-0.45), with best cutoff value at −1.2 (sensitivity 90 %, specificity 80 %), AUC for TDI GLSS was 0.76 (95 % CI; 0.1-0.44), with best cutoff value at −14.9 (sensitivity 100 %, specificity 82 %), and AUC for TDI GLSSR was 0.92 (95 % CI; 0.35-0.49), with best cutoff value at −1.2 (sensitivity 100 %, specificity 73 %).

**Conclusion:** LV global longitudinal systolic strain and strain rate by STE showed better correlation with both APACHE II and %EF than TDI. Both approaches showed sensitive prediction of mortality in sepsis, but STE approach was more specific.

## ADVANCES IN TRAUMA CARE AND CARDIAC ARREST MANAGEMENT

### A1104 Cardiac arrest in surgical theaters and coronary catheters suites. Incidence and outcome. A 7 years, single center study

#### H. Ly, H. David, P. Burtin, C. Charpentier, M. Barral, P. Courant

##### ^1^Clinique du Millénaire, Hérault, Montpellier, France; ^2^Clinique du Millénaire, Montpellier, France

###### **Correspondence:** H. Ly – Clinique du Millénaire, Hérault, Montpellier, France

**Introduction:** Peroperative cardiac arrests(PAC) are rarely studied(1–4).Incidence and outcome are influenced by numerous factors with overall survival ranging from 32 to 55 %.There is a lack of data for PAC occurring in angiographic and cardiac catheterism patients(PACANGIO) while they may obviously be at risk.Moreover, anesthetic care is not codified in these settings.

**Objectives:** We performed a retrospective, monocentric study comparing incidence and outcome of PAC occuring in surgical theatres(adult only; cardiac, abdominal and neurosurgery) and PACANGIO.

**Methods:** All IHCA(defined by presence of chest compression and/or EES) were recorded on an internal database through a dedicated follow-up form including demographic data, medical history, location, event time sequence of CPR initial cardiac rhythm(VF, VT, PEA, Asystole), first treatment attempt and immediate survival rate(ISR).Survivors of CPR were followed-up until hospital discharge and SAPS II score, duration of ICU stay, treatments applied and hospital survival rate(HSR) were recorded.2 groups were defined :PACSURG and PACANGIO and compared by CHI2 and student t tests.

**Results:** From 01/01/2009 to 12/31/2015, 414 PAC were recorded: 32 PACSURG (0,4/1000), 67 PACANGIO(1,5/1000).Baseline characteristics were similar in both groups except for a higher proportion of myocardial ischemia in the PACANGIO group(46,9 % vs 91 % p < 0,00001).Initial rhythms were predominantly shockable for PACANGIO(VT + VF: 62,6 %) and non-shockable for PACSURG(DEM + ASYSTOLE: 70 % p < 0.015).Time to ROSC was shorter for PACANGIO(4,8+/−7,5 vs 11,6+/−12,4 p = 0,001)(Table 85).SAPS II score for survivors was higher in PACSURG(52+/−10,6 vs 47,2+/−10,9 p = 0,041).ISR(75 % vs 68,7 % p = 0,51) and HSR(59,4 % vs 55,2 % p = 0,7) were similar in both groups(Table 86).Presence of pre anesthesia shock was the main cause in the PACSURG group(37.5 %). Anesthesia was directly the cause of 34 % of PACSURG events.62 % of the PACANGIO were due to myocardial ischemia.

**Conclusions:** Incidence and outcome of PACSURG are consistent with previous report.This is the first european study assessing incidence and outcome of PACANGIO.The coronary angiography suite has an incidence of PAC 4 times higher that of the surgical theatre.We believe that full BLS/ACLS education should be ensured in these settings.The PACANGIO group cumulates multiple known favorable outcome factors with no clear benefit on the observed HSR.This suggests the possible presence of specific risk factors in this group. The high incidence of PAC related to the anesthesia procedure could be decreased by implementation of a quality improvement program focused on detection of patients at risk and preventive care.The overall observed HSR suggest that OR and diagnostic intervention areas are no place for DNR orders in case of PAC.

**References**

1- Anesthesiology 2016 ; 124: 723–29.

2- Anesthesiology 2013; 119: 1322–39.

3- Br. J. Anesth 2006; 96: 569–75.

4- Anesthesiology 2003; 99: 259–69.

**Table 84 (abstract A1104).** Initial management

	PAC SURG n = 32	PAC ANGIO n = 67	Student t tests CHI2
Shockable Initial rhythms	9 ( 30 % )	42 ( 62,6 % )	p = 0,0027
Non schockable initial rhythms	21 ( 70 % )	25 ( 37.4 % )	p = 0,0153
MV before PAC	23 ( 71.9 % )	8 ( 11.9 % )	p <0,00001
Delay before intervention	1,02 +/− 0,09	1,03 +/− 0,17	p = 0,7557
CEE	11 ( 36.7 % )	48 ( 71.6 % )	p = 0,0009
Adrenaline use	28 ( 87,5 % )	39 ( 58.2 % )	p = 0,007
CPR duration	22 +/− 23,7	15,8 +/− 15,4	p = 0,121
ROSC	11,6 +/− 12,4	4,8 +/− 7,5	p = 0,001

*MV* Mechanical ventilation, *PAC SURG* Peroperative cardiac arrests occuring in surgical theatres, *PAC ANGIO* Peroperative cardiac arrests occurring in angiographic and cardiac catheterism patients

**Table 85 (abstract A1104).** Survivors follow up

	PAC SURG	PAC ANGIO	Student t tests CHI2
Sample size	32	67	
ISR	24 (75 %)	46 (68.7 %)	p = 0,515
SAPS 2 score	52 +/− 10,6	47,2 +/− 10,9	p = 0,041
ICU stay	7,4 +/− 12	4,5 +/− 5,4	p = 0,01
Hypothermia	5 (20,8 %)	3 (6.7 %)	p = 0,131
MV duration	4,7 +/− 8,7	2,1 +/− 5,7	p = 0,079
Hospital stay	12,2 +/− 11,7	8 +/− 5,5	p = 0,017
HSR	19 (59.4 %)	37 (55.2 %)	p = 0,697

*ISR* Immediat survival rate, *HSR* Hospital survival rate, *ICU* Intensive care unit, *SAPS* Simplified Acute Physiology, *MV* Mechanical ventilation, *PAC SURG* Peroperative cardiac arrests occuring in surgical theatres, *PAC ANGIO* Peroperative cardiac arrests occurring in angiographic and cardiac catheterism patients

**Fig. 119 (abstract A1104).**
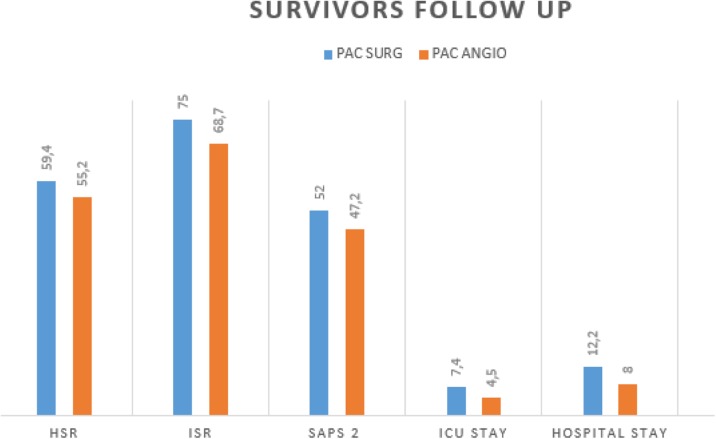
Survivors follow up

**Fig. 120 (abstract A1104).**
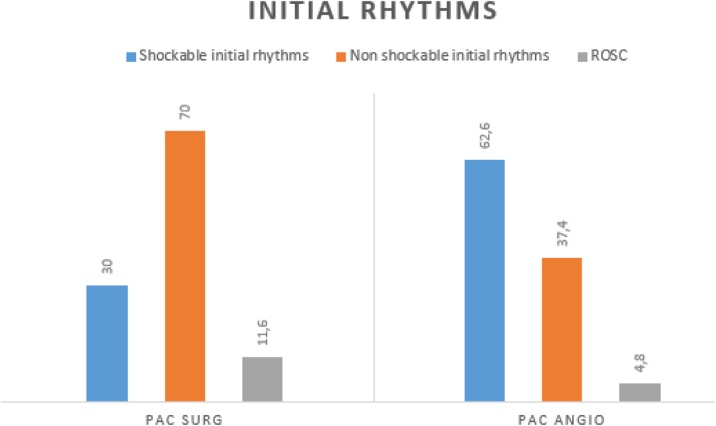
Nitial rhythms

### A1105 ECLS in refractory cardiac arrest : a retrospective study of factors predicting survival and neurological outcome

#### E. Fournel^1^, L. Gaide-Chevronnay^1^, M. Durand^1^, P. Albaladejo^1^, J.-F. Payen^1^, O. Chavanon^2^

##### ^1^CHU Grenoble, Anesthesiology and Intensive Care, Grenoble, France; ^2^CHU Grenoble, Cardiac Surgery, Grenoble, France

###### **Correspondence:** O. Chavanon – CHU Grenoble, Cardiac Surgery, Grenoble, France

**Introduction:** The use of extra-corporeal life support (ECLS) in refractory cardiac arrest (RCA) has spread in the last twenty years. However, morbidity and mortality associated with this practice remains high. Previous studies identified several risk factors, leading to recommendations, for example french AFAR criteria (1), aiming to target RCA patients eligible for ECLS. Moreover, other studies suggested a higher survival rate among in-hospital than out-of-hospital victims of RCA (2).

**Objectives:** We studied factors linked to survival of patients who underwent ECLS during RCA or shortly after recuperation.

**Methods:** This is a monocentric study from January 2006 to December 2014, conducted in universitary hospital in Grenoble, France. 196 patients admitted for ECLS after RCA or shortly after RCA recuperation were retrospectively included. All factors which could impact survival were studied e.g. cardiovascular risk factors and co-morbidity, delay before medical intervention, ECLS complications, cause of death, in- versus out-of-hospital RCA, initial pulse, treatment following the AFAR criteria.Our primary outcome was 30 days survival. Secondary outcomes were neurological status at 3 months (using CPC score) and ECLS-induced complications.

**Results:** Overall survival rate was 15.8 % (n = 31). Mean age (±sd) was 49.4 (±15.6) years old, with no difference between survivors and non-survivors (respectively 46.2 ± 17 and 50 ± 15.4, p = 0.22). Low-flow duration was significantly lower for survivors than non-survivors (42,7 ± 34.1 min versus 70 ± 41.7 min, p = 0.002), as well as time before medical intervention (1.5 ± 4.1 min vs 6 ± 9.9 min, p = 0.04). Initial shockable rhythm was associated to survival (p = 0.03). 62,6 % of out-of-hospital RCA cases filled AFAR criteria with a trend for better survival (14.5 % vs 8.1 %, p = 0.063). 23 patients had a neurological status evaluation after recovery, 5 were lost to follow-up. Among them, 20 (74,2 %) had none or minor sequelae (CPC score 1 or 2), and the remaining 3 suffering heavy neurological damage (CPC score 3).

**Conclusions:** Mortality of patients undergoing ECLS after refractory cardiac arrest remains high, but survivors have good neurological recovery, suggesting criteria to start the procedure must be more selective.

**References**

(1) Guidelines for indications for the use of extracorporeal life support in refractory cardiac arrest. French Ministry of Health. Ann Fr Anesth Reanim. 2009 Feb;28(2):182–90

(2) Kagawa et al. Assessment of outcomes and differences between in- and out-of-hospital cardiac arrest patients treated with cardiopulmonary resuscitation using extracorporeal life support. Resuscitation. 2010 Aug;81(8):968–73.

**Grant acknolwedgment**

None.

### A1106 Extracorporeal cardiopulmonary resuscitation: a single-center study

#### A. Blandino Ortiz, S. Pozzebon, O. Lheureux, A. Brasseur, J.-L. Vincent, J. Creteur, F.S. Taccone

##### Erasme University Hospital, Université Libre de Bruxelles, Department of Intensive Care, Brussels, Belgium

###### **Correspondence:** A. Blandino Ortiz – Erasme University Hospital, Université Libre de Bruxelles, Department of Intensive Care, Brussels, Belgium

**Introduction:** Veno-arterial Extracorporeal membrane oxygenation (VA-ECMO) is commonly used to treat severe cardio-pulmonary failure in critically ill patients. Nevertheless, the use of VA-ECMO for refractory cardiac arrest (ECPR) has shown promising results for in-hospital cardiac arrest (IHCA) while its use for out-of-hospital cardiac arrest (OHCA) remains controversial. Moreover, the intensity of therapy and morbidity associated with such interventions are poorly described.

**Methods:** We reviewed our institutional VA-ECMO database (n = 159) from November 2008 to December 2014 and identified those patients who were treated with ECPR for IHCA or OHCA. We recorded demographics data as well as organ dysfunction and concomitant therapies, complications and neurological outcome at 3 months after arrest.

**Results:** A total of 70 patients (age 53 [41–63] years) were treated over the study period; 46 patients experienced OHCA and in 57 (81 %) patients the cause of arrest was cardiac. 58 patients received a bystander cardiopulmonary resuscitation and median time to basic life support (BLS) was 2 [1–7] minutes. Ten (14 %) of these patients could not be percutaneously cannulated and immediately died. Total time from arrest to ECMO onset was 59 [45–70] minutes (n = 60). Median ECMO blood flow on admission was 4.0 [3.5-4.2] L/min, FiO_2_ of 1.0 [1.0-1.0] and gas flow of 6 [5–7] L/min. Initial lactate levels were 14.3 [8.1-18.3] mmol/L and most of patients were on vasopressors therapy. Coronary angiography was performed in 28/57 (49 %) patients with a cardiac cause; continuous renal replacement therapy was initiated in 27 out of the 52 patients (52 %) developing acute kidney injury during the ICU stay. 14 patients showed a full neurological recovery during the ICU stay (23 %) but only 12 were still alive with intact neurological function at 3 months (20 %); 6/57 after OHCA (11 %) and 6/23 (26 %) after IHCA. Eight patients (13 %) with irreversible brain damage had organ function suitable for donation and 4 were eventually explanted.

**Conclusions:** ECPR provided acceptable survival rate with good neurologic recovery in refractory cardiac arrest. These patients underwent several additional therapeutic interventions, which, in case of irreversible brain damage, could stabilize extra-cerebral organ function and potentially provide some available organs for donation.

### A1107 Post-resuscitation treatment with inhaled argon improves outcome even after a prolonged untreated cardiac arrest in a porcine model

#### F. Fumagalli^1^, S. Scala^1^, R. Affatato^1^, M. De Maglie^2^, D. Zani^2^, D. Novelli^1^, C. Marra^1^, A. Luciani^1^, D. De Zani^3^, M. Luini^3^, T. Letizia^4^, D. Pravettoni^2^, L. Staszewsky^1^, S. Masson^1^, A. Belloli^2^, M. Di Giancamillo^2^, E. Scanziani^2^, R. Latini^1^, G. Ristagno^1^

##### ^1^IRCCS - Istituto di Ricerche Farmacologiche “Mario Negri”, Milan, Italy; ^2^University of Milan, Milan, Italy; ^3^Istituto Zooprofilattico Sperimentale della Lombardia e dell'Emilia, Lodi, Italy, ^4^Sacco Hospital, Milan, Italy

###### **Correspondence:** F. Fumagalli – IRCCS - Istituto di Ricerche Farmacologiche “Mario Negri”, Milan, Italy

**Introduction:** After the initial success of cardiopulmonary resuscitation (CPR), the majority of patients die, mainly due to post-resuscitation (PR) cardiac failure and ischemic brain damage. Inhaled argon has shown neuroprotective effects in a porcine model of cardiac arrest (CA) of short duration.

**Objectives:** To investigate the effect of post-resuscitation treatment with inhaled argon on outcome in a preclinical porcine model of prolonged untreated CA and CPR. We hypothesized that argon would ameliorate post-resuscitation neurologic dysfunction.

**Methods:** The left anterior descending coronary artery was occluded in 24 pigs (39 ± 2 kg), and ventricular fibrillation (VF) was induced. After 12 min of untreated VF, CPR, including mechanical chest compression, ventilation and adrenaline administration, was performed for 5 min prior to defibrillation. Following successful resuscitation, animals were subjected to 4 hr ventilation with (a) 70 % argon - 30 % O_2_ (n = 10) or (b) 70 % N_2_ - 30 % O_2_ (n = 10). Hemodynamics were continuously monitored and systolic myocardial function (i.e. ejection fraction (EF), shortening fraction (SF)) was assessed by echocardiography. Serial blood samples were obtained for blood gas, serum neuron specific enolase (NSE) and plasma high sensitive cardiac troponin T (hs-cTnT) assays. Animals were observed up to 96 hr for assessment of survival and neurological recovery (Cerebral Performance Categories (CPC) scale).

**Fig. 121 (abstract A1107).**
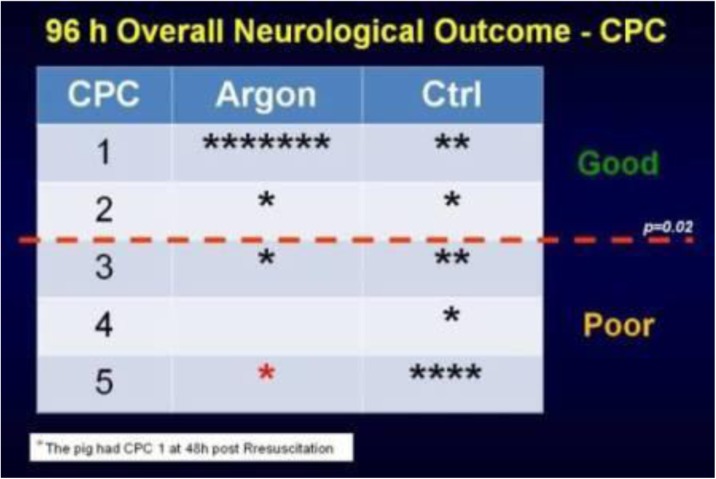
96 h Overall Neurological Outcome- CPC

**Results:** Twenty animals were successfully resuscitated and enrolled in the study (Table 87). Ventilation with argon did not have any detrimental effects on respiratory gas exchange during the 4 hr ventilation (Table 87). Animals receiving argon showed a significantly lower heart rate and higher mean arterial pressure and stroke volume compared to controls during the 4 hr of observation (Table 87). Animals treated with argon presented also a significantly better recovery of systolic myocardial function, as represented by the higher SF at 96 hr compared to controls (Table 87). Nine of the 10 resuscitated animals in the argon group survived for 96 h in comparison to 6 out of 10 in the control group. Animals treated with argon presented a significantly better neurological recovery (CPC 1.7 ± 1.3) in contrast to animals in the control group (3.4 ± 1.6, Figure 121). Lower circulating levels of hs-cTnT (median: 1332 ng/mL vs. 8015 ng/mL, p < 0.05) and NSE (median 8.5 ng/mL vs. 21.2, p not significant) were observed in the animals ventilated with argon compared to controls.

**Conclusions:** In this severe model of CA, post-resuscitation treatment with argon allowed for improved hemodynamics, myocardial function and neurologic recovery, without detrimental effects on respiratory gas exchanges.

**References**

1. Ristagno G. et al., Shock 2014 Jan;41(1):72–8; Brucken A et al., Neurocrit Care 2015 Feb;22(1):112–20

**Grant acknolwedgment**

Italian Ministry of Health, Italy (Convenzione n.46/GR-2011-02348099); and Fondazione Sestini, Bergamo, Italy.

**Table 86 (abstract A1107).** Hemodynamics & myocardial function

	Argon	Control
Resuscitation, n/n	10/12	10/12
PH: Baseline, PR 2 hr, PR 4 hr	7.49 ± 0.07, 7.40 ± 0.07, 7.44 ± 0.05	7.49 ± 0.09, 7.37 ± 0.07, 7.42 ± 0.07
PCO2 (mmHg): Baseline, PR 2 hr, PR 4 hr	37 ± 3, 40 ± 2, 40 ± 2	38 ± 2, 40 ± 2, 40 ± 2
PO2 (mmHg): Baseline, PR 2 hr, PR 4 hr	85 ± 11, 112 ± 21, 114 ± 26	79 ± 14, 110 ± 16, 103 ± 22
Heart rate (b/min): Baseline, PR 2 hr, PR 4 hr	88 ± 23, 131 ± 16, 120 ± 27	104 ± 33, 173 ± 20**, 165 ± 39*
Mean arterial pressure (mmHg): Baseline, PR 2 hr, PR 4 hr	102 ± 15, 107 ± 14, 102 ± 12	98 ± 10, 90 ± 16*, 86 ± 16*
Ejection fraction (%): Baseline, PR 2 hr, PR 4 hr, PR 96 hr	68 ± 6, 30 ± 7, 38 ± 15, 62 ± 1	75 ± 8, 36 ± 6, 36 ± 12, 54 ± 23
Stroke volume (ml): Baseline, PR 2 hr, PR 4 hr	49 ± 11, 23 ± 4, 23 ± 7	47 ± 14, 19 ± 4*, 19 ± 5
Shortening fraction (%): Baseline, PR 2 hr, PR 4 hr, PR 96 hr	46 ± 8, 24 ± 6, 26 ± 16, 41 ± 8	47 ± 11, 17 ± 7, 18 ± 6, 30 ± 10*

*p < 0.05

**p < 0.01

### A1108 Effect of therapeutic hypothermia on mitogen activated protein kinase pathway in the brain tissue of a swine cardiac arrest model

#### Y.C. Kye^1^, G.J. Suh^2^, W.Y. Kwon^2^, K.S. Kim^2^, K.M. Yu^2^

##### ^1^VHS Medical Center, Emergency Medicine, Seoul, Republic of Korea; ^2^Seoul National University Hospital, Emergency Medicine, Seoul, Republic of Korea

###### **Correspondence:** Y.C. Kye – VHS Medical Center, Emergency Medicine, Seoul, Republic of Korea

**Objective:** To investigate the change in mitogen-activated protein kinase pathways in the brain tissue after therapeutic hypothermia in swine cardiac arrest model.

**Design.** Prospective animal study

**Setting.** University animal laboratory

**Subjects.** Male domestic pigs (n = 24)

Interventions: After the return of spontaneous circulation by cardiopulmonary resuscitation following 6 min of no flow time induced by ventricular fibrillation, pigs were randomly assigned to one of four groups (sham, normothermia, 24 hr of therapeutic hypothermia, 48 hr of therapeutic hypothermia). Therapeutic hypothermia (core temperature 32-34 °C) was maintained and the pigs were then rewarmed for 8 hr. At 60 hr after the return of spontaneous circulation, the pigs were sacrificed and brain tissues were harvested.

**Measurement and main results:** We measured the tissue levels of p38, JNK, and ERK pathway expressions in swine brain hippocampus of the four groups. The phosphorylated p38 to p38 ratio and phosphorylated JNK to JNK ratios were significantly increased in all of the intervention groups, relative to the sham group. but the phosphorylated ERK to ERK ratio was increased only in the therapeutic hypothermia groups (*p*-value = 0.026 in the 24 hr of therapeutic hypothermia group and *p*-value = 0.002 in the 48 hr of therapeutic hypothermia group, both compared to the sham group).

**Conclusions:** Normothermia activated the p38 and JNK pathway. And did not activate the ERK pathway in ischemia-reperfusion injury after cardiac arrest. Therapeutic hypothermia, however, did not attenuate the activation of the p38 and JNK pathways, but activated the ERK pathway, which seemed to be dose dependent with the duration of therapeutic hypothermia.

### A1109 Effect of permissive hypercapnia on outcome of cardiac arrest in a porcine model of cardiopulmonary resuscitation

#### G. Babini^1^, G. Ristagno^1^, L. Grassi^1^, F. Fumagalli^1^, S. Bendel^2^, M. De Maglie^3^, R. Affatato^1^, S. Masson^1^, R. Latini^1^, E. Scanziani^3^, M. Reinikainen^4^, M. Skrifvars^5^

##### ^1^IRCCS - Istituto di Ricerche Farmacologiche “Mario Negri”, Milan, Italy; ^2^Kuopio University Hospital, Kuopio, Finland; ^3^University of Milan, Milan, Italy; ^4^North Karelia Central Hospital, Karelia, Finland; ^5^Helsinki University Hospital, Helsinki, Finland

###### **Correspondence:** L. Grassi – IRCCS - Istituto di Ricerche Farmacologiche “Mario Negri”, Milan, Italy

**Introduction:** Despite advances in post-resuscitation care, cardiac arrest mortality remains high, mainly because of severe neurological injury. Recently, clinical studies have reported a potential benefit of permissive hypercapnia following cardiac arrest on survival and neurological outcome.

**Objectives:** To evaluate effects of a hypercapnic ventilatory strategy on outcome of cardiac arrest in a porcine model.

**Methods:** An established model of cardiac arrest in the pig was used. Fourteen animals were endotracheally intubated and mechanically ventilated. Arterial and central venous lines were established for hemodynamic measures. End-tidal CO_2_ (EtCO_2_) was continuously monitored. Ventricular fibrillation was induced and untreated for 12 minutes. Cardiopulmonay resuscitation, with chest compressions, mechanical ventilation, and adrenaline, was then performed for 5 min prior to defibrillation. After resuscitation, the pigs were assigned to either normocapnic control ventilation (EtCO_2_ 35–40 mmHg) or hypercapnic ventilation (EtCO_2_ 45–50 mmHg) for 4 hrs. Samples for blood gas analyses and biomarkers of cerebral (serum neuron-specific enolase, NSE) and cardiac injury (high sensitive troponin T, hs-cTnT) were taken. Survival and neurological recovery was evaluated up to 96 hr after resuscitation. Animals were then sacrificed and hearts and brains harvested.

**Fig. 122 (abstract A1109).**
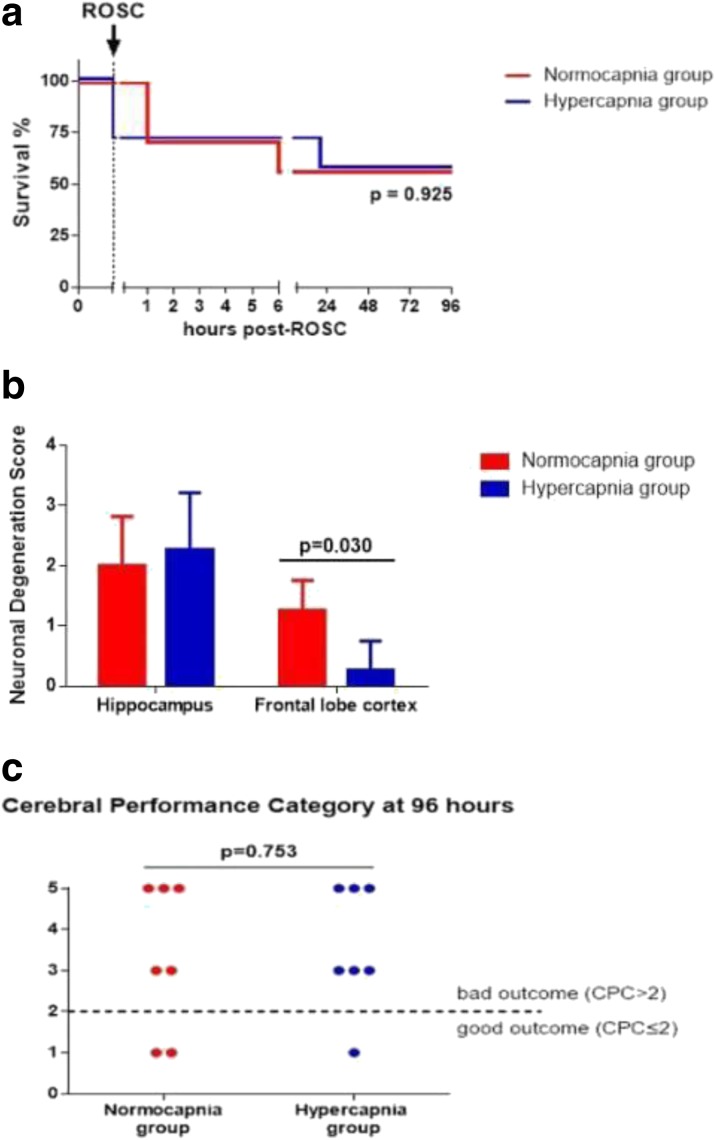
ᅟ

**Results:** Twelve pigs were successfully resuscitated and eight pigs survived until 96 hrs (Table 88, Figure 122). Pigs in the hypercapnic group showed a trend towards longer survival. ETCO_2_ and PCO_2_ were significanlty higher in the hypercaninc group compared to the normocpanic one (Table 88). pH and PO_2_ trended to be lower in the hypercapnic group during the 4 hrs of observation. Hypercapnia was associated with significantly higher mean arterial pressure during the post-resuscitation (PR) period (Table 88). No differences were observed in hs-cTnT and in NSE between groups, although the values were lower at 96 hrs in the normocapnic group (hs-cTnT: 295 ng/ml vs. 89 ng/ml and NSE: 30 ng/ml vs. 23 ng/ml, p not significant - table). The infarct area of the left ventricle was not different between groups. Lesser neuronal degeneration was seen in the frontal cortex in the hypercapnic group compared to the normocapnic one (Figure 122). Neurological recovery was equivalent in the two groups (Figure 122).

**Conclusions:** Permissive hypercapnia after resuscitation was associated with better mean arterial pressure and lesser neuronal degeneration in pigs.

**Grant support.** Laerdal Foundation for Acute Care, Norway

**Table 87 (abstract A1109).** ᅟ

	NORMOCAPNIA GROUP (n = 7)	HYPERCAPNIA GROUP (n = 7)
Survival duration, hr	56 ± 50	81 ± 34
96 hr survival, n/n (%)	4/7 (57)	4/5 (80)
ETCO2, Basal PR2hr PR4hr	37 ± 1 36 ± 1 36 ± 2	38 ± 2 48 ± 1 ** 48 ± 2 **
pH, Basal PR2hr PR4hr	7.48 ± 0.08 7.34 ± 0.07 7.40 ± 0.08	7.46 ± 0.06 7.23 ± 0.10 7.27 ± 0.11
PaO2 (mmHg), Basal PR2hr PR4hr	76 ± 16 111 ± 19 103 ± 28	81 ± 9 85 ± 24 95 ± 21
PaCO2 (mmHg), Basal PR2hr PR4hr	38 ± 2 40 ± 2 41 ± 1	38 ± 3 54 ± 6 ** 54 ± 7 **
Heart Rate (bpm), Basal PR2hr PR4hr	103 ± 28 171 ± 23 153 ± 43	113 ± 44 162 ± 52 177 ± 30
Mean Arterial Pressure (mmHg), Basal PR2hr PR4hr	97 ± 11 83 ± 13 76 ± 8	97 ± 17 102 ± 18 102 ± 18 *
Right Atrial Pressure (mmHg), Basal PR2hr PR4hr	6 ± 2 8 ± 2 8 ± 2	5 ± 2 6 ± 2 7 ± 4

*p < 0.05

**p < 0.01

### A1110 Oligoanalgesia in trauma patients at a physician staffed emergency service in Munich

#### F. Kappler, M. Blobner, S.J. Schaller

##### Technische Universität München, Klinik für Anaesthesiologie, Munich, Germany

###### **Correspondence:** S.J. Schaller – Technische Universität München, Klinik für Anaesthesiologie, Munich, Germany

**Introduction:** Pain is the main indication for utilisation of the physician staffed prehospital emergency service in Germany.^1^ Data from Switzerland^2^ showed that oligoanalgesia (inappropriate treatment of pain with NRS > 3) is common in trauma patients.

**Objectives:**

1. Determination of the frequency of oligoanalgesia in trauma patients at our prehospital emergency service location in Munich, Germany, which is staffed with physicians working at a university hospital in the specialities anaesthesia or surgery.
2. Test if there is a difference between specialists and residents in pain treatment of trauma patients.

**Methods:** After Ethics Committee approval, retrospective analysis of the protocols of our prehospital emergency service location in Munich, Germany of 2014–2015. Statistical calculation was done using logistic regressions with STATA14 (College Station, TX, USA).

**Fig. 123 (abstract A1110).**
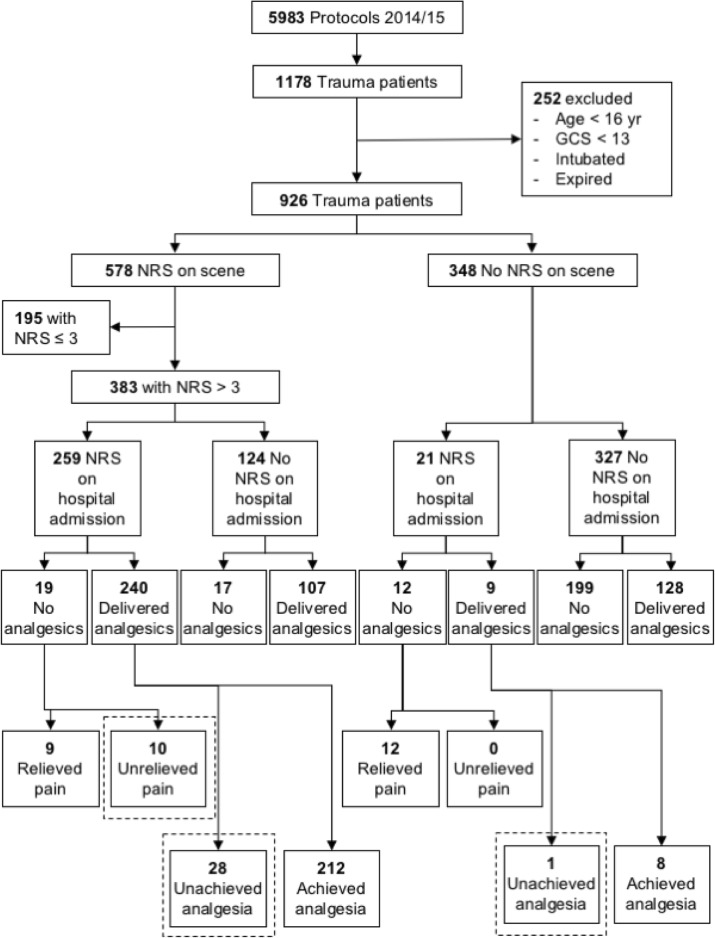
Flow chart/Consort Diagramm

**Results:** 1178 documented trauma cases. 242 trauma cases could be assessed for frequency of oligoanalgesia, which was present in 39 of these cases (see Figure 123, dashed frames), leading to an relative frequency of 16 % of cases. There was no difference in frequency between residents and specialists (Table 89).

Relatively more trauma cases where handed by specialists, while documentation of pain was better in residents (Table 89). Documentation of pain, however, was insufficient, since pain assessment at hospital admission was documented in 38 % of possible cases of oligoanalgesia only.

**Conclusions:** Frequency of oligoanalgesia in trauma patients seems to vary in different systems, since it was much lower in Munich compared to Switzerland (16 % vs. 43 %, respectively). There are several possible explanations: Data from Swizerland was from an air resuce service while our data is from a ground based system. Second, in our system possibility of treatment by a specialist was much higher (83 % residents in Switzerland). Third, documentation in our system was inadequate. Theoretically, frequency of oligoanalgesia could increase up to 35 % if all cases without adequate pain documentation were counted as oligoanalgetic. To assess appropriate numbers improvement in documentation is essential.

**References**

1. Hossfeld, B. et al. Prähospitale Analgesie beim Erwachsenen. Notf.med. up2date 10, 269–284 (2015)

2. Albrecht, E. et al. Undertreatment of acute pain (oligoanalgesia) and medical practice variation in prehospital analgesia of adult trauma patients: a 10 yr retrospective study. Br. J. Anaesth.,110 (1): 96–106 (2013)

**Table 88 (abstract A1110).** Differences residents - specialists

	Resident	Specialist	Total	Difference	P-Value
Trauma cases	371	807	1178	1.2 (1.1–1.4)	0.006
Documentation on the scene	233	415	648	0.5 (0.3–0.7)	<0.001
… at hospital admission	160	274	434	0.6 (0.4–0.8)	<0.001
… of both	144	261	405	0.6 (0.5–0.9)	0.007
Opioids administered	110	244	354	1.0 (0.7–1.4)	n.s.
Ketamin administered	60	96	156	0.6 (0.4–1.0)	0.028
No analgesic administered	186	420	606	1.2 (0.9–1.7)	n.s.
Effectiveness of therapy	85	135	220	1.3 (0.6–2.8)	n.s.
Oligoanalgesia	17	22	39	0.8 (0.4–1.6)	n.s.

### A1111 The impact of rapid response team on epidemiology of in-hospital cardiac arrest: a 5-years observational study

#### A. Roasio, E. Costanzo, S. Cardellino

##### Cardinal Massaia Hospital, Anesthesia and Intensive Care Unit, Asti, Italy

###### **Correspondence:** A. Roasio – Cardinal Massaia Hospital, Anesthesia and Intensive Care Unit, Asti, Italy

**Introduction:** Data about in-hospital cardiac arrest (IHCA) were reported in different studies but none concerns Italian situation^1–2^. Furthermore the efficacy of Rapid Response Team (RRT) on incidence and outcome of IHCA is debated^3^.

**Objectives:** to assess the epidemiology of IHCA in an Italian center and to evaluate the impact of RRT on its incidence and outcome.

**Methods:** This is a prospective, single center, observational study developed in a 500-bed hospital in Northern Italy from July 1^st^ 2010 to June 30^th^ 2015. Inclusion criteria: adult patients, IHCA treated with cardiopulmonary resuscitation (CPR). Exclusion criteria: do not resuscitate order. Protocol: data were collected anonymously according to the Utstein style. Follow-up: 6 months long using registry office and telephonic interview. Data: age, sex, cerebral performance category (CPC 1 good 2 moderate disability, 3 severe disability, 4 unconscious), site of cardiac arrest, presumed etiology, initial rhythm (shockable or unshockable), witnessed event, monitored, CPR started within 1 minute. Primary end points: return of spontaneous circulation (ROSC), survival to hospital discharge and CPC 1–2. Secondary end points: 6 months survival and CPC 1–2. Statistics: numerical data are expressed as mean ± standard deviation or median (interquartile range), as percentage if ordinal data. Chi-square test for ordinal data and T Student's test for numerical data were performed. P significant if < 0.05.

**Results:** 440 cardiac arrests, CPR was carried out in 389 cases (88 %). Incidence rate: 5.1 events/1000 recoveries per year, reduced from 7.2 to 4.1 (P = 0.02). Age: 76 ± 13 years. Male: 60 %. CPC 1–2 before cardiac arrest in 85.6 % of cases. Site: 21.5 % monitored ward, 57 % unmonitored ward, 18.5 % emergency department and 3 % other location. Presumed etiology: cardiac 53 %, respiratory 31 %, others 16 %. Shockable rhythm 15 %, unshockable 84 %, unknown rhythm 1 %. 90.4 % witnessed and 46.7 % monitored. 85.8 % of CPR started within 1 minute. Outcome data: 37.5 % ROSC, 15.1 % discharged alive from hospital. CPC 1–2 in 91.5 % of patients. Secondary end points: 12.3 % alive at 6 months; 95.5 % of them with CPC 1–2.

**Conclusions** Our experience reflects some aspects common with other European countries: less monitored events as well as more frequent cardiac arrests in unmonitored wards^1^. RRT allowed a reduction of cardiac arrests thus reducing their incidence without modifying mortality.

**References**

1) Nolan JP et al. Incidence and outcome of in-hospital cardiac arrest in the United Kindom National Cardiac Arrest Audit. Resuscitation 2014;85:987–992.

2) Kazaure H, et al. Epidemiology and outcomes of in-hospital cardiopulmonary resuscitation in the United States, 2000–2009. Resuscitation 2013;84:1255–1260.

3) Solomon RS et al. Effectiveness of rapid response teams on rates of in-hospital cardiopulmonary arrest and mortality: a systematic review and meta-analysis. J Hosp Med 2016; doi:10.1002/jhm.2554.

**Grant acknolwedgment**

None.

### A1112 Acute liver failure after cardiac arrest

#### E. Iesu, F. Zama Cavicchi, V. Fontana, L. Nobile, J.L. Vincent, J. Creteur, F.S. Taccone

##### ULB Université Libre de Bruxelles, Department of Intensive Care, Erasme University Hospital, Bruxelles, Belgium

###### **Correspondence:** E. Iesu –ULB Université Libre de Bruxelles, Department of Intensive Care, Erasme University Hospital, Bruxelles, Belgium

**Introduction:** Hypoxic liver injury typically occurs in individuals with right-sided congestive heart failure and/or low cardiac output. Despite patients surviving after cardiac arrest (CA) develop several extra-cerebral organ dysfunction, acute liver failure has been rarely described in this setting.

**AIM.** To describe the occurrence of ALF in patients after CA.

**Methods:** Analysis of an adult CA patients database, admitted to our Department of Intensive Care from January 2012 through December 2015. We excluded patients who died within the first 24 hours (n = 38). We retrieved all data concerning CA characteristics as well as liver function (in particular total bilirubin [Bil], alanine [ALT] and aspartate [AST] aminotransferase and international normalized ratio, INR). Acute Liver Failure was defined as a bilirubin > 1.2 mg/dL and INR > 1.5 during the first 3 days since admission. Neurological outcome was evaluated 3 months after CA (assessed during follow-up visits or by telephone interview with the general practitioner). Favorable neurological outcome (FO) was defined as a Cerebral Performance Categories (CPC) score of 1–2; poor neurological outcome (PO) as a CPC scores of 3–5.

**Results:** We included a total of 145 patients (age 63 [52–72] years; male gender 109/145). 78 (54 %) patients had an out-of-hospital CA and 58 (40 %) had a shockable initial rhythm. ALF was observed in 38 patients (26 %). Patients with ALF were younger (57 [50–66] vs. 65 [53–73] years; p = 0.01) and had higher baseline lactate (7.2 [4.0-11.0] vs. 5.2 [2.5-8.6] mmol/L; p = 0.02) than others. No other differences were found for the rate of bystander cardiopulmonary resuscitation, use of vasopressors and/or inotropic agents between the two groups. As expected, patients with ALF had higher ALT and AST on admission then others. Poor neurological outcome was observed in 27/38 (71 %) patients with ALF and 60/107 (56 %) in those without ALF (p = 0.005).

**Conclusions:** ALF is not a rare complication after CA; patients with ALF have a higher rate of poor neurological outcome in this setting.

### A1113 Is the pulmonary artery (PA) temperature really gold standard for targeted temperature management (TTM)?

#### M. Park^1^, K.M. You^1^, G.J. Suh^1^, W.Y. Kwon^1^, S.B. Ko^2^, K.S. Kim^1^

##### ^1^Seoul National University Hospital, Emergency Medicine, Seoul, Republic of Korea; ^2^Seoul National University, Neurology, Seoul, Republic of Korea

###### **Correspondence:** M. Park – Seoul National University Hospital, Emergency Medicine, Seoul, Republic of Korea

**Introduction:** During targeted temperature management (TTM), pulmonary artery(PA) temprerature was recommended for estimating brain temperature. Howevere, there have been few data to show if pulmonary artery temperature well estimates brain parenchymal temperature.

**Objectives:** We performed this study to investigate whether pulmonary artery temperature well estimates brain parenchymal temperature during TTM in swine cardiac arrest model.

**Methods:** This study was conducted on 4 male domestic pigs (30 ± 5 kg). After 8 minutes of no-flow time that was induced by ventricular fibrillation, cardiopulmonary resuscitation was provided, and the return of spontaneous circulation was achieved. TTM (core temperature, 32 - 34 °C) was maintained for 8 hours post-return of spontaneous circulation, and the animals were rewarmed for 12 hours. During TTM, brain parenchymal and pulmonary artery temperatures were measured by Bowman perfusion monitor®(HemedexTM, Cambridge, MA) and Swan-Ganz catheter(Edwards Lifesci. Corp., Irvine, CA), respectively. Rectal temperature was also measured. Data were compared using repeated measures ANOVA test with Dunnett *post-hoc* test.

**Results:** During TTM, brain parenchymal temperature was higher than pulmonary artery temperature (p = 0.026). The mean difference between the brain parenchymal and pulmonary artery temperature was 0.44 ± 0.10 °C. Rectal temperature was not different from brain parenchymal and pulmonary artery temperatures (p = 0.064 and 0.914, respectively)

**Conclusions:** Pulmonary artery temperature did not well estimate brain parenchymal temperature during TTM.

**Fig. 124 (abstract A1113).**
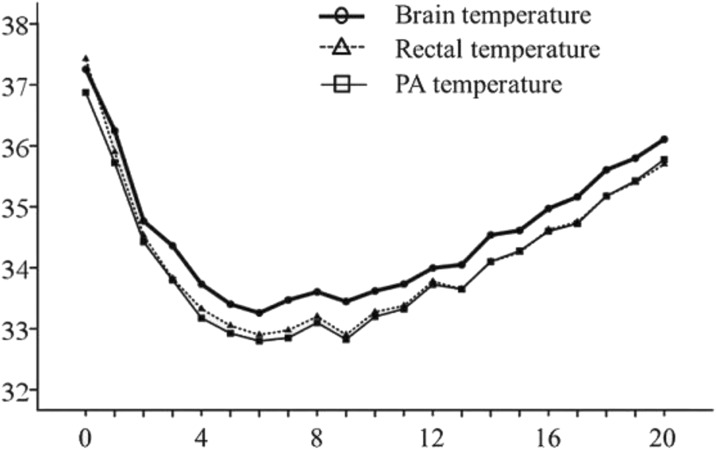
ᅟ

### A1114 A case series of veno-arterial-venous (VAV) ECMO

#### A. Xini, L. Marca, O. Lheureux, A. Brasseur, J.L. Vincent, J. Creteur, F.S. Taccone

##### Erasme Hospital, Brussels, Belgium

###### **Correspondence:** A. Xini – Erasme Hospital, Brussels, Belgium

**Introduction:** Extracorporeal membrane oxygenation (ECMO) is increasingly used to treat cardio-pulmonary failure in critically ill patients. Peripheral cannulation may be complicated by a persistent low cardiac output in case of veno-venous cannulation (VV-ECMO) or by differential hypoxia (e.g. lower PaO2 in the upper than in the lower body) in case of veno-arterial cannulation (VA-ECMO) and severe impairment of pulmonary function associated with cardiac recovery. The treatment of such complications remains challenging. We reported our experience with the use of veno-arterial-venous (VAV) ECMO in this setting.

**Methods and results:** We reviewed our institutional ECMO database (n = 285) from November 2008 to December 2015 andidentified those patients that needed a conversion from the initial VV- or VA-ECMO to a VAV technique. We collected demographic data as well as comorbidities and ECMO characteristics, hemodynamics and arterial blood gas values before and after the VAV implementation. We treated 9 patients (age 53 [ranges: 31–66] years; 7 male) with VAV, most being placed on ECMO (n = 8 VA-ECMO; n = 1 VV-ECMO) on the day of ICU admission. The indications for VA-ECMO were cardiogenic shock (n = 4), refractory cardiac arrest (n = 3)and septic shock (n = 1); the indication for VV-ECMO was septic shock (n = 1). Overall ICU mortality was 6/9 (66 %). The median time from ECMO implantation to VAV conversion was 0 [0–2] days. Before VAV implantation, PaO2 was 65 [40–100] mmHg, cardiac output (CO) was 5.1 [3.5-7.9] L/min and lactate 4.7 [3.5-20] mmol/L; dose of norepinephrine was 66 [15–1470] mcg/min. ECMO blood flow was 3.8 [3.5-4.5] L/min, gas flow at 6 [3–7] L/min and FiO2 100 [100–100]%. After VAV implantation, we observed a trend in the reduction of norepinephrine dose (32 [5–630] mcg/min; p = 0.1) and a significant improvement in PaO2 (85 [54–176] mmHg; p = 0.05). ECMO blood flow was increased to 5.5 [4.4-6.5] L/min, with 2.7 [1.3-3.1] L/min for the arterial and 3.6 [2.4-3.9]L/min for the venous reinjection.

**Conclusions:** VAV ECMO should be considered to treat differential hypoxia and persistently low cardiac output syndrome during ECMO. It could be a valid alternative to central ECMO in these patients.

### A1115 Practices and perspectives in cardiopulmonary resuscitation attempts: a cross-sectional survey in a low middle income country

#### A. Beane^1,2,3^, M.C.K.T. Thilakasiri^1,2,4^, A.P. De Silva^1,2,5^, T. Stephens^1^, C.S. Sigera^1,2^, P. Athapattu^2,6^, S. Jayasinghe^2,7^, A. Padeniya^8,9^, R. Haniffa^2,7,10^

##### ^1^Network for Improving Critical Care Systems and Training (NICST), Colombo, Sri Lanka; ^2^National Intensive Care Surveillance, Quality Secretariat Building, Castle Street Hospital for Women, Colombo, Sri Lanka; ^3^Royal London Hospital, Barts Health, Adult Critical Care Unit, London, United Kingdom; ^4^Colombo North Teaching Hospital Sri Lanka, Ragama, Sri Lanka; ^5^Intensive Care National Audit & Research Centre, London, United Kingdom; ^6^Office of Medical Services, Ministry of Health, Colombo, Sri Lanka; ^7^Department of Clinical Medicine, Faculty of Medicine, University of Colombo Sri Lanka, Colombo, Sri Lanka; ^8^Government Medical Officers' Association, Colombo, Sri Lanka; ^9^Lady Ridgeway Hospital for Children, Colombo, Sri Lanka; ^10^Mahidol Oxford Tropical Medicine Research Unit, Faculty of Tropical Medicine, Mahidol University, Bangkok, Thailand

###### **Correspondence:** A. Beane – Network for Improving Critical Care Systems and Training (NICST), Colombo, Sri Lanka

**Introduction:** Inclusion of Advanced Life Support (ALS) algorithms during cardiopulmonary resuscitation (CPR) is considered a bench-mark of a country´s health system. In Europe, proactive decisions are increasingly made as to whether resuscitation should be attempted in the event of a cardiac arrest. A national cardiac arrest audit undertaken in Sri Lanka in 2015 reported a high ratio of resuscitation attempts to deaths and poor outcomes following those attempts [1].

**Objectives:** To explore the characteristics of in hospital CPR practices, the use of Do Not Attempt Resuscitation (DNAR) orders and the perspectives of junior doctors involved in those attempts.

**Methods:** A cross-sectional telephone survey aimed at all consultant led medical and surgical wards in secondary and tertiary hospitals in Sri Lanka. Junior doctor interviews explored the practices and outcomes following CPR attempts, their perceptions regarding occurrence of cardiac arrest and probability of successful return of spontaneous circulation (ROSC) along with the use of DNAR orders.

**Results:** 82 (338 wards) of the 90 hospitals included were successfully contacted. The remaining 8 hospitals were not reachable despite multiple attempts. 42 CPR attempts were reported. 16 (4.7 %) wards had at least one patient with an informal DNAR order. 3 CPR attempts were excluded as the doctor interviewed did not participate in the attempt.

42 deaths were reported. 8 deaths occurred without a known resuscitation attempt. Of these 6 deaths occurred on wards with an informal DNAR order in place.

Of the 39 attempted resuscitations 34 were immediately unsuccessful, 5 resulted in ROSC (3 sent to ICU for post-resuscitation care, whilst 2 remained on the ward). At 24 hours 2 (both in ICU) were still alive. Defibrillation was attempted in 5 cases. Intubation was attempted on 5 occasions. In 5 (13 %) of the resuscitation attempts CPR was the only intervention reported while 27 (69 %) received more than 1 vial of adrenaline, or defibrillation, and or intubation.

**Fig. 125 (abstract A1115).**
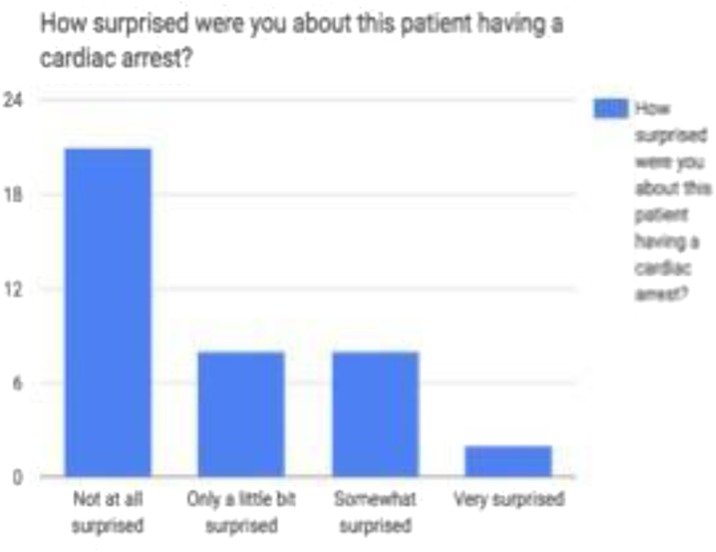
ᅟ

**Fig. 126 (abstract A1115).**
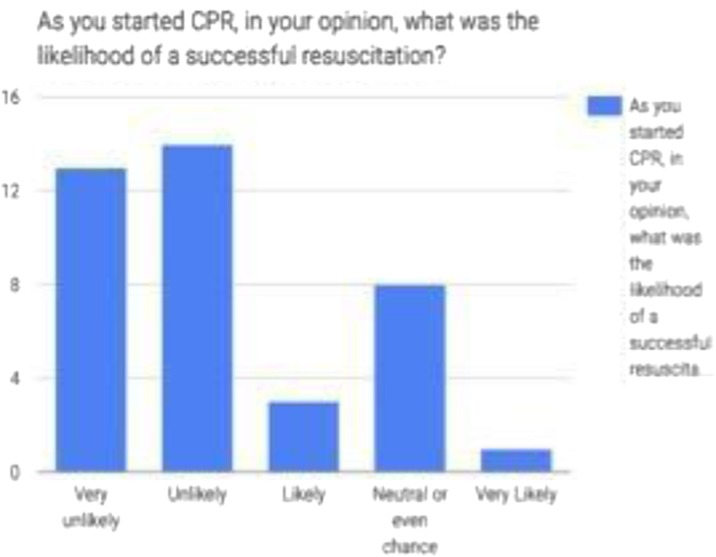
ᅟ

Interviewees reported that in 25 (64 %) of these patients they were 'not at all' or only a 'little bit surprised' by the patient having a cardiac arrest (Fig 125). They further described the chances of a successful outcome as 'unlikely or very unlikely 61 % of the time and likely or very likely only 10.3 % of the time (Fig 126).

**Conclusions:** Perspectives of junior doctors interviewed suggest many cardiac arrests were not a surprise and that the probability of ROSC following attempted resuscitation was unlikely. There is high incidence of patients receiving CPR attempts before death in hospitals across Sri Lanka with DNAR practices remaining uncommon. Outcomes remain poor, with ROSC after cardiac arrest being 12.8 % and survival at 24 hrs 5.1 %. Of the 34 unsuccessful resuscitation attempts, defibrillation and or repeated adrenaline was reported in 67.6 % of cases.

**References**

1. Available online at:https://clinicaltrials.gov/ct2/show/NCT02368392

### A1116 Are cardiorespiratory arrests in- and outside of the ICU similar?

#### A. Iglesias Santiago, V. Chica Sáez, R. de la Chica Ruiz-Ruano, A. Sánchez González

##### Hospital Virgen de las Nieves, Medicina Intensiva, Granada, Spain

###### **Correspondence:** A. Iglesias Santiago – Hospital Virgen de las Nieves, Medicina Intensiva, Granada, Spain

**Objectives:** Assessment of characteristics and outcomes of patients who suffer cardiopulmonary arrest resuscitated in a tertiary hospital, inside and outside Intensive Care Unit, according to Utstein style.

**Methods:** A prospective cohort study was performed according to Utstein style. Every arrest occurred in the hospital “Virgen de las Nieves” (Granada, Spain) for a period of 3 years (July/09-June/12) were included. All arrest occurred in all areas of the hospital were included, except those in operating rooms and anesthesia recovery room (not attended by the resuscitation team) and those commenced in the prehospital setting. We also excluded patients in whom no resuscitation attempt was made or those suspended either by existence of a living will, by orders DNR or considered futile. The variables were grouped according to the location (inside or outside the ICU). Chi^2^ test was performed when the dependent variable was qualitative and a t-Student test when it was quantitative.

**Results:** During this period a total of 297 patients suffered at least one episode of arrest and they were resuscitated. Most frequent sex was male (61.3 %) with a median age 69 years (65.2 ± 14.2 years; interquartile 57–76 years). The cardiac origin was the most common aetiology (40.4 %). The ICU was the area most frequent location (43.8 %). When comparing the characteristics of ICU arrests with the rest of the hospital, significant differences were observed. It was most likely to have a shockable initial rhythm (χ2: 10.6; p = 0.004), younger age (62,1 ± 14,9 vs 67,5 ± 13,2 years; t = 3,3; p = 0,001), shorter interval to defibrillation (2,1 ± 2,3 vs 4,7 ± 2,4 min; t = 3,6; p = 0,001), shorter period until start of resuscitation (1,3 ± 2,3 vs 4,9 ± 4,3 min; t = 8,9; p < 0,001) and shorter total duration (15 ± 15,2 vs 24,7 ± 19,5 min; t = 4,4; p < 0,001). However, no differences were found in coronary aetiology, sex, recovery of spontaneous circulation and hospital survival (22.1 % vs 23.8 % in ICU).

**Conclusions:** Despite higher frequency in initial shockable rhythms and lower intervals until defibrillation and resuscitation in the ICU, no differences were found in initial recovery or hospital survival.

### A1117 Delayed onset of cardio-pulmonary resuscitation (CPR) does not induce hyperfibrinolysis in a piglet model of ventricular fibrillation - a pilot study in Göttingen Minipigs

#### N. Kunze-Szikszay^1^, S. Wand^1^, P. Klapsing^1^, A. Wetz^1^, T. Heyne^1^, K. Schwerdtfeger^1^, M. Troeltzsch^2^, M. Bauer^1^, M. Quintel^1^, O. Moerer^1^

##### ^1^University Medical Centre Göttingen, Department for Anaesthesiology, Göttingen, Germany; ^2^University Medical Centre Göttingen, Department for Oral and Maxillofacial Surgery, Göttingen, Germany

###### **Correspondence:** N. Kunze-Szikszay – University Medical Centre Göttingen, Department for Anaesthesiology, Göttingen, Germany

**Introduction:** Pro-coagulatoric effects after cardiac arrest and consecutively appearing microthromboses have been considered major contributors to morbidity and mortality after CPR [1]. In contrast, recently published data suggest that 35–53 % of patients after out-of-hospital cardiac arrest (OHCA) present with hyperfibrinolysis during and after CPR [2;3]. The interpretation of these inconsistent observations remains unclear and complicated, because of methodological differences and lacking analytical approach in the underlying studies. Fibrinolytic activation might be the physiological reaction to restore perfusion after hypoperfusion due to microthromboses. This leads to the question, if the duration of no-flow (time without chest compressions) after cardiac arrest influences the level of coagulation activation and subsequent fibrinolysis during CPR.

**Objective:** To investigate the influence of a delayed onset of CPR on the extent of fibrinolysis and the function of the coagulation system measured by rotational thrombelastometry (ROTEM).

**Methods:** After approval of the local authorities (Nds. LAVES, approval G13-1088) cardiac arrest was induced in 10 anaesthetized female Göttingen Minipigs via rapid ventricular overpacing resulting in ventricular fibrillation (VF). In order to simulate a BLS-CPR in 5 animals (CPR-Group), chest compressions (CC) and ventilation were started after 5 min of VF (30:2-ratio, FiO_2_ = 0.21). In order to simulate consecutive ALS-CPR, continuous CC (100 min^−1^) and ventilations (15 min^−1^, FiO_2_ = 1.0) were started 5 minutes later. No CPR was started in the remaining 5 pigs (Non-CPR-Group). Blood samples for a complete ROTEM analysis (ROTEM delta® analyzer, Tem Int. GmbH, Munich, Germany) and laboratory analyses were taken before induction of VF (baseline) and 10, 20 and 30 min after VF. All parameters were investigated for normal distribution (Shapiro-Wilks-Test). Statistical significance of differences (p < 0.05) was investigated using the unpaired t-test (normal distributed parameters) and the Mann-Whitney-U-Test (not-normal distributed parameters).

**Fig. 127 (abstract A1117).**
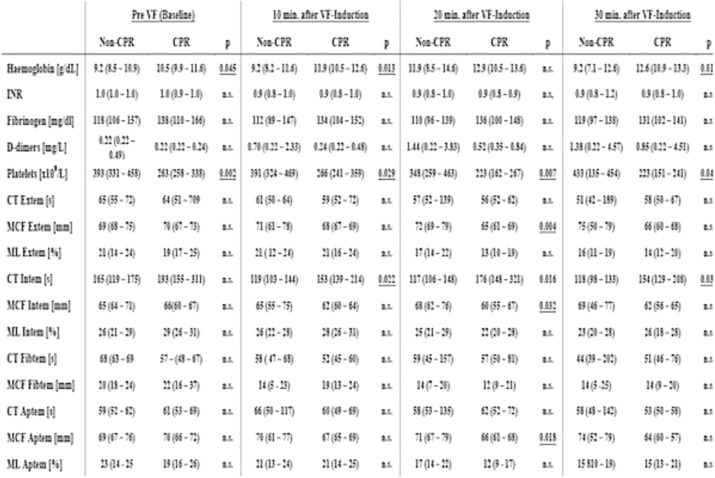
ROTEM and laboratory results

**Fig. 128 (abstract A1117).**
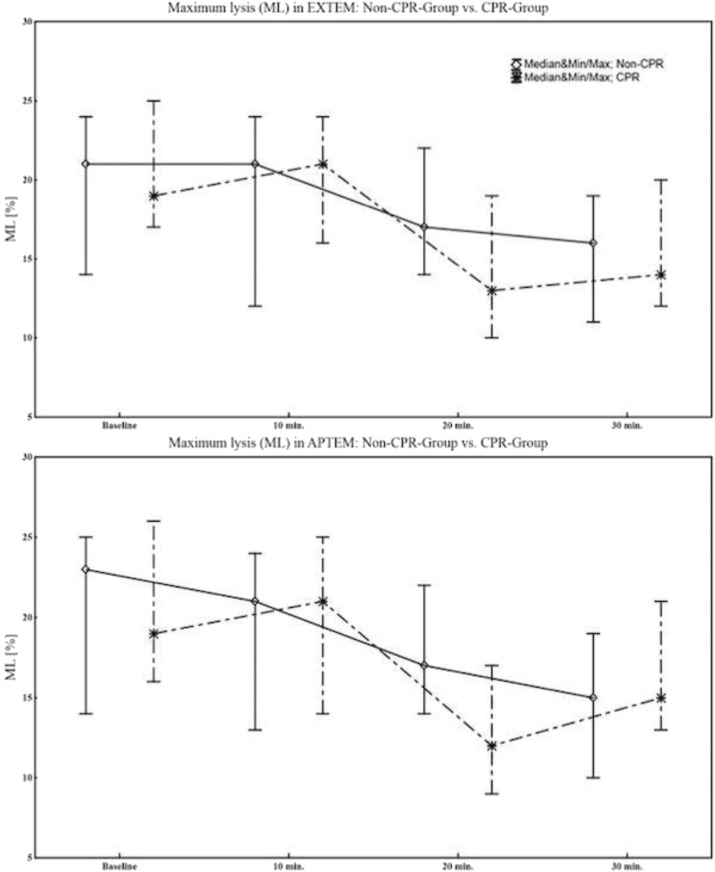
ML in EXTEM & APTEM

**Fig. 129 (abstract A1117).**
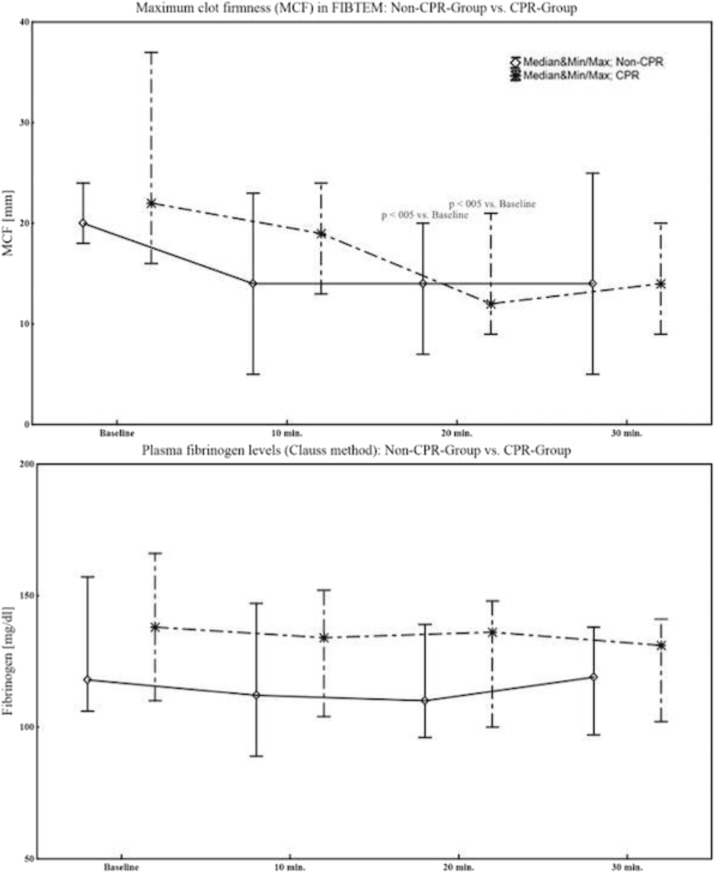
MCF FIBTEM & Plasma Fibrinogen

**Results:** Figure 127 summarizes laboratory and ROTEM results. In no group maximum lysis increased significantly after cardiac arrest (Figure 128). Maximum clot firmness (MCF) in FIBTEM analyses decreased significantly in both groups (Figure 129), but plasma fibrinogen levels (measured using the Clauss method) remained stable.

**Conclusions:** Delayed onset of CPR does not lead to severe HF visible in ROTEM. Further analytical research is needed to answer the question what triggers HF during CPR. Plasma fibrinogen levels during CPR might be overestimated by the Clauss method.

**References**

[1] Böttiger et al. (1995); Circulation; 92(9): 2572–78

[2] Viersen et al. (2012); Resuscitation; 83(12): 1451–55

[3] Schoechl et al. (2013); Resuscitation; 84(4): 454–59

**Grant Acknowledgement**

Financed by departmental funds. Authors thank Tem International for providing a ROTEM analyzer for the study.

## ICU ORGANISATION

### A1118 Methodological quality and reporting of cluster randomized controlled trials in critical care medicine: a systematic review

#### D.J. Cook, W.B. Rutherford, D.C. Scales, N.K. Adhikari, B.H. Cuthbertson

##### Sunnybrook Health Sciences Centre, Critical Care, Toronto, Canada

###### **Correspondence:** D.J. Cook – Sunnybrook Health Sciences Centre, Critical Care, Toronto, Canada

**Introduction:** Compared to individual-patient randomized controlled trials (RCTs), the design, conduct, and analysis of cluster (c)RCTs involve unique methodological and ethical considerations. The CONSORT cRCT recommendations, published in 2004 (updated 2012), provide a benchmark to assess this.(1)

**Objectives:** We sought to evaluate the rationale, methodological quality, and reporting of cRCTs in critical care medicine (CCM).

**Methods:** We systematically identified all cRCTs, of any intervention, in intensive care units using Medline, Embase, and Cochrane Central Register of Controlled Trials (2005-present). Two reviewers independently screened citations, reviewed full texts, protocols, and supplements of potentially eligible studies for inclusion, abstracted data, and assessed methodology.

**Fig. 130 (abstract A1118).**
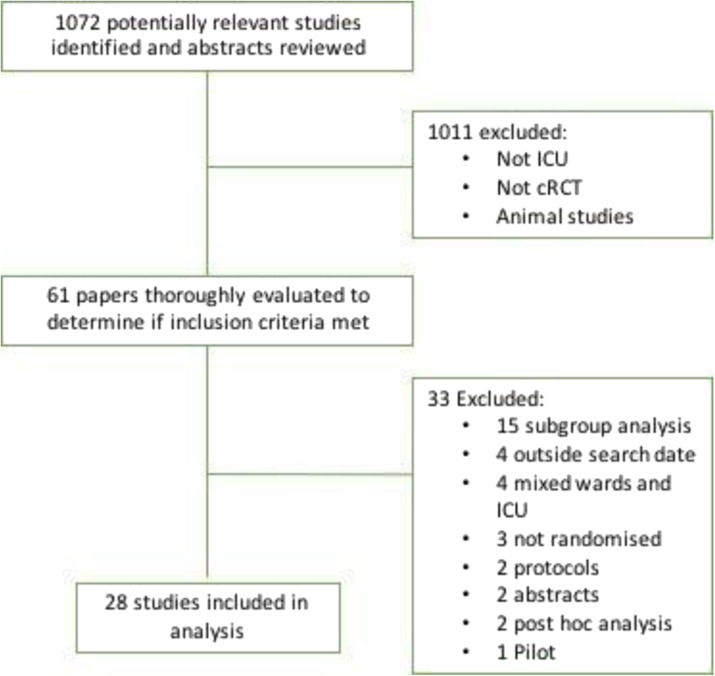
Consort Diagram

**Results:** From 1072 unique citations, 28 (3 %) cRCTs met selection criteria (Fig 130). Most focused on quality improvement (37 %), antimicrobial (22 %), or infection control (26 %) interventions. Designs included parallel arm (48 %), crossover (44 %), and stepped-wedge (7 %). The median number of clusters was 14 (IQR 7.5-28.5); median total sample size 2449 (IQR 1098–6805); median patients per cluster 356 (IQR 90–706). Most (70 %) cRCTs obtained waivers of consent.

96 % of studies reported sample size calculations. These were adequately described in 22 (79 %). Of 24/28 studies using individual patients as the unit of analysis, 13 (54 %) reported an estimate of intracluster coefficient [ICC; median 0.025 (IQR 0.01-0.071)].

In 4/28 (14 %) studies the number of clusters and patients required to achieve a pre-specified effect size was identified; 4/4 (100 %) achieved these numbers.

In 14/28 (50 %) a fixed number of clusters were identified at trial commencement and the number of patients required to achieve a pre-specified effect size was then identified; 8/14 (57 %) achieved these numbers.

In 3/28 (11 %) a fixed number of clusters and an anticipated number of patients were identified at trial commencement and a range or point estimate of detectable effect sizes were identified; 2/3 (66 %) achieved these numbers.

When analyzing data, few (2/28, 7 %) studies failed to consider the effects of clustering; 4/28 (14 %) used the cluster as the unit of analysis.

Blinding was reported in 14/28 (50 %) studies. Physicians and data analysts were blinded in 8 %, no studies reported blinding of patients. 14/28 (50 %) cRCTs reported adequate concealment of randomization.

Only 2/28 (7 %) of studies reported loss of a whole cluster after randomization, but 9/28 (32 %) reported a loss of participants. 39 % reported potential Hawthorne effects.

**Conclusion:** Cluster RCTs in CCM typically involve a small and fixed number of relatively large clusters, and are frequently underpowered relative to initial sample size estimates. The reporting of key methodological aspects of these trials is often inadequate.

**References**

1. The CONSORT Extension for Cluster Trials. http://www.consort-statement.org/extensions. Accessed on 14/04/16.

**Fig. 131 (abstract A1118).**
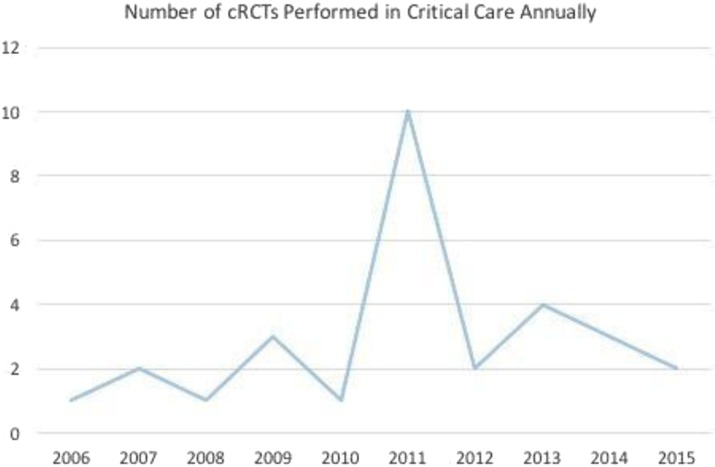
cRCTs in CCM Per Year

### A1119 Volume of extracorporeal life support in Japan: analysis of a nationwide administrative database

#### T. Suzuki^1^, T. Takei^1^, K. Fushimi^2^, M. Iwamoto^3^, S. Nakagawa^4^

##### ^1^Yokohama City Minato Red Cross Hospital, Department of Emergency and Critical Care Medicine, Yokohama, Japan; ^2^Tokyo Medical and Dental University, Health Policy and Informatics Section, Department of Health Policy, Tokyo, Japan; ^3^Center for Cancer Control and Information Services, Division of Health Services Research, Tokyo, Japan; ^4^National Center for Child Health and Development, Department of Critical Care, Tokyo, Japan

###### **Correspondence:** T. Suzuki – Yokohama City Minato Red Cross Hospital, Department of Emergency and Critical Care Medicine, Yokohama, Japan

**Introduction:** There are no standard rules for referral of patients treated with extracorporeal membrane oxygenation (ECMO) in Japan.

**Objectives:** To investigate the overall picture of patient volume and distribution of ECMO care.

**Methods:** We used the administrative claims data of all Diagnosis Procedure Combination (DPC) hospitals in Japan from April 1, 2011 to March 31, 2012, and retrospectively reviewed the number and outcome at discharge of patients who were on ECMO support.

**Results:** We identified 3,336 patients who received ECMO support during the 1-year study period. The average age was 58.7, and only 6.7 % of the patients were under 20 years old. The most common diagnosis was acute coronary syndrome, followed by cardiac arrest and pulmonary embolism. The overall survival rate at discharge was 39.6 % [95%CI 38.0-42.3]. Among the 1,449 acute care DPC hospitals, ECMO support was provided in 394 hospitals (27.2 %), and therefore the annual ECMO patient volume per hospital was 8.5, which is much lower than international standards for ECMO centers. Adjusted odds ratio for discharge alive stratified by annual ECMO volume per hospital were 1.10 [95%CI 0.78-1.54] and 1.23 [95%CI 0.90-1.28] for medium (131 hospitals treating 4 to 9 patients) and high volume centers (122 hospitals treating 10 or more patients), respectively, compared with low volume centers (141 hospitals treating 3 or less patients).

**Conclusions:** ECMO support was administered to many adult cardiac patients, and provided in a substantial proportion of acute care hospitals in Japan. No significant patient volume effect for survival discharge was found.

**References**

1. Extracorporeal Membrane Oxygenation for ARDS in Adults. N Engl J Med 2011; 365:1905–1914.

2. Cardiopulmonary resuscitation with assisted extracorporeal life-support versus conventional cardiopulmonary resuscitation in adults with in-hospital cardiac arrest: an observational study and propensity analysis. Lancet. 2008;372:554–61.

3. ECLS Registry Report. https://www.elso.org/Registry/Statistics/InternationalSummary.aspx.

**Grant acknolwedgment**

There are no grants.

### A1120 A nationwide census of icu beds and admissions in Mongolia

#### N. Mendsaikhan^1^, T. Begzjav^1^, G. Lundeg^2^, M.W. Dünser^3^

##### ^1^Intermed Hospital, Intensive Care Department, Ulaanbaatar, Mongolia; ^2^Health Sciences University of Mongolia, Division of Emergency Medicine and Anaesthesia, Ulaanbaatar, Mongolia; ^3^University Hospital Salzburg and Paracelsus Private Medical University, Department of Anesthesiology, Perioperative and General Intensive Care Medicine, Salzburg, Austria

###### **Correspondence:** N. Mendsaikhan – Intermed Hospital, Intensive Care Department, Ulaanbaatar, Mongolia

**Introduction:** Despite of a high burden of critical illness in middle- and low-income countries, management of critically ill patients faces serious challenges in these regions of the world (1). Little is known about national intensive care capacities and the burden of critical illness in low- and middle-income countries.

**Objectives:** To determine the intensive care unit (ICU) capacity and number of ICU admissions for adults and children in 2014.

**Methods:** A census of all hospitals with an ICU in the Mongolian capital city and 21 provinces was performed. Data on adult and pediatric ICU capacity and the number of ICU admissions in 2014 were collected.

**Results:** In total, 70 ICUs with 349 ICU beds were counted (11.7 ICU beds/100,000 inhabitants; 1.7 ICU beds/100 hospital beds). Of these, 241 (69 %) were adult (8.1 adult ICU beds/100,000 inhabitants) and 108 (31 %) pediatric ICU beds (3.6 pediatric ICU beds/100,000 inhabitants). Mechanical ventilators were available for approximately half of the ICU beds (5.1 mechanical ventilators/100,000 inhabitants; 3.6 adult mechanical ventilators/100,000 inhabitants; 1.5 pediatric mechanical ventilators/100,000 inhabitants). While all provincial hospitals ran a pediatric/neonatal ICU, only dedicated pediatric hospitals in the capital city did so. In 2004, we recorded 17,749 ICU admissions (10,676 adult ICU admission; 357 adult ICU admissions/100,000 inhabitants; 1.43 % of all adult hospital admissions; 7,073 pediatric ICU admissions; 237 pediatric ICU admissions/100,000 inhabitants; 0.95 % of all pediatric hospital admissions). The number of adult and pediatric/neonatal ICU admissions varied between provinces. The number of adult ICU beds and adult ICU admissions per 100,000 inhabitants were correlated (Spearman correlation coefficient, 0.5; p = 0.02), while the number of pediatric/neonatal ICU beds and pediatric/neonatal ICU admissions per 100,000 inhabitants were not (Spearman correlation coefficient, 0.25; p = 0.26).

**Conclusions:** With 11.7 ICU beds per 100,000 inhabitants the ICU bed capacity in Mongolia compares favorably with other low- and middle-income countries. Substantial heterogeneities in the standardized ICU bed capacity and ICU admissions exist between Mongolian provinces. Availability of mechanical ventilators for only half of the ICU beds indicates important resource restrictions in Mongolian ICUs. Pediatric/neonatal ICU beds make up one third of the national ICU capacity and appear to meet or even exceed the demand of pediatric/neonatal critical care in Mongolia.

**References**

1. Dondorp AM, Iyer SS, Schultz MJ (2016) Critical care in resource-restricted settings. JAMA 315:753–754.

### A1121 Perception and knowledge of intensive care unit (ICU) staff about self - protection and evacuation plans

#### D. González Romero^1^, J.L. Santana Cabrera^1^, J.D. Martín Santana^2^, Y. Santana Padilla^1^, H. Rodríguez Pérez^1^, R. Lorenzo Torrent^1^

##### ^1^Hospital Insular Las Palmas GC, Las Palmas de Gran Canaria, Spain; ^2^Hospital Insular Las Palmas GC, Department of Economy. University of Las Palmas de Gran Canaria, Las Palmas de Gran Canaria, Spain

###### **Correspondence:** H. Rodríguez Pérez – Hospital Insular Las Palmas GC, Las Palmas de Gran Canaria, Spain

**Introduction:** Perception and knowledge of hospital staff involved in an emergency evacuation of hospitalized patients is usually low. This is especially remarked in an ICU due to the complexity of moving patients who depend on invasive monitoring and organ support due to acute illness.

**Objectives:** To analyze the differences between different members of ICU staff about their perception and knowledge of self-protection and evacuation plans.

**Methods:** A quantitative, descriptive and cross-sectional study was carried out by a fully structured and self-administered survey in 12 public and private ICU staff through a total sample of 434 participants. They were asked to complete a questionnaire about their perception and knowledge of self-protection and evacuation plans in the ICU. This study pretended to analyze the differences between participants, taking into account their demographic and occupational characteristics and their level of satisfaction and commitment to their jobs.

**Results:** On a rating scale from 1 to 7, ICUs workers perceive that their preparation and knowledge were too low to meet a possible emergency that could require an evacuation, although they were aware of the need to make an update. However, they state that the different ICUs where they work do not have these plans, and, consequently, they do not feel prepared to act in an emergency situation, even though they think this type of situation may occur. The significant differences (p < 0,000) were observed when levels of satisfaction and commitment to their jobs were high.

**Conclusions:** The results of this study showed that there is a need for more knowledge in the area of emergency training. This should be the basis for the development of educational programs and also promoting awareness of ICU staff on self-protection and evacuations plans.

**References**

1. King MA, Niven AS, Beninati W, et al. Grissom; on behalf of the Task Force for Mass Critical Care. Evacuation of the ICU. Care of the Critically Ill and Injured During Pandemics and Disasters: CHEST Consensus Statement. CHEST 2014; 146(4_Suppl ): e44S -e60S.

2. Murphy GRF, Foot C. ICU fire evacuation preparedness in London: a cross-sectional study. British Journal of Anaesthesia 2011 March;106(5):695–698.

3. Kelly FE, Hardy R, Hall EA. Fire on an intensive care unit caused by an oxygen cylinder. Anaesth 2013;68:102–104.

### A1122 Integrating nurse practitioners and physician assistants in the ICU: results of a national survey

#### R. Kleinpell

##### Rush University Medical Center, Chicago, United States

**Introduction:** An increasing number of intensive care units (ICUs) are integrating advanced practice providers (APP) including nurse practitioners (NPs) and physician assistants (PAs) to meet workforce demands to care for acute and critically ill patients. Although these roles are established ones, limited information is known about the specific care models used in ICU settings. This information is crucial in objectively evaluating the effectiveness of APP roles.

**Objectives:** To address this gap, a national survey was conducted targeting NPs and PAs, including those working in ICU settings.

**Methods:** A web-based survey was used to assess 4 domains including role components (i.e. direct care management; care coordination; performing procedures; education; quality assurance; research); role responsibilities (i.e. practice autonomy, prescriptive authority, credentialing and privileging delineations) unit-level organization (i.e. physician staffing models, components of the multidisciplinary care team); and hospital organization (i.e. academic status, bed size, location, payer-mix).

**Results:** A total of 1851 APPs responded to the national survey including 1350 NPs and 491 PAs. The respondents reported working in a variety of settings including hospitals, clinics, urgent care centers and specialty practice sites. A total of 636 reported working in an ICU setting. Of these, 286 reported 24/7 coverage of ACNPs and PAs in the ICU. Main role components included patient care management as part of the multiprofessional ICU team; teaching to patients, families and healthcare staff; involvement in quality improvement and research initiatives and administrative components such as committee work. Specific aspects include conducting history & physical exams, ordering and interpreting diagnostic test/labs; providing care coordination, performing specialty procedures such as wound care or other specialty care. Major areas of impact that were identified included continuity of care, improving evidence based practice care, reducing hospital length of stay, preventing hospital readmissions and promoting patient, family and staff satisfaction.

**Conclusions:** The results of the study provide information from a large national sample of NPs and PAs that identifies the comprehensive care components of the role as well as areas of impact, highlighting the value of APP care. Globally, this information can be useful to other countries who are considering use of NPs and PAs in the ICU.

**Acknowledgement**

Funding from the American Nurses Association Impact Grant is gratefully acknowledged.

### A1123 Online questionnaire survey on which criteria guide intensivists´ decisions about admission or refusal of admission in greek public intensive care units

#### I. Chouris^1^, V. Radu^1^, M. Stougianni^1^, A. Lavrentieva^2^, D. Lagonidis^1^

##### ^1^Giannitsa General Hospital, Intensive Care Unit, Giannitsa, Greece; ^2^G. Papanikolaou Hospital, A' Intensive Care Unit, Thessaloniki, Greece

###### **Correspondence:** I. Chouris – Giannitsa General Hospital, Intensive Care Unit, Giannitsa, Greece

**Introduction:** Critically ill patients are a growing and demanding population that represents a significant consumption of the Greek national health care resources. With 470 ICU beds, the reserves of the system are often overwhelmed. The responsibility for a rational management and distribution of these costly resources burdens the admitting Intensivist.

**Objectives:** To assess the factors that affect and guide the Intensivists' decisions about ICU admissions in Greek public hospitals.

**Methods:** Online anonymous questionnaire survey (survs.com), available through the site of the Greek Critical Care Society (icu.gr), involving ranking of factors based on importance (scale of 1–5, 5 signifying the most important) and related questions.

**Results:** Fifty-nine out of (estimated) three hundred Intensivists responded and completed the questionnaire. Respondents were Intensivists of 5 different basic specialties: Pulmonologists (21), Internists (15), Anesthesiologists (15), Surgeons (6) and Cardiologists (2). The average years of practice in intensive care was 13,77. Thirty-four of the respondents work at a tertiary hospital (58 %).

Regarding admission to the ICU, the four most important (ranked 4–5) factors were: bed availability (93 %), prognosis of underlying disease (82 %), prognosis of acute illness (75 %) and availability of necessary medical specialty (72 %).

Least important factors (ranked 1–2) were: patient's socioeconomic profile (88 %), religious convictions (81 %), drug abuse (79 %), severe psychiatric illness (78 %).

The most decisive criteria leading to refusal of ICU admission were: prognosis of underlying disease (59 %), bed availability (49 %), previous functional condition (42 %) and lack of necessary medical specialty (41 %).

Decisions about admission in the ICU are mostly individualized (85 %), with department protocols being of limited use (15 %).

Admission protocols are based on vital signs (45 %) and to a lesser extent on prognostic (27 %) and diagnostic (18 %) criteria. Scoring systems (APACHE II etc.) account for 9 %.

Intensivists not using protocols expressed a strong desire (79 %) to introduce protocol based criteria for admission.

**Conclusions:** The most important factors influencing decisions about admission (or refusal of admission) in the ICU are bed availability and prognosis of the underlying disease. Socioeconomic and religious criteria are clearly of marginal significance. Drug abuse and severe psychiatric disease do not emerge as compelling causes of biased decisions. It appears that the Intensivist's decisions are largely individualized, as application of admission protocols is limited among the ICUs. However, the responses documented in this survey strongly indicate that introduction of such protocols would be welcome by a majority of our colleagues.

**References**

1. Escher M et al. National questionaire survey on what influences doctors; decisions about admission to intensive care, BMJ 2004;329(7463):425

### A1124 An audit of the practices of parent team reviews of critical care patients

#### R.D.T. Price, A. Day, N. Arora

##### Heart of England NHS Foundation Trust, Birmingham, United Kingdom

###### **Correspondence:** R.D.T. Price – Heart of England NHS Foundation Trust, Birmingham, United Kingdom

**Introduction:** On an Intensive Therapy Unit (ITU) in the UK, it is recommended that care is led by a Consultant Intensivist^1^. Current standards state that consultants need to have appropriate information to make care decisions. They also stress the importance of continuity of care following discharge from critical care.

The Royal College of Physicians recommends that patients should have a consultant review within 24 hours of transfer from an Acute Medical Unit^2^. This seems a reasonable time frame for a parent team review following an ITU admission.

**Objectives:** This audit aimed to identify the proportion of patient's admitted to ITU who were reviewed by their parent team whilst on ITU. Our standards were that 100 % of patients should have documentation of a discussion with their parent consultant prior to ITU admission and 100 % of patients admitted to ITU should have a parent consultant review within 24 hours of admission.

**Methods:** This prospective audit was conducted in a district general hospital ITU. It was originally performed in 2015 over a two week period and was re-audited in January 2016 over a period of one calendar month. Data were collected from patient case notes.

**Results:** In the initial audit, 12/16 (75 %) patients were seen by a medical consultant prior to ITU admission and 5/16 (31.3 %) patients were reviewed within 24 hours of admission. In the re-audit, the parent consultant was aware of the ITU admission prior to it in 25/40 (62.5 %) cases and 25/40 (62.5 %) patients were reviewed by their parent consultant within 24 hours of admission.

**Conclusions:** In this loop closing audit, the percentage of patients reviewed by their parent consultant within 24 hours had increased from 31.3 % to 62.5 %. However the percentage of patients with consultant input or awareness prior to ITU admission had decreased from 75.0 % to 62.5 %. There were differences in sample size between the audits and the first included medical patients only, where the second also included surgical patients. In addition, a parent consultant review at baseline was defined as a review within 24 hours in the re-audit, whereas this was not the case in the initial audit. Overall however, in both audits neither the standards of 100 % for documentation of discussion with parent consultant prior to ITU admission nor for consultant review within 24 hours of ITU admission were met. Medical and surgical consultants should be encouraged to review their patients on ITU and further efforts should be made by ITU teams to inform parent consultants of a patient's admission to ITU when they are unaware of this.

**References**

1. Danbury CM et al. Core Standards for Intensive Care Units (1st ed). The Faculty of Intensive Care Medicine/The Intensive Care Society. 2013

2. Acute care toolkit 2. High-quality acute care. Royal College of Physicians. 2011

**Grant acknolwedgment**

No grant application was made for this project.

### A1125 The availability of inhaled nitric oxide in general intensive care units - a national Scottish survey

#### M.A. Henderson^1^, S. Hickey^2^

##### ^1^Queen Elizabeth University Hospital, NHS GG&C, Intensive Care Unit, Glasgow, United Kingdom; ^2^Golden Jubilee National Hospital, Department of Anaesthesia, Clydebank, United Kingdom

###### **Correspondence:** M.A. Henderson – Queen Elizabeth University Hospital, NHS GG&C, Intensive Care Unit, Glasgow, United Kingdom

**Introduction:** Congenital cardiac disease is increasingly a disease of adulthood - 90 % of patients live beyond adolescence. [1] Consequently, there is a need for general (non-cardiac) Intensive Care Units (ICUs) to facilitate more elective and emergency surgery for these patients. Inhaled nitric oxide, a selective pulmonary vasodilator, may be required for this purpose.

**Objectives:** We wished to determine the availability of inhaled nitric oxide in general Scottish ICUs.

**Methods:** In 2015 we surveyed the 20 general adult ICUs in Scotland. [2] We excluded tertiary paediatric, cardiothoracic and neuro-critical care units. An online survey was distributed followed, if necessary, by a telephone survey. Caldicott Guardianship approval was not required.

**Results:** Four (20 %) general ICUs had nitric oxide immediately available. Two units (10 %) had an established means by which nitric oxide could be obtained from other hospitals. In units where nitric oxide was immediately available the estimated usage was 1–4 times per annum (median 2.5). These units reported that they were comfortable administering nitric oxide with a median “comfort score” of nine (range 8–9 of an incremental 1–10 score). Fourteen (70 %) were unable to administer nitric oxide.

**Conclusions:** It is essential that non-cardiac surgery should be delivered in the most appropriate clinical setting. In Scotland, adults with moderate to complex congenital cardiac disease are managed by the Scottish Adult Congenital Cardiac Service (SACCS), based at the Golden Jubilee National Hospital (GJNH), near Glasgow. [3] Existing guidelines have established when patients should have elective non-cardiac surgery performed at GJNH. However, many surgical specialties are not routinely available at GJNH, the bed occupancy rate is high and with an increasing SACCS population there is a need for appropriate patients to receive optimal care at their base hospital. Additionally, urgent and emergency non-cardiac surgery ought to be performed at the base hospital. While nitric oxide is a core cardiac therapy we have shown that it is scarce in Scotland and unfamiliar to many ICUs. There is a need for a national discourse and consensus to ensure that nitric oxide is more widely available as part of a bundle of optimal cardiac critical care. This should include education, material resources, clinical guidelines and perhaps cardiac critical care outreach services to support general ICUs.

**References**

1. Kelleher A. Adult congenital heart disease (grown-up congenital heart disease). Continuing Education in Anaesthesia Critical Care and Pain. 2012;12:28–32.

2. Scottish Intensive Care Society Audit Group [Internet]. Audit of Critical Care in Scotland 2015 Reporting on 2014. [Cited 20th August 2015] Available from: www.sicsag.scot.nhs.uk/Publications/Main.htm

3. Scottish Adult Congenital Cardiac Service [Internet]. [Cited 30th September 2015] Available from: www.saccs.info

### A1126 Time spent in the emergency department and outcomes for critically ill patients

#### M.I. Almeida Costa^1^, J.P. Carvalho^2^, A.A. Gomes^1^, P.J. Mergulhão^3^

##### ^1^Centro Hospitalar de S.João, Porto, Portugal; ^2^University of Porto, Porto, Portugal; ^3^Centro Hospitalar de São João/University of Porto, Porto, Portugal

###### **Correspondence:** M.I. Almeida Costa – Centro Hospitalar de S.João, Porto, Portugal

**Introduction:** Scarcity of Intensive Care Unit (ICU) beds has long been a problem. Among other things, it increases the work load of Emergency Department (ED), contributing to its crowding and probably to worst care, jeopardizing outcomes. Despite the plausibility of this premise, studies aren't consensual about the impact on outcome of delayed ICU admission from ED.

Hospital de São João is a Portuguese tertiary care center. ED receives around 150 000 adult admissions per year, and is spatially organized according to Manchester triage priorities. Emergency Room (ER) receives patients from the street and all patients from other areas of the ED that need critical care. It is staffed by trained personnel and is equipped with level III ICU material. Intensive care department is composed by 37 level III and 28 level II ICU beds

**Objectives:** Assess if there's a link between time spent in ED and outcome of patients admitted to level II and/or III ICU beds.

**Methods:** This is a retrospective study analysing older than 18 years old patients admitted to ICU from ED from 1^st^ January to 31^st^ December 2014. We excluded patients transferred from other hospitals. Demographic and clinical data was collected from records. We selected hospital outcome (dead, alive, transferred), hospital length of stay, ICU length of stay, vital status at 28 and 90 days after admission and ED and ER duration as outcomes. Simplified Acute Physiologic Score (SAPS) II and Sequential Organ Failure Assessment (SOFA) were calculated by considering the worst values in the first 24 hours of hospital admission. We performed a descriptive analysis, with median and interquartile ranges presented for continuous variables and proportions for categorical variables. For analysis of subgroups we did a chi-square or Mann-Whitney test. Statistical analyses were done on IBM-SPSS (version 20). A p-value of < 0,05 was considered significant.

**Results:** 147239 adults were assisted in ED in this period, with a median length of stay of 434 minutes. 500 were admitted to ICU beds, which accounts for 0,34 % of all adults cared for in ED. Around 66 % of patients admitted in ICU were treated in the ER at some point of their ED care.

Patients admitted to ICU stayed around 470 minutes in ED. The more severe the disease, the least time spent (p = 0,001). Patients treated in ER were significantly more likely to be admitted quickly in ICU (p < 0,001). Taking in consideration the time spent in the ED, we found an opposite relation with global outcome, meaning that patients staying longer periods in ED had lower ICU mortality and lower length of stay in ICU. There was no association with hospital mortality.

**Conclusions:** Time spent in ED had no negative impact on outcome. However, given the fact that the majority of patients admitted to ICU beds were cared in a devoted area with trained staff and full level III equipment, we hypothesize that what might impact the most on outcome is provision of early critical care.

### A1127 Determination of ICU bed requirement using resampling

#### K.K.C. Chan^1^, H.P. Shum^2^, W.W. Yan^2^

##### ^1^Tuen Mun Hospital, Anaesthesia & Intensive Care, Hong Kong, Hong Kong, China; ^2^Pamela Youde Nethersole Eastern Hospital, Intensive Care, Hong Kong, Hong Kong, China

###### **Correspondence:** K.K.C. Chan – Tuen Mun Hospital, Anaesthesia & Intensive Care, Hong Kong, Hong Kong, China

**Introduction:** Planning for ICU-bed provision, with a statistical confidence level, required the average number of critically-ill patients, their average ICU length of stay (LOS), and the fluctuation/variance of these two parameters. The actual ICU bed occupancy would under-estimates the variance, as ICU could never exceed its full capacity. With an under-estimate, the predicted ICU bed requirement would be inaccurate, with a tendency of under estimation.

**Objectives:** Estimate the bed requirement to cover 97.5 % of time, by resampling of admission/discharge entries in 2014, for the two busiest ICUs in Hong Kong (~1600 admissions/year each)

**Methods:** We assumed that the chance of an ICU admission was identical in a period of four weeks before and after a certain date. Based on this assumption, a computer simulation of ICU admissions was performed as if the year 2014 happened again. In brief, we pooled patients admitted on a particular date in 2014, and those admitted on the same day of week in the previous four and subsequent four weeks. Then patients were randomly selected from the pool to simulate ICU admission on that particular date. A mechanism (not described here) was in place to handle the public holidays. The hourly ICU occupancy was calculated using the actual ICU LOS of the selected patient. Re-sampling for the whole year was repeated 200 times to provide the estimates required.

**Results:** The actual hourly medians of ICU occupancy were 82 % and 77 %. They were close to that obtained using resampling (77 % and 77 %). As predicted, the distributions of the actual occupancy were skewed to left, indicating a negative bias on the variance estimates. The observed standard deviations of the two ICUs' occupancy were 11.5 % and 8.6 % respectively. After resampling, the distributions became more symmetrical, and had higher standard deviations of 18.9 % and 16.9 % (both p = 0.000). The 97.5 percentile occupancy in reality were 95 % and 92 %, while that from resampling were significantly higher at 118 % and 112 % (both P = 0.000). This corresponded to three or four additional ICU beds in each ICU.

**Conclusions:** In conclusion, using a simple and conservative assumption, resampling could provide valuable insight for ICU bed planning.

### A1128 A model for estimating shortage in intensive care unit beds in source limited hospitals; a Delphi consensus based prospective cross-sectional study

#### B. Maghsoudi^1^, S.H. Tabei^2^, M. Masjedi^1^, G. Sabetian^1^, H.R. Tabatabaei^3^, A. Akbarzadeh^4^, Student Research Committee - Shiraz University of Medical Sciences

##### ^1^Shiraz University of Medical Sciences, Shiraz, Iran,Shiraz Anesthesiology and Critical Care Research Center, Shiraz, Islamic Republic of Iran; ^2^Shiraz University of Medical Sciences, Shiraz Anesthesiology and Critical Care Research Center, Shiraz, Islamic Republic of Iran; ^3^Department of Epidemiology, School of Public Health - Shiraz University of Medical Sciences, Shiraz, Islamic Republic of Iran; ^4^Student Research Committee - Shiraz University of Medical Sciences, Shiraz, Islamic Republic of Iran

###### **Correspondence:** S.H. Tabei – Shiraz University of Medical Sciences, Shiraz Anesthesiology and Critical Care Research Center, Shiraz, Islamic Republic of Iran

**Introduction:** The number of available intensive care unit (ICU) beds are limited while the request for the beds are high. Thus rationing the admission to ICU is necessary especially in developing countries where the resources are limited. Also models are needed to estimate and re-estimate regularly, shortage in the number of ICU beds in any hospital. In the current study we tried to design a model for estimating shortage in the number of intensive care beds in a developing country tertiary university hospital after an initial Delphi consensus study.

**Objectives:** Designing a model for estimating shortage in the number of ICU beds in a hospital.

**Methods:** Initially the standard indications for ICU triage were extracted from the literature. Four intensivists were served as steering committee and the initial questionnaire were further prioritized by 22 experts with three rounded Delphi method and formed a standardized checklist for ICU triage. Indications were considered as critical, important, and all indications.

Then a cross-sectional study being performed during a 1-month period from August to September 2013 for all admissions to Nemazee hospital, a tertiary healthcare center affiliated to Shiraz University of Medical Sciences. Cardiac, transplantation and pediatrics patients were excluded from the study, as to be studied separately.

The checklist were filled every day by an observing physician and any indications for ICU admission were marked in the questionnaire. Decision making for requesting ICU admission were performed by the specialized physicians of each ward regardless of the results of completed checklists.The results were further assessed according to the mentioned criteria and the reliability and viability was calculated. Finally assuming that there was no available ICU bed-days, the required ICU bed-days were compared with the total ICU bed-days of the hospitals, to estimate the shortage of ICU beds.

**Results:** Totally 893 patients were admitted and studied.The required bed-day regarding critical indications, important indications, and all indications for ICU admission was 561, 1083, and 1950. By comparing the required bed-days with available bed-days of the hospital, 30 beds were calculated as shortage of ICU beds.

**Conclusions:** The results of the current study indicate that our center has deficiency in the number of ICU beds. It seems that a checklist is not only useful for prioritizing patients but also it is useful for estimating the required number of the ICU beds.The actual number of shortage is greater as three group of patients were not included.

**References**

1. Triage of Patients Consulted for **ICU** Admission During Times of **ICU**-**Bed Shortage**. Orsini J, Blaak C, Yeh A, Fonseca X, Helm T, Butala A, Morante J. J Clin Med Res. 2014 Dec;6(6):463–8.

**Grant acknolwedgment**

Shiraz Anesthesiology and Critical Care Research Center, Shiraz University of Medical Sciences, Shiraz, Iran.

### A1129 Distribution and qualitative assessment of critical care facilities in Madhya Pradesh, India

#### S. Saigal^1^, A. Pakhare^2^, R. Joshi^3^

##### ^1^AIIMS Bhopal, Department of Trauma & Emergency, Bhopal, India; ^2^AIIMS Bhopal, Community and Family Medicine, Bhopal, India; ^3^AIIMS Bhopal, Medicine, Bhopal, India

###### **Correspondence:** S. Saigal – AIIMS Bhopal, Department of Trauma & Emergency, Bhopal, India

**Introduction:** There is paucity of information on organization of intensive care facilities in low-middle income countries (LMICs) [1]. In a LMIC such as India where health-systems are weak, the number of available ICU beds is expected to be low. There is no study from Indian subcontinent that has reported characteristics and distribution of existing ICUs.

**Objective:** We performed this study to understand the characteristics and distribution of ICUs in Madhya-Pradesh state of Central India. Various intensive care professional bodies have defined levels of ICU care based on set of services and functionalities available within a facility. During our pilot, we found many facilities were having functionalities corresponding to a higher level, yet lacking in a functionality that was expected at a lower level of ICU care. Hence we also aimed to develop a consensus scoring system and internally validate it to define levels of care and to improve health system planning and to strengthen referral networks in the State.

**Methods:** We obtained list of potential ICU facilities from various sources and then performed a cross-sectional survey by visiting each facility, and determining characteristics for each facility. We collected variables with respect to infrastructure, human resources, equipment, support services, procedures performed, training courses conducted and in-place policies or standard operating procedure documents (Table 90)

Facility with score of 5–10 was identified as Level 1; facility with score of 11–20 was identified as Level 2 and with score of 21–30 as level 3.

**Fig. 132 (abstract A1129).**
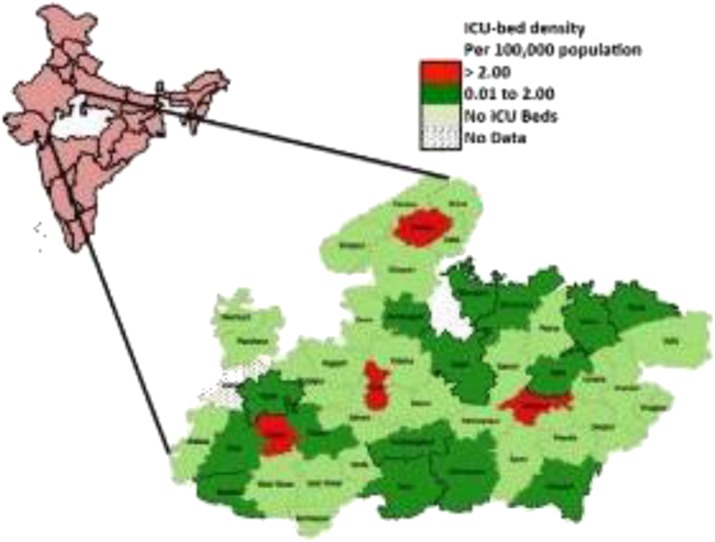
List of intensive care facilities in MP

**Results:** We identified a total of 123 ICUs in Madhya Pradesh. Out of 123 ICUs, 35 were level 1 facilities, 74 were level 2 facilities and only 14 were level 3 facilities (Figure 132)

Overall 85 (75 %) facilities were private for-profit, 9 (8 %) were private not-for profit, and 19 (17 %) were public sector facilities. Overall there were 0.17 facilities per 100,000 population (95 % 0.14 to 0.20 per 100,000 population). There were a total of 1816 ICU beds in the state, with an average of 2.5 beds per 100,000 population (95 % CI 2.4 to 2.6 per 100,000 population). Of the total number of ICU beds, 250 are in level 1, 1141 are in level 2 and 425 are in level 3 facilities. This amounts to 0.34, 1.57 and 0.59 ICU beds per 100,000 population for levels 1, 2 and 3 respectively.

**Conclusion:** ICU-bed density is low, and expected quality in ICUs is deficient in Madhya Pradesh state of India. Further with only a handful of designated ICU-beds in the public sector, this high-end care is out-of reach for most economically deprived individuals. Organization of ICUs needs to be strengthened.

**References**

1. Baker T: Critical care in low-income countries. Trop Med Int Health 2009; 14:143–8.

**Grant acknolwedgment**

Nil

**Table 89 (abstract A1129).** Scoring grid for intensive care units

Parameters	Score 1	Score 2	Score 3
No of ICU beds	6 or less	7–12	12 or more
Nurse to patient ratio	1:4 or less	1:3	1:2 or more
Invasive Ventilator to bed ratio	1:4 or less	1:3–1:2	1:1 or more
Qualification of Doctor in charge of intensive care facility	MBBS or Post-MBBS diploma	MD or MS	Post MD/MS intensive care qualification
Monitoring devices and advanced gadgets	Non-invasive monitors	Non-invasive and Invasive monitors	Non-invasive and Invasive monitors and advanced gadgets (Hemodialysis, IABP, advanced ventilators etc.)
Imaging facilities Laboratory facilities	Radiography/Ultrasonography Pathology and Clinical chemistry	Radiography/Ultrasonography Computerized tomography and Echocardiography Pathology and Clinical chemistry and Basic microbiology	Radiography/Ultrasonography Computerized tomography and Echocardiography & MRI Pathology and Clinical chemistry and Basic & advanced microbiology
Level of Procedures conducted	Peripheral and central venous cannulation	Venous and arterial cannulation.	Venous, arterial, and cannulation and percutaneous tracheostomy.
Policies and protocols	Standard operating protocols	Standard operating protocol and infection control policy	Standard operating protocol, infection control policy, and Ethics committee
Teaching and training facilities	Short term courses (upto 6 months)	Indian diploma in critical care (up-to 1 year)	DM in critical care (3 year course)

### A1130 Transfer delay from intensive care unit: retrospective analytical study in an Indian tertiary care hospital

#### S.K. Pattnaik^1^, B. Ray^2^

##### ^1^Apollo Hospitals, Critical Care Unit, Bhubaneswar, India; ^2^Apollo Hospitals, Bhubaneswar, India

###### **Correspondence:** S.K. Pattnaik – Apollo Hospitals, Critical Care Unit, Bhubaneswar, India

**Introduction:** There lies scarcity of Intensive Care Unit (ICU) beds in every tertiary care hospitals, and on top of it delayed transfer of patients from ICU to wards is further increasing the burdensome. Numerous factors affect in making delayed transfer, which in itself is a risk factor for patient related morbidity and mortality, especially the after hour transfers.

**Objectives:** The aim of the study was to analyze the hours of transfer delay and their effect on readmission rates in the ICU.

**Methods:** We conducted a retrospective study of patients transfer from our ICU to the wards over last one year (Jan-Dec'2015).Data collected from the ICU database by the secretarial staff during the study period and divided into following categories of transfer delays:

1. Less than 4 hrs
2. 4–8 hrs
3. 8–24 hrs
4. More than 24 hrs
5. After hour transfers (from 8 PM-8 AM)

**Results:** There were 3362 patients admitted to our ICU during the study period of which 2475 patients were shifted to the wards. The average delay in shifting was around 6.5 hours (2–10.5 hrs).Delayed transfer of more than 8 hrs was found in 64 % patients and the percentage of after-hours transfer was 43 % of the total transfers. There were 16 readmissions into the ICU within 48 hrs of shift out among patients transferred in after hours as against 3 in patients transferred during routine hours.

**Conclusions:** Prevalence of delayed discharge from ICU was significant, especially the after hour discharges, which has got an impact on readmission rate as well. Discharge delay should be considered as an important quality indicator for critically ill patients to decrease the morbidity and mortality in ICU patients. Further studies are warranted to identify factors associated with delayed discharge.

### A1131 Incidence of bone fractures after critical illness

#### A.-F. Rousseau^1^, L. Michel^2^, M. Bawin^2^, E. Cavalier^3^, J.-Y. Reginster^4^, P. Damas^1^, O. Bruyere^4^

##### ^1^University Hospital of Liège, General Intensive Care Unit and Burn Centre, Liège, Belgium; ^2^University of Liège, Liège, Belgium; ^3^University Hospital of Liège, Clinical Chemistry Department, Liège, Belgium; ^4^University of Liège, Department of Public Health, Epidemiology and Health Economics, Liège, Belgium

###### **Correspondence:** A.-F. Rousseau – University Hospital of Liège, General Intensive Care Unit and Burn Centre, Liège, Belgium

**Introduction:** Critical illness (CI) and stay in an intensive care unit (ICU) are known to induce physical and functional changes. Bone is often forgotten in survivors. Limited published data reported an altered bone metabolism in case of prolonged ICU stay [1] and a decreased in bone mineral density (BMD) in the year following ICU admission [2]. Clinical impact of these changes is still not well described.

**Objectives:** Our retrospective study aimed to assess incidence of any new bone fractures (BF) two years after a severe CI.

**Methods:** Patients admitted in our ICU during 2013 were screened. Adults >18 years (y) old with an ICU length of stay (LOS) >7 days (d) were included. Lost to follow-up were considered exclusion criteria. Patients who died in ICU or who died during the follow-up period (FUP) with an ICU LOS ≤ 7d were also excluded. Demographic data, medical history and ICU related data were analyzed. Basal fracture risk before CI was calculated using the FRAX tool (https://www.shef.ac.uk/FRAX). In January 2016, referent family doctors were contacted by phone to check out new BF occurred during the 2 years after ICU discharge. Data are expressed as median (min-max) or percentages. Unpaired data were compared using Mann-Whitney test (p < 0.05 = significant).

**Results:** From the 1446 patients admitted in 2013, 884 had an ICU LOS ≤ 7d, 3 were < 18y, 278 died in ICU or died after an ICU LOS ≤ 7d and 31 were lost to follow-up. We analyzed 178 patients who were alive in January 2016 and 72 patients who died outside ICU during the FUP after an ICU LOS >7d. Regarding alive patients (64 % males), admission was mainly related to cardiovascular, respiratory and neurological failure, or trauma. Age was 64 (18–91)y, simplified acute physiologic score (SAPS II) was 44.5 (6–85), ICU LOS was 15 (8–106)d. According to the FRAX tool, the 10-y probability of major osteoporotic BF (major FRAX risk) was 5.4 (1–32)%. Nine patients (4 men) developed BF in 7.5 (1–28) months after CI, equivalent to a 5 % risk of new BF 2y after CI. A context of fall at home was noted in every case. Age, ICU LOS and SAPS II of these patients were not statistically different from non-fractured patients. Their major FRAX risk was 16 (3–29)%, significantly higher than non-fractured patients (p = 0.0029). Finally, among the 72 dead patients, only one 62y man experienced hip fracture at the 17^th^ month after CI.

**Conclusions:** Present incidence of new BF in the 2y following severe CI with a prolonged ICU stay is similar to previously published data [3]. Patients who experienced new BF after CI had a higher FRAX risk than the non-fractured patients. Influence of CI or ICU stay on BF risk is thus questioned. However, to be relevant, our results need to be compared to a control population: this work is ongoing.

**References**

^1^ J Clin Endocrinol Metab 2003, 88:4623–4632,

^2^ Am J Respir Crit Care Med 2015, 10.1164/rccm.201508-1514OC,

^3^ Crit Care Med 2011, 39 :1295–1300

## CARDIOGENIC SHOCK/CARDIOVASCULAR SURGERY/PULMONARY ARTERIAL HYPERTENSION

### A1132 Reduced total psoas area is associated with prolonged ICU length of stay for patients with aortic disease

#### J.-C. Zhou

##### Sir Run Run Shaw Hospital, Intensive Care Medicine, Hanghzou, China

**Introduction:** Most risk scoring systems to assess perioperative risk of patients with aortic disease were somewhat too complicated and time-consuming. Recently, frailty status has been demonstrated to be a convenient but valuable tool to estimate the preoperative mortality risk and disposition for patients underwent various operations. However, the prediction value of sarcopenia on the outcomes in patients with aortic disease is not completely clear.

**Objectives:** To explore the predictive role of preoperative sarcopenia on the outcomes in patients with aortic disease.

**Methods:** All patients with new diagnosed aortic diseases admitted to our hospital and with accessible abdominal CT images from January 1, 2013 to December 31, 2014 were retrospectively reviewed. Normalized total cross-sectional area of bilateral psoas major muscles at the level of L3 on CT for height was used to define pre-operative sarcopenia. The association between sarcopenia and patients' outcomes were evaluated with logistics regression.

**Results:** A total of 158 patients with new diagnosed aortic disease and accessible CT images were included in the study. Of them, 23 patients were categorized as sarcopenia on admission. Sarcopenia patients were tend to be much older (63.1 ± 14.0 yrs *vs* 55.5 ± 15.1 yrs, P = 0.026) and lighter body weight (63.3 ± 9.7 kg *vs* 69.0 ± 11.5 kg, P = 0.025). The total psoas muscle area was significantly lower in sarcopenia group both for male and female patients compared to non-sarcopenia group (P < 0.001 and P = 0.003). Moreover, sarcopenia patients were more likely to have a longer ICU length of stay (median 99 hrs *vs* 67 hrs, P = 0.047). After adjustment for patient demographics, concomitant hypertension, aortic involvement extend and management strategy, sarcopenia on admission for patients with aortic disease was significantly associated with longer ICU length of stay (OR: 2.77, 95 % CI: 1.01-7.69, P = 0.049).

**Conclusions:** Lower psoas major muscle cross section area on the admission CT was associated longer ICU length of stay for patients with aortic disease.

**References**

1. Reisinger KW, van Vugt JL, Tegels JJ, et al. Functional compromise reflected by sarcopenia, frailty, and nutritional depletion predicts adverse postoperative outcome after colorectal cancer surgery. Ann Surg. 2015;261(2):345–52.

2. Du Y, Karvellas CJ, Baracos V, Williams DC, Khadaroo RG. Sarcopenia is a predictor of outcomes in very elderly patients undergoing emergency surgery. Surgery. 2014;156(3):521–7.

3. Weijs PJ, Looijaard WG, Dekker IM, Stapel SN, Girbes AR, Oudemans-van Straaten HM, Beishuizen A. Low skeletal muscle area is a risk factor for mortality in mechanically ventilated critically ill patients. Crit Care. 2014;18(1):R12.

### A1133 Acetate- versus lactate-based balanced colloids used as priming solutions for cardiopulmonary bypass: an experimental pilot study

#### H. Cauwenberghs^1^, A. De Backer^1^, H. Neels^2^, I. Deblier^3^, J. Berghmans^1^, D. Himpe^1^

##### ^1^ZNA Middelheim General Hospital, Department of Anaesthesiology, Antwerp, Belgium; ^2^University of Antwerp, Department of Pharmaceutic Sciences, Antwerp, Belgium; ^3^ZNA Middelheim General Hospital, Department of Cardiac Surgery, Antwerp, Belgium

###### **Correspondence:** H. Cauwenberghs – ZNA Middelheim General Hospital, Department of Anaesthesiology, Antwerp, Belgium

**Introduction:** Using Stewart's approach it was definitely demonstrated that acid-base problems with cardiopulmonary bypass (CPB) are mainly due to infusing large fluid volumes, which can be resolved by administering balanced fluids with a high Strong Ion Difference (SID) [1]. Such balanced fluids contain electrolytes and organic ions as lactate or acetate metabolized to bicarbonate without influences on blood pH, even after several liters [2]. However, lactate might have some disadvantages. Therefore, this randomized, prospective study was conducted to compare acetate and lactate in a balanced gelatin-based colloidal prime.

**Objectives:** Classic and Stewart's approach acid-base changes during CPB till the end of surgery were primary end-points, such as the Strong Ion Gap (SIG) = apparent SID-effective SID. Secondary variables included glucose levels, oncotic pressure, diuresis, osmolarity and oxygen uptake.

**Methods:** Following IRB approval, 20 male non-diabetics gave consent and were randomly assigned to receive either succinylatedgelatin 30 g (Geloplasma®) in 150 mEq Na+, 5 mEq K+, 100 mEq Cl-, 3 mEq Mg++, 30 mEq lactate (SID 58) or succinylated gelatin 30 g (Isogelo®) 151 mEq Na+, 4 mEq K+, 2 mEq Ca, 103 mEq Cl-, 2 mEq Mg++, 30 mEq acetate (SID 56). With a power of 80 %, 10 subjects in each arm were sufficient to detect SIG differences over 2 mEq. Statistics included the Friedman test for changes over time, followed by Mann-Whitney-U tests to compare both groups testing the hypothesis whether or not lactate and acetate affected variables differently at each time point. Significance was fixed at p < 0,05 and Bonferroni corrected.

**Fig. 133 (abstract A1133).**
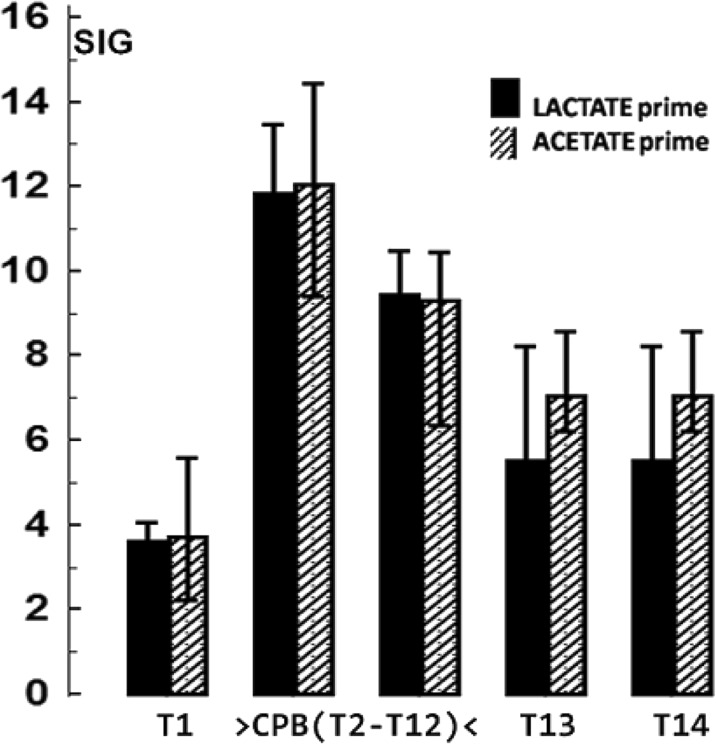
Strong Ion Gap (SIG) over time expressed in mEg/L as median and 95%CI. Black and hatched bars represent respectively group I (lactate) and group II (acetate). No significant differences were found

**Results:** Demographics were comparable. Acid-base variables changed similarly throughout without significant differences between groups (SIG shown in Figure 133).

**Fig. 134 (abstract A1133).**
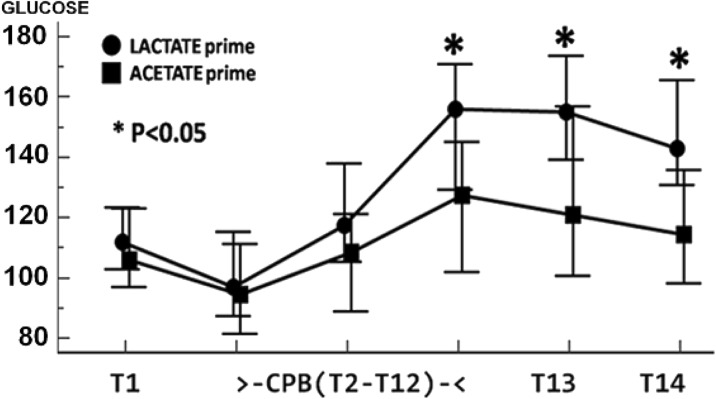
Glucode levels over time expressed in mg/dL as median and 95%CI. Closed circles and squares represent respectively group I (lactate) and group II (acetate). At the end of CPB and during the post-pump period, glucose was significantly more elevated in group I (*p < 0.05)

By contrast, glucose levels rose very significantly in the lactate group and persisted post CPB (Figure 134). Oncotic pressure, diuresis, osmolarity and oxygen uptake did not differ between groups.

**Discussion.** Concerning acid-base variables and secondary end-points, both of the solutions provided consistency and none of them seemed to be inferior, either. However, the significant hyperglycaemia observed in the lactate group is problematic and limits its further use as a CPB prime.

**References**

1. Elbers PW. The perioperative period. In: Kellum JA, Elbers PW (Eds.) Stewart's Textbook of AcidBase. AcidBase.org, Amsterdam, The Netherlands, 2009, 463–78.

2. Langer T, Ferrari M, Zazzeron L, Gattinoni L et Caironi PL Effects of intravenous solutions on acid-base equilibrium: from crystalloids to colloids and blood components. Anaesthesiology Intensive Therapy, 2014, 46:350–360.

**Grant acknowledgment**

All authors have no conflict of interest to declare

### A1134 Seric pro-adrenomedullin levels in low cardiac output syndrome (LCOS) after cardiac surgery

#### J.A. Barea-Mendoza^1^, I. Prieto Portillo^1^, M. Valiente Fernández^1^, R. Garcia Gigorro^1^, J.L. Perez Vela^1^, H. Marín Mateos^1^, S. Chacón Alves^1^, G. Morales Varas^1^, A. Rodriguez-Biendicho^1^, E. Renes Carreño^1^, J.C. Montejo González^1^

##### ^1^Hospital 12 de Octubre, Cardiac Intensive Care Unit, Critical Care Department, Madrid, Spain

###### **Correspondence:** J.A. Barea-Mendoza – Hospital 12 de Octubre, Cardiac Intensive Care Unit, Critical Care Department, Madrid, Spain

**Introduction:** After cardiac surgery, Low Cardiac Output Syndrome (LCOS) has an incidence between 3-45 %. LCOS increases ICU stay, mortality and thus costs. However, the risk assessment of patients undergoing cardiac surgery remains an unanswered question. Pro-Adrenomedullin (AM) is a vasoactive peptide isolated in pheochromocytoma. Recently, AM has been associated with outcomes in cardiac ischemia and diastolic or systolic dysfunction. Several publications have suggested that AM could be a good predictor for LCOS and mortality after cardiac surgery.(1)⁠⁠

**Objectives:** The aim of this study was to investigate the role of Pro-Adrenomedullin levels in prediction of LCOS after cardiac surgery.

**Methods:** Prospective single center study was developed according with the previous protocol. All patients from elective cardiac surgery were eligible from May to December 2015. We excluded emergency surgery. All patients signed the informed consent. We also collected relevant clinical information. LCOS was defined according current guidelines (2,3)⁠. We measured AM at 4 time-points (T1-T4): before surgery; at admission; 6 h and 18 h after surgery. Continuous data were showed as average (SD) and categorical ones in percents. Comparisons were performed with Kruskall-Wallis and ANOVA tests. The ROC approach was used to assess the predictor capacity of AM. All analyses were performed with STATA12. The Ethical Committee approved the study.

**Results:** 68 patients were included. The average of age was 65 ± 13.9 years, and were women 62.3 %. The median (IQR) for Euroscore was 5 (2–7). Comorbidities were hypertension (59 %), diabetes mellitus (26 %) and atrial fibrillation (18.8 %). On-pump surgery was performed in the 88 % and the coronary bypass was the most frequent (31 %). The incidence of Low Cardiac Output Syndrome was 23 %. AM levels (mmols/l) were: 0.82 ± 0.46 (before surgery); 1.07 ± 0.67 (at admission); 1.22 ± 1.03 (6 h) and 1.48 ± 1.3 (18 h), p < 0.05.

The AUC for ROC analyses was 0.73 (0.61-0.86 IC 95 %) for LCOS prediction. With using the most accurate cutoffs by the ROC analysis (1.48 mmols/l) values for sensibility and specificity were 100 % and 69 %. Patients with levels above the cutoff showed prolonged ICU stay (4 vs 2 days) and also hospital stay (13 vs 10); p < 0.05.

**Conclusions:** We detected a rise in Pro-Adrenomedullin levels after cardiac surgery. The results suggest that AM could be useful for LCOS prediction. More data are necessary to confirm the role in the prediction of relevant outcomes.

**References**

1. Schoe A et al. Postoperative Pro-Adrenomedullin Levels Predict Mortality in Thoracic Surgery Patients. Crit Care Med. 2015;43(2):373–81.

2. Pérez Vela JL et al. Med Intensiva. SEGO; 2012 May;36(4):e1-44.

3. Cecconi M al. Consensus on circulatory shock and hemodynamic monitoring. Task force of the European Society of Intensive Care Medicine. Intensive Care Med . 2014 Nov 13;1795–815.

**Fig. 135 (abstract A1134).**
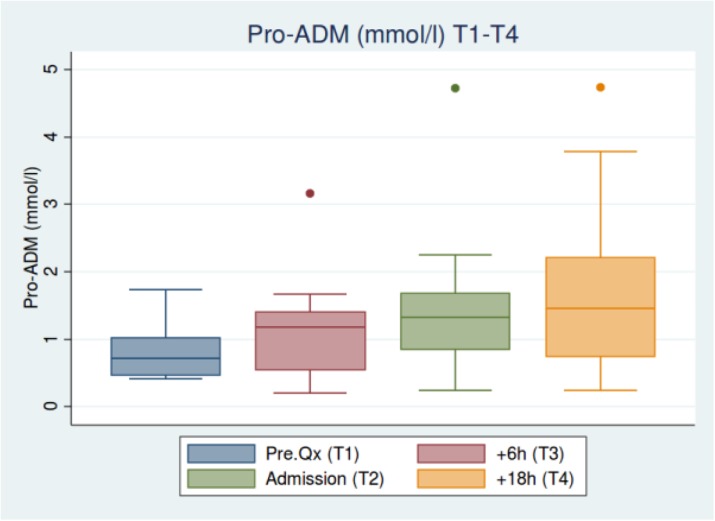
Pro-ADM levels T1-T4

### A1135 How to improve quality of life in patients with pulmonary arterial hypertension via multidisciplinary care model

#### J.-S. Yang^1^, C.-H. Chiang^2^, W.-T. Hung^3^, W.-C. Huang^3^, C.-C. Cheng^2^, K.-C. Lin^2^, S.-C. Lin^3^, K.-R. Chiou^2^, S.-R. Wann^3^, K.-L. Lin^3^, P.-L. Kang^2^, G.-Y. Mar^2^, C.-P. Liu^2^

##### ^1^Fooyin University, Department of Physical Therapy, Kaohsiung City, Taiwan, Province of China; ^2^Kaohsiung Veterans General Hospital, Cardiovascular Division, Kaohsiung City, Taiwan, Province of China; ^3^Kaohsiung Veterans General Hospital, Critical Care Division, Kaohsiung City, Taiwan, Province of China

###### **Correspondence:** C.-H. Chiang – Kaohsiung Veterans General Hospital, Cardiovascular Division, Kaohsiung City, Taiwan, Province of China

**Objectives:** Pulmonary arterial hypertension (PAH) is associated with reductions in health-related quality of life (HRQL). The patient care still played an important role in improvement of HRQL, even though more drug therapy was identified in recent decade.. In this study, we investigated to provide quality care for patients with pulmonary arterial hypertension via multidisciplinary care model.

**Methods:** A multidisciplinary team was organized.in a tertiary medical center, including intensivists, cardiologists, pulmonologists, cardiac surgeons, rheumatologists, chest surgeons, rehabilitation physicians, psychologist, pharmacologists, hospice care physicians, nutritionist, social workers and nursing staffs. The key interventions include home based rehabilitation therapy, 24 hours hot line care, PAH care nurse training program, hospice care information and consultation, phychological care and autogenic training, prompt PAH referral system, social care connections, on-line self PAH risk assessment system, on-line and innovative mobile apps patient instructions, facebook patient care group and ourdoor PAH patient education program. The PAH patients were divided into three groups: pre-interventional group from May to Dec 2013, Interventional group from Jan to June 2014 and post-interventional group from July 2014 to Feb 2016. HRQL was measured using the *Short Form 36* Health Survey (SF-36) in all enrolled subjects.

**Results:** The average physical compartment scale of SF-36, including physical functioning; role limitations due to physical health, pain and general health improved from 49 ± 30 in pre-interventional group, to 52 ± 34 in interventional group and to 72 ± 22 in post-interventional group (p < 0.05). The average mental compartment scale of SF-36 , including role limitations due to emotional problems, energy/fatigue, emotional well-being and social functioning, improved from 54 ± 30 in pre-interventional group, to 56 ± 31 in interventional group and to 71 ± 17 in post-interventional group (p < 0.05).

**Conclusions:** The study demonstrated multidisciplinary care model could improve HRQL of patients with pulmonary arterial hypertension.

### A1136 Blood pressure management with urapidil for patients with aortic dissection is associated with less esmolol usage than nicardipine

#### J.-C. Zhou

##### Sir Run Run Shaw Hospital, Intensive Care Medicine, Hanghzou, China

**Introduction:** Acute aortic disease is a common but challenging entity in clinical practice. Titration the blood pressure and heart rate to a target level is of paramount importance in the acute phase regardless of whether the patient will undergo a surgery or not eventually. In addition to the initially intravenous β-blockers, parenteral infusion of nicardipine and urapidil are the most common used antihypertensive therapy currently in mainland China. However, few empirical data was available with respect to the different effect on patients' outcome of the two antihypertensive strategies, especially given the deleterious reflex tachycardia of vasodilators which may increase force of ventricular contraction and potentially worsen aortic disease.

**Objectives:** To evaluate the difference of the abovementioned two antihypertensive strategies on the outcome of patients with aortic disease.

**Methods:** All patients with new diagnosed aortic diseases presented to our hospitals from January 1, 2013 to June 30, 2014 were retrospectively reviewed. The antihypertensive strategies and their association with patients' outcomes were evaluated with logistics regression.

**Results:** A total of 120 patients with new diagnosed aortic disease were included in the study. Of them, 47 patients received urapidil while 73 patients received nicardipine antihypertensive therapy. Patients with nicardipine were more quickly to reach the target blood pressure level than those treated with urapidil (median 18 *vs* 35 mins, P = 0.024). After adjustment for patient demographics, co-morbidity, involved extend of aorta, interventional strategies, antihypertensive therapy with nicardipine (with urapidil as reference) for patients with aortic disease was significantly associated with high esmolol cost (OR: 6.2, 95%CI: 1.8-21.6, P = 0.004) and longer ICU length of stay (OR: 3.9, 95%CI: 1.5-10.3, P = 0.006). However, there was no significant correlation between nicardipine use and ICU mortality (OR: 0.3; 95%CI, 0.1-1.4, P = 0.123).

**Conclusions:** Although nicardipine achieved the target blood pressure level more quickly than urapidil for patients with aortic disease, it was associated with more esmolol use and longer ICU length of stay.

**References**

1. Boodhwani M, Andelfinger G, Leipsic J, et al. Canadian Cardiovascular Society position statement on the management of thoracic aortic disease. Can J Cardiol 2014;30(6):577–89.

2. Hiratzka LF, Bakris GL, Beckman JA, et al. 2010 ACCF/AHA/AATS/ACR/ASA/SCA/SCAI/SIR/STS/SVM guidelines for the diagnosis and management of patients with Thoracic Aortic Disease. Circulation 2010;121:e266-369.

3. Pagni S, Ganzel BL, Trivedi JR, et al. Early and midterm outcomes following surgery for acute type A aortic dissection. J Card Surg 2013;28:543–9.

4. Melby SJ, Zierer A, Damiano RJ Jr, et al. Importance of blood pressure control after repair of acute type a aortic dissection. J Clin Hypertens (Greenwich) 2013;15:63–8.

### A1137 Postoperative excessive blood loss after cardiac surgery can be predicted with international society on thrombosis and hemostasis (ISTH) scoring system

#### Y.J. Choi^1^, S.Z. Yoon^2^

##### ^1^Pusan National University Yangsan Hospital, Anesthesia and Pain Medicine, Yangsan-si, Republic of Korea; ^2^Korea University Anam Hospital, Anaesthesiology and Pain Medicine, Seoul, Republic of Korea

###### **Correspondence:** Y.J. Choi – Pusan National University Yangsan Hospital, Anesthesia and Pain Medicine, Yangsan-si, Republic of Korea

**Introduction:** Postoperative bleeding is one of the most common complications of cardiac surgery. Excessive perioperative bleeding continues to complicate cardiac surgery with cardio-pulmonary bypass (CPB) in spite of improvements in extracorporeal oxygenation and surgical techniques. Even bleeding after cardiac surgery has variable causes, we thought the applying ISTH scoring system may be able to predict the postoperative excessive blood loss in patients after cardiac surgery with CPB.

**Objectives:** The aim of present study was to examine the effectiveness of International Society on Thrombosis and Hemostasis (ISTH) scoring system in patients with cardiac surgery.

**Methods:** The medical records of patients undergoing elective cardiac surgery using CPB between Mar. 2010 and Feb. 2014 were retrospectively reviewed. These demographic and clinical characteristics, perioperative laboratory findings, and postoperative complications were assessed using computerized databases from our institution. ISTH score was calculated in ICU and Patients were divided with overt DIC group and non-overt DIC group.

**Results:** Among 384 patients with cardiac surgery, 70 patients with Overt DIC Group (n = 20) or Non-overt DIC Group (n = 50) were enrolled. Mean DIC scores at ICU admission was 5.35 ± 0.59 (Overt DIC Group) and 2.66 ± 1.29 (Non-overt DIC Group) and overt DIC was induced in 29 % (20/70). Overt DIC group had much more EBL for 24 hrs (*P* = 0.006) and maintained longer time of intubation time (*P* = 0.005)

**Conclusions:** In spite of limitation of retrospective design, management using ISTH score in patients after cardiac surgery seems to be helpful for prediction of the post-CPB excessive blood loss and prolonged tracheal intubation duration.

**References**

1. Woodman RC, Harker LA - Bleeding complications associated with cardiopulmonary bypass. Blood. 1990;76:1680–1697.

2. Bakhtiari K, Meijers JC, de Jonge E, Levi M - Prospective validation of the International Society of Thrombosis and Haemostasis scoring system for disseminated intravascular coagulation. Critical care medicine. 2004;32:2416–2421.

3. Taylor FB, Jr., Toh CH, Hoots WK, Wada H, Levi M - Towards definition, clinical and laboratory criteria, and a scoring system for disseminated intravascular coagulation. Thrombosis and haemostasis. 2001;86:1327–1330.

**Grant acknolwedgment**

This study was supported by a Korea University Research Grant.

### A1138 Preoperative use of statin and event of atrial fibrillation in cardiac surgery. Analysis with propensity score

#### A. Gordillo-Brenes^1^, M.D. Fernandez-Zamora^2^, L. Perez-Borrero^3^, M.D. Arias-Verdu^4^, E. Aguilar-Alonso^5^, A. Herruzo-Aviles^6^, M. Garcia-Delgado^7^, R. Hinojosa-Perez^6^, E. Curiel-Balsera^2^, R. Rivera-Fernandez^3^, ARIAM-ANDALUCIA

##### ^1^Hospital Puerta del Mar, Cadiz, Spain; ^2^Hospital Regional, Intensive Care, Malaga, Spain; ^3^Hospital Serrania, Ronda, Spain; ^4^Hospital Regional, Intensive Care, Málaga, Spain; ^5^Hospital Infanta Margarita, Intensive Care, Cabra, Spain; ^6^Hospital Virgen del Rocio, Intensive Care, Sevilla, Spain; ^7^Hospital Virgen de las Nieves, Intensive Care, Granada, Spain

###### **Correspondence:** M.D. Arias-Verdu – Hospital Regional, Intensive Care, Málaga, Spain

**Introduction:** Statin therapy has shown conflicting data in the prevention of the postoperative atrial fibrilation (AF) in cardiac surgery.

**Objective:** To analyze the relationship between preoperative use of statin and development of AF in postoperative cardiac surgery patients according to the type of surgery performed.

**Methods:** A prospective multicenter study in 11 hospitals, between 2008 to 2012, included in Spanish ARIAM database. The patients with previous use of statins were paired with those who do not, according to a propensity score that quantify the probability of being treated with statins preoperatively based on demographics dates, comorbidities, medication and preoperative diagnosis according surgery type (valvular or coronary bypass). We analyze differences in the postoperative incidence of AF in both groups.

**Results:** Cohort of 7276 patients, Mean age 63.91 ± 12.45 years. 85.9 % was elective surgery. EuroSCORE 5.86 ± 3.14 points. ICU mortality was 7.6 %.Prior to surgery, 51.5 % and 25.8 % taking statins present a history of AF. 21.9 % postoperative AF episode presented.

The patients treated before surgery with statins had AF 19.8 % vs 22.5 % (P =0.006). We pair a group of 1528 patients with statins with other similar group that do not take, based on an propensity score . The frequency of AF was 20.4 % in treated and 24.9 % in untreated (p = 0.003), OR: 0.772 (0.652-0.916). In coronary bypass surgery (n = 1649), AF occurs in 17.2 % of 331 patients that not taking statins and 14 % of 1318 patients with previous statins treatment (p =0.141). We calculated a specific propensity score and matching was performed; 276 patients without statins occurred AF in 17.4 % and other 276 patients with statins in 13.8 % (p = 0.241), OR: 0.76 (0.48-1.21).

In valvular surgery (n = 6245), AF occurs in 24.4 % of 2475 patients with statins and 22.9 % of 1790 without statins (p = 0.256). After propensity score calculation, matching is performed; in 842 patients with statins , the AF appears in 27.7 % and in 842 patients without statins 22.8 % (p = 0.021), OR: 0.772 (0.62 -0.96).

**Conclusions:** Preoperative statin use was associated with a lower risk of atrial fibrillation after cardiac surgery, observed in valve surgery (significant differences) and bypass (although significant differences by possible under sample number).

### A1139 Predictors of mortality after cardiac surgery in elderly patients in the intensive care unit in a referral hospital

#### S.P. Gómez Lesmes, L.E. De la Cruz Rosario, A. Ansotegui Hernández, A.N. García Herrera, E. Regidor Sanz, M.J. Gómez Sánchez, J. Barado Hualde, O. Agudo Pascual, J.P. Tirapu León, J.M. Guergue Irazabal

##### Complejo Hospitalario de Navarra, Intensive Care Medicine, Pamplona, Spain

###### **Correspondence:** S.P. Gómez Lesmes – Complejo Hospitalario de Navarra, Intensive Care Medicine, Pamplona, Spain

**Introduction:** Although satisfactory results have been achieved in the literature, higher mortality have been reported in elderly patients after cardiac surgery.

**Objectives:** Knowing the prognostic factors associated with mortality in over 75 years undergoing cardiac surgery.

**Methods:** We conducted a observational and retrospective study of older patients than 75 years who underwent cardiac surgery in a referral hospital from July 2009 to June 2015. The sample was divided among the patients who died and those who do not. Demographic variables, prognostic scales,type of surgery,early complications, ICU stay and mortality were compared.The variables that reached statistical significance in the univariate analysis were analyzed in multivariate logistic regression. Data expressed as mean and standard deviation, percentage, mean difference, odds ratio and corresponding confidence intervals. Statistical significance level of *P* < 0.05.

**Results:** A total of 1656 patients were analyzed, ≥ 75 years 527(31.8 %) of which 32 died(6.1 %). The mean age was 78.9 years (SD 0.7), 59.4 % are women. Both groups differed in both the APACHE II, being 18.6 (SD 7.2) vs 13.2 (14.3), *P* < 0.001 and the average stay in the ICU, being 20.5 days (SD 31.2) vs 5.83 days (11.3), *P* = 0.014. There were differences in the preoperative variables: COPD 18.8 % vs 6.5 %, *P* = 0.009; Peripheral artery disease 18.8 % vs 8.1 %, *P* = 0.038; average logistic EuroSCORE 24.1 (22.5) vs 10.8 (8.2), *P* = 0.002; LVEF 56.9 % (SD 15.9) vs 57 % (SD 13.5).Type of surgery: valve 59.4 % (being the most frequent aortic valve) vs 66.1 %; coronary 6.3 % vs 16.6 %, mixed 18.8 % vs 13.9 %, without significant differences. They presented differences in postoperative complications: cardiac arrest 31.3 % vs 0.6 %, *P* ≤ 0,001; reoperation 31.3 % vs 6.7 %, *P* ≤ 0,001; cardiogenic shock 47 % vs 4 %, *P* ≤ 0,001; postoperative hemorrhage 43.8 % vs 10.7 %, *P* ≤ 0,001; Atrial fibrillation 34.4 % vs 17.8 %, *P* = 0.02; encephalopathy 46.9 % vs 8.1 %, *P* ≤ 0.001; acute renal failure, 65.6 % vs 20 %, p ≤ 0,001; respiratory failure, 40.6 % vs 13.5 %, *P* ≤ 0,001; Mechanical > 48 h 67.7 % vs 13.7 %, *P* ≤ 0.001 ventilation. The variables that reached statistical significance in the multivariate analysis as predictors of mortality were APACHE II OR 1.13 (95 % CI 1.1-1.2), *P* = 0.001, Euroscore OR 1.4 (95 % CI 1,2- 1.6), *P* < 0.001; acute respiratory failure OR 3.4 (95 % CI 1.4 to 8.2), *P* = 0.008; acute renal failure OR 3.1 (95 % CI 1.3-7.5), postoperative bleeding OR 4.9 (95 % CI 2.1 to 11.9), *P* < 0.001

**Conclusions:** Mortality in this group is similar to other series, being patients with more comorbidities, with the highest score in the EuroSCORE and APACHE II and more often subjected to mixed surgery. The Euroscore, APACHE II, respiratory failure, renal failure and postoperative bleeding, predict higher mortality.

**References**

Schurr P, et al. Predictors of postoperative complications in octogenarians undergoing cardiac surgery. ThoracCardiovasc Surg. 2010Jun;58(4):200–3

**Grant acknolwedgment**

none.

### A1140 Ventricular assist devices, transfusion and health-related quality of life

#### A. González Pérez^1^, P. Alvarez Fernández^2^, L. Lopéz Amor^3^, G. Muñiz Albaiceta^1^

##### ^1^Hospital Universitario Central de Asturias, ICU 1, Oviedo, Spain; ^2^Hospital Universitario Central de Asturias, ICU 3, Oviedo, Spain; ^3^Hospital Universitario Central de Asturias, Oviedo, Spain

###### **Correspondence:** A. González Pérez – Hospital Universitario Central de Asturias, ICU 1, Oviedo, Spain

**Introduction:** Recent studies have established increasing blood product volume as an independent predictor of increased postoperative mortality following ventricular assist devices (VAD) but it is unknown how it may affect long-term health-related quality of life. Patients in cardiogenic shock who require VAD are patients who receive multiple transfusions.

**Objectives:** The aim of this study is to assess the influences between multiple transfusions of and long term Health Related quality of life (HRQOL) after cardiogenic shock that required VAD.

**Methods:** Prospective observational study of patients in cardiogenic shock who required VAD in our institution from January 2008 to June 2014. Demographic and clinical variables of the computerized database of the Hospital are extracted together with the blood products that were transfused during the Hospital stay. Survivors HRQOL were analyzed by EUROQOL 5D in February 2016 by telephone interview. A linear regression model was used to calculate regression coefficients. We used the Visual Analogue Scale (VAS) of EUROQUOL 5D to evaluate the relationship between these values and the number of Packed Red Blood Cell or total blood products (TBP) transfused. Values expressed in mean ± SD. Significance level of p < 0.05. CI 95 %.

**Results:** A total 44 patients were included. 54 % males, age 50,45 SD 11.08 years, BMI 26 SD 3.68, mean LVEF pre procedure 21.07 % SD 10.37 %, ischemic etiology 36.4 %,median RBC transfused 21.5 (3–86), median TBP transfused 37 (4–126); VAD ( Abiomed BVS500 or Levitronix) n = 20; ECMO n = 24. The ECMO patients were transfused more fresh frozen plasma units (FFP) p < 0.05. Twenty-six patients died (59 %) and these were transfused least RBC p < 0.05. 13 patients of total survivors (n = 18) answered the questionnaire EUROQOL 5D.The most affected HRQOL dimensions were pain/discomfort and anxiety/depression. The regression coefficient was used to study the effect of multi transfusion of RBC and TBP on HRQOL (VAS). The observed effect was (−1.27, 95 % CI −1.67 to −0.91, p < 0.01) and (−0.51, 95 % CI −0.76 to −0.27, p < 0.01) respectively.

**Conclusions:** The multiple transfusions of RBC and TBP could have a negative effect on long-term HRQOL in patients in cardiogenic shock requiring cardio circulatory support.

**References**

See comment in PubMed Commons below

1. **Ventricular assist devices and increased blood product utilization for cardiac transplantation.** Stone ML et all. J Card Surg. 2015 Feb;30(2):194–200.

2. **Bleeding complications and blood product utilization with left ventricular assist device implantation.** Schaffer JM et all. Ann Thorac Surg. 2011 Mar;91(3):740–7;

### A1141 Current situation of postoperative cardiac surgery in octogenarian population in the intensive care unit in a tertiary hospital

#### S.P. Gómez Lesmes, L.E. De la Cruz Rosario, A. Ansotegui Hernández, E. Regidor Sanz, M.J. Gómez Sánchez, S. Aldunate Calvo, A.N. García Herrera, J. Barado Hualde, O. Agudo Pascual, J.P. Tirapu León

##### Complejo Hospitalario de Navarra, Intensive Care Medicine, Pamplona, Spain

###### **Correspondence:** S.P. Gómez Lesmes – Complejo Hospitalario de Navarra, Intensive Care Medicine, Pamplona, Spain

**Introduction:** The increased life expectancy has made the heart surgery is performed increasingly common in elderly patients who often have multiple comorbidities, which can lead to complications in the postoperative period.

**Objectives:** to analyze the experience of a referral cardiac surgery center with all types of cardiac surgery interventions performed in patients ≥80 years old over a six years' period

**Methods:** We conducted a retrospective observational study of patients undergoing cardiac surgery in a referral hospital for a population of 650,000 inhabitants, between July 2009 and June 2015, after completion of cardiac surgery. The patients were divided into two groups: aged < 80 and age ≥80 years. Association analysis of demographic, clinical, therapeutic factors and complications during ICU stay. Univariate analysis using Chi square (Fisher if applicable) and T Student. Data expressed as percentages, means, estándar desviation (SD), mean differences (dm), odds ratio (OR), and confidence intervals 95 % (CI95%)

**Results:** A total of 1656 patients admitted to the unit of which 183 were older than 80 years (11.05 %, mean age 81.9 years (SD = 1.74) vs 65.8 years (SD = 10.7 ), *P* < 0.001, with 64.6 % men and 51.4 % women, *P* ≤ 0,001. Type of surgery performed in octogenarians: valvular (74.9 %), coronary and valvular (13.7 %), coronary (8.2 %), thoracic aorta (1.6 %). Surgical ischemia time 82,84 min (SD = 31.07) vs 94 min (SD = 41.9), dm 11.26 min (95 % CI 6.2 to 16.3) *P* < 0.001, perfusion time 122,6 min (SD = 41.7) vs 138.5 min (SD = 54.4), dm 15,9 min (95 % CI 9.1 to 22.6), *P* < 0.001. Apache II 13.35 (SD 4.79) vs 11.09 (SD = 5.52), dm 6.17 (95 % CI 1.4 to 3.1) *P* < 0.001. EUROSCORE probability 13.30 (SD = 10.30) vs 7.13 (SD = 8.3), dm 6.2 (95 % CI 4.6 to 7.3), *P* < 0.001; no major differences in preoperative ejection fraction (EF), COPD or pulmonary hypertension. Acute renal failure 23 % vs 13.6 %, OR 1.9 (95 % CI = 1.3 to 2.7), *P* = 0.001. No significant differences in cardiac arrest, tamponade, re intervention, arrhythmias, respiratory distress, days of mechanical ventilation, neurological impairment or MODS. Intra-ICU mortality 7.7 % vs 3.3 %, OR 2.5 (95 % CI 1.3 to 4.6), *P* = 0.006. The days of ICU stay 5.6 days (SD = 10) vs 5.7 (SD = 12.7), dm 0.2 (95 % CI −3.0 to 3.5).

**Conclusions:** The ICU stay and early complications evaluated not differ between the two groups , except for acute renal failure and higher mortality, despite the use of shorter times in cardiac surgical in octogenarians. There is a progressive decrease in coronary artery bypass surgery in recent years in this group probably in favor of percutaneous techniques.High-risk patients who require intensive perioperative management,should be identified to reduce the incidence of postoperative complications.

**References**

1. Scandroglio AM, Finco G, Pieri M, et al. Cardiac surgery in 260 octogenarians: a case series. BMC Anesthesiol. 2015 Jan 26;15:15

**Grant acknolwedgment**

The study was supported by departmental funds only

### A1142 A bedside predictive model of mortality in the octogenarian and over octogenarian undergoing heart surgery

#### A. Corona, C. Ruffini, A. Spazzadeschi, F. Marrazzo, A. Gandola, R. Sciurti, C. Savi, E. Catena

##### ASST Sacco Fatebenefratelli, Milano, Italy

###### **Correspondence:** A. Corona – ASST Sacco Fatebenefratelli, Milano, Italy

**Introduction:** Up to date octogenarians and over-octogenarians represents 6-10 % of patients undergoing heart surgery (HS) (1). The 30^th^ day mortality seems to be higher (3,1-21 %, vs. 1,6-2,2 p < 0.001) if compared with younger patients, even if not associated with longer ICU stay. (2,3).

**Objectives:** Retrospective observational cohort study to asses predictors of 30^th^ day mortality.

**Methods:** We considered all the patients undergoing any type of HS since January 1994 through 2015 at Luigi Sacco Hospital, Milano. Patients were divided into two group in relation if their age was ≥ 85 or < 85 yrs. End point variable is 30^th^ day mortality. On all the patients we collected the following variables: (i) demographics, chronic diseases and type of cardiac diseases; (ii) risk score (i.e. NYHA, Euro-Score) and left ventricular ejection fraction; (iii) intra- and post-operative variables (i.e. organ failure, vaso-active drugs, IABP, duration of mechanical ventilation). Stata 12.1 was used for statistical analysis.

**Results:** A total of respectively 889 (6.9 %) over 85 yrs. and 11966 < 85 yrs patients were found. The 30^th^ day overall mortality was 4.3 % higher in the over 85 yrs. Same difference was confirmed after stratifying patients according type of HS, particularly in case of combined and aorta surgery. A Cox´s proportional model found the following variables (out the 32 considered) as predictors of 30^th^ day death: (i) pre-operative chronic renal failure [HR: 1.826, (95 % CI: 1.141- 2.933) p = 0.012]; (ii) vasculopathy [HR: 2.088, (95%CI: 1.396 - 3.125) p < 0.0001]; (iii) aorta disease [HR: 2.512, (95 % CI: 1.489 - 4.238) p = 0.001)]; (iv) pre-operative cardiogenic shock [HR: 7.654, (95 % CI: 2.327 - 25.17) p = 0.001]; (v) post-operative septic shock [HR: 5.410 (95 % CI: 2.459 - 11.90) p < 0.0001]. Surprisingly age **≥**85 [HR 1.576, (95 % CI: 0.843 - 2.915) p = 0.156] was not confirmed as a variable significantly affecting the mortality. The age interaction with the predictors was tested without finding any statistical significance or model modifications.

**Conclusions:** Despite its limitations, our study suggests that HS might be an option for octogenarian and over since an age ≥ 85 yrs. does not seem to impact on post-operative mortality.

**References**

1. Bhamidipati CM, LaPar DJ, Fonner E Jr, Kern JA, Kron IL, Ailawadi G. Outcomes and cost of cardiac surgery in octogenarians is related to type of operation: a multiinstitutional analysis. Ann Thorac Surg. 2011 Feb;91(2):499–505.

2. Abel NJ, Rogal GJ, Burns P, Saunders CR, Chamberlain RS. Aortic valve replacement with and without coronary artery bypass graft surgery in octogenarians: is it safe and feasible? Cardiology. 2013;124(3):163–73.

3. Sen B, Niemann B, Roth P, Aser R, Schönburg M, Böning A. Short- and long-term outcomes in octogenarians after coronary artery bypass surgery. Eur J Cardiothorac Surg. 2012 Nov;42(5):e102-7.

**Grant acknolwedgment**

None.

### A1143 Caffeic acid phenethyl ester reverses monocrotaline-induced PAH in rat via the inhibition of Hif-1a regulated PDGF signaling

#### M.-W. Ke^1^, C.-C. Cheng^2^, W.-C. Huang^1^, C.-H. Chiang^2^, W.-T. Hung^1^, K.-C. Lin^1^, S.-C. Lin^1^, S.-R. Wann^1^, K.-R. Chiou^2^, C.-J. Tseng^3^, P.-L. Kang^2^, G.-Y. Mar^2^, C.-P. Liu^2^

##### ^1^Kaohsiung Veterans General Hospital, Critical Care Division, Kaohsiung City, Taiwan, Province of China; ^2^Kaohsiung Veterans General Hospital, Cardiovascular Division, Kaohsiung City, Taiwan, Province of China; ^3^Kaohsiung Veterans General Hospital, Department of Medical Education and Research, Kaohsiung City, Taiwan, Province of China

###### **Correspondence:** C.-H. Chiang – Kaohsiung Veterans General Hospital, Cardiovascular Division, Kaohsiung City, Taiwan, Province of China

**Introduction:**Pulmonary arterial hypertension (PAH) is a disease with gradually increased pulmonary vascular resistance and pressure, often leads to right ventricular (RV) failure and death. Excessive proliferation of pulmonary arterial smooth muscle cells (PASMCs) is regarded as the major cause of the remodeling of pulmonary artery, whereas the underlying mechanism is largely unclear. Caffeic acid phenethyl ester (CAPE) is the main component of propolis, which is known as a versatile compound of antimitogenic, anticarcinogenic and anti-inflammatory potentials.

**Objectives:** To investigate the effects of CAPE on the improvement of the hemodynamic function in PAH animal model and to explore the underlying mechanisms in *in vitro* PASMCs.

**Methods:** Animal model of PAH symptom was induced in 200–250 grams Sprague-Dawley rats by subcutaneous injection of monocrotaline (MCT, 60 mg/kg). 2 weeks later, the MCT-induced PAH rats received intraperitoneal administration of CAPE with various dosages of 5 or 10 mg/kg once per day, for further 2 weeks. Hemodynamic functions, including RV systolic pressure (RVSP) and Fulton index, were measured before sacrifice. The lung tissues were harvested for examining the vascular remodeling of pulmonary artery. To investigate the molecular mechanisms, *in vitro* cultured human PASMCs challenged with either 3 % oxygen level or recombinant human PDGF (40 ng/mL), followed by the treatment of CAPE in 5 or 10 mM. The change of expression level and phosphorylation of the cellular signaling molecules, including ERK, AKT, NF-kB, or Hif-1a, were analyzed by semi-quantitative PCR and western blotting, respectively.

**Results:** In MCT-induced PAH rats, CAPE significantly improved the hemodynamic values of RVSP, Fulton index, and attenuated the severity of pulmonary vascular remodeling. Furthermore, the administration of CAPE critically reduced the expression levels of Hif-1a, NF-kB and PDGF molecules in the lung of MCT-induced PAH rats. In vitro assay showed that an increased expression level of *hif-1α* and *pdgf* genes in hPASMCs was observed under hypoxia or PDGF stimulation, which was significantly suppressed following CAPE treatment. For chemical inhibition, we indicated that cellular signaling molecules ERK, AKT and NF-kB were involved in the up-regulation of *hif-1a* and *pdgf* genes, which were responsible for the proliferation of hPASMCs exposed to hypoxia or PDGF stimulation. In addition, CAPE also significantly promoted the number of apoptotic cells and the number of cell arrested in G0 phase of hPASMCs by TUNEL assay and SA-b-galactosidase staining, respectively.

**Conclusions:** We showed evidence that the natural compound CAPE could provide therapeutic benefits on the reversal of experimental PAH rats. Importantly, the results further indicated that the *hif-1a*-mediated *pdgf* expression is a positive feedback mechanism underlying the pathogenesis of PAH, which was regulated by the AKT/ERK/NF-kB signaling.

### A1144 Right ventricular arterial coupling after cardiac surgery: a preliminary report

#### P. Bertini^1^, F. De Sanctis^2^, F. Guarracino^1^

##### ^1^University Hospital of Pisa, Department of Anaesthesia and Critical Care Medicine, Cardiothoracic and Vascular Anaesthesia, Pisa, Italy; ^2^University of Pisa, Scuola di Specializzazione in Malattie del l'Apparato Cardiovascolare, Pisa, Italy

###### **Correspondence:** P. Bertini – University Hospital of Pisa, Department of Anaesthesia and Critical Care Medicine, Cardiothoracic and Vascular Anaesthesia, Pisa, Italy

**Introduction:** Right ventriculo-arterial coupling (rVAC), defined as the ratio of end-systolic elastance (rEes) to pulmonary arterial elastance (rEA) is considered a sensitive method to assess right heart performance [1].

**Objectives:** In this study we aim to identify the feasibility of measuring rVAC in hemodynamic deranged patients undergoing complex/emergency cardiac surgery using cardiopulmonary bypass (CPB) as an experimental model of further hemodynamic impairment.

**Methods:** We measured rEa as ratio of pulmonary pressure at the dicrotic notch (dyPAP) and stroke volume (SV) [2] and rEes as ratio of the difference between mean pulmonary artery pressure (mPAP) and wedge pressure (PCWP) and end systolic volume (rESV) (mPAP-PCWP/rESV) [3] after the induction of anaesthesia (T0) via pulmonary artery catheter (SwanGanz 774 F45 and Vigilance II Monitor by Edwards Lifesciences), after weaning from CPB (T1) and 4 h after in ICU(T2) in 4 patients.

**Results:** Measure of rVAC has been demonstrated feasible in all four patients undergoing cardiac surgery.

As expected all the patients were found uncoupled (rVAC > 1) before surgery, immediately after weaning from CPB rVAC worsen and in ICU it was restored to the basal.

**Conclusions:** In this preliminary analysis we demonstrated the feasibility of measuring rVAC in critical patients undergoing cardiac surgery, to our knowledge this is the first report in this field. As expected rVAC is very much influenced by CPB although further investigation is needed to confirm the utility of this technique to monitor the right heart in such patients.

**References**

1. Ryan JJ, Tedford RJ: Diagnosing and treating the failing right heart. *Current opinion in cardiology* 2015, 30(3):292–300.

2. Chemla D, Hebert JL, Coirault C, Salmeron S, Zamani K, Lecarpentier Y: Matching dicrotic notch and mean pulmonary artery pressures: implications for effective arterial elastance. *The American journal of physiology* 1996, 271(4 Pt 2):H1287-1295.

3. Sanz J, Garcia-Alvarez A, Fernandez-Friera L, Nair A, Mirelis JG, Sawit ST, Pinney S, Fuster V: **Right ventriculo-arterial coupling in pulmonary hypertension: a magnetic resonance study**. *Heart* 2012, **98**(3):238–243.

### A1145 Mixed venous to arterial carbon dioxide difference is an early marker of hypoperfusion during veno-arterial ECMO: a case report

#### P. Bertini, R. Baldassarri, F. Guarracino

##### University Hospital of Pisa, Department of Anaesthesia and Critical Care Medicine, Cardiothoracic and Vascular Anaesthesia, Pisa, Italy

###### **Correspondence:** P. Bertini – University Hospital of Pisa, Department of Anaesthesia and Critical Care Medicine, Cardiothoracic and Vascular Anaesthesia, Pisa, Italy

**Introduction:** Detection of tissue hypoperfusion is paramount in the management of VA ECMO. Arterial to pulmonary artery CO_2_ difference has been demonstrated to be an early marker of hypoperfusion in the shock patient [1] and during hypothermic cardiopulmonary bypass [2],

**Objectives:** In this report we investigated the accuracy and feasibility of mixed venous to arterial CO_2_ difference as an early marker of perfusion mismatch during VA ECMO.

**Methods:** In a patient treated with VA ECMO for refractory cardiac arrest due to acute myocarditis we performed serial measurements of pulmonary artery to arterial CO_2_ difference as well as SVO_2_, MAP, urine output and lactate level.

**Results:** During reduced perfusion periods, assessed by elevated lactacidemia (>2 mmol/l) we observed high > 7 CO_2_ difference which is concordant to literature [3]. During 15 episodes of reduced systemic perfusion, demonstrated by increase of serum lactic acid we were able to early detect hemodynamic derangement (avg 16 minutes) by identifying elevated (>6 mmHg) CO_2_ difference.

**Conclusions:** This case report underlines the importance of pulmonary artery to arterial CO_2_ difference as an early marker of hypoperfusion if compared to lactate level in the intensive care unit. To our knowledge this is the first report on venous to arterial carbon dioxide difference in VA ECMO. Further investigation is needed to confirm those preliminary results.

**References**

1. Naumann DN, Midwinter MJ, Hutchings S: Venous-to-arterial CO2 differences and the quest for bedside point-of-care monitoring to assess the microcirculation during shock. *Annals of translational medicine* 2016, 4(2):37.

2. Johnson G, Tamblyn J: Model of pCO2 gap during hypothermic cardiopulmonary bypass. *ASAIO journal* 2006, 52(5):588–591.

3. Futier E, Robin E, Jabaudon M, Guerin R, Petit A, Bazin JE, Constantin JM, Vallet B: Central venous O(2) saturation and venous-to-arterial CO(2) difference as complementary tools for goal-directed therapy during high-risk surgery. *Critical care* 2010, 14(5):R193.

## THE THREAT OF MULTIDRUG-RESISTENCE IN THE ICU

### A1146 The incidence of acquired resistance in AGNBS during 20 years of SDD in a Dutch ICU

#### S.H. Buitinck^1,2^, P.H.J. van der Voort^1,2^

##### ^1^OLVG Hospital, Department of Intensive Care, Amsterdam, Netherlands; ^2^Tilburg University; TIAS School for Business and Society, Tilburg, Netherlands

###### **Correspondence:** S.H. Buitinck – OLVG Hospital, Department of Intensive Care, Amsterdam, Netherlands

**Introduction:** Many studies have shown clinical benefits from SDD for critically ill patients. However, there is still doubt concerning the emergence of antimicrobial resistance in the long term. Previously no evidence to support this view was found but long-term effects of SDD on antimicrobial resistance on the unit level is understudied.^1,2^

**Objectives:** To determine the incidence of antimicrobial resistance in aerobic gram-negative potentially pathogenic micro-organisms (AGNBs) to the components of SDD and frequently used i.v. antibiotics on ICU-level over a 20 year period with unchanged antibiotic policy.

**Methods:** This is a single-center observational cohort study in a Dutch 20-bed adult intensive care unit in a teaching hospital. All consecutive patients admitted to the ICU between January 1994 and December 2013 were included when at least one culture was taken during ICU-admission. Data on all cultures taken during ICU stay were collected from the hospital database. Susceptibility testing was performed following the guidelines of the 'clinical and laboratory standards institute' (CSLI) until 2011 and 'the European committee on antimicrobial susceptibility testing' (EUCAST) from 2011 until 2013.

Incidence rates of antimicrobial resistance to tobramycin, ciprofloxacin, polymyxin B or cefotaxime were calculated per year. Only ICU-acquired resistant pathogens were selected by excluding resistant pathogens in cultures taken on day 1-day 3. Patients at risk were defined as all admissions with a length of stay longer than 2 days. Differences between the incidence in the first and last year of the study were tested using Chi-square test.

**Fig. 136 (abstract A1146).**
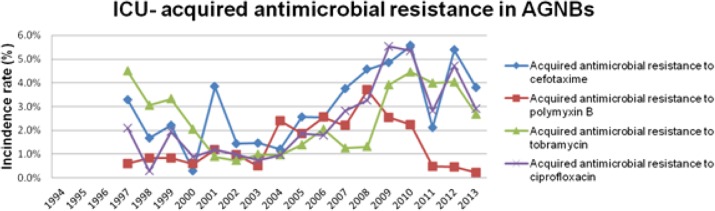
ICU-acquired antimicrobial resistance in AGNBs

**Results:** Data of 127.830 cultures was analyzed containing 15.557 AGNBs. The number of admissions with a length of stay more than 2 days was 6781. In 237 admissions newly acquired resistance to cefotaxime was found, in 117 to polymxin B, 183 to tobramycin and 180 to ciprofloxacin. Figure 136 presents incidence rates per year. In 1994–1996 date of discharge to the ward was unknown and therefore incidence rates could not be calculated for these years but absolute numbers were comparably low. There was no significant difference in incidence of ICU acquired resistance in cefotaxime (χ^2^ = 0.14, *p =* 0.71*)*, polymyxin B (χ^2^ = 0.71, *p =* 0.40*)*, tobramycin (χ^2^ = 1.89, *p =* 0.17*)* and ciprofloxacin (χ^2^ = 0.28, *p =* 0.59*)* between 1997 and 2013.

**Conclusions:** The incidence of newly acquired AGNB resistant to cefotaxime, polymyxin B, tobramycin and ciprofloxacin continues to be low during a 20 year unchanged antimicrobial policy of SDD. The increase in resistance in the society may impact these numbers and should be studied.

**References**

1. Daneman N. et al. *Lancet. Infect. Dis.***13,** 328–341 (2013).

2. Houben, A.J.M. et al. *J.antimicrob Chemother*. **69**, 797–804 (2013).

### A1147 Bacterial colonization and complications with peripheral intravenous catheter in critically ill patients: closed- vs. open-system

#### J. Oto^1^, E. Nakataki^2^, Y. Tsunano^2^, M. Izawa^2^, N. Tane^1^, M. Onodera^2^, M. Nishimura^2^

##### ^1^Tokushima University Hospital, Emergency and Disaster Medicine, Tokushima, Japan; ^2^Tokushima University Graduate School, Emergency and Critical Care Medicine, Tokushima, Japan

###### **Correspondence:** J. Oto – Tokushima University Hospital, Emergency and Disaster Medicine, Tokushima, Japan

**Introduction:** Adverse effects of peripheral intravenous catheters (PIVCs), such as phlebitis, extravasation or catheter-related infection, are not rare. To prevent them, closed-system PIVC is introduced, however its clinical performance has not been well evaluated.

**Objectives:** To compare the incidence of bacterial colonization and PIVCs-related complications with closed- and open-system PIVCs in critically ill patients.

**Methods:** Patients older than 18 years needing PIVCs for at least 24 h were enrolled. They were randomly assigned to using closed-system PIVC (Safewing cath^TM^, Japan Medical Supply Co., Ltd, Tokyo, Japan) or open-system PIVC (Supercath 5^TM^, Medikit Co., Ltd, Tokyo, Japan). The closed-system PIVC was composed of catheter with integrated extension tube, a stabilizing wings and a passive needle shielding mechanism. The open-system PIVC has just passive needle shielding mechanism. The PIVCs were removed according to clinical indications. After removing PIVCs were sent to laboratory for bacterial culture. One PIVC from each patient was included for the study. Indwell time of PIVCs, the incidence of bacterial colonization and PIVCs-related complications (phlebitis, extravasation, catheter occlusion and hematoma) were recorded. The protocol of the study was approved by the IRB of Tokushima University Hospital.

**Results:** Ninety-one closed-system PIVCs and 89 open-system PIVCs were evaluated. The median indwell time did not differ between the closed- and open-system PIVCs (median [inter quartile range], closed-system PIVCs: 79 [43, 120] vs. open-system PIVCs: 58 [39, 118] h, p = .07). The closed-system PIVCs reduced the incidence of bacterial colonization (closed-system PIVCs: 20.9 cases/1000 catheter-days vs. open-system PIVCs: 51.9cases/1000 catheter-days, p = .03). The incidence of PIVCs-related complications were not different between the closed- and open-system PIVCs (closed-system PIVCs: 120.8 cases/1000 catheter-days vs. open-system PIVCs: 162.9 cases/1000 catheter-days, p = .16).

**Conclusions:** The closed-system PIVCs reduced the rate of bacterial colonization, while it did not reduce PIVCs-related complications.

**Grant acknolwedgment**

Departmental funding.

### A1148 Colistin resistance in enterobacteriacae: 1-year clinical data from an Indian hospital

#### S. Ghosh^1^, A. Gupta^2^

##### ^1^Fortis Escorts Hospital, Critical Care Medicine, Faridabad, India; ^2^Fortis Escorts Hospital, Microbiology, Faridabad, India

###### **Correspondence:** S. Ghosh – Fortis Escorts Hospital, Critical Care Medicine, Faridabad, India

**Introduction:** With emergence of multidrug resistant enterobacteriacae isolates, colistin is increasingly being used for treating enterobacteriacae infection. However, resistance to Colistin is being reported in these pathogens.

**Objectives:** To investigate clinical characteristics of patients whose clinical specimen has grown Colistin-resistant Klebsiella pneumonae (KP) and Escherichia coli (EC).

**Methods:** Study was performed in a 213-bed urban tertiary care private hospital. All patients, from whom a clinical specimen yielded a KP or EC isolate resistant to Colistin (VITEK2 COMPACT,bioMerieux, Hazelwood, MO). Isolates were considered colistin sensitive with MIC < 0.5 mcg/ml and colistin resistant with MIC >16 mcg/ml), in the time period between 01 January 2015 and 31 December 2015, were identified from the database of the microbiological laboratory of the hospital and were included in this study as cases. Demographic data, severity of illness, risk factors for colistin-resistance (described in previous studies), clinical management and hospital outcome of all cases were recorded. MDR - Resistant to at least one agent from 3 different classes. PDR - Resistant to all classes.

**Results:** 13 of 207 KP (6.2 %) and 8 of 445 EC (1.8 %) isolates were colistin resistant. 14 of 207 (6.76 %) of KP and 8 of 445 EC (1.8 %) isolates were colistin resistant. Total no of patients identified was 19 (22 isolates - 7 Respiratory, 15 Urine). 2 isolates were considered colonization. 31.5 % of these patients had a previous exposure to colistin. 31.5 % did not have any known risk factors (including 3 infants). PDR EC was isolated from a 1-year old infant urine without any risk factors. 78.5 % of KP isolates were PDR, rest being MDR. 3 of 8 EC isolates (37.5 %) were MDR. 1 EC isolate was PDR.Non-standardized combination of agent prescribed. 9 of 19 patients expired (47.3 %, culture report received post-mortem in 4 patients). One patient got discharged and report was overlooked. 1-Patient was discharged with persistent urinary colonization with PDR KP.

**Conclusion:** Colistin resistance is increasing in Enterobacteriacae isolates with limited treatment options - most of these isolates being MDR or PDR. Mortality remains high. Prior use of Coloistin is strongly associated with resistance. To prevent this phenomenon judicious use of colistin is the need of the hour.

**References**

1. Antoniadou A, Kontopidou F, Poulakou G, et al. Colistin-resistant isolates of Klebsiella pneumoniae emerging in intensive care unit patients: first report of a multiclonal cluster. J Antimicrob Chemother. 2007; 59:786–90.

2. Matthaiou DK, Michalopoulos A, Rafailidis PI, et al. Risk factors associated with the isolation of colistin-resistant gram-negative bacteria: a matched case-control study. Crit Care Med. 2008; 36:807–11.

**Grant acknolwedgment**

Nil

### A1149 Carbapenem resistant *Klebsiella Pn* (CRKP) infections early after liver transplantation (OLT): single center experience

#### A. De Gasperi^1^, E. Mazza^2^, R. Limuti^2^, M. Prosperi^2^

##### ^1^Niguarda Ca Granda Hospital, Anesthesia CCM 2, Milan, Italy; ^2^Niguarda Ca Granda Hospital, Milan, Italy

###### **Correspondence:** A. De Gasperi – Niguarda Ca Granda Hospital, Anesthesia CCM 2, Milan, Italy

**Introduction:** CRK Pn infections are increasing among liver transplanted patients in Italy. In the most recent series, one year mortality rate was reported to range between 45 and 71 %.

**Objectives:** To study the incidence of CRKP infections after OLT.

**Methods:** Retrospective analysis of 310 consecutive patients (pts) who underwent 336 OLT procedures (26 reOLT) from Jan 2012 to Dec 2015 to define

1. the incidence of CRKP postOLT infection;
2. the impact on ICU and Hospital (H) mortality;
3. the associated risk factors.

**Results:** Overall, ICU and H mortality rates were 94 and 87 %. 17.7 % of the pts (55/310) became infected in the early postoperative period. (ICU and H mortality rate 21 and 29 % respectively). CRPK infections were present in 8 pts (2.5 % of the entire series, 14.5 % of the infected pts). Sarcopenia (50 % vs 10 %, p = 0.0045) and MELD (29 + 9 vs 19 + 8, p = 0.0022) were significant preoperative risk factors. ICU and H mortality rates were 37 % and 75 % in CRPK pts, 19 % and 25 % in non - CRKP infected pts respectively : while ICU mortality was not different (p = 0.65), H mortality was significantly higher in CRKP pts (p = 0.0056 ICU vs H, CI 7.3 - 74.6). If compared to non -CRKP pts , CRKP pts were more often in septic shock (75 % vs 34 %, p = 0.031) and more frequently underwent CRRT (75 % vs 36 % p = 0.0404). Intraabdominal infections were largely represented (80 %) among CRKP pts. Blood loss and transfusion needs, Early gratt dysfunction and reOLT were more represented in infected vs non infected pts. However, no differences were found when CRKP and non- CRKP transplanted pts were compared.

**Conclusions:** CRKP infections are on the rise also in Italy. Post OLT mortality is high and strategies able to control CRKP are urgently needed to be implemented.

### A1150 The problem of Acinetobacter baumannii associated infection in ICU: 5 - year study

#### N. Bissenova, A. Yergaliyeva

##### National Scientific Medical Research Center, Clinical Microbiology, Astana, Kazakhstan

###### **Correspondence:** A. Yergaliyeva – National Scientific Medical Research Center, Clinical Microbiology, Astana, Kazakhstan

**Introduction:** The prevalence of antibiotic- resistant pathogens in ICU conditions makes it difficult to treat these infections, and treatment becomes impossible in some cases. Acinetobacter baumannii is important infectious agent ICU patients, which effective antibiotic therapy is currently limited.

**Objectives:** We aimed to determine the range of A.baumannii associated infections among ICU patients, to summarize the level of resistance to antimicrobial drugs, and provide an overview of strategies to prevent the spread of resistance.

**Methods:** A prospective microbiological study of the prevalence and antibiotic resistance of A.baumannii strains isolated from adult ICU patients hospitalized to the tertiary hospital after cardiac surgery from 2010 to 2014.

**Results:** A total of 781isolates from ICU patients were included to the study. 52.3 % of the isolated strains (409) were Gram-negative, among which 20.9 % (164) of A.baumannii isolates. Strains of A. baumannii showed a high level of resistance to the III generation cephalosporins (96.9 % to ceftazidime, 97.6 % to cefotaxime, 93.5 % to ceftriaxone). Resistance to carbapenems was at 88 %. Investigation of antimicrobial activity of ciprofloxacin showed the resistance in 96.8 % of strains, to levofloxacin - 88.9 %. The lowest level of resistance recorded to doxycycline - 24.5 % and polymyxin - 4.8 %.

**Conclusions:** Rapid microbiological diagnostics (including the results of antibiotic resistance), strict adherence to infection control, the appointment of an effective regime of antibiotic therapy, optimization schemes appointment of antibiotics, all of which are the most important priorities for the effective fight against A. baumannii associated infections in ICU patients. In order to reduce the emergence and spread of drug-resistant strains in the ICU, it is strongly recommended to carry out microbiological monitoring and optimization of the use of antibiotics in each hospital. Therefore local resistance surveillance programs have the greatest value in the development of appropriate therapeutic recommendations for specific types of patients and infections.

**References**

Shorr AF, Zilberberg MD, Micek ST, Kollef MH. Predictors of hospital mortality among septic ICU patients with Acinetobacter spp. bacteremia: a cohort study. BMC Infect Dis 2014. 14:572

### A1151 Rifampicin combination therapies for the treatment of icu infections due to colistin resistant acinetobacter SPP

#### L. Talan^1^, G. Yılmaz^2^, G. Güven^1^, F. Yoruk^2^, N.D. Altıntas^1^

##### ^1^Ankara University Faculty of Medicine, Department of Internal Medicine Division of Intensive Care, Ankara, Turkey; ^2^Ankara University Faculty of Medicine, Clinical Microbiology and Infection Disease, Ankara, Turkey

###### **Correspondence:** N.D. Altıntas – Ankara University Faculty of Medicine, Department of Internal Medicine Division of Intensive Care, Ankara, Turkey

**Introduction:** Acinetobacter spp. are opportunistic, nosocomial pathogens that may colonize the surfaces in intensive care units. Their tendency to harbor multi-drug resistance and to develop resistance mechanisms to commonly available drugs make their treatment a challenge. Carbapenem resistance, and newly reported colistin resistance has led to a search for new treatment options. There are in vitro studies which report synergistic effect with rifampicin in combination therapies.

**Objectives:** We aimed to present and discuss the results of our patients who were infected with either panresistant (5 patients) or only tigecycline susceptible (5 patients) acinetobacter spp. and were treated with rifampicin combination regimens.

**Methods:** Patients reported to be infected with colistin resistant acinetobacter spp. and treated with rifampicin combination regimens upon decision of the responsible teams were traced from the intensive care unit (ICU) records between the years 2014 and 2016 retrospectively. Their demographic data, liver function tests, ICU and hospital outcomes were recorded.

**Results:** There were a total of 10 patients, 6 were women. Mean age was 69.5. In 8 patients pulmonary site was the source. Nine patients had positive blood cultures. Mean SOFA score at the start of therapy was 14.5; all were intubated, and 5 (50 %) were on vasopressor therapy. Combination regimens comprised of at least 3 antibiotics and all regimens included rifampicin and tigecycline. At the end of first week, mean SOFA score was 12.6. Of these 7 (70 %) survived to hospital discharge. Patients who were lost had higher initial and follow-up SOFA scores. Initial and follow-up liver enzymes and renal function tests were similar to their basal values in patients who survived; unlike the patients who were lost. When lost patients were re-evaluated: the first patient had irreversible lung fibrosis due to bleomycine; in the second patient; combination treatment was delayed until 9 days after the cultures were performed; the third patient had been admitted to ICU with acute renal failure and acute respiratory distress syndrome, after autologous stem cell transplantation for multiple myeloma.

**Conclusions:** When the importance of accurate antibiotic choice is taken into account for treatment success; rifampicin combinations may be considered as an appropriate treatment option for infections caused by colistin resistant acinetobacter strains.

**References**

Poulikakos P. et al. Combination antibiotic treatment versus monotherapy for multidrug-resistant, extensively drug-resistant,and pandrug-resistant Acinetobacter infections:a systematic review. Eur J Clin Microbiol Infect Dis. 2014 Oct;33(10):1675–85.

Dizbay M. et al.In vitro synergistic activity of tigecycline and colistin against XDR-Acinetobacter baumannii. The Journal of Antibiotics (2010) 63, 51–53.

**Grant acknolwedgment**

None.

### A1152 Emergence of colistin resistant enterobacteriacae in Indian intensive care units and role of intravenous fosfomycin therapy

#### D.N. Mukherjee^1^, L.K. Agarwal^2^, K. Mandal^3^

##### ^1^Woodlands Multispeciality Hospital, Clinical Microbiology Dept, Kolkata, India; ^2^Woodlands Multispeciality Hospital, Nephrology Dept, Kolkata, India; ^3^Cure Clinic Nursing Home, Kolkata, India

###### **Correspondence:** D.N. Mukherjee – Woodlands Multispeciality Hospital, Clinical Microbiology Dept, Kolkata, India

**Introduction:** Carbapenem resistant enterobacteriacae (CRE) emerged in recent years as one of the most challenging group of antibiotic resistant pathogens. Polymyxins are considered as the last resort for the treatment of infections with carbapenem resistant gram negative bacilli (GNB). Inadequateor extensive use of colistin leads to emergence of colistin resistance, increasing mortality and morbidity and necessitating prudent use of alternative antibiotics. Fosfomycin, a phosponic acid derivative which acts by disrupting bacterial cell wall synthesis, is a broad spectrum antibiotic. It is available as sodium/disodium formulation for intravenous use and is showing promising result against multi drug resistant(MDR)/Pan Drug Resistant (PDR) pathogens.

**Methods:** A total of eight colistin resistant (MIC ≥ 4) GNB were isolated from ICU patients with nosocomial MDR infections during a period of one year. All eight isolates were *Klebsiella pneumonia*. Among these isolates five were from blood and three from endotracheal aspirate. All the isolates were sensitive to fosfomycin in vitro. All of these patients had multiple co-morbidities with recent history of colistin exposure. Intravenous fosfomycin was given as a combination therapy.

**Results:** Among the five bacterimic patients, three recovered completely from sepsis. One patient took discharge against medical advice and the only one bacterimic patient who died during the course of therapy was later on diagnosed to have azole resistant fungemia as super infection. The patient with ventilator associated pneumonia also responded well after initiation of fosfomycin therapy. Average duration of antibiotic therapy in all these cases was ten days.

**Conclusions:** Based on the evidence of clinical experience and available studies, intravenous fosfomycin therapy may be considered as the last option for the treatment of MDR GNB infection where there is documented colistin resistance and where there is literally no other choice of antibiotic therapy. The success of the therapy is encouraging in selected group of patients. Further research on intravenous fosfomycin use specially against MDR pathogens and on the effectiveness and safety of the drug in the treatment of patients with such infections may be warranted.

### A1153 Evolution of ICU acquired multiresistant bacterias

#### M. Palomar^1^, B. Balsera^1^, M. Vallverdu^1^, M. Martinez^2^, M. Garcia^1^, D. Castellana^1^, R. Lopez^1^, F. Barcenilla^1^

##### ^1^HU Arnau de Vilanova, Lleida, Spain; ^2^IRBLL, Lleida, Spain

###### **Correspondence:** M. Palomar – HU Arnau de Vilanova, Lleida, Spain

**Introduction:** Multiresistant bacterias (MRB) are an increasing worldwide threat. Surveillance and infection control measures are important in combating resistance

**Objectives:** To describe the trends in the acquisition (infection or colonization) of MRB in ICU over 13 years, as well as the impact of surveillance and prevention programs.

**Methods:** Prospective study from 2003 to 2015 in a 20-bed ICU in an university hospital. All MRB (MRSA, *P aeruginosa, A baumannii, ESBL Klebsiella pn,* ESBL- *E coli*, C difficile) producing colonization or infection were documented. They were counted 1 time by MRB. Surveillance was introduced in 2006 (at admission and 1 times weekly) and in 2013 a national control program was implemented Zero Resistance). The incidence density (ID) was calculated for 1000 days in ICU. Negative Binomial regression models were fitted to the incident number of infections with an offset with the annual number of ICU stays.

**Fig. 137 (abstract A1153).**
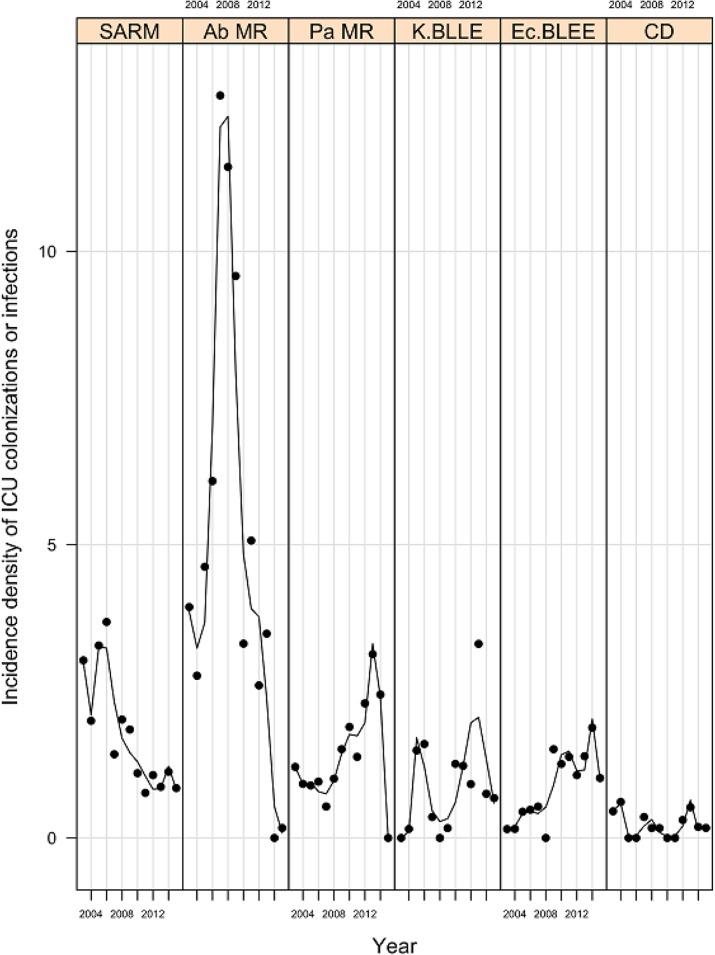
Observed and adjusted trends (time modelling)

**Fig. 138 (abstract A1153).**
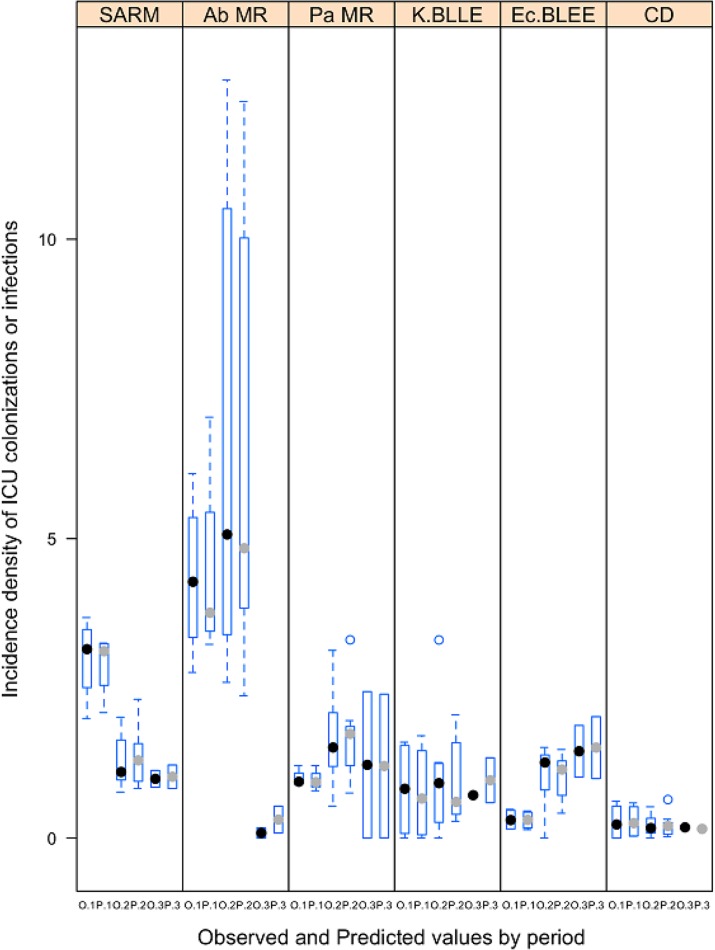
Observed and adjusted trends by period

**Results:** A total of 815 MRB were acquired during the 79896 ICU days of stay (ID 10,2 x 1000 ICU days). In 306 cases were infections (ID 3,82) and 509 colonization (ID 6,38). The MRB were N total (infection): *A baumannii* 401(101), MRSA 144 (70), *P aeruginosa* 111 (74),ESBL- *Klebsiella pn* 74(21), ESBL-*E coli* 68(27) and *C Difficilli* 18(18). The annual decrease in overall SARM infections was an estimated IDR of 0.89 per year, with p-value = 0.013; There was a significant annual decrease in the overall number of incident *A baumannii* infections per year since 2003 until 2015, estimated in 0.86, with p-value = 0.0007. The annual increase in overall E coli ESBL was also significant and an estimated IDR of 1.19 per year, with p-value = 0.002. Absence of an statistically significant trend or change for the other MRB. Figures 137 and 138

**Conclusions:** Changes in the type of MBR were observed along the period, with ESBL producing enterobacteria increase and decrease of MRSA and *A. baumannii*

**References**

1. Combatting resistance in intensive care: the multimodal approach of the Spanish ICU “*Zero Resistance*” program. Garnacho Montero et als. Crit Care. 2015; 19(1): 114

### A1154 Principal features in potsurgical patients with multidrug-resistant organisms isolation, acquired before the admission at the intensive care unit

#### G.E. Kaminsky, R. Carreño, A. Escribá, M. Fuentes, V. Gálvez, R. Del Olmo, B. Nieto, C. Vaquerizo, J. Alvarez, M.A. De la Torre, E. Torres

##### Hospital Universitario de Fuenlabrada, Intensive Care Unit, Madrid, Spain

###### **Correspondence:** G.E. Kaminsky – Hospital Universitario de Fuenlabrada, Intensive Care Unit, Madrid, Spain

**Introduction:** Patients at the Intensive care units have an increased risk of infection due to their underlying diseases or conditions, impaired immunity, and exposure to multiple invasive procedures (surgery, mechanical ventilation, central venous catheters, artherial catheters, urinary tract catheters). Multidrug-resistant organisms infection has become a public health problem and has been associated with increased morbidity, mortality, and costs.

**Objectives:** To analyze the principal features of postsurgical patients with colonization or infection by multidrug-resistant organisms, acquired before the admission at the Intensive Care Unit.

**Methods:** Retrospective observational study, descriptive, case series, collected from 04/01/2014 to 29/11/2015 in a 400-bed hospital, with a 10-bed polyvalent Intensive Care Unit in Fuenlabrada, Madrid, Spain. The Hospital is attached to Resistance Zero Project, with screening at admission and every week in all patients (pharyngeal, rectal, nasal, wounds and bronchial suction).

18 postsurgical patients have been identified with multidrug-resistant organisms isolation in screening in the first 24 hours of admission to the Intensive Care Unit.

Studied Variables: age, sex, adjusted Charlson comorbidity index, Barthel index, APACHE II, SAPS 3, days of hospitalization prior to ICU, days of antibiotic treatment administered before ICU, previous days of parenteral nutrition, prealbumin, surgical wound infection, multigrug-resistant organisms identified sample. Statistical analysis: SPSS20. Categorical in frequencies and percentages, mean and standard deviation or median and interquartile range. Analysis Kolmogorov Smirnov, Shapiro Wlik and QQPlot to normality. Confidence intervals (CI) 95 % by T Student for normal variables, Boot stramp to not normal.

**Results:** In 20 months we identified 18 postsurgical patients with multidrug-resistant organisms in screening at the admission or before the admission to the Intensive Care Unit.

Multidrug-resistant organisms identified: Pseudomonas aeruginosa 6 (33.3 %), ESBL enterobacteriaceae 6 (33.3 %), MRSA 3 (16.7 %), Stenotrophomonas maltophilia 1 (5.6 %).

Isolated on: surgical wound 7 (38.6 %), bronchial suction 2 (11.1 %), peritoneal fluid 2 (11.1 %), exudates monitoring 2 (11.1 %), blood 1 (5.6 %).

Antibiotic therapy: Carbapenem 8 (44.4 %), Piperacilina-tazobactam 6 (33.3 %).

**Conclusions:** In our study the risk of prior acquisition of multidrug-resistant organisms at the admission to the Intensive Care Unit in postsurgical patients was characterized by long hospital stay, high comorbidity and dependence, malnutrition, prolonged use of broad-spectrum antibiotic, parenteral nutrition and surgical wound infection.

**Table 90 (abstract A1154).** Variables

Variables		Confidence Intervals 95 %
Hospitalization days prior ICU admission	21,0 +/− 20,0	7,5–23
Antibiotic days prior ICU admission	8 +/− 8	7,0–11,0
Days with parenteral nutrition prior ICU admission	4 +/− 18,3	0–14,5

**Table 91 (abstract A1154).** Variables

Variable		Confidence Intervals 95 %
Age	62,3 +/− 12,8	56,0–68,7
Sex	10 (55.6 %)	30–78
Charlson	7,3 +/− 3,4	5,6–9,0
Barthel	56,7 +/− 24,43	44,5–68,8
APACHE II	18 +/− 4,7	15,6–20,4
SAPS 3	62,2 +/− 14,4	55,0–69,4
Prealbumin	8,6 +/− 11,6	5,2–14,1
Wound infection	16 (88.9 %)	58,5–96,4

### A1155 *Klebsiella pneumoniae* carbapenemase (KPC) a case-control study in an university hospital in Rio de Janeiro

#### E. Bogossian^1^, S. Aranha Nouer^2^, D. Ribeiro Salgado^1^

##### ^1^Universidade Federal do Rio de Janeiro, ICU, Rio de Janeiro, Brazil; ^2^Universidade Federal do Rio de Janeiro, Infectious Diseases, Rio de Janeiro, Brazil

###### **Correspondence:** E. Bogossian – Universidade Federal do Rio de Janeiro, ICU, Rio de Janeiro, Brazil

**Introduction:** The emergence and dissemination of Klebsiella pneumoniae carbapenemase (KPC) is of great concern. Outbreaks have been reported in different types of Intensive Care Units (ICU). In Brazil, there have been reports of KPC since 2006. We recently experienced a large outbreak at our hospital. Risk factors for KPC colonization and outcome of ICU patients are still to be determined.

**Objectives:** To study the differences between patients who acquired from those who did not acquired KPC during their stay in the ICU, focusing on risk factors and outcomes.

**Methods:** A retrospective case-control study was conducted from May 2014 to January 2015 during a KPC outbreak in the 10-bed ICU of a tertiary university hospital in Rio de Janeiro, Brazil. All patients admitted to the ICU were included in the study and classified as case (KPC yielded from any biological material, either considered as colonization or infection) or control (all other patients who did not have KPC isolation). Both groups were compared according to demographic data, comorbidities, sepsis diagnosis, type and time of life support, SOFA and SAPS III scores at ICU admission, length of stay (LOS) at ICU and hospital, and hospital costs, ICU and hospital mortality.

**Results:** 63 patients were admitted during the studied period. 24 patients had KPC samples isolated from different biological material, but only 3 were considered as having KPC infection. There was no difference between cases and controls patients considering gender, age, type of admission, and SAPS III and SOFA scores on ICU admission. Patients with KPC had greater ICU and hospital LOS than control patients (33 [13–60] vs 5 [1–12] days, p < 0.001 and 57 [37–80] vs 20 [11–37] days,

p < 0.001, respectively) Both groups had similar frequency of sepsis, but KPC patients had more life-organ support requirements as mechanical ventilation (96 % x 54 %; p = 0.001) and dialysis

(50 % x 24 %; p = 0,008). ICU and hospital mortality rate was higher in KPC than in control group

(45.8 % vs 25.6 % p = 0.099) and (79.2 % vs 35.9 % p = 0.002), respectively. Hospital costs were higher in KPC than control patients ($ 1260 vs $ 630, p < 0.05).

**Conclusions:** During a KPC outbreak in the ICU of an academic tertiary hospital in Rio de Janeiro, the isolation of KPC associated with colonization or infection was associated with greater ICU and hospital LOS, more requirements of life-organ support, higher ICU and hospital mortality rates, and higher hospital costs.

**References**

1) Hussein, K. Impact of carbapenem resistance on the outcome of patients' hospital-acquired bacteraemia caused by *Klebsiella pneumoniae*. Journal of Hospital Infection 83 (2013).

2) Monteiro J e col. First report of KPC-2-producing *Klebsiella pneumoniae* strains in Brazil. Antimicrob Agents Chemother 2009.

3) Borer A, et al. Attributable mortality rate for carbapenem-resistant *Klebsiella pneumoniae* bacteremia. Infect Control Hosp Epidemiol 2009

### A1156 Utility of surveilance cultures for multirresistant bacteria detection

#### S. Carvalho Brugger, G. Jiménez Jiménez, M. Miralbés Torner, M. Vallverdú Vidal, B. Balsera Garrido, X. Nuvials Casals, F. Barcenilla Gaite, J. Trujillano Cabello, M. Palomar Martínez

##### Hospital Universitario Arnau de Vilanova, Lleida, Spain

###### **Correspondence:** S. Carvalho Brugger – Hospital Universitario Arnau de Vilanova, Lleida, Spain

**Introduction:** Multirresistant bacteria (MRB) development is a growing phenomenon. In 2013, the “Zero Resistance” (RZ) program was launched in Spain, to help prevent the emergence of MRB in critically ill patients. One of its recommendations is to complete a checklist upon patient admission in Intensive Care Unit (ICU) to identify those patients at high risk for colonization or infection by MRB.

**AIMS.** To assess the relation between most common MRB and specific risk factors (RF) for colonization or infection, and the samples where they are identified, assessing the cultures profitability.

**Methods:** A prospective study from March/14 to January/16. All patients admitted to a polyvalent ICU of a general hospital were submitted to the checklist proposed, with the application of contact precaution measures in patients at risk for colonization or infection by MRB. Bacteriologic swabs (nasal, pharyngeal, axillary and rectal) were routinely performed to all patients admitted, besides diagnostic cultures when needed. Furthermore, we analysed other pathological variables and comorbidities. The difference between groups of MRB was made by Chi-square test for qualitative variables and the Kruskal-Walls test for the continuous ones. Statistical significance was set at P < .05.

**Results:** 1651 admitted. In 136 patients were identified one or more MRB (148 in total). 60 patients (40,5 %) were ESBLs carriers, 47 (31,7 %) MRSA, 23 (15,5 %) *P aeruginosa*, 13 (8,8 %) *Acinetobacter spp* and 6 (4 %) others MRB carriers. In 36 cases (27,2 %) the presence of a MRB caused infection. Nasal swabs detected 31 % of MRB carriers (64 % of all MRSA), pharyngeal swabs 32 % (44,7 % of MRSA), axillary swabs 13 % (19 % of MRSA, 23 % of *Acinetobacter)*, and rectal swabs 36 % (82 % of ESBLs, 46,2 % of *Acinetobacter)*. In 4 cases (13 %) just the axillary swab was positive, and in 35 cases (36 %) the rectal was the only swab able to detect a MRB. Diagnostic cultures (blood, urine, bronchoaspirate, surgical wound and others) detected MRB in less than 30 %. The checklist did not detected neither colonization nor infection by MRB in 49 (36 %) patients (45 % MRSA, 61,5 % *Acinetobacter*, 30 % of ESBLs). All patients with *P aeruginosa* had RF, but one. There was no statistical significance between groups of MRB and other comorbidities.

**Conclusions:** The surface cultures realized at admission detected 70 % of MRB not detected by diagnostic cultures. In our environment, we observed a sampler different from the Spanish one, with a predominance of ESBLs, followed by MRSA, higher than the national media. MRSA carriers were identified mostly by nasal swabs while the ESBL carriers were identified mainly by rectal swabs. The rectal, nasal and pharyngeal swabs were the most useful to detect MRB.

**References**

J Montero et al. (Scientific Expert Committee for the “Zero Resistance” Project). Combatting resistance in intensive care: the multimodal approach of the Spanish ICU “Zero Resistance” program. Critical Care (2015) 19:114.

**Fig. 139 (abstract A1156).**
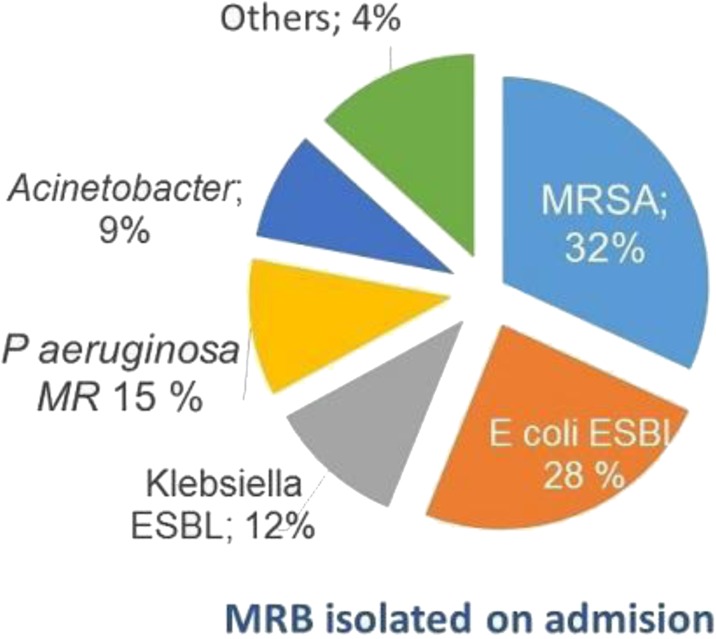
MRB isolated on admision

**Fig. 140 (abstract A1156).**
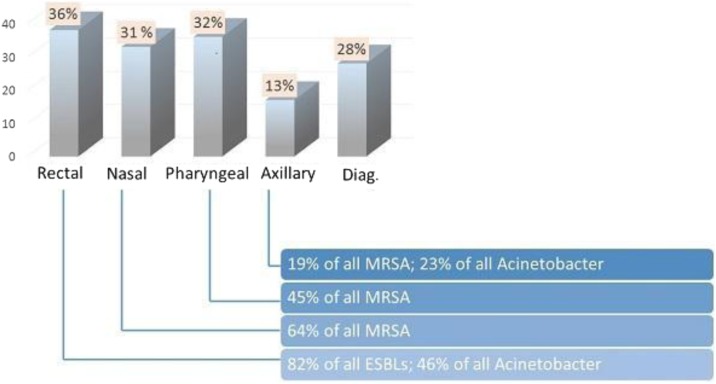
Cultures profitability

**Fig. 141 (abstract A1156).**
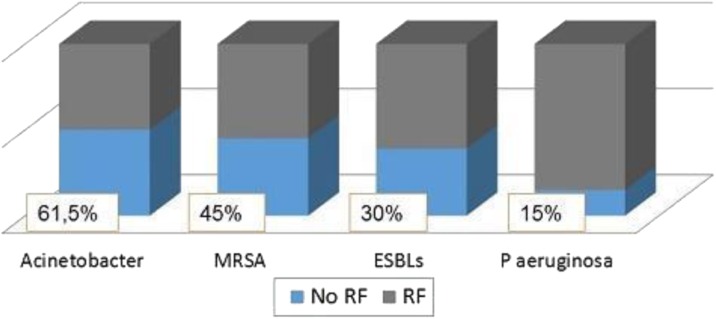
Risk factors and MRB

### A1157 The frequency of the transformation of vancomycin resistance enterococcus colonization into infection in the adult intensive care patients and the evaluation of the risk factors

#### M. Doganci^1^, S. Izdes^1^, S. Guzeldag Besevli^1^, A. Alkan^2^, B. Kayaaslan^3^

##### ^1^Yildirim Beyazit University, Anesthesiology and Reanimation, Ankara, Turkey; ^2^Yildirim Beyazit University, Biostatistics and Medical Informatics, Ankara, Turkey; ^3^Yildirim Beyazit University, Infectious Diseases and Microbiology, Ankara, Turkey

###### **Correspondence:** M. Doganci – Yildirim Beyazit University, Anesthesiology and Reanimation, Ankara, Turkey

**Introduction:** Despite strict infection control measures, vancomycin-resistant enterococci (VRE) colonization and VRE infections are still an important problem today. However, there are very few studies, which examine the factors that lead to the transformation of VRE colonization into VRE infection in intensive care unit (ICU) (1,2).

**Objectives:** This study aims to determine the frequency of the VRE colonization and the transformation into infection and the risk factors, which lead to infection.

**Methods:** The patients who were hospitalized for at least 24 hours in tertiary mixed type ICU between 2012 and 2015 and had VRE colonization and VRE infection during or following their hospitalization were included in the study and their medical records were examined retrospectively. VRE rectal swab sample was taken from each patient at his arrival and once a week afterwards. When negativity was detected in the rectal swab sample, which had been taken total 3 times successively from those with positive VRE; that patient was considered VRE negative. Their demographic data, APACHE II scores, invasive procedures, treatments (corticosteroid, antibiotics, etc.), nutrition types, laboratory results and ICU outcome were recorded.

**Results:** VRE colonization was detected in 110 of 1730 patients (6.4 %) admitted to ICU. VRE infection developed in 12 of 110 VRE-colonized patients (10.9 %). Among these infected patients; it was (n = 5) 41.7 % primary bloodstream infection, (n = 6) %50 urinary tract infection, (n = 1) % 8.3 pneumonia. In VRE colonized patients (64.3 %) and infected patients (91 %), the most frequent factor was E. faecium. In 67 % of the VRE-colonized patients, VRE became negative in their stay at ICU. Previous renal replacement treatment was significantly higher in statistical terms in the VRE-infected group (66.7 %) when compared to VRE-colonized group (26.1 %) (p < 0.05). In the VRE-infected group, colonization with VRE lasted longer than 1 week in 10 patients (83.3 %) were determined. Demographic data, APACHE II scores, treatments, nutrition types**,** previous antibiotic usage and types, invasive procedures, laboratory results and ICU outcome were similar between the VRE-colonized and infected patients.

**Conclusions:** Previous renal replacement treatment and the colonized patients' long stay at ICU increase the transformation of the VRE colonization into VRE infection. Strategies to reduce the duration of ICU stay of VRE-colonized patients are the main objects to controlling VRE infection rate.

**References**

1- Papadimitriou-Olivgeris M, et al. Infection 2014; 42:1013–22

2- Pan SC, et al. PLoS One. 2012; 7(10):e47297.

### A1158 Enteral paramomycin to eradicate colistin and carbepemenase resistant microorganisms in rectal colonization to prevent icu multiresistant nosocomial infections

#### C. Sánchez Ramírez^1^, L. Caipe Balcázar^1^, M. Cabrera Santana^1^, M.A. Hernández Viera^1^, S. Hípola Escalada^1^, C.F. Lübbe Vázquez^1^, S.M. Marrero Penichet^2^, F. Artiles Campelo^3^, M.A. De La Cal López^4^, P. Saavedra Santana^5^, S. Ruíz Santana^1^

##### ^1^University Hospital of Gran Canaria Dr. Negrín, Intensive Care Unit, Las Palmas de Gran Canaria, Spain; ^2^University Hospital of Gran Canaria Dr. Negrín, Pharmacy Department, Las Palmas de Gran Canaria, Spain; ^3^University Hospital of Gran Canaria Dr. Negrín, Microbiology Department, Las Palmas de Gran Canaria, Spain; ^4^Hospital of Getafe, Intensive Care Unit, Madrid, Spain; ^5^University of Las Palmas de Gran Canaria, Mathematics and Informatcs Deparment, Las Palmas de Gran Canaria, Spain

###### **Correspondence:** C. Sánchez Ramírez – University Hospital of Gran Canaria Dr. Negrín, Intensive Care Unit, Las Palmas de Gran Canaria, Spain

**Objective:** To assess the value of enteral paramomycin to decontaminate patients with rectal colistin and/or carbepemenase resistant microorganisms colonization to prevent the development of ICU nosocomial infections

**Methods:** All consecutive patients admitted to the ICU from October 2011 to September 2015, expected to require tracheal intubation for longer than 48 hours, were given SDD with a 4-day course of intravenous cefotaxime, plus enteral colistin, tobramycin, nystatin in an oropharyngeal paste and in a digestive solution. Oropharyngeal and rectal swabs were obtained on admission and once weekly. Rectal swabs colonized by colistin and/or carbepemenase resistant microorganisms were treated with enteral paramomycin 1 gram every 6 hours a day, in order to eradicate them and prevent nosocomial infections. Categorical variables were summarized as frequencies and percentages and the continuous ones as medians and interquartile ranges (IQR) or means and standard desviations. Statistical significance was set at *p* ≤ 0.05.

**Fig. 142 (abstract A1158).**
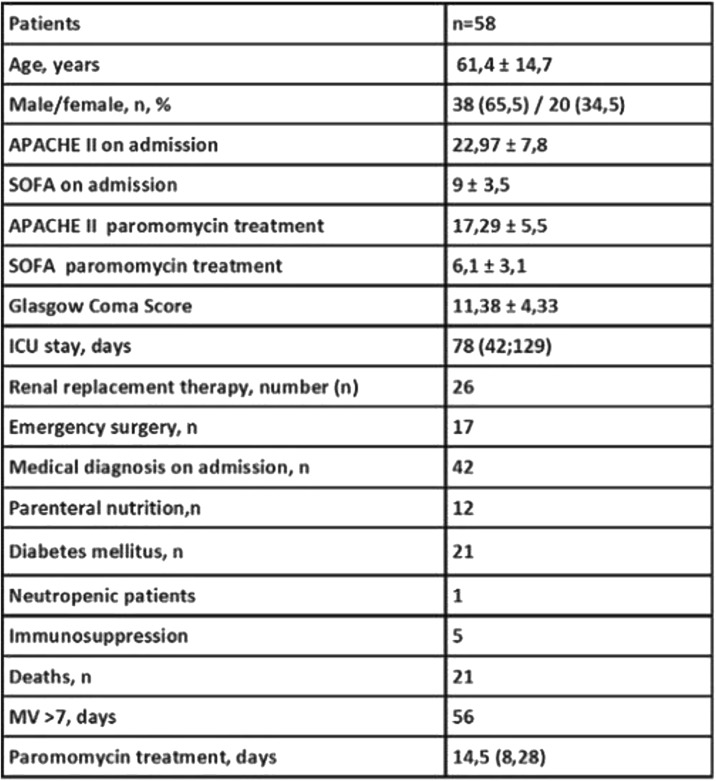
Patients data

**Results:** We applied paromomycin treatment to 58 colonized patients with rectal colistin resistant microorganisms. All of them had colonization by Extended Spectrum Beta-lactamases (ESBLs). Also, all of them but two were *Klebsiella pneumonia.* Out of these two, one patient was colonized by *Enterobacter spp* and other one by *Escherichia coli*. Demographic data and type of admission are shown in Fig. 142.

Forty out of 58 (68,9 %) of the studied patients the rectal swab became negative. Five out of the 58 patients were colonized by carbapenemases producing microorganisms and one of these died with persistent multirresistant rectal colonization. Only 16 out of the 40 patients that negativized the colonization received concurrent susceptible IV antibiotics. Only 1 of the paromonycin treated patients developed a mediastinitis infection due to one of the treated microorganisms. Finally, 21 patients died in the ICU.

**Conclusion:** Our data show that enteral paramomycin is effective in treating rectal colistin and/or carbepemenase resistant microorganisms colonization allowing clinicians preventing the development of ICU nosocomial infections.

### A1159 Epidemiological impact of twin-room intensive care unit on extended-spectrum beta-lactamase-producing *enterobacteriaceae* carriage

#### X. Repessé^1^, M. Artiguenave^1^, S. Paktoris-Papine^1^, F. Espinasse^2^, A. Dinh^3^, F. El Sayed^4^, C. Charron^5^, G. Géri^6,7^, A. Vieillard-Baron^8,9,10^

##### ^1^Intensive Care Unit, Ambroise Paré University Hospital, Assistance Publique, Hôpitaux de Paris, Boulogne-Billancourt, France; ^2^Infection control Unit, Ambroise Paré University Hospital, Assistance Publique, Hôpitaux de Paris, Boulogne-Billancourt, France; ^3^Infectious Diseases Unit, Ambroise Paré University Hospital, Assistance Publique, Hôpitaux de Paris, Boulogne-Billancourt, France; ^4^Microbiology Unit, Section Biology Pathology and Health Products, Ambroise Paré University Hospital, Assistance Publique, Hôpitaux de Paris, Boulogne-Billancourt, France; ^5^Intensive Care Unit, Ambroise Paré University Hospital, Assistance publique, Hôpitaux de Paris, Boulogne-Billancourt, France; ^6^Intensive Care Unit, Ambroise Paré University Hospital, Assistance Publique - Hôpitaux de Paris, Boulogne-Billancourt, France; ^7^Rescu, Li Ka Shing Institute at St Michael's Hospital, Toronto, Canada; ^8^Intensive Care unit, Ambroise Paré University Hospital, Assistance Publique, Hôpitaux de Paris, Boulogne-Billancourt, France; ^9^University of Versailles Saint-Quentin en Yvelines, Faculty of Medicine Paris Ile-de-France Ouest, Saint-Quentin en Yvelines, France; ^10^INSERM U-1018, CESP, Team 5 (EpReC, Renal and Cardiovascular Epidemiology), Villejuif, France

###### **Correspondence:** X. Repessé – Intensive Care Unit, Ambroise Paré University Hospital, Assistance Publique, Hôpitaux de Paris, Boulogne-Billancourt, France

**Introduction:** Multi-drug-resistant organisms (MDRO), particularly extended-spectrum beta-lactamase-producing enterobacteriaceae (ESBL-PE), are responsible for longer length of stay and poorer outcomes in intensive care unit (ICU).

**Objectives:** Our primary objective was to study whether the absence of geographic isolation could be responsible for increased transmission rates of ESBL-PE among ICU patients. Our secondary objectives were to describe the epidemiology of ESBL-PE in our ICU and to identify transmission risk factors.

**Material and methods:** This observational study was prospectively conducted during 11 months (June 2014-April 2015) in the 12-bed ICU of Ambroise Paré hospital, Boulogne-Billancourt, France, a tertiary ICU organized as a six twin bedrooms divided in three units.

The study was limited to consecutive adult patients admitted to ICU during a period that included two distinct nursing shifts. Admission characteristics (age, gender, SAPSII) and clinical data during hospital stay (mechanical ventilation duration, ICU length-of-stay, ICU mortality) were collected. Microbiological colonization and/or acquisition of ESBL-PE were monitored by rectal swabs collected at admission and once weekly for the whole duration of the ICU stay. Each strain of ESBL-PE was then identified and the enzyme type sequenced by PCR.

**Results:** During the study period, 550 patients were admitted to the ICU, among which 470 met the inclusion criteria. At admission, median age, IGS-II and SOFA score were 66 [54–77], 46 [32–62] and 7 [4–9], respectively. Two hundred and eighty three (60.2 %) patients were mechanically ventilated, 224 (47.8 %) received catecholamines and 75 (16 %) were immunocompromised. ICU mortality was 16.4 % and did not differ between ESBL carriers and non-carriers. The rate of ESBL colonization at admission and ESBL acquisition were 13.2 % and 4.3 %, respectively. Escherichia coli was the most frequently observed bacteria. The results of the univariate analysis for ESBL acquisition are presented in Table 93. In multivariate analysis, IGS-II and ICU length of stay were strongly associated with ESBL acquisition (Table 94).

**Discussion and conclusion:** The observed rate of ESBL carriage on admission was comparable to other rates in French ICUs (15 %). Despite the unfavourable twin-bed architecture of our ICU, the incidence of ESBL acquisition was 4.3 % which was actually lower than transmission rates previously published in other ICUs. ESBL acquisition was strongly associated with ICU length of stay and severity score at admission. This study is fully consistent with previous ones challenging the geographic isolation in a non-epidemic setting and suggests that environmental contamination may not play a substantial role in the transmission of ESBL-PE. Still in process PCRs for ESBL strains identification would provide a better understanding of inter-patient ESBL transmission.

**Table 92 (abstract A1159).** Risk factor associated with ESBL carriage

Variable	No ESBL acquisition	ESBL acquisition	p
Age (y)	66 [54–77]	82 [64–82]	0.1
SAPS-II	45 [32–61]	72 [49–80]	0.006
SOFA score	6 [4–9]	10 [7–13]	<0.001
CKD	57 (12.4)	4 (36.4)	0.042
Mechanical ventilation	272 (59.3)	11 (100)	0.004
MV duration (d)	2 [0–5]	8 [4–11]	<0.001
Catecholamine use	213 (46.5)	11 (100)	<0.001
ICU LOS (d)	4 [2–8]	11 [9–26]	<0.001
Duration of antibiotics in ICU (d)	3 [0–6]	8 [5–12]	<0.001

**Table 93 (abstract A1159).** Multivariate analysis

Variable		Odds ratio	95 % confidence interval
ICU LOS (d)	<4	0.01	0.00–0.94
	4–7	1	1.00–1.00
	7–10	2.66	1.01–7.01
	>10	4.39	1.02–18.98
SAPS-II	<32	1.00	1.00–1.00
	33–45	1.68	1.02–2.78
	45–60	2.64	1.04–6.73
	>60	5.29	1.06–26.29

## SPONTANEOUS ASSISTED BERATHING AND WEANING

### A1160 Diaphragmatic thickness at different levels of end expiratory lung volume (EELV) in mechanically ventilated patients

#### K. Marmanidou, M. Oikonomou, C. Nouris, K. Dimitroulakis, E. Soilemezi, D. Matamis

##### Papageorgiou Hospital, Intensive Care Unit, Thessaloniki, Greece

###### **Correspondence:** K. Marmanidou – Papageorgiou Hospital, Intensive Care Unit, Thessaloniki, Greece

**Introduction:** Diaphragmatic thickness increases as lung volume increases towards TLC. It has been shown that in healthy subjects, diaphragmatic thickness, increases as lung volume increases, above 0.5 of the vital capacity (VC). In mechanically ventilated patients, different levels of PEEP are used to improve oxygenation. There is no information about the diaphragmatic thickness when in ICU patients, lung volume increases with PEEP towards TLC.

**Methods:** In patients with Acute Respiratory failure (ARF) and lower lobe atelectasis detected by Lung ECHO, two levels of PEEP (8 and 15 ± cmH2O) are used to increase lung volume and to improve oxygenation. End Expiratory Lung volume (EELV), and diaphragmatic thickness was measured at baseline (ZEEP) and at the two levels of PEEP. EELV was measured with a Nitrogen indirect dilution method and diaphragmatic thickness at the zone of apposition with echography using a 12 MHz linear probe. Statistical analysis was performed by one way ANOVA and normal distribution by Colmogorof-Smyrnof test.

**Results:** 22 patients (17 M and 7 F) with a mean age of 58 ± 18 were studied. Diaphragmatic thickness at baseline was 0,21 cm and EELV at 1276 ml (43 %) of the predicted (2942 ml). At the intermediate and high level of PEEP diaphragmatic thickness did not change significantly (0.22 and 0.23 cm, respectively, p = 0.38) and EELV increased at 55 % (1621 ml) and 68 % (1995 ml) of the predicted. The increase in lung volume induced by PEEP was at 32 % and 40 % of the predicted VC (5049 ml). Mean PaO2/FiO2 ratio did not change significantly

**Conclusions:** Mechanically ventilated patients for ARF have a severe reduction in their EELV or FRC. The use of PEEP re-establishes partially the EELV, but not to his normal levels(predicted FRC). Despite high levels of PEEP, diaphragmatic thickness remained constant because the increase in EELV never attained the 50 % the vital capacity.

**Fig. 143 (abstract A160).**
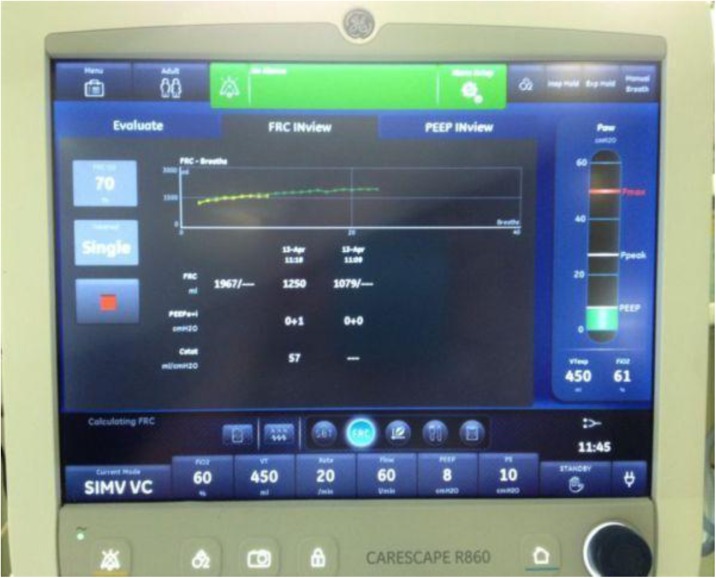
EELV measured with a Nitrogen indirect dilutio

### A1161 Lung ultrasound enables to detect weaning-induced pulmonary oedema

#### A. Ferré^1^, M. Guillot^1^, J.-L. Teboul^1^, D. Lichtenstein^2^, G. Mézière^3^, C. Richard^1^, X. Monnet^1^

##### ^1^Hôpital de Bicêtre, Hôpitaux universitaires Paris-Sud, Université Paris-Sud, Service de réanimation médicale, Inserm UMR_S999, Le Kremlin Bicêtre, France; ^2^Hôpital Ambroise Paré, Hôpitaux universitaires Paris-Ile-de-France-Ouest, Université de Versailles Saint Quentin en Yvelines, Service de réanimation polyvalente, Boulogne-Billancourt, France; ^3^Centre hospitalier de Sens, Service de réanimation polyvalente, Sens, France

###### **Correspondence:** A. Ferré – Hôpital de Bicêtre, Hôpitaux universitaires Paris-Sud, Université Paris-Sud, Service de réanimation médicale, Inserm UMR_S999, Le Kremlin Bicêtre, France

**Purpose.** We tested whether lung ultrasound (LUS) could detect weaning-induced pulmonary oedema (WIPO).

**Methods:** Before and at the end of 62 one-hour T-tube spontaneous breathing trials (SBT) performed in 43 patients, we assessed the LUS profile in the anterior regions of left and right chest walls. WIPO was diagnosed with LUS if, on at least one upper or lower part of both sides, the LUS profile moved from A (normal) to B (interstitial oedema) or from B to “B+”, where B+ consisted in at least a doubling of the B lines number. The reference diagnosis of WIPO was established on other criteria by experts blind for LUS.

**Results:** WIPO occurred in 15 (24 %) SBT. Among cases with WIPO, the LUS profile did not change during SBT in one case, changed for LUS signs of pulmonary oedema in 13 cases (true positives) and changed but without typical LUS signs of WIPO in one case. Among cases without WIPO, the LUS profile did not change during SBT in 26 cases, changed but without typical signs of WIPO in 18 cases and changed with pulmonary oedema signs of WIPO in 3 cases (false positives). LUS diagnosed WIPO with a sensitivity of 81 % (95 % CI: 54-96 %), a specificity of 96 % (95 % CI: 85-100 %), a positive predictive value of 87 % (95 % CI: 60-980 %) and a negative predictive value of 94 % (95 % CI: 83-99 %).

**Conclusions:** LUS performed during a SBT is reliable to establish the diagnosis of WIPO. In particular, it has a very good negative predictive value.

### A1162 Sedation and weaning protocols are not associated with a higher proportion of simple weaning. A sub-analysis of the wind study

#### T. Pham^1,2,3^, G. Beduneau^4,5^, F. Schortgen^6^, L. Piquilloud^7^, E. Zogheib^8,9^, M. Jonas^10^, F. Grelon^11^, I. Runge^12^, N. Terzi^13,14^, S. Grangé^4^, G. Barberet^15^, P.-G. Guitard^16^, J.-P. Frat^17,18^, A. Constan^19^, J.-M. Chrétien^20^, J. Mancebo^21^, A. Mercat^22^, J.-C.M. Richard^23^, L. Brochard^24^, The WIND study group

##### ^1^CHU Tenon, APHP, Medical and Surgical ICU, Paris, France; ^2^University of Toronto, Interdepartmental Division of Critical Care, Toronto, Canada; ^3^Université Paris Diderot, Sorbonne Paris Cité, UMR 1153, Paris, France; ^4^Rouen University Hospital, Medical Intensive Care, Rouen, France; ^5^Rouen University Hospital, UPRES EA3830 IRIB, Rouen, France; ^6^CHU Henri Mondor, Medical ICU, Créteil, France; ^7^University Hospital of Lausanne, Intensive Care and Burn Unit, Lausanne, Switzerland; ^8^CHU d'Amiens, Cardiothoracic and Vascular ICU, Amiens, France; ^9^Université Jules Verne, Picardie, INSERM U1088 - CURS, Amiens, France; ^10^CHU Hotel Dieu, Medical ICU, Nantes, France; ^11^Hospital Le Mans, Intensive Care Unit, Le Mans, France; ^12^CHR d'Orleans, Medical ICU, Orleans, France; ^13^CHU Grenoble Alpes, Medical ICU, Grenoble, France; ^14^Université Grenoble-Alpes, Inserm U1042, Grenoble, France; ^15^CHR de Mulhouse, Medical ICU, Mulhouse, France; ^16^Rouen University Hospital, Surgical Intensive Care, Rouen, France; ^17^CHU de Poitiers, Medical ICU, Poitiers, France; ^18^Université de Poitiers, INSERM, CIC- 1402, Équipe 5 ALIVE, Poitiers, France; ^19^CHU Henri Mondor and ReVA Network, Medical ICU, Créteil, France; ^20^CHU d'Angers, Clinical Research Institute, Angers, France; ^21^Hospital de Sant Pau, Servei de Medicina Intensiva, Barcelona, Spain; ^22^CHU d'Angers, Medical ICU, Angers, France; ^23^Annecy Genevois General Hospital, Annecy, France; ^24^Saint Michael's Hospital and Keenan Research Centre, Interdepartmental Division of Critical Care, Toronto, Canada

###### **Correspondence:** T. Pham – CHU Tenon, APHP, Medical and Surgical ICU, Paris, France

**Introduction:** The majority of patients entering the weaning process from mechanical ventilation (MV) in the Intensive Care Unit (ICU) will have a short and simple weaning (SW) successfully terminated within 24 hours, while other may take up to one week (difficult weaning) or longer. Studies have shown that using a sedation or a weaning protocol could reduce the length of mechanical ventilation and the weaning duration.

**Objectives:** To describe factors associated with SW and particularly assess if sedation and weaning protocol are associated with the proportion of patients having a SW.

**Methods:** We used the data from the WIND (Weaning accordIng New Definition) study, a prospective multicenter observational study performed in France (29 ICUs), Spain (6 ICUS) and Switzerland (1 ICU) from April to August 2013. Ventilation and weaning modalities were daily assessed until discharge in all intubated patients admitted to the participating ICUs. We defined 1) weaning attempt (WA) as a spontaneous breathing trial (SBT) or an extubation attempt (with or without SBT), 2) successful weaning as an extubation without death or invasive mechanical ventilation within 7 days. We considered patients as having a SW if weaning was successfully terminated within 24 h following their first WA. Having a protocol for sedation or for weaning (or both) was asked to each center.

Quantitative and qualitative variables are presented as mean (standard deviation), median [interquartile range] or number (percentage) as appropriate. Comparisons of proportions were made using Chi2 or exact Fisher tests and continuous variables were compared using Student t-test or Wilcoxon rank sum test as appropriate. We performed a multivariable analysis of factors associated with SW by means of a logistic regression, forcing both sedation and weaning protocols in the final model. All statistical tests were two-sided and P values of 0.05 or less were considered significant.

**Results:** Among the 2729 patients included, 2051 patients entered the weaning process and we only kept in the present analysis the 1812 patients who did not have any decision of withholding or withdrawing mechanical ventilation. Among these 1812 patients, 1413 (78 %) had a SW and 399 (22 %) had a weaning duration longer than 24 hours. Main clinical characteristics are shown in Table 95.

**Conclusions:** In this study of 1812 patients with a daily assessment of the weaning process, hospitalization in an ICU using a sedation protocol or a weaning protocol (as declared by the center) was not associated with a higher proportion of patients having a simple and short weaning. Admission for planned surgery, younger age, lower SOFA score at admission and shorter duration of ventilation before any weaning attempt were associated with a higher proportion of simple and short weaning.

**Grant acknolwedgment**

This study benefited of a grant of the non-profit Association Départementale des Insuffisants Respiratoires (ADIR) of the Haute Normandie, France.

**Table 94 (abstract A1162).** Patients characteristics, mean ± SD or median [IQR]

	All N = 1812 included patients	Patients with a SW (N = 1413)	Patients with a longer weaning (N = 399)	p-value
Age, y	60 ± 16	58 ± 17	58 ± 17 65 ± 14	<0.001
SAPS II at admission, points	45 ± 17	44 ± 16	50 ± 18	<0.001
SOFA at admission, points	6.7 ± 3.5	6.4 ± 3.5	7.9 ± 3.5	<0.001
Admission:Medical/Planned/Unplanned surgery, n (%)	1280 (70.6 %)/256 (14.1)/276 (15.2)	968 (68.5 %)/231 (16.4)/214 (15.2)	312 (78.2 %)/25 (6.3)/62 (15.5)	<0.001/<0.001/0.91
Reintubation, n (%)	200 (11.0)	19 (1.3)	181 (45.4)	<0.001
Sedation protocol, n (%)	995 (54.9)	735 (52.0)	260 (65.2)	<0.001
Weaning protocol, n (%)	653 (36.0)	513 (36.3)	140 (35.0)	0.76
Delay from intubation to 1st WA, daysinvasive MV	3 [2;6]	3 [1;5]	6 [3;10]	<0.001
Death, n (%)	52 (2.9)	9 (0.6)	43 (10.8)	<0.001

**Table 95 (abstract A1162).** Multivariable analysis(factors associated with SW)

	OR	95 % IC	p-value
Planned surgery	2.27	1.124–4.78	0.024
Age (for 1 year)	0.98	0.97–0.99	<0.001
SOFA at admission (for 1 points)	0.94	0.91–0.97	0.003
Mechanical Ventilation days before the 1st WA, (for 1 day)	0.92	0.91–0.94	<0.001
Weaning protocol	0.84	0.65–1.09	0.198
Sedation protocol	0.72	0.39–1.32	0.576

### A1163 Ultrasonographically assessed excursions of the right hemi-diaphragm: an accurate predictor of weaning success

#### S. Prīdāne^1^, O. Sabeļņikovs^2,3^

##### ^1^Rīga Stradiņš University, Faculty of Continuing Education, Residency Section, Riga, Latvia; ^2^Rīga Stradiņš University, Department of Anaesthesiology & Intensive Care, Riga, Latvia; ^3^Pauls Stradins Clinical University Hospital, Department of Intensive Care, Riga, Latvia

###### **Correspondence:** S. Prīdāne – Rīga Stradiņš University, Faculty of Continuing Education, Residency Section, Riga, Latvia

**Introduction:** Many different tools are found to predict weaning success, but despite the wide usage of them there is still a high rate of unsuccessful liberation from the ventilator [1]. Ultrasonographically (US) assessed excursions of the right hemi-diaphragm could be a useful measurement for prediction success in weaning from mechanical ventilation (MV) [2].

**Objectives:** To compare the accurateness of ultrasonographically assessed excursions of the right hemi-diaphragm (DE) with other common weaning criteria.

**Methods:** A prospective observational study was conducted in patients undergoing weaning from MV in Pauls Stradins Clinical University Hospital ICU. US was performed after patient met weaning criteria (according to local protocol) and it was decided to discontinue MV. Patients with neuromuscular disorders and diaphragmatic paralysis were excluded. Measurements were performed once on Pressure Support Ventilation (PS ≤ 10cmH_2_O, PEEP ≤ 5cmH_2_O). The right hemi-diaphragms of patients were evaluated by M-mode ultrasonography (Esaote MyLabGamma AC2541 1–8 MHz convex probe). The average diaphragm excursions value (DE_avg_) was estimated from 3 sequential measurements. The rapid shallow breathing index (SBI), dynamic compliance (C_dyn_), minute ventilation (M_V_) and spontaneous tidal volume (V_T_ spont) were obtained from the ventilator (*Servo*^*i*^*, Maquet*). Unsuccessful weaning was defined as new onset of MV within 48 h after liberating from the ventilator.

**Results:** We analyzed 60 patients, 23 (38.3 %) of them failed weaning from MV. There were no significant differences between the successful weaning (SW) and unsuccessful weaning (UW) groups in baseline data (Table 97).

DE_avg_ and C_dyn_ differed significantly between SW and UW groups, but SBI, M_V_ and V_T_ spont didn't show significant difference between the groups (Table 98). (Data are shown as median [IQR])

DE_avg_ showed the highest discriminative power for predicting success in weaning from MV compared with SBI, C_dyn_, M_V_ and V_T_ spont (Table 99) with best DE_avg_ cut-off value 10.5 mm (sensitivity 89 % , specificity 99 %).

**Conclusions:** Our findings suggest that right hemi-diaphragm excursions assessed with M-mode ultrasonography is more accurate predictor of weaning success than other common weaning criteria.

**References**

1. Boles JM, Bion J, Connors A, Herridge M, Marsh B, Melote C et al. (2007) Weaning from mechanical ventilation. Statement of the Sixth International Consensus Conference on Intensive Care Medicine organized jointly by ERS, ATS, ESICM, SCCM and SRLF. Eur Respir J 29:1033–1056

2. Kim WY, Suh HJ, Hong SB, Koh Y, Lim CM (2011) Diaphragm dysfunction assessed by ultrasonography: influence of weaning from mechanical ventilation. Crit Care Med 39:2627–2630

**Table 96 (abstract A1163).** Baseline data

	Successful weaning (n = 37)	Unsuccessful weaning (n = 23)	P value
Gender (women n, %)	16 (57.1 %)	12 (42.9 %)	0.59
Age (mean, ±SD)	60 (±18.6)	67 (±16.3)	0.219
Length of MV (mean, ±SD)	9.59 (±7.2)	15.26 (±9.7)	0.062
PaO2/FiO2	293 (±62)	275 (±42)	0.18
pCO2 (mmHg, ±SD)	38 (±5)	37(±4)	0.89

**Table 97 (abstract A1163).** Group comparison

	SW (n = 37)	UW (n = 23)	P value
SBI (breaths/min/L)	56 [38–70]	56 [39–72]	0.885
VT spont (L)	0.44 [0.37–0.54]	0.43 [0.37–0.53]	0.909
MV (L/min)	9.4 [8.2–12]	11 [9–13]	0.112
DEavg (mm)	14.8 [8.2–12]	8.7 [7.9–9.5]	0.001
Cdyn (L/cmH2O2)	56 [46–65]	35 [29–47]	0.001

**Table 98 (abstract A1163).** AUC ROC values to predict weaning success

Test result variables	Area
SBI (breaths/min/L)	0.489
MV (L/min)	0.378
DE avg dx (mm)	0.948
Vt spont (L)	0.579
Cdyn (L/cmH2O2)	0.774

### A1164 Cycling-off guided by real-time waveforms analysis (IntelliSync+): pilot study on next-generation PSV

#### F. Mojoli, A. Orlando, I. Bianchi, F. Torriglia, S. Bianzina, M. Pozzi, G.A. Iotti, A. Braschi, PLUG Working Group

##### Fondazione IRCCS Policlinico S. Matteo, Anesthesia and Intensive Care, University of Pavia, Pavie, Italy

###### **Correspondence:** F. Mojoli – Fondazione IRCCS Policlinico S. Matteo, Anesthesia and Intensive Care, University of Pavia, Pavie, Italy

**Introduction:** Standard waveforms displayed on the ventilator screen may help detecting whether the ventilator is switching form inspiration to expiration at the right time, thus allowing appropriate setting of expiratory trigger sensitivity (ETS) [1].

**Objectives:** To test reliability and effectiveness of cycling-off guided by automated real-time waveforms analysis (IntelliSync+) available on G5 ventilator (Hamilton Medical, CH).

**Methods:** In 6 patients under PSV, IntelliSync + was compared to standard cycling-off with both default setting (ETS = 25 % of peak inspiratory flow) and optimized setting guided by bedside waveforms analysis of patient-ventilator interaction (PVI). Two levels of pressure support were tested: clinically set level (PS basal) and 50 % increase (PS + 50). ETS optimized at PS basal (ETS opti1) was selected as initial value at PS + 50 and then if necessary re-optimized (ETS opti2). Inspiratory trigger sensitivity was set at 2 l/min throughout the study.

**Results:** PS basal and PS + 50 were 12 ± 2 and 18 ± 3 cmH_2_O. ETS opti1 and ETS opti2 were 36 ± 8 (range 25–50) and 51 ± 13 % (range 35–70). Early cycling was not observed. Compared to default setting, ETS opti1 decreased cycling delay and unassisted efforts at PS basal, but these favorable effects were not maintained at PS + 50. Further optimization (ETS opti2) decreased cycling and trigger delay but did not affect unassisted efforts. When IntelliSync + was activated, cycling delay was shorter and values of trigger delay and unassisted efforts were at least as low as with optimized settings of ETS. Table 100 summarizes the results obtained in the 6 conditions tested.

**Conclusions:** Bedside optimization of ETS guided by waveforms on the ventilator screen improved PVI. Increase of pressure support level worsened PVI and mandated re-optimization of ETS. IntelliSync + performed better than default setting of ETS and at least as good as optimized setting.

**References**

1. Mojoli F et al. Intensive Care Med. 2015 [Epub ahead of print]

**Table 99 (abstract A1164).** Effects on patient-ventilator interaction of pressure support, optimization of ETS, and IntelliSync + .

	PS basal ETS 25	PS basal ETS opti1	PS basal IntelliSync+	PS + 50 ETS opti1	PS + 50 ETS opti2	PS + 50 IntelliSync+
Unassisted breaths (%)	12,4	3,5*	5,6*	12,1	10,8	5,6*
Trigger delay (ms)	238 ± 92	240 ± 89	209 ± 158**	295 ± 122**	265 ± 104	249 ± 117
Cycling delay (ms)	282 ± 315	113 ± 143***	54 ± 152****	304 ± 229	121 ± 134***	43 ± 149****

*p < 0.0001 vs. PS basal - ETS 25, PS + 50 ETS opti1 and PS + 50 - ETS opti2

**p < 0.0005 vs. all the other conditions

***p < 0.0001 vs. PS basal - ETS 25, PS + 50 ETS opti1, PS basal - Intellisync + and PS + 50 - Intellisync+

****p < 0.0001 vs. default and optimized settings of ETS

### A1165 Characteristics and factors associated with prolonged weaning. A sub-analysis of the wind study

#### G. Beduneau^1,2^, T. Pham^3,4,5^, F. Schortgen^6^, L. Piquilloud^7^, E. Zogheib^8,9^, M. Jonas^10^, F. Grelon^11^, I. Runge^12^, N. Terzi^13,14^, S. Grangé^1^, G. Barberet^15^, P.-G. Guitard^16^, J.-P. Frat^17,18^, A. Constan^19^, J.-M. Chrétien^20^, J. Mancebo^21^, A. Mercat^22^, J.-C.M. Richard^23^, L. Brochard^24^, The WIND study group

##### ^1^Rouen University Hospital, Medical Intensive Care, Rouen, France; ^2^Rouen University Hospital, UPRES EA3830 IRIB, Rouen, France; ^3^CHU Tenon, APHP, Medical and Surgical ICU, Paris, France; ^4^University of Toronto, Interdepartmental Division of Critical Care, Toronto, Canada; ^5^Université Paris Diderot, Sorbonne Paris Cité, UMR 1153, Paris, France; ^6^CHU Henri Mondor, Medical ICU, Créteil, France; ^7^University Hospital of Lausanne, Intensive Care and Burn Unit, Lausanne, Switzerland; ^8^CHU d'Amiens, Cardiothoracic and Vascular ICU, Amiens, France; ^9^Université Jules Verne, Picardie, INSERM U1088 - CURS, Amiens, France; ^10^CHU Hotel Dieu, Medical ICU, Nantes, France; ^11^Hospital Le Mans, Intensive Care Unit, Le Mans, France; ^12^CHR d'Orleans, Medical ICU, Orleans, France; ^13^CHU Grenoble Alpes, Medical ICU, Grenoble, France; ^14^Université Grenoble-Alpes, Inserm U1042, Grenoble, France; ^15^CHR de Mulhouse, Medical ICU, Mulhouse, France; ^16^Rouen University Hospital, Surgical Intensive Care, Rouen, France; ^17^CHU de Poitiers, Medical ICU, Poitiers, France; ^18^Université de Poitiers, INSERM, CIC- 1402, Équipe 5 ALIVE, Poitiers, France; ^19^CHU Henri Mondor and ReVA Network, Medical ICU, Créteil, France; ^20^CHU d'Angers, Clinical Research Institute, Angers, France; ^21^Hospital de Sant Pau, Servei de Medicina Intensiva, Barcelona, Spain; ^22^CHU d'Angers, Medical ICU, Angers, France; ^23^Annecy Genevois General Hospital, Annecy, France; ^24^Saint Michael's Hospital and Keenan Research Centre, Interdepartmental Division of Critical Care, Toronto, Canada

###### **Correspondence:** G. Beduneau – Rouen University Hospital, Medical Intensive Care, Rouen, France

**Introduction:** Patients with a prolonged weaning represent a small part of the total ICU population but this prolonged state has many implications on their later recovery and can highly impact health expenditures.

**Objectives:** To better characterize patients with prolonged weaning and assess factors associated with their survival.

**Methods:** The prospective multicentre observational WIND (Weaning accordIng New Definition) study was performed from April to August 2013. Ventilation and weaning modalities were daily assessed until discharge in all intubated patients admitted to the participating ICUs. We defined 1) weaning attempt (WA) as a spontaneous breathing trial (SBT) or an extubation (with or without SBT), 2) successful weaning as an extubation without death or invasive mechanical ventilation within 7 days. We considered patients as having a prolonged weaning if weaning was not terminated at 7 days following their first WA.

Variables are presented as mean ± standard deviation, median [interquartile range] or number (percentage). Comparisons were made using Chi2 test, exact Fisher tests, Student t-test or Wilcoxon rank sum test as appropriate. Logistic regression was used to assess factors associated with death. All statistical tests were two-sided and P value ≤ 0.05 were considered significant.

**Results:** Among the 2729 patients included, 2051 patients had at least 1 WA and 235 were still under mechanical ventilation 7 days after their first WA (11.5 % of the patients entering the weaning process). Their main characteristics and a comparison between survivors (70.2 %) and non survivors (29.8 %) are shown in Table 101. The majority (79 %) was admitted for a medical cause; they had a median number of WA of 3 [2–5] and 148 (63 %) had at least 1 extubation failure. Tracheostomy was performed in 66 patients (28 %) with a median time between intubation and the procedure of 22 [14; 32] days. Among survivors, 20 % of the total ICU length of stay and 30 % of the time after the 1^st^ WA was spent in the ICU without invasive mechanical ventilation.

Multivariable analysis showed that only immunodeficiency (OR = 2.5 [1.2;5.6]) and chronic cardiac failure (OR = 2.6 [1.2;5.6]) were associated with death.

**Conclusions:** In this multicentre international prospective cohort, 11.5 % of the patients entering the weaning process had a prolonged weaning with a high mortality rate of 29.8 %. The only baseline factor associated with death were previous immunodeficiency and chronic cardiac failure. These patients highly impact the ICU workload as they receive mechanical ventilation for a median duration of 19 days and their median length of stay in the ICU is 25 days. Patients with a prolonged weaning spend a long ICU time after the end of weaning without mechanical ventilation raising the issue of the need for specialized units.

**Grant acknolwedgment**

This study benefited of a grant of the non-profit Association Départementale des Insuffisants Respiratoires (ADIR) of the Haute Normandie, France

**Table 100 (abstract A1165).** Patients characteristics, mean ± SD or median [IQR]

	Prolonged weaning N = 235	Survivors N = 165	Deceased N = 70	p-value
Age, years	65 ± 13	65 ± 13	68 ± 13	0.09
COPD/Cardiac/Immunosuppression, N (%)	49(21)/33(16)/31(15)	32(22)/17(22)/16(11)	17(25)/16(23)/15(22)	0.85/0.054/0.060
SAPS II at admission,points	53 ± 18	51 ± 19	56.7 ± 17	0.036
SOFA score at admission, points	8.1 ± 3.7	7.8 ± 3.4	8.9 ± 4.3	0.047
Time from intubation to 1st WA, days	6 [3;10]	7 [3;10]	6 [3;10]	0.76
At least 1 reintubation, N (%)	148 (63.0)	106 (64.2)	42 (60.0)	0.54
Total number of days of invasive MV, days	19 [15;31]	18 [15;34]	20 [15;29]	0.85
Percentage of time spent in spontaneous ventilation (or NIV) after the 1st WA, %	23 [8;40]	18 [15;34]	8 [0;23]	<0.001
Length of stay in the ICU, days	25 [18;41]	26 [18;46]	22 [17;31]	0.02

### A1166 Ineffective effort events are associated with increased ICU stay and mortality

#### E. Kondili^1,2^, C. Psarologakis^2^, S. Kokkini^2^, V. Amargianitakis^2^, D. Babalis^1,3^, A. Chytas^4^, I. Chouvarda^4^, K. Vaporidi^1,2^, D. Georgopoulos^1,2^

##### ^1^University of Crete, School of Medicine, Intensive Care, Heraklio, Crete, Greece; ^2^University Hospital of Heraklio, Intensive Care, Heraklio, Crete, Greece; ^3^General Hospital of Larissa, Intensive Care, Larissa, Greece; ^4^Aristotle University of Thessaloniki, Lab of Medical Informatics, Thessaloniki, Greece

###### **Correspondence:** E. Kondili – University of Crete, School of Medicine, Intensive Care, Heraklio, Crete, Greece

**Introduction:** Ineffective efforts (IE), defined as the inability of patient's inspiratory effort to trigger a ventilator-delivered breath, is a commonly encountered asynchrony, and has been reported to adversely affect patient outcome1,2,3. The incidence of IE depends on several factors, including patient population, ventilator settings, and the observation period, which in most studies so far was limited2,3.

**Objectives:** Aim of this study was to investigate the incidence of ineffective efforts, using continuous recordings, in critically ill patients mechanically ventilated only on assisted mode and their potential effects on patient outcome.

**Methods:** 110 adult critically ill patients hospitalized in the ICU of the University Hospital of Heraklion on mechanical ventilation for >12 h were enrolled. Patients were studied when they were on assisted ventilation for >1 hour and expected to remain on assisted ventilation for the next 24 hours. Patients were studied again on the 3rd and 6th day if they remained on assisted ventilation. Continuous 24 h measurements were obtained using a monitor validated to identify ineffective efforts (PVI monitor)4. The output of PVI monitor data was processed before analysis to optimize data quality and re-sampled to a time-series with the number of IEs calculated in uniform intervals of 30secs while preserving the total number and duration of IEs5. The IE index was calculated as previously described. Because IE occurred in clusters, the concept of IE event was introduced, to describe variable periods of time containing IE > 10 % of breaths. IE events were characterized by their duration and power (number of IE)5.

**Results:** The analysis included 228 recordings corresponding to 2946 h of ventilation. The median IE index was 2.7 (1.2-6), 18 % of patients had IE < 1 %, and 14.5 % had IE > 10 %. There was a trend for higher mortality in patients with IE > 10 % (43 % vs. 27 % for IE < 10 %, p = 0.23, Fisher's exact test). In multivariate regression analysis, mortality was associated with age, OR = 1.039 (1.008-1.072), sepsis OR = 6.065(1.694-21.711) and mean event duration OR = 1.062 (1.014-1.113). Increased % of IE belonging to events, but not IE index was associated with increased ICU stay and duration of mechanical ventilation (p < 0.05, Kendall's tau).

**Conclusions:** this study, using continuous 24 h recordings, confirms the significance of IE and introduces the concept of IE event, a period of clustered IE. Characteristics of IE events, such as power and duration are found to be significantly associated with patient outcome.

**References**

1. Intensive Care Med (2015) 41:633–641

2. Intensive Care Med (2006) 32:1515–1522

3. Crit Care Med (2009) 37: 2740–2745

4. Intensive Care Med (2007) 33:1337–1346

5. Conf Proc IEEE Eng Med Biol Soc. 2015 Aug;2015:1963–6

**Grant acknowledgement**

Co-funded by the Horizon 2020 Framework Programme of the European Union under Grant Agreement n° 644906 'AEGLE'.

### A1167 Monitoring of electrical activity of the diaphragm shows failure of a spontaneous breathing trial earlier than protocol based parameters in prolonged weaning in non-communicative neurological patients

#### O. Trapp^1^, A. Kalenka^2^

##### ^1^Asklepios Schlossberg Klinik, Early Neurological Rehabilitation, Bad König, Germany; ^2^University Heidelberg, Anaesthesiology and Intensiv Care Medicine, Heppenheim, Germany

###### **Correspondence:** O. Trapp – Asklepios Schlossberg Klinik, Early Neurological Rehabilitation, Bad König, Germany

**Introduction:** During weaning from prolonged ventilation overload of diaphragm as main breathing muscle should be avoided. Clinical criteria are used for determining the end of the spontaneous breathing trial (SBT) in the context of a discontinuously concept for weaning. In addition the patients subjective feeling of breathing exhaustion plays an important role. In incommunicable patients lacks this possibility for feedback.Continuous monitoring of diaphragm electrical activity could give information of respiratory muscle effort during SBT.

**Objectives:** In tracheotomized patients undergoing prolonged weaning the relationship between the protocol-based definition of the end of a SBT and the course of the electrical activity of the diaphragm (EAdi) should be examined.

**Methods:** Prospective observation study conducted in a 30 beds intensive care unit in an early rehabilitation clinic. 29 patients that were not communicable because of stroke (17), cerebral hypoxaemia (5), traumatic brain injury (7) have been included. Using an EAdi-catheter usually applied in NAVA (neurally adjusted ventilatory assist)-Ventilation, peak of diaphragm electrical activity (Eadi peak) was continuously recorded 30 minutes before disconnection from ventilator up to 30 minutes after reconnection. The weaning protocol contained two possibilities for terminating of the SBT: reaching clinical signs of ventilation exhausting or reaching a previously fixed time limit.

**Results:** Median duration of mechanical ventilation at study start was 22 days and 37 days at successful weaning (28/29 patients, 1 died). 152 SBT have been recorded, 91 terminated because of exhaustion, 61 by time limit. Median duration over all was 244 minutes (exhaustion: 232/time limit: 315). With multiple regression analysis, the relationship between the duration of the SBT and the EAdi peak was examined. Looking at all SBT, which were terminated due to exhaustion, shows that the duration of the SBT has a highly significant impact on EAdi ( p < 0.0001). The mean increase of Eadi peak was 10.899 μV (absolute) and 1.446 (relatively). In SBT terminated because the time limit has been reached, there was no significant correlation between the time and course of Eadi peak.

**Conclusions:** Continuous recording of the electrical diaphragmatic activity during weaning of prolonged ventilation in incommunicable patients can be used as supplementary parameter in monitoring the respiratory function.

### A1168 Detection of patient inspiratory efforts by waveforms analysis: a step towards better patient-ventilator interaction

#### F. Mojoli, A. Orlando, I. Bianchi, F. Torriglia, S. Bianzina, M. Pozzi, G.A. Iotti, A. Braschi, PLUG Working Group

##### Fondazione IRCCS Policlinico S. Matteo, Anesthesia and Intensive Care, University of Pavia, Pavie, Italy

###### **Correspondence:** F. Mojoli – Fondazione IRCCS Policlinico S. Matteo, Anesthesia and Intensive Care, University of Pavia, Pavie, Italy

**Introduction:** Patient-ventilator asynchronies are associated with poor outcome. It was suggested that bedside analysis of ventilator waveforms may help detecting different types of asynchrony and setting properly the ventilator [1].

**Objectives:** To test accuracy of a “waveform” method, based on specific signs on airway pressure (Paw) and flow curves, in detecting spontaneous respiratory activity and asynchronies in patients under Pressure Support Ventilation (PSV).

**Methods:** 16 recordings (12 min each) of esophageal pressure (Pes), Paw and flow were obtained in obstructive (75 %) and restrictive (25 %) patients under PSV with clinical evidence of poor patient-ventilator interaction. Tracings of 4426 breaths were visually analyzed for detection of spontaneous respiratory activity both with Pes (reference method) and without Pes (waveform method) by different operators. Breaths were defined as assisted, unassisted or autotriggered, and assisted breaths as delayed triggered, early cycled or delayed cycled. The waveforms method was applied in a selection of tracings (20 min, 544 breaths) by 4 different operators for assessment of inter-rater agreement.

**Results:** The reference method detected 6 autotriggered (0.1 %), 976 unassisted (22.1 %) and 3444 assisted (77.8 %) breaths; among assisted breaths, 897 delayed triggered (26.0 %), 1231 delayed cycled (35.7 %) and 439 early cycled (12.7 %). Table 102 shows sensitivities and specificities (95 % CI) of the waveform method in evaluating patient-ventilator interaction. The waveform method detected the start of patient's inspiration and expiration with a bias of −23 and −32 ms and a precision (±1.98 SD) of 184 and 202 ms respectively. Absolute agreement among operators was almost perfect for unassisted breaths, strong for delayed triggered, delayed cycled and early cycled breaths, and weak for autotriggered breaths.

**Conclusions:** The waveforms method is a reliable, accurate and reproducible method to assess patient-ventilator interaction and could help optimal setting of the ventilator. Automation of this method may allow continuous monitoring of ventilated patients and/or improved breath triggering and cycling.

**References**

1. Mojoli F et al. Intensive Care Med. 2015 [Epub ahead of print]

**Table 101 (abstract A1168).** Accuracy of the waveform method in the assessment of patient-ventilator interaction

	Sensitivity (%)	Specificity (%)
Assisted breaths	99.8 (99.6–99.9)	99.9 (99.4–100.0)
Autotrigger	83.3 (35.9–99.6)	99.9 (99.7–100.0)
Unassisted breaths	98.3 (97.2–99.0)	100.0 (99.9–100.0)
Delayed trigger	76.8 (73.9–79.5)	90.1 (89.0–91.0)
Delayed cycling	89.1 (87.2–90.8)	83.8 (82.4–85.0)
Early cycling	93.2 (90.4–95.3)	99.6 (99.3–99.8)

### A1169 Measurement of neural times during mechanical ventilation by the derivative of the flow signal

#### J.A. Benítez Lozano^1^, P. Carmona Sánchez^2^, J.E. Barrueco Francioni^3^, F. Ruiz Ferrón^4^, J.M. Serrano Simón^2^

##### ^1^Hospital Quirón de Málaga, Intensive Care Unit, Málaga, Spain; ^2^Hospital Universitario Reina Sofía, Intensive Care Unit, Córdoba, Spain; ^3^Hospital de Manises, Valencia, Spain, ^4^Complejo Hospitalario de Jaén, Jaén, Spain

###### **Correspondence:** J.M. Serrano Simón – Hospital Universitario Reina Sofía, Intensive Care Unit, Córdoba, Spain

**Introduction:** The neural timing during mechanical ventilation can be obtained from conventional airway flow tracing, or invasive esophageal and gastric signal; however, it is difficult clinical practice and could be imprecise. The first derivative of airway flow signal show line segments with distinctly different slopes and with well-defined the inflections points, therefore this closely indicate the respiratory times, it can be calculated easily.

**Objectives:** To evaluate the accuracy of the derivative of the flow signal (DF) as method for measurement of the respiratory times compared with esophageal-gastric signals.

**Methods:** We studied a group de mechanically patients during the weaning time, at Pressure Support Ventilation (PSV) with different levels of assistance (High 15–23 cmH2O, medium 10–14 cmH2O, low 5–9 cmH2O). Esophageal, gastric, airway pressure, and airway flow were registered, samplig 278Hz. We determined the phase difference (Φ) relationships between the neuronal times obtained from derivative flow versus esophageal or gastric signal respect to machine cycle, by calculating the phase delay, dividing by the cycle time of ventilator*360°. Times (T) definitions: T0 = onset inspiratory effort, T1/2 = effort maximum. Data were analyzed by descriptive statistical methods and are expressed as mean ± SD, medians, interquartile range (IRQ, 25-75 % quartile), and coefficient of variation (CV). The comparisons were performed by Mann-Whitney Test. The relationships between measurement methods was examined using single linear regression and Bland-Altman analysis.

**Fig. 144 (abstract A1169).**
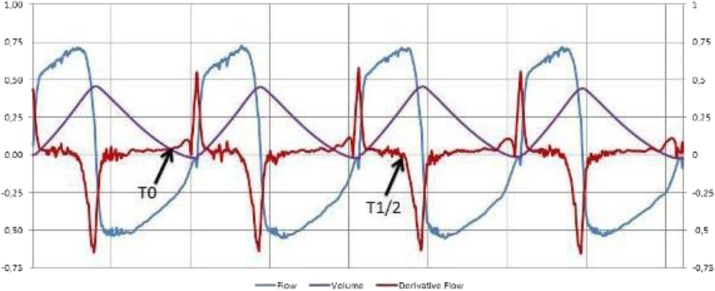
Neural times from the derivative flow signal

**Results:** 10 patients were studied. For all data angle phase Φ median (IRQ): T0: 1,29 (−5,06 to ,05), T1/2: −1,61 (−7,07 to 4,98). The mean comparison of T0 and T1/2 between Pes and DF did not showed statistical differences for any level of support, and correlation R^2^ > 0,99. The CV for all data at the T0 of Pes and DF: 11 % and 1 %, respectively; and for the T1/2 of Pes and DF: 50 % and 14 %, respectively; without differences between levels of assistance. Table 103 below show results from Bland-Altman analysis. Figure 144 show representative tracing of DF with well-defined inflection points (arrows) at T0 and T1/2, as the onset inspiratory flow and transition from inspiratory to expiratory flow.

**Conclusions:** The derivative of flow signal is useful to measure with accuracy neuronal and cycling times, it´s more homogeneous and precise than obtained for esophaeal or gastric pressure for all levels of assistance. The derivative of flow signal is a non-invasive signal which can be calculated easily and useful by conventional ventilator.

**Table 102 (abstract 1169).** Concordance analysis respiratory times Pes vs DF

	All data		High assist		Medium assist		low assist	
	T0	T1/2	T0	T1/2	T0	T1/2	T0	T1/2
Pes (mean, SD)	7,18 ± 4,86	5,00 ± 4,12	7.89 ± 5,56	5,31 ± 4,61	6,26 ± 3,81	3,48 ± 3,43	7,23 ± 4,87	5,13 ± 4,18
DF (mean, SD)	7,17 ± 4,87	5,04 ± 4,14	7,91 ± 5,55	5,40 ± 4,67	6,23 ± 3,79	4,48 ± 3,38	7,22 ± 4,90	5,15 ± 4,20
Bias (means differences)	−,003	,043	,026	,094	−,03	,0006	−,01	,02
2SD (Limits of agreement, CI 95 %)	−,204 to ,198	−,273 to ,359	−,23 to ,29	−,34 to ,53	−,19 to ,12	−,228 to ,229	−,12 to ,10	−,10 to ,14

### A1170 Inspiratory muscle function following pressure support advice by a decision support system from states of over- and under-support

#### S. Spadaro^1^, D.S. Karbing^2^, A. Gioia^1^, F. Moro^1^, F. Dalla Corte^1^, T. Mauri^3^, C.A. Volta^1^, S.E. Rees^2^, Plug working group

##### ^1^University of Ferrara/Intensive Care Unit, Morphology, Surgery and Experimental Medicine, Ferrara, Italy; ^2^Aalborg University, Respiratory and Critical Care group (rcare) Center for Model-based Medical Decision Support (MMDS) Department of Health Science and Technology, Aalborg, Denmark; ^3^University of Milan, Department of Anesthesia, Critical Care and Emergency Fondazione IRCCS Ca' Granda Ospedale Maggiore Policlinico, Milano, Italy

###### **Correspondence:** S. Spadaro – University of Ferrara/Intensive Care Unit, Morphology, Surgery and Experimental Medicine, Ferrara, Italy

**Introduction:** Providing appropriate levels of pressure support (PS) at the bedside is challenging. Physicians should avoid both over-support, which increases the risk of lung trauma, muscle atrophy and prolonged weaning; and under-support, which increases the risk of patient discomfort and respiratory muscle fatigue. The latter can be determined by the using the Tension Time index of the inspiratory muscles (TTi_es_) derived from measurement of esophageal pressure. TTi_es_ values higher than 0.18 indicate fatiguing patient effort. The Beacon Caresystem (Mermaid Care, Denmark) advises on level of PS using physiological models of lung mechanics, pulmonary gas exchange, respiratory drive, acid-base status and muscle function; along with clinical preference functions quantifying the risk of muscle atrophy, patient stress, and lung trauma. Mathematical models are tuned to measurements allowing advice to be patient specific.

**Objectives:** This study investigates the variation of TTi_es_ and other indices of respiratory muscle function induced by an increase/decrease of the level of PS, and whether the consequent advice proposed by the Beacon System results in appropriate patient effort.

**Methods:** Ten patients with acute respiratory failure residing in an ICU in Ferrara, Italy, have currently been included for this analysis. An esophageal balloon was inserted and its correct position determined by the occlusion test. The advice of the Beacon system was followed for an hour from states of over- and under-support defined as 150 % and 50 % of baseline PS. The level of PEEP was kept constant throughout the study. Data were analysed in terms of TTi_es_ and esophageal pressure developed in the first 100 ms of an occluded inspiration (P_0.1_)

**Results:** The baseline TTi_es_ values of 0.10 ± 0.05 were consistent with absence of fatiguing effort in all patients but one. As expected, reducing/increasing the level of PS resulted in TTi_es_ and P_0.1_increase or decrease, respectively. In 3 patients the reduction of PS was associated with impending muscle fatigue. The levels of PS proposed by the Beacon system resulted in TTi_es_ of 0.13 ± 0.01, slightly higher than obtained by the treating physician, but always below the values indicating muscle fatigue, a part from the patient in which the TTi_es_ indicated fatigue at baseline. Of note, this new value of TTies was not associated with a significant variation P_0.1_, which implies that the proposed level of PS was not associated with an increased respiratory drive or higher transpulmonary pressure

**Conclusion:** These initial results indicate that Beacon Caresystem responds appropriately to over- and under-support avoiding muscle fatigue and excessive P0.1.

### A1171 The use of vo2 level changes as a predictor for weaning success in the mechanically ventilated patients

#### M.V. Petrova, R. Mohan, A.V. Butrov, S.D. Beeharry, M.V. Vatsik, F.I. Sakieva

##### Peoples' Friendship University of Russia, Anesthesiology & Intensive Care, Moscow, Russian Federation

###### **Correspondence:** M.V. Petrova – Peoples' Friendship University of Russia, Anesthesiology & Intensive Care, Moscow, Russian Federation

**Introduction:** Recent studies have shown that prolonged mechanical ventilation (MV) is associated with adverse clinical outcomes such as a higher risk of ventilator associated pneumonia, lung injury & a high mortality at 28-35 % ^1,2^ thus, MV should be discontinued as soon as possible. Parameters such as VT, respiratory rate (RR), minute ventilation (V̇), vital capacity, maximal inspiratory pressure & RSBI were not suggested to be used as weaning success predictors (WSP) ^3,4^ moreover, little evidence exists on the use of CROP, IWI & CORE indices as WSP.

**Objectives:** To determine a predictor of weaning success with a faster reaction time than respiratory rate & pulse rate.

**Methods:** 15 patients (9 male, 6 female) on MV >21 days were included in our study diagnosed with sepsis (n = 2), pneumonia (n = 2), pancreonecrosis (n = 6), obesity hypoventilation syndrome (n = 2), intestinal obstruction (n = 3). Oxygen consumption (VO2) monitoring in different stages of MV support reduction was recorded using E-COVX indirect calorimeter gas analyzer, GE. Vital signs were monitored on CARESCAPE B650, GE. Weaning algorithm was as follows: 1) Ventilation mode was set in CPAP-PSV at a comfort pressure support (PS) level. 2) Baseline reading was recorded for 2 hours. 3) Stepwise reduction in PS by 2 cmH2O at different time intervals (10, 30, 60 min.) avoiding PS ≤ 8 cmH2o & ≤ 3 cmH2o for patients with ET tube and tracheostomy tube respectively. 4) Vitals, VO2, and V̇ & arterial blood gas (ABG) were recorded. If patient had active movement or tracheal suction was performed, VO2 was considered an artefact.

**Results:** It was noted that 10 of 15 patients were successfully weaned. VO2 in weaned patients increased in the early stage of PS reduction but returned to baseline levels. Also noted that Δ VO2 was 15 % ± 13 % & 22 % ± 23 % in weaned and non-weaned respectively, pulse rate (PR) 85 ± 11 & 94 ± 18 in weaned and non-weaned respectively & respiratory rate (RR) 22 ± 5 & 27 ± 6 in weaned and non-weaned respectively.

**Conclusions:** Evidently VO2 in weaned patients increased in the presence of a stable PR and RR but increase in VO2 in non- weaned patients was significant & followed by an increase in PR & RR which supports our initial hypotheses that changes in VO2 during weaning process could make a valuable predictor of weaning readiness because of its rapid reaction time compared to PR, RR and ABG.

**References**

1) Esteban A. et al.- Evaluation of mortality over time in patients receiving mechanical ventilation. 2013.

2) Protsenko DN, et al.- The use of Mechanical ventilation in intensive care unit in Russia: National epidemiological survey RUVENT- 2012.

3) Conti G, et al. A prospective, blinded evaluation of indexes proposed to predict weaning from mechanical ventilation. Inten Care Med 2004;30(5):830–836.

4) Tanios MA, et al. A randomized, controlled trial of the role of weaning predictors in clinical decision making. Crit Care Med 2006;34(10):2530–2535.

**Fig. 145 (abstract A1171).**
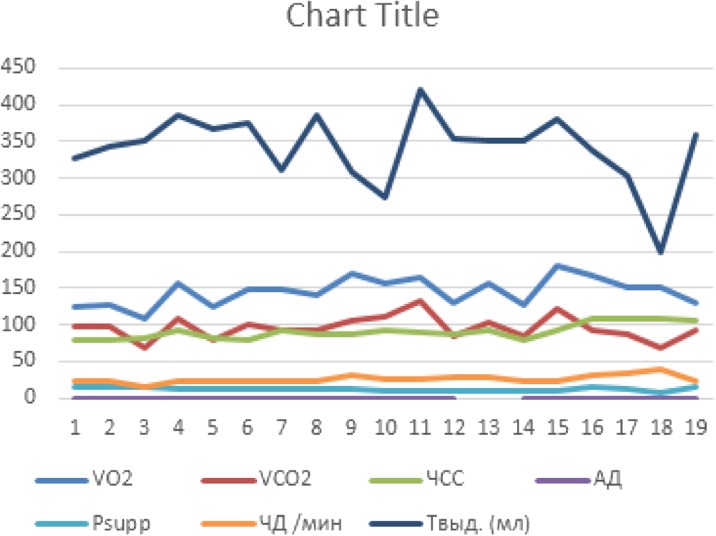
Unsuccessful

### A1172 An attempt to assess cough peak flow (CPF) using ventilator built-in flow-meter to predict extubation success: a single centre study

#### F. Gobert^1,2^, H. Yonis^1,2^, R. Tapponnier^1^, R. Fernandez^1^, M.-A. Labaune^1^, J.-F. Burle^1^, J. Barbier^1^, B. Vincent^1^, M. Cleyet^1^, J.-C. Richard^1,2^, C. Guérin^1,2,3^

##### ^1^Hospices Civils de Lyon, Hôpital de la Croix Rousse, Service de Reanimation Médicale, Lyon, France; ^2^Université de Lyon, Université Claude Bernard Lyon 1, Villeurbanne, France; ^3^INSERM, 955 Equipe 13, Créteil, France

###### **Correspondence:** F. Gobert – Hospices Civils de Lyon, Hôpital de la Croix Rousse, Service de Reanimation Médicale, Lyon, France

**Introduction:** Successful weaning from mechanical ventilation in ICU patients depends on patient ability to breathe spontaneously and on cough efficiency. Previous studies found that cough peak flow (CPF) at −60 L/min threshold predicted extubation failure. These studies measured CPF using a dedicated flow-meter that required patient disconnection from the ventilator, limiting the generalizability of this procedure. This study aimed to predict extubation outcome in a consecutive series of patients by measuring CPF from the ventilator flow-meter.

**Objectives:** The objective was to assess the performance of CPF to predict early extubation outcome.

**Methods:** We performed a prospective observational study in our 15-bed medical ICU from November 10^nd^ 2014 to October 30^st^ 2015. Inclusion criteria were: age > 18 years, intubation > 24 h, no withdrawal decision of life supporting care, eligible for scheduled weaning trial and then scheduled extubation, mechanical ventilation from Evita XL ventilator (Dräger, Germany) and patient's agreement to participate. Once daily checked criteria for weanibility were present, patients were switched to a standardised pressure support ventilation (inspiratory pressure = 7cmH2O, PEEP = 4 cmH2O, FIO2 = 0.40) for 1 h (if no chronic respiratory failure-CRF), 2 h (if CRF) or 12 h (if neuromuscular CRF). The procedure of CPF measurement was explained to the patient, who was encouraged to cough as strong as possible just before extubation. CPF measurements were done by freezing ventilator screen and scrolling the cursor to the maximal value of CPF during expiration and Tidal Volume (TV) in preceding inspiration. Three measurements were averaged. Early extubation success rate was defined as the proportion of patients who were alive and not reintubated 48 h after scheduled extubation. Median values were compared by using non parametric tests. Diagnostic performance of CPF and TV was assessed by using area under curve (AUC) of the ROC method. After having defined cut-off values for CPF and TV, we described the performance of a test combining CPF and TV values to predict the early extubation outcome.

**Fig. 146 (abstract A1172).**
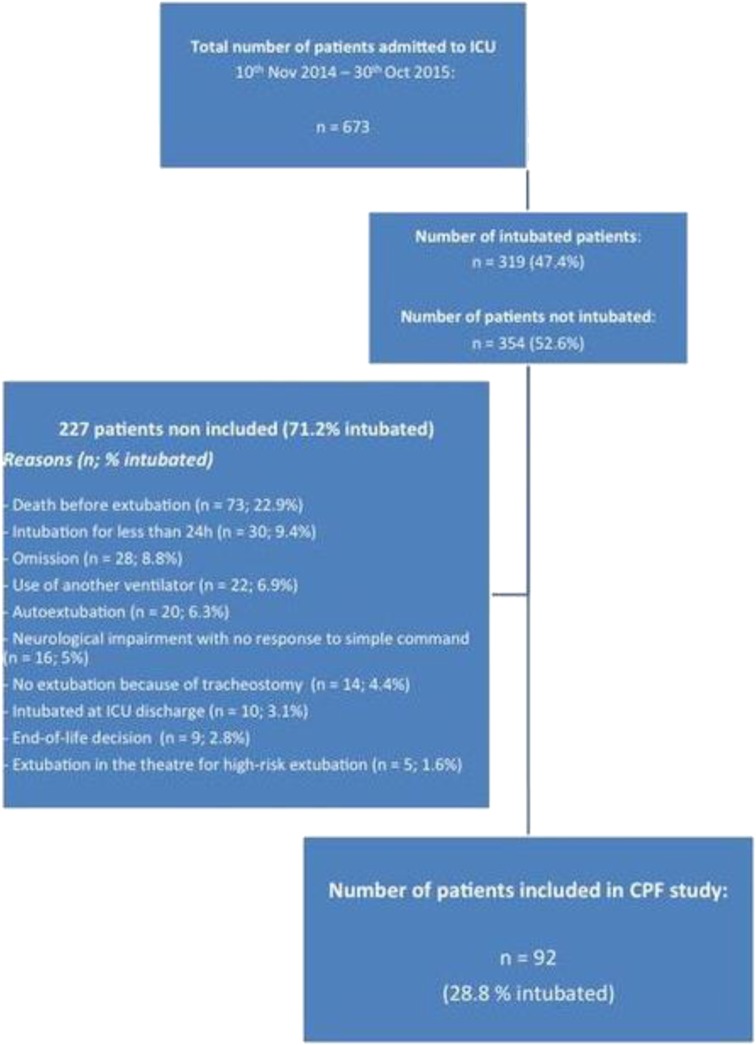
ᅟ

**Fig. 147 (abstract A1172).**
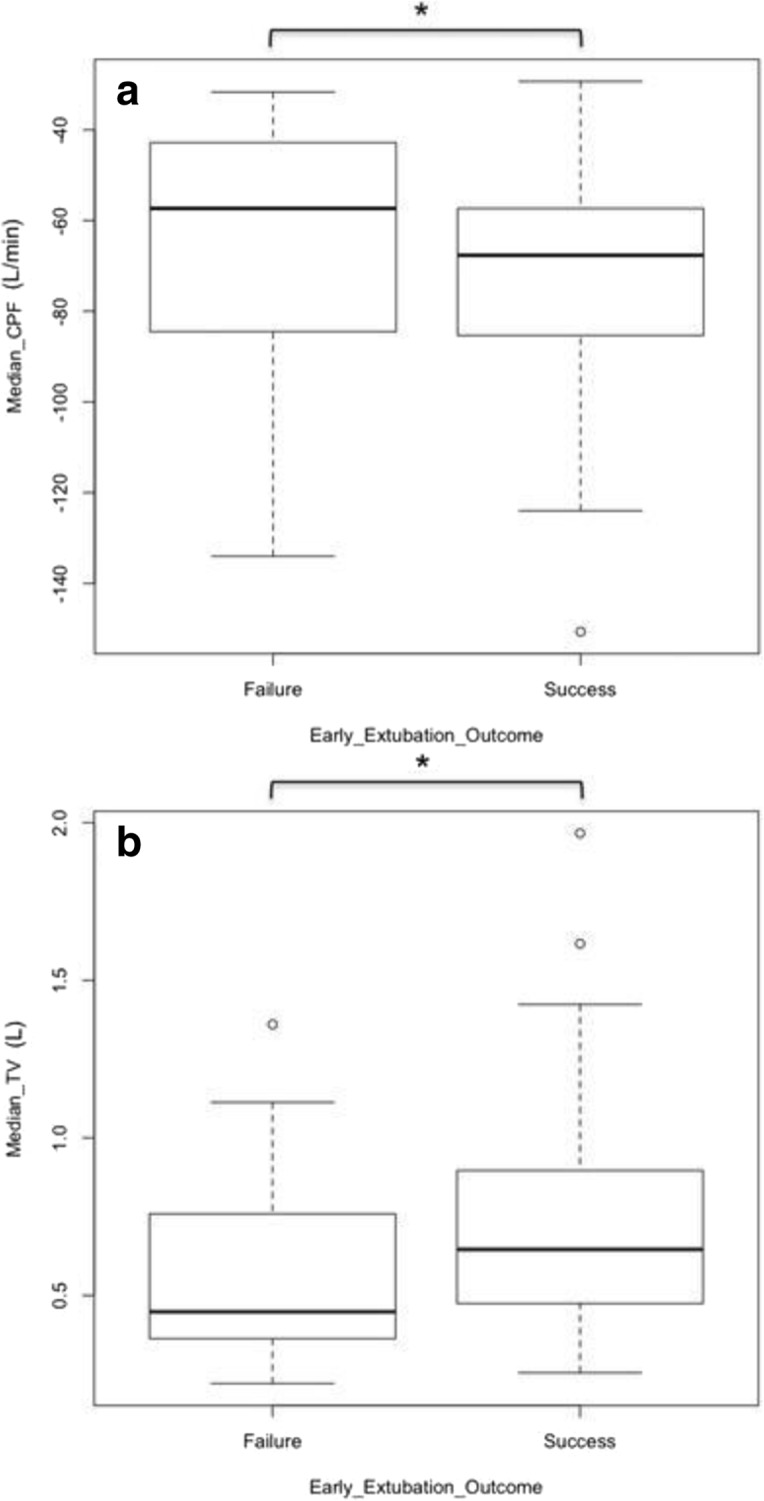
ᅟ

**Fig. 148 (abstract A1172).**
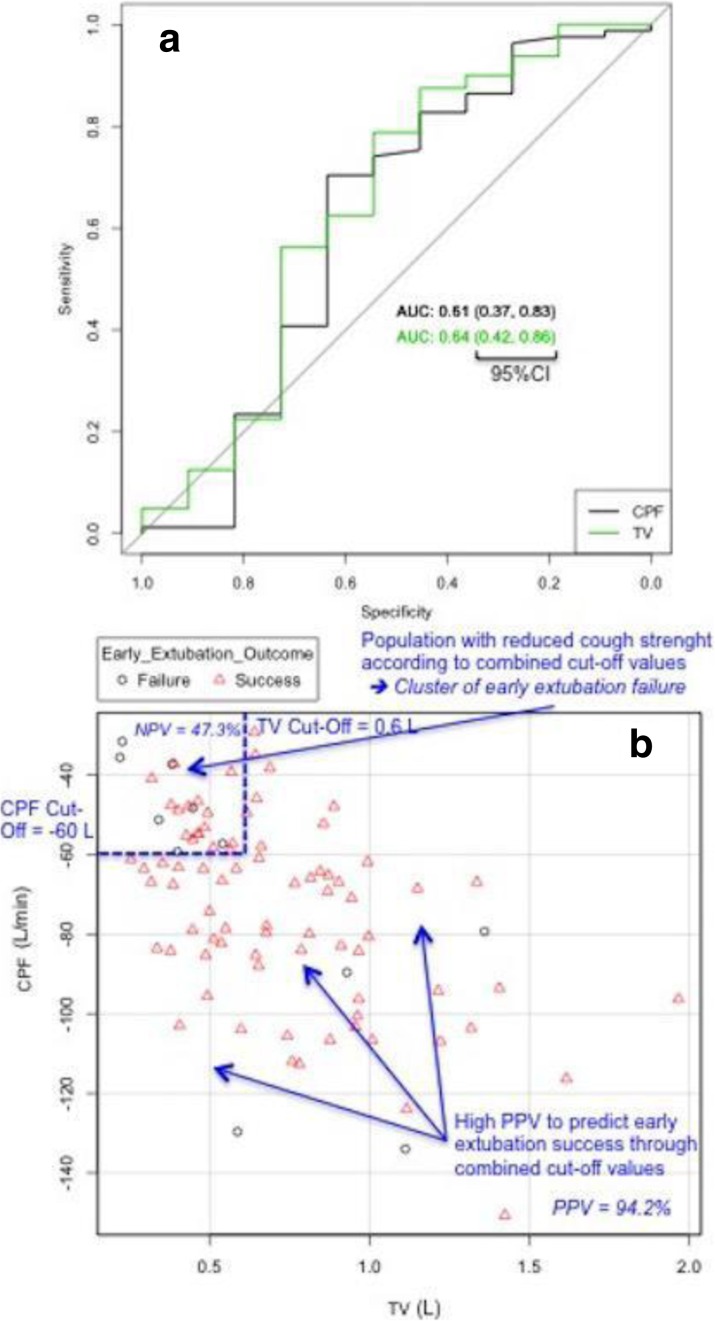
ᅟ

**Results:** During the study period, 673 patients were admitted to our ICU of who 319 were intubated and 92 patients included (Fig 146). Between the 81 patients who succeeded and the 11 patients who failed extubation, median CPF was −67.7 L/min and −57.3 L/min, respectively (p = 0.03, Fig 147A), median TV 0.646 L and 0.448 L, respectively (p = 0,078, Fig 147B), and AUC averaged 0.61 and 0.64, respectively (Fig 148A). Bi-dimensional analysis showed a synergistic effect of CPF and TV to predict early extubation success (Fig 148B). The combination of thresholds (CPF < −60 L/min and TV > 0.600 L) had a 94.2 % positive predictive value (PPV) and a 47.3 % negative predictive value for extubation success.

**Conclusions:** In the present study, measurement of CPF at the ventilator was feasible. Combined measures of CPF with TV reached a high PPV for early extubation success.

## SEPSIS AND PATHOPHYSIOLOGY OF AKI

### A1173 Role of the chloride anion in the development of acute kidney injury in septic patients

#### C. Righy Shinotsuka, J. Creteur, F.S. Taccone

##### Erasme University Hospital/Université Libre de Bruxelles, Department of Intensive Care, Brussels, Belgium

###### **Correspondence:** C. Righy Shinotsuka – Erasme University Hospital/Université Libre de Bruxelles, Department of Intensive Care, Brussels, Belgium

**Introduction:** Experimentally, hyperchloraemia may induce vasoconstriction of the renal afferent arterioles and tubular dysfunction, potentially resulting in acute kidney injury (AKI). The clinical implications of these findings are not well established, especially in septic patients.

**Objectives:** To investigate whether chloride serum and urinary concentrations as well as chloride load, output, balance and urinary anion gap are associated with the development of AKI in septic patients.

**Methods:** Retrospective analysis of an institutional database including all patients admitted to the Intensive Care Unit (ICU) for severe sepsis and septic shock from January 2011 to June 2015. Inclusion criteria were length of stay in the ICU ≥ 72 hours and complete data available on serum and urinary samples for at least 3 days. Patients were excluded if they had anuria on ICU admission, continuous bladder irrigation, if they were on hemodialysis (of recent onset and chronic) and if they were kidney-transplanted. Demographics and data on outcome were also analysed from the database. We collected chloride levels on daily blood (BCl) and urinary (UCl) analyses; chloride load (CL) was calculated by considering the amount of chloride present in the IV fluids administered daily to the patient, while chloride balance (CB) was calculated as: CL - CO, where CO is chloride output (UCl * daily urine output). Creatinine clearance (CrCl) was calculated on 24-hr urinary collection. AKI was defined according to standard criteria.

**Results:** Of the 288 patients who met the inclusion criteria, 240 were analysed. Median age was 62 [51–72] years and 151 (63 %) were male. ICU mortality was 30 % (n = 71). Two hundred and four patients (85 %) presented AKI on admission or developed this complication during ICU stay and 30 % (n = 62) of them needed continuous renal replacement therapy. Patients developing AKI had higher serum creatinine on admission (1.7 [1.3-2.6] vs. 0.6 [0.5-0.8] mg/dL; p < 0,0001) and lower 24-hr urine output (983 [501–1800] vs. 1601 [985–2363] mL; p = 0.0007), as well as lower CrCl (34.6 [14.8-65.6] vs. 141.7 [94.4-238.4] mL/min; p < 0,0001) compared to patients without AKI. Although the initial BCl (100 [96–104] vs. 101 [97–104] mEq/L) and CL (349 [199–562] vs. 375 [208–442] mEq) were similar between groups, patients with AKI had a lower UCl (33 [20–57] vs. 60 [20–92] mEq/L; p = 0.003) and CO (32 [12–75] vs. 100 [34–173] mEq; p = 0.0002) than patients without AKI. This resulted in a higher CB (284 [158–504] vs. 219 [48–357] mEq; p = 0.02). Urinary anion gap (48 [30–65] vs. 53 [25–90]) was similar between both groups.

**Conclusions:** Most of septic patients developed AKI and this complication was associated with a significant reduction in renal chloride elimination. The impact of such findings on the management of fluid therapy in this setting remains to be further evaluated.

### A1174 IL-6 trans-signaling in acute kidney injury in critically ill patients with severe sepsis

#### S. Törnblom^1^, S. Nisula^1^, S. Vaara^1^, M. Poukkanen^2^, S. Andersson^1^, V. Pettilä^1^, E. Pesonen^1^

##### ^1^University of Helsinki and Helsinki University Hospital, Helsinki, Finland; ^2^Lapland Central Hospital, Rovaniemi, Finland

###### **Correspondence:** S. Törnblom – University of Helsinki and Helsinki University Hospital, Helsinki, Finland

**Introduction:** Plasma interleukin 6 (IL-6) is associated with acute kidney injury (AKI) in sepsis. IL-6 receptor (IL-6R) is not expressed in the kidney. Circulating IL-6 in a complex with soluble IL-6R (sIL-6R) activates ubiquitously expressed transmembrane signal transducing glycoprotein 130 on renal epithelial cells. This IL-6 trans-signaling is associated with mortality in experimental sepsis.

**Objectives:** To study IL-6 trans-signaling in patients with sepsis in a clinical intensive care setting.

**Methods:** In 190 septic patients showing first organ failure at intensive care unit (ICU) admission ±24 hours, we measured plasma IL-6 and sIL-6R at admission and 24 hours later. Our primary endpoint was AKI during the first five ICU days by KDIGO criteria. Mann-Whitney's, Spearman's correlation and Chi Square tests were used.

**Results:** Plasma IL-6 was significantly higher in patients with AKI at 0 h (p = 0.001) and 24 h (p < 0.001). Plasma IL-6 correlated with KDIGO stage at 0 h (R = 0.214, p = 0.003) and 24 h (R = 0.361, p < 0.001). Plasma sIL-6R did not differ between AKI and non-AKI groups. Using cut-off values of 328 pg/ml of IL-6 and 53491 pg/ml of sIL-6R at 0 h (detected by Youden method), the combination of low IL-6 and low sIL6R was associated with non-AKI (p < 0.001).

**Conclusions:** Combination of low IL-6 and low sIL-6R in plasma is associated with decreased incidence of AKI, suggesting that IL-6 trans-signaling contributes to septic AKI.

**References**

1. Chawla et al. ClinJAmSocNephrol 2:22, 2007; Pallua et al. CritCareMed 31:1495, 2003; Barkhausen et al. CritCareMed 39:1407, 2011.

**Grant acknolwedgment**

The study has been supported by the Sigrid Juselius Foundation, Päivikki and Sakari Sohlberg Foundation, and Institutional Grants from the Helsinki University Hospital.

**Table 103 (abstract A1174).** Characteristics and laboratory results of patients

	All patients (N = 190) median [IQR] or n(%)	AKI (N = 99) median [IQR] or n(%)	No AKI (N = 91) median [IQR] or n(%)
Age (years)	62 [51.8–72]	64 [55–73]	61 [48–72]
Gender (male)	119 (62.6)	59 (59.6)	60 (65.9)
SAPS II score (points)	43 [34–52]	47 [37–55]	39 [31–47]
Highest SOFA score (minus renal points)	9 [7–11]	10 [7–11]	8 [5–10]
Septic shock	137 (72.1)	81 (81.8)	56 (61.5)
Plasma IL-6 0 h (pg/mL)	203.36 [53.72–934.6]	389.33 [73.1–1896.3]	111.36 [38.49–412.28]
Plasma IL-6 24 h (pg/mL)	100.08 [27.4–267.41]	173.62 [56.69–610.4]	53.08 [0–147.24]
Plasma sIL-6R 0 h (pg/mL)	36418.5 [25375–53855]	39380 [24608–59290]	35480 [25820–51810]
Plasma sIL-6R 24 h (pg/mL)	42920 [30446–54800]	44300 [29330–58099.25]	39810 [30860–52650]

### A1175 Relationship of blood pressure variability with acute kidney injury in sepsis

#### Z. Xie, X. Liao, Y. Kang, J. Zhang

##### West China Hospital, Sichuan University, Department of Critical Care Medicine, Chengdu, China

###### **Correspondence:** Z. Xie – West China Hospital, Sichuan University, Department of Critical Care Medicine, Chengdu, China

**Introduction:** Sepsis has been defined as organ dysfunction as a result of the inappropriate host response to infection.^[1]^ Renal function is often injured at the early stage of sepsis.^[2]^ Autoregulation, which plays an important role in maintaining an adequate renal blood flow against changes in blood pressure, could be impaired during sepsis,^[3]^ thus resulting in AKI if blood pressure fluctuates greatly.

**Objectives:** To investigate if there is any relationship between blood pressure variability (BPV) and AKI in septic patients.

**Methods:** Clinical data of patients admitted to our 50 bed medical ICU between 05/2014 and 03/2015 were reviewed. Continuous records of blood pressure were analysed. Blood pressure variability was calculated as the coefficient of variation (CV) of mean arterial pressure in the first 24 h of admission. AKI was defined by the KDIGO definition according to creatinine change and urine output criteria.^[4]^

**Results:** 275 adult patients with sepsis (age: 59.9 ± 17.3 years old; APACHE II score: 23.6 ± 7.1; male: 63.3 %) who stayed at ICU for more than three days were identified. AKI was presented in 70 (25.5 %) of them (stage 1: n = 33; stage 2: n = 15; stage 3: n = 22). The BPV was 12.0 ± 3.7 % for the patients with AKI versus 9.8 ± 3.2 % for the others (P < 0.001). ICU mortality was 41.4 % for the AKI group compared to 18.5 % for the non-AKI group (OR = 3.11; 95 % CI, 1.72 - 5.62, p < 0.001). Multivariate analysis indicated that the BPV was well associated with AKI (adjusted OR = 1.18; 95 % CI, 1.08 - 1.28, p < 0.001) while the mean blood pressure was not (adjusted OR = 0.99; 95 % CI, 0.95 - 1.02, p = 0.384).

**Conclusions:** Elevated blood pressure variability is associated with increased risk of AKI in septic patients. This understanding may be helpful to develop requirement for stabilising blood pressure in the BP management of septic patients.

**References**

1. Mervyn S, Clifford SD, Christopher WS, et al. The Third International Consensus Definitions for Sepsis and Septic Shock (Sepsis-3). *JAMA*,2016,315(8):801–810.

2. Uchino S, Kellum JA, Bellomo R, et al. Acute renal failure in critically ill patients: a multinational, multicenter study. *JAMA*,2005,294(7):813–818.

3. Julie B, Thierry B, Stephan E, et al. Relation between mean arterial pressure and renal function in the early phase of shock: a prospective, explorative cohort study. *Crit Care,*2011,15(3):R135.

4. Kellum JA,Lameire N. Diagnosis, evaluation, and management of acute kidney injury: a KDIGO summary (Part 1). *Crit Care*,2013,17(1):204.

**Grant acknolwedgment**

This study is supported by the National Key Technology R&D Program of China (No. 2012BAI11B05)

### A1176 Relationship between preoperative serum haptoglobin concentration and postoperative acute kidney injury in cardiovascular surgery patients with chronic renal impairment

#### K. Kubota, M. Egi, S. Mizobuchi

##### Kobe University Hospital, Anesthesiology, Kobe, Japan

###### **Correspondence:** K. Kubota – Kobe University Hospital, Anesthesiology, Kobe, Japan

**Introduction:** In critically ill patients with acute kidney injury (AKI), cardiovascular surgery is one of the most common diagnoses. AKI occurs in approximately 30 % of patients after cardiovascular surgery, and its incidence is known to be higher in patients with chronic renal impairment. During cardiac surgery, plasma free hemoglobin would increase due to hemolysis. Since plasma free hemoglobin is thought to be nephrotoxic, serum haptoglobin (sHp), which is a free hemoglobin scavenger, may have a protective role for postoperative AKI (pAKI). Thus, it is possible that a low preoperative sHp concentration increases the risk of pAKI. However, there has been no study in which the association between preoperative sHp concentration and incidence of pAKI was assessed.

**Objectives:** This study was a retrospective observational study to assess the association of preoperative sHp and incidence of pAKI in cardiovascular surgery patients with chronic renal impairment. We screened cardiovascular surgery patients requiring cardio-pulmonary bypass (CPB) from 2008 to 2013.

**Methods:** We included patients with preoperative estimated glomerular filtration rate (eGFR) < 60 mL/min/1.73 m^2^, because preoperative renal impairment is considered to be a risk factor of pAKI. We excluded patients who had descending aortic aneurysm replacement and those who required preoperative renal replacement therapy. AKI was diagnosed by AKIN criteria within 72 hours after the operation. We divided the patients into two groups by median of preoperative sHp, lower group (L group) and higher group (H group), and compared the incidences of pAKI in the two groups. Multivariate logistic regression analysis was performed to assess the independent association of preoperative sHp with incidence of pAKI.

**Results:** We included 110 patients. All patients were admitted in our intensive care unit postoperatively. Median preoperative sHp was 101 mg/dL, and the incidence of pAKI was 44.5 % (49/110). Median values of preoperative sHp were 46 mg/dL in the L group and 130.5 mg/dL in the H group. The incidence of pAKI was 53.7 % (29/54) in the L group and 34.0 % (20/56) in the H group (odds ratio: 2.1 (0.97-4.5), p = 0.059). After adjustment for confounders including Euroscore2, preoperative eGFR, and CPB duration, a lower level of preoperative sHp was independently associated with increased risk of pAKI (adjusted odds ratio: 2.3 (1.05-5.23), p = 0.037).

**Conclusions:** In patients with chronic renal impairment who undergo cardiovascular surgery requiring CPB, a lower level of preoperative sHp is independently associated with higher risk of pAKI.

**Table 104 (abstract A1176).** The association of lower serum haptoglobin concent

Co-variate	Adjusted odds ratio for low sHp (95 % C.I.)	p-value for low sHp
Low sHp	2.1 (0.97–4.5)	0.059
Low sHp + Euroscore 2	2.2 (1.02–4.9)	0.044
Low sHp + Euroscore 2 + preoperative eGFR	2.2 (1.02–5.0)	0.044
Low sHp + Euroscore 2 + preoperative eGFR + CPB duration	2.3 (1.05–5.2)	0.037

### A1177 Urinary angiotensinogen as a possible predictor of acute kidney injury in severe sepsis

#### S. Hegazy^1^, A. El-Keraie^2^, E. El Sayed^3^, M. Abd El Hamid^1^

##### ^1^Alexandria University, Critical Care Medicine, Alexandria, Egypt; ^2^Alexandria University, Internal Medicine-Nephrology Unit, Alexandria, Egypt; ^3^Alexandria University, Clinical and Chemical Pathology, Alexandria, Egypt

###### **Correspondence:** S. Hegazy – Alexandria University, Critical Care Medicine, Alexandria, Egypt

**Introduction:** Acute kidney injury (AKI) is a frequent and serious complication of sepsis in intensive care units (ICU). According to acute kidney injury criteria (AKIN), the most current diagnostic criteria for AKI is an abrupt (within 48 hrs.) reduction in kidney function currently defined as an absolute increase in serum creatinine of more than or equal to 0.3 mg/dl, or1.5 fold from baseline or a reduction in urine output (documented oliguria of < 0.5 ml/kg per hr. for >6 hr.). By time of occurrence of these criteria actual kidney insult has occurred & probably this leads to late intervention for kidney protection &/or renal replacement therapy (RRT). So early prediction of AKI by using biomarkers like urinary angiotensinogen could help patients to benefit from a quicker and more appropriate therapy. Urinary angiotensinogen appears quite promising due to its reported correlation with the intrarenal angiotensinogen and Angiotensin II levels which play a major role in molecular mechanisms of AKI.

**Objectives:** The aim of this work was to evaluate the role of urinary angiotensinogen as a possible predictor of AKI in patients with severe sepsis.

**Methods:** The study was carried on 100 adult patients who were admitted to the Department of Critical Care Medicine, at the Alexandria Main University Hospital and who suffered from severe sepsis. Patients were categorized into two groups according to AKI development; non AKI group which consisted of 30 patients (Group I), and AKI group which consisted of 70 patients (Group II). Patients were excluded if they have chronic kidney disease, already started RRT, received angiotensin convertase enzyme inhibitors (ACEI) or angiotensin receptor blockers (ARBS), or septically shocked. Urinary angiotensinogen and creatnine were withdrawn once from each patient on the day of admission to calculate Urinary angiotensinogen/creatinine ratio (uAnCR, ng/mg). AKIN staging was assessted daily for seven days.

**Results:** there was a significant difference between the two studied groups regarding uAnCR Ratio on admission (p < 0.001), whereas this difference was not statistically significant regarding creatinine level on admission (p = 0.317). Moreover, there was a positive correlation between uAnCR ratio on admission with AKIN staging and creatinine level of the all studied patient in the follow up days. The cutoff value of uAnCR on admission to predict later occurrence of AKI during ICU stay was 52.24 ng/mg: at this level, (88.57 % sensitivity and 53.30 % specificity).

**Conclusions:** Urinary angiotensinogen is a new promising biomarker in early prediction of AKI in patients with severe sepsis*.*

### A1178 Acute kidney injury in patients with severe sepsis or septic shock: a comparison between the “risk, injury, failure, loss of kidney function, end-stage kidney disease” (RIFLE), acute kidney injury network (AKIN) and kidney disease improving global outcomes (KDIGO) classificationsy

#### N.J. Rodrigues^1^, M. Pereira^1^, I. Godinho^1^, J. Gameiro^1^, M. Neves^1^, J. Gouveia^2^, Z. Costa e Silva^2^, J.A. Lopes^1^

##### ^1^Centro Hospitalar Lisboa Norte, Nephrology and Renal Transplantation, Lisbon, Portugal; ^2^Centro Hospitalar Lisboa Norte, Intensive Care Medicine, Lisbon, Portugal

###### **Correspondence:** N.J. Rodrigues – Centro Hospitalar Lisboa Norte, Nephrology and Renal Transplantation, Lisbon, Portugal

Sepsis is the leading cause of acute kidney injury (AKI) in Intensive Care Units (ICU). We aimed to compare the incidence of AKI according to RIFLE (Risk, Injury, Failure, Loss of kidney and End-stage kidney disease), AKIN (Acute Kidney Injury Network) and KDIGO (Kidney Dialysis Improvement Global Outcomes) classifications proposed for AKI, and the ability of these classifications in predicting in-hospital mortality in septic patients.

Retrospective analysis of 457 critically ill patients with severe sepsis or septic shock hospitalized between January 2008 and December 2014. Multivariate logistic regression was employed to evaluate the association between RIFLE, AKIN and KDIGO with in-hospital mortality. Model fit was assessed by the goodness of-fit test, and discrimination by the area under the receiver operator characteristic (AuROC) curve. Statistical significance was defined at a P < 0.05.

RIFLE (84.2 %) and KDIGO (87.5 %) identified more patients with AKI than AKIN (72.8 %) (P < 0.001, respectively). AKI defined by AKIN and KDIGO was associated with in-hospital mortality (AKIN - adjusted OR 2.3, 95%CI 1.3-4, P = 0.006; KDIGO - adjusted OR 2.7, 95%CI 1.2-6.2, P = 0.021) while AKI defined by RIFLE was not (adjusted OR 2.0, 95%CI 1–4, P = 0.063). The AuROC curve for in-hospital mortality was similar between the three classifications (RIFLE 0.652, P < 0.001; AKIN 0.686, P < 0.001; KDIGO 0.658, P < 0.001).

RIFLE classification had higher sensitivity than AKIN and KDIGO to detect AKI in critically ill septic patients; however their prognostic accuracy in terms of mortality was similar.

**Grant acknolwedgment**

None.

**Conflict of interest**

Nothing to declare.

### A1179 Acute muscle wasting quantified on routine ct imaging in complex pancreatitis icu patients and its relation to changes in creatinine

#### J. Mckinlay^1^, M. Kostalas^1,2^, G. Kooner^1^, G. Dudas^1,3^, A. Horton^4^, C. Kerr^4^, N. Karanjia^2,4^, B. Creagh-Brown^1,2^, L. Forni^1,2^

##### ^1^Royal Surrey County Hospital, ICU and SPACeR research group, Guildford, United Kingdom; ^2^University of Surrey, Guildford, United Kingdom; ^3^University of Semmelweis, Budapest, Hungary; ^4^Royal Surrey County Hospital, Guildford, United Kingdom

###### **Correspondence:** J. Mckinlay – Royal Surrey County Hospital, ICU and SPACeR research group, Guildford, United Kingdom

**Introduction:** Acute pancreatitis with organ dysfunction is termed severe acute pancreatitis (SAP) and complex SAP if local complications develop (such as infected pseudocyst). We receive tertiary referrals of complex SAP patients to our unit, who often have multiple CT scans. Muscle wasting is known to occur in critically ill patients (1) and can be quantified by measurement of the cross-sectional area (CSA) of para-spinal muscles at the third lumbar vertebral level on CT imaging. AKI is one of the most common causes of death in SAP patients (2) and is a risk factor for developing CKD (3). KDIGO guidelines suggest using creatinine changes to detect AKI (4) but creatinine changes may be inaccurate in the presence of muscle-wasting (myopenia) (3).

**Objectives:** To utilise measurements of L3 para-spinal muscle CSA (L3MCSA) from complex pancreatitis patients between April 2008-December 2014 and compare these to changes in plasma creatinine during their ICU stay.

**Methods:** Patients were identified from our ICU patient database (WardWatcher software) and additional clinical details including creatinine/eGFR level on CT-scan days, were acquired from electronic databases. Images were exported from our PACS system as DICOM files and analysed using ImageJ software (REF) in duplicate by two independent users, average values were used. For patients who had no renal-replacement therapy (RRT), between-scan L3MCSA and creatinine change were paired and analysis was with Excel (MS) and GraphPad (PRISM).

**Results:** 45 patients met inclusion criteria. 21 patients had ≥ 2 CT scans in ICU, enabling serial estimation of L3MCSA.

8/21 (38.1 %) patients did not have RRT in ICU. There was no statistically significant difference in overall (start to end of ICU) % change of L3MCSA between patients who did/did not have RRT. There was also no correlation between overall (start-to-end of ICU) % creatinine change and % change/day L3MCSA: r = −0.14, p = 0.75.

For between-scan data (n = 23): The median (IQR) % creatinine change/scan was −17.1 % (−9.0 to −44) and the % L3MCSA change/scan was −5.75 % (−1.8 to −9.8). However, there was no correlation between % L3MCSA change and % creatinine change between scans ( r = −0.14, p = 0.62).

**Conclusions:** L3MCSA (relating to lean muscle mass) was shown to decrease in complex severe acute pancreatitis (SAP) patients. However, there was no correlation with change in L3MCSA and change in creatinine. This suggests that normal/stable creatinine values may be falsely reassuring in the context of muscle mass loss (myopenia) and ongoing AKI could be under-diagnosed. Acknowledging myopenia and interpreting creatinine value in context is therefore vital.

**References**

1. JAMA 2013;310(15):1591

2. J Crit Care. 2010 Jun;25(2):225–9.

3. Clin J Am Soc Nephrol. 2014 Jun 6; 9(6): 1015–1023.

4. Kidney inter., Suppl. 2012; 2: 1–138

**Table 105 (abstract A1179).** Demographics

	All (n = 21)	No RRT (n = 8)	Had RRT (n = 13)
Age (yrs) Mean (s.d.)	53 (16.6)	57 (17.8)	50 (15.8)
APACHE II Median (IQR)	17 (13–21)	14.5 (12–19.8)	19 (14.5–23)
Length of Stay (days) Median (IQR)	40 (24–56)	34 (27.6)	43.9 (30.4–73.5)
ICU Mortality %	19.1	12.5	23.1

### A1180 Comparison of cytokine removal by AN69ST and PMMA membrane filters in a pig sepsis model

#### A. Yamazaki

##### Aomori Jekeikai Hospiyal, Clinical Engineer, Aomori, Japan

**Introduction:** Cytokine elimination during continuous hemofiltration (CHF) depends largely on the character of the filter membrane. Both AN69ST and polymethyl methacrylate (PMMA) membranes have strong adsorption capacity. Cytokines play important roles as the main mediators affecting critically ill patients. However, differences in the cytokine elimination by specific membranes during CHF have not yet been fully investigated.

**Objective:** The objective of this study was to determine the elimination of cytokines by AN69ST and PMMA membrane filters during CHF in a pig sepsis model.

**Methods:** Piglets (n = 7) weighing 20–30 kg were anesthetized and administered 30 μg/kg endotoxin. The Baxter sepXiris (AN69ST membrane) and the Toray Hemofeel 1.8 W (PMMA membrane) were used as hemofilters. Samples were taken at 1, 2, 4, and 6 hours after endotoxin administration, and the inlet plasma, outlet plasma, and filtrate concentrations of TNF-α, IL-1β, IL-6, and IL-8 were measured. Clearance values were calculated for each cytokine.

**Results:** Endotoxin administration induced increases in the inlet plasma concentrations of all cytokines measured. The AN69ST membrane filter showed higher adsorption and clearance of IL-8 than the PMMA membrane filter at 6 hours after endotoxin administration (AN69ST: 23.66 ± 17.01 mL/min; PMMA: −6.62 ± 48.89 mL/min; *P* < 0.05). However, the PMMA membrane filter showed higher adsorption and clearance of IL-1β than the AN69ST membrane filter. IL-6 did not appear in the filtrate of the PMMA membrane filter, while IL-8 was not eliminated in the filtrate of the AN69ST membrane filter. In addition, the filtrate concentration of TNF-α increased after its plasma concentration decreased with the PMMA membrane filter.

**Conclusions:** Shiga *et al*.^1^ previously reported the efficacy of cytokine absorption by AN69ST membrane filters during continuous hemodiafiltration, and Matsuda *et al*.^2^ reported the efficacy of cytokine absorption by PMMA membranes. However, the cytokine absorption efficacy by these two membrane filters had not been directly compared. The results shown here confirm that there are differences in cytokine adsorption by the AN69ST and PMMA membrane filters.

**References**

1) Shiga et al. Blood Purif 2014;38:211

2) Matsuda et al. Contrib Nephrol 2010;166:83

### A1181 Changes of antithrombin levels is the key factor in determining circuit lifespan during CRRT

#### M. Sanz Ganuza^1^, J.A. Martinez Molina^1^, F. Hidalgo Martinez^1^, M.T. Chiquito Freile^1^, N. Garcia Fernandez^2^, P. Medrano Travieso^1^

##### ^1^Clinica Universidad de Navarra, Anesthesia and Critical Care Unit, Pamplona, Spain; ^2^Clinica Universidad de Navarra, Nephrology, Pamplona, Spain

###### **Correspondence:** M. Sanz Ganuza – Clinica Universidad de Navarra, Anesthesia and Critical Care Unit, Pamplona, Spain

**Introduction:** Continuous renal replacement therapy (CRRT) is the most common therapy in critical ill patients with acute renal failure, having circuit coagulation as the most frequent complication. The CRRT circuit requires careful anticoagulation to avoid coagulation and bleeding complications. Critically ill patients with acquired antithrombin (AT) deficiency, may have a shorter filter lifespan.

**Objectives:** Evaluate the relation between the modification of AT levels from baseline and circuit survival during CCRT. We would like to determine the existence of an AT critical level, related to the risk of the clotting filter.

**Methods:** We started an observational study with prospective data collection in a university hospital. From October 2013 to April 2015, 61 patients were included, with 122 filters in total. We measured the level of AT activity at the beginning (basal AT), daily, and at the moment of circuit coagulation. We divided the patients in two groups depending in their AT´s basal level (<60 % or > 60 %). Then, we observed the percentage of change in AT from baseline, and we divided the patients in tertiles to obtain three comparable groups. The main outcome measure was filter lifespan of first circuit and the correlation with AT´s levels.

**Results:** Low AT´s basal level (<60 %) has significant association with longer filter life span (**p = 0.009**). We obtained three groups according to a percentage changes of ±8 % in AT from baseline. One group declined the AT´s basal level (8 % decrease), other had little changes (between 8 % decrease and 8 % increase) and the last one had an increase (8 % increase). The group which presented the highest percentual increase showed the largest median survival time to circuit coagulation (37 hours; 95 % CI: 26–48). We observed a significant association (**p = 0.008**) between the greater percentage change in AT from baseline, and a larger time intervals to circuit coagulation.

**Conclusions:** The circuit lifespan shows a narrow correlation with evolution of AT´s levels since the start of CRRT until filter clotting. AT measurement should be considered an essential factor during CRRT.

**References**

(1) (Kidney Disease Outcomes Quality Initiative. KDIGO Clinical Practice Guidelines for Acute Kidney Injury. Kidney Int Suppl. 2012;2:1–138.

(2) du Cheyron D, Bouchet B, et al. Antithrombin supplementation for anticoagulation during continuous hemofiltration in critically ill patients with septic shock: a case-control study. Crit Care 2006;10(2):R45.

(3) Lafargue M, Joannes-Boyau O, et al. Acquired deficit of antithrombin and role of supplementation in septic patients during continuous veno-venous hemofiltration. ASAIO J 2008 Jan-Feb;54(1):124–128.

### A1182 The influence of central line positioning on efficacy of intravenous noradrenaline treatment in hypotensive piglets during continuous renal replacement therapy

#### A. Bandert, R. Frithiof, M. Lipcsey, D. Smekal

##### Uppsala University, Surgical Sciencies, Uppsala, Sweden

###### **Correspondence:** A. Bandert – Uppsala University, Surgical Sciencies, Uppsala, Sweden

**Introduction:** Continuous renal replacement therapy (CRRT) in intensive care is a cornerstone in the supportive treatment arsenal. Its influence on thermodilution cardiac output measurements, and the possible influence of central venous dialysis catheter(CVDC) position, has been studied but the results are of uncertain clinical impact (1–3). There have been case reports describing the possibility of direct aspiration into a CVDC of drugs given in adherent central venous catheter(CVC) (4,5).

**Objectives:** The aim of this study was to investigate if different positions of central lines influence infused noradrenaline during continuous renal replacement therapy (CRRT) in an experimental animal model.

**Methods:** Ten anesthetized piglets received a CVC in the right jugular vein and two CVDCs (one via the same jugular vein as the CVDC and the other through a femoral vein). After randomization the CRRT was started in either one of the CVDCs and a nitroprusside infusion was started in an auricular vein. The dose was titrated until the mean arterial pressure (MAP) was 50 mmHg and then kept constant during the rest of the experiment. After reaching the intended blood pressure an infusion of noradrenaline was started and titrated with the goal of increasing the blood pressure to a MAP of 75 mmHg during 30 minutes. After a washout period the CRRT circuit was changed to the other CVDC and the experiment was repeated.

**Results:** The median dose of noradrenaline with the CRRT in the jugular vein was 0.34 (IQR 0.25) and in the femoral vein 0.14 (IQR 0.13) μg/kg/min (p = 0.021).

**Conclusions:** During CRRT, the noradrenaline dose needed to reach a target blood pressure in hypotensive piglets was twice as high with the CVC and CVDC close together, compared with CVC and CVDC on opposite sides of the diaphragm. This suggests that there is a possible clearance of noradrenaline and that the clearance is affected by catheter positioning

**References**

1. Sakka SG, Hanusch T, Theumer O et al. (2007) The influence of venovenous renal replacement therapy on measurements by the transpulmonary thermodilution technique. Anesth Analg 105(4):1079–1082

2. Schmidt S, Westhoff TH, Hofmann C (2007) Effect of the venous catheter site on transpulmonary thermodilution measurement variables. Crit Care Med 35: 783–786

3. Pathil A, Stremmel W, Schwenger V et al. (2013) The influence of haemodialysis on haemodynamic measurements using transpulmonary thermodilution in patients with septic shock: an observational study. Eur J Anaesthesiol; 30(1): 16–20

4. Strikker KH, Takala J, Hullin R et al. (2009) When Drugs Disappear from the Patient: eliminaiton of intravenous medication by hemodiafiltration. Anesth Analg 109: 1640–1643

5. Mohammad A, Zafar N, Feerick A (2010) Cardiac arrest in intensive care unit: Case report and future recommendations. Saudi J Anaesth 4(1): 31–34

**Grant acknolwedgment**

This study was funded by Uppsala University Hospital Research Fund and Swedish Research Council (grant 523-2014-2569)

### A1183 A first evaluation of omni, a new device for renal replacement therapy

#### P. Schlaepfer^1,2^, J.-D. Durovray^1,2^, V. Plouhinec^1^, C. Chiappa^1^, R. Bellomo^3^, A.G. Schneider^1^

##### ^1^Centre Hospitalier Universitaire Vaudois, Adult Intensive Care Unit, Lausanne, Switzerland; ^2^Centre Hospitalier Universitaire Vaudois, Anaesthesiology Department, Lausanne, Switzerland; ^3^University of Melbourne, Intensive Care Medicine, Melbourne, Australia

###### **Correspondence:** P. Schlaepfer – Centre Hospitalier Universitaire Vaudois, Adult Intensive Care Unit, Lausanne, Switzerland

**Introduction:** Several generations of devices have gradually improved the safety and feasibility of continuous renal replacement therapy (CRRT) to support critically ill patients with acute kidney injury (AKI). Omni® (B. Braun, Melsungen, Germany), a new third generation CRRT device has been designed with the aim of improving therapy accuracy and fluid balance management. Such improvements are thought to facilitate the achievement of the target renal dose and net fluid removal.

**Objectives:** We sought to evaluate the safety and feasibility of providing CRRT with Omni® in critically ill patients with acute kidney injury.

**Methods:** In a tertiary university affiliated hospital, we used Omni® to provide CRRT in ten critically ill patients. RRT was provided in CVVH mode with heparin anticoagulation or CVVHD mode with regional citrate anticoagulation. We collected patients' characteristics, filter life time, circuit pressures, interruption of therapy duration and reasons (alarm types), achieved and targeted renal dose, metabolic parameters (serum creatinine and potassium levels and arterial base excess). In addition, we administered a survey to all nurses providing the therapy to assess the ease of use and user interface of the device.

**Results:** RRT was applied using Omni® in CVVH-heparin mode in six patients (total duration 365.5 hours) and in CVVHD-citrate mode in four (total duration 249.7 hours). No major adverse events were observed and no therapy needed to be discontinued for safety concerns. Mean filter life was 22.8 hours (SD 14.2) in CVVH-heparin mode and 33.5 (SD 22.2) in CVVHD-citrate mode. Recirculation mode was successfully attempted in five patients (total duration 12.7 hours). Therapy interruption due to alarms corresponded to a total of 25.5 hrs (7 % of total therapy time) in CVVH-heparin mode and 11.9 hours

(4 % of total therapy time) in CVVHD-citrate mode. Mean achieved renal dose was 26.3 ml/kg/hr corresponding to 96 % of the targeted dose in CVVH-heparin mode and 29.8 ml/kg/hr corresponding to 98 % of the targeted dose in CVVHD-citrate mode. In both RRT modes, excellent metabolic control and adequate fluid balance were achieved. Overall, the interface, design and ease of use were evaluated by users as excellent.

**Conclusions:** CRRT in both CVVH and CVVHD modes could be provided using Omni® in a safe and efficient way in ten critically ill patients. Users provided positive feedback regarding therapy setup, management and user interface.

### A1184 Intermittent haemofiltration outside itu led by the intensive care team. Experience at a tertiary cardiothoracic centre

#### S. Mitchell, J. Durrant, H. Street, E. Dunthorne, J. Shears, C. Hernandez Caballero

##### Royal Brompton and Harefield NHS Foundation Trust, London, United Kingdom

###### **Correspondence:** C. Hernandez Caballero – Royal Brompton and Harefield NHS Foundation Trust, London, United Kingdom

**Introduction:** Due to the lack of conventional dialysis facilities in our centre, intermittent renal replacement therapies (IRRT) are led and performed by the ITU team. This team comprises a group of specialist outreach nurses with the support of intensivists. IRRT are performed nocturnally by ITU nurses in level 2 areas according to our Hospital policy.

**Objectives:** To describe the use and results of IRRT in level 2 areas in patients that have left ITU with established AKI. These therapies are directed and performed by specialist intensive care nurses with the support of the ITU medical team.

**Methods:** Retrospective observational study that included those patients admitted to level 3 areas at Harefield Hospital during 2015 that were transferred to level 2 areas still requiring IRRT. Demographic variables were collected, along with the indication and duration of IRRT and results.

**Fig. 149 (abstract A1184).**
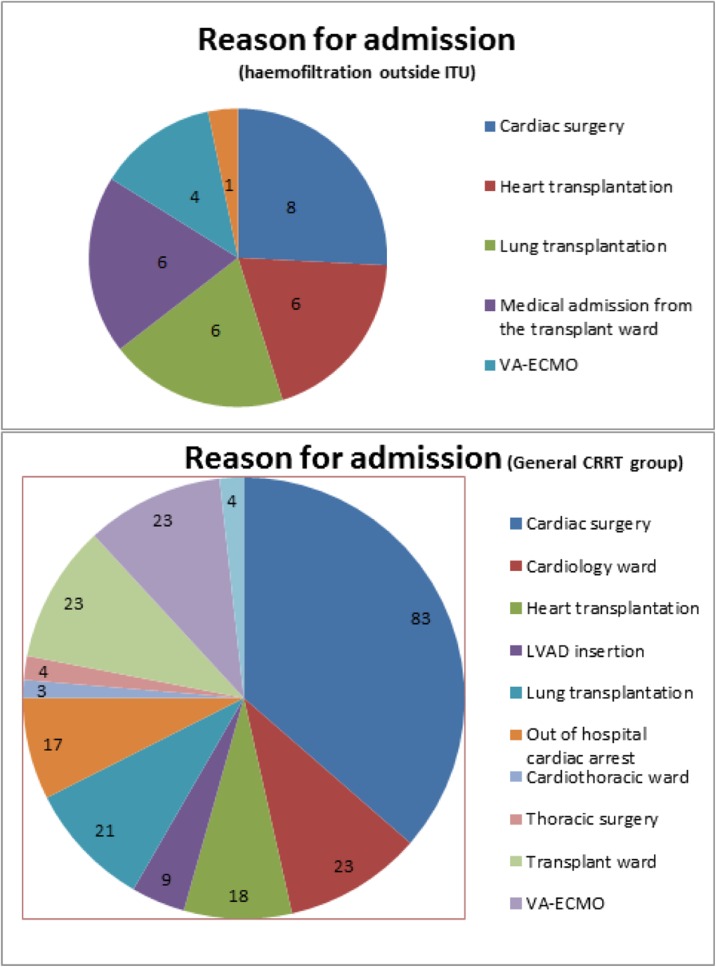
Reason for admission

**Fig. 150 (abstract A1184).**
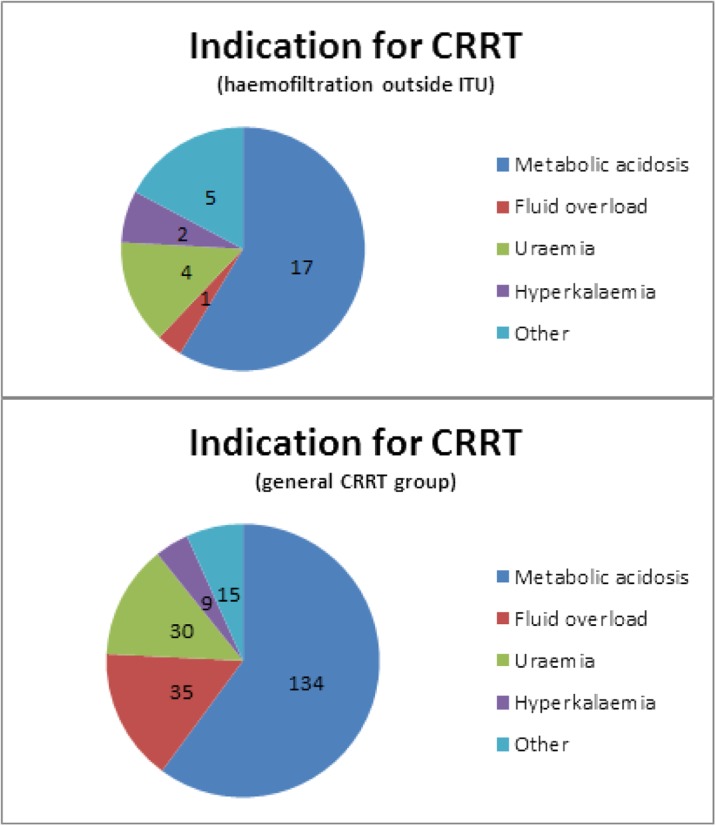
Indication for CRRT initiation

**Results:** 1829 patients were admitted to Harefield Hospital level 3 areas during 2015, of which 229 patients required continuous renal replacement therapies (CRRT). This population included patients admitted after cardiac and thoracic surgery, heart or lung transplantation, mechanical circulatory devices, out of hospital cardiac arrests (OOHCA) and medical admissions from the cardiology or cardio-thoracic surgical wards. Demographic variables were collected, along with the indication and duration of CRRT. 31 of those patients still required intermittent renal replacement therapies at their discharge to a level 2 area. 19 of them (61.3 %) were male and the group was a median age of 49.34 years. 4 of them (12 %) were hypertensive and 6 (19 %) were diabetic. As shown in Figure 149, the most frequent reason for admission to intensive care was cardiac surgery (25.8 %, 8 patients), followed by lung transplantation, heart transplantation and medical admissions from the transplantation ward. The reasons for admission to intensive care in the general CRRT group are also shown in Figure 149. The most frequent indication for initiation of CRRT was metabolic acidosis (54.8 %, 17 patients), followed by a combination of uraemia and fluid overload (16.1 %, 5 patients), uraemia (12.9 %, 4 patients) and fluid overload (3.2 %, 1 patients) as shown in Figure 150. The median time of RRT was 93 days days whilst the median time of filtration in the general RRT group was 13 days. The in-hospital mortality (after discharge from ITU) was 32.3 % and was 45.4 % in the general CRRT group. No complications were associated with the use of intermittent renal replacement therapies in level 2 areas.

**Conclusions:** The group of patients that required intermittent renal replacement therapies beyond their discharge from ITU had longer ITU and hospital lengths of stay. These therapies were performed safely in level 2 areas by the ITU team, allowing these patients to leave level 3 areas to continue their care.

**Fig. 151 (abstract A1184).**
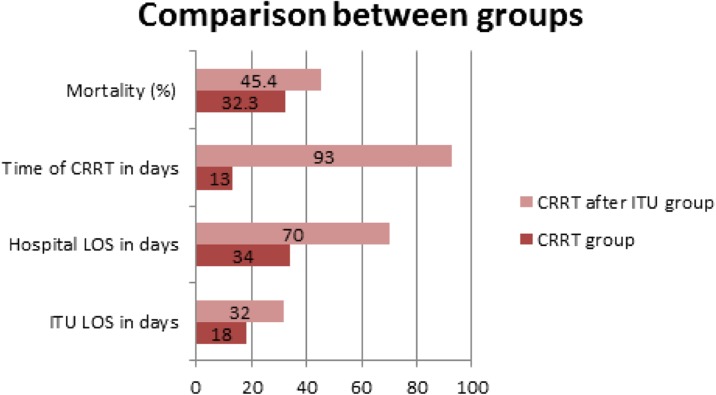
Results

### A1185 Implementation of citrate anticoagulation, for post dilution CRRT in patients contraindicated for heparin, results in acceptable filter life with low calcium requirement

#### R. Hutchison^1^, S. Schwarze^1^, S. Ghabina^1^, E. Thompson^2^, J.R. Prowle^1^, C.J. Kirwan^1^

##### ^1^Barts Health NHS Trust, Adult Critical Care, London, United Kingdom; ^2^Nikisso Medical, London, United Kingdom

###### **Correspondence:** R. Hutchison – Barts Health NHS Trust, Adult Critical Care, London, United Kingdom

**Introduction:** Regional citrate administration (RCA) is an effective anticoagulation technique for continuous renal replacement therapy (CRRT). Commonly, RCA protocols incorporate calcium free dialysate or replacement solution.

Following modification of the Aquarius haemofilter (Nikkiso), we designed and implemented a protocol for RCA with stand alone citrate administration pre filter (ACD-A (Acid Citrate Dextrose Formula-A) containing 113 mmol/L of citrate) and post dilution CVVHF using calcium containing replacement fluid (Accusol 35 containing 1.75 mmol/L Ca) and, when needed, supplementary calcium depending on systemic iCa. The protocol can deliver 25 or 35 mLs/kg/hr of CRRT.

We compare the efficacy of this new protocol, which we have initially implemented in patients with a relative contraindication to heparin, to a historical cohort of patients who received CRRT with prostacyclin and or pre-dilution CVVHF but would not have been contraindicated for RCA. We also present relevant biochemical data.

**Methods:** A prospective audit of the first 30 adult critically ill patients receiving RCA with post dilution CVVHF. Crude comparison was made with a historical group of consecutive critically ill patients who received CRRT without heparin prior to the introduction of the RCA protocol. Patients were excluded from the RCA protocol and the comparison if they had, severe acute liver injury. Data is presented as median (range) with non parametric analysis and filter survival as a Kaplan Meier for the event ´filter clotting´ and censored for medical cessation or technical failure.

**Results:** There were 113 filters used in 30 patients who received RCA and 73 in 24 in the comparison group respectively. One patient (14 filters) from the RCA group was excluded from the filter survival analysis due to a triglyceride level of 18.9 mmol/L, causing repeated filter failure.

Reasons for filter cessation are displayed in Table 106.

**Fig. 152 (abstract A1185).**
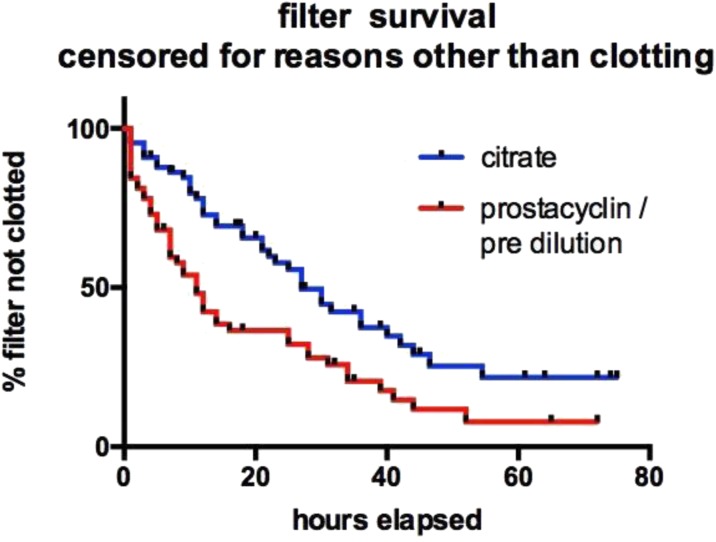
Filter survival censored for reasons other than clotting

Median (range) filter life was 21 (1–75), for RCA, versus 8 (1–72) (p = 0.0006) hours in the non heparin group. Time to filter failure due to clotting was significantly greater p = 0.0015 (Figure 152) in the RCA group.

Biochemical data for post dilution RCA is shown in Table 107.

Calcium supplementation was required with 34 filters (34 %) in 18 patients (60 %). In these patients, the median supplementary calcium dose (in addition to replacement fluid Ca) was 0.5 mmol/hr (0.023–1.39). 11 of those 18 were initiated with calcium with only 5 requiring further calcium in the next filter and 5 did not (1 patient who started on calcium only used 1 filter).

One patient in the citrate group was discontinued for alkalosis. No patients were discontinued for hypocalcaemia.

**Conclusions:** Post dilution RCA, using replacement fluid which contains calcium, in patients with a relative contraindication to heparin, reduces need for post filter calcium supplementation and provides acceptable filter life.

**Table 106 (abstract A1185).** ᅟ

	Citrate	Prostacyclin/pre-dilution
Patients	29	24
Filters	99	73
Reason for filter cessation		
Clotted	61	48
Access	5	1
Technical	6	3
Medical	11	10
Filter expired	5	2
Unknown	11	9

**Table 107 (abstract A1185).** ᅟ

Post Filter iCa (mmol/L)	0.39 (0.28–0.64)
Ca:iCa	1.9 (1.01–2.59)
pH (range)	7.11–7.53
Bicarbonate (mmol/L)	28.3–33.2
Sodium	130–155

### A1186 Mortality risk factors in continuous renal replacement therapy in a university hospital from Colombia

#### C.A. Gonzalez^1^, J.L. Pinto^2^, V. Orozco^3^, J.A. Patiño^3^, P.K. Garcia^1^, K.M. Contreras^1^, P. Rodriguez^1^, J.E. Echeverri^4^

##### ^1^Hospital Universitario San Ignacio, Internal Medicine, Nephrology, Bogota, Colombia; ^2^Pontificia Universidad Javeriana, Nefrology, Bogota, Colombia; ^3^Pontificia Universidad Javeriana, Internal Medicine, Bogota, Colombia; ^4^Hospital Militar Central, Nephrology, Bogota, Colombia

###### **Correspondence:** C.A. Gonzalez – Hospital Universitario San Ignacio, Internal Medicine, Nephrology, Bogota, Colombia

**Introduction:** Acute kidney injury (AKI) occurs in more than 50 % of critically ill patients, 23 % need renal replacement therapy, preferring continuous therapies. However mortality seems not to change with this technology. The research available focus on the right time to start therapy, but only evaluating renal dysfunction characteristics.

**Objectives:** To identify mortality risk factors at the start of continuous renal replacement therapy (CRRT) for acute kidney injury and early mortality risk factors in this patients.

**Methods:** A cohort study was performed in patients over 18 years old with AKI who required CRRT in the intensive care unit of a university hospital in Bogota Colombia between 2009 and 2014. The CRRT was provided with Aquarius® Edwards® technology, polyethersulfone membrane of 1.2 and 1.4 m2 (Aquamax®) and replacement fluids with lactate (Premixed®). Modality selection were guided by the hospital guideline. Sample size calculation was estimated selecting 10 cases (death) for each variable associated with mortality. A description of demographic and clinical variables was performed, bivariate analysis with mortality and early death defined as death within 24 hours of onset of CRRT, and finally we proceed to perform a multivariate prediction analysis. We considered statistically significant p value < 0.05.

**Results:** A total of 214 patients required CRRT during the period, 20 (9.34 %) patients were excluded, (age under 18 years old, incomplete data and 10 chronic kidney disease on dialysis). The mean age was 61.5 years (±15.47), 57.73 % men. The most frequent cause of AKI was sepsis in 30.9 % of cases. A total of 774 CRRT days were conducted with a median of 3 days per patient (range 1–19). Mean Charlson comorbidity index was 5.22 (±2.85), APACHE II score 29.65 (±6.66), total non-renal SOFA had a median of 11 (range 6–18) at the time of starting therapy. The hospital mortality was 68.4 % and early mortality was 19.07 %. In multivariate analysis: age (p = 0.004), SOFA (p = 0.038), days door- support (p = 0.03) and the presence of hypotension (p = 0.004) were independent risk factors for hospital mortality with an area under the curve of 0.76. For early death lactic acid levels (p = 0.007), glucosa (p = 0.01) and age (P = 0.02) were independent risk factors with an area under the curve of 0.73.

**Conclusions:** Patients with AKI on CRRT have high mortality. Age, multiple organ dysfunction, hypotension and time door-support were independent mortality risk factors. Low levels of glucose and high lactate at onset of CRRT are independent risk factors of early death.

**References**

1. Hoste EA, Bagshaw SM, Bellomo R, Cely CM, Colman R, Cruz DN, et al. Epidemiology of acute kidney injury in critically ill patients: the multinational AKI-EPI study. Intensive care medicine. 2015;41(8):1411–23

2. Villa G, Ricci Z, Ronco C. Renal Replacement Therapy. Critical care clinics. 2015;31(4):839–48.

